# Late-Stage Saturation of Drug Molecules

**DOI:** 10.1021/jacs.4c00807

**Published:** 2024-04-15

**Authors:** De-Hai Liu, Philipp M. Pflüger, Andrew Outlaw, Lukas Lückemeier, Fuhao Zhang, Clinton Regan, Hamid Rashidi Nodeh, Tim Cernak, Jiajia Ma, Frank Glorius

**Affiliations:** † Frontiers Science Center for Transformative Molecules, Shanghai Key Laboratory for Molecular Engineering of Chiral Drugs, School of Chemistry and Chemical Engineering and Zhangjiang Institute for Advanced Study, Shanghai Jiao Tong University, Shanghai 200240, P. R. China; ‡ Organisch-Chemisches Institut, Westfälische Wilhelms-Universität Münster, Corrensstraße 40, 48149 Münster, Germany; § Department of Medicinal Chemistry, University of Michigan, Ann Arbor, Michigan 48109, United States

## Abstract

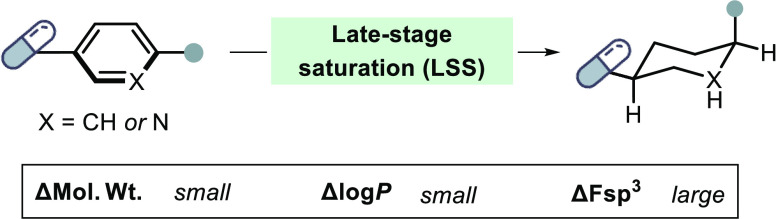

The available methods of chemical synthesis have arguably contributed to the prevalence of aromatic rings, such as benzene, toluene, xylene, or pyridine, in modern pharmaceuticals. Many such sp^2^-carbon-rich fragments are now easy to synthesize using high-quality cross-coupling reactions that click together an ever-expanding menu of commercially available building blocks, but the products are flat and lipophilic, decreasing their odds of becoming marketed drugs. Converting flat aromatic molecules into saturated analogues with a higher fraction of sp^3^ carbons could improve their medicinal properties and facilitate the invention of safe, efficacious, metabolically stable, and soluble medicines. In this study, we show that aromatic and heteroaromatic drugs can be readily saturated under exceptionally mild rhodium-catalyzed hydrogenation, acid-mediated reduction, or photocatalyzed-hydrogenation conditions, converting sp^2^ carbon atoms into sp^3^ carbon atoms and leading to saturated molecules with improved medicinal properties. These methods are productive in diverse pockets of chemical space, producing complex saturated pharmaceuticals bearing a variety of functional groups and three-dimensional architectures. The rhodium-catalyzed method tolerates traces of dimethyl sulfoxide (DMSO) or water, meaning that pharmaceutical compound collections, which are typically stored in wet DMSO, can finally be reformatted for use as substrates for chemical synthesis. This latter application is demonstrated through the late-stage saturation (LSS) of 768 complex and densely functionalized small-molecule drugs.

## Introduction

**Figure 1:**
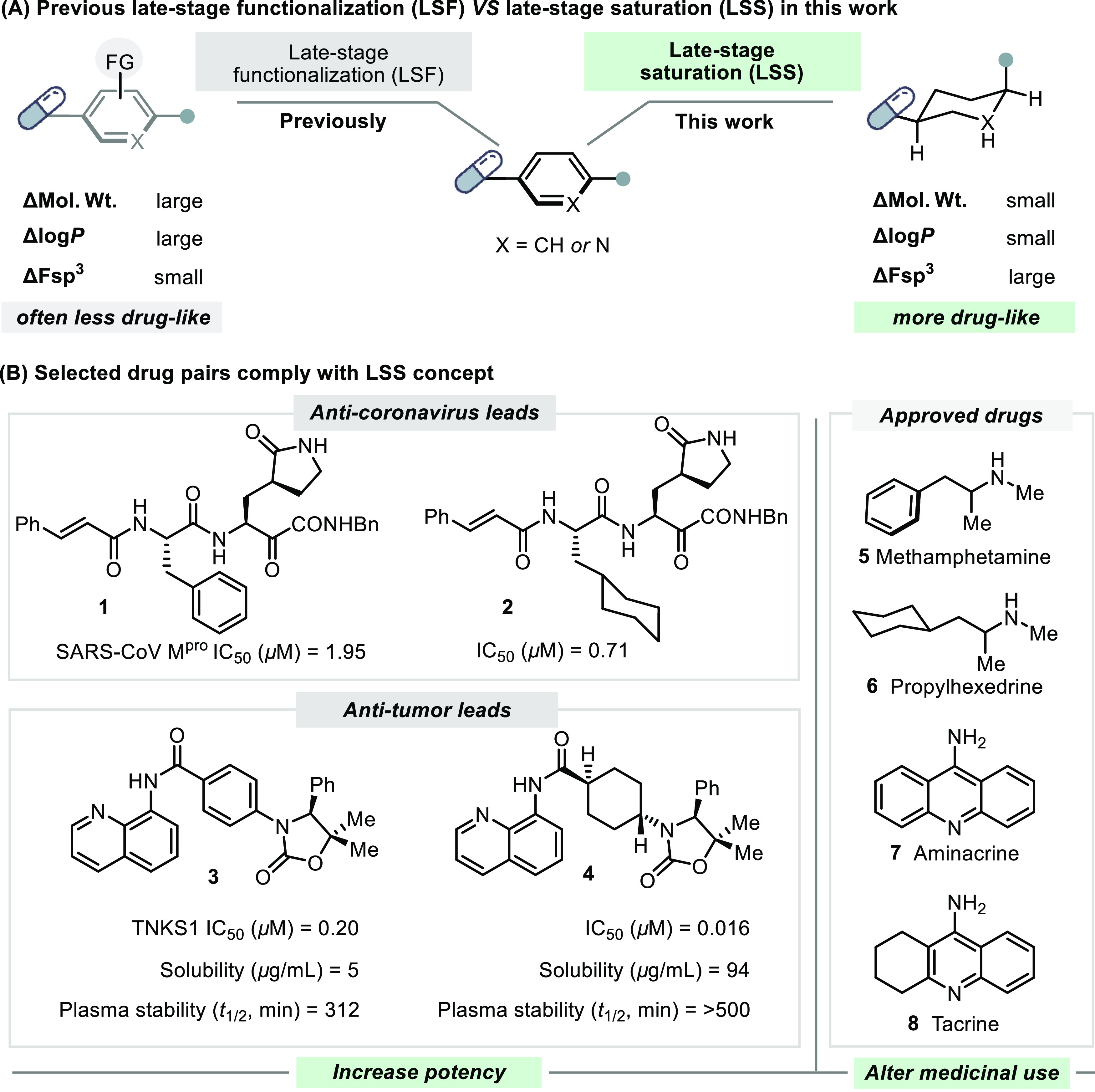
Late-stage saturation can improve drug properties. (A) The well-established late-stage functionalization (LSF) approach facilitates chemical space exploration around drug leads, but the overall drug properties of LSF analogues are often decreased due to the large increase in molecular weight (mol wt). Late-stage saturation (LSS) can markedly change physicochemical properties, most notably the fraction of sp^3^ atoms (Fsp^3^), while maintaining overall drug-likeness since only 6 hydrogen atoms are installed (Δmol weight = 6.04 g/mol); (B) examples of aromatic-saturated matched pairs of molecules with dramatically modified drug properties.

Medicines require a balance of properties to ensure their safety and efficacy. Aromatic molecules are comparatively easy to access synthetically, but a high degree of aromaticity has been shown to correlate with unfavorable outcomes such as poor solubility, selectivity, and metabolic stability.^1,2^ To address this issue, many recent reaction methods have sought to increase access to saturated molecules *viade novo* synthesis.^3,4^ Meanwhile, strategic advances in chemical synthesis now enable modification of complex molecular structures at a late stage for the navigation of chemical space and property space. For example, late-stage functionalization (LSF)^5^ decorates the periphery of complex molecules while emerging skeletal editing techniques^6^ manipulate shape and property space by altering a molecule’s backbone.^5,7−9^ Herein, we consider that late-stage saturation (LSS), where aromatic rings of complex drug substrates are saturated by dearomative hydrogenation, could be a valuable addition to the drug hunter’s toolbox (Figure 1a). Most drugs contain at least one aromatic moiety, with benzene (74.4%) and pyridine (10.8%) rings accounting for the vast majority of aromatic 6-membered rings in drugs in the PubChem database (see Supporting Information Figure S3).^10^ Escaping from flat aromatic structures toward saturated molecules with a higher fraction of sp^3^ atoms (Fsp^3^) has been invoked as a drug design strategy to improve clinical outcomes,^1,2^ solubility,^11^ selectivity for target proteins, and efficacy.^12^ Drug candidates with a high Fsp^3^ are desirable, yet the chemical synthesis of saturated analogues, potentially with multiple stereocenters, is often less facile than stitching together aromatic building blocks. Recognizing that the three most popular reactions used in pharmaceutical research—the amide coupling, Suzuki coupling, and Buchwald–Hartwig coupling^13^—can easily click together aromatic building blocks, LSS would be a powerful complementary method. A successful LSS strategy would require mild reaction conditions with high functional group tolerance and robust substrate scope to be impactful while obviating the need for autoclaves or other special hydrogenation equipment to facilitate adoption by medicinal chemists in diverse academic and industrial settings.

Drug discovery campaigns often seek to “escape from flatland” toward analogues with a higher Fsp^3^.^1,2,11,12^ As an example (Figure 1b), the partially saturated lead compound **2** is a SARS-CoV M^pro^ inhibitor that is more than twice as active as its aromatic congener **1**.^14,15^ Similarly, a saturation-induced potency increase was observed between TNKS1 inhibitors **3** and **4**, wherein other drug properties including plasma stability and aqueous solubility simultaneously improved upon saturation.^16^ Furthermore, several approved drugs have a saturated analogue with an entirely different medicinal use. For instance, methamphetamine (**5**) is a scheduled substance and stimulant, while its saturated analogue, propylhexedrine (**6**), is a topical vasoconstrictor. Aminacrine (**7**) is a topical antiseptic while its partially saturated congener, tacrine (**8**), is approved to treat dementia.

## Results and Discussion

### Data-Guided Validation of the LSS Concept

**Figure 2:**
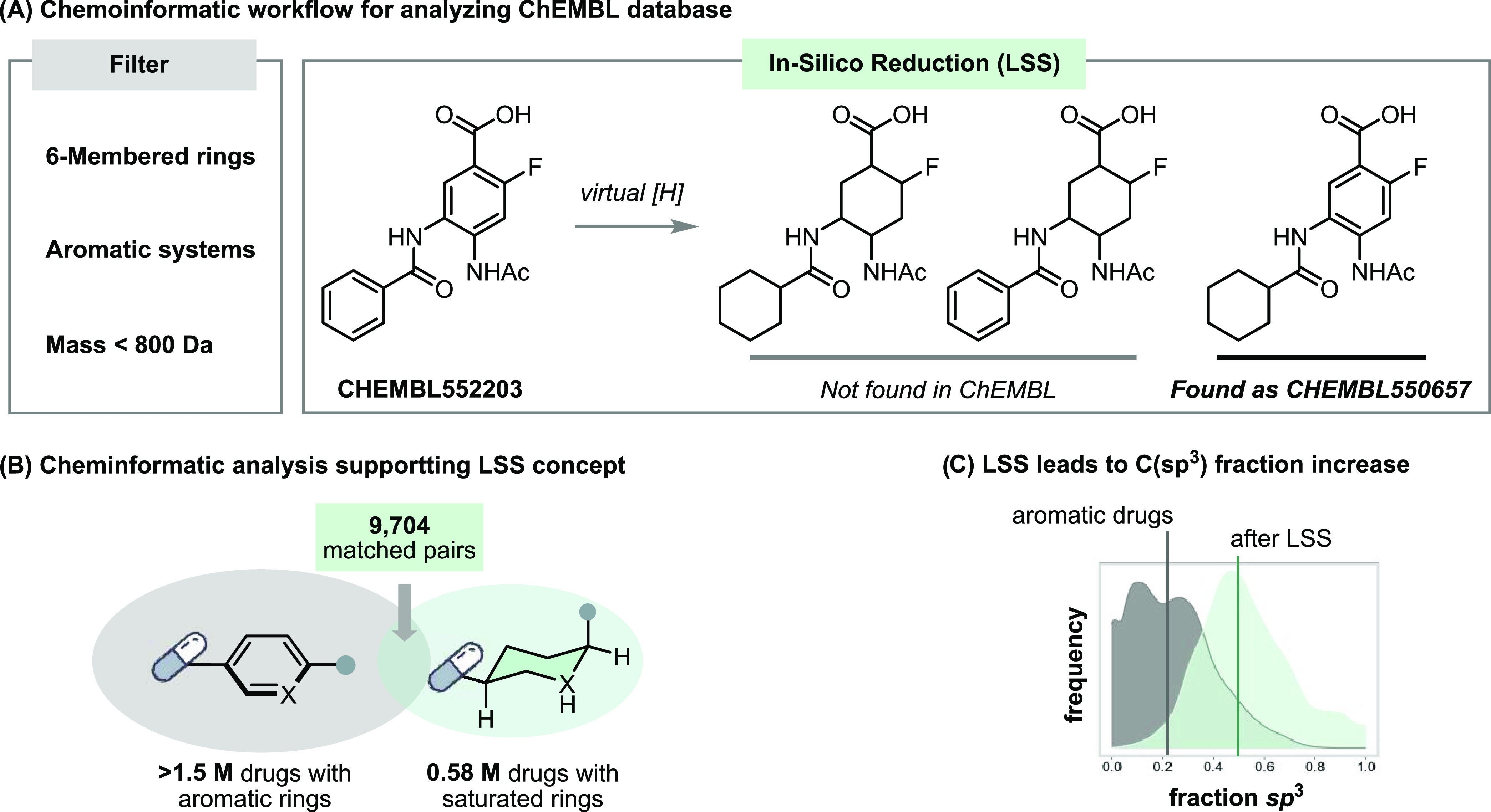
Data-guided validation of the LSS concept. (A) Cheminformatic workflow for analyzing the ChEMBL database. (B) The resulted 9704 compound pairs show that aromatic-saturated matched pairs exist, but those available were generally accessed by total synthesis. (C) Kernel density of the fraction of sp^3^ atoms showing the change in this property upon performing a virtual LSS (green) of the aromatic drugs (gray) in the ChEMBL database. For details of cheminformatic analysis, see Supporting Information, Section 5.

To statistically explore the direct impact of saturation on aromatic drugs, we performed a cheminformatic analysis within the ChEMBL database of 2.1 M druglike molecules,^17^ to highlight the impact of the LSS strategy (Figure 2). First, virtual compounds were filtered to include only substrates having 6-membered aromatic rings, leaving over 1.5 M compounds, and highlighting that current bioactive molecular space consists mainly of planar, C(sp^2^)-rich compounds. Next, we performed *in silico* saturation on each aromatic ring, resulting in a virtual library of saturated products. A matched-pair analysis identified 9,704 pairs of compounds in ChEMBL for which there was an aromatic compound and an exact analogue wherein the only structural change was saturation of the aromatic ring. Most pairs (88.0%) comprised benzene-cyclohexyl analogues, with the second-most popular pyridine-piperidine analogue pairing accounting for a much smaller proportion of the library (5.9%) (see Supporting Information Figure S3a–b). This is somewhat surprising since drugs containing piperidine rings are twice as prevalent as those containing pyridine.^18^ This discrepancy may be explained by a follow-up observation that most saturated drugs among the 9704 pairs were accessed by resource-intensive total synthesis, challenging their facile production and highlighting the need for a practical LSS technology. Specifically, we concluded from these data that a method to reduce benzene and pyridine rings would be one of the most impactful starting points for LSS. Also, LSS can in principle access multiple regio- and stereoisomers, and this combinatorial explosion of permutations (see Supporting Information Figure S3c) is a viable computational approach to generate ultralarge virtual libraries, which can increase the quality of lead compounds in computer-aided drug discovery.^19^ Although the reduction of benzenoid rings will lead to a considerable increase in Fsp^3^ and a modest predicted contribution to partition coefficient (log *P*), the saturation of heteroarenes may have a dramatic impact on compound properties, as for instance when the reduction of a pyridine ring generates a piperidine that is several orders of magnitude more basic and with an additional hydrogen bond donor (see Supporting Information Figures S5 and S6, and Table S16).

### Twisting Drug Molecule Shape from Flatland by LSS Logic

The hydrogenation of arenes has evolved continuously since Sabatier and Senderens’ seminal report in 1901.^20^ We^21−23^ and others^24−29^ have explored increasingly robust and selective protocols for arene saturation. Recently, developments in mild hydrogenation from our lab,^23^ Dou,^28^ and Du^29^ have enabled reduction of simple benzene or heteroaromatic substrates using boron-derived reductants in alcoholic solvents without the need for additional hydrogen gas. These operationally simple conditions obviate the need for gas handling in a high-pressure autoclave, facilitating adoption in diverse industrial, academic, and discovery settings. While these preliminary studies on simple substrates demonstrate that arenes containing select functional groups can be successfully saturated, no study has demonstrated the mild saturation of drugs or other complex molecules, which is a critical aspect of an LSS strategy. To explore if mild arene hydrogenation conditions could achieve LSS on drugs, we performed a high-throughput experimentation (HTE) reaction array surveying rhodium catalysts, boron-derived reductants at various stoichiometries, and alcohol solvents using the drugs propranolol, gemfibrozil, and ketoprofen as substrates (see Supporting Information Section 3).

Among the 96 reactions surveyed, [Rh(COD)OH]_2_, B_2_(OH)_4_ in ethanol and [Rh(COD)Cl]_2_, NH_3_·BH_3_ in 1,1,1-trifluoroethanol exhibited the best performance, reducing propranolol in 85% and 67% assay yield, respectively. In general, increasing the amount of reductant led to increased levels of LSS. Importantly, the solvents *n*-butanol and ethylene glycol, which were included as high-boiling solvents for the application of reaction miniaturization at nanomole scale,^30−32^ exhibited good performance using [Rh(COD)OH]_2_, B_2_(OH)_4_, with nearly identical results obtained using ethanol or ethylene glycol for the three drugs tested. These latter conditions in ethylene glycol suggested potential translation of LSS to drug compounds acquired from pharmaceutical compound collections in 96-, 384-, or 1536-well plates.

We next tested the impact of DMSO and water poisoning on the reaction. Pharmaceutical compound collections are available in high chemical diversity and are typically formatted in microtiter plates for rapid delivery to biochemical or cellular high-throughput screening campaigns. However, drugs in physical libraries are generally available in <10 mg amounts and are commonly stored in DMSO, which is most often wet owing to nonanhydrous storage and handling conditions. While wet DMSO is a suitable stock solution for many miniaturized bioassays, both DMSO and water are extreme inhibitors of most transition-metal-catalyzed reactions, often poisoning reactions when present in trace amounts. We studied the impact of DMSO and water on the present Rh-catalyzed hydrogenation and found that stoichiometric but not excess amounts of these additives were tolerated (see Supporting Information Figure S2b). Collectively, the finding that the protocol tolerated trace DMSO or water suggested that direct access to pharmaceutical compound collections *via* LSS could be possible to enable deep chemical space exploration and medicinal property optimization on a traditional (∼50 mg) scale in ethanol or miniaturized (∼50 μg) scale in ethylene glycol.

**Figure 3:**
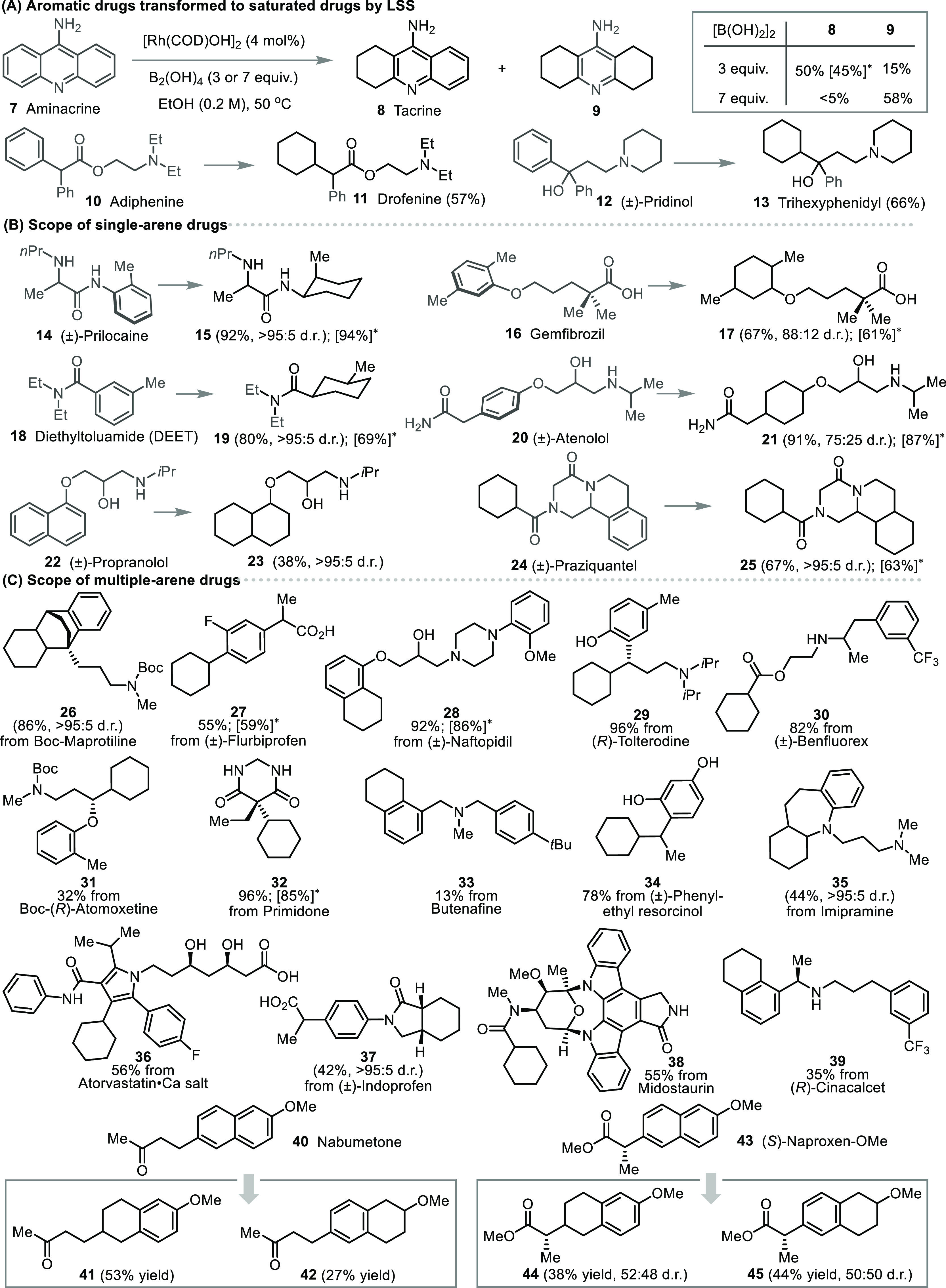
Late-stage saturation scope of benzene-containing drugs under rhodium catalysis conditions. (A) Aromatic drugs transformed to saturated drugs by LSS: production of saturated drugs with distinct medicinal properties. (B) Scope of drugs containing a single arene. (C) Scope of drugs containing multiple arenes. Note: the catalyst [Rh(COD)OH]_2_ is bench-stable for at least half a year and the reaction was conducted without the need of an autoclave or glovebox (for experimental details, see the Supporting Information). * Isolated yields shown in square brackets were obtained using ethylene glycol as solvent.

Starting on the traditional scale, we explored LSS for a variety of drugs (Figure 3) using ethanol or ethylene glycol as a solvent. Accordingly, **7** was reduced to give saturation product **8** in 50% yield along with doubly saturated product **9** in 15% yield. By increasing the amount of B_2_(OH)_4_ from 3 to 7 equiv, **9** became predominant (Figure 3a). This change in chemoselectivity is attributed to an increase in available H_2_.^23,28^ This observation highlights the ability to access diverse saturated analogues in chemical space through simple modification of reaction conditions. Next, adiphenine (**10**) and pridinol (**12**) were successfully reduced to furnish the saturated drugs drofenine (**11**) and trihexyphenidyl (**13**), respectively. A variety of other arene-containing pharmaceuticals were readily reduced to provide the saturated drug analogues in good to excellent yields (Figure 3b–c). Several examples highlight the remarkable functional group tolerance, for instance, primidone gave **32** in 96% yield despite the presence of two unprotected N–H bonds while (*R*)-tolterodine gave **29** even though a free phenol and basic amine were present. Remarkably, atorvastatin could be submitted to the reaction as its calcium salt, producing **36** in 56% yield. Free carboxylic acids (**17**, **27**, **37**), fluorine (**27**) and trifluoromethyl (**30**) were well tolerated. Densely functionalized alkaloids such as midostaurin were viable substrates, giving **38** in 55% yield. The calcium-modulating drug (*R*)-cinacalcet was readily reduced to give **39**, a compound with generally superior calculated physicochemical and pharmacokinetic properties compared to the starting material (Figure 6; for details, see Supporting Information Section 6). Interestingly, reduction of nabumetone (**40**) and (*S*)-naproxen-OMe (**43**) gave regioisomers **41**–**42** and **44**–**45**, respectively. While this example highlights the opportunity to produce a diversity of saturated analogues in a single reaction, we observed very high regio- and diastereoselectivity for most substrates.

**Figure 4:**
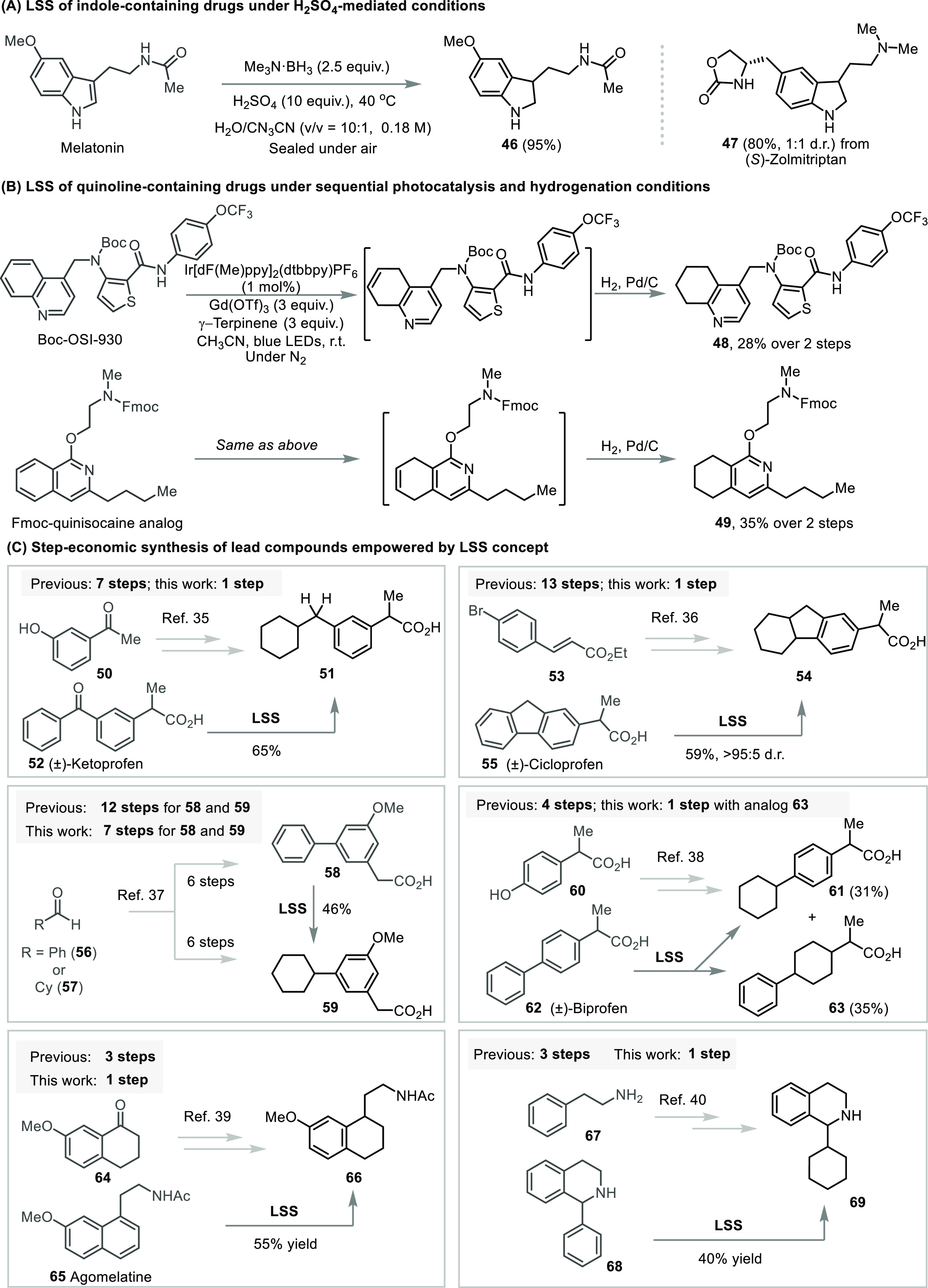
(A) LSS of indole-containing drugs under H_2_SO_4_-mediated reduction conditions. (B) LSS of quinoline-containing drugs under sequential photocatalysis and hydrogenation conditions. (C) Step-economic synthesis of lead compounds empowered by the LSS concept (for experimental details, see the Supporting Information).

LSS is a logic for expanding the drug space by converting aromatic drugs to their saturated congeners that conceptually embraces any arene reduction protocol that is able to successfully reduce complex substrates. Therefore, any mild and selective arene reduction protocols can be considered a part of the LSS toolbox. While we recommend the rhodium-catalyzed conditions described above as a first pass, with some substrates, particularly indoles and quinolines, other protocols gave superior results (Figure 4a–b). Thus, a sulfuric acid-mediated reduction method was used to produce indoline products **46** and **47**.^33^ Likewise, a photocatalysis-hydrogenation sequential protocol was applied to saturate Boc-OSI-930 and a Fmoc-quinisocaine analogue in a highly selective fashion, thus smoothly leading to the reduced products **48** and **49**, respectively.^34^ It is noteworthy that the photocatalyzed conditions selectively reduced the benzene ring rather than the pyridine in these (iso)quinolines.

Furthermore, LSS can dramatically improve the synthetic efficiency and complement retrosynthetic strategies (Figure 4c). For example, compound **51**, an inhibitor for chemotactic activation of neutrophils, has been conventionally obtained by a 7-step linear synthesis from **50**.^35^ In contrast, the commercial drug ketoprofen **52** can be directly saturated to afford **51** in a single step. Similarly, the anti-inflammatory agent **54** was previously synthesized in 13 steps from **53**,^36^ while LSS logic provided **54** directly from commercial cicloprofen (**55**) in 59% yield. The status quo to access saturated analogues by total synthesis is exemplified in the production of **58** (aromatic) and **59** (aliphatic)^37^ in 6 steps each from **56** and **57**, whereas LSS converts **58** to **59** in 1-step and 46% yield with no protection of the carboxylic acid needed. While we have generally observed excellent regioselectivity in our studies, product mixtures are occasionally obtained. The synthesis of separable analogues **61** and **63** from commercially available biprofen (**62**) showcases how LSS can more expediently explore three-dimensional chemical space, compared to the production of **61** alone from **60**.^38^ Additional applications of LSS to shortcut *de novo* syntheses are shown for leads **66** and **69**.^39,40^

**Figure 5:**
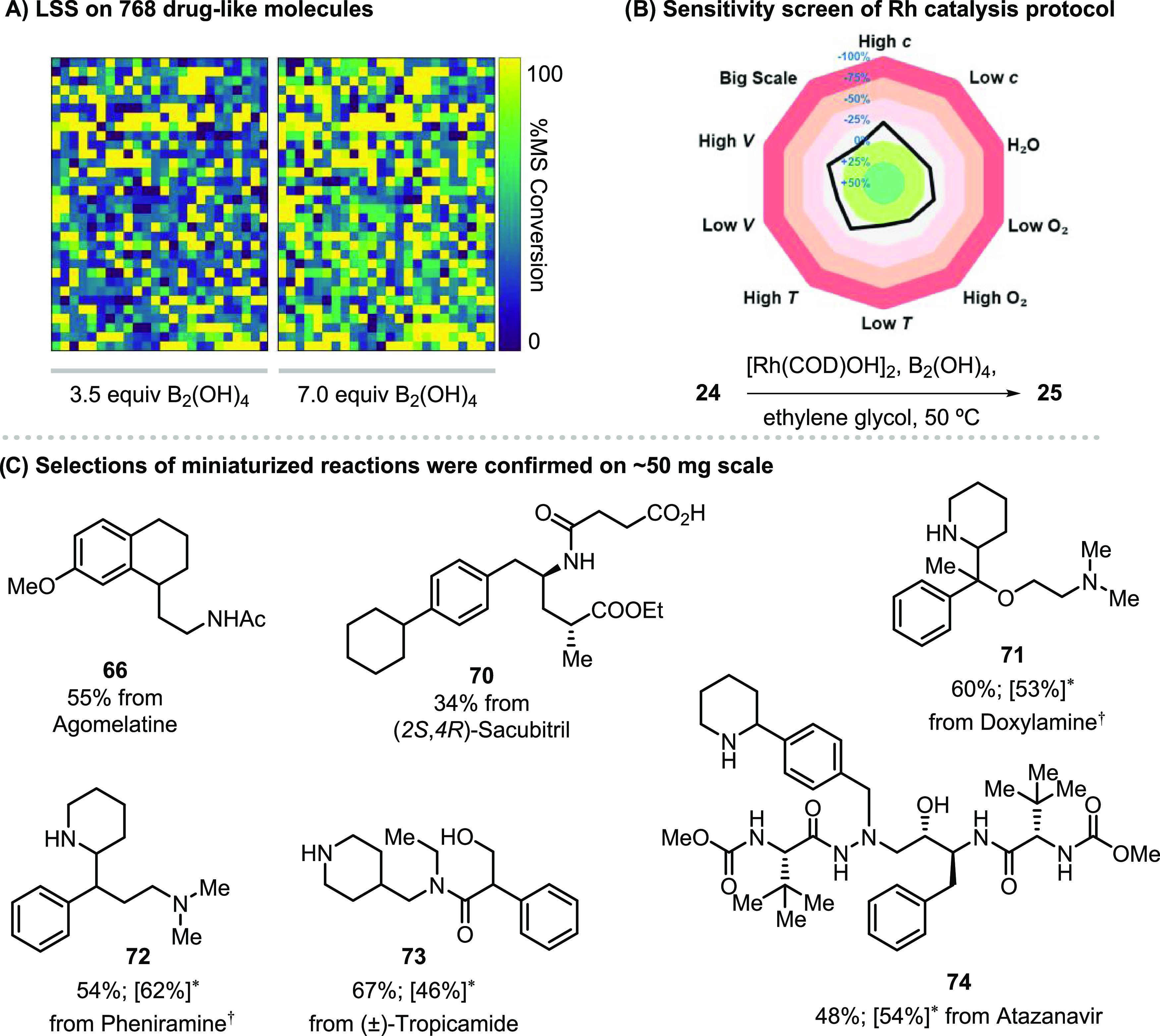
Late-stage saturation (LSS) of drug libraries *via* ultrahigh-throughput experimentation. (A) A library of 768 pharmaceutically relevant complex molecules stored in wet DMSO were subjected to LSS using [Rh(COD)OH]_2_, B_2_(OH)_4_ in ethylene glycol. (B) A sensitivity screen shows the protocol is tolerant of various changes to reaction conditions. (C) Selections of miniaturized reactions were confirmed on an ∼50 mg scale in ethanol to obtain isolated yields (for experimental details, see the Supporting Information). * Isolated yields shown in square brackets were obtained using ethylene glycol as solvent. ^†^One isomer was isolated from the diastereoisomers mixture.

Miniaturization of reactions *via* ultraHTE^30,31^ can be impactful for advancing medicinal chemistry, particularly *via* modern direct-to-biology approaches.^31^ We acquired a library of drugs from The University of Michigan Drug Discovery Center that had been stored in DMSO for many months, with no special precautions taken to exclude ambient moisture. Since the LSS protocol tolerates traces of, but not an excess of, DMSO and water (see Supporting Information Figure S2b), we reformatted a library of 768 drugs from DMSO solutions into a 384-well microtiter source plate and removed volatiles by centrifugal evaporation. The residues were reconstituted in ethylene glycol, then transferred to a 1536-well reaction plate along with [Rh(COD)OH]_2_ (4 mol %) and B_2_(OH)_4_ (3.5 or 7.0 equiv). The plate containing 1536 reaction mixtures was then sealed and heated at 50 °C for 24 h (see the Supporting Information for plate sealing studies). Analysis of the conversion to product by ultraperformance liquid chromatography–mass spectrometry (UPLC-MS) showed product formation for 690 of the 768 drugs. The conversion to product was generally good to excellent (Figure 5a), with an average saturation conversion of 42.9% over the 1536 reactions. The stoichiometry of B_2_(OH)_4_ (3.5 versus 7.0 equiv) had a modest impact on reaction conversion. The performance of the LSS protocol on such a broad diversity of pharmaceutically relevant molecules is exceptional, especially when compared to other miniaturized high-throughput reaction campaigns on pharmaceuticals and natural products.^30−32,41^ A cheminformatic analysis (Supporting Information Figure S19) showed no obvious pockets of chemical space where substrates did not yield the saturated product. In addition to high reaction performance on a diversity of complex molecules that were reformatted from DMSO solutions, we studied the susceptibility of the Rh-catalyzed LSS protocol to varied conditions by subjecting the anthelminthic praziquantel (**24**), [Rh(COD)OH]_2_, B_2_(OH)_4_ in ethylene glycol to a sensitivity screen.^42^ We observed that the reaction temperature, volume, concentration, scale, and addition of water or oxygen had little impact on the reaction yield (Figure 5b). The fact that the Rh-catalyzed LSS protocol was agnostic of reaction scale was further verified by comparing reaction performance from reactions run on an ∼50 μg scale in ethylene glycol to reactions run on a ∼50 mg scale in ethanol or ethylene glycol for 34 drugs, and the correlation of UPLC-MS conversion from the miniaturized reactions to isolated yields from reactions performed on a traditional scale was excellent (see Supporting Information Figure S20). A sampling of the isolated products (**66**–**74**) is shown in Figure 5c. It is worth noting that the pyridine-containing drugs were also tolerated well to give the piperidine congeners **71**–**74**. To our knowledge, this represents the first successful application of a transition-metal-catalyzed reaction to a library of drugs reconstituted from wet DMSO.

General chemoselectivity trends emerged. For instance, we noted that the Rh-catalyzed hydrogenation protocol was the best method for reducing benzene- and pyridine-containing drugs, while the acid-mediated reduction and photocatalyzed-hydrogenation sequence were most suitable for indole- and quinoline-containing drugs, respectively. Under the Rh-catalyzed hydrogenation, the less-substituted (i.e., **27**–**31**, **70**) or less-electron-rich (i.e., **66**) benzene rings are more prone to be reduced. Further, pyridines, which generally have lower aromatic resonance energy than benzenes, are typically more easily reduced (i.e., **71**–**74**). We performed a simple linear regression on our HTE data and observed no consistent structural features that correlated with reactivity, although our data set was likely too sparse to enable such trends to emerge. A complementary systematic evaluation of arene saturation chemoselectivity was recently described by Doyle, Lyons, and co-workers.^43^ The diastereoselectivities observed in the Rh-catalyzed method were generally *cis* (i.e., **15**, **19**, **37**, and **54**) which is consistent with earlier reports on arene reduction.^25^ The relative stereochemistry of some analogues, such as **25**, could not be unequivocally assigned (see Supporting Information Figure S23).

**Figure 6:**
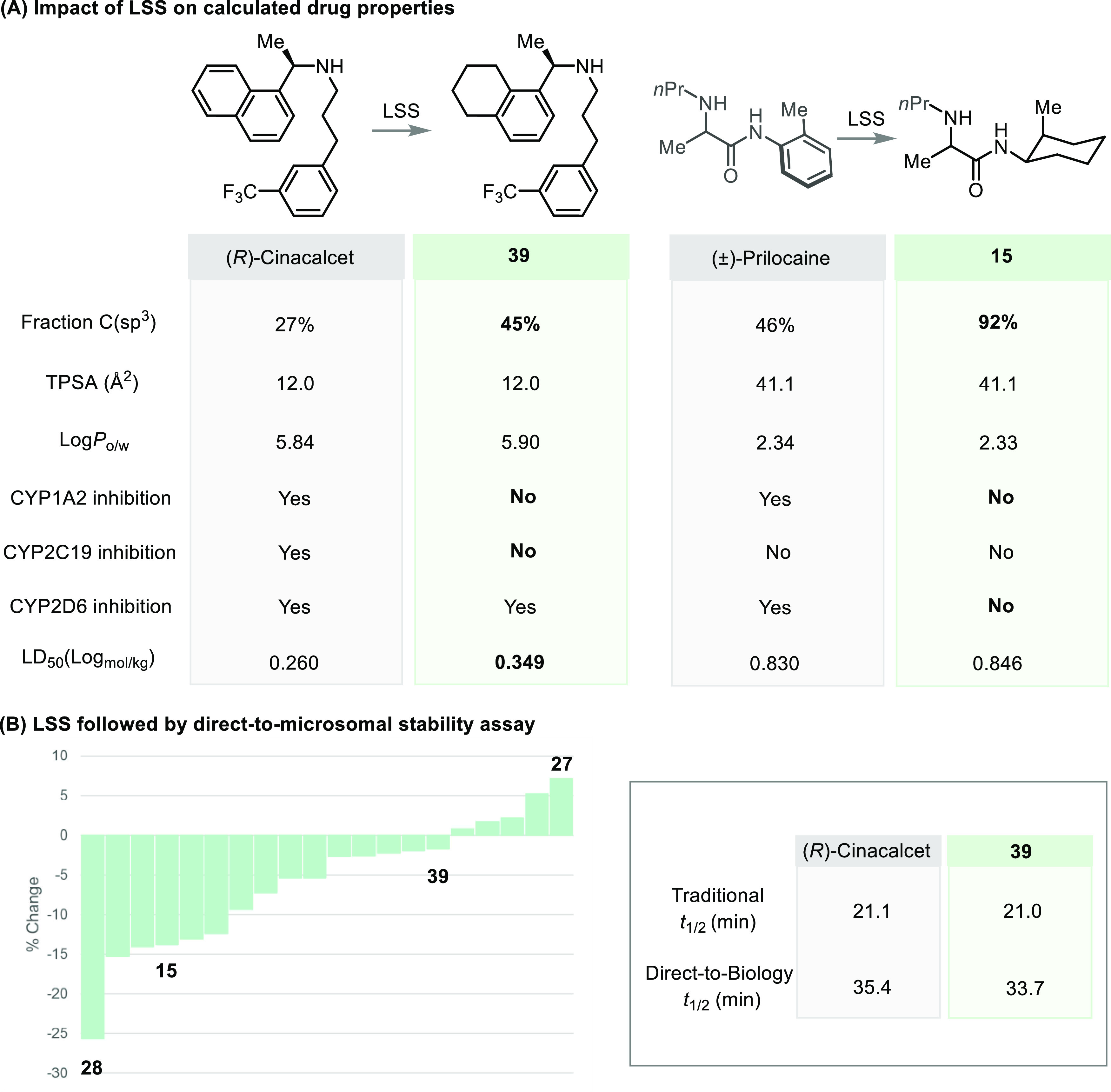
(A) Predicted properties for Prilocaine, Cinacalcet, and their LSS analogues **15** and **39**. (B) Microsomal stability data obtained for 24 LSS reaction mixtures show the change in microsomal stability following saturation of various drugs.

Increasing the three-dimensionality of pharmaceuticals is expected to modulate their overall properties, and calculations on cinacalcet and prilocaine alongside their saturated congeners **39** and **15** highlight some potential differences (Figure 6a). The measured octanol–water partition coefficient (Log *P*) of several drugs and their saturated congeners suggests that LSS is a viable strategy to modulate hydrophilicity, with most drugs showing a slight increase in Log *P* upon saturation (see Supporting Information Table S28). A final experiment was performed to merge LSS with microsomal stability testing in a direct-to-biology^31^ format. Here, 24 drugs were saturated and, following reaction quenching and metal scavenging, directly transferred an incubation with human liver microsomes (Figure 6b and Supporting Information Figure S22). While most drugs exhibited little change or a modest decrease in microsomal stability upon saturation, several drugs, most notably flurbiprofen congener **27** exhibited an increase in stability (see Supporting Information Table S25). Given the well-documented benefits of increasing a pharmaceutical’s Fsp^3^,^1,2,11,12^ this ability to saturate and *in situ* demonstrate improved metabolic stability could be beneficial to drug discovery efforts.

## Conclusions

Our results highlight the potential to convert aromatic drugs to their saturated congeners in a rapid and user-friendly format. A growing body of evidence suggests that three-dimensional molecules have medicinal properties superior to those of their flat aromatic analogues. Nonetheless, cross-coupling of aromatic building blocks, commercially available in high diversity, is a robust technology with considerable user adoption, making the production of flat drugs quite facile. The saturation of such aromatic molecules at a late stage as described here will allow for rapid exploration of three-dimensional chemical space toward improved clinical outcomes and other diverse applications. Further, we consider that other user-friendly and chemoselective methods for LSS will emerge, including those based on earth-abundant metal catalysts.

## Supporting Information Available

Cheminformatic analysis, experiment details, characterization data, property prediction data, and NMR spectra (PDF)
