# Supplementary material for: Late-Stage Saturation of Drug Molecules
Source: J Am Chem Soc. 2024 Apr 15;146(17):11866–75. doi: 10.1021/jacs.4c00807 (PMC11066876; doi:10.1021/jacs.4c00807)
Supplement: Supplementary file 1 — ja4c00807_si_001.pdf [file ja4c00807_si_001.pdf]

Supporting Information for

Late-Stage Saturation of Drug Molecules

De-Hai Liu,[1,‡] Philipp M. Pflüger,[2,‡] Andrew Outlaw,[3,‡] Lukas Lückemeier,[2] Fuhao Zhang,[2] Clinton Regan,[3] Hamid Rashidi Nodeh,[3] Tim Cernak,[3,*] Jiajia Ma,[1,2*] Frank Glorius[2,*]

[1]Frontiers Science Center for Transformative Molecules, Shanghai Key Laboratory for Molecular Engineering of Chiral Drugs, School of Chemistry and Chemical Engineering and Zhangjiang Institute for Advanced Study, Shanghai Jiao Tong University, Shanghai 200240, P. R. China

[2]Organisch-Chemisches Institut, Westfälische Wilhelms-Universität Münster, Corrensstraße 40, 48149 Münster, Germany

[3]Department of Medicinal Chemistry, University of Michigan, Ann Arbor, Michigan, 48109, USA

[‡]These authors contributed equally: De-Hai Liu, Philipp M. Pflüger, Andrew Outlaw.

*Corresponding author: tcernak@med.umich.edu; majj@sjtu.edu.cn; glorius@uni-muenster.de

Content

1. General Information S3

2. Experimental and Characterization Data of LSS Products S5

3. Procedure for Arene Reduction Comparison Screen S76

4. Step-Economic Synthesis of Lead Compounds S80

5. Data Guided Validation of the LSS Concept S94

5.1 Theoretical Background S94
5.2 Data Acquisition S95
5.2.1 PubChem S95
5.2.2 ChEMBL S95
5.3 Validation of the LSS Concept (Program) S95
5.3.1 Finding of Drug/LSS Pairs S95
5.3.2 Data Analyzation S102
5.3.3 Library Expansion by LSS S110
5.4 Validation of the LSS Concept (Results) S119
5.4.1 LSS Pairs S119
5.4.2 Aromatic Patterns in Drugs and Pairs S121
5.4.3 Substitution Patterns on Arenes in Drugs and Pairs S125
5.4.4 Library Expansion by LSS S127
5.5 ADMET Analysis S129
5.5.1 Background S129
5.5.2 Property and Activity prediction S130
5.5.3 Statistical Data Analysis (Program) S145
5.5.4 Statistical Data Analysis (Results) S150

6. Measurement of Metabolic stability in Human Liver Microsomes S156

7. Reaction-Condition-Based Sensitivity Assessment S161

8. Ultra-High Throughput Synthesis of a Diversified Drug Library using LSS S163

8.1. Determination of DMSO Tolerance S163

8.2 Quantitation Method using UPLC-MS S165

8.3 Microtiter Plate Sealing Studies S166

8.4 Procedure for 1,536-Well Plate Experiment S168

8.5 SMILES Strings S174

8.6 Procedure for High-Throughput Human Liver Microsomal Stability Assay S240

9. Relative Configuration Consideration S246

10. Measurement of LogPo/w S247

11. References S251

1. General Information

General remarks

Unless otherwise noted, all reactions were carried out under an atmosphere of argon or nitrogen in dried glassware. Reaction temperatures are referred to the ones of the heating/cooling media (heating block, cryogenic bath), unless otherwise stated. Dry solvents were either purchased from ACROS Organics or Adamas (H_2O < 50 ppm), stored under activated molecular sieves, withdrawn under positive argon pressure or purified using a custom-made SPS purification system (activated alumina columns) and transferred under argon.

Solvents for flash column chromatography (petroleum ether, EtOAc, CH_2Cl_2) were purchased of technical grade and purified by atmospheric pressure distillation or purchased of reagent grade (MeOH) and used without additional purification. Reagents were purchased from Sigma-Aldrich, ACROS Organics, TCI, Adamas, Bidepharm and used without additional purification, unless otherwise stated.

Reaction setup involving dosing of substrates and reagents into the 1,536-well plate experiment were set up in an MBraun LABmaster Pro Glove Box (H_2O level <0.1 ppm, O_2 level <0.1 ppm). Transferring and dosing of 384 and 1,536-well plates was conducted using an SPT Labtech Mosquito liquid handling robot and the corresponding mosquito software to develop dosing and mixing procedures. Glass 1-dram (Fisherbrand™ parts No. 03-339-21B) or 2-dram vials (Fisherbrand™ parts No. 03-339-21D) were used as reaction vessels, fitted with screwcaps with Teflon-coated silicone septa (CG-4910-02), and magnetic stir bars (Fisher Scientific #14-513-93 or #14-513-65).

Microtiter plate evaporation was performed using a Genevac HT-4X Series II centrifugal evaporator with vacuum pump controller (Genevac #70-0938), BOC-Edwards XDS-5 dry scroll pump (Edwards #A724-01-104), and side-bridge swing attachments for well plate evaporation. Vacuum plot capacity: 2.3 L. Vacuum control range: 1-1000 mbar. Standard evaporation method listed below: 40°C internal chamber temperature, 1 mbar chamber vacuum, very low rotor speed, 2 hours runtime.

Purification techniques

Flash chromatography was performed on silica gel (200-300 mesh) under a slight positive pressure. Thin layer chromatography (TLC) was carried out on YANTAI XINNUO silica gel $60F_{254}$ pre-coated glass sheets and were visualized using UV light (254 nm) and stained with basic aqueous potassium permanganate (2 g $KMnO_4$, 10 g K_2CO_3, 0.3 g of NaOH in 200 ml of deionized water) or alcoholic

solution of phosphomolybdic acid (10 g of PMA in 100 ml of absolute EtOH) dips.

Analytical techniques

NMR-spectra were recorded on a Bruker Avance II 400 MHz or 500 MHz spectrometers. Chemicals shifts (δ) are quoted in ppm downfield of tetramethyl silane (0.00 ppm). The residual solvent signals were used as references for ^1H and ^{13}C NMR spectra ($CDCl_3$: δ_H = 7.26 ppm, δ_C = 77.16 ppm; DMSO-d_6: δ_H = 2.50 ppm, δ_C = 39.52 ppm; CD_3OD: δ_H = 4.87 ppm, δ_C = 49.00 ppm; acetone-d_6: δ_H = 2.05 ppm, δ_C = 206.26 ppm). ^{19}F NMR spectra were calibrated using absolute referencing to the ^1H NMR spectrum, as suggested by IUPAC.[1] Coupling constants (*J*) are quoted in Hz and rounded to the nearest 0.1 Hz. The multiplicity abbreviations used (or combinations thereof) are: s = singlet, d = doublet, t = triplet, q = quartet, hept = heptet, m = multiplet.

High Resolution Mass Spectrometry (HRMS) analysis was obtained using Electrospray Ionization (ESI) and reported as m/z (relative intensity). ESI was acquired using a Waters/Micromass LCT Classic (ESI-TOF). Reversed-phase HPLC chromatography was performed on Agilent 1260.

DMSO quantitation experiments were performed using an Agilent 8890 Gas Chromatograph System and 5977B Series Mass Selectivity Detector. 1.0 µL liquid sample injections were taken from Methanol stock solutions and microtiter well plate samples using splitless injection mode with a PAL RSI 120 Series 2 autosampler (CTC Analytics #G7368-66668). Column used: Agilent HP-5MS capillary column (Agilent #AG19091S-433), 0.25 mm x 30 m dimensions with 0.250 mm film. Inlet temperature: 260°C. Helium Carrier gas flow rate: 0.9 mL/min. The Mass Selectivity Detector was set to scan mode. Ion Source: 72 eV. Quadrupole temperature: 230°C. Typical oven program gradient: oven start temperature of 70°C, ramp 14°C/min until oven temperature of 150°C, hold 150°C oven temperature for 2 minutes.

Reaction analysis for high throughput screening experiments was performed using a Waters I-class ACQUITY UPLC-MS equipped with in-line photodiode array detector (PDA) and QDa mass detector (ESI positive ionization mode). 1.0 µL sample injections were taken from DMSO solutions of reaction mixtures (~1 mM). A partial loop injection mode was used with the needle placement at 1.0 mm from bottom of the wells and a 0.2 µL air gap at pre-aspiration and post-aspiration. Column used: Waters Cortecs UPLC C18+ column, 2.1mm x 50 mm with (Waters #186007114) with Waters Cortecs UPLC C18+ VanGuard Pre-column 2.1mm x 5 mm (Waters #186007125), Mobile Phase A: 0.1 % formic acid in Optima LC/MS-grade water, Mobile Phase B: 0.1% formic acid in Optima LC/MS-grade

MeCN. Flow rate: 1 mL/min. Column temperature: 45°C. The PDA sampling rate was 20 points/sec. The QDa detector monitored m/z 150-750 with a scan time of 0.06 seconds and a cone voltage of 30 V. The PDA detector range was between 210 nm – 400 nm with a resolution of 1.2 nm. A two-minute method was used and the method gradients are below: 0 min: 0.8 mL/min, 95% 0.1% formic acid in water/5% 0.1% formic acid in acetonitrile; 1.5 min : 0.8 mL/min, 0.1% 0.1% formic acid in water/99.9% 0.1% formic acid in acetonitrile; 1.91 min : 0.8 mL/min, 95% 0.1% formic acid in water/5% 0.1% formic acid in acetonitrile.

2. **Experimental and Characterization Data of LSS Products**

General Procedure

A dried 5 or 10 mL Schlenk tube was charged with the starting material drug or lead compound, $B_2(OH)_4$ (3-7 equivalents) and $[Rh(COD)OH]_2$ (2.5-5 mol%) in ethanol (0.1 M) under nitrogen or argon atmosphere.[2,3] The reaction mixture was stirred vigorously at 50-80 °C until the full conversion of the starting material. Afterwards, the reaction was quenched by saturated aqueous $NaHCO_3$ and extracted with CH_2Cl_2 or EtOAc (10 mL × 3). The organic phases were combined and concentrated under reduced pressure. The product was obtained by flash chromatography on silica gel (petroleum ether/EtOAc, petroleum ether/Et_2O or CH_2Cl_2/MeOH, optionally, with NH_3 (7.0 M in MeOH) as additive). Note, in case the substrate drug was a carboxylic acid, the reaction mixture was concentrated and subjected to the column chromatography directly.

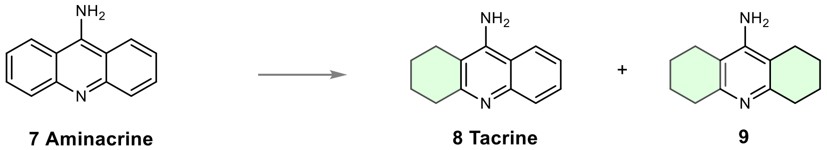

7 Aminacrine **8 Tacrine** **9**

According to the general procedure, a mixture of **aminacrine** (38.8 mg, 0.20 mmol, 1.0 equiv.), $B_2(OH)_4$ (54.0 mg, 0.60 mmol, 3.0 equiv.) and $[Rh(COD)OH]_2$ (3.7 mg, 4.0 mol%, 0.008 mmol) in EtOH (1.0 mL, 0.2 M) was stirred under nitrogen atmosphere for 26 hours at 50 °C to afford **tacrine** as a yellow solid (19.9 mg, 50% yield) and the second product **9** (6.0 mg, 15% yield).

Under an alternative condition with $B_2(OH)_4$ (113.3 mg, 1.4 mmol, 7.0 equiv.) and $[Rh(COD)OH]_2$ (3.7 mg, 4.0 mol%, 0.008 mmol), **7** (23.3 mg, 58% yield) was afforded whereas tacrine was not obtained.

Purification conditions: NH_3(7.0 M solution in MeOH)/CH_2Cl_2 = 1:50 to 1:20

R_f(tacrine) = 0.7 in NH_3(7.0 M solution in MeOH)/CH_2Cl_2 = 1:10.

R_f(**9**) = 0.5 in NH_3(7.0 M solution in MeOH)/CH_2Cl_2 = 1:10.

Analytical data of **tacrine**:

^{1}H NMR (400 MHz, $CDCl_3$) δ 7.89 (d, J = 8.8 Hz, 1H), 7.73 (d, J = 8.4 Hz, 1H), 7.59 - 7.53 (m, 1H), 7.39 - 7.33 (m, 1H), 4.84 (br, 2H), 3.02 (t, J = 6.0 Hz, 2H), 2.59 (t, J = 6.0 Hz, 2H), 1.98 - 1.87 (m, 4H)

HRMS (ESI, m/z) calcd for $C_{13}H_{15}N_2^+$ $[M+H]^+$: 199.1235, found: 199.1237.

All other analtical data is consistent with the previous report.[4]

Analytical data of **9**:

^{1}H NMR (400 MHz, $CDCl_3$) δ 4.70 (br, 2H), 2.86 - 2.77 (m, 4H), 2.44 - 2.36 (m, 4H), 1.86 - 1.73 (m, 8H).

^{13}C NMR (100 MHz, $CDCl_3$) δ 151.5, 151.1, 113.2, 30.8, 22.9, 22.4.

HRMS (ESI, m/z) calcd for $C_{13}H_{19}N_2^+$ $[M+H]^+$: 203.1548, found: 203.1549

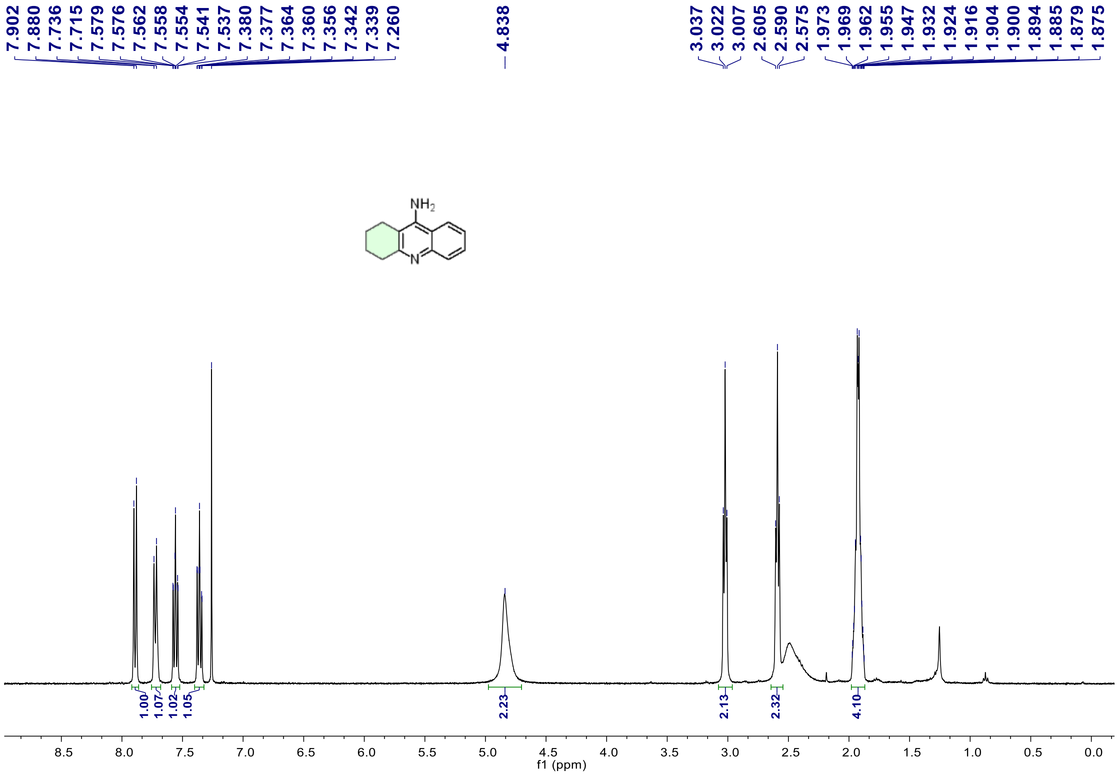

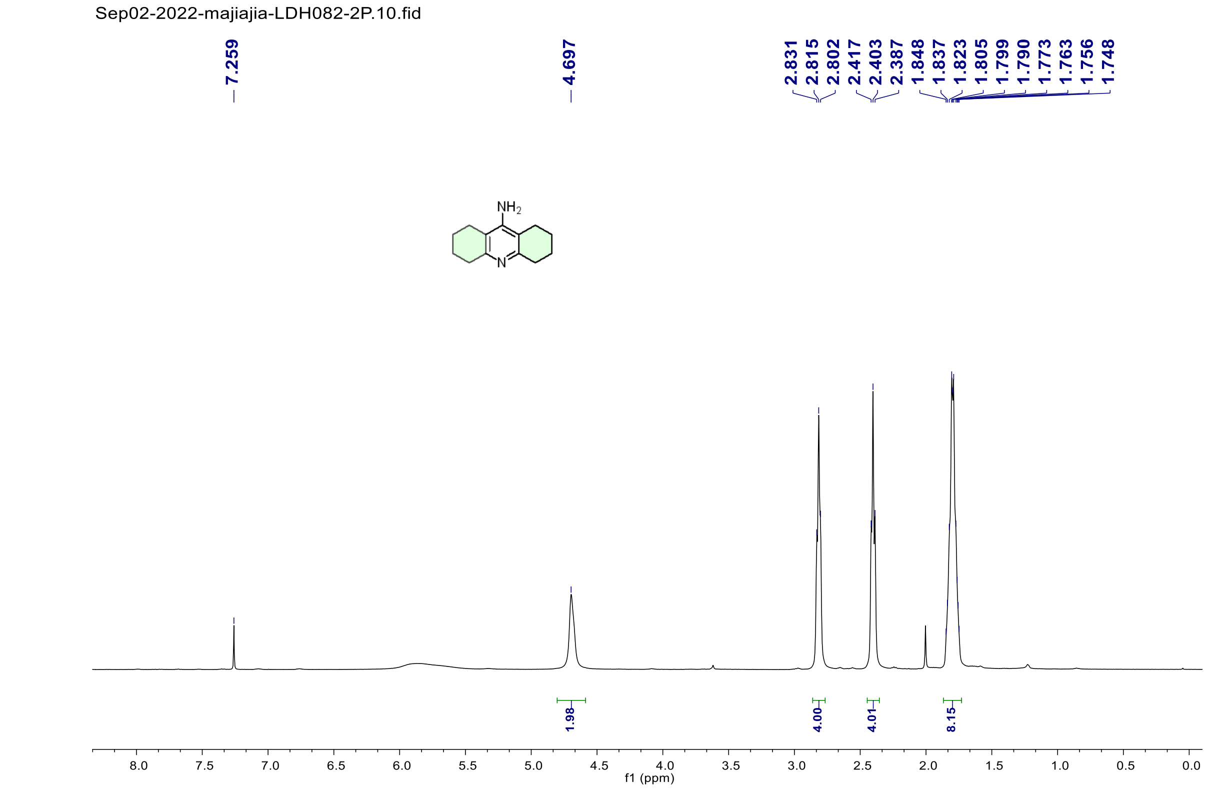
Sep02-2022-majiajia-LDH082-2P.10.fid
7.259
4.697
2.831
2.815
2.802
2.417
2.403
2.387
1.848
1.837
1.823
1.805
1.799
1.790
1.773
1.763
1.756
1.748
NH2
N
1.98
4.00
4.01
8.15
f1 (ppm)

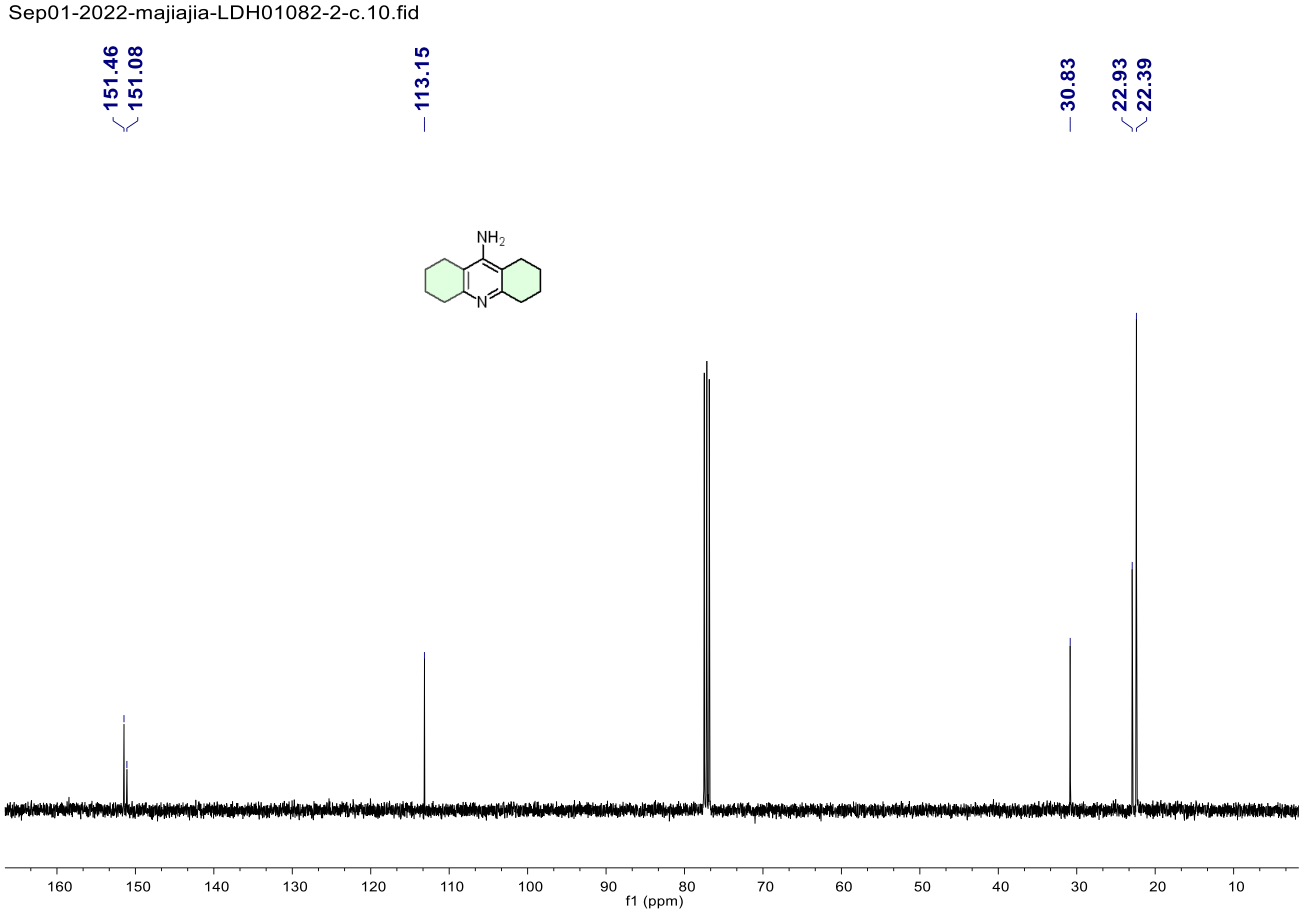
Sep01-2022-majiajia-LDH01082-2-c.10.fid
151.46
151.08
113.15
30.83
22.93
22.39
NH2
N
f1 (ppm)

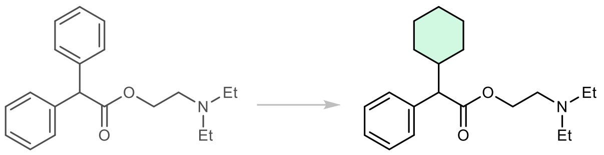

10 Adipheine **11 Drofenine** (57% yield)

According to the general procedure, a mixture of **adiphenine** (62.3 mg, 0.2 mmol, 1.0 equiv.), $B_2(OH)_4$ (107.5 mg, 1.2 mmol, 6.0 equiv.) and $[Rh(COD)OH]_2$ (3.7 mg, 4.0 mol%, 0.008 mmol) in in *i*-PrOH/EtOH (v/v = 9:1, 1.0 mL) was stirred under nitrogen atmosphere for 24 hours at 80 °C to afford **drofenine** as a colorless oil (36.4 mg, 57% yield).

^{1}H NMR (400 MHz, $CDCl_3$) δ 7.35 - 7.24 (m, 4H), 7.27 - 7.18 (m, 1H), 4.20 - 4.04 (m, 2H), 3.22 (d, *J* = 10.8 Hz, 1H), 2.64 (t, *J* = 6.0 Hz, 2H), 2.50 (q, *J* = 7.2 Hz, 4H), 2.01 (qt, *J* = 11.2, 3.2 Hz, 1H), 1.87 - 1.79 (m, 1H), 1.78 - 1.68 (m, 1H), 1.67 - 1.53 (m, 2H), 1.36 - 1.24 (m, 2H), 1.19 - 1.02 (m, 3H), 0.97 (t, *J* = 7.2 Hz, 6H), 0.73 (qd, *J* = 12.4, 3.6 Hz, 1H).

^{13}C NMR (100 MHz, $CDCl_3$) δ 174.0, 138.0, 128.7, 128.5, 127.2, 62.9, 59.0, 51.1, 47.6, 41.1, 32.1, 30.5, 26.4, 26.12, 26.09, 12.0.

HRMS (ESI, *m/z*) calcd for $C_{20}H_{32}NO_2^+$ $[M+H]^+$:318.2428, found: 318.2433

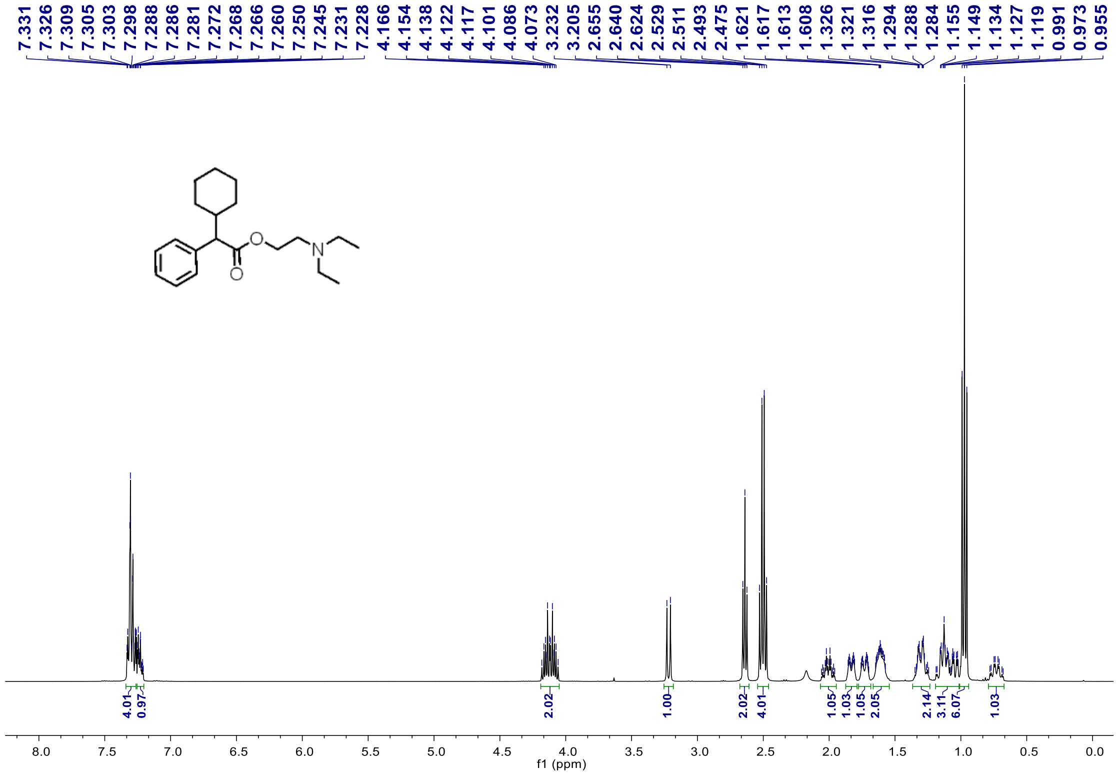

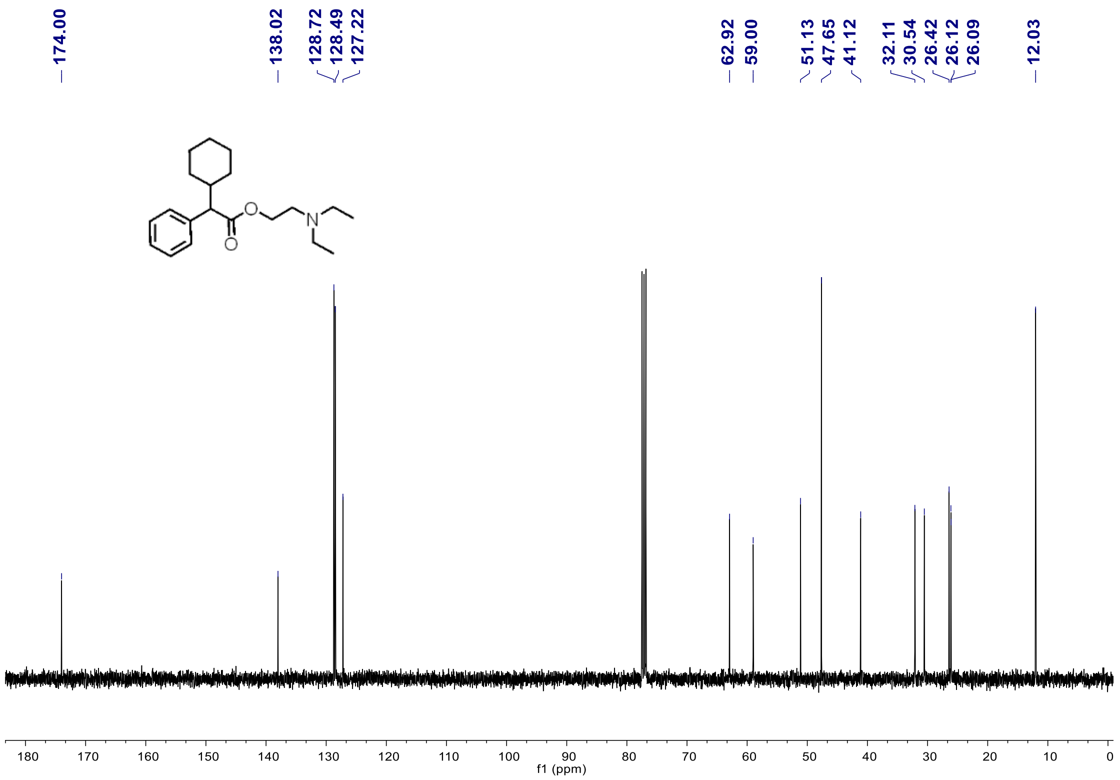

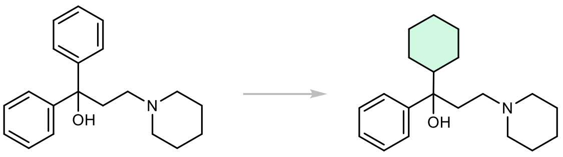

12 (±)-Pridinol **13 Trihexyphenidyl** (66% yield)

According to the general procedure, a mixture of **(±)-pridinol** (59.1 mg, 0.20 mmol, 1.0 equiv.), $B_2(OH)_4$ (80.6 mg, 0.9 mmol, 4.5 equiv.) and $[Rh(COD)OH]_2$ (3.7 mg, 4.0 mol%, 0.008 mmol) in EtOH (1.0 mL, 0.2 M) was stirred under nitrogen atmosphere for 36 hours at 80 °C to afford **trihexyphenidyl** as a white solid (39.8 mg, 66% yield).

Purification conditions: Acetone/petroleum ether = 1:8 to 1:4.

R_f = 0.4 in Acetone/petroleum ether = 1:3.

^{1}H NMR (400 MHz, $CDCl_3$) δ 7.39 (d, J = 7.6 Hz, 2H), 7.30 (t, J = 7.6 Hz, 2H), 7.18 (t, J = 7.2 Hz, 1H), 2.64 - 2.37 (br, 1H), 2.35 - 2.18 (m, 3H), 2.15 - 1.92 (m, 3H), 1.80 - 1.68 (m, 2H), 1.65 - 1.49 (m, 7H), 1.46 - 1.34 (m, 2H), 1.32 - 1.12 (m, 3H), 1.12 - 0.97 (m, 4H).

^{13}C NMR (100 MHz, $CDCl_3$) δ 147.0, 127.7, 126.3, 125.8, 79.8, 55.5, 54.4, 49.1, 33.1, 27.2, 27.0, 26.9, 26.8, 26.6, 25.9, 24.1.

HRMS (ESI, m/z) calcd for $C_{20}H_{32}NO^+$ $[M+H]^+$: 302.2478, found: 302.2482.

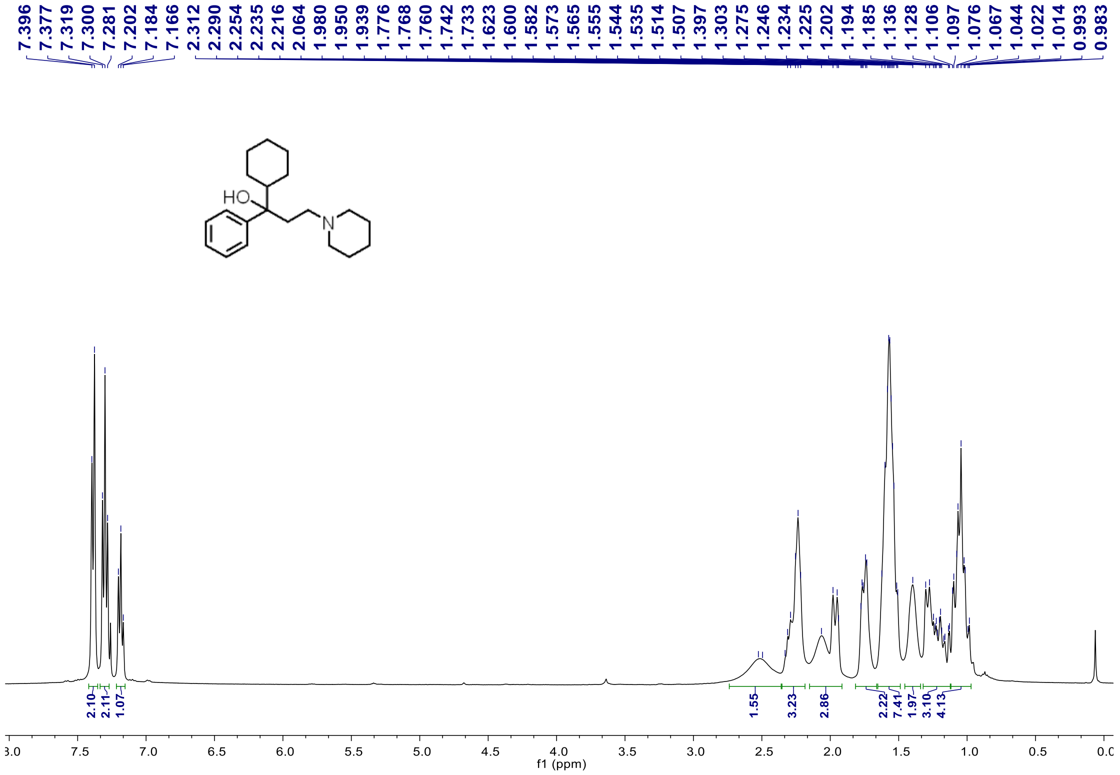
7.396
7.377
7.319
7.300
7.281
7.202
7.184
7.166
2.312
2.290
2.254
2.235
2.216
2.064
1.980
1.950
1.939
1.776
1.768
1.760
1.742
1.733
1.623
1.600
1.582
1.573
1.565
1.555
1.544
1.535
1.514
1.507
1.397
1.303
1.275
1.246
1.234
1.225
1.202
1.194
1.185
1.136
1.128
1.106
1.097
1.076
1.067
1.044
1.022
1.014
0.993
0.983
HO
N
2.10
2.11
1.07
1.55
3.23
2.86
2.22
7.41
1.97
3.10
4.13
f1 (ppm)

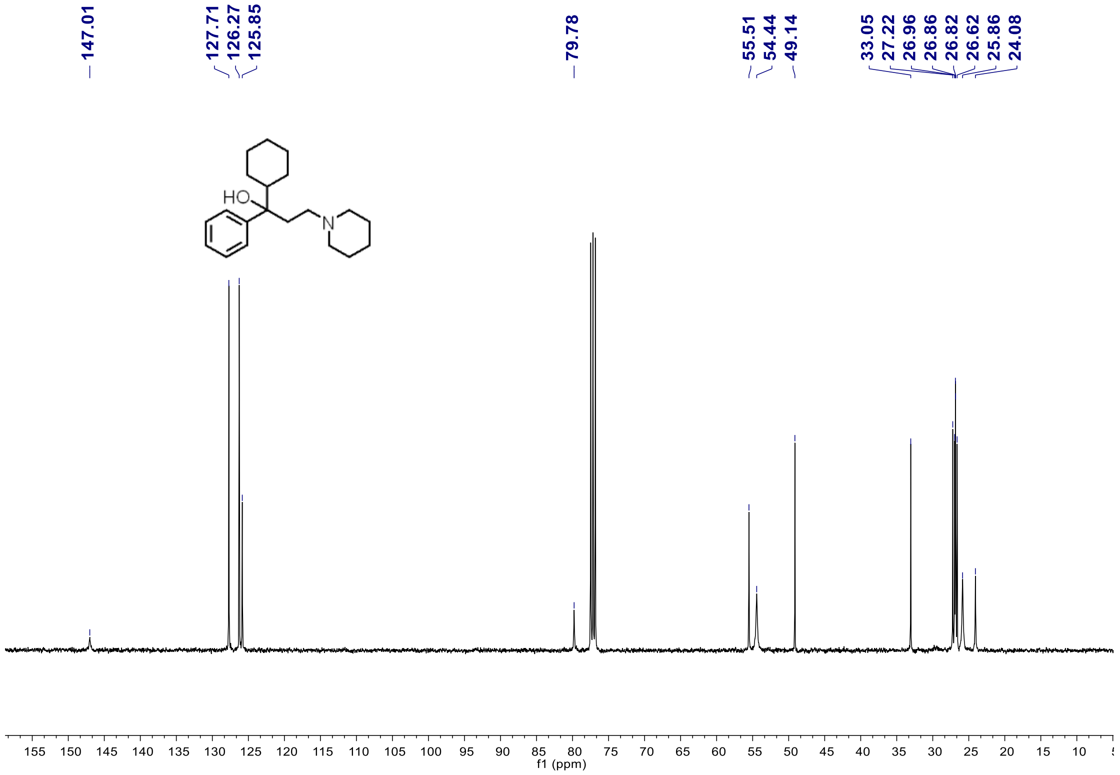
147.01
127.71
126.27
125.85
79.78
55.51
54.44
49.14
33.05
27.22
26.96
26.86
26.82
26.62
25.86
24.08
HO
N
f1 (ppm)

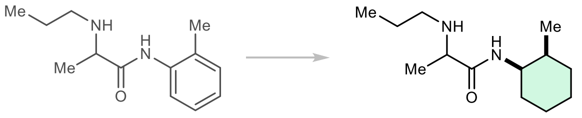

14 (±)-Prilocaine **15** (92% yield, >95:5 d.r.)

According to the general procedure, a mixture of **(±)-prilocaine** (44.0 mg, 0.2 mmol, 1.0 equiv.), $B_2(OH)_4$ (107.5 mg, 1.2 mmol, 6.0 equiv.) and $[Rh(COD)OH]_2$ (3.6 mg, 4 mol%, 0.0080 mmol) in EtOH (1.0 mL, 0.2 M) was stirred under argon atmosphere for 14 hours at 50 °C to afford **15** as a colorless oil (41.0 mg, 92% yield, >95:5 d.r.).

^{1}H NMR (400 MHz, $CDCl_3$, mixture of diastereomers) δ 7.53 (br, 1H), 4.03-3.96 (m, 1H), 3.12 (qd, *J* = 6.9, 1.9 Hz, 1H), 2.63-2.57 (m, 1H), 2.52 - 2.42 (m, 1H), 1.84 - 1.73 (m, 1H), 1.68 - 1.59 (m, 2H), 1.56 - 1.43 (m, 5H), 1.37 - 1.16 (m, 7H), 0.94 - 0.91 (m, 3H), 0.84 (t, *J* = 7.1 Hz, 3H).

^{13}C NMR (101 MHz, $CDCl_3$, mixture of diastereomers) δ 174.6, 58.9, 58.8, 50.8, 50.7, 48.6, 48.4, 34.1, 34.1, 30.4, 30.3, 30.2, 24.2, 23.7, 23.7, 22.0, 22.0, 20.5, 20.4, 17.2, 17.0, 11.9, 11.9.

HRMS (ESI, *m/z*) calcd for $C_{13}H_{27}N_2O$ $[M+H]^+$: 227.2123, found: 227.2120.

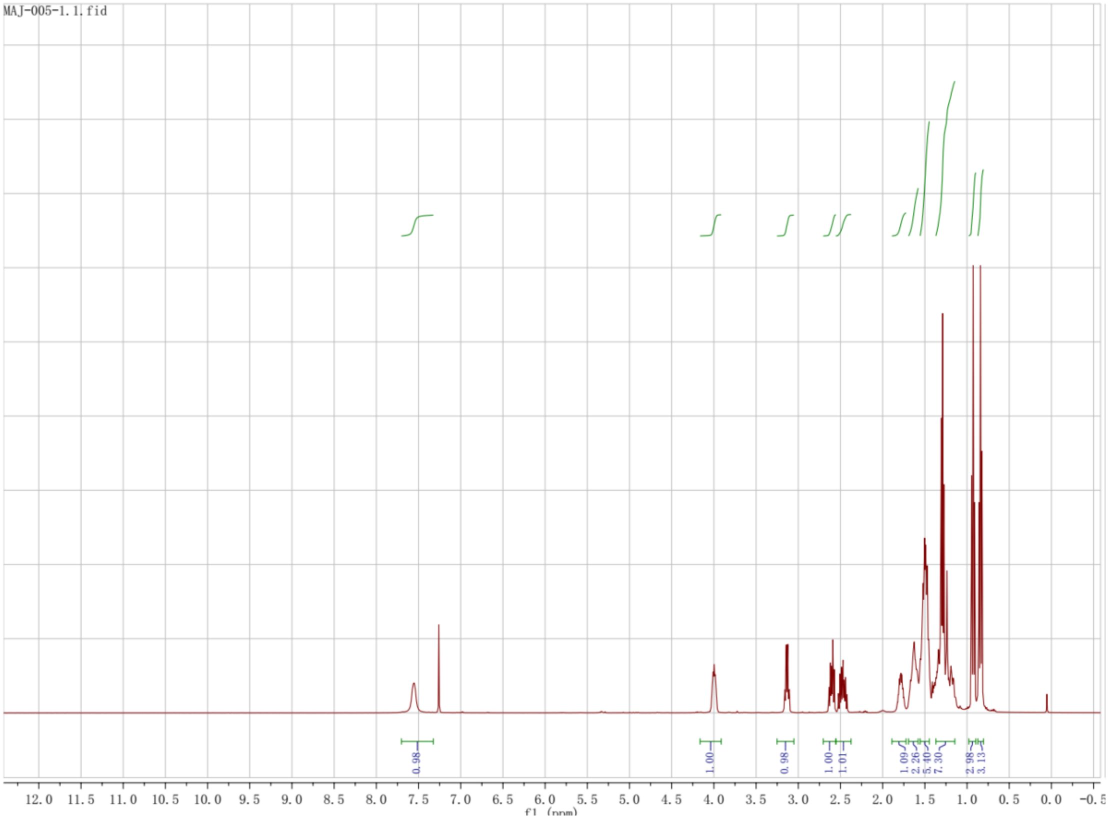

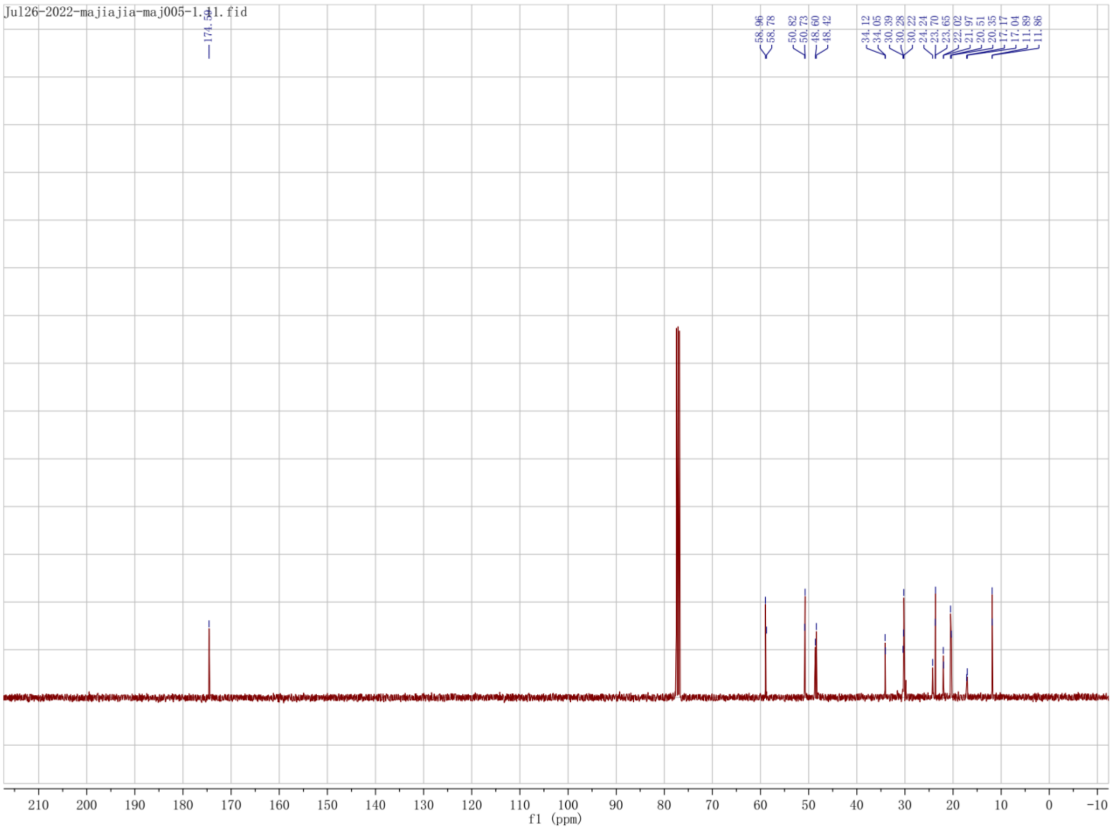

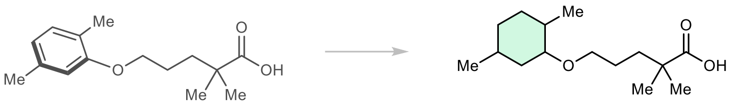

16 Gemfibrozil

Isolated diastereomers:
17a (59% yield), 17**b** (8% yield)

According to the general procedure, a mixture of **gemfibrozil** (100.1 mg, 0.4 mmol, 1.0 equiv.), $B_2(OH)_4$ (161.4 mg, 1.8 mmol, 4.5 equiv.) and $[Rh(COD)OH]_2$ (4.6 mg, 2.5 mol%, 0.010 mmol) in EtOH (2.0 mL, 0.2 M) was stirred under argon atmosphere for 14 hours at 50 °C to afford **17a** (60.6 mg, 59% yield) and **17b** (8.2 mg, 8% yield) as colorless oil.

Analytical data of **17a**:

^{1}H NMR (400 MHz, $CDCl_3$) δ 3.48 - 3.43 (m, 1H), 3.35 - 3.27 (m, 2H), 2.14 (m, 1H), 1.64 - 1.52 (m, 6H), 1.47 - 1.31 (m, 3H), 1.20 (s, 6H), 1.15 - 1.02 (m, 2H), 0.91 (d, J = 6.4 Hz, 3H), 0.86 (d, J = 7.1 Hz, 3H).

^{13}C NMR (151 MHz, $CDCl_3$) δ 184.5, 80.0, 68.1, 42.1, 37.1, 35.3, 32.0, 30.6, 30.4, 28.7, 25.8, 25.07, 25.06, 22.5, 11.2.

HRMS (ESI, m/z) calcd for $C_{15}H_{28}O_3Na^+$ $[M+Na]^+$: 279.1931, found: 279.1930.

Analytical data of **17b**:

^{1}H NMR (400 MHz, $CDCl_3$) δ 3.53 - 3.47 (m, 1H), 3.28 (m, 1H), 3.25 - 3.20 (m, 1H), 1.91 (dq, J =

13.6, 3.3 Hz, 1H), 1.65 - 1.59 (m, 4H), 1.57 - 1.50 (m, 3H), 1.42 - 1.33 (m, 3H), 1.21 (s, 6H), 0.93 - 0.84 (m, 8H).

^{13}C NMR (151 MHz, $CDCl_3$) δ 183.0, 78.7, 69.2, 42.0, 38.3, 37.3, 36.4, 35.0, 29.0, 26.0, 25.9, 25.2, 25.1, 22.6, 18.5.

HRMS (ESI, *m*/*z*) calcd for $C_{15}H_{28}O_3Na^+$ $[M+Na]^+$: 279.1931, found: 279.1931.

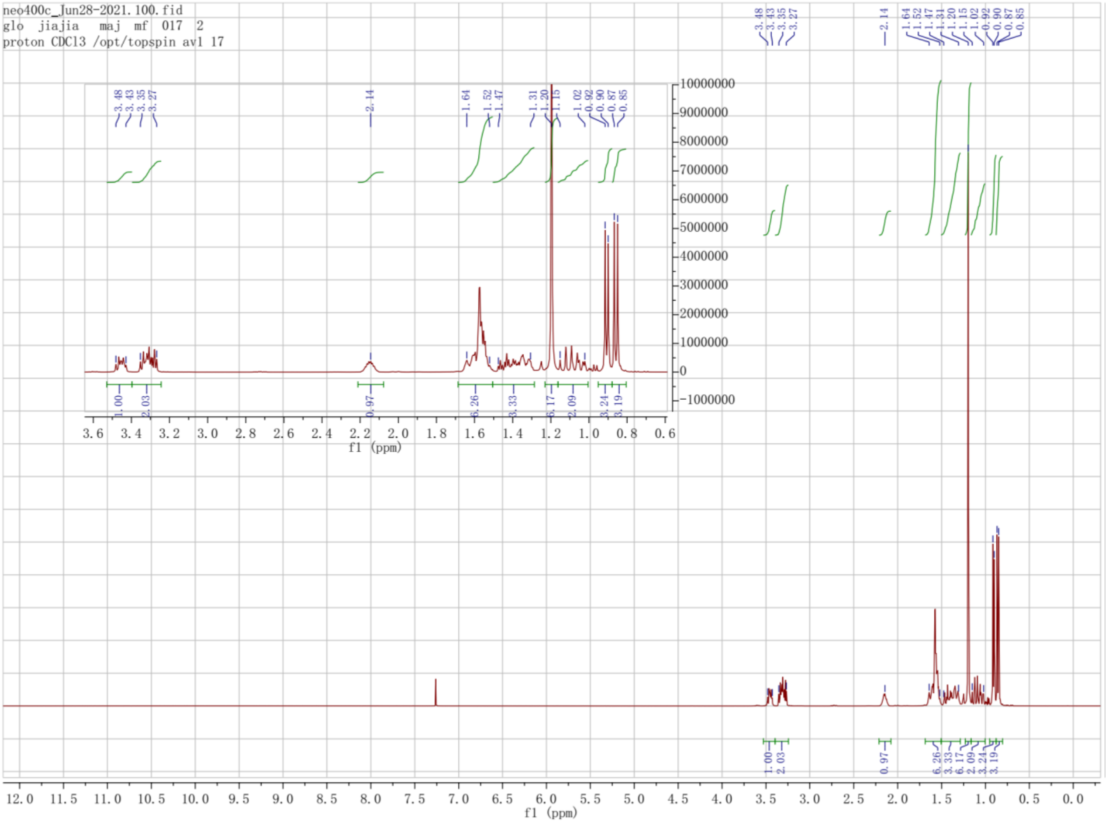

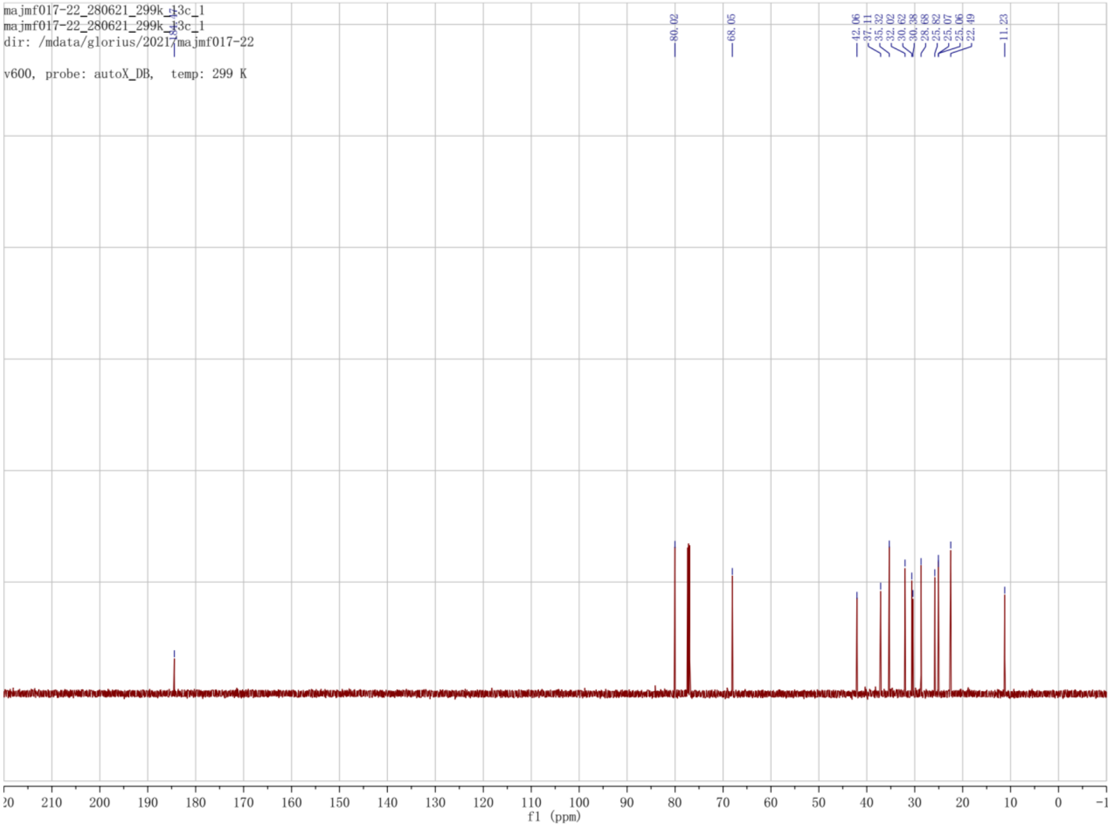
majmf017-22_280621_299k_13c_1
majmf017-22_280621_299k_13c_1
dir: /mdata/glorius/2021/majmf017-22
v600, probe: autoX_DB, temp: 299 K
80.02
68.05
42.06
37.11
35.32
32.02
30.62
30.38
28.88
25.82
25.07
25.06
22.49
11.23
f1 (ppm)

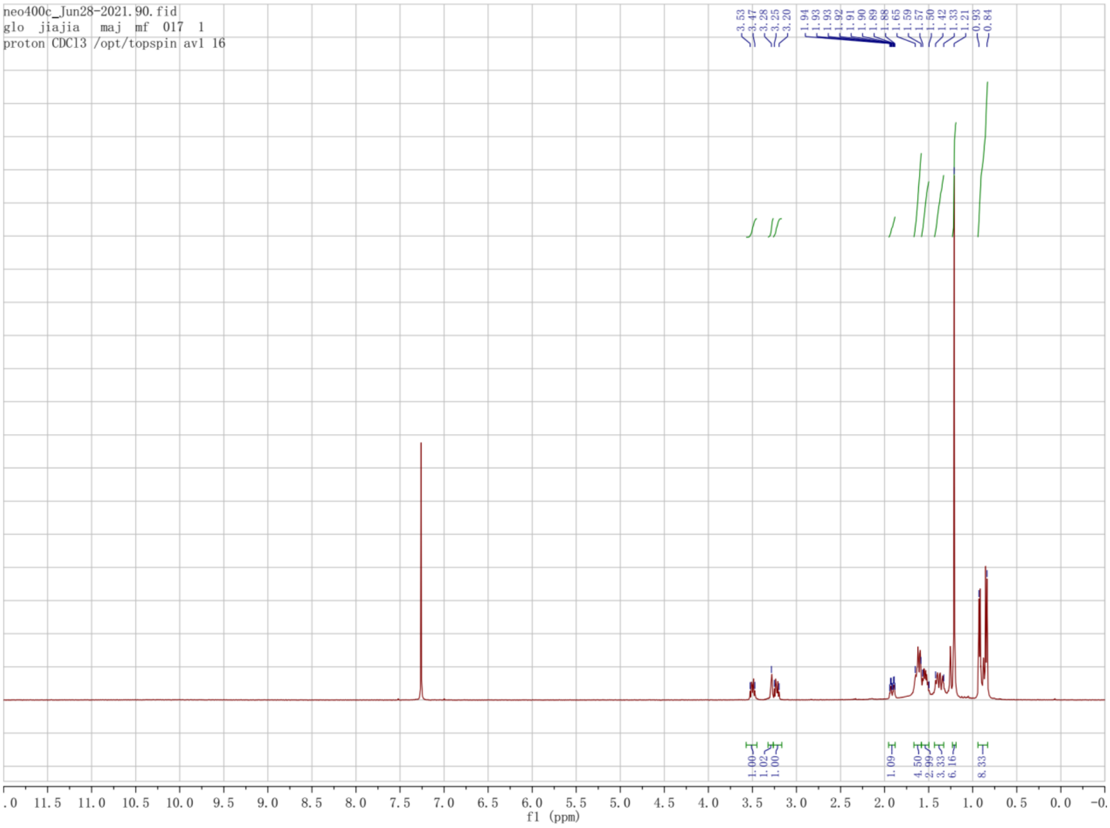
neo400c_Jun28-2021.90.fid
glo jiajia maj mf 017 1
proton CDCl3 /opt/topspin av1 16
3.53
3.47
3.28
3.25
3.20
1.94
1.93
1.93
1.92
1.91
1.90
1.89
1.88
1.65
1.59
1.57
1.50
1.42
1.33
1.21
0.93
0.84
1.00
1.02
1.00
1.09
4.50
2.99
3.33
6.16
8.33
f1 (ppm)

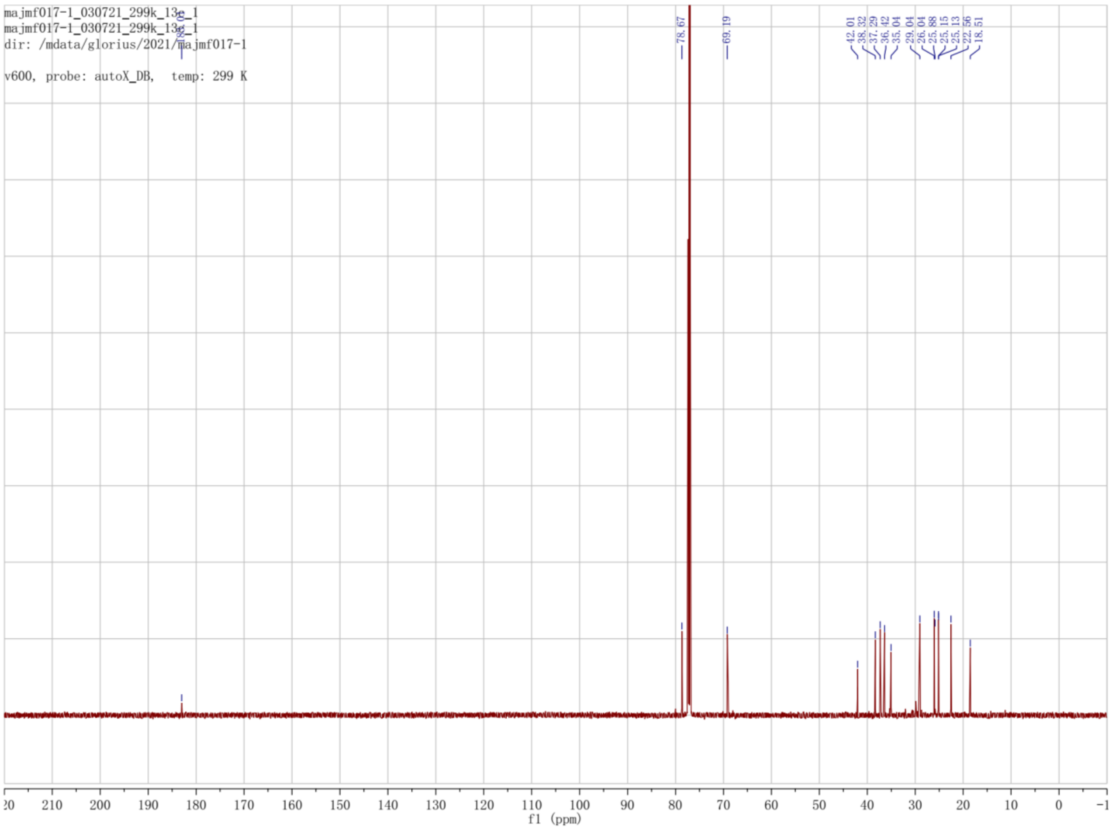

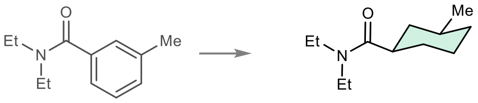

18 Diethyltoluamide (DEET) **19** (80%, >95:5 d.r.)

According to the general procedure, a mixture of **diethyltoluamide** (38.3 mg, 0.20 mmol, 1.0 equiv.), $B_2(OH)_4$ (107.5 mg, 1.20 mmol, 6.0 equiv.) and $[Rh(COD)OH]_2$ (3.7 mg, 4.0 mol%, 0.008 mmol) in EtOH (1.0 mL, 0.2 M) was stirred under nitrogen atmosphere for 40 hours at 50 °C to afford **19** as a white oil (31.7 mg, 80% yield, >95:5 d.r.).

Purification conditions: petroleum ether/EtOAc = 10:1 to 6:1.

R_f = 0.5 in petroleum ether/EtOAc (2.5:1).

^{1}H NMR (400 MHz, $CDCl_3$) δ 3.39 - 3.27 (m, 4H), 2.42 (tt, J = 12.0, 4.0 Hz, 1H), 1.82 - 1.75 (m, 1H), 1.69 - 1.60 (m, 3H), 1.52 - 1.35 (m, 2H), 1.33 - 1.24 (m, 2H), 1.17 (t, J = 8.0 Hz, 3H), 1.08 (t, J = 8.0 Hz, 3H), 0.98 - 0.93 (m, 1H), 0.90 (d, J = 6.4 Hz, 3H).

^{13}C NMR (100 MHz, $CDCl_3$) δ 175.5, 41.8, 40.8, 40.2, 38.2, 34.6, 32.4, 29.3, 25.9, 22.8, 15.1, 13.3.

HRMS (ESI, m/z) calcd for $C_{12}H_{24}NO$ $[M+H]^+$: 198.1858, found: 198.1855.

Jul26-2022-majiajia-ldh01053-p-1.10.fid

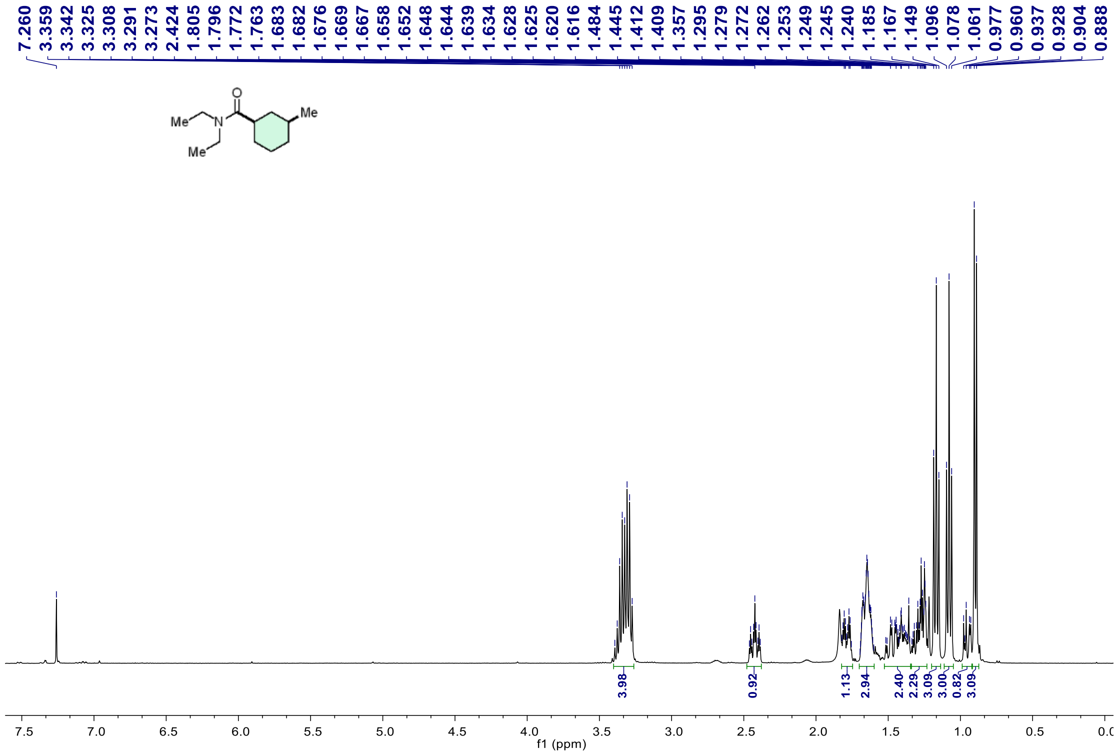

Jul26-2022-majiajia-ldh01053p1.10.fid

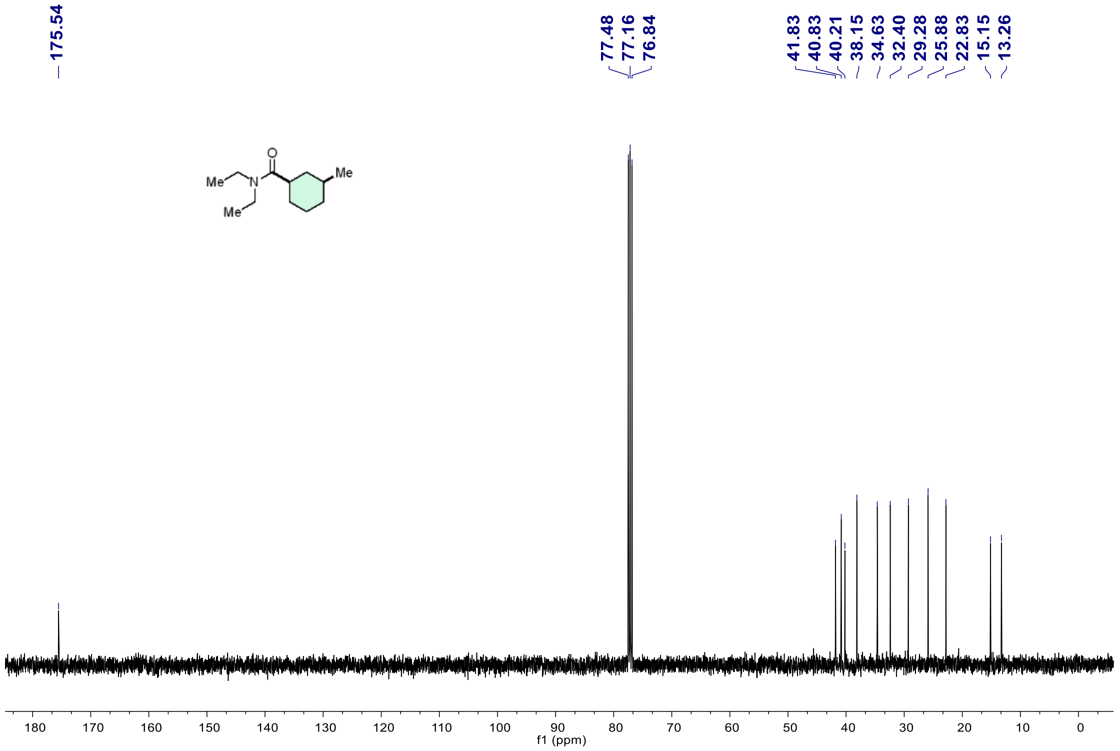

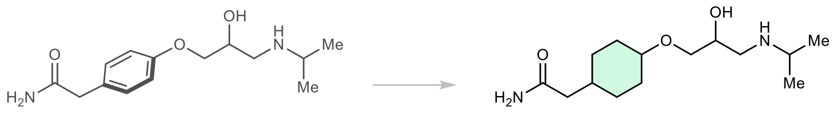

20 (±)-Atenolol **21** (91% yield, 3:1 d.r.)

According to the general procedure, a mixture of **(±)-atenolol** (53.3 mg, 0.2 mmol, 1.0 equiv.), $B_2(OH)_4$ (89.6 mg, 1.0 mmol, 5.0 equiv.) and $[Rh(COD)OH]_2$ (2.3 mg, 2.5 mol%, 0.005 mmol) in EtOH (1.0 mL, 0.2 M) was stirred under argon atmosphere for 14 hours at 50 °C to afford **21** as a colorless oil (49.3 mg, 91% yield, 3:1 d.r.).

^{1}H NMR (600 MHz, $CDCl_3$, mixture of diastereomers) δ 5.97 - 5.73 (m, 2H), 3.79 (m, 1H), 3.50 (m, 1H, major), 3.47 - 3.44 (m, 1H, minor), 3.41 - 3.33 (m, 2H, major; m, 1H, minor), 3.19 - 3.14 (m, 1H, minor), 2.82 - 2.70 (m, 4H), 2.60 - 2.54 (m, 1H), 2.08 (d, J = 7.1 Hz, 2H, major), 2.05 (d, J = 7.1 Hz, 2H, minor), 2.01 - 1.99 (m, 2H, minor), 1.85 - 1.70 (m, 3H), 1.51 - 1.48 (m, 2H, major), 1.45 - 1.41 (m, 2H, major), 1.34 - 1.28 (m, 2H, major), 1.26 - 1.19 (m, 2H, minor), 1.05 - 1.03 (m, 6H), 1.00 - 0.93 (m, 1H, minor).

^{13}C NMR (151 MHz, $CDCl_3$, mixture of diastereomers) δ 175.2 (major), 175.0 (minor), 78.6, 74.1, 71.0 (minor), 70.8 (major), 69.26 (minor), 69.24 (major), 49.9 (major), 49.8 (minor), 48.95 (minor), 48.94 (major), 43.1 (minor), 43.0 (major), 34.5, 34.00 (major), 31.9, 31.9, 31.1, 29.3, 29.2, 27.1 (major), 22.96 (minor), 22.93 (major), 22.90 (major).

HRMS (ESI, m/z) calcd for $C_{14}H_{29}N_2O_3$ $[M+H]^+$: 273.2173, found: 273.2171.

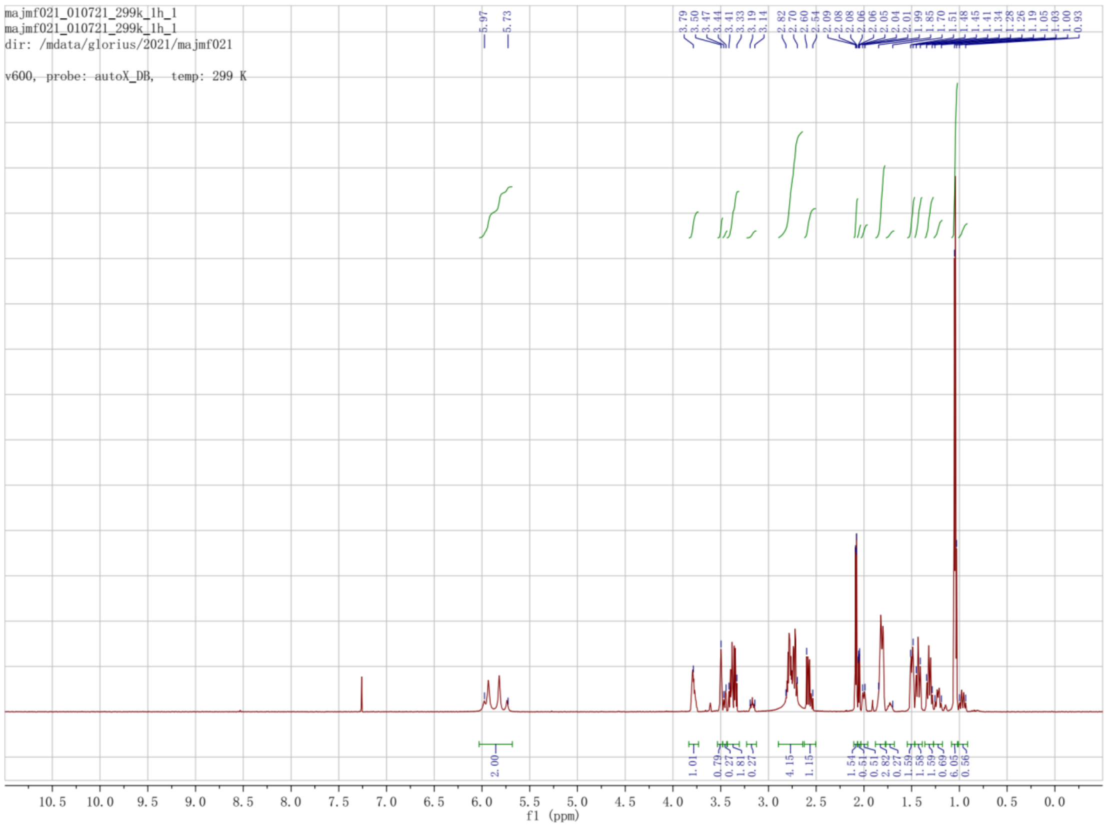
majmf021_010721_299k_1h_1
majmf021_010721_299k_1h_1
dir: /mdata/glorius/2021/majmf021
v600, probe: autoX_DB, temp: 299 K
5.97
5.73
3.79 3.50 3.47 3.44 3.41 3.33 3.19 3.14 2.82 2.70 2.60 2.54 2.09 2.08 2.08 2.06 2.06 2.05 2.04 2.01 1.99 1.85 1.70 1.51 1.48 1.45 1.41 1.34 1.28 1.26 1.19 1.05 1.03 1.00 0.93
2.00
1.01 0.79 0.27 1.81 0.27 4.15 1.15 1.54 0.51 0.51 2.82 0.27 1.59 1.58 1.59 0.69 6.05 0.56
f1 (ppm)

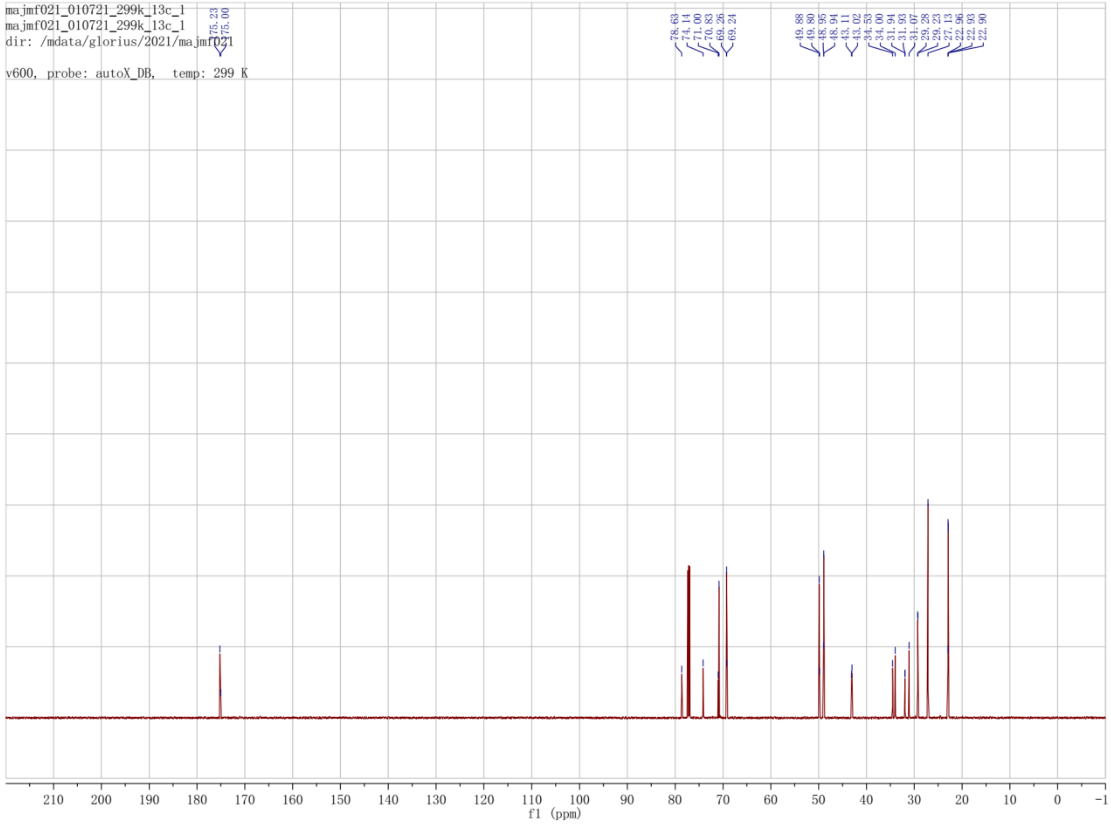
majmf021_010721_299k_13c_1
majmf021_010721_299k_13c_1
dir: /mdata/glorius/2021/majmf021
v600, probe: autoX_DB, temp: 299 K
175.23
175.00
78.63 74.14 71.00 70.83 69.26 69.24
49.88 49.80 48.95 48.94 43.11 43.02 34.53 34.00 31.94 31.93 31.67 29.28 29.23 27.13 22.96 22.93 22.90
f1 (ppm)

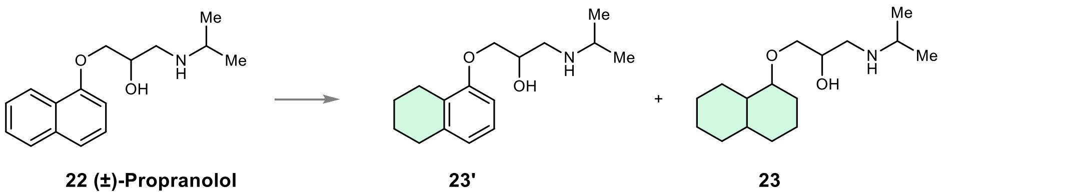

2.5 mol% [Rh], 4.5 eq. [B]: **23'** (83% yield), **23** (<5% yield)

5.0 mol% [Rh], 7.0 eq. [B]: **23'** (41% yield), **23** (38% yield,>95:5 d.r.)

According to the general procedure, a mixture of **(±)-propranolol** (51.9 mg, 0.2 mmol, 1.0 equiv.), $B_2(OH)_4$ (80.6 mg, 0.9 mmol, 4.5 equiv.) and $[Rh(COD)OH]_2$ (2.3 mg, 2.5 mol%, 0.005 mmol) in EtOH (1.0 mL, 0.2 M) was stirred under argon atmosphere for 14 hours at 50 °C to afford **23'** (43.1 mg, 83% yield) as colorless oil.

Under an alternative condition with $B_2(OH)_4$ (113.3 mg, 1.4 mmol, 7.0 equiv.) and $[Rh(COD)OH]_2$ (4.6 mg, 5 mol%, 0.01 mmol), **23'** (25.1 mg, 41% yield) and **23** (19.9 mg, 38% yield, >95:5 d.r.) were afforded as colorless oil.

Analytical data of **23'**:

^{1}H NMR (400 MHz, $CDCl_3$) δ 9.49 (br, 1H), 8.48 (br, 1H), 7.01 (m, 1H), 6.70 (d, *J* = 7.7 Hz, 1H), 6.56 (d, *J* = 8.1 Hz, 1H), 4.62 (m, 1H), 4.06 (dd, *J* = 9.6, 4.2 Hz, 1H), 3.93 (dd, *J* = 9.6, 6.1 Hz, 1H), 3.48 (m, 1H), 3.32 (m, 1H), 3.18 (m, 1H), 2.72 (t, *J* = 5.9 Hz, 2H), 2.62 (t, *J* = 6.1 Hz, 2H) 1.81 - 1.66 (m, 4H), 1.50 - 1.47 (m, 6H).

^{13}C NMR (101 MHz, $CDCl_3$) δ 155.9, 138.9, 126.1, 125.9, 122.3, 107.9, 69.5, 66.0, 51.6, 48.5, 29.7, 23.2, 22.9, 19.2, 19.1.

HRMS (ESI, *m*/*z*) calcd for $C_{16}H_{26}NO_2^+$ $[M+H]^+$: 264.1958, found: 264.1956.

Analytical data of **23**:

^{1}H NMR (400 MHz, $CDCl_3$, mixture of diastereomers) δ 3.87 - 3.81 (m, 1H), 3.57 - 3.47 (m, 1H), 3.44 - 3.36 (m, 1H), 3.30 (dt, *J* = 11.5, 4.6 Hz, 1H), 2.87 - 2.77 (m, 2H), 2.64 (ddd, *J* = 11.9, 7.8, 1.7 Hz, 1H), 2.54 (br, 2H), 2.05 - 1.96 (m, 1H), 1.80 - 1.76 (m, 2H), 1.70 - 1.66 (m, 1H), 1.63 - 1.54 (m, 3H), 1.53 - 1.29 (m, 6H), 1.27 - 1.14 (m, 3H), 1.10 (d, *J* = 6.3 Hz, 6H).

^{13}C NMR (101 MHz, $CDCl_3$, mixture of diastereomers) δ 81.7, 81.6, 70.73, 70.69, 69.3, 49.91, 49.90, 49.08, 49.06, 39.97, 39.89, 35.8, 32.1, 26.78, 26.73, 26.5, 25.01, 24.49, 22.96, 22.93, 21.7, 19.6.

HRMS (ESI, *m*/*z*) calcd for $C_{16}H_{32}NO_2^+$ $[M+H]^+$: 270.2428, found: 270.2426.

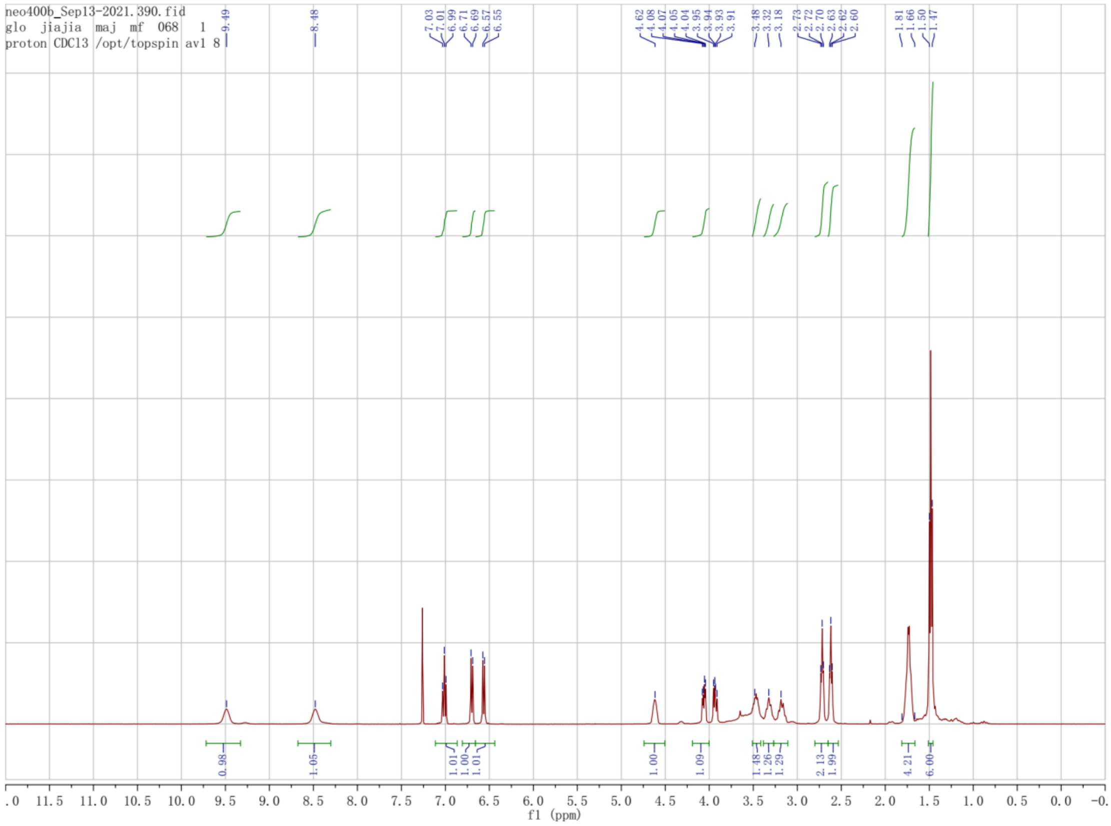
neo400b_Sep13-2021.390.fid
glo jiajia maj mf 068 1
proton CDCl3 /opt/topspin av1 8
9.49
8.48
7.03 7.01 6.99 6.71 6.69 6.57 6.55
4.62 4.08 4.07 4.05 4.04 3.95 3.94 3.93 3.91
3.48 3.32 3.18
2.73 2.72 2.70 2.63 2.62 2.60
1.81 1.66 1.50 1.47
0.98 1.05 1.01 1.00 1.01 1.00 1.09 1.48 1.26 1.29 2.13 1.99 4.21 6.00
f1 (ppm)

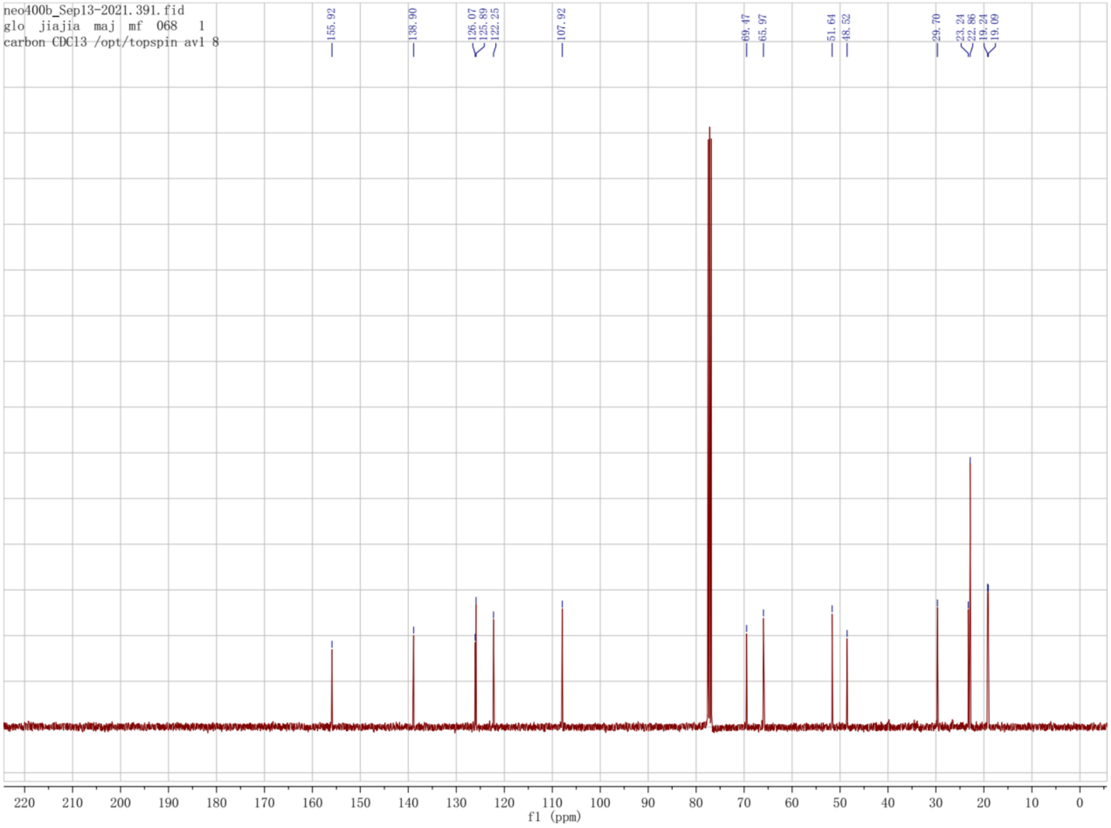
neo400b_Sep13-2021.391.fid
glo jiajia maj mf 068 1
carbon CDCl3 /opt/topspin av1 8
155.92
138.90
126.07 125.89 122.25
107.92
69.47 65.97
51.64 48.52
29.70 23.24 22.86 19.24 19.09
f1 (ppm)

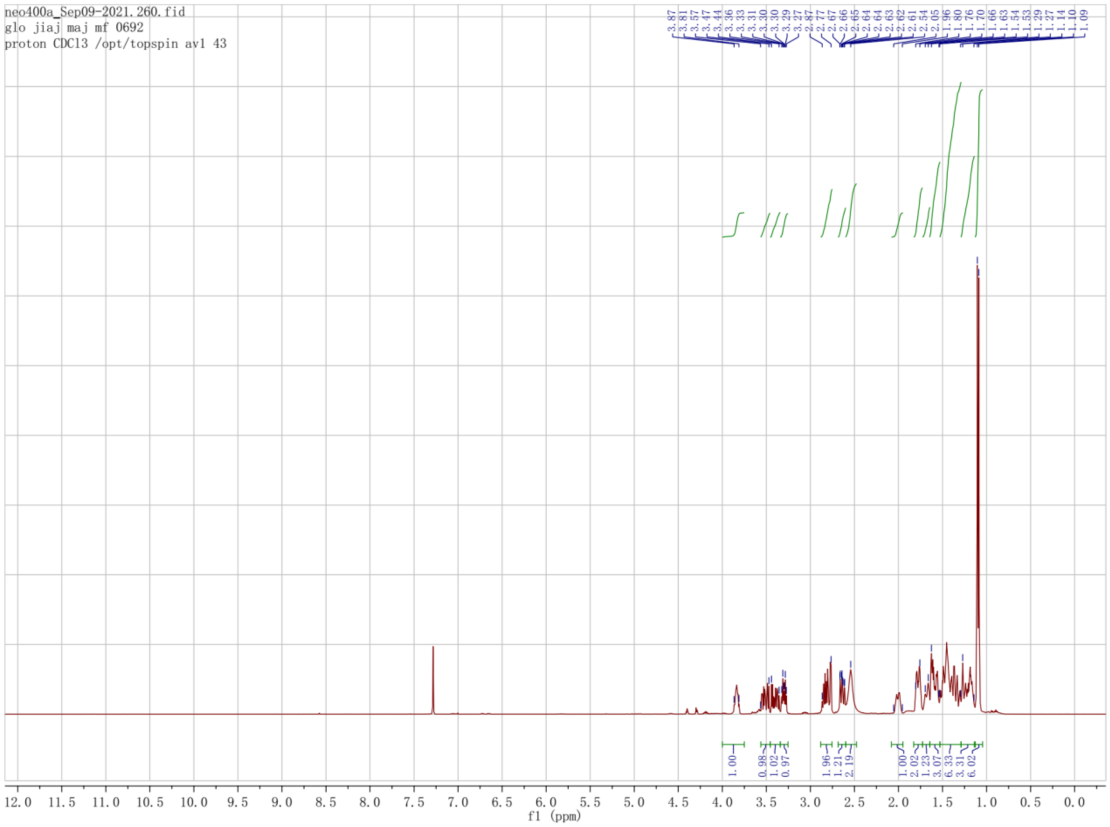
neo400a_Sep09-2021.260.fid
glo jiaj maj mf 0692
proton CDCl3 /opt/topspin av1 43
3.87 3.81 3.57 3.47 3.44 3.36 3.33 3.31 3.30 3.29 3.27 2.87 2.77 2.67 2.66 2.65 2.64 2.64 2.63 2.62 2.61 2.54 2.05 1.96 1.80 1.76 1.70 1.66 1.63 1.54 1.53 1.29 1.27 1.14 1.10 1.09
1.00 0.98 1.02 0.97 1.96 1.21 2.19 1.00 2.02 1.23 3.07 6.33 3.31 6.02
12.0 11.5 11.0 10.5 10.0 9.5 9.0 8.5 8.0 7.5 7.0 6.5 6.0 5.5 5.0 4.5 4.0 3.5 3.0 2.5 2.0 1.5 1.0 0.5 0.0
f1 (ppm)

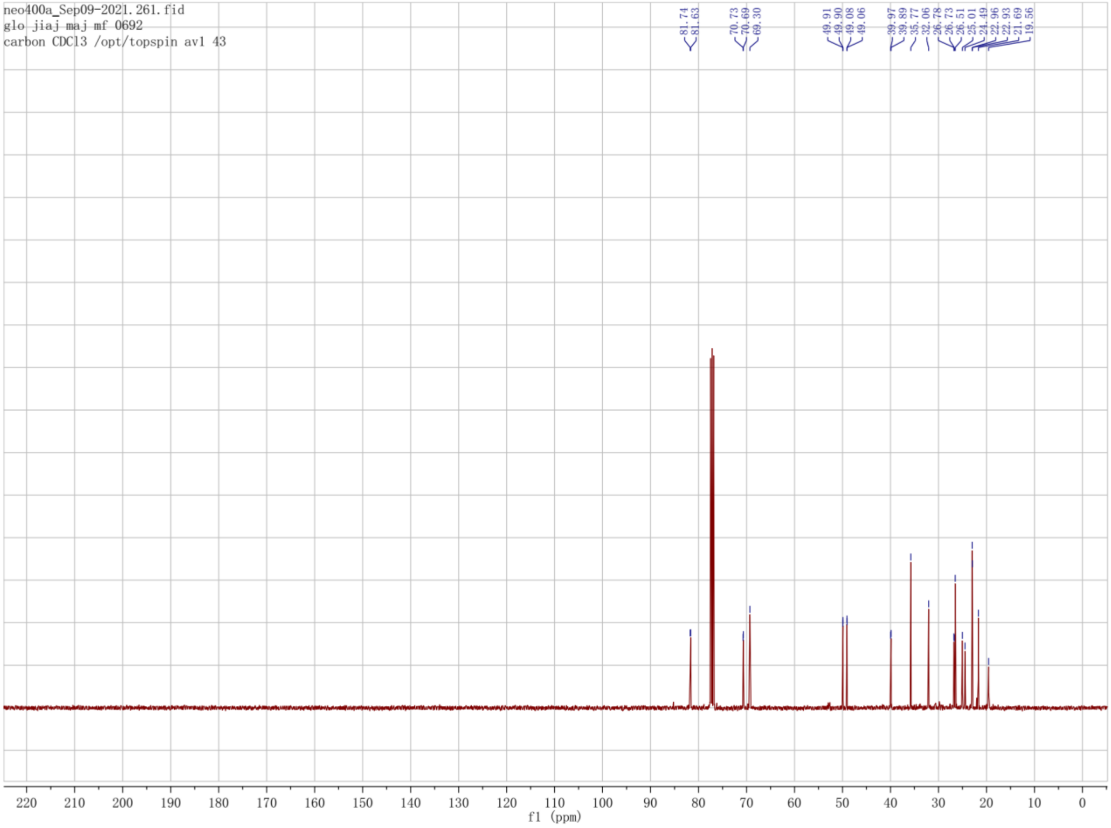
neo400a_Sep09-2021.261.fid
glo jiaj maj mf 0692
carbon CDCl3 /opt/topspin av1 43
81.74 81.63 70.73 70.69 69.30 49.91 49.90 49.08 49.06 39.97 39.89 35.77 32.06 26.78 26.73 26.51 25.01 24.49 22.96 22.93 21.69 19.56
220 210 200 190 180 170 160 150 140 130 120 110 100 90 80 70 60 50 40 30 20 10 0
f1 (ppm)

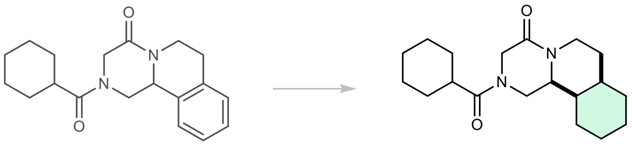

24 (±)-Praziquantel **25** (67% yield, >95:5 d.r.)

According to the general procedure, a mixture of **(±)-praziquantel** (62.4 mg, 0.2 mmol, 1.0 equiv.), $B_2(OH)_4$ (80.6 mg, 0.9 mmol, 4.5 equiv.) and $[Rh(COD)OH]_2$ (4.6 mg, 5 mol%, 0.010 mmol) in EtOH (1.0 mL, 0.2 M) was stirred under argon atmosphere for 14 hours at 50 °C to afford **25** as a colorless oil (42.5 mg, 67% yield, >95:5 d.r.).

^{1}H NMR (400 MHz, $CDCl_3$) δ 4.75-4.72 (m, 1H), 4.19 (d, J = 17.3 Hz, 1H), 4.07 - 4.00 (m, 2H), 3.54 (dd, J = 13.9, 5.6 Hz, 1H), 3.39 (td, J = 5.1, 3.0 Hz, 1H), 2.55 (dt, J = 12.0, 6.2 Hz, 1H), 2.39 (tt, J = 11.6, 3.5 Hz, 1H), 1.92 - 1.66 (m, 10H), 1.57 - 1.18 (m, 12H).

^{13}C NMR (101 MHz, $CDCl_3$) δ 174.4, 164.4, 58.3, 49.1, 43.1, 41.2, 41.1, 40.7, 35.9, 31.6, 29.3, 28.8, 25.96, 25.92, 25.8, 25.7, 24.5, 20.5, 19.9.

HRMS (ESI, m/z) calcd for $C_{19}H_{30}N_2O_2Na^+$ $[M+Na]^+$: 341.2199, found: 341.2200.

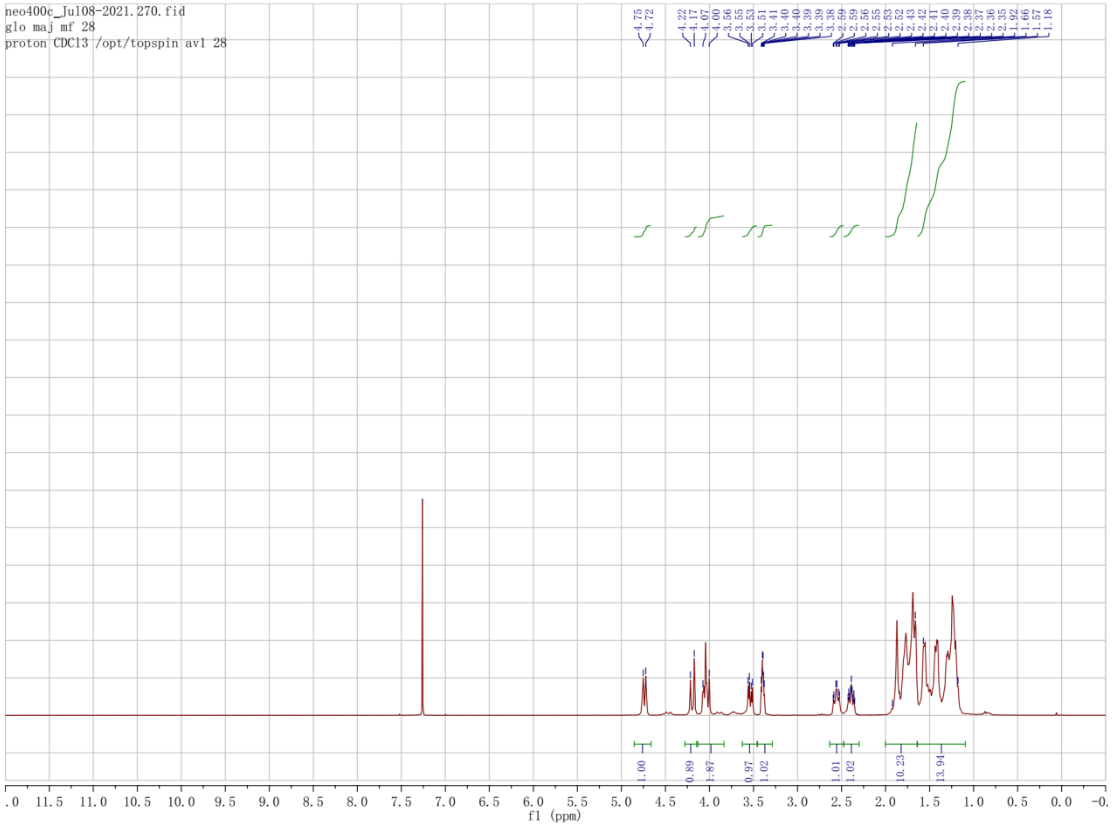

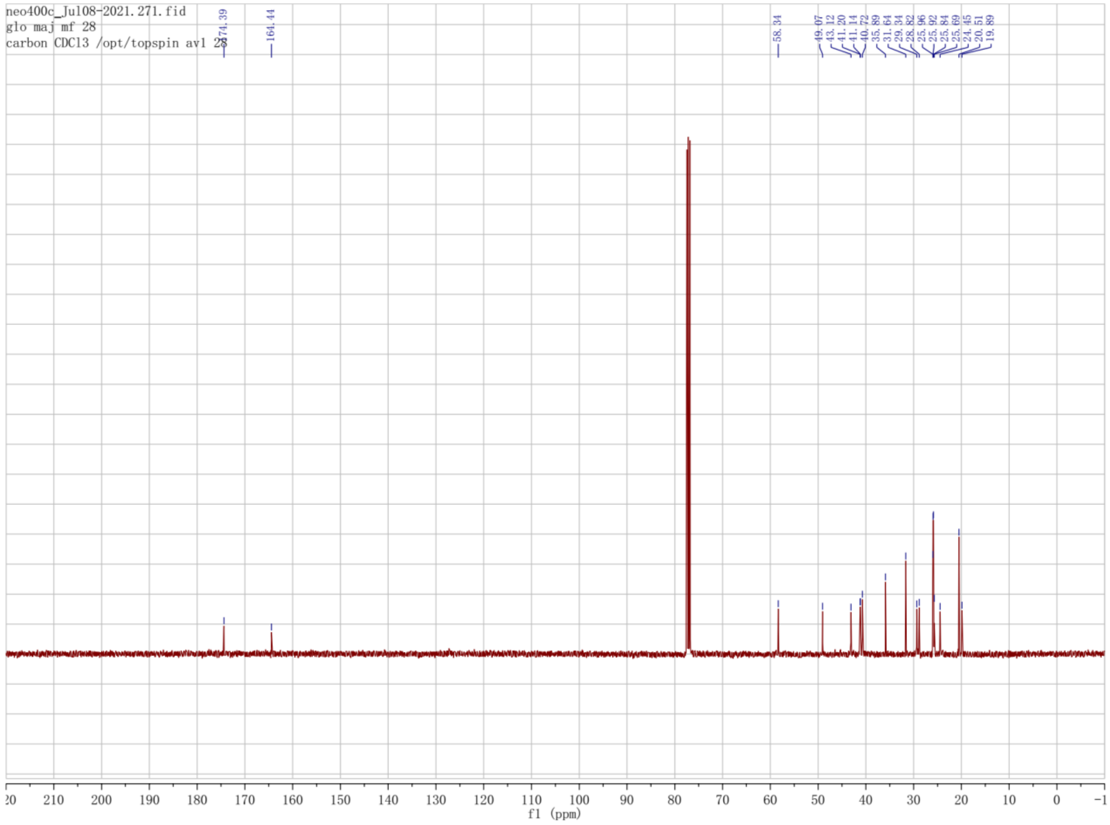

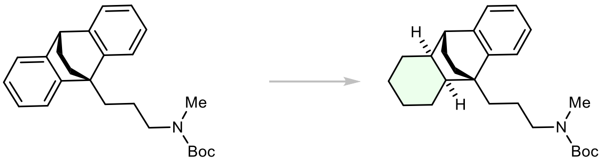

Boc-Maprotiline **26** (86% yield)

According to the general procedure, a mixture of **Boc-Maprotiline** (75.4mg, 0.20 mmol, 1.0 equiv.), $B_2(OH)_4$ (80.6 mg, 0.9 mmol, 4.5 equiv.) and $[Rh(COD)OH]_2$ (3.7 mg, 4.0 mol%, 0.008 mmol) in EtOH (1.0 mL, 0.2 M) was stirred under nitrogen atmosphere for 48 hours at 80 °C to afford **26** as a white solid (66.5 mg, 86% yield, >95:5 d.r.)

Purification conditions: petroleum ether/CH_2Cl_2 = 5:1 to 2:1.

R_f = 0.3 in petroleum ether/ CH_2Cl_2 = 1:1

^{1}H NMR (400 MHz, $CDCl_3$) δ 7.24 - 7.08 (m, 4H), 3.31 (br, 2H), 2.91 (s, 3H), 2.70 - 2.65 (m, 1H), 2.06 - 1.94 (m, 1H), 1.90 - 1.55 (m, 8H), 1.54 - 1.44 (m, 11H), 1.43 - 1.24 (m, 5H), 1.23 - 1.10 (m, 2H).

^{13}C NMR (100 MHz, $CDCl_3$, mixture of rotamers) δ 155.99, 155.97, 146.2, 145.9, 143.8, 143.0, 125.6, 125.6, 125.4, 125.3, 123.4, 122.7, 121.6, 79.3, 79.3, 41.6, 41.1, 40.1, 40.1, 39.9, 39.4, 37.6, 34.6, 34.5, 30.6, 30.2, 30.0, 28.6, 27.2, 24.8, 23.0, 20.9, 20.6, 20.11, 20.09, 19.7, 18.9.

HRMS (ESI, *m*/*z*) calcd for $C_{25}H_{37}NO_2Na^+$ [M+Na]$^+$: 406.2717, found: 406.2715.

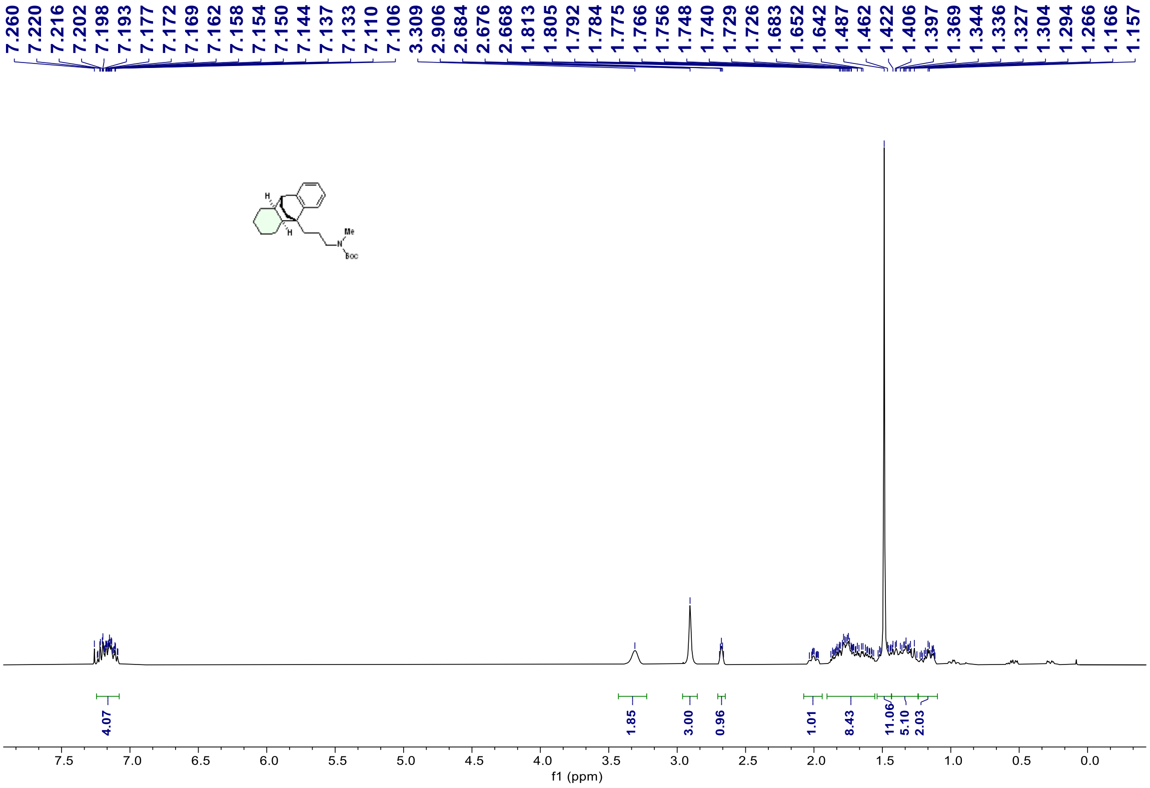

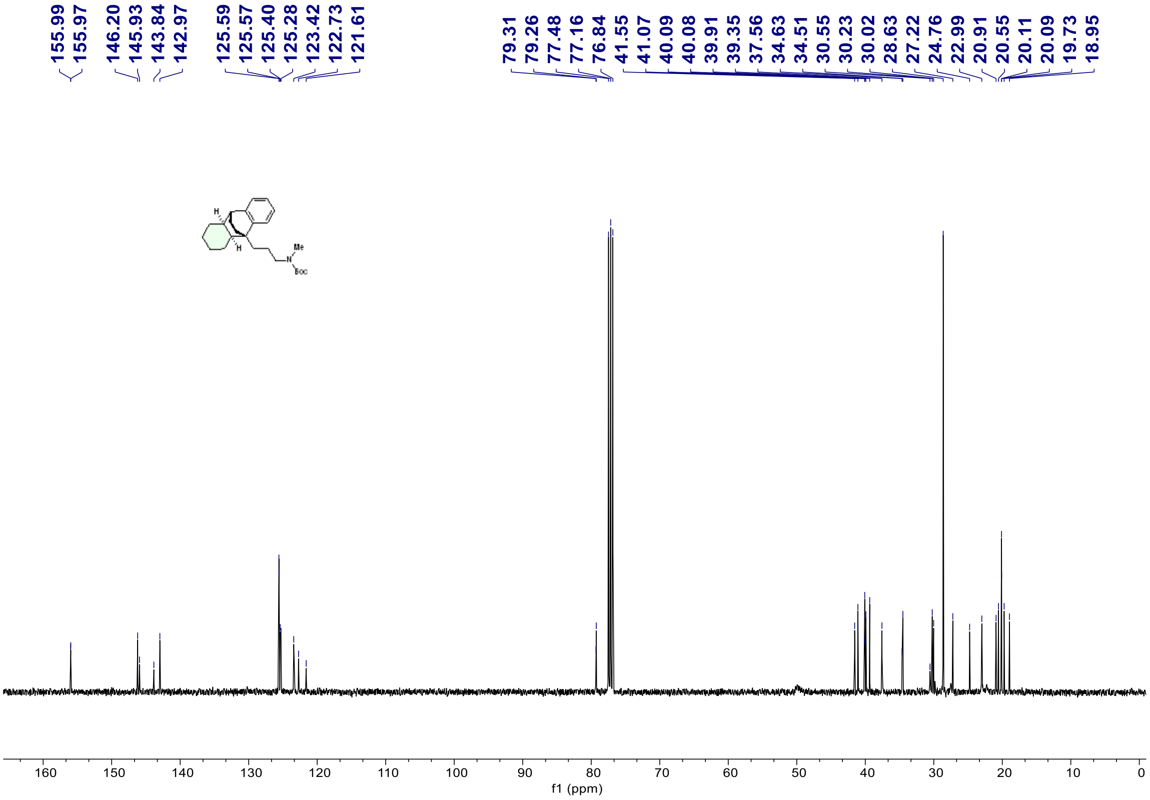

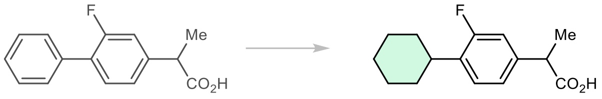

(±)-Flurbiprofen **27** (55% yield)

According to the general procedure, a mixture of **(±)-flurbiprofen** (48.8 mg, 0.20 mmol, 1.0 equiv.), $B_2(OH)_4$ (80.6 mg, 0.90 mmol, 4.5 equiv.) and $[Rh(COD)OH]_2$ (2.4 mg, 2.5 mol%, 0.008 mmol) in EtOH (3.0 mL, 0.07 M) was stirred under nitrogen atmosphere for 40 hours at 50 °C to afford **27** as a colorless oil (27.5 mg, 55% yield).

Purification conditions: petroleum ether/acetone = 30:1 to 5:1.

R_f= 0.25 in petroleum ether/acetone = 3:1.

^{1}H NMR (400 MHz, $CDCl_3$) δ 7.17 (t, *J* = 7.6 Hz, 1H), 7.03 (dd, *J* = 8.0, 2.0 Hz, 1H), 6.98 (dd, *J* = 11.6, 2.0 Hz, 1H), 3.69 (q, *J* = 7.2 Hz, 1H), 2.88 - 2.76 (m, 1H), 1.83 (d, *J* = 8.8 Hz, 4H), 1.75 (d, *J* = 13.6 Hz, 1H), 1.49 (d, *J* = 6.8 Hz, 3H), 1.45 - 1.34 (m, 4H), 1.30 - 1.23 (m, 1H).

^{13}C NMR (100 MHz, $CDCl_3$) δ 180.1, 160.68 (d, *J* = 243.0 Hz), 139.0 (d, *J* = 8.0 Hz), 133.7 (d, *J* = 15.0 Hz), 127.96.0 (d, *J* = 5.0 Hz), 123.4 (d, *J* = 3.0 Hz), 114.6 (d, *J* = 24.0 Hz), 44.8, 37.10, 37.09, 33.1, 27.0, 26.3, 18.1.

^{19}F NMR (376 MHz, $CDCl_3$) δ -118.98.

HRMS (ESI, *m/z*) calcd for $C_{15}H_{19}O_2FNa^+$ $[M+Na]^+$: 273.1267, found: 273.1262.

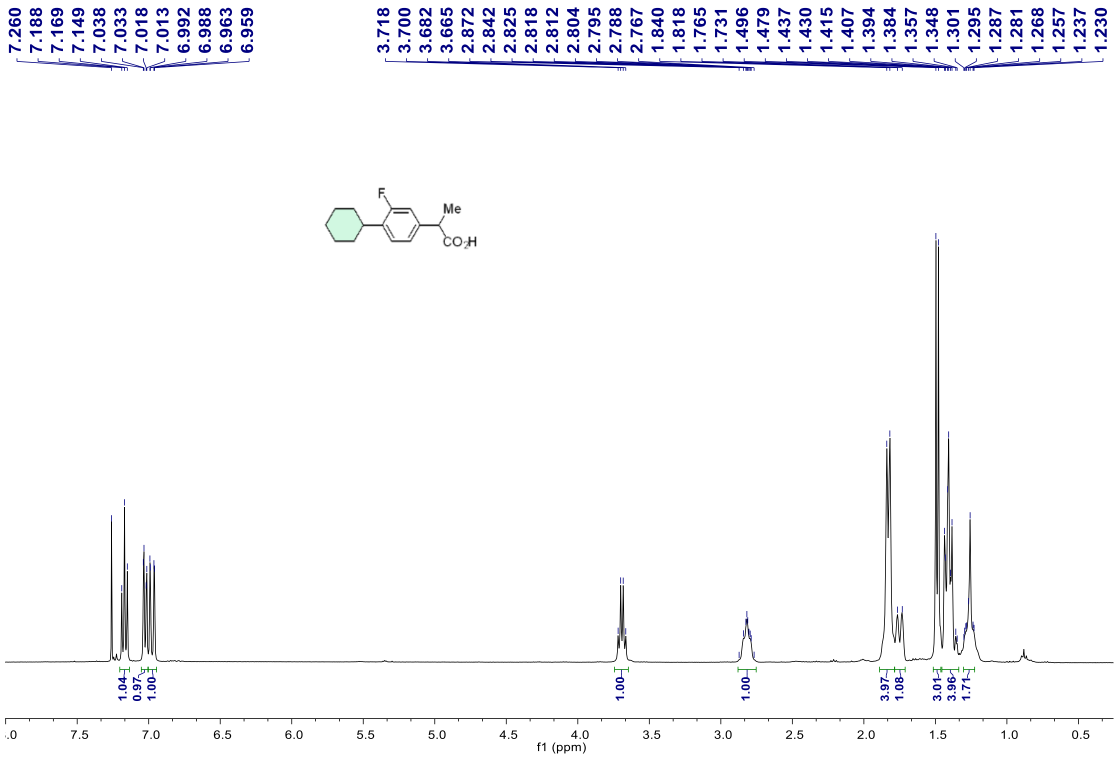

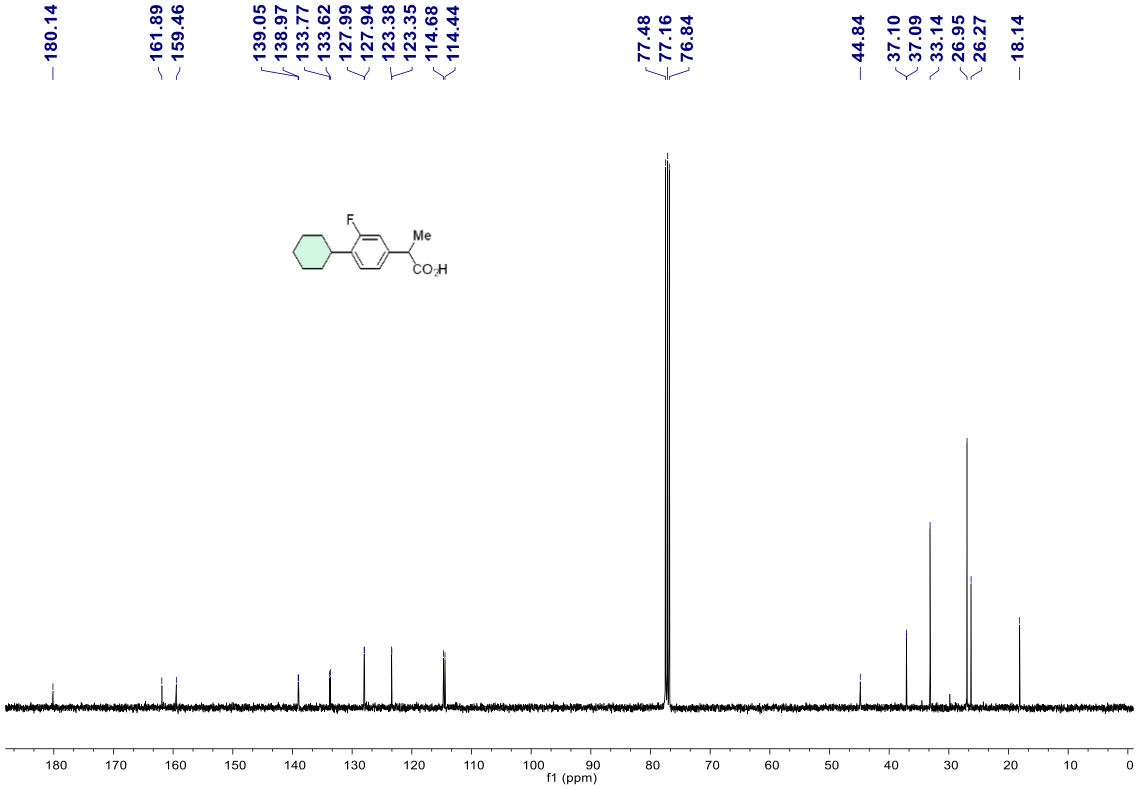
180.14
161.89
159.46
139.05
138.97
133.77
133.62
127.99
127.94
123.38
123.35
114.68
114.44
77.48
77.16
76.84
44.84
37.10
37.09
33.14
26.95
26.27
18.14
F
Me
CO2H
180 170 160 150 140 130 120 110 100 90 80 70 60 50 40 30 20 10 0
f1 (ppm)

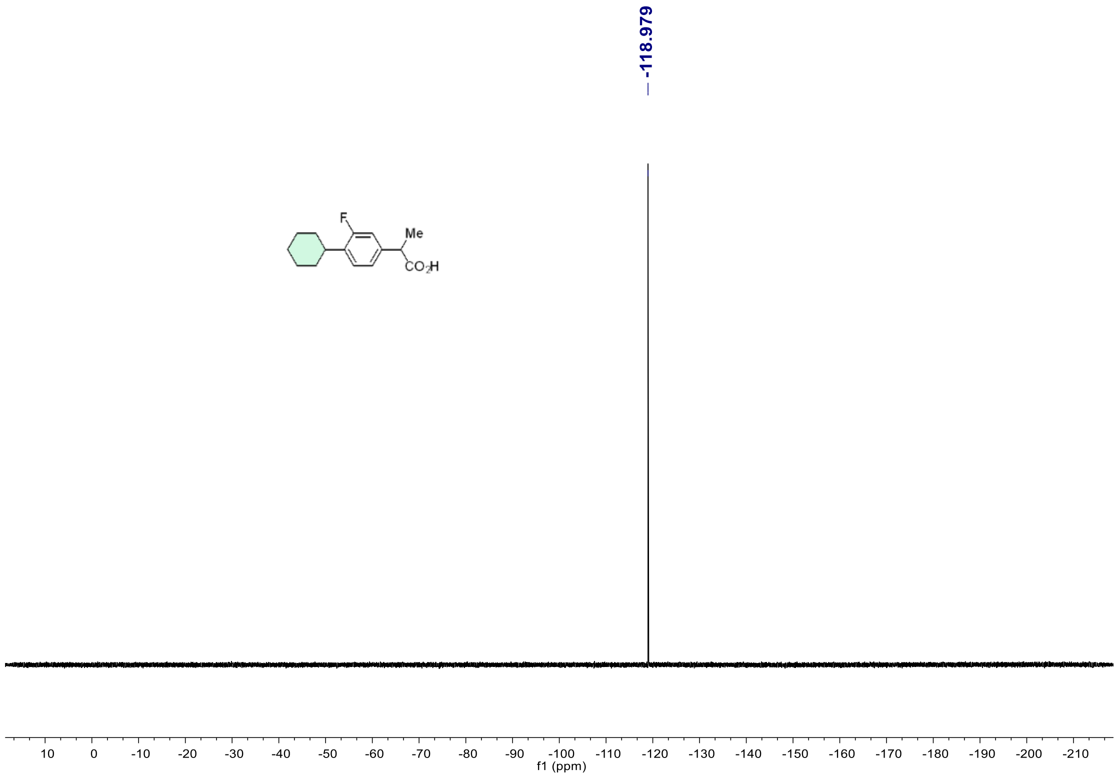
-118.979
F
Me
CO2H
10 0 -10 -20 -30 -40 -50 -60 -70 -80 -90 -100 -110 -120 -130 -140 -150 -160 -170 -180 -190 -200 -210
f1 (ppm)

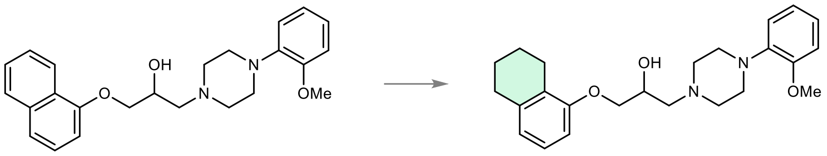

(±)-Naftopidil **28** (92% yield)

According to the general procedure, a mixture of **(±)-naftopidil** (78.50 mg, 0.2 mmol, 1.0 equiv.), $B_2(OH)_4$ (80.6 mg, 0.9 mmol, 4.5 equiv.) and $[Rh(COD)OH]_2$ (2.3 mg, 2.5 mol%, 0.005 mmol) in EtOH (1.0 mL, 0.2 M) was stirred under argon atmosphere for 14 hours at 50 °C to afford **28** as a colorless oil (72.0 mg, 92% yield).

^{1}H NMR (400 MHz, $CDCl_3$) δ 7.09 - 7.01 (m, 2H), 6.98 - 6.93 (m, 2H), 6.89 (d, J = 7.8 Hz, 1H), 6.73 (d, J = 7.7 Hz, 1H), 6.67 (d, J = 8.1 Hz, 1H), 4.23 - 4.18 (m, 1H), 4.05 (dd, J = 9.4, 5.1 Hz, 1H), 3.99 (dd, J = 9.6, 5.0 Hz, 1H), 3.88 (s, 3H), 3.57 (br, 1H), 3.15 (m, 4H), 3.04 - 2.89 (m, 2H), 2.78 - 2.69 (m, 8H), 1.80 - 1.75 (m, 4H).

^{13}C NMR (101 MHz, $CDCl_3$) δ 156.4, 152.3, 141.2, 138.7, 126.2, 125.7, 123.2, 121.8, 121.1, 118.32, 111.3, 107.9, 70.2, 65.8, 61.1, 55.4, 53.7, 50.7, 29.7, 23.2, 22.89, 22.86.

HRMS (ESI, m/z) calcd for $C_{24}H_{33}N_2O_3^+$ $[M+H]^+$: 397.2486, found: 397.2483.

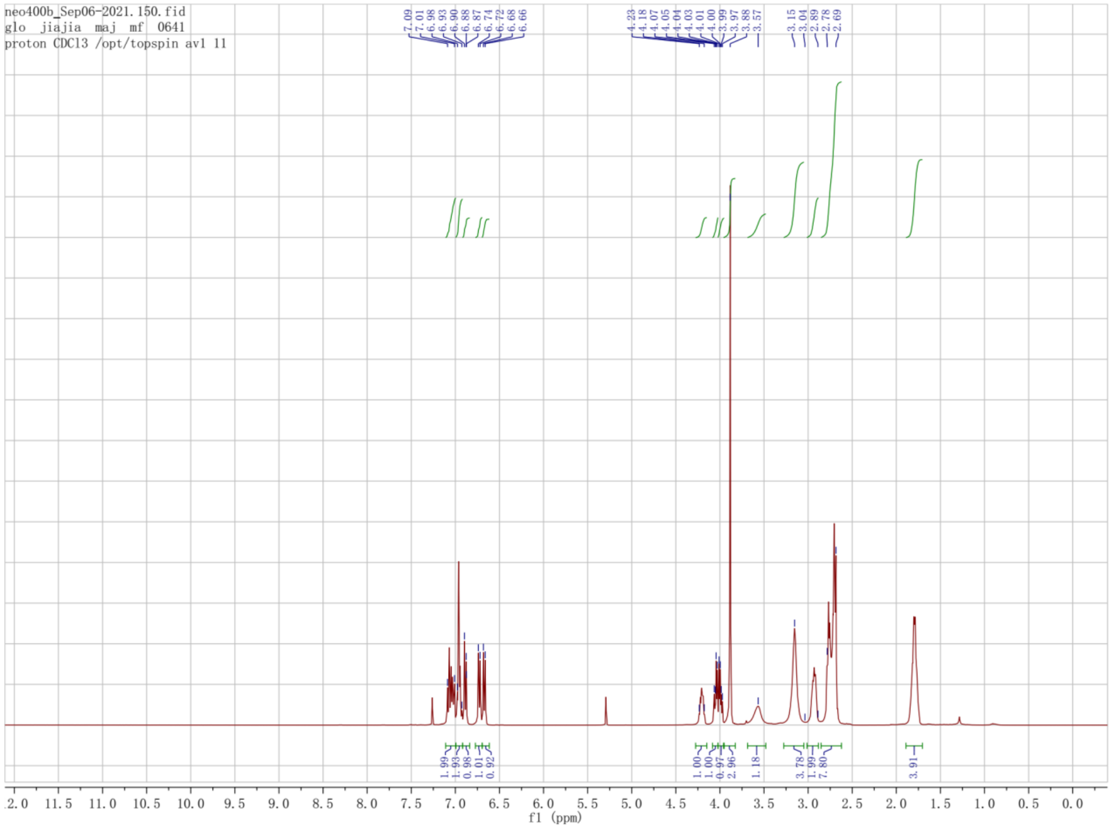

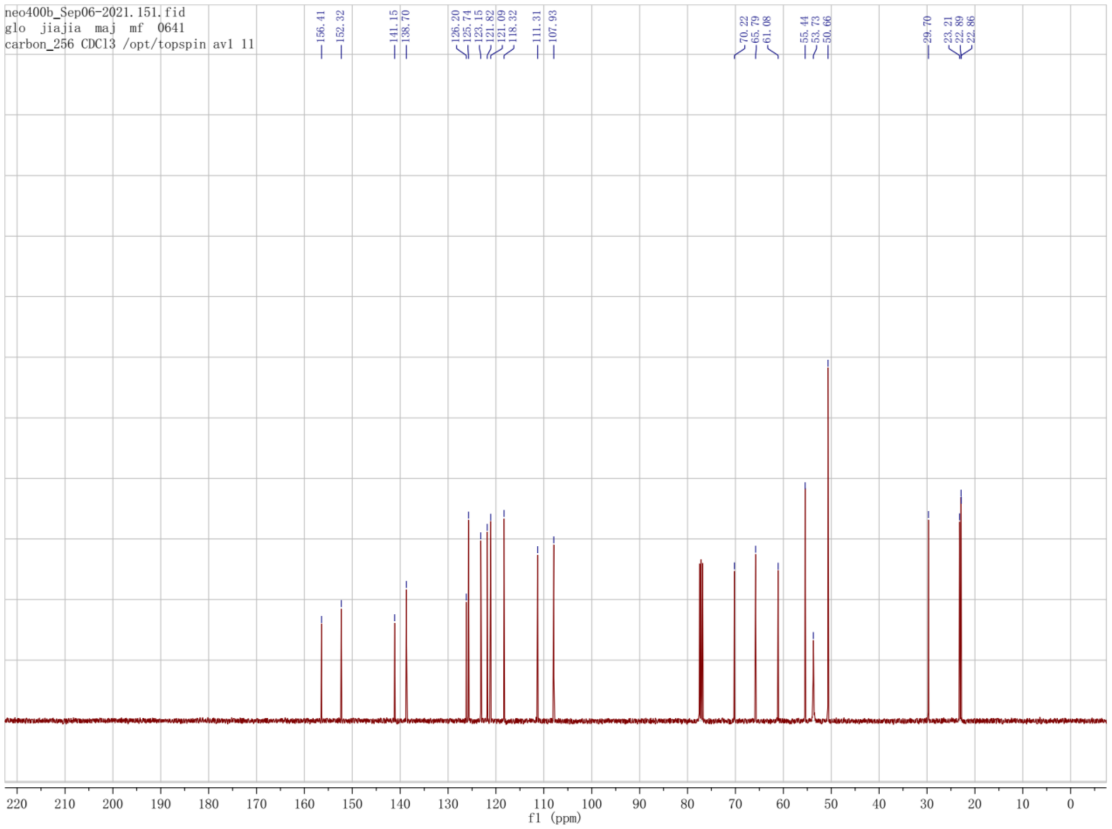

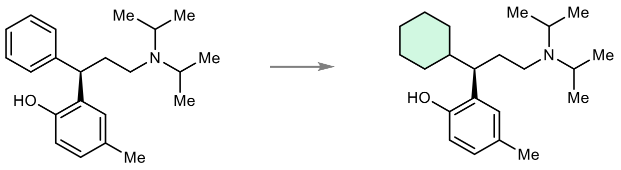

(*R*)-Tolterodine **29** (96% yield)

According to the general procedure, a mixture of ***(R)*-tolterodine** (65.1 mg, 0.2 mmol, 1.0 equiv.), $B_2(OH)_4$ (80.6 mg, 0.9 mmol, 4.5 equiv.) and $[Rh(COD)OH]_2$ (2.3 mg, 2.5 mol%, 0.005 mmol) in EtOH (1.0 mL, 0.2 M) was stirred under argon atmosphere for 14 hours at 50 °C to afford **29** (63.4 mg, 96% yield) as colorless oil.

^{1}H NMR (400 MHz, Methanol-d_4) δ 6.89 - 6.96 (m, 2H), 6.69 (d, J = 8.0 Hz, 1H), 3.65 - 3.54 (m, 2H), 2.94 (ddd, J = 13.8, 12.1, 5.6 Hz, 1H), 2.76 - 2.60 (m, 2H), 2.23 (s, 3H), 2.19 - 2.00 (m, 3H), 1.82 - 1.75 (m, 1H), 1.73 - 1.62 (m, 3H), 1.44 - 1.41 (m, 1H), 1.32 - 1.22 (m, 13H), 1.18 - 1.12 (m, 2H), 1.01 (qd, J = 12.4, 3.6 Hz, 1H), 0.90 - 0.79 (m, 1H).

^{13}C NMR (101 MHz, Methanol-d_4) δ 154.5, 130.5, 129.9, 129.2, 128.9, 116.3, 56.2, 42.7, 32.54, 32.50, 31.2, 27.7, 27.5, 27.4, 20.7, 17.9.

HRMS (ESI, m/z) calcd for $C_{22}H_{38}NO^+$ $[M+H]^+$: 332.2948, found: 332.2946.

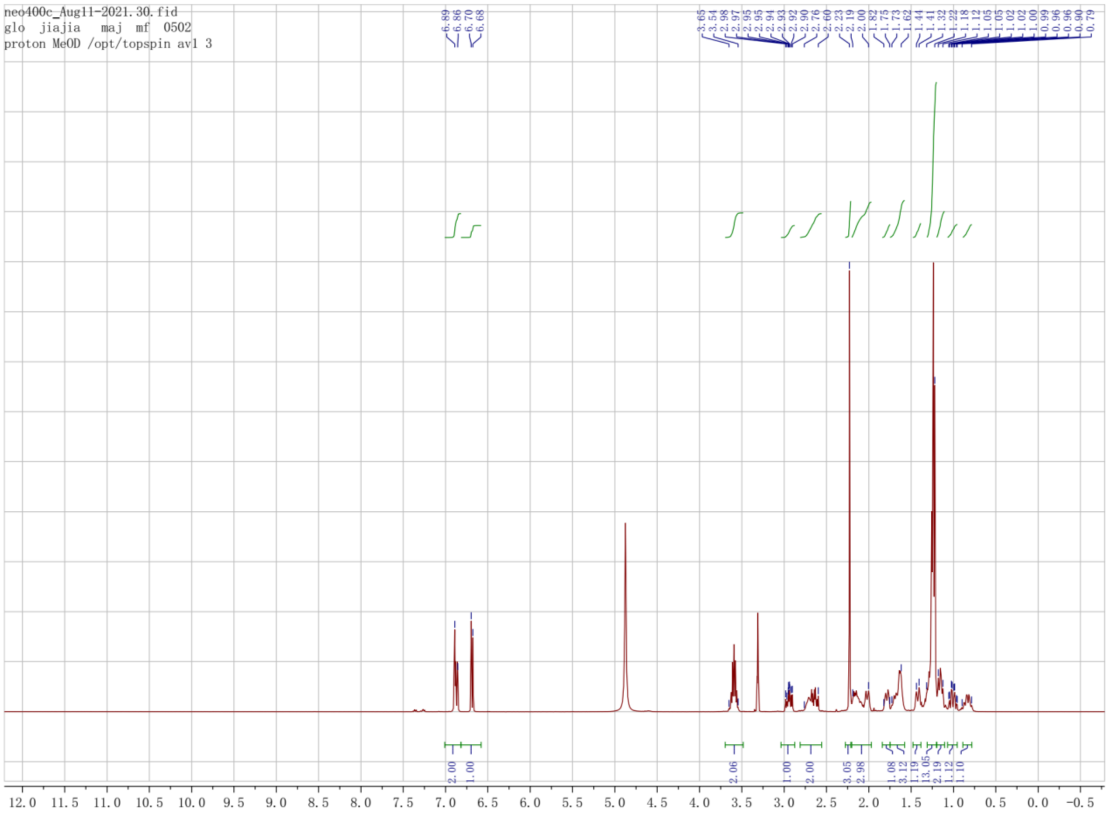
neo400c_Aug11-2021.30.fid
glo jiajia maj mf 0502
proton MeOD /opt/topspin av1 3
6.89 6.86 6.70 6.68
3.65 3.54 2.98 2.97 2.95 2.95 2.94 2.93 2.92 2.90 2.76 2.60 2.23 2.19 2.00 1.82 1.75 1.73 1.62 1.44 1.41 1.32 1.22 1.18 1.12 1.05 1.05 1.02 1.02 1.00 0.99 0.96 0.96 0.90 0.79
2.00 1.00 2.06 1.00 2.00 3.05 2.98 1.08 3.12 1.19 13.05 2.19 1.12 1.10
12.0 11.5 11.0 10.5 10.0 9.5 9.0 8.5 8.0 7.5 7.0 6.5 6.0 5.5 5.0 4.5 4.0 3.5 3.0 2.5 2.0 1.5 1.0 0.5 0.0 -0.5

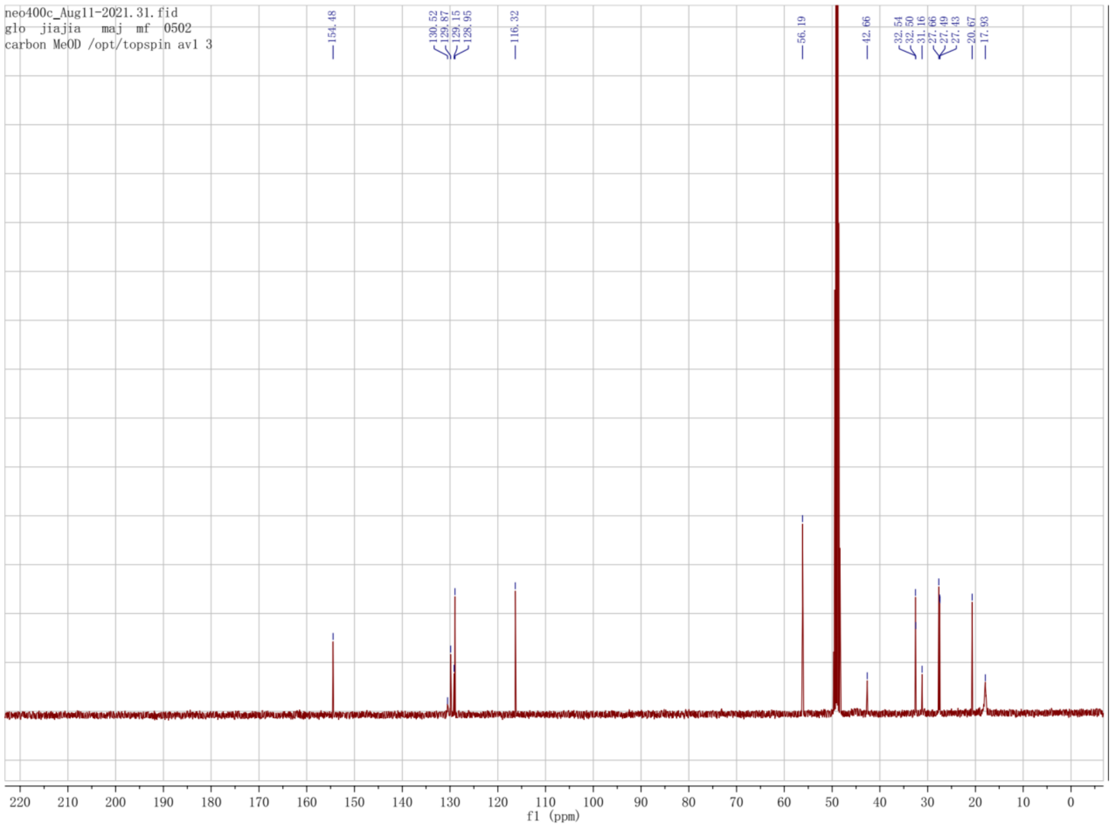
neo400c_Aug11-2021.31.fid
glo jiajia maj mf 0502
carbon MeOD /opt/topspin av1 3
154.48
130.52 129.87 129.15 128.95
116.32
56.19
42.66
32.54 32.50 31.16 27.66 27.49 27.43
20.67 17.93
220 210 200 190 180 170 160 150 140 130 120 110 100 90 80 70 60 50 40 30 20 10 0
f1 (ppm)

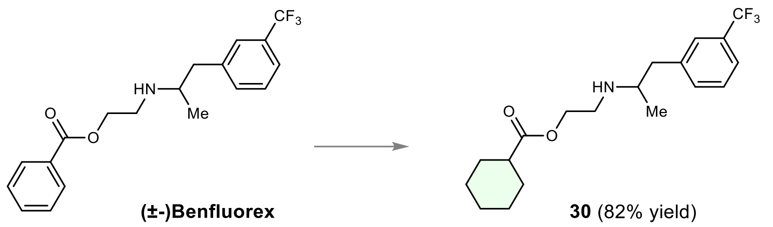

According to the general procedure, a mixture of (±)-**benfluorex** (70.3 mg, 0.20 mmol, 1.0 equiv.), $B_2(OH)_4$ (107.5 mg, 1.20 mmol, 6.0 equiv.) and $[Rh(COD)OH]_2$ (3.7 mg, 4.0 mol%, 0.008 mmol) in i-PrOH/EtOH (*v*/*v* = 9:1, 1.0 mL) was stirred under nitrogen atmosphere for 24 hours at 50 °C to afford **30** as a colorless oil (58.6 mg, 82% yield).

Purification conditions: CH_2Cl_2/MeOH = 50 to 30:1.

R_f= 0.4 in CH_2Cl_2/MeOH = 20 :1

^{1}H NMR (400 MHz, $CDCl_3$) δ 7.47 (d, *J* = 7.6 Hz, 1H), 7.44 (s, 1H), 7.42 - 7.35 (m, 2H), 4.19 - 4.12 (m, 1H), 4.11 - 4.04 (m, 1H), 2.99 - 2.86 (m, 2H), 2.82 - 2.78 (m, 1H) 2.77 - 2.73 (m, 1H), 2.69 (dd, *J* = 13.2, 6.4 Hz, 1H), 2.17 (tt, *J* = 11.2, 3.6 Hz, 1H), 1.80 - 1.65 (m, 4H), 1.65 - 1.57 (m, 1H), 1.37 - 1.13 (m, 6H), 1.07 (d, *J* = 6.4 Hz, 3H).

^{13}C NMR (100 MHz, $CDCl_3$) δ 176.0, 140.3, 132.8, 132.8, 130.83 (q, *J* = 32.0 Hz), 128.9, 126.0 (q, *J* = 3.7 Hz), 124.2 (q, *J* = 271.0 Hz), 123.23 (q, *J* = 3.9 Hz), 63.7, 54.1, 45.7, 43.5, 43.1, 29.0, 29.0, 25.8, 25.5, 25.5, 20.2.

^{19}F NMR (376 MHz, $CDCl_3$) δ -62.6.

HRMS (ESI, *m*/*z*) calcd for Chemical Formula: $C_{19}H_{27}F_3NO_2^+$ $[M+H]^+$:358.1988, found: 358.1993.

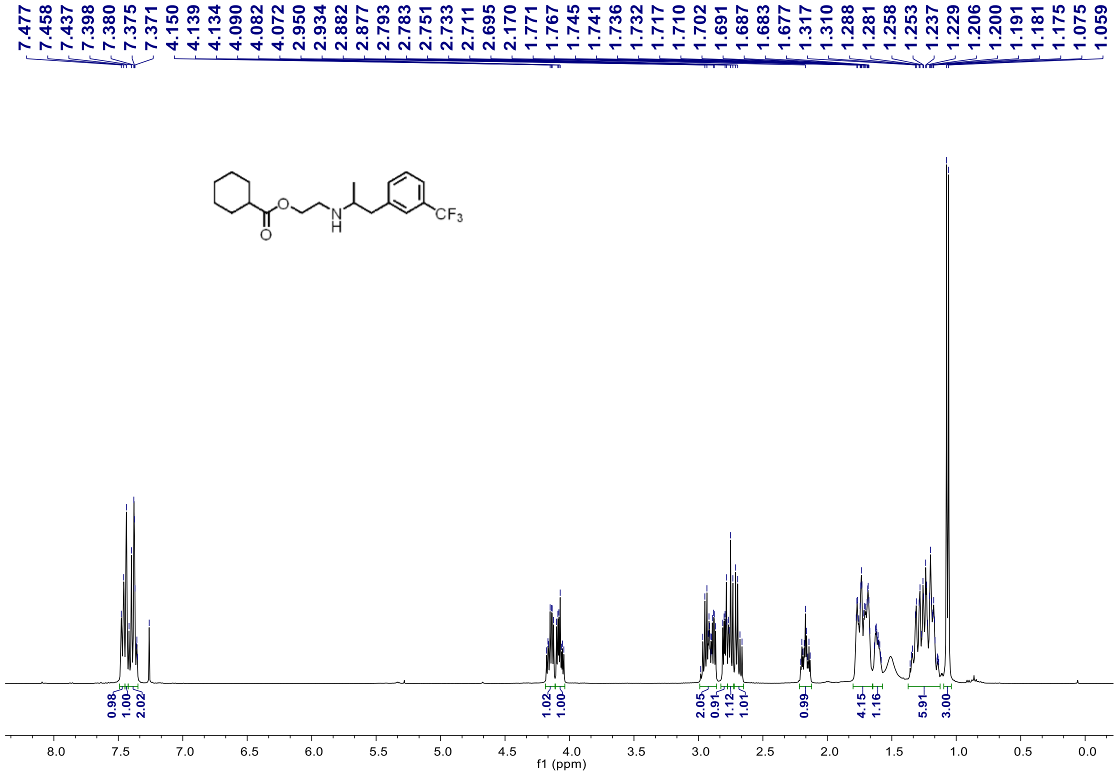

7.477
7.458
7.437
7.398
7.380
7.375
7.371
4.150
4.139
4.134
4.090
4.082
4.072
2.950
2.934
2.882
2.877
2.793
2.783
2.751
2.733
2.711
2.695
2.170
1.771
1.767
1.745
1.741
1.736
1.732
1.717
1.710
1.702
1.691
1.687
1.683
1.677
1.317
1.310
1.288
1.281
1.258
1.253
1.237
1.229
1.206
1.200
1.191
1.181
1.175
1.075
1.059
f1 (ppm)

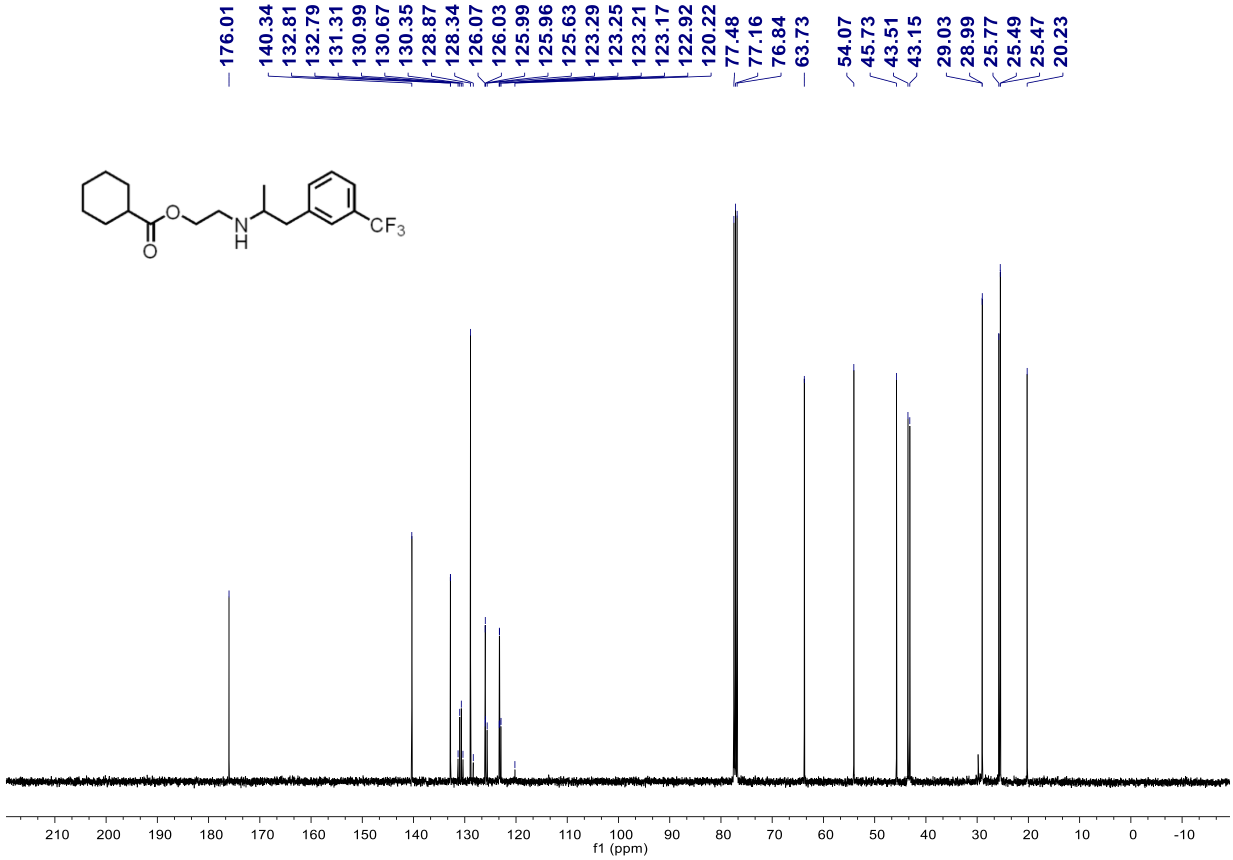

176.01
140.34
132.81
132.79
131.31
130.99
130.67
130.35
128.87
128.34
126.07
126.03
125.99
125.96
125.63
123.29
123.25
123.21
123.17
122.92
120.22
77.48
77.16
76.84
63.73
54.07
45.73
43.51
43.15
29.03
28.99
25.77
25.49
25.47
20.23
f1 (ppm)

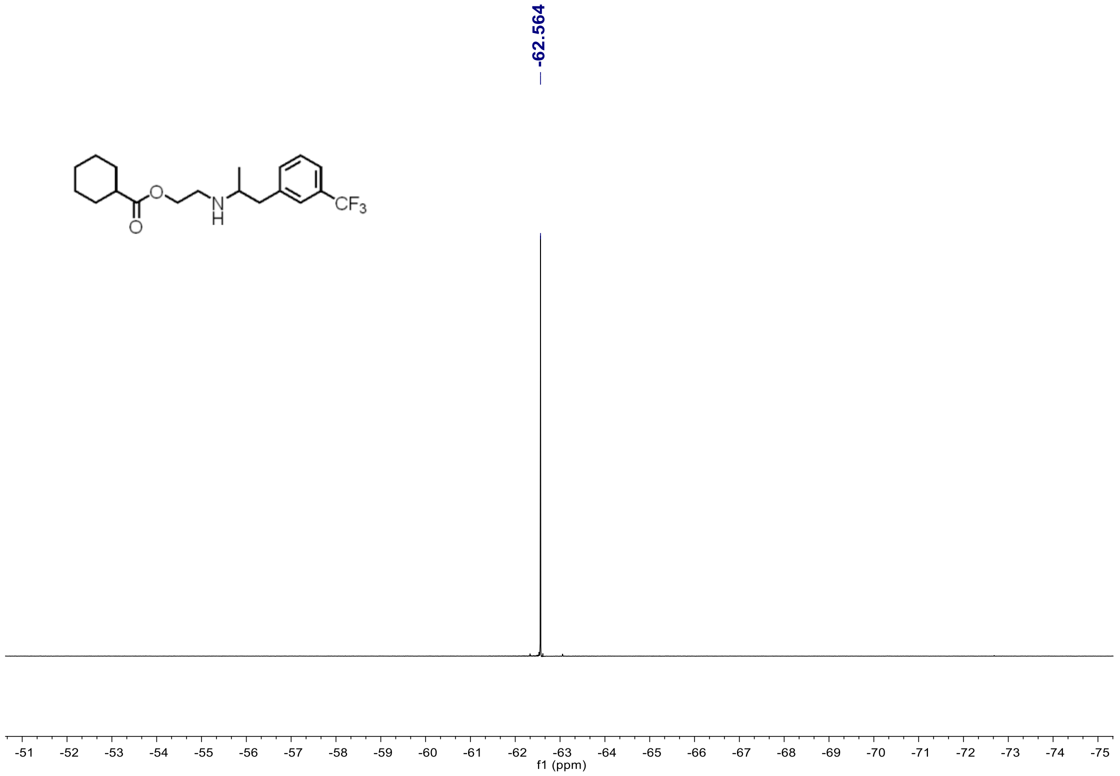

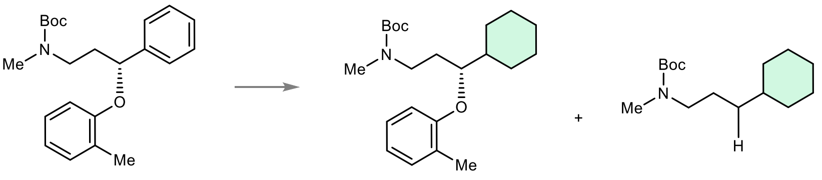

Boc-(*R*)-Atomoxetine **31** (32% yield) **31'** (58% yield)

According to the general procedure, a mixture of **Boc-(*R*)-atomoxetine** (71.1 mg, 0.2 mmol, 1.0 equiv.), $B_2(OH)_4$ (164.6 mg, 1.8 mmol, 9.0 equiv.) and $[Rh(COD)OH]_2$ (2.3 mg, 2.5 mol%, 0.005 mmol) in EtOH (1.0 mL, 0.2 M) was stirred under argon atmosphere for 14 hours at 50 °C to afford **31** (23.4 mg, 32% yield) and **31´**(29.6 mg, 58% yield) as colorless oil.

Analytical data of **31**:

^{1}H NMR (600 MHz, $CDCl_3$) δ 7.13-7.09 (m, 2H), 6.80 (t, J = 7.4 Hz, 1H), 6.76 (d, J = 8.2 Hz, 1H), 4.13 (m, 1H), 3.34-3.23 (m, 2H), 2.79 (s, 3H), 2.22 (s, 3H), 1.88-1.85 (m, 2H), 1,80-1.66 (m, 6H), 1.41 (s, 9H), 1.26-1.19 (m, 2H), 1.17-1.11 (m, 3H).

^{13}C NMR (151 MHz, $CDCl_3$) δ 156.4, 155.7, 130.8, 127.3, 126.6, 119.8, 111.6, 79.3, 78.6, 45.9, 40.5, 34.3, 28.6, 28.4, 26.6, 26.3, 26.2, 16.5.

HRMS (ESI, *m*/*z*) calcd for $C_{22}H_{35}NO_3Na^+$ $[M+ Na]^+$: 384.2511, found: 384.2509.

Analytical data of **31´**:

^{1}H NMR (600 MHz, $CDCl_3$) δ 3.15 (m, 2H), 2.82 (s, 3H), 1.72-1.64 (m, 5H), 1.52-1.46 (m, 2H), 1.44

(s, 9H), 1.24-1.17 (m, 3H), 1.16-1.11 (m, 3H), 0.93-0.82 (m, 2H).

^{13}C NMR (151 MHz, $CDCl_3$) δ 156.0, 79.2, 37.4, 34.5, 34.2, 33.5, 28.64, 28.62, 26.8, 26.5, 25.2.

HRMS (ESI, *m/z*) calcd for $C_{15}H_{29}NO_2Na^+$ $[M+ Na]^+$: 278.1931, found: 278.2123.

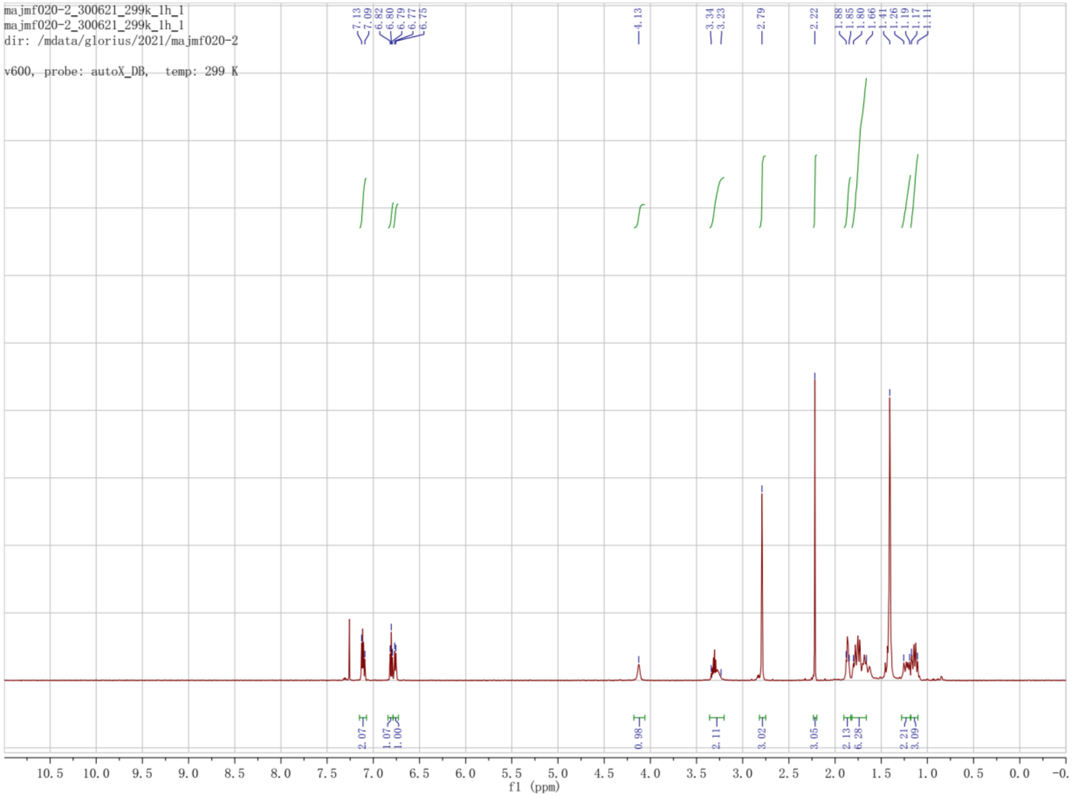

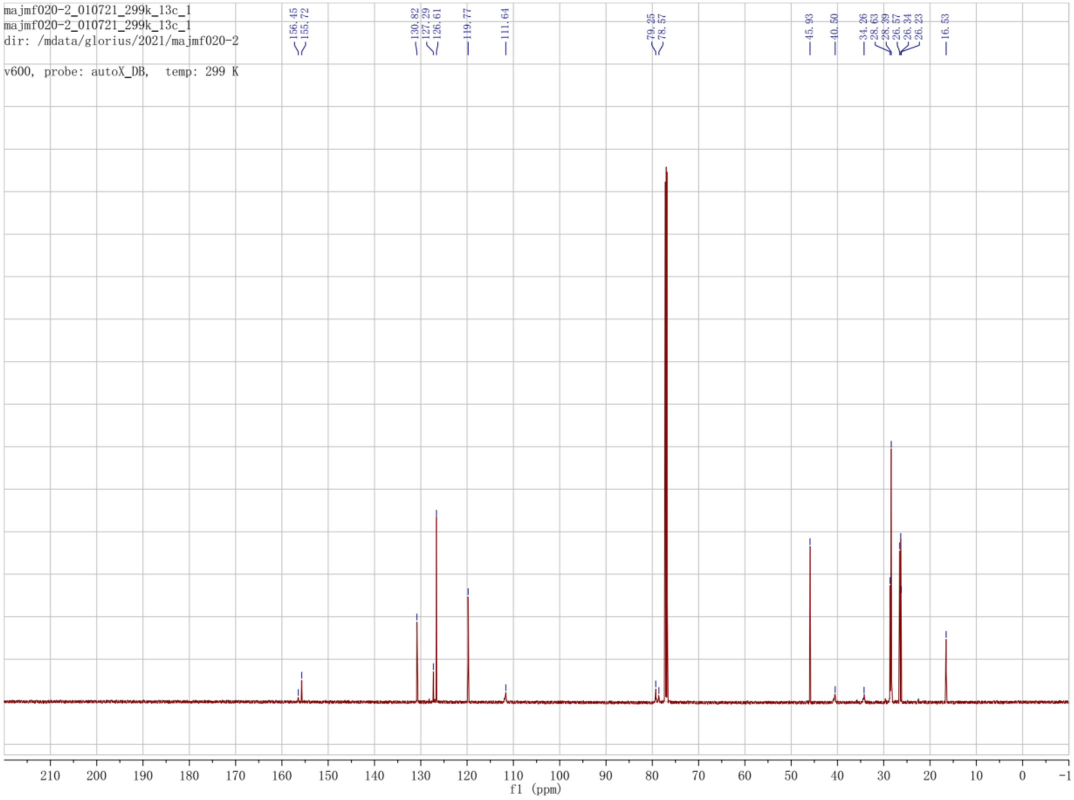

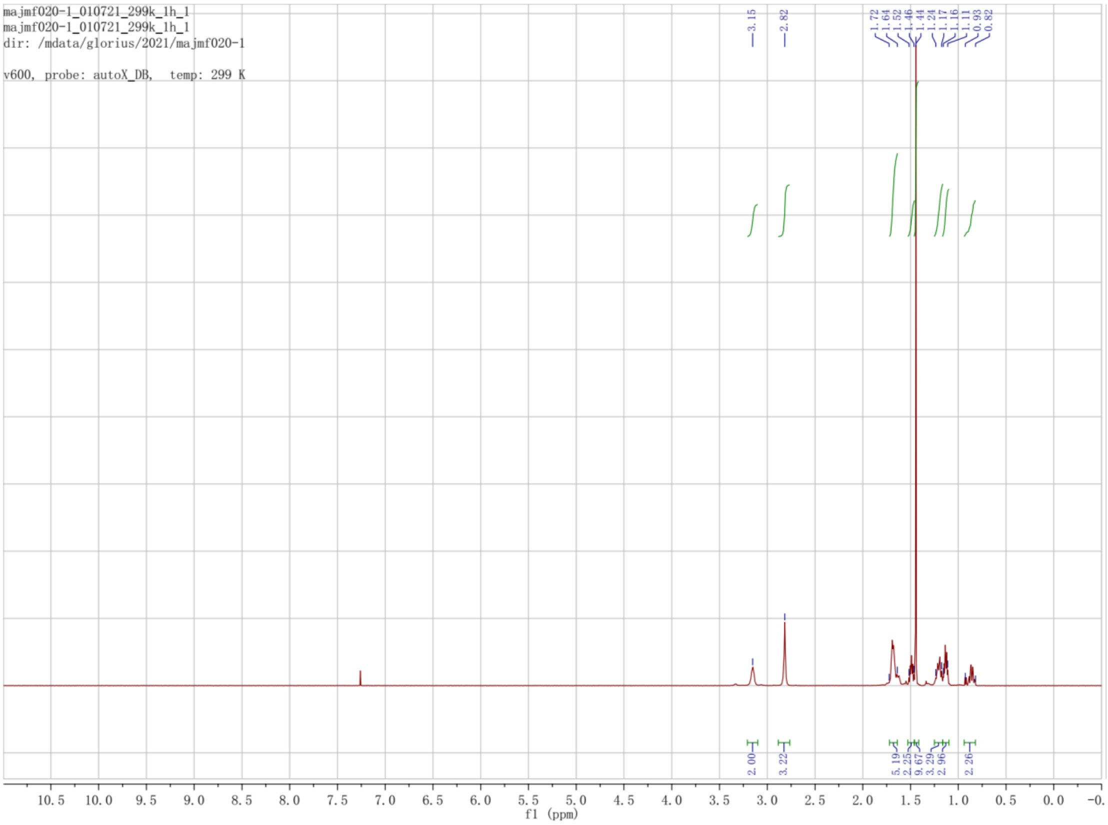
majmf020-1_010721_299k_1h_1
majmf020-1_010721_299k_1h_1
dir: /mdata/glorius/2021/majmf020-1
v600, probe: autoX_DB, temp: 299 K
3.15
2.82
1.72
1.64
1.52
1.46
1.44
1.24
1.17
1.16
1.11
0.93
0.82
2.00
3.22
5.19
2.25
9.67
3.29
2.96
2.26
f1 (ppm)

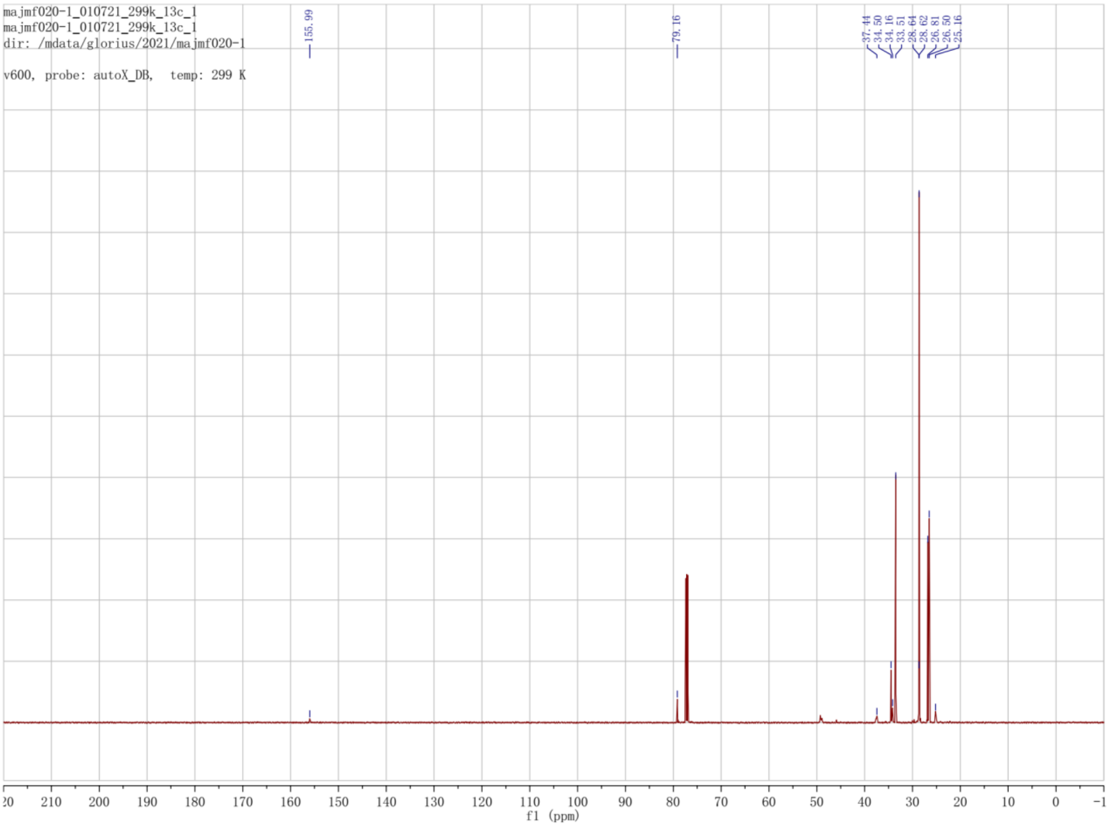
majmf020-1_010721_299k_13c_1
majmf020-1_010721_299k_13c_1
dir: /mdata/glorius/2021/majmf020-1
v600, probe: autoX_DB, temp: 299 K
155.99
79.16
37.44
34.50
34.16
33.51
28.64
28.62
26.81
26.50
25.16
f1 (ppm)

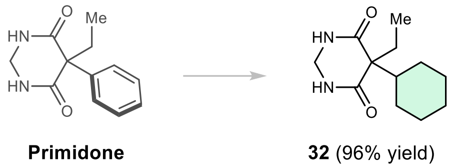

According to the general procedure, a mixture of **primidone** (43.6 mg, 0.2 mmol, 1.0 equiv.), $B_2(OH)_4$ (80.6 mg, 0.9 mmol, 4.5 equiv.) and $[Rh(COD)OH]_2$ (2.3 mg, 2.5 mol%, 0.005 mmol) in EtOH (1.0 mL, 0.2 M) was stirred under argon atmosphere for 14 hours at 50 °C to afford **32** as a colorless oil (43.1 mg, 96% yield).

^{1}H NMR (600 MHz, Methanol-*d*$_4$) δ 4.64 (d, *J* = 10.9 Hz, 1H), 4.44 (d, *J* = 10.9 Hz, 1H), 1.91 (q, *J* = 7.6, 7.2 Hz, 2H), 1.86 (m, 1H), 1.81 - 1.77 (m, 4H), 1.65 (d, *J* = 12.6 Hz, 1H), 1.27 - 1.19 (m, 4H), 1.17 - 1.11 (m, 1H), 0.79 (t, *J* = 7.1 Hz, 3H).

^{13}C NMR (151 MHz, Methanol-*d*$_4$) δ 174.1, 60.6, 51.9, 47.0, 29.8, 27.9, 27.4, 26.3, 10.0.

HRMS (ESI, *m*/*z*) calcd for $C_{12}H_{21}N_2O_2^+$ [M+H]$^+$: 225.1597, found: 225.1598.

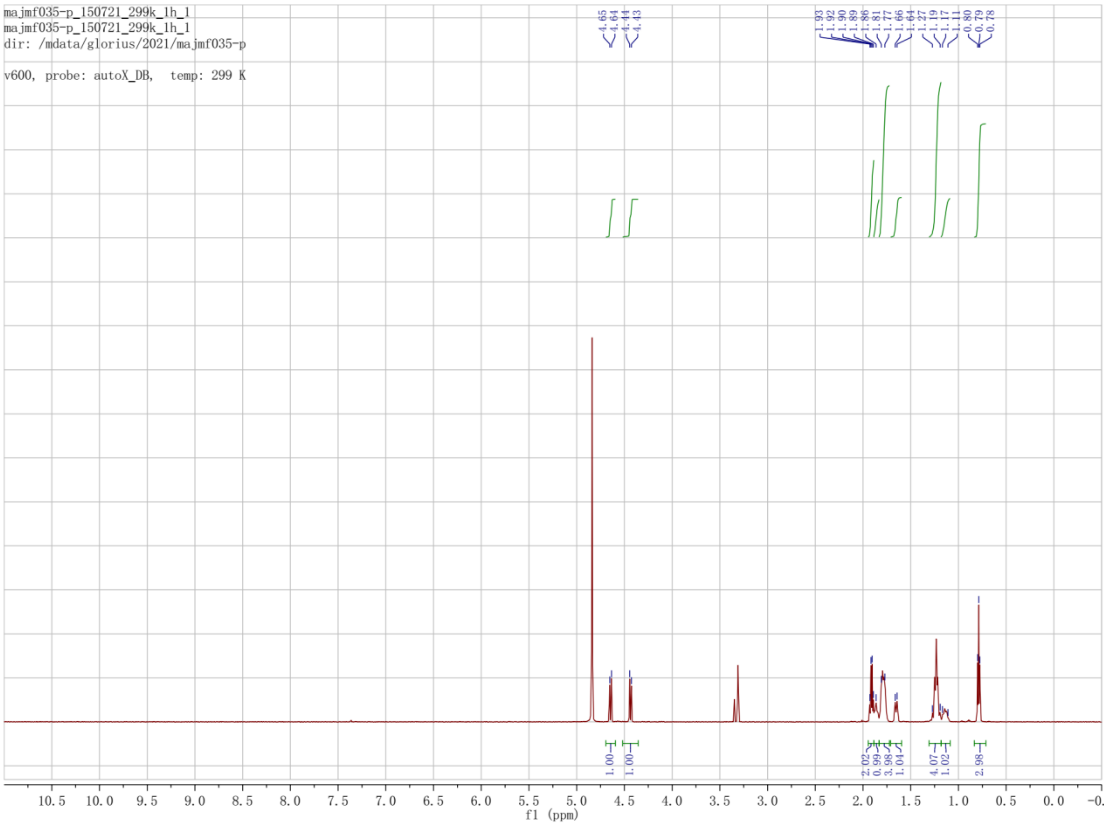

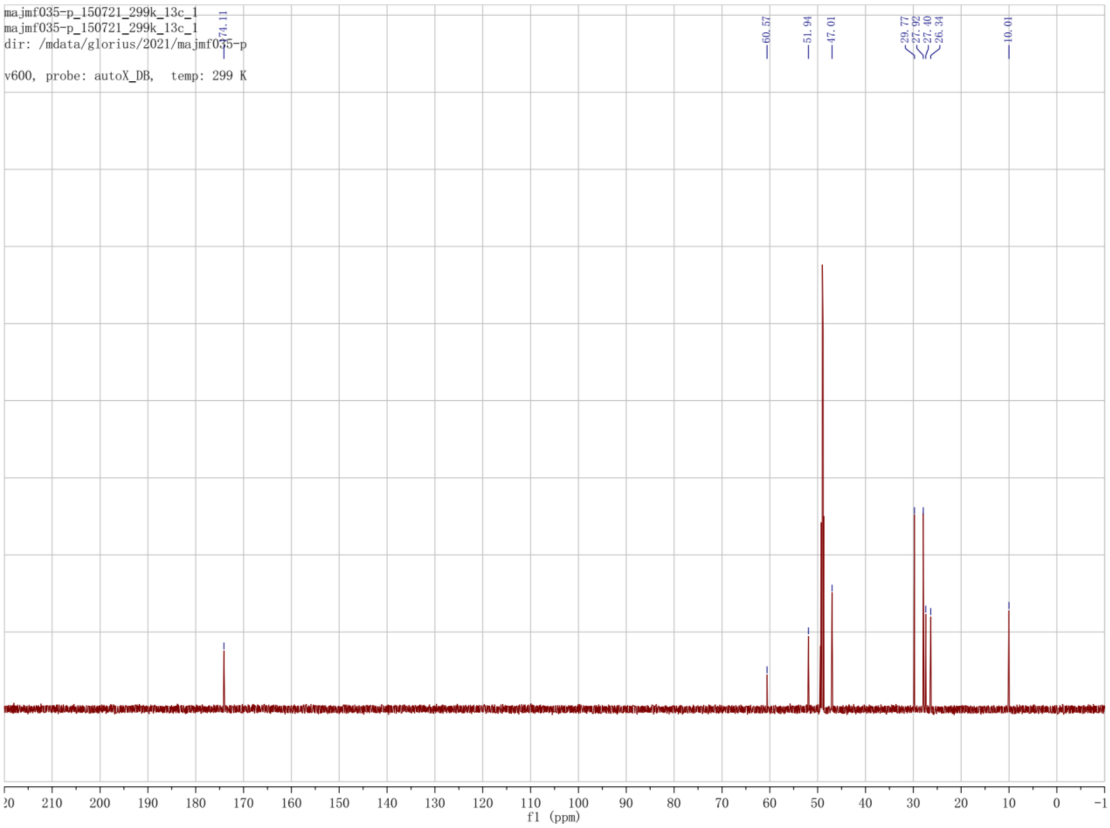

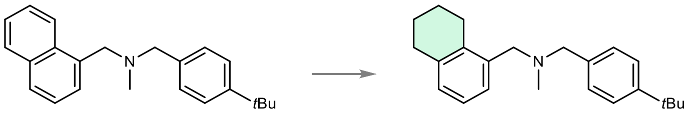

According to the general procedure, a mixture of **butenafine** (63.4 mg, 0.20 mmol, 1.0 equiv.), $B_2(OH)_4$ (80.6 mg, 0.90 mmol, 4.5 equiv.) and $[Rh(COD)OH]_2$ (2.4 mg, 2.5 mol%, 0.001 mmol) in EtOH (1.0 mL, 0.2 M) was stirred under nitrogen atmosphere for 16 hours at 50 °C to afford **33** as a yellow oil (8.6 mg, 13% yield).

Purification conditions: CH_2Cl_2/MeOH = 200:1 to 80:1.

Rf = 0.5 in CH_2Cl_2/MeOH) = 50:1.

^{1}H NMR (400 MHz, $CDCl_3$) δ 7.33 (d, J = 8.4 Hz, 2H), 7.26 (d, J = 8.0 Hz, 2H), 7.18 (d, J = 7.2 Hz, 1H), 7.06 (t, J = 7.6 Hz, 1H), 6.98 (d, J = 7.6 Hz, 1H), 3.47 (d, J = 14.0 Hz, 4H), 2.79 (t, J = 6.0 Hz, 4H), 2.15 (s, 3H), 1.84 - 1.74 (m, 4H), 1.31 (s, 9H).

^{13}C NMR (100 MHz, $CDCl_3$) δ 150.0, 137.5, 136.6, 128.9, 128.4, 127.5, 125.9, 125.2, 125.0, 61.9, 59.8, 42.2, 34.6, 31.5, 30.3, 26.0, 23.4, 23.0.

HRMS (ESI, m/z) calcd for $C_{23}H_{32}N^+$ $[M+H]^+$: 322.2535, found: 322.2537.

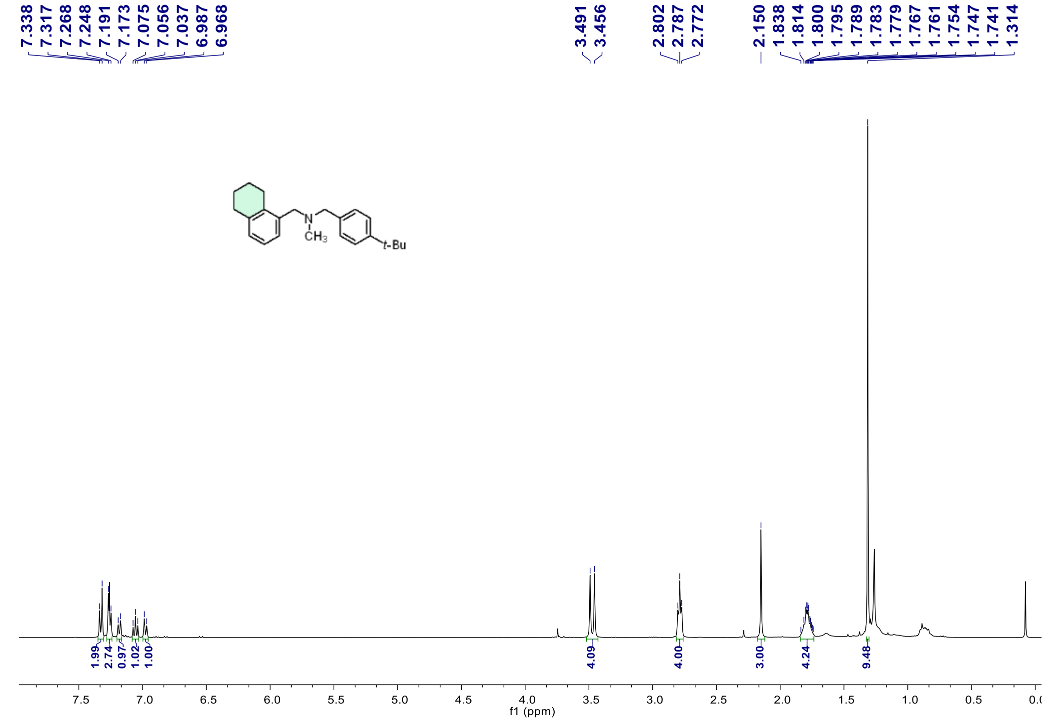
7.338 7.317 7.268 7.248 7.191 7.173 7.075 7.056 7.037 6.987 6.968
3.491 3.456
2.802 2.787 2.772
2.150 1.838 1.814 1.800 1.795 1.789 1.783 1.779 1.767 1.761 1.754 1.747 1.741 1.314
N
CH3
t-Bu
1.99 2.74 0.97 1.02 1.00
4.09
4.00
3.00
4.24
9.48
7.5 7.0 6.5 6.0 5.5 5.0 4.5 4.0 3.5 3.0 2.5 2.0 1.5 1.0 0.5
f1 (ppm)

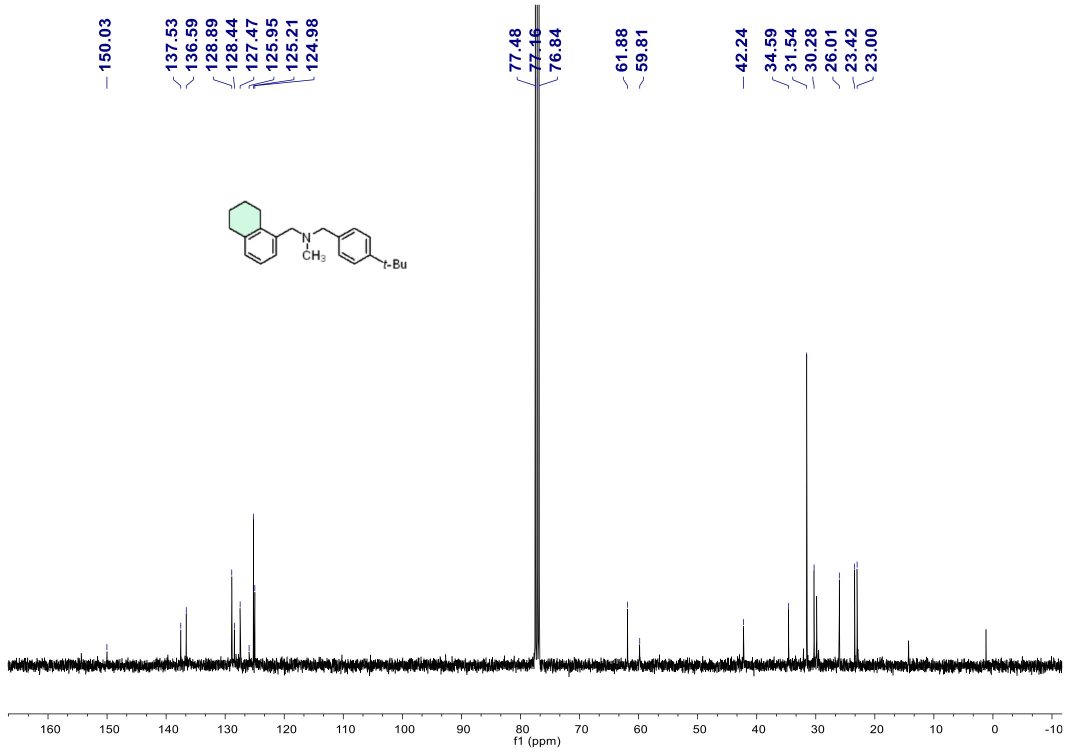
150.03
137.53 136.59 128.89 128.44 127.47 125.95 125.21 124.98
77.48 77.16 76.84
61.88 59.81
42.24
34.59 31.54 30.28 26.01 23.42 23.00
N
CH3
t-Bu
160 150 140 130 120 110 100 90 80 70 60 50 40 30 20 10 0 -10
f1 (ppm)

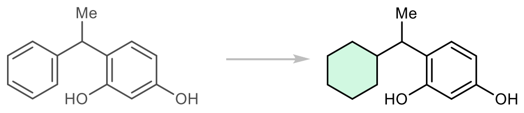

(±)-Phenylethyl resorcinol **34** (78% yield)

According to the general procedure, a mixture of **(±)-phenylethyl resorcinol** (42.8 mg, 0.2 mmol, 1.0 equiv.), $B_2(OH)_4$ (143.3 mg, 1.6 mmol, 8.0 equiv.) and $[Rh(COD)OH]_2$ (3.6 mg, 4 mol%, 0.0080 mmol) in EtOH (1.0 mL, 0.2 M) was stirred under nitrogen atmosphere for 14 hours at 50 °C to afford **34** as a colorless oil (34.5 mg, 78% yield).

^{1}H NMR (400 MHz, $CDCl_3$) δ 6.94 (d, J = 8.3 Hz, 1H), 6.39 (dd, J = 8.4, 2.4 Hz, 1H), 6.30 (d, J = 2.5 Hz, 1H), 5.22 (br, 1H), 5.04 (br, 1H), 2.69 (p, J = 7.2 Hz, 1H), 2.09 - 1.85 (m, 2H), 1.76 - 1.72 (m, 1H), 1.68 - 1.56 (m, 2H), 1.52 - 1.49 (m, 1H), 1.45 - 1.36 (m, 1H), 1.18 (d, J = 7.0 Hz, 3H), 1.15 - 1.10 (m, 2H), 0.99 - 0.85 (m, 2H).

^{13}C NMR (101 MHz, $CDCl_3$) δ 154.1, 154.0, 128.9, 125.4, 107.9, 102.9, 43.6, 37.7, 31.5, 30.6, 29.8, 26.70, 26.67, 18.1.

HRMS (ESI, m/z) calcd for $C_{14}H_{20}O_2Na^+$ $[M+Na]^+$: 243.1361, found: 243.1355.

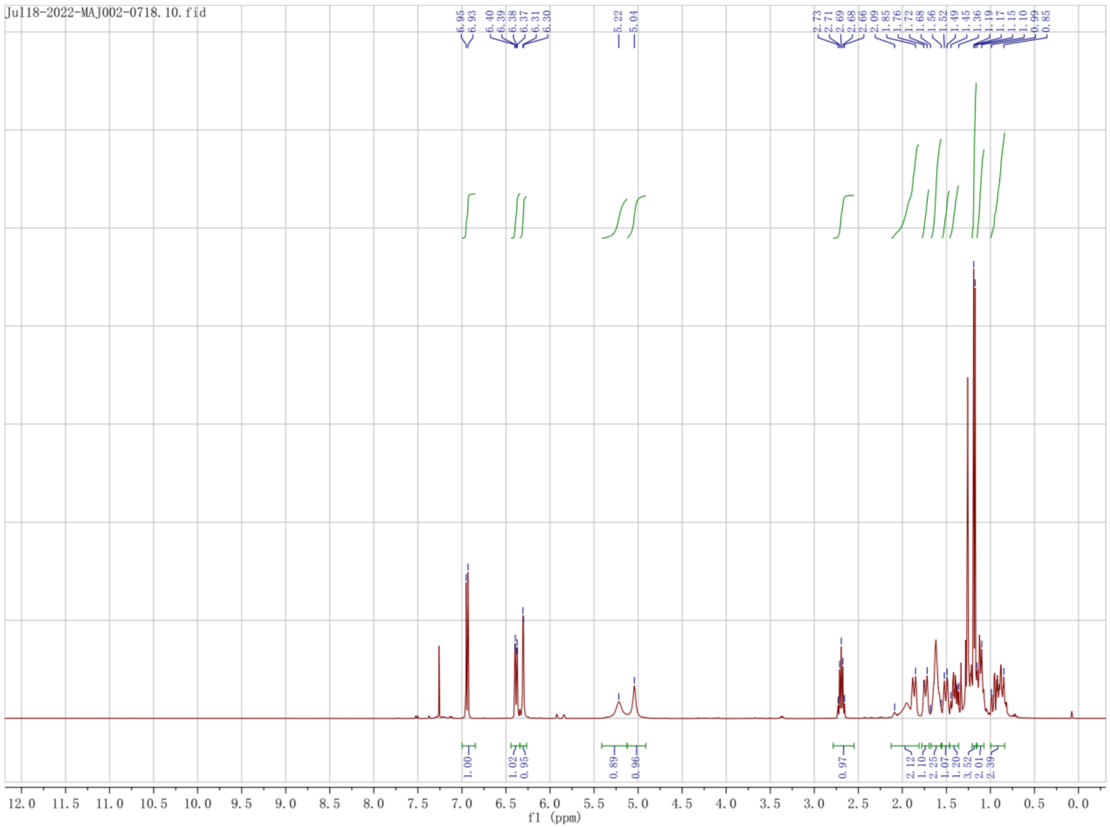

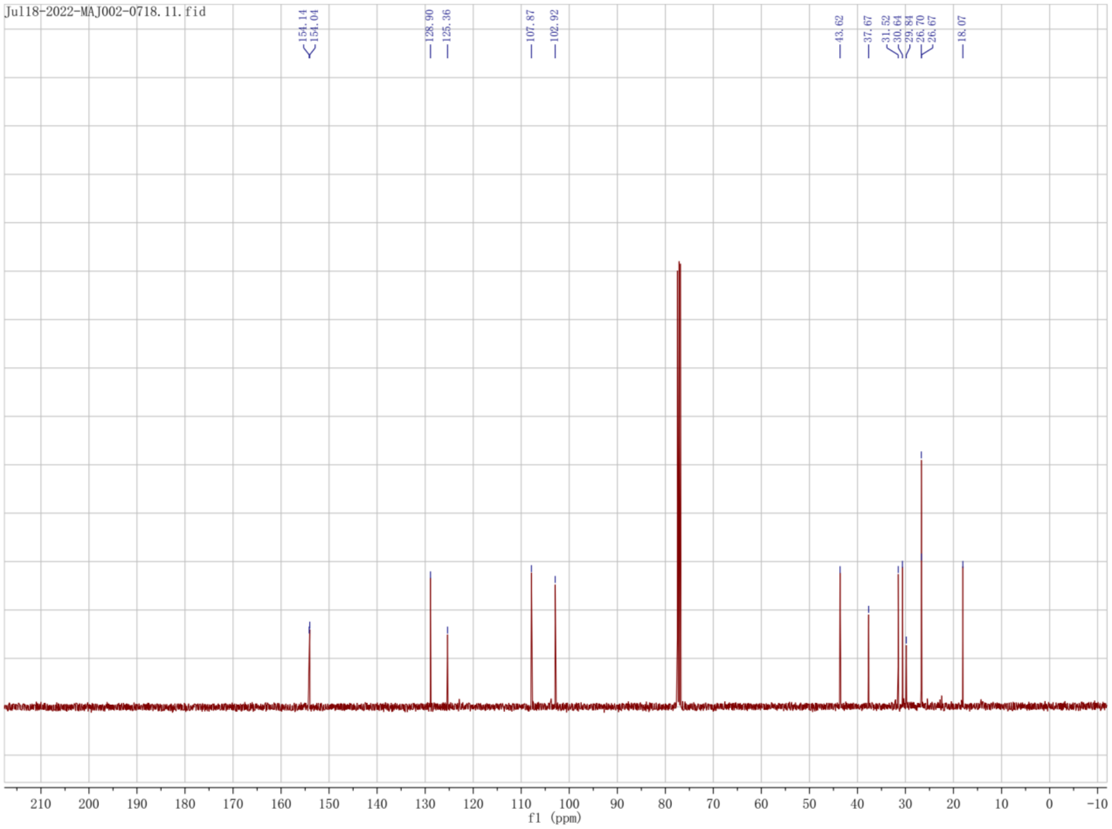

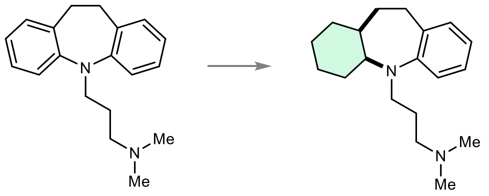

According to the general procedure, a mixture of **imipramine** (56.0 mg, 0.2 mmol, 1.0 equiv.), $B_2(OH)_4$ (80.6 mg, 0.9 mmol, 4.5 equiv.) and $[Rh(COD)OH]_2$ (4.6 mg, 5 mol%, 0.010 mmol) in EtOH (1.0 mL, 0.2 M) was stirred under argon atmosphere for 14 hours at 50 °C to afford **35** as a colorless oil (24.9 mg, 44% yield, >95:5 d.r.).

^{1}H NMR (400 MHz, $CDCl_3$) δ 7.13 - 7.07 (m, 2H), 6.92 - 6.85 (m, 2H), 3.47 - 3.37 (m, 1H), 3.27 - 3.12 (m, 2H), 2.98 - 2.85 (m, 2H), 2.75 - 2.56 (m, 8H), 2.14 - 1.96 (m, 3H), 1.68 - 1.51 (m, 5H), 1.36 - 1.09 (m, 4H), 0.78 (m, 1H).

^{13}C NMR (151 MHz, acetone-*d*$_6$, mixture of diastereomers) δ 148.6, 137.8, 129.8, 128.8, 126.5, 126.2, 122.7, 121.7, 120.7, 119.9, 62.1, 55.5, 50.0, 41.8, 33.8, 32.6, 31.9, 26.4, 26.2, 24.0, 23.5, 20.4.

HRMS (ESI, *m*/*z*) calcd for $C_{19}H_{31}N_2^+$ [M+H]$^+$: 287.2482, found: 287.2480.

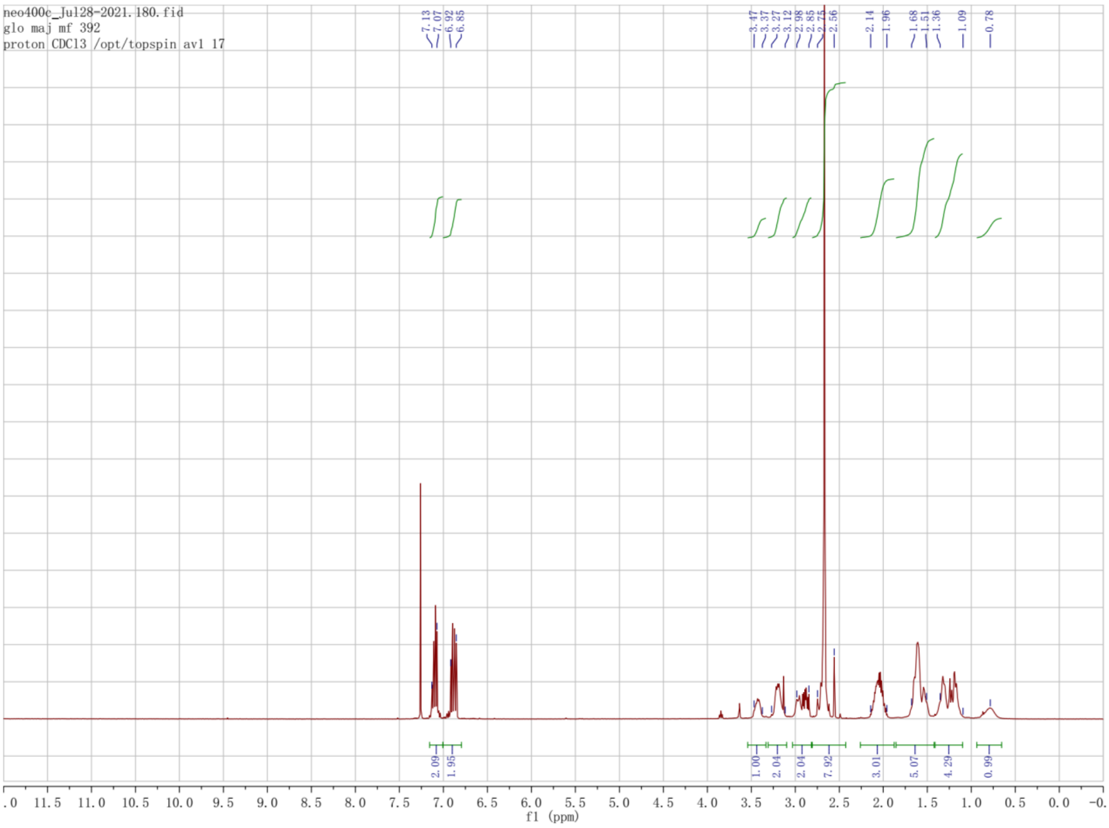
neo400c_Jul28-2021.180.fid
glo maj mf 392
proton CDCl3 /opt/topspin av1 17
7.13
7.07
6.92
6.85
3.47
3.37
3.27
3.12
2.98
2.85
2.75
2.56
2.14
1.96
1.68
1.51
1.36
1.09
0.78
2.09
1.95
1.00
2.04
2.04
7.92
3.01
5.07
4.29
0.99
f1 (ppm)

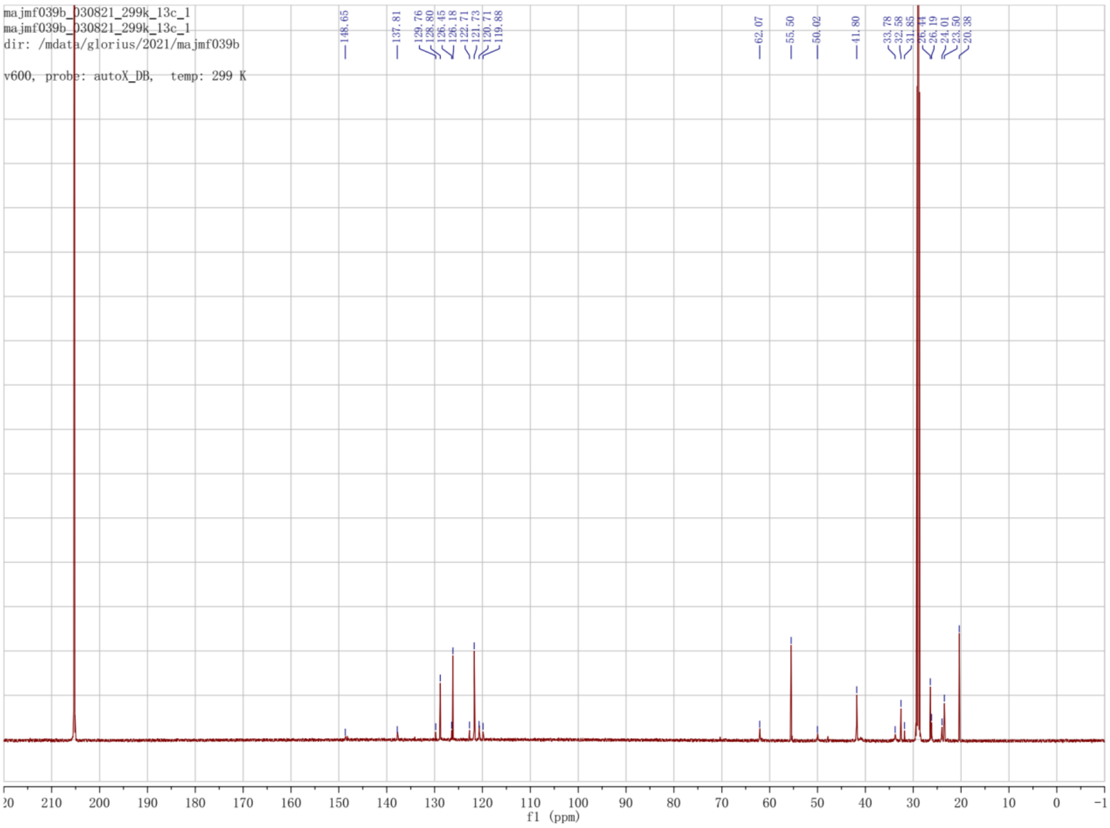
majmf039b_030821_299k_13c_1
majmf039b_030821_299k_13c_1
dir: /mdata/glorius/2021/majmf039b
v600, probe: autoX_DB, temp: 299 K
148.65
137.81
129.76
128.80
126.45
126.18
122.71
121.73
120.71
119.88
62.07
55.50
50.02
41.80
33.78
32.58
31.85
26.44
26.19
24.01
23.50
20.38
f1 (ppm)

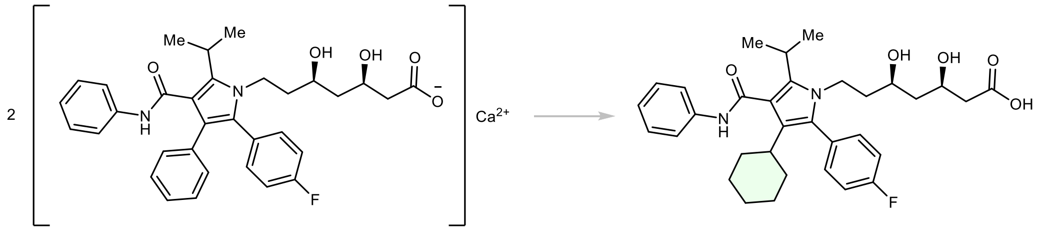

Atorvastatin Calcium Trihydrate **36** (56% yield)

According to the general procedure, a solution of Atorvastatin Calcium Trihydrate (120.9 mg, 0.1 mmol, 0.5 equiv.), $B_2(OH)_4$ (125.5 mg, 1.4 mmol, 7.0 equiv.) and $[Rh(COD)OH]_2$ (3.7 mg, 4.0 mol%, 0.005 mmol) in EtOH (1.0 mL, 0.2 M) was stirred under argon atmosphere for 24 hours at 50 °C to afford **36** (31.8 mg, 56% yield) as colorless oil.

Analytical data of **36**:

^{1}H NMR (500 MHz, $CDCl_3$) δ 7.18 - 7.05 (m, 7H), 6.98 (t, J = 8.6 Hz, 2H), 4.11 (dt, J = 14.3, 6.9 Hz, 2H), 3.96 (dt, J = 14.6, 7.2 Hz, 1H), 3.79 - 3.62 (m, 2H), 3.39 (p, J = 7.0 Hz, 1H), 2.45 (d, J = 3.2 Hz, 2H), 1.69 - 1.61 (m, 2H), 1.59 - 1.41 (m, 12H), 1.41 - 1.20 (m, 4H), 1.14 (d, J = 14.5 Hz, 1H), 1.00 (d, J = 10.8 Hz, 1H), 0.77 (t, J = 11.8 Hz, 2H).

^{13}C NMR (500 MHz, $CDCl_3$) δ 167.9, 165.9, 163.6, 138.8, 134.4, 132.8, 130.1, 128.8, 128.2, 127.9, 126.1, 121.9, 116.8, 115.6, 115.5, 69.6, 69.1, 49.0, 41.9, 41.3, 40.6, 37.1, 31.9, 26.9, 25.4, 24.5, 22.7, 22.2.

HRMS (ESI, *m/z*) calcd for $C_{33}H_{42}N_2O_5F^+$ $[M+H]^+$: 565.3078, found: 564.3965.

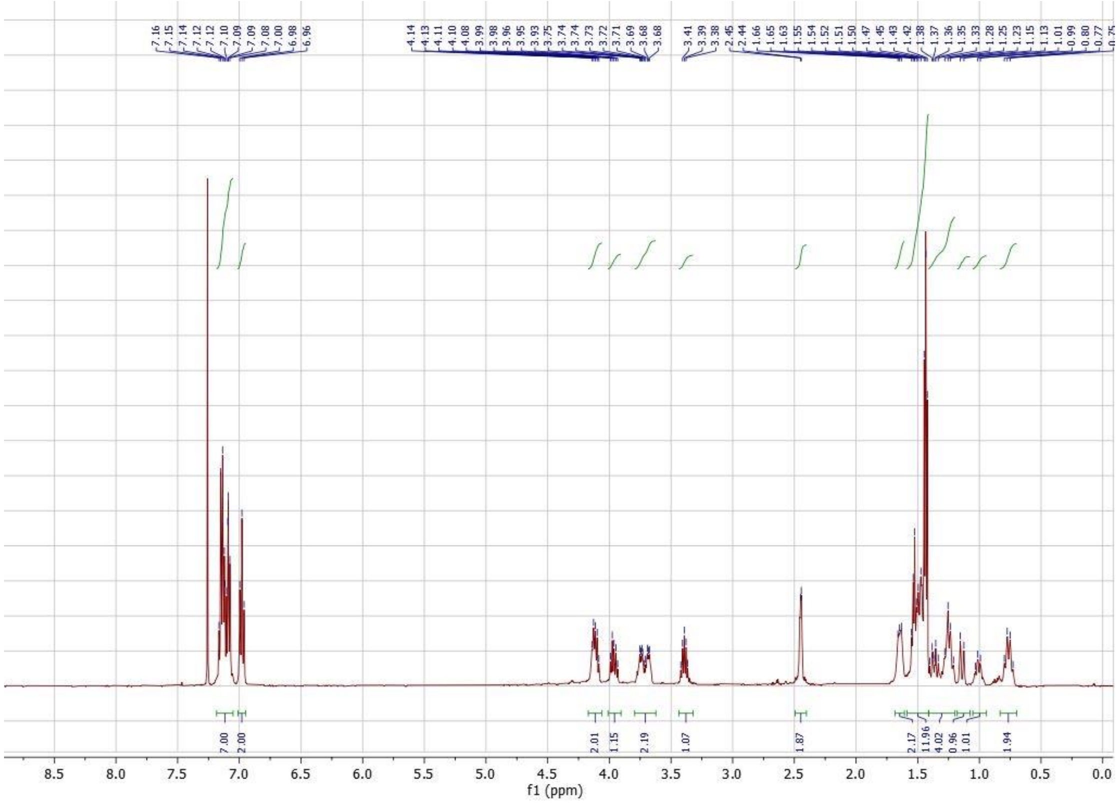

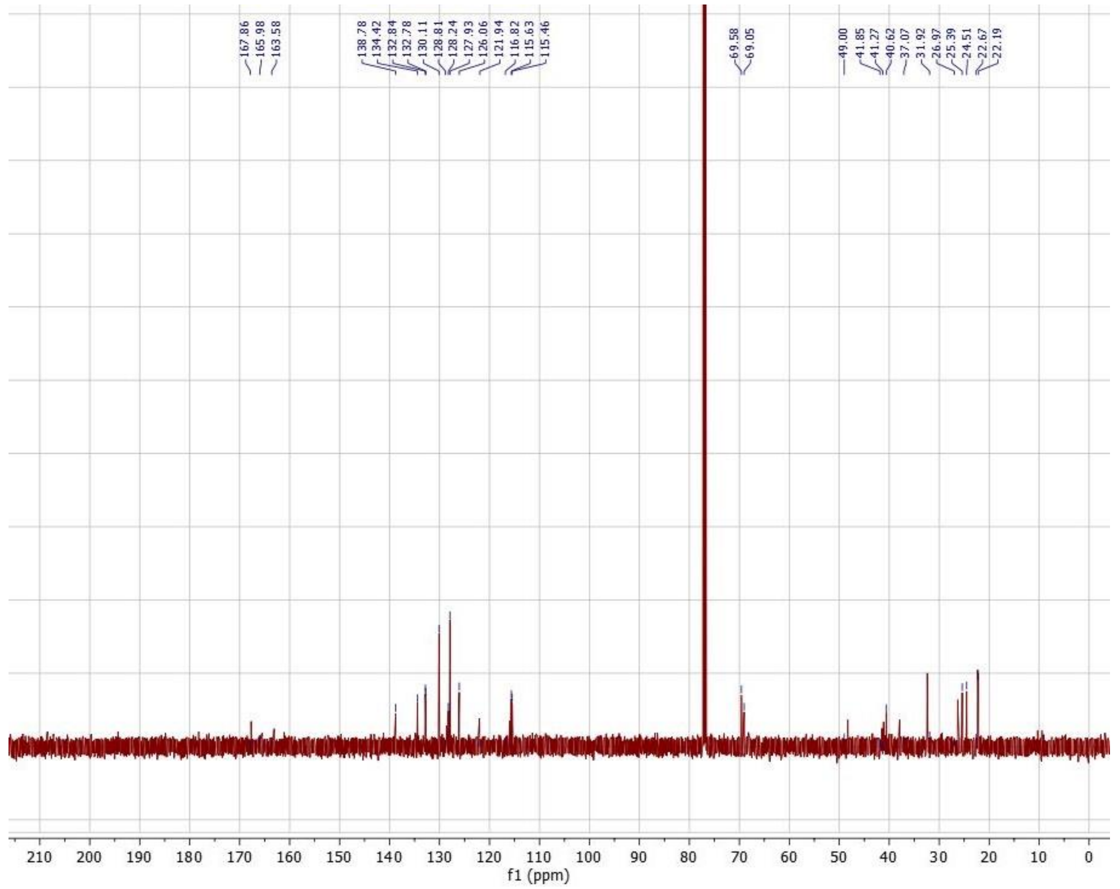

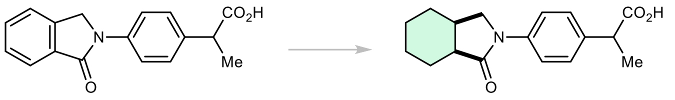

(±)-Indoprofen **37** (42% yield)

According to the general procedure, a mixture of **(±)-indoprofen** (56.9 mg, 0.20 mmol, 1.0 equiv.), $B_2(OH)_4$ (107.5 mg, 1.20 mmol, 6.0 equiv.) and $[Rh(COD)OH]_2$ (3.7 mg, 4.0 mol%, 0.008 mmol) in EtOH (3.0 mL, 0.07 M) was stirred under nitrogen atmosphere for 40 hours at 50 °C to afford **37** as a white oil (23.5 mg, 42% yield, >95:5 d.r.).

Purification conditions: petroleum ether/EtOAc = 30:1 to 5:1.

R_f= 0.3 in petroleum ether/EtOAc (1.5:1).

^{1}H NMR (400 MHz, $CDCl_3$) δ 7.59 (d, J = 8.4 Hz, 2H), 7.30 (d, J = 8.4 Hz, 2H), 3.81 (dd, J = 9.6, 6.0 Hz, 1H), 3.70 (q, J = 7.2 Hz, 1H), 3.36 (dd, J = 9.6, 2.0 Hz, 1H), 2.71 - 2.63 (m, 1H), 2.47 - 2.38 (m, 1H), 2.15 - 2.06 (m, 1H), 1.80 - 1.71 (m, 1H), 1.64 - 1.51 (m, 3H), 1.48 (d, J = 7.8 Hz, 3H), 1.33 - 1.23 (m, 3H).

^{13}C NMR (100 MHz, $CDCl_3$) δ 179.8, 175.5, 139.3, 135.9, 128.1, 119.9, 52.8, 44.9, 43.5, 32.2, 28.0, 23.7, 23.6, 22.9, 18.3.

HRMS (ESI, m/z) calcd for $C_{17}H_{22}NO_3^+$ $[M+H]^+$: 288.1600, found: 288.1596.

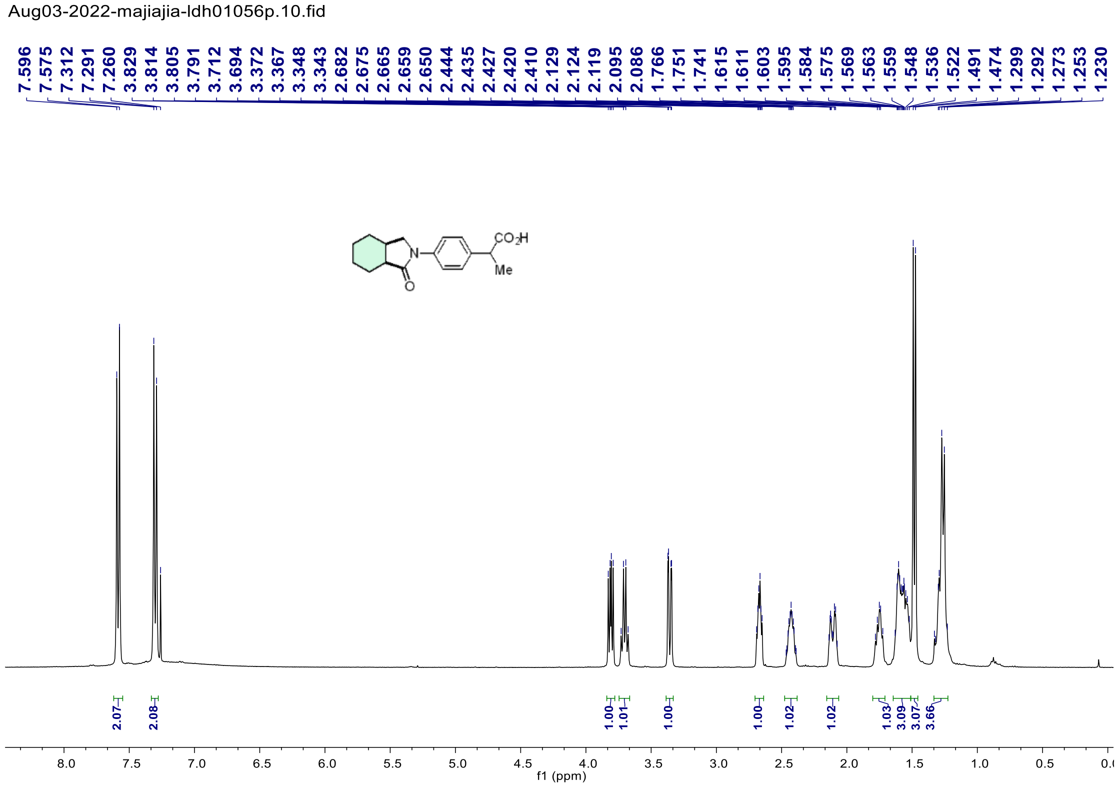

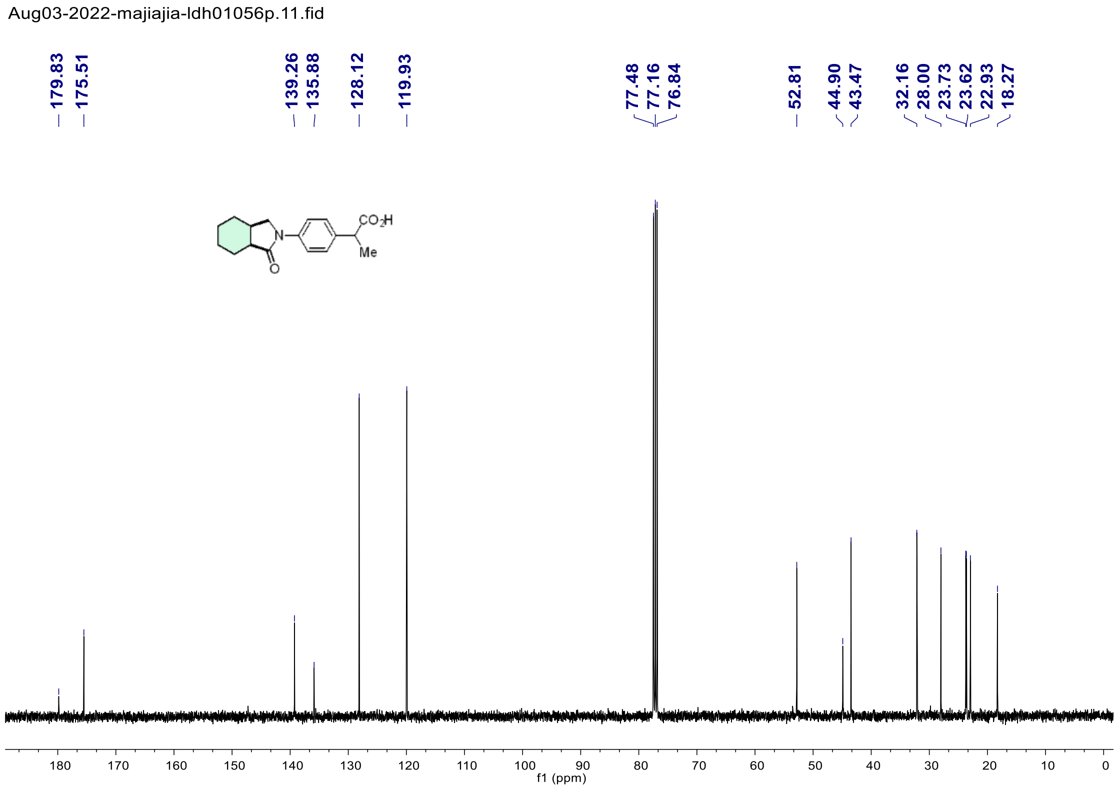

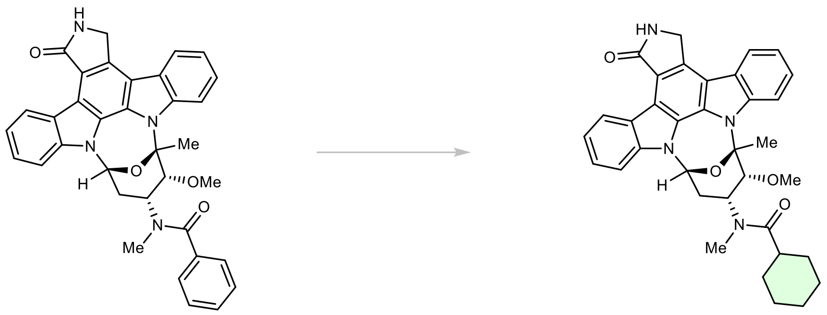

According to the general procedure, a mixture of **midostaurin** (114.0 mg, 0.20 mmol, 1.0 equiv.), $B_2(OH)_4$ (107.5 mg, 1.2 mmol, 6.0 equiv.) and $[Rh(COD)OH]_2$ (3.7 mg, 4.0 mol%, 0.008 mmol) in EtOH (1.0 mL, 0.2 M) was stirred under nitrogen atmosphere for 48 hours at 80 °C to afford **38** as a white solid (63.3 mg, 55% yield)

Purification conditions: petroleum ether/Acetone = 10:1 to 4:1.

R_f= 0.3 in petroleum ether/Acetone = 5:1

^{1}H NMR (400 MHz, $CDCl_3$) δ 9.47 (d, *J* = 8.0 Hz, 1H), 7.84 (d, J = 7.6 Hz, 1H), 7.72 (d, *J* = 8.8 Hz, 1H), 7.44 (q, *J* = 8.0 Hz, 2H), 7.37 (t, *J* = 7.6 Hz, 1H), 7.30 (t, *J* = 7.6 Hz, 1H), 7.20 (d, *J* = 8.0 Hz, 1H), 7.13 (br, 1H), 6.62 (dd, *J* = 8.0, 5.6 Hz, 1H), 5.19 - 5.09 (m, 1H), 4.96 (s, 2H), 3.99 (d, *J* = 2.0 Hz, 1H), 2.86 (s, 3H), 2.58 - 2.49 (m, 2H), 2.49 - 2.45 (m, 3H), 2.42 (s, 3H), 1.87 - 1.77 (m, 3H), 1.73 (d, *J* = 15.2 Hz, 3H), 1.66 - 1.45 (m, 2H), 1.31 - 1.23 (m, 3H).

^{13}C NMR (100 MHz, $CDCl_3$) δ 177.1, 173.5, 138.7, 136.6, 132.6, 130.5, 126.9, 126.3, 125.4, 125.0, 124.7, 123.7, 121.5, 120.5, 120.1, 119.3, 116.3, 114.6, 112.4, 107.8, 94.7, 84.9, 82.5, 60.6, 48.9, 46.1, 41.5, 31.3, 29.3, 29.2, 28.2, 25.95, 25.91, 25.85.

HRMS (ESI, *m/z*) calcd for $C_{35}H_{36}N_4NaO_4^+$ $[M+Na]^+$: 599.2629, found: 599.2631.

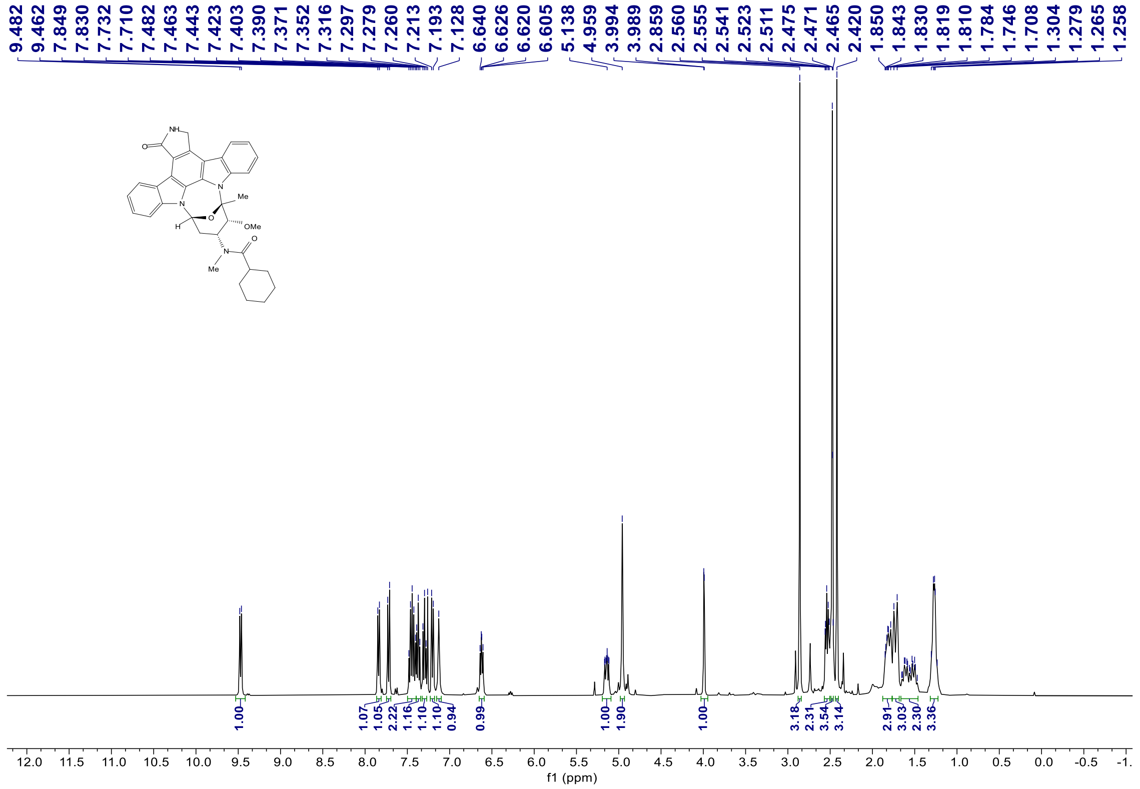

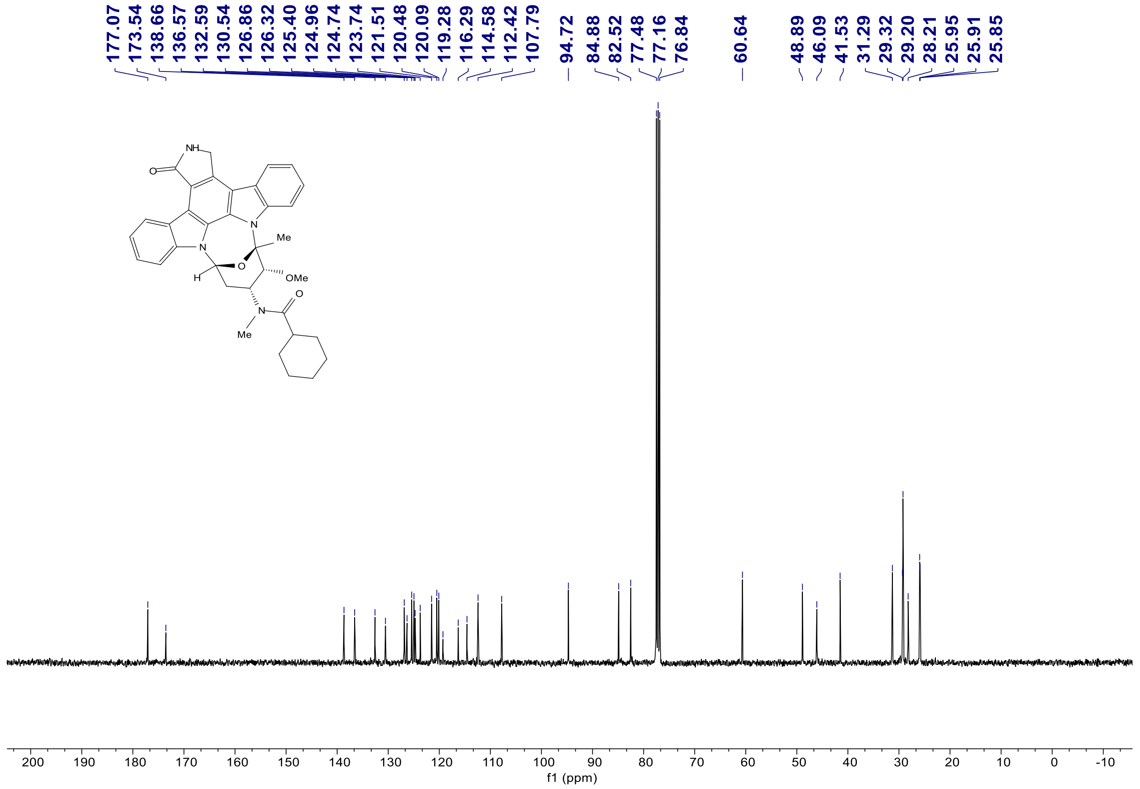

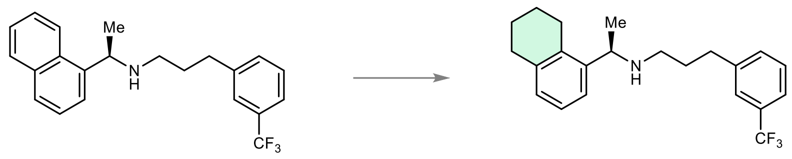

(R)-Cinacalcet **39** (35% yield)

According to the general procedure, a mixture of ***(R)*-Cinacalcet** (71.4 mg, 0.20 mmol, 1.0 equiv.), $B_2(OH)_4$ (107.5 mg, 1.20 mmol, 6.0 equiv.) and $[Rh(COD)OH]_2$ (3.7 mg, 4.0 mol%, 0.008 mmol) in EtOH (1.0 mL, 0.2 M) was stirred under nitrogen atmosphere for 48 hours at 50 °C to afford **39** as a colorless oil (25.4 mg, 35% yield).

Purification conditions: CH_2Cl_2/NH_3(7.0 M solution in MeOH) = 500:1 to 200:1.

R_f= 0.25 in CH_2Cl_2/NH_3(7.0 M solution in MeOH) = 200:1.

^{1}H NMR (400 MHz, $CDCl_3$) δ 7.38 - 7.32 (m, 2H), 7.31 - 7.23 (m, 2H), 7.22 - 7.17 (m, 1H), 7.06 (t, J = 7.6 Hz, 1H), 6.90 (d, J = 7.6 Hz, 1H), 3.98 (q, J = 6.4 Hz, 1H), 2.73 (t, J = 6.4 Hz, 2H), 2.69 - 2.54 (m, 4H), 2.52 - 2.39 (m, 2H), 1.78 - 1.66 (m, 7H), 1.22 (d, J = 6.8 Hz, 3H).

^{13}C NMR (100 MHz, $CDCl_3$) δ 143.2, 137.5, 134.3, 131.91, 131.90, 130.72 (q, J = 31.9 Hz), 128.5, 128.0, 125.9, 125.20 (q, J = 3.7 Hz), 124.4 (q, J = 282 Hz), 122.79 (q, J = 3.8 Hz), 122.5, 77.5, 77.2, 76.8, 52.8, 47.2, 33.5, 31.8, 30.5, 25.9, 23.5, 23.2, 22.8.

^{19}F NMR (376 MHz, $CDCl_3$) δ -62.52.

HRMS (ESI, *m/z*) calcd for $C_{22}H_{27}F_3N^+$ $[M+H]^+$: 362.2096, found: 362.2095.

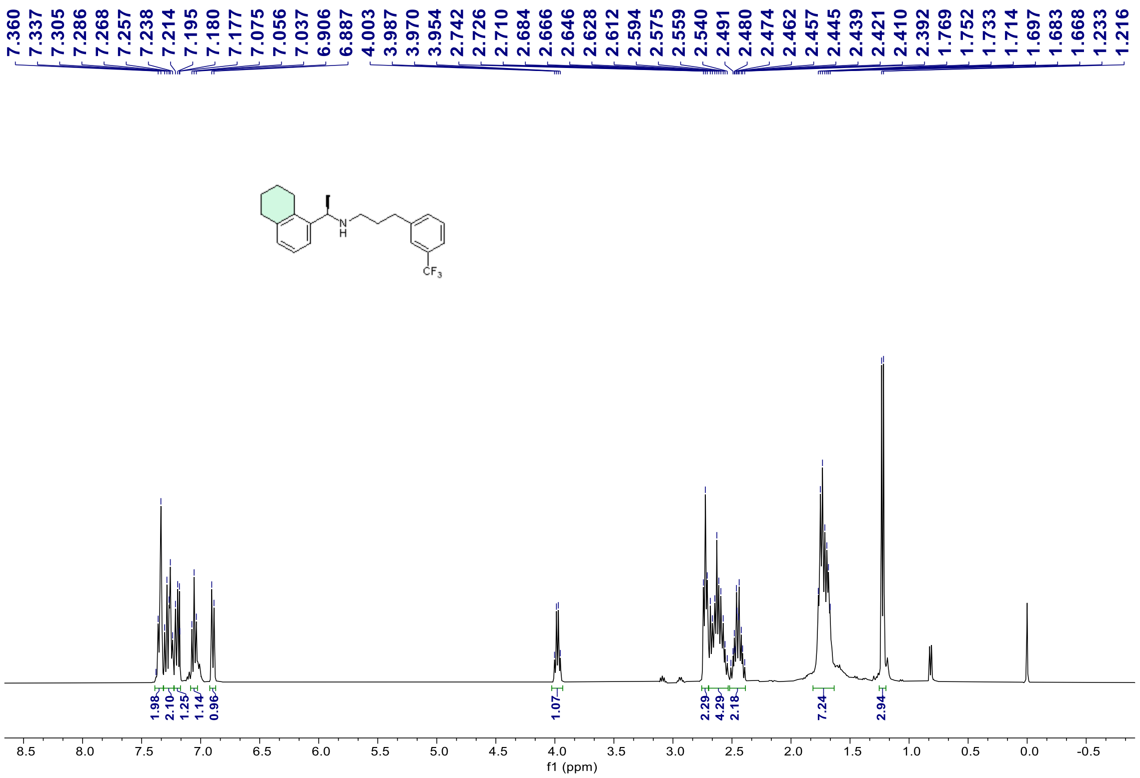

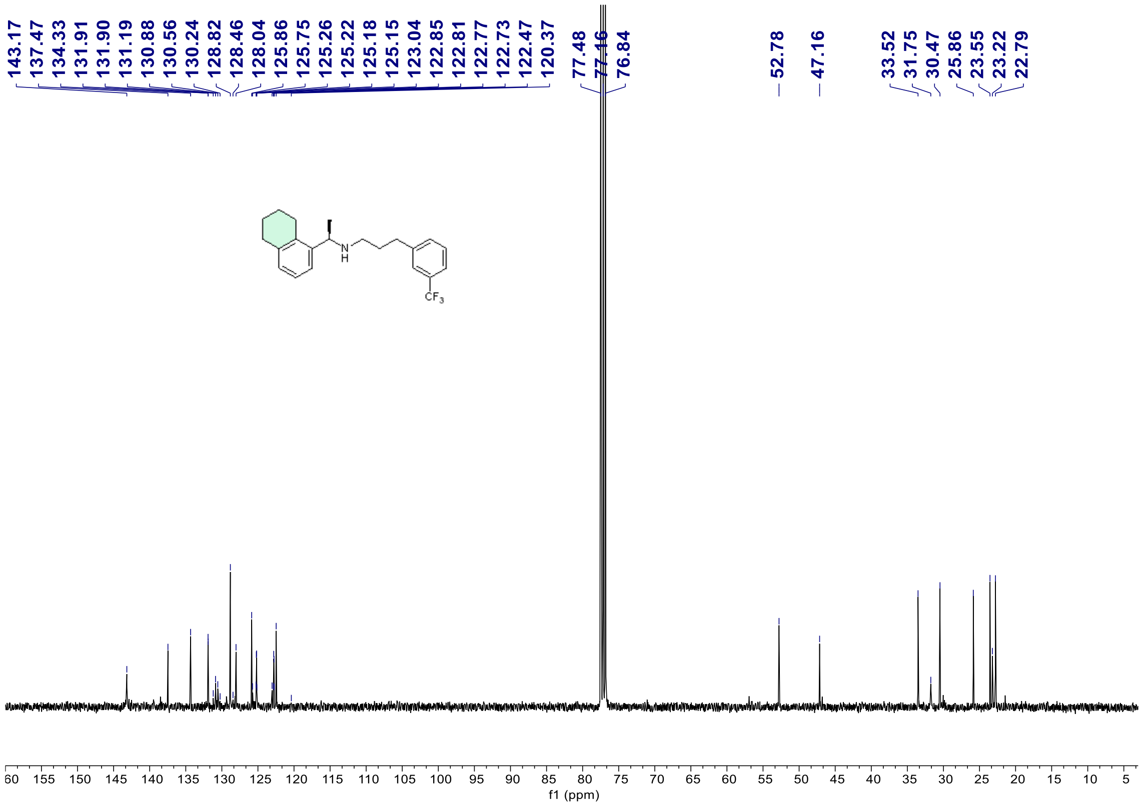

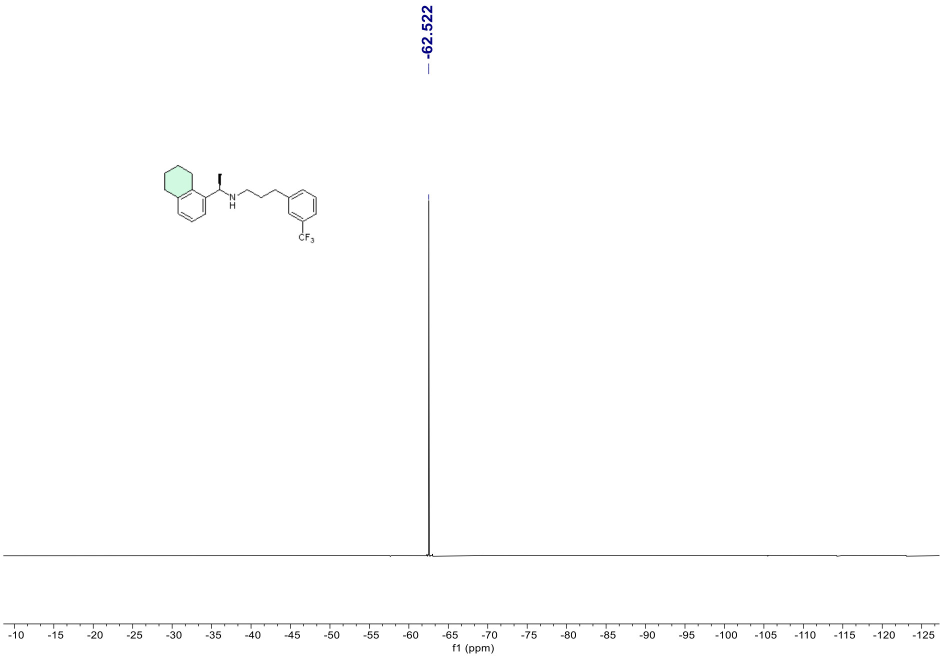

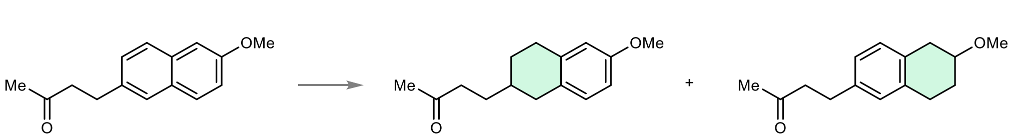

40 Nabumetone **41** (53% yield) **42** (27% yield)

According to the general procedure, a mixture of **nabumetone** (45.7 mg, 0.2 mmol, 1.0 equiv.), $B_2(OH)_4$ (53.7 mg, 0.6 mmol, 3.0 equiv.) and $[Rh(COD)OH]_2$ (2.3 mg, 2.5 mol%, 0.005 mmol) in

EtOH (1.0 mL, 0.2 M) was stirred under argon atmosphere for 14 hours at 50 °C to afford **41** (24.5 mg, 53% yield) and **42** (12.4 mg, 27% yield) as colorless oil.

Analytical data of **41**:

^{1}H NMR (600 MHz, $CDCl_3$) δ 6.96 (d, *J* = 8.4 Hz, 1H), 6.68 (d, *J* = 8.5 Hz, 1H), 6.61 (s, 1H), 3.77 (s, 3H), 2.81-2.74 (m, 3H), 2.54 (t, *J* = 6.8 Hz, 2H), 2.35 (dd, *J* = 16.2, 9.4 Hz, 1H), 2.17 (s, 3H), 1.94-1.87 (m, 1H), 1.69-1.63 (m, 3H), 1,42-1.35 (m, 1H).

^{13}C NMR (151 MHz, $CDCl_3$) δ 209.2, 157.7, 137.8, 130.0, 128.5, 113.5, 112.0, 55.4, 41.5, 35.3, 34.2, 30.3, 30.0, 29.5, 29.4.

HRMS (ESI, *m*/*z*) calcd for $C_{15}H_{20}O_2Na^+$ $[M+Na]^+$: 255.1355, found: 255.1354.

Analytical data of **42**:

^{1}H NMR (600 MHz, $CDCl_3$) δ 7.04 - 6.97 (m, 1H), 6.93 (d, *J* = 8.1 Hz, 1H), 6.91 (s, 1H), 3.67 - 3.63 (m, 1H), 3.42 (s, 3H), 3.04 (dd, *J* = 16.4, 4.8 Hz, 1H), 2.92 - 2.87 (m, 1H), 2.84 - 2.81 (m, 2H), 2.77 - 2.72 (m, 4H), 2.14 (s, 3H), 2.08 - 2.05 (m, 1H), 1.85 - 1.78 (m, 1H).

^{13}C NMR (151 MHz, $CDCl_3$) δ 208.2, 138.6, 136.3, 132.4, 129.7, 128.5, 125.9, 75.9, 56.0, 45.5, 34.9, 30.2, 29.5, 27.8, 27.0.

HRMS (ESI, *m*/*z*) calcd for $C_{15}H_{20}O_2Na^+$ $[M+Na]^+$: 255.1355, found: 255.1355.

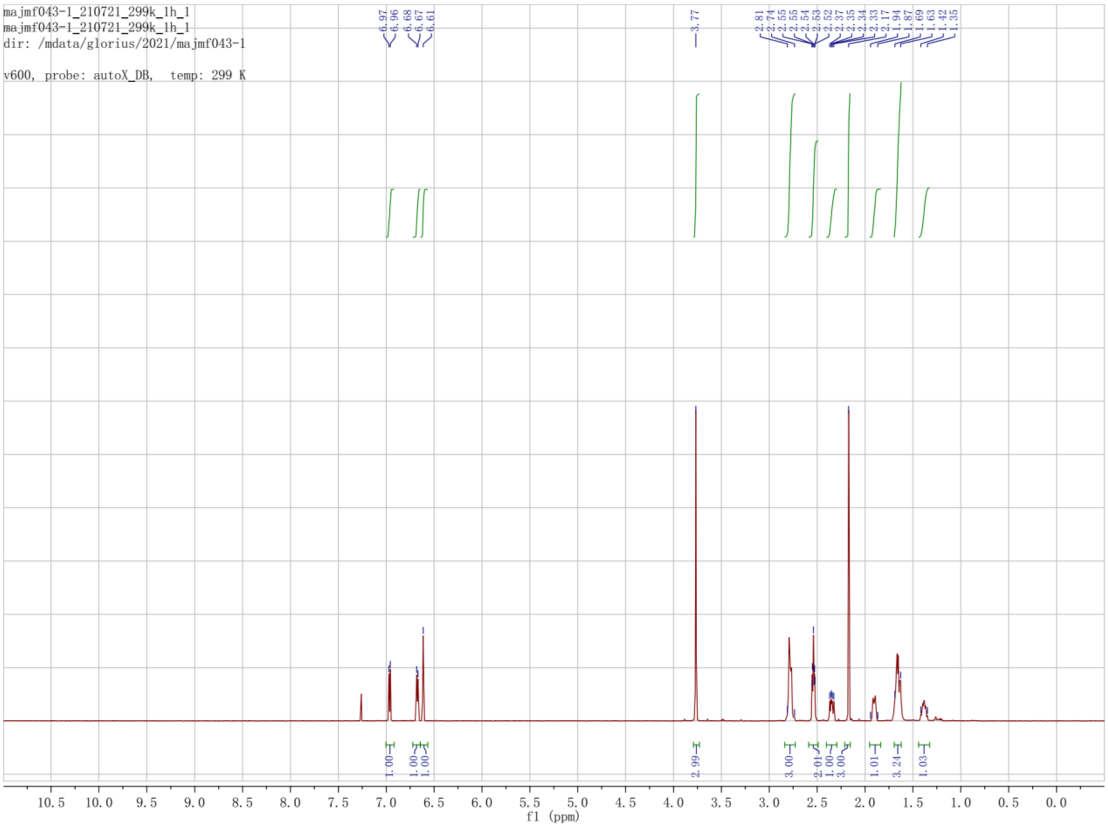

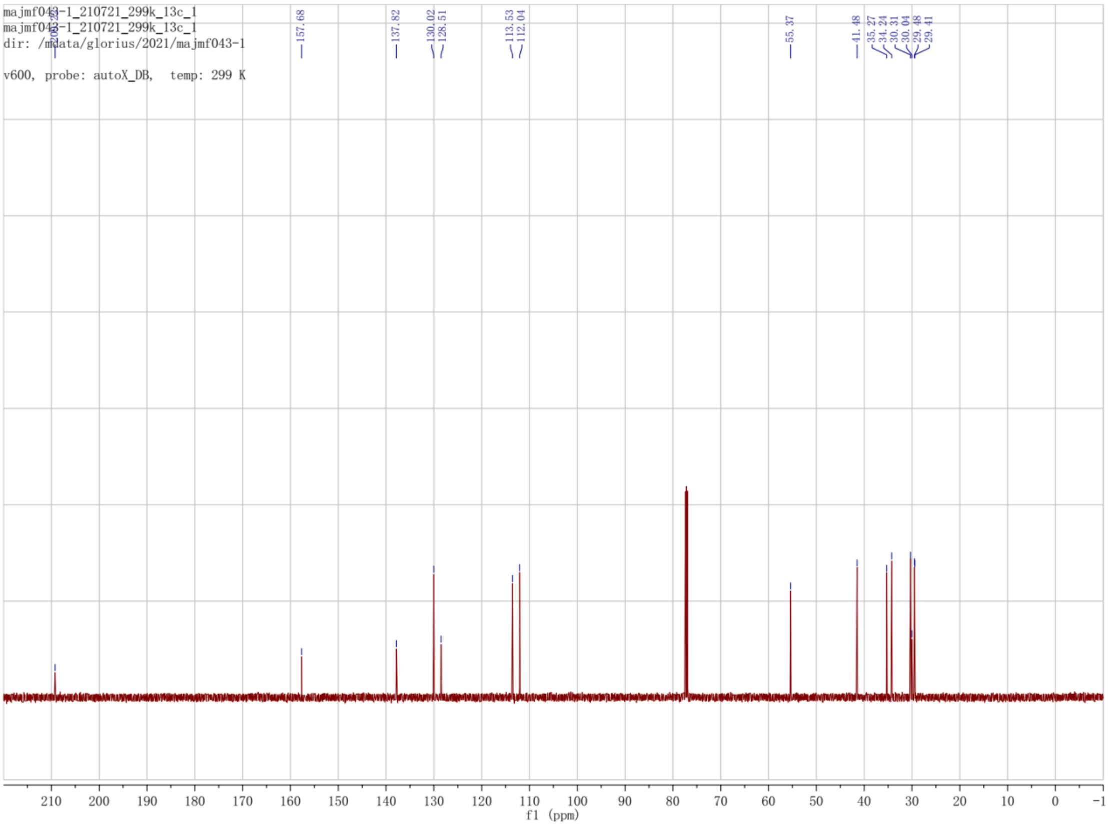

majmf043-1_210721_299k_13c_1
majmf043-1_210721_299k_13c_1
dir: /mdata/glorius/2021/majmf043-1
v600, probe: autoX_DB, temp: 299 K
157.68
137.82
130.02
128.51
113.53
112.04
55.37
41.48
35.27
34.24
30.31
30.04
29.48
29.41
f1 (ppm)

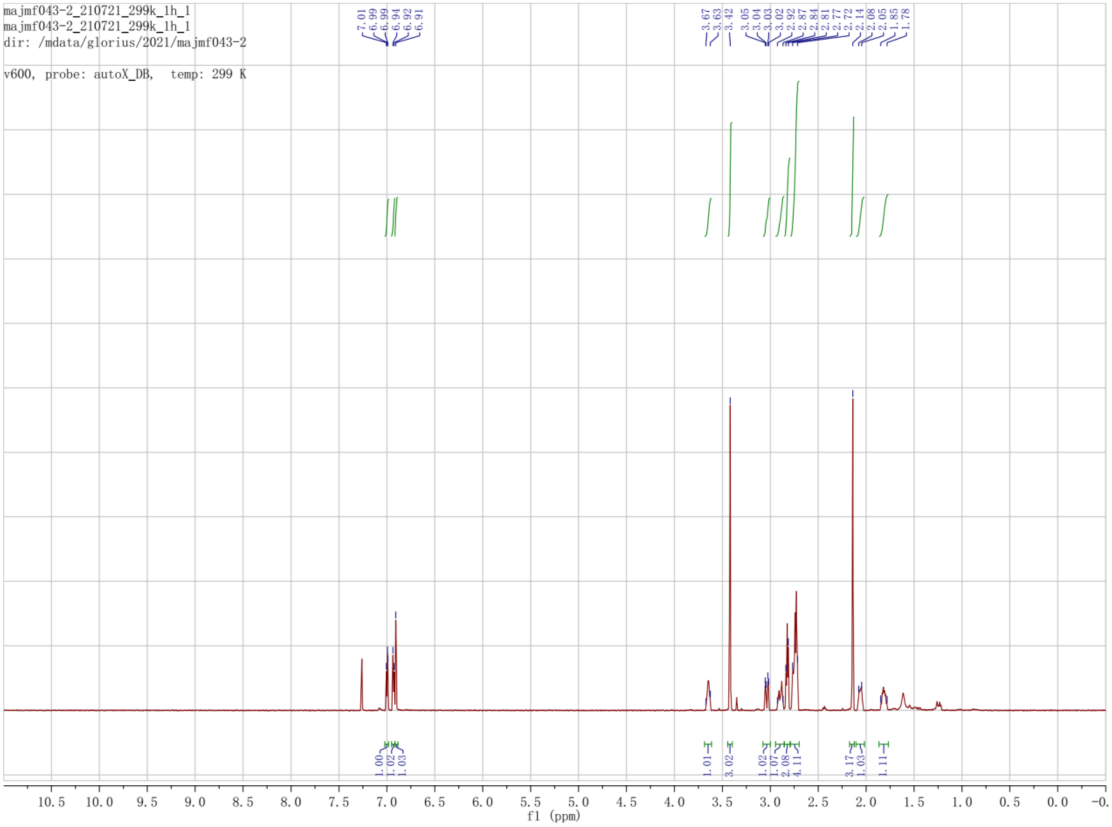

majmf043-2_210721_299k_1h_1
majmf043-2_210721_299k_1h_1
dir: /mdata/glorius/2021/majmf043-2
v600, probe: autoX_DB, temp: 299 K
7.01
6.99
6.99
6.94
6.92
6.91
3.67
3.63
3.42
3.05
3.04
3.03
3.02
2.92
2.87
2.84
2.81
2.77
2.72
2.14
2.08
2.05
1.85
1.78
1.00
1.02
1.03
1.01
3.02
1.02
1.07
2.08
4.11
3.17
1.03
1.11
f1 (ppm)

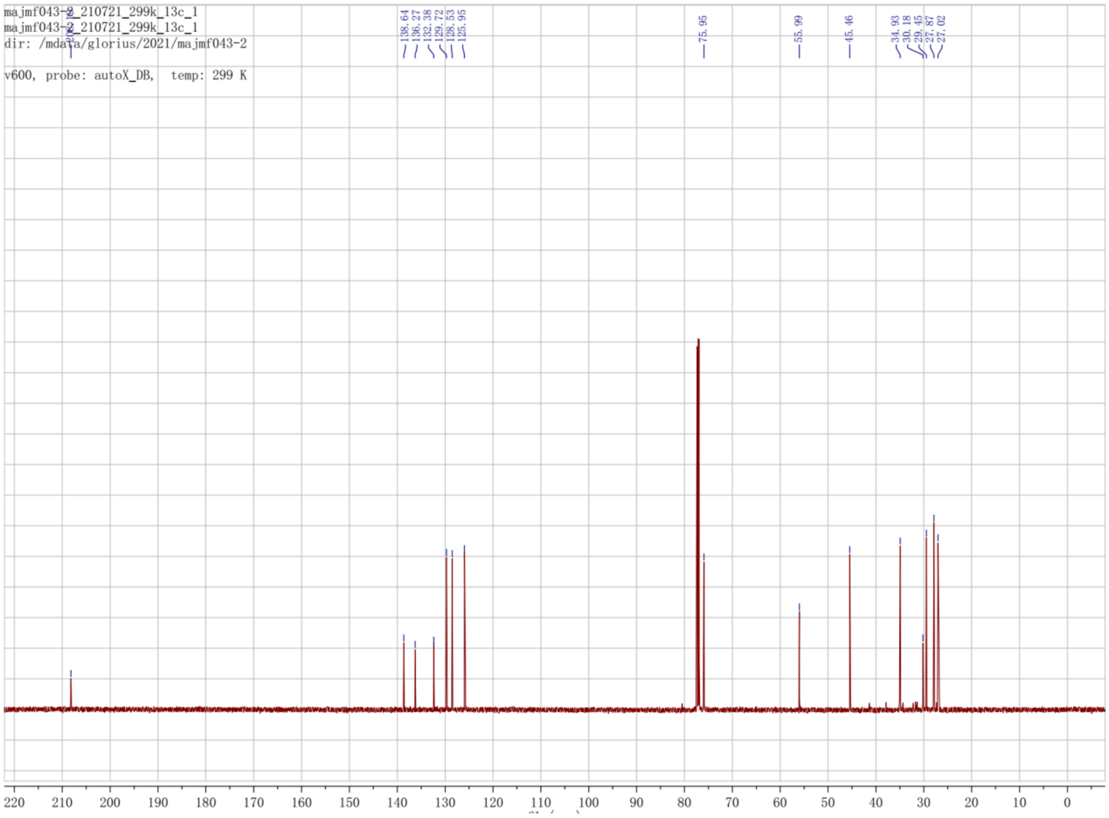

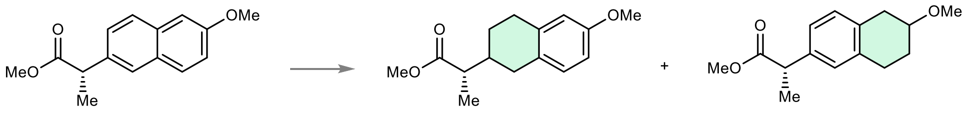

43 (*S*)-**Naproxen**-OMe **44** (38% yield) **45** (44% yield)

According to the general procedure, a mixture of **(*S*)-Naproxen-OMe** (48.8 mg, 0.2 mmol, 1.0 equiv.), $B_2(OH)_4$ (80.6 mg, 0.9 mmol, 4.5 equiv.) and $[Rh(COD)OH]_2$ (2.3 mg, 2.5 mol%, 0.005 mmol) in EtOH (1.0 mL, 0.2 M) was stirred under argon atmosphere for 14 hours at 50 °C to afford **44** (18.6 mg, 38% yield, 52:48 d.r.) and **45** (21.7 mg, 44% yield, 50:50 d.r.) as white solid.

Analytical data of **44**:

^{1}H NMR (599 MHz, $CDCl_3$, mixture of diastereomers) δ 6.97 (m, 1H), 6.68 (m, 1H), 6.62 (s, 1H), 3.77 (s, 3H), 3.70 (s, 3H), 2.83 - 2.68 (m, 3H), 2.51 - 2.42 (m, 2H), 2.14 - 1.81 (m, 2H), 1.48 - 1.39 (m, 1H), 1.23 - 1.21 (m, 3H).

^{13}C NMR (151 MHz, $CDCl_3$, mixture of diastereomers) δ 176.63, 176.56, 157.60, 157.56, 137.46, 137.37, 129.99, 129.94, 128.12, 127.98, 113.31, 113.30, 112.01, 111.96, 55.23, 55.22, 51.44, 44.66, 44.42, 37.67, 37.61, 32.99, 31.91, 29.56, 29.47, 27.64, 26.19, 14.28, 14.12.

HRMS (ESI, *m*/*z*) calcd for $C_{15}H_{20}O_3Na^+$ $[M+Na]^+$: 271.1304, found: 271.1304.

Analytical data of **45**:

^{1}H NMR (599 MHz, $CDCl_3$, mixture of diastereomers) δ 7.04 (d, J = 1.2 Hz, 2H), 7.01 (s, 1H), 3.67-3.62 (m, 5H), 3.42 (s, 3H), 3.05 (dd, J = 16.3, 4.9 Hz, 1H), 2.92 (dq, J = 16.5, 5.5 Hz, 1H), 2.76 (dd, J = 16.3, 7.4 Hz, 2H), 2.18 - 1.99 (m, 1H), 1.86 - 1.78 (m, 1H), 1.47 (d, J = 7.1 Hz, 3H).

^{13}C NMR (151 MHz, $CDCl_3$, mixture of diastereomers) δ 175.31, 175.29, 138.26, 138.24, 136.48, 136.47, 133.55, 133.54, 129.88, 129.87, 127.69, 127.65, 125.12, 125.07, 75.84, 56.00, 52.14, 45.19, 45.17, 34.98, 34.97, 27.74, 27.73, 26.99, 26.97, 18.81, 18.79.

HRMS (ESI, m/z) calcd for $C_{15}H_{20}O_3Na^+$ $[M+Na]^+$: 271.1304, found: 271.1304.

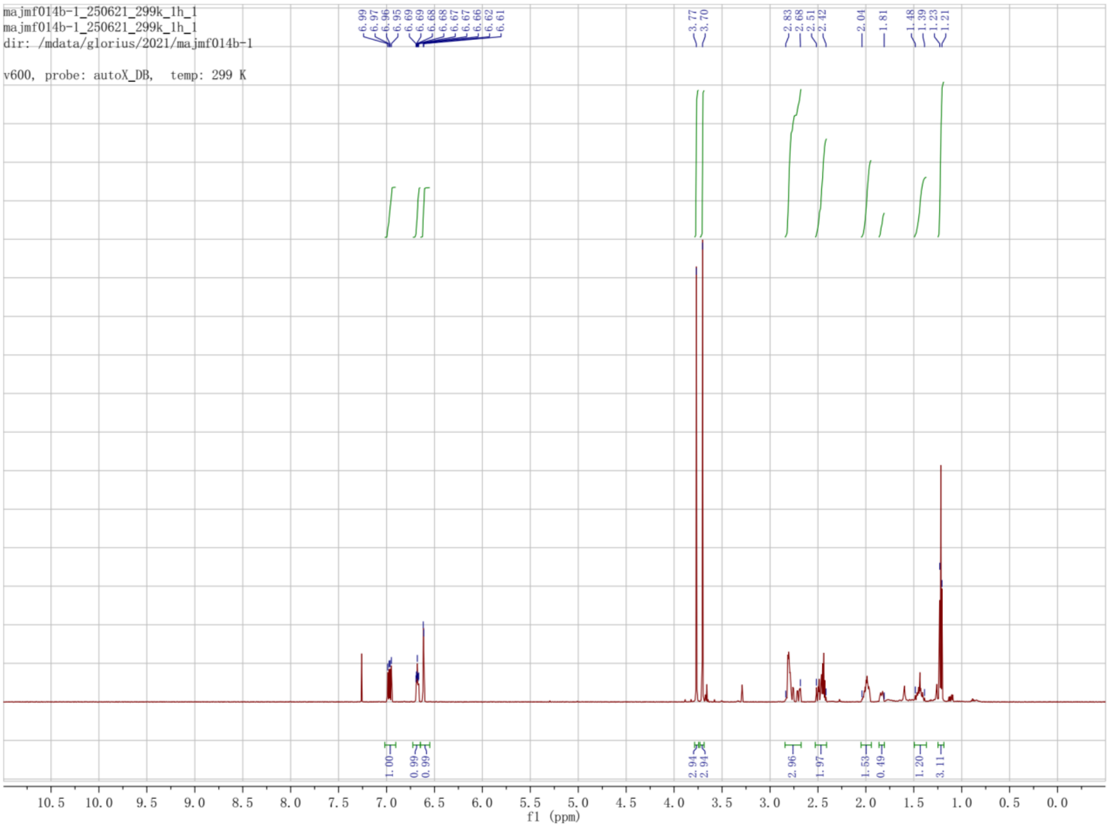

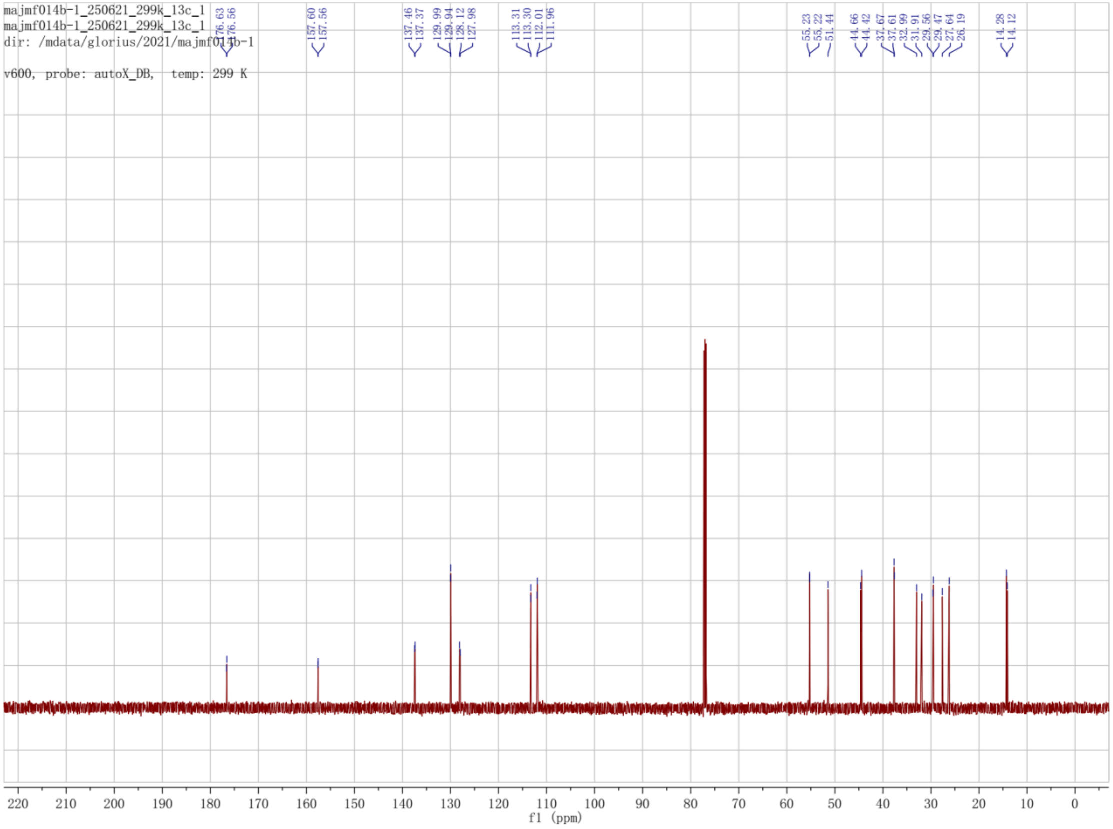
majmf014b-1_250621_299k_13c_1
majmf014b-1_250621_299k_13c_1
dir: /mdata/glorius/2021/majmf014b-1
v600, probe: autoX_DB, temp: 299 K
176.63
176.56
157.60
157.56
137.46
137.37
129.99
129.94
128.12
127.98
113.31
113.30
112.01
111.96
55.23
55.22
51.44
44.66
44.42
37.67
37.61
32.99
31.91
29.56
29.47
27.64
26.19
14.28
14.12
f1 (ppm)

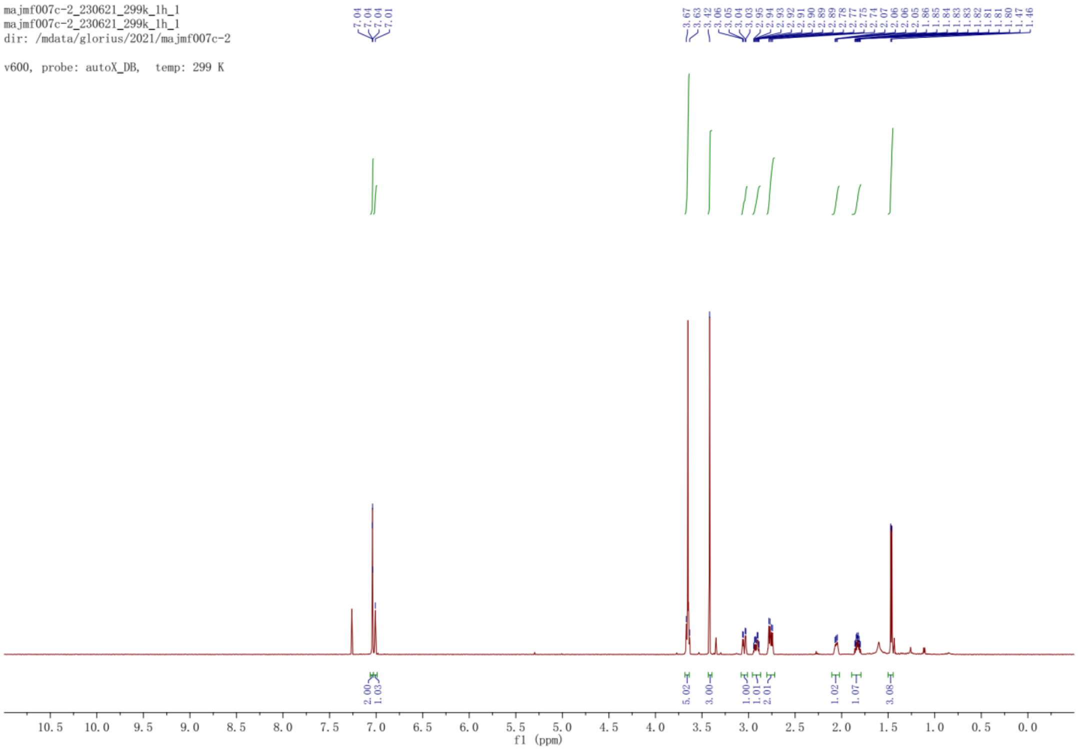
majmf007c-2_230621_299k_1h_1
majmf007c-2_230621_299k_1h_1
dir: /mdata/glorius/2021/majmf007c-2
v600, probe: autoX_DB, temp: 299 K
7.04
7.04
7.04
7.01
3.67
3.63
3.42
3.06
3.05
3.04
3.03
2.95
2.94
2.93
2.92
2.91
2.90
2.89
2.78
2.77
2.75
2.74
2.07
2.06
2.06
2.05
1.86
1.85
1.84
1.83
1.83
1.82
1.81
1.81
1.80
1.47
1.46
2.00
1.03
5.02
3.00
1.00
1.01
2.01
1.02
1.07
3.08
f1 (ppm)

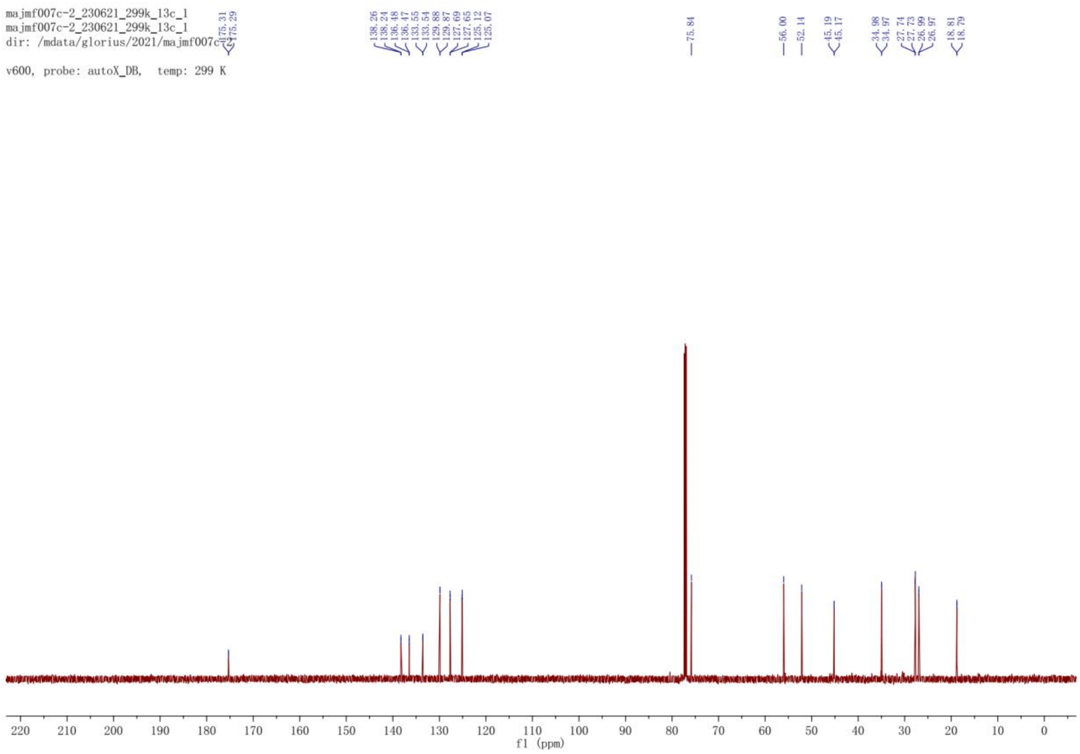

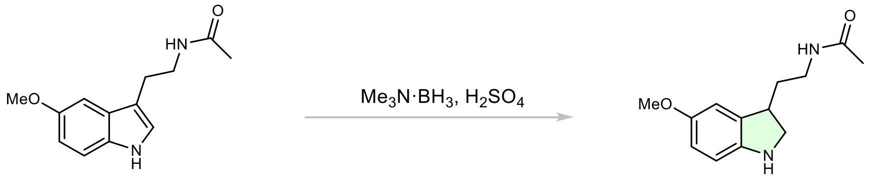

Melatonin **46** (95% yield)

To a Schenk tube, H_2SO_4 (98%) (19.6 mg, 2.0 mmol, 10.0 equiv.) was added to a solution of **melatonin** (46.5 mg, 0.20 mmol, 1.0 equiv.) and $Me_3N{\bullet}BH_3$ (38.6 mg, 0.50 mmol, 2.5 equiv.) in H_2O/CN_3CN (*v*/*v* = 10:1, 1.1 mL).[5] The mixture was stirred at 40 °C for 12 hours. Subsequently, the reaction mixture was neutralized with saturated $NaHCO_3$ solution and extracted with CH_2Cl_2 for three times. The combined organic phase was dried over anhydrous sodium sulfate, and concentrated under reduced pressure. The residue was purified by flash column chromatography over silica gel to afford **46** as a yellow oil (44.5 mg, 95% yield).

Purification conditions: CH_2Cl_2/MeOH = 50:1 to 20:1

R_f= 0.4 in CH_2Cl_2/MeOH = 15:1.

^{1}H NMR (400 MHz, $CDCl_3$) δ 6.67 (s, 1H), 6.59 - 6.51 (m, 2H), 6.47 - 6.36 (m, 1H), 3.69 (s, 3H), 3.65 - 3.58 (m, 1H), 3.34 - 3.21 (m, 3H), 3.20 - 3.12 (m, 2H), 1.97 - 1.91 (m, 1H), 1.90 (s, 3H), 1.74 1 1.62 (m, 1H).

^{13}C NMR (100 MHz, $CDCl_3$) δ 170.5, 153.6, 144.9, 134.0, 112.5, 110.8, 110.5, 56.0, 53.6, 40.2, 37.4, 33.7, 23.2.

HRMS (ESI, *m/z*) calcd for $C_{13}H_{19}N_2O_2^+$ $[M+H]^+$: 235.1447, found: 235.1450.

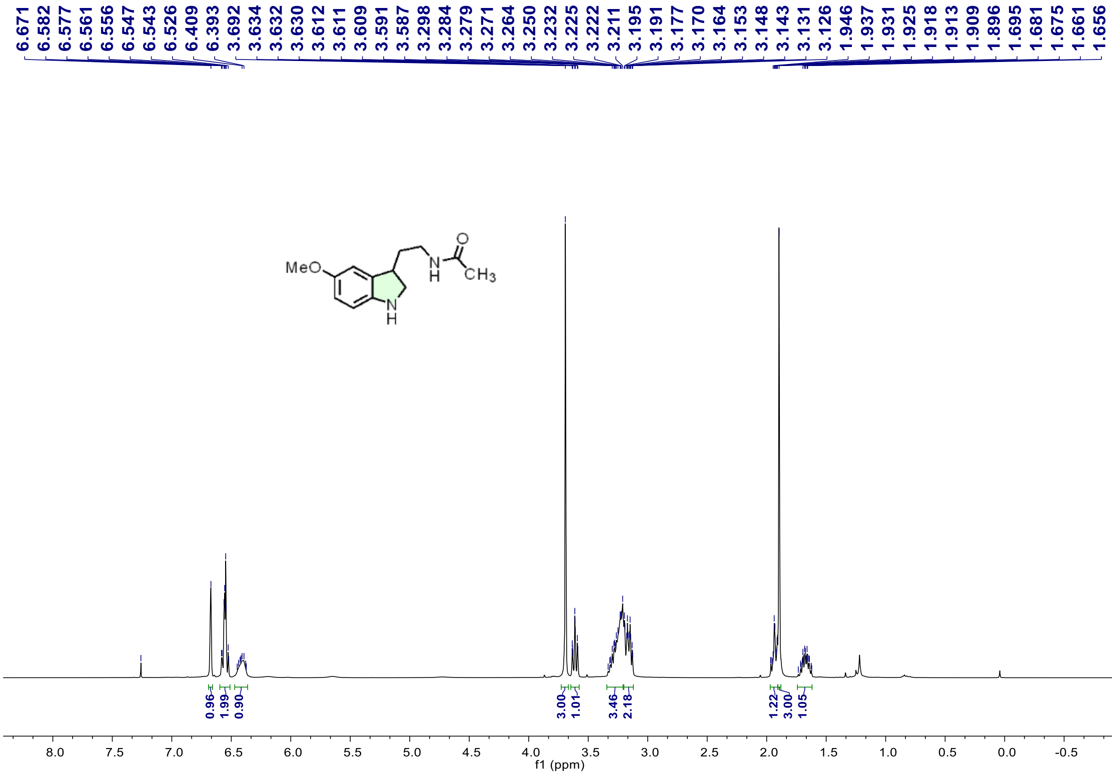

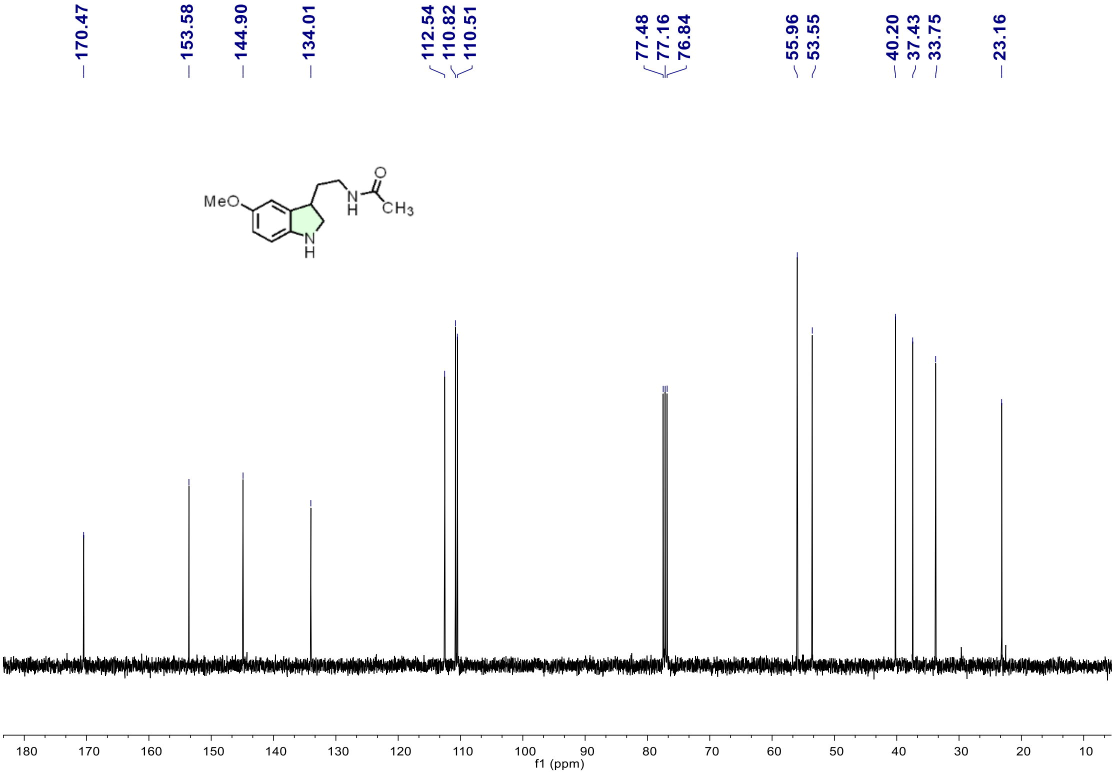

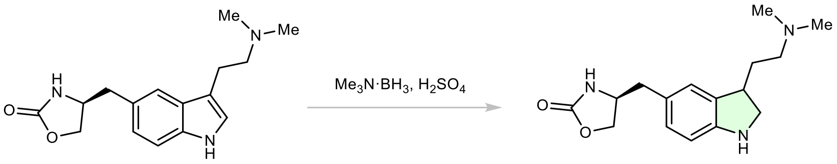

To a Schenk tube, H_2SO_4 (98%) (19.6 mg, 2.0 mmol, 10.0 equiv.) was added to a solution of (*S*)-**zolmitriptan** (57.5 mg, 0.20 mmol, 1.0 equiv.) and Me_3N•BH_3 (38.6 mg, 0.50 mmol, 2.5 equiv.) in H_2O/CN_3CN (*v*/*v* = 10:1, 1.1 mL). The mixture was stirred at 40 °C for 12 hours. Subsequently, the reaction mixture was neutralized with saturated $NaHCO_3$ solution and extracted with CH_2Cl_2 for three times. The combined organic phase was dried over anhydrous sodium sulfate, and concentrated under reduced pressure. The residue was purified by flash column chromatography over silica gel to afford **47** as a yellow oil (46.3 mg, 80% yield; d.r. = 1:1 was determined by ^{1}H-NMR).

Purification conditions: Reverse-phase column chromatography (MeOH/H_2O = 1:50 to 1:3) on SepaBean machine (Santai Technology Inc., China) equipped with C18-bonded SepaFlash columns. R_f = 0.45 in NH_3(7.0 M solution in MeOH)/CH_2Cl_2 = 1:10.

^{1}H NMR (400 MHz, $CDCl_3$, mixture of two diastereomers) δ 6.89 (d, *J* = 9.6 Hz, 1H), 6.81 (d, *J* = 7.6 Hz, 1H), 6.56 (d, *J* = 8.0 Hz, 1H), 5.69 (d, *J* = 20.0 Hz, 1H), 4.42 (t, *J* = 8.4 Hz, 1H), 4.16 - 4.08 (m, 1H), 4.05 - 3.94 (m, 1H), 3.68 (td, *J* = 8.4, 2.0 Hz, 2H), 3.32 - 3.24 (m, 1H), 3.24 - 3.18 (m, 1H), 2.75 (d, *J* = 8.0 Hz, 2H), 2.45 - 2.31 (m, 2H), 2.28 (s, 3H), 2.25 (s, 3H), 2.04 - 1.93 (m, 1H), 1.78 - 1.64 (m, 1H).

^{13}C NMR (100 MHz, $CDCl_3$, one of two diastereomers) δ 159.5, 150.6, 133.8, 128.3, 126.0, 124.8, 109.8, 69.8, 57.6, 54.3, 53.7, 45.5, 41.2, 40.2, 32.2.

^{13}C NMR (100 MHz, $CDCl_3$, the second diastereomer) δ 159.5, 150.6, 133.6, 128.3, 125.9, 124.6, 109.8, 69.7, 57.5, 54.2, 53.6, 45.4, 41.1, 40.2, 31.8.

HRMS (ESI, *m*/*z*) calcd for $C_{16}H_{24}N_3O_2^+$ $[M+H]^+$: 290.1869, found: 290.1870.

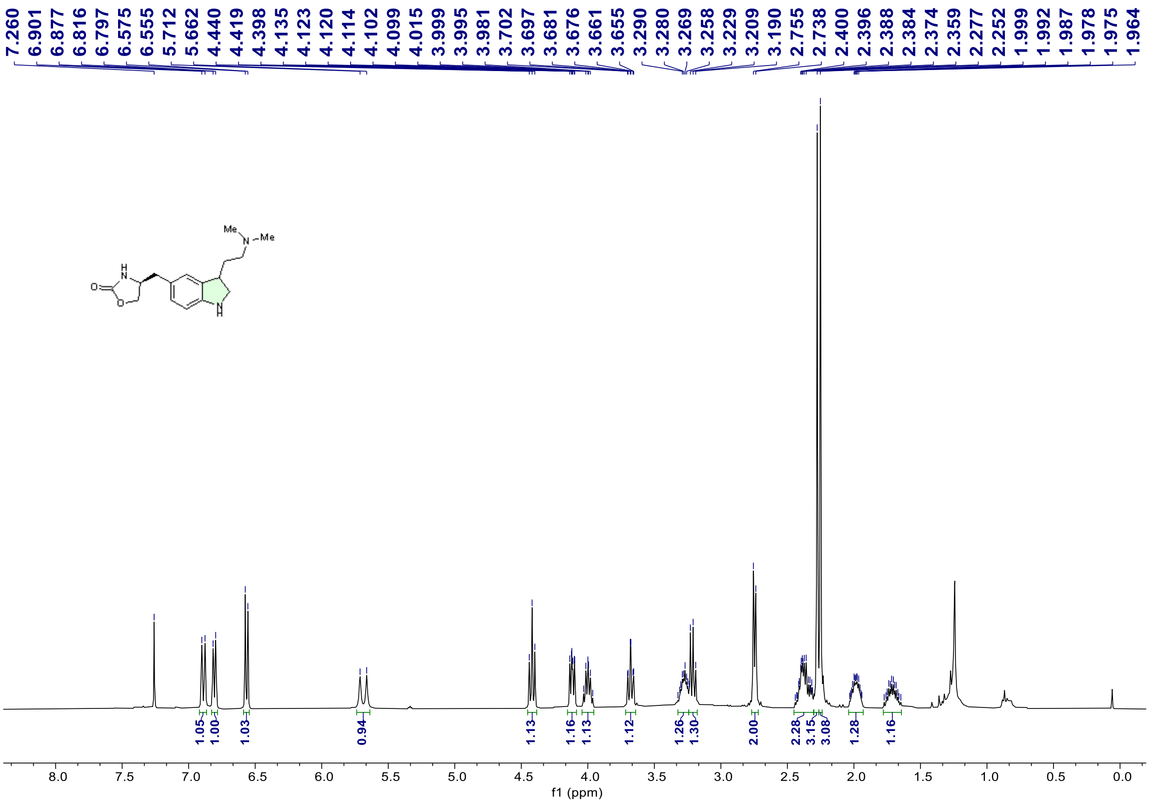
7.260
6.901
6.877
6.816
6.797
6.575
6.555
5.712
5.662
4.440
4.419
4.398
4.135
4.123
4.120
4.114
4.102
4.099
4.015
3.995
3.981
3.702
3.697
3.681
3.676
3.661
3.655
3.290
3.280
3.269
3.258
3.229
3.209
3.190
2.755
2.738
2.400
2.396
2.388
2.384
2.374
2.359
2.277
2.252
1.999
1.992
1.987
1.978
1.975
1.964
Me
N
Me
H
N
O
O
N
H
1.05
1.00
1.03
0.94
1.13
1.16
1.13
1.12
1.26
1.30
2.00
2.28
3.15
3.08
1.28
1.16
8.0
7.5
7.0
6.5
6.0
5.5
5.0
4.5
4.0
3.5
3.0
2.5
2.0
1.5
1.0
0.5
0.0
f1 (ppm)

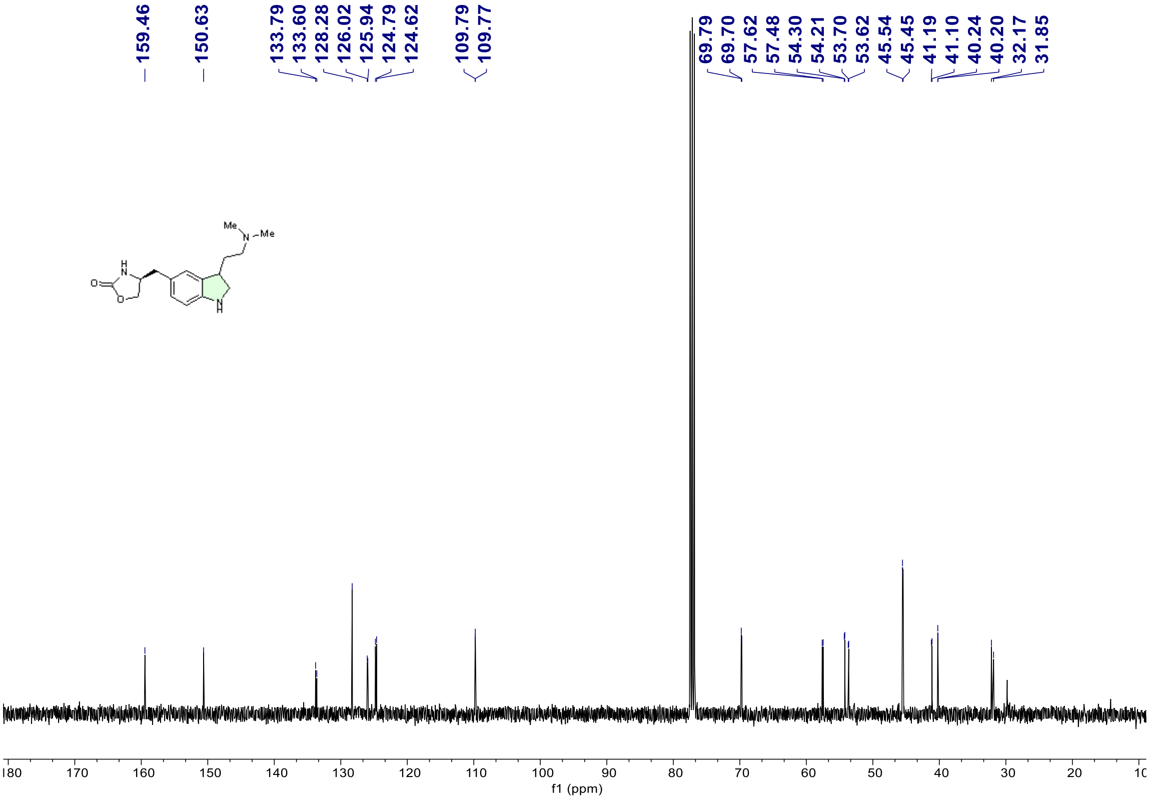
159.46
150.63
133.79
133.60
128.28
126.02
125.94
124.79
124.62
109.79
109.77
69.79
69.70
57.62
57.48
54.30
54.21
53.70
53.62
45.54
45.45
41.19
41.10
40.24
40.20
32.17
31.85
Me
N
Me
H
N
O
O
N
H
180
170
160
150
140
130
120
110
100
90
80
70
60
50
40
30
20
f1 (ppm)

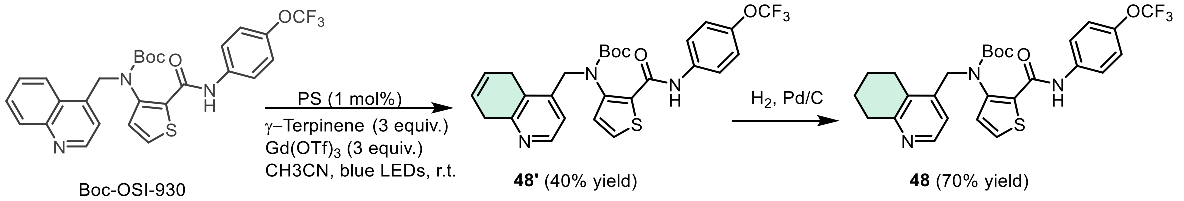

A dried 10 mL Schlenk tube was charged with the Boc-OSI-930 (0.2 mmol, 108.7 mg, 1.0 equiv.), Ir[dF(Me)ppy]$_2$(dtbbpy)PF$_6$ (0.002 mmol, 1.0 mol%, 2.2 mg), Gd(OTf)$_3$ (0.4 mmol, 2.0 equiv., 242 mg), *γ*-terpinene (0.6 mmol, 3.0 equiv., 96 μL) and CH_3CN (4.0 mL, 0.05 M). The mixture was degassed by three cycles of freeze-pump-thaw, subsequently irradiated with 10 W blue LEDs (λ_{max} =455 nm) for 4 hours under nitrogen at 20 °C. After the starting material was fully converted as checked by TLC, the reaction was quenched by saturated aqueous $NaHCO_3$ and extracted with CH_2Cl_2 (10 mL × 4). The organic phases were combined and concentrated under reduced pressure. The crude residue was purified by flash chromatography on silica gel to give the desired product **48'** as a colorless oil (43.6 mg, 40% yield).

Purification conditions: EtOAc/petroleum ether = 1:4 to 1:1

R_f (**48**') = 0.4 in EtOAc/petroleum ether = 1:1.

Analytical data of **48'**:

1H NMR (500 MHz, CDCl3) δ 8.83 - 8.61 (m, 1H), 8.29 (d, J = 5.0 Hz, 1H), 7.47 (d, J = 8.5 Hz, 2H), 7.42 (d, J = 5.5 Hz, 1H), 7.16 (d, J = 8.5 Hz, 2H), 6.95 (d, J = 5.0 Hz, 1H), 6.84 (d, J = 5.0 Hz, 1H), 5.90 - 5.84 (m, 1H), 5.75 - 5.68 (m, 1H), 4.73 (s, 2H), 3.50 - 3.43 (m, 2H), 3.28 - 3.21 (m, 2H), 1.41 (s, 9H).

All the other data is consistent with a previous report.[21]

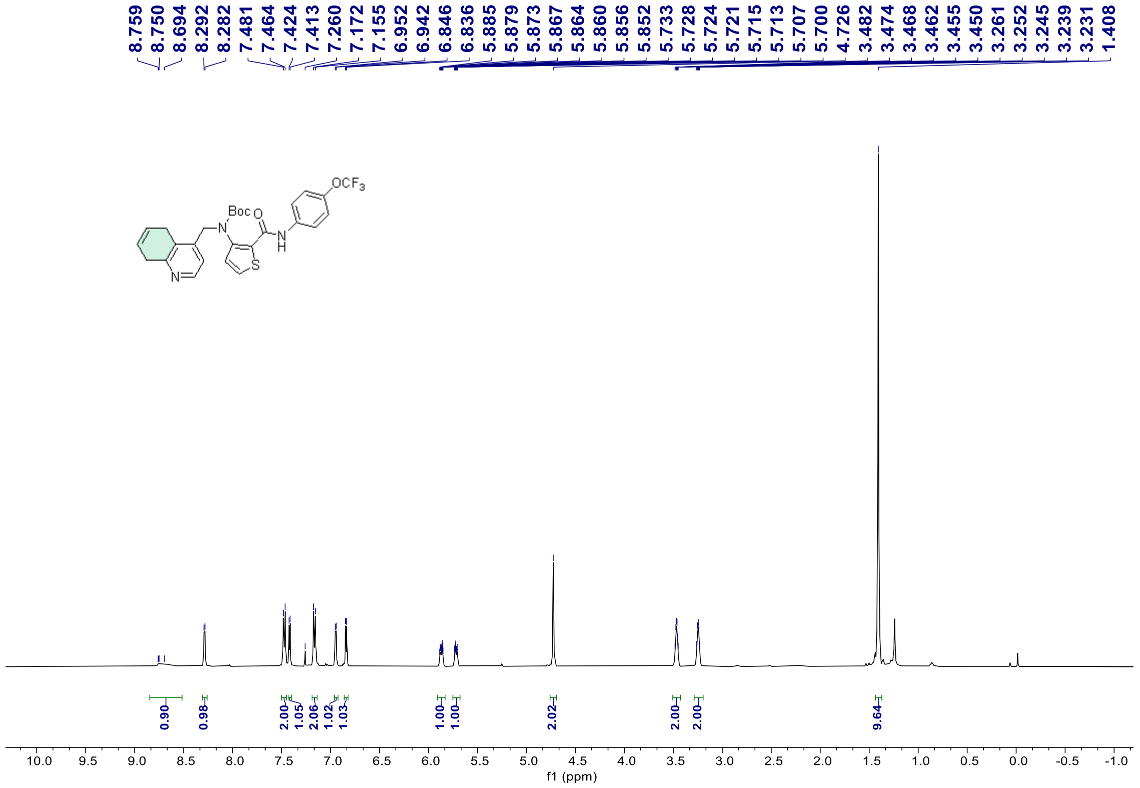

To a solution of **48’** (54.5 mg, 0.1 mmol, 1.0 equiv.), $KHCO_3$ (2.0 mg, 0.02 mmol, 0.2 equiv.) and Pd/C (Pd 5% on Carbon wetted with ca. 55% water, 11 mg) in MeOH (1.5 mL) was stirred for overnight under hydrogen atmosphere at room temperature. After completion of reaction, the resulting mixture was filtered by diatomaceous earth and washed with EtOAc (3 × 3mL). The organic phases were combined and concentrated under reduced pressure. The crude residue was purified by flash chromatography on silica gel to give the product **48** as a colorless oil (48.1 mg, 70% yield).

Purification conditions: EtOAc/petroleum ether = 1:5 to 1:2

R_f (**48**) = 0.3 in EtOAc/petroleum ether = 1:1.

Analytical data of **48**:

^1H NMR (500 MHz, $CDCl_3$) δ 8.81 (br, 1H), 8.24 (d, J = 5.0 Hz, 1H), 7.48 (d, J = 10.0 Hz, 2H), 7.42 (d, J = 5.0 Hz, 1H), 7.18 (d, J = 10.0 Hz, 2H), 6.94 (d, J = 5.0 Hz, 1H), 6.83 (d, J = 5.0 Hz, 1H), 4.71 (s, 2H), 2.86 (t, J = 5.0 Hz, 2H), 2.52 (t, J = 5.0 Hz, 2H), 1.71 (m, 4H), 1.42 (s, 9H).

^{19}F NMR (471 MHz, $CDCl_3$) δ -58.10.

^{13}C NMR (126 MHz, $CDCl_3$) δ 159.4, 157.9, 155.2, 146.6, 145.5, 143.9, 138.6, 136.4, 130.3, 128.8, 126.8, 122.0, 121.0, 120.9, 120.6 (q, J = 258.3 Hz), 120.2, 83.2, 51.0, 32.9, 28.3, 25.3, 22.5, 22.5.

HRMS (ESI, m/z) calcd for $C_{27}H_{28}F_3N_3O_4SNa^+$ $[M+Na]^+$: 570.1650, found: 570.1649.

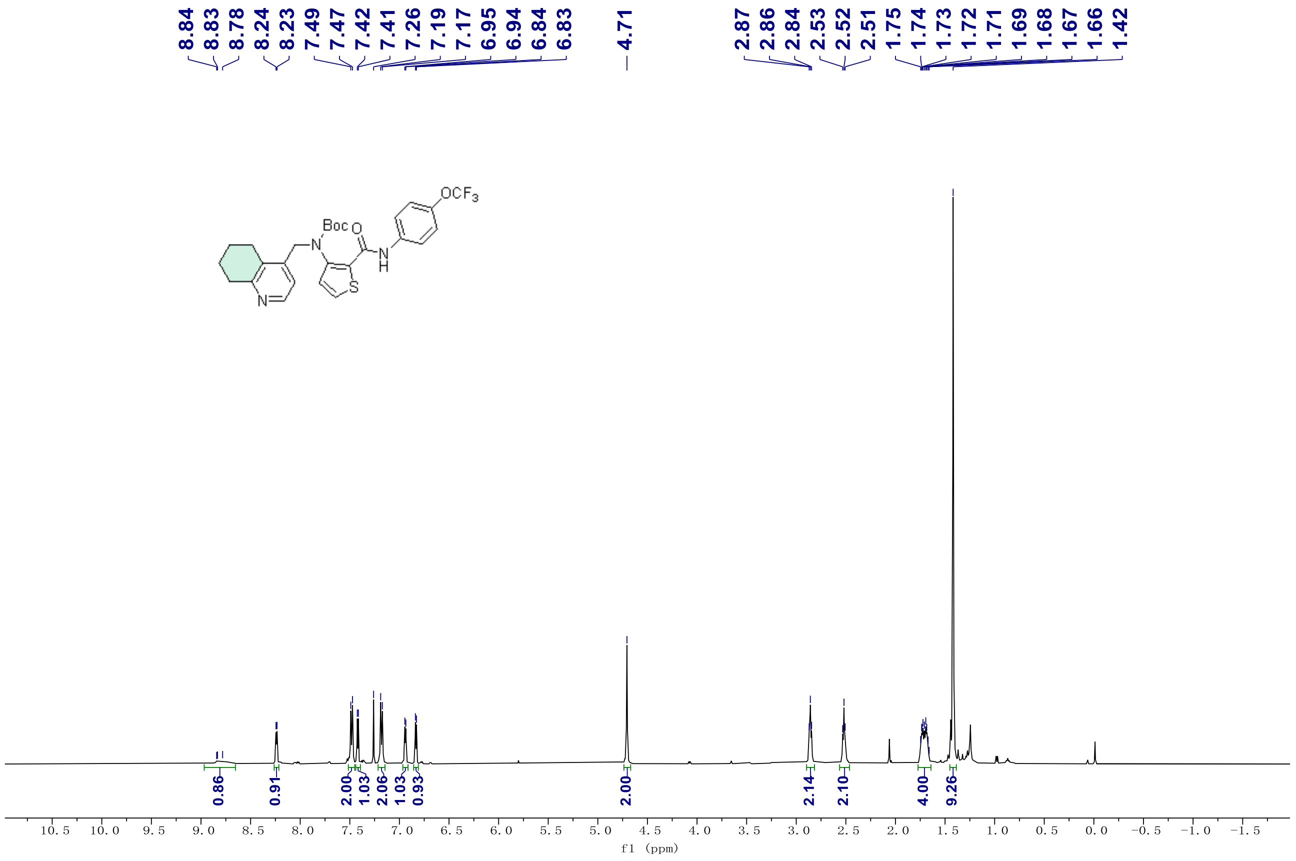
8.84
8.83
8.78
8.24
8.23
7.49
7.47
7.42
7.41
7.26
7.19
7.17
6.95
6.94
6.84
6.83
4.71
2.87
2.86
2.84
2.53
2.52
2.51
1.75
1.74
1.73
1.72
1.71
1.69
1.68
1.67
1.66
1.42
Boc
O
N
H
OCF3
S
N
0.86
0.91
2.00
1.03
2.06
1.03
0.93
2.00
2.14
2.10
4.00
9.26
f1 (ppm)

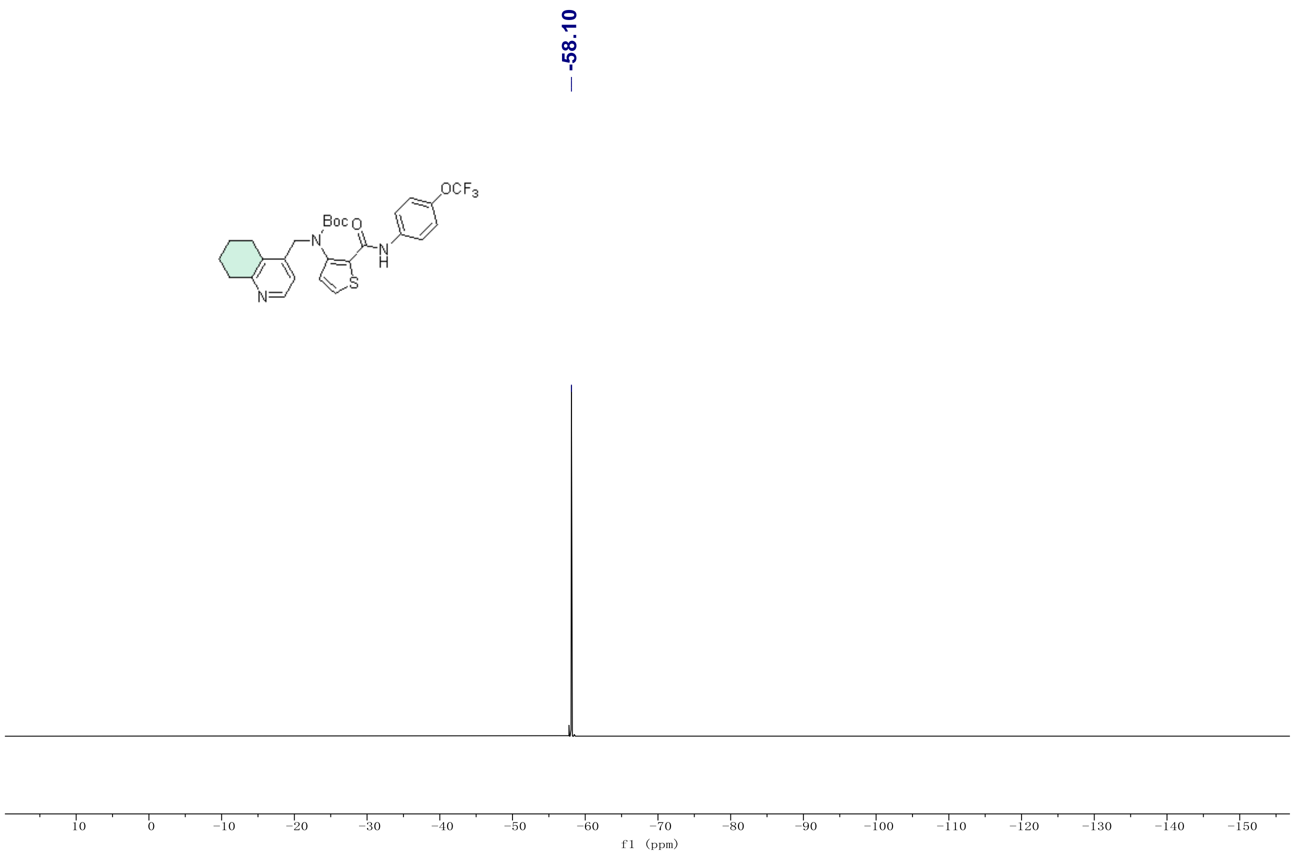
-58.10
Boc
O
N
H
OCF3
S
N
f1 (ppm)

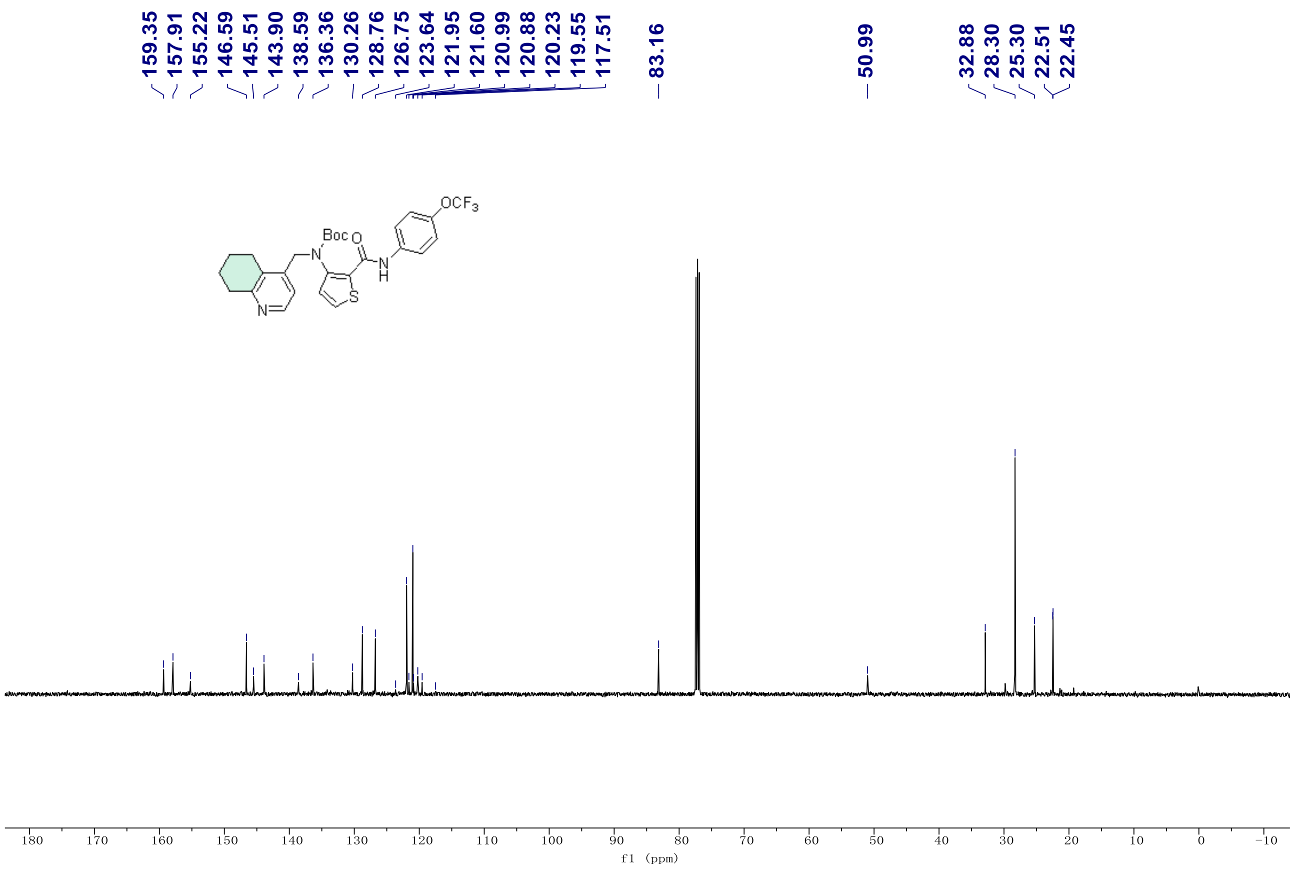

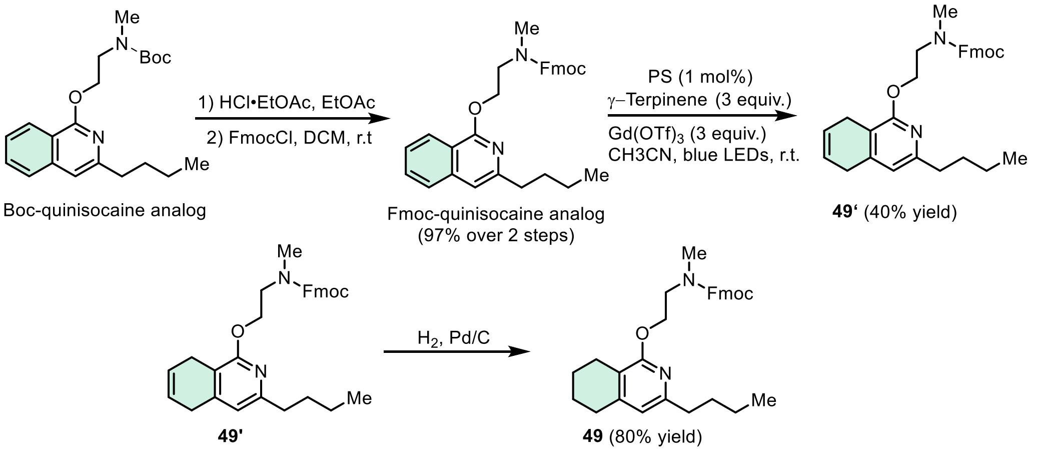

To a solution of **Boc-quinisocaine analog** (179.2 mg, 0.5 mmol, 1.0 equiv.), HCl (4.0 M in EtOAc) (0.25 mL, 1.0 mmol, 2 equiv.) in EtOAc (5.0 mL) was stirred for 2 hours at room temperature. After completion of reaction, the reaction was quenched by saturated aqueous $NaHCO_3$ and extracted with CH_2Cl_2 (10 mL × 3). The organic phases were combined and concentrated under reduced pressure. The crude residue was not purified and could be used directly for the next step. To a solution of the crude residue, 9-Fluorenylmethyl chloroformate (142.3 mg, 0.55 mmol, 1.1 equiv.), $NaHCO_3$ (84.0 mg, 1.0 mmol, 2.0 equiv.) in 1,4-dioxane/H_2O (*v/v* = 2:1, 3.0 mL) was stirred at the room temperature.

After the starting material was fully converted as checked by TLC, the reaction was quenched by saturated aqueous $NaHCO_3$ and extracted with CH_2Cl_2 (10 mL × 4). The organic phases were combined and concentrated under reduced pressure. The crude residue was purified by flash chromatography on silica gel to give the product **Fmoc-quinisocaine analog** as a colorless oil (233.1 mg, 97% yield over 2 steps).

Purification conditions: EtOAc/petroleum ether = 1:30 to 1:10

R_f = 0.3 in EtOAc/petroleum ether = 1:10.

Analytical data of **Fmoc-quinisocaine analog**:

^{1}H NMR (500 MHz, $CDCl_3$) δ 8.19 (t, *J* = 10.0 Hz, 1H), 7.75 (t, *J* = 7.5 Hz, 2H), 7.66 (d, *J* = 10.0 Hz, 1H), 7.64 - 7.59 (m, 2H), 7.55 (d, *J* = 10.0 Hz, 1H), 7.46 (q, *J* = 5.0 Hz, 1H), 7.38 (q, *J* = 5.0 Hz, 2H), 7.34 - 7.26 (m, 2H), 7.04 (d, *J* = 5.0 Hz, 1H), 4.72 (t, *J* = 5.0 Hz, 1H), 4.58 (t, *J* = 5.0 Hz, 1H), 4.46 (d, *J* = 5.0 Hz, 1H), 4.38 (d, *J* = 5.0 Hz, 1H), 4.17 (dt, *J* = 95.0, 5.0 Hz, 1H), 3.84 (t, *J* = 5.0 Hz, 1H), 3.74 (t, *J* = 10.0 Hz, 1H), 3.12 (d, *J* = 5.0 Hz, 3H), 2.79 (t, *J* = 7.5 Hz, 2H), 1.85 - 1.74 (m, 2H), 1.49 - 1.38 (m, 2H), 1.03 - 0.95 (m, 3H).

^{13}C NMR (126 MHz, $CDCl_3$, mixture of rotamers) δ 159.6, 159.4 (rotamer), 156.5, 156.4 (rotamer), 153.0, 152.9 (rotamer), 144.2, 141.4, 138.8, 130.4, 130.3 (rotamer), 127.7, 127.1, 125.8, 125.8 (rotamer), 125.7, 125.65 (rotamer), 125.1, 125.0, 124.0, 123.97 (rotamer), 120.0, 118.0, 112.2, 112.1 (rotamer), 67.5, 67.4 (rotamer), 64.2, 63.6 (rotamer), 48.7, 48.1 (rotamer), 47.5, 47.3 (rotamer), 37.6, 35.9, 35.8 (rotamer), 31.6, 31.57 (rotamer), 22.5, 14.2.

HRMS (ESI, *m/z*) calcd for $C_{31}H_{32}N_2O_3Na^+$ $[M+Na]^+$: 503.1214, found: 503.1211.

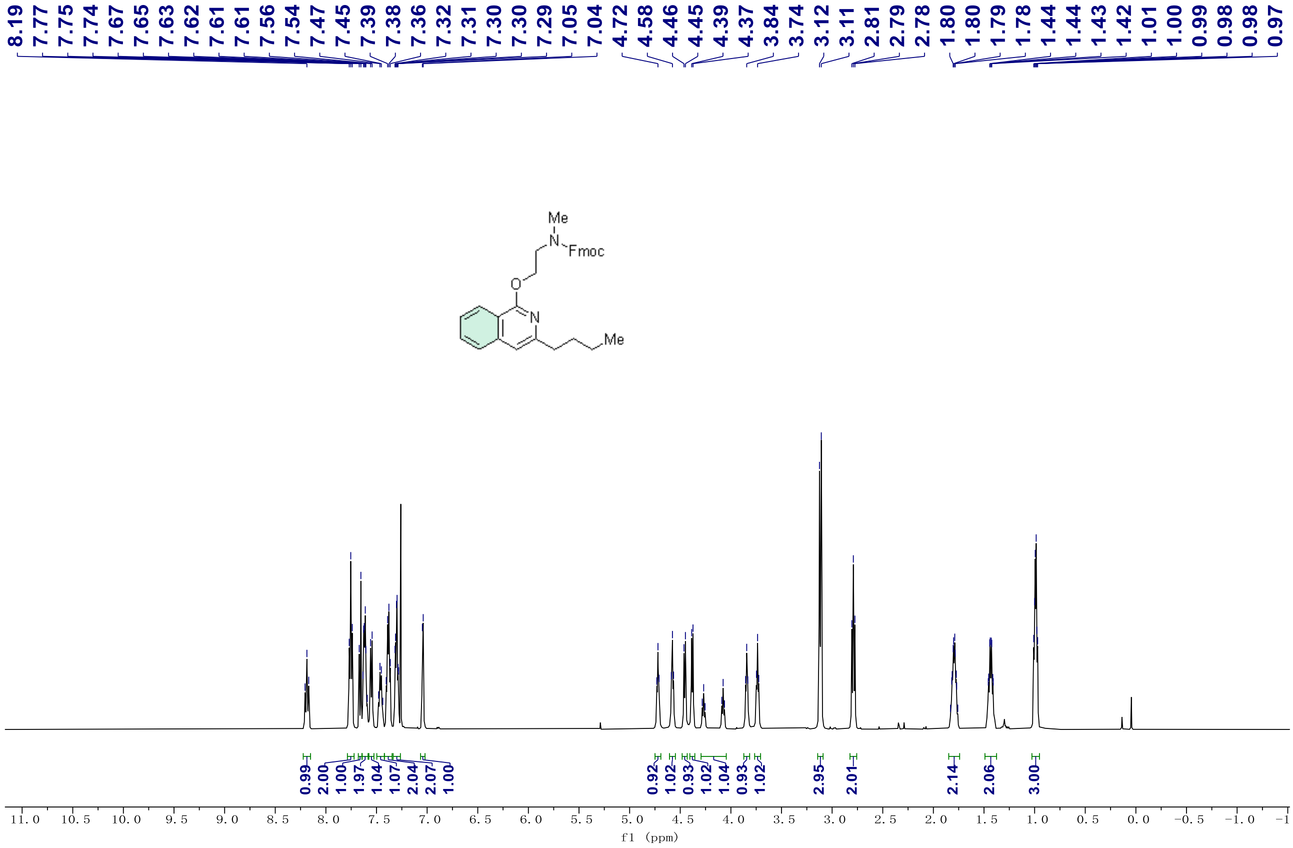
8.19 7.77 7.75 7.74 7.67 7.65 7.63 7.62 7.61 7.61 7.56 7.54 7.47 7.45 7.39 7.38 7.36 7.32 7.31 7.30 7.30 7.29 7.05 7.04 4.72 4.58 4.46 4.45 4.39 4.37 3.84 3.74 3.12 3.11 2.81 2.79 2.78 1.80 1.80 1.79 1.78 1.44 1.44 1.43 1.42 1.01 1.00 0.99 0.98 0.98 0.97
Me
N
Fmoc
O
N
Me
0.99 2.00 1.00 1.97 1.04 1.07 2.04 2.07 1.00
0.92 1.02 0.93 1.02 1.04 0.93 1.02
2.95 2.01 2.14 2.06 3.00
11.0 10.5 10.0 9.5 9.0 8.5 8.0 7.5 7.0 6.5 6.0 5.5 5.0 4.5 4.0 3.5 3.0 2.5 2.0 1.5 1.0 0.5 0.0 −0.5 −1.0
f1 (ppm)

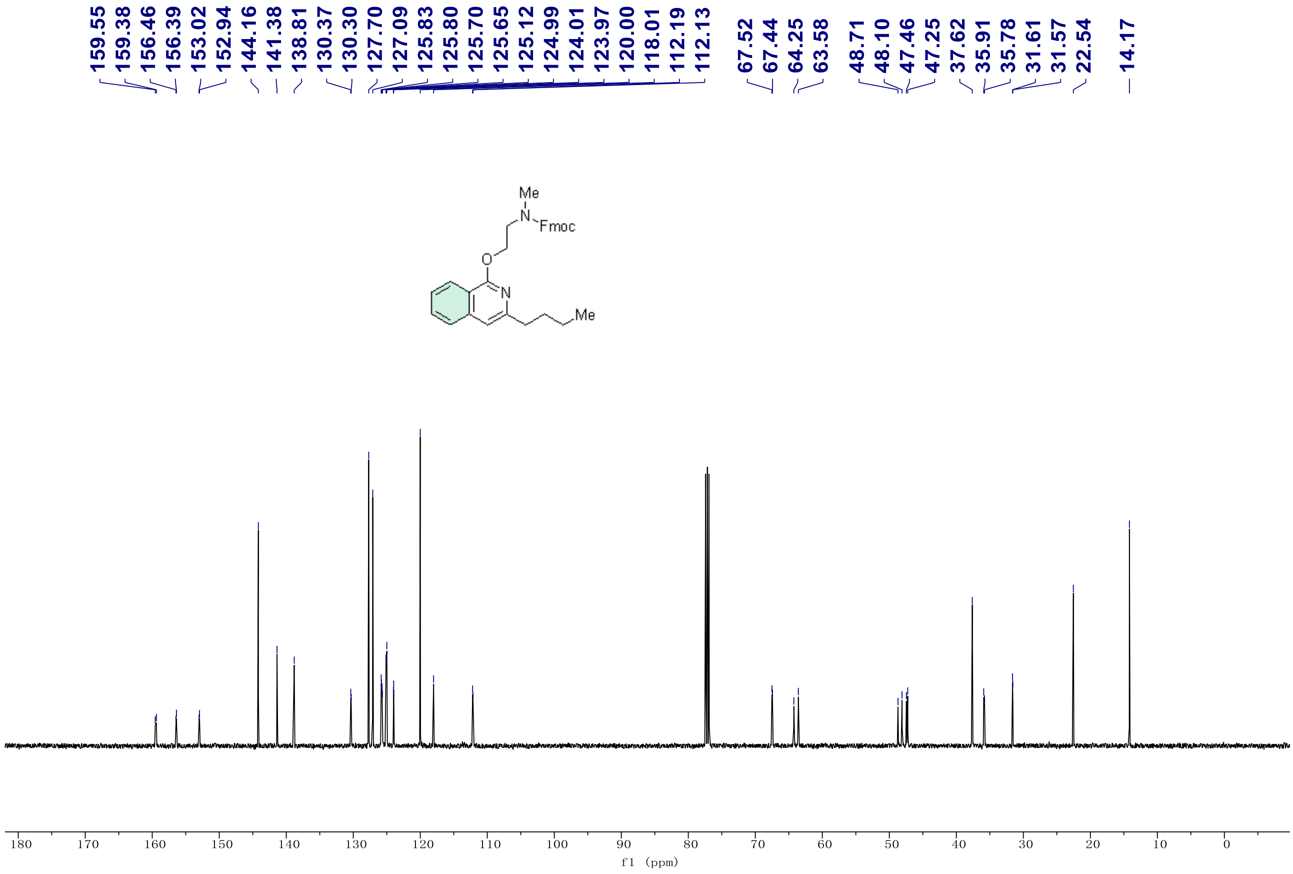
159.55 159.38 156.46 156.39 153.02 152.94 144.16 141.38 138.81 130.37 130.30 127.70 127.09 125.83 125.80 125.70 125.65 125.12 124.99 124.01 123.97 120.00 118.01 112.19 112.13 67.52 67.44 64.25 63.58 48.71 48.10 47.46 47.25 37.62 35.91 35.78 31.61 31.57 22.54 14.17
Me
N
Fmoc
O
N
Me
180 170 160 150 140 130 120 110 100 90 80 70 60 50 40 30 20 10 0
f1 (ppm)

A dried 10 mL Schlenk tube was charged with the **Fmoc-quinisocaine analog** (0.2 mmol, 96.1 mg, 1.0 equiv.), Ir[dF(Me)ppy]$_2$(dtbbpy)PF$_6$ (0.002 mmol, 1.0 mol%, 2.2 mg), Gd(OTf)$_3$ (0.4 mmol, 2.0 equiv., 242 mg), *γ*-terpinene (0.6 mmol, 3.0 equiv., 96 μL) and CH_3CN (4.0 mL, 0.05 M). The mixture was degassed by three cycles of freeze-pump-thaw, subsequently irradiated with 10 W blue LEDs (λ_{max} =455 nm) for 4 hours under nitrogen at 20 °C. After the starting material was fully converted as checked by TLC, the reaction was quenched by saturated aqueous $NaHCO_3$ and extracted with CH_2Cl_2 (10 mL × 4). The organic phases were combined and concentrated under reduced pressure. The crude residue was purified by flash chromatography on silica gel to give the desired product **49'** as a colorless oil (38.6 mg, 40% yield).

Purification conditions: EtOAc/petroleum ether = 1:4 to 1:1

R_f (**49'**) = 0.4 in EtOAc/petroleum ether = 1:1.

Analytical data of **49'**:

^{1}H NMR (400 MHz, $CDCl_3$, mixture of rotamers) δ 7.79 - 7.69 (m, 2H, major; m, 2H, minor), 7.58 (dd, *J* = 12.0, 8.0 Hz, 2H, major; dd, *J* = 12.0, 8.0 Hz, 2H, minor), 7.38 (t, *J* = 8.0 Hz, 2H, major; t, *J* = 8.0 Hz, 2H, minor), 7.33 - 7.25 (m, 2H, major; m, 2H, minor), 6.46 (s, 1H), 6.43 (s, 1H, minor), 5.91 (br, 1H), 5.82 (br, 1H), 4.49 (t, *J* = 8.0 Hz, 1H), 4.44 - 4.32 (m, 3H, major; m, 3H, minor), 4.29 - 4.10 (m, 1H, major; m, 1H, minor), 3.69 (t, *J* = 4.0 Hz, 1H; major; t, *J* = 4.0 Hz, 1H; minor), 3.62 (t, *J* = 4.0 Hz, 1H, major; t, *J* = 4.0 Hz, 1H; minor), 3.32 - 3.23 (m, 2H), 3.19 - 3.09 (m, 2H), 3.04 (m, 3H, major; m, 3H, minor), 2.62 - 2.57 (m, 2H, major; m, 2H, minor), 1.69 - 1.61 (m, 2H, major; m, 2H, minor), 1.45 - 1.29 (m, 2H, major; m, 2H, minor), 1.02 - 0.87 (m, 3H, major; m, 3H, minor).

^{13}C NMR (101 MHz, $CDCl_3$, mixture of rotamers) δ 156.5, 156.4 (rotamer), 144.3, 141.5, 127.7, 127.1, 125.2, 125.1 (rotamer), 124.9, 123.2, 120.1, 115.6, 115.5 (rotamer), 114.1, 114.0 (rotamer), 67.6, 67.4 (rotamer), 63.8 (rotamer), 63.3, 48.9, 48.3 (rotamer), 47.6, 47.4 (rotamer), 37.3, 35.9, 35.8 (rotamer), 31.8, 29.1, 23.8, 22.6, 14.2.

HRMS (ESI, *m/z*) calcd for $C_{31}H_{34}N_2O_3Na^+$ $[M+Na]^+$: 505.2470, found: 505.2467.

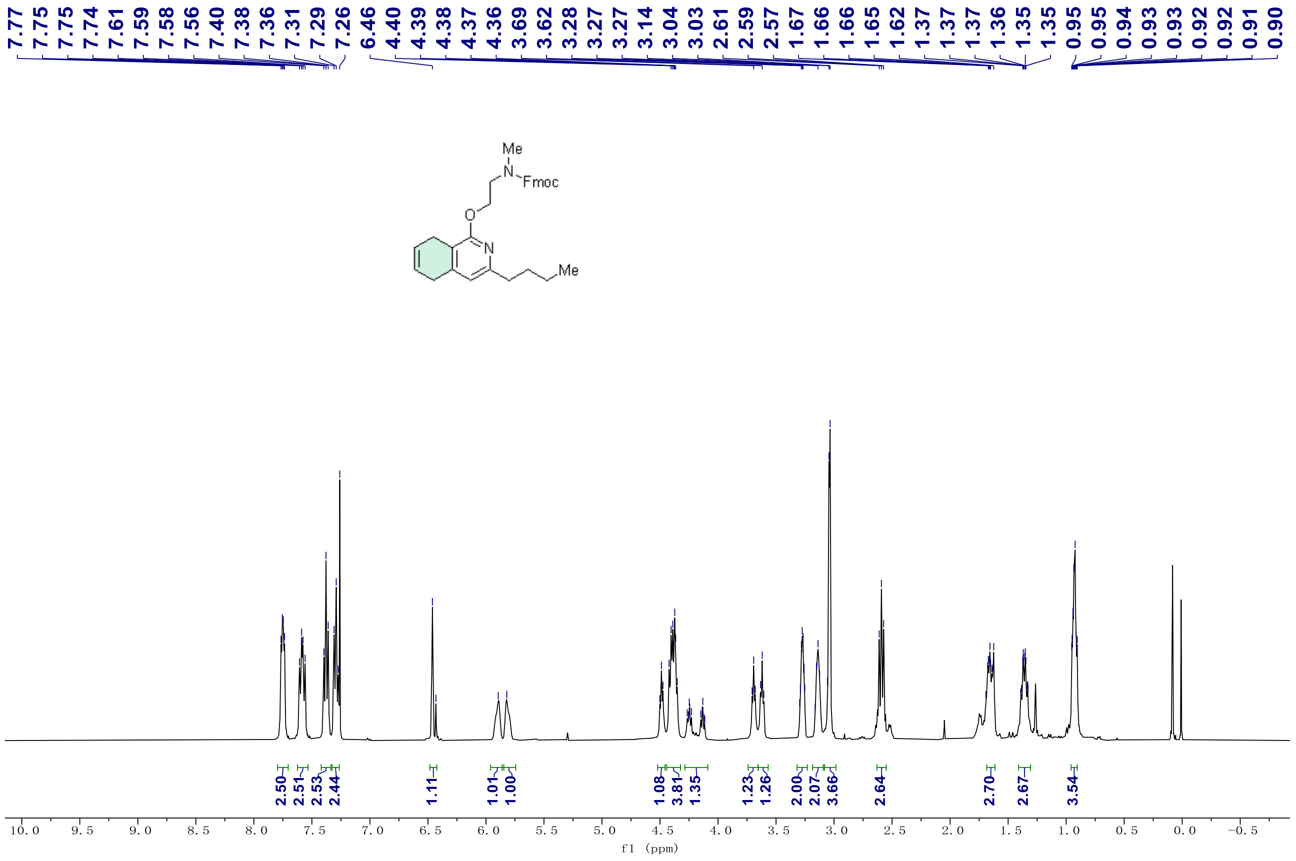
7.77 7.75 7.75 7.74 7.61 7.59 7.58 7.56 7.40 7.38 7.36 7.31 7.29 7.26 6.46 4.40 4.39 4.38 4.37 4.36 3.69 3.62 3.28 3.27 3.27 3.14 3.04 3.03 2.61 2.59 2.57 1.67 1.66 1.66 1.65 1.62 1.37 1.37 1.37 1.36 1.35 1.35 0.95 0.95 0.94 0.93 0.93 0.92 0.92 0.91 0.90
Me
N
Fmoc
O
N
Me
2.50 2.51 2.53 2.44 1.11 1.01 1.00 1.08 3.81 1.35 1.23 1.26 2.00 2.07 3.66 2.64 2.70 2.67 3.54
10.0 9.5 9.0 8.5 8.0 7.5 7.0 6.5 6.0 5.5 5.0 4.5 4.0 3.5 3.0 2.5 2.0 1.5 1.0 0.5 0.0 -0.5
f1 (ppm)

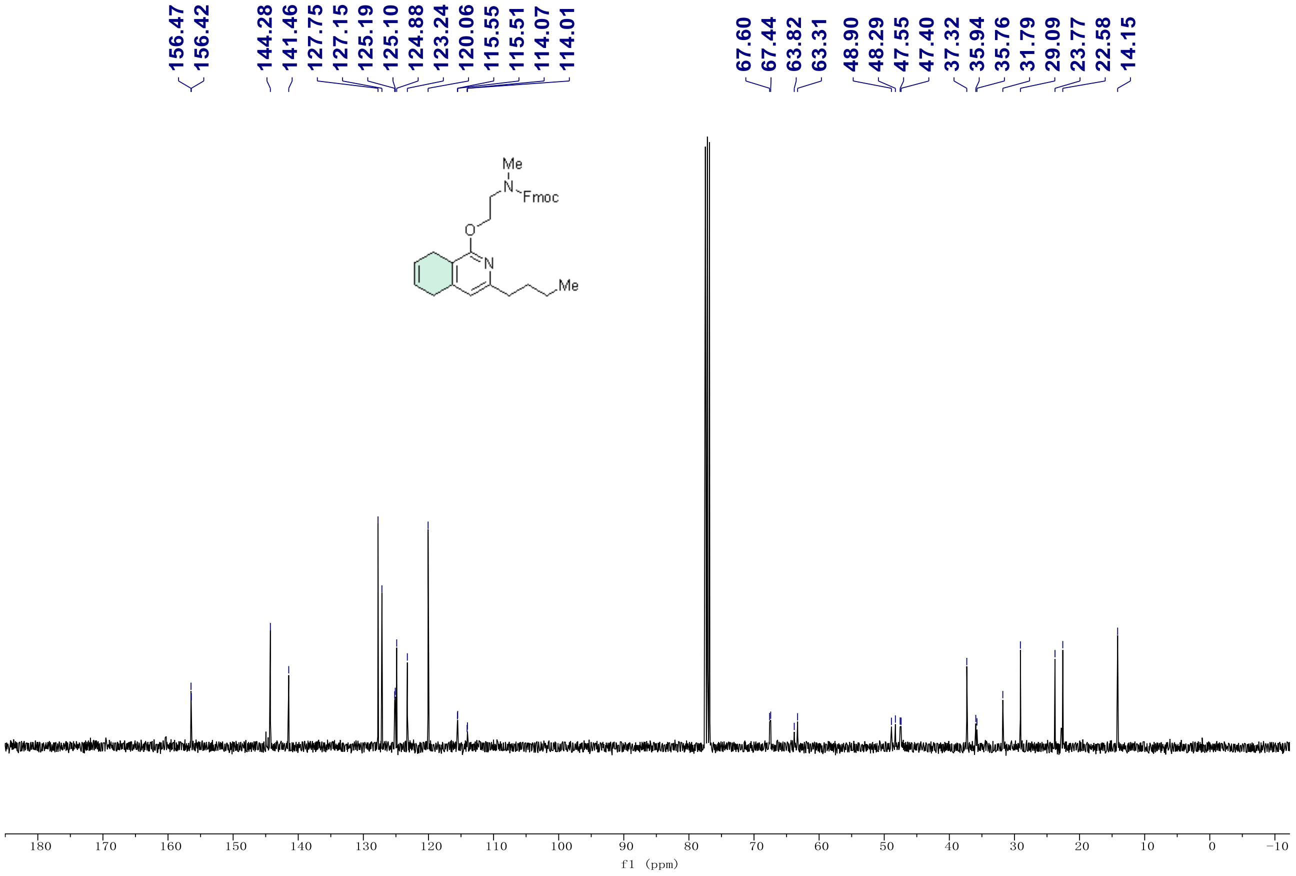
156.47 156.42 144.28 141.46 127.75 127.15 125.19 125.10 124.88 123.24 120.06 115.55 115.51 114.07 114.01 67.60 67.44 63.82 63.31 48.90 48.29 47.55 47.40 37.32 35.94 35.76 31.79 29.09 23.77 22.58 14.15
Me
N
Fmoc
O
N
Me
180 170 160 150 140 130 120 110 100 90 80 70 60 50 40 30 20 10 0 -10
f1 (ppm)

To a solution of **49’** (30.0 mg, 0.062 mmol, 1.0 equiv.) and Pd/C (Pd 5% on Carbon wetted with ca. 55% water, 6.0 mg) in MeOH (2.0 mL) was stirred for 6 hours under hydrogen atmosphere at room temperature. After completion of reaction, the resulting mixture was filtered by diatomaceous earth and washed with EtOAc (3 × 3mL). The organic phases were combined and concentrated under reduced pressure. The crude residue was purified by flash chromatography on silica gel to give the product **49** as a colorless oil (26.5 mg, 88% yield).

Purification conditions: EtOAc/petroleum ether = 1:5 to 1:2

R_f (**49**) = 0.4 in EtOAc/petroleum ether = 1:1.

Analytical data of **49**:

^{1}H NMR (400 MHz, $CDCl_3$) δ 7.76 (d, *J* = 8.0 Hz, 2H), 7.60 (t, *J* = 8.0 Hz, 2H), 7.39 (t, *J* = 8.0 Hz, 2H), 7.30 (t, *J* = 8.0 Hz, 2H), 6.44 (s, 1H), 4.48 (t, *J* = 4.4 Hz, 1H), 4.44 - 4.32 (m, 3H), 4.23 (dt, *J* = 28.0, 8.0 Hz, 1H), 3.66 (dt, *J* = 28.0, 4.0 Hz, 2H), 3.05 (d, *J* = 8.0 Hz, 3H), 2.67 - 2.50 (m, 6H), 1.80 - 1.61 (m, 6H), 1.37 (h, *J* = 8.0 Hz, 2H), 0.93 (t, *J* = 8.0 Hz, 3H).

^{13}C NMR (101 MHz, $CDCl_3$, mixture of rotamers) δ 160.9 (rotamer), 160.7, 156.5, 155.7, 148.1, 144.3, 141.4, 127.7, 127.1, 125.2 (rotamer), 125.1, 120.1, 116.6, 116.3 (rotamer), 67.6, 67.5 (rotamer), 63.8, 63.4 (rotamer), 48.9, 48.4 (rotamer), 47.5, 47.4 (rotamer), 37.3, 36.0, 35.8 (rotamer), 31.8, 29.1, 22.8, 22.7, 22.6, 22.5, 14.2.

HRMS (ESI, *m*/*z*) calcd for $C_{31}H_{36}N_2O_3Na^+$ $[M+ Na]^+$: 507.2628, found: 507.2624.

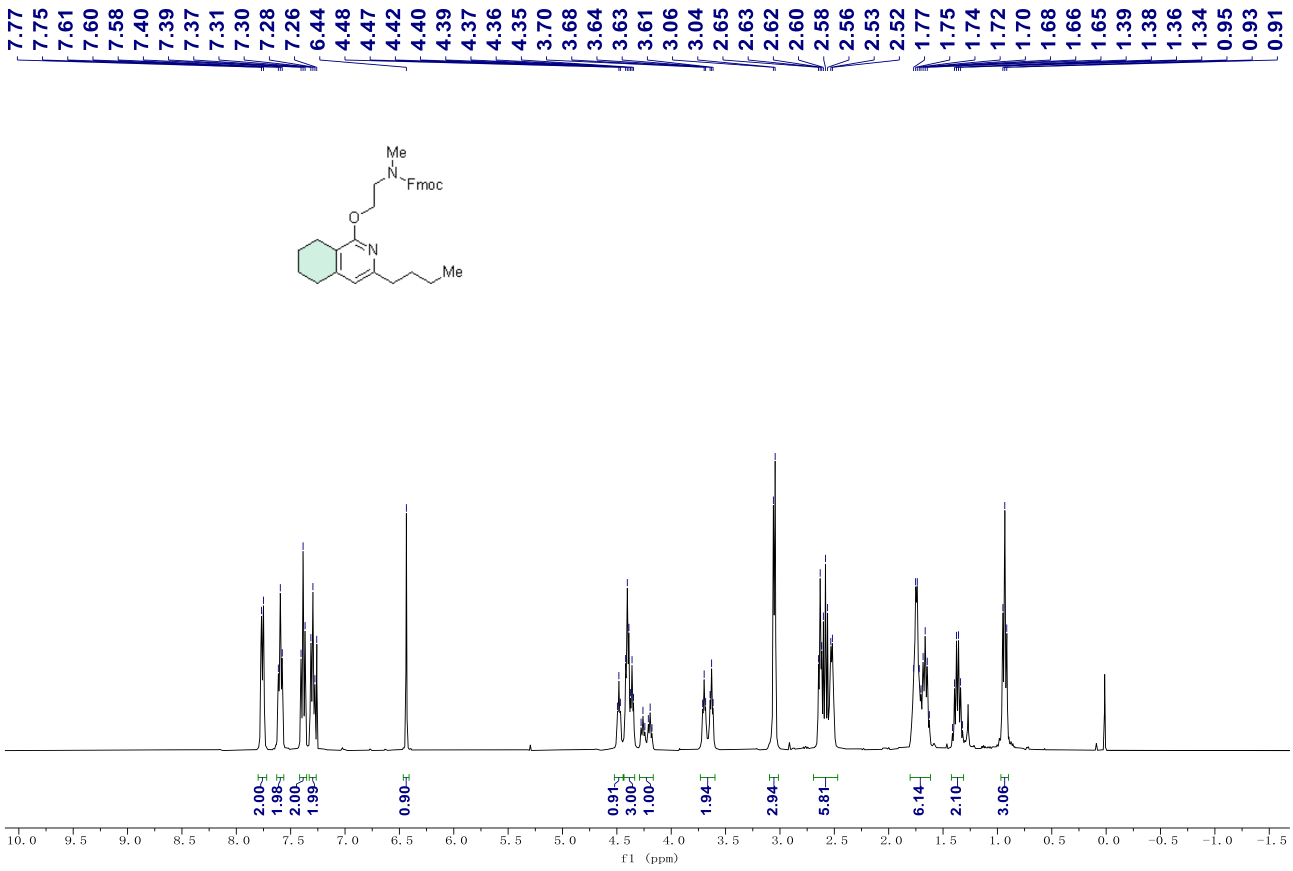
7.77 7.75 7.61 7.60 7.58 7.40 7.39 7.37 7.31 7.30 7.28 7.26 6.44 4.48 4.47 4.42 4.40 4.39 4.37 4.36 4.35 3.70 3.68 3.64 3.63 3.61 3.06 3.04 2.65 2.63 2.62 2.60 2.58 2.56 2.53 2.52 1.77 1.75 1.74 1.72 1.70 1.68 1.66 1.65 1.39 1.38 1.36 1.34 0.95 0.93 0.91
Me
N
Fmoc
O
N
Me
2.00 1.98 2.00 1.99 0.90 0.91 3.00 1.00 1.94 2.94 5.81 6.14 2.10 3.06
f1 (ppm)

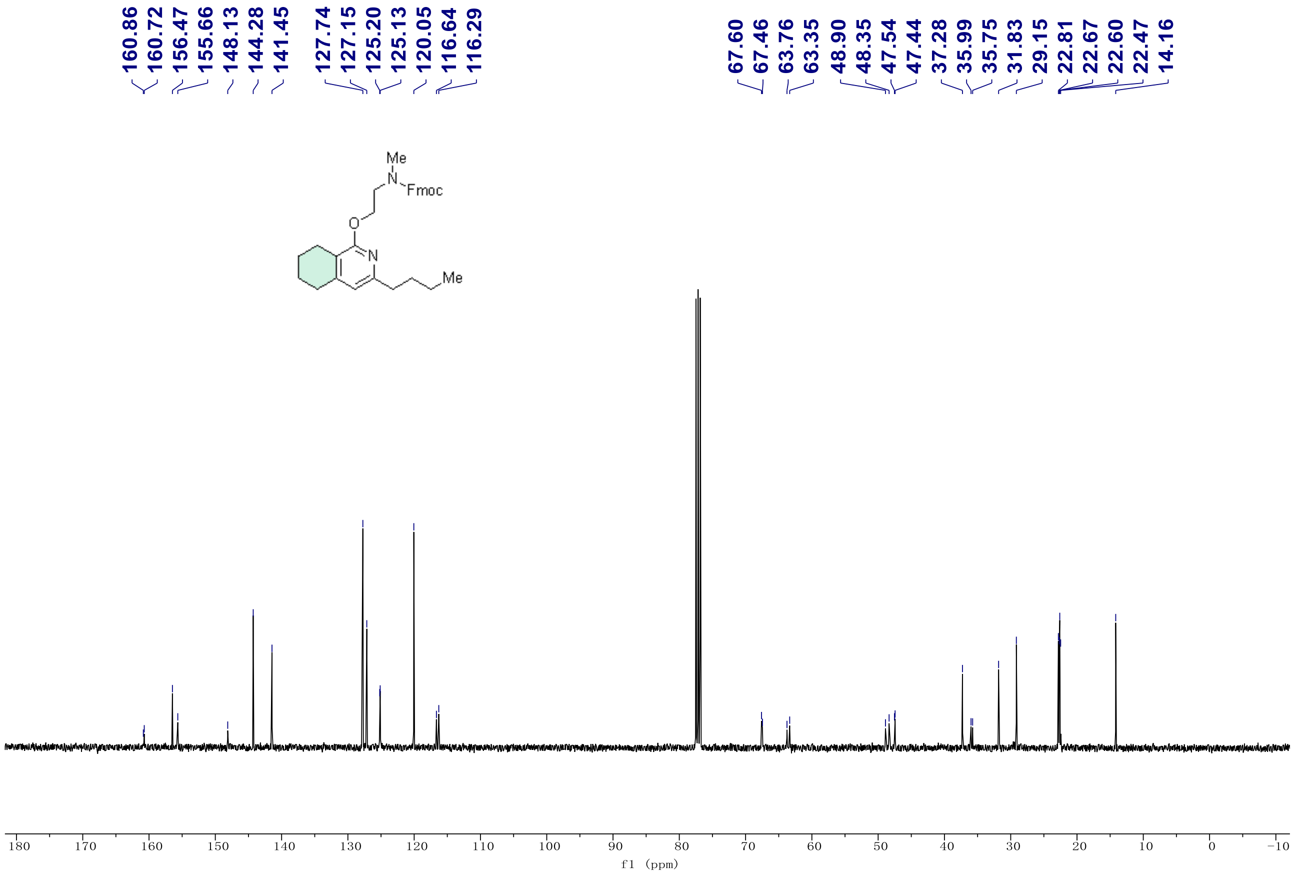
160.86 160.72 156.47 155.66 148.13 144.28 141.45 127.74 127.15 125.20 125.13 120.05 116.64 116.29
67.60 67.46 63.76 63.35 48.90 48.35 47.54 47.44 37.28 35.99 35.75 31.83 29.15 22.81 22.67 22.60 22.47 14.16
Me
N
Fmoc
O
N
Me
f1 (ppm)

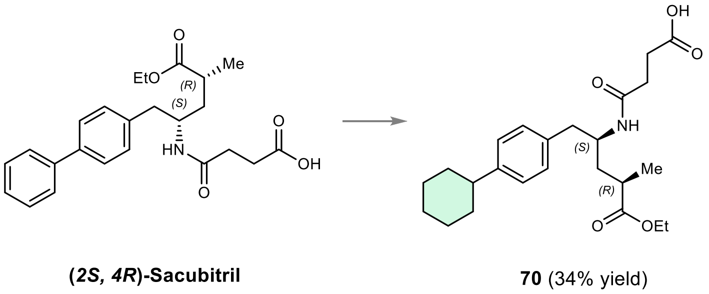

According to the general procedure, a mixture of **(2*S*, 4*R*)-Sacubitril** (82.3 mg, 0.20 mmol, 1.0 equiv.), $B_2(OH)_4$ (107.5 mg, 1.20 mmol, 6.0 equiv.) and $[Rh(COD)OH]_2$ (3.7 mg, 4.0 mol%, 0.008 mmol) in EtOH (1.0 mL, 0.2 M) was stirred under nitrogen atmosphere fo 48 hours at 80 °C to afford **70** as a white solid (28.5 mg, 34% yield).

Purification conditions: Reverse-phase column chromatography (MeCN/H_2O = 1:5 to 1.5:1) on SepaBean machine (Santai Technology Inc., China) equipped with C18-bonded SepaFlash columns.

R_f= 0.4 in petroleum ether/acetone = 4:1 (the mixture of product and byproducts).

^{1}H NMR (400 MHz, $CDCl_3$) δ 7.11 (d, *J* = 8.0 Hz, 2H), 7.06 (d, *J* = 8.0 Hz, 2H), 5.93 - 5.83 (m, 1H), 4.24 - 4.14 (m, 1H), 4.10 (q, *J* = 7.2 Hz, 2H), 2.75 (d, *J* = 6.4 Hz, 2H), 2.64 - 2.57 (m, 2H), 2.56 - 2.38 (m, 4H), 1.95 - 1.78 (m, 5H), 1.73 (d, *J* = 12.4 Hz, 1H), 1.53 - 1.42 (m, 1H), 1.42 - 1.31 (m, 4H), 1.21 (t, *J* = 7.2 Hz, 4H), 1.13 (d, *J* = 7.2 Hz, 3H).

^{13}C NMR (100 MHz, $CDCl_3$) δ 176.6, 176.4, 172.0, 146.5, 134.7, 129.5, 127.0, 60.7, 48.9, 44.3, 40.5, 37.4, 36.6, 34.6, 31.0, 30.0, 27.0, 26.3, 17.7, 14.3.

HRMS (ESI, *m*/*z*) calcd for $C_{24}H_{35}NO_5Na^+$ $[M+ Na]^+$: 440.2413, found: 440.2418.

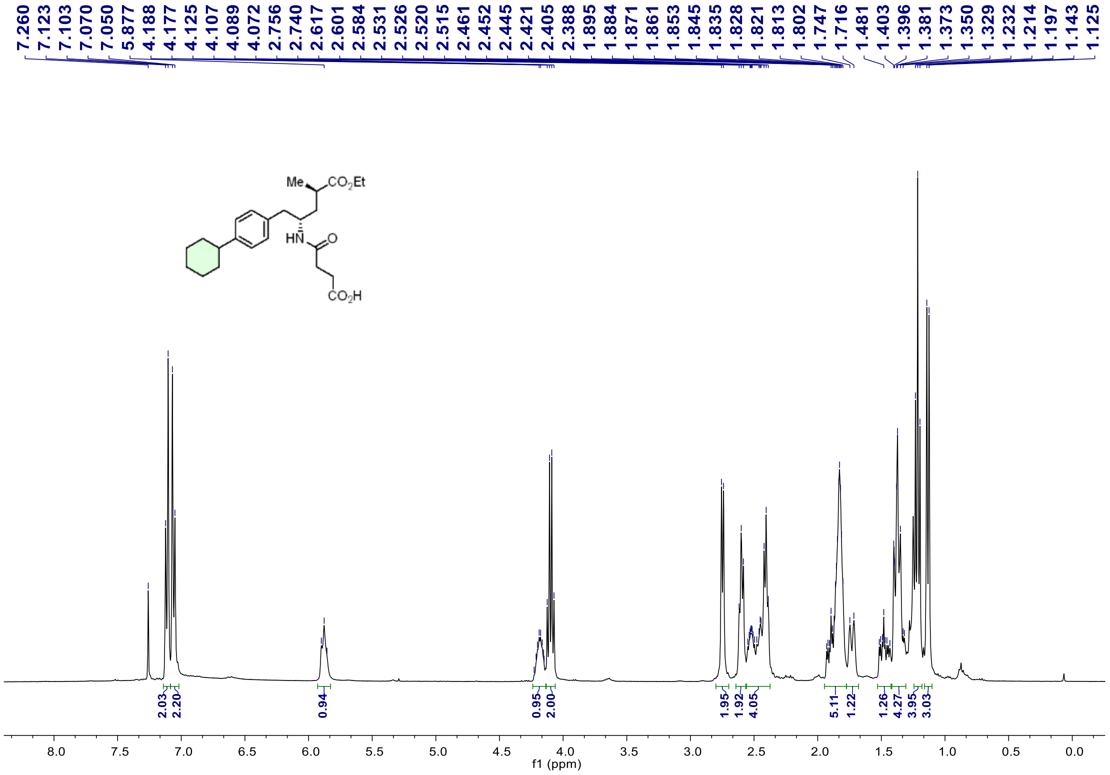
Me
CO2Et
HN
O
CO2H
f1 (ppm)

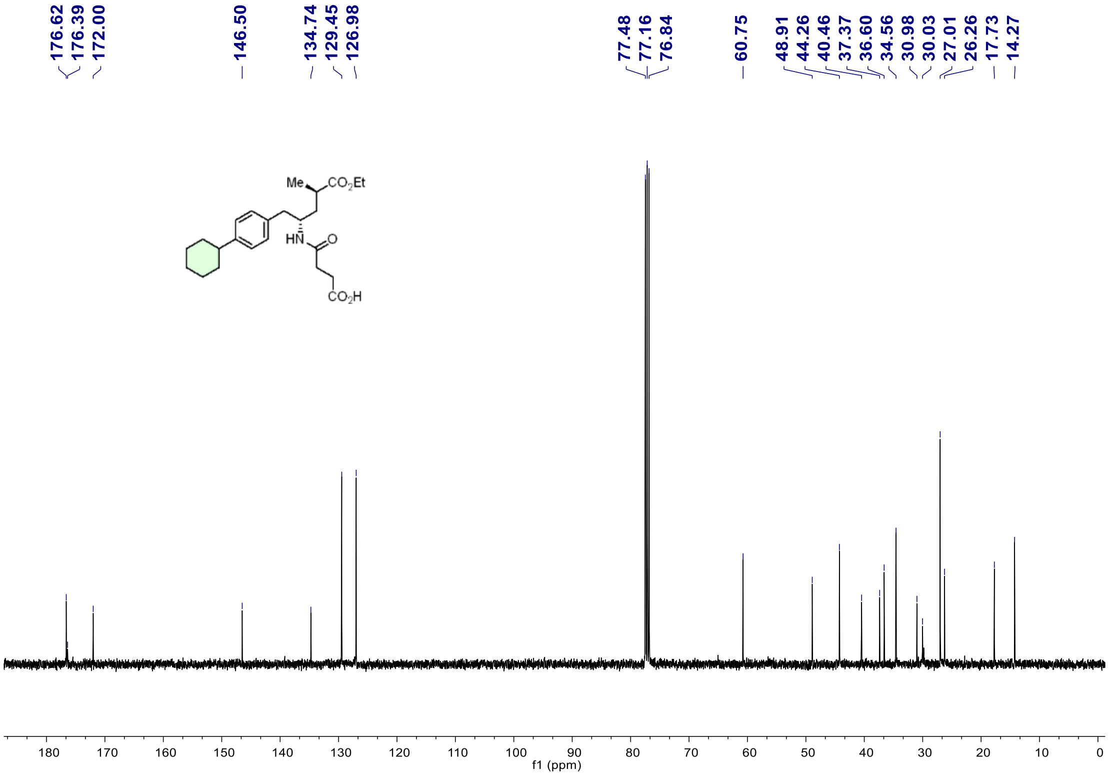
Me
CO2Et
HN
O
CO2H
f1 (ppm)

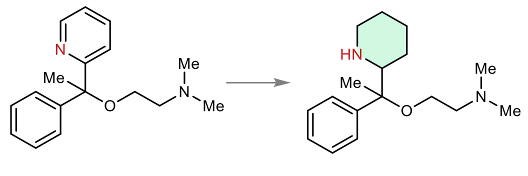

Doxylamine **71** (60% yield)

According to the general procedure, a mixture of **doxylamine** (54.0 mg, 0.20 mmol, 1.0 equiv.), $B_2(OH)_4$ (107.5 mg, 1.20 mmol, 6.0 equiv.) and $[Rh(COD)OH]_2$ (3.7 mg, 4.0 mol%, 0.008 mmol) in EtOH (1.0 mL, 0.2 M) was stirred under nitrogen atmosphere for 48 hours at 80 °C to afford **71** as a yellow oil (33.2 mg, 60% yield, major diastereoisomer).

Purification conditions: Reverse-phase column chromatography (MeOH/H_2O = 1:9 to 1.5:1) on SepaBean machine (Santai Tech-nology Inc., China) equipped with C18-bonded SepaFlash columns. R_f = 0.2 in NH_3(7.0 M solution in MeOH)/CH_2Cl_2 = 1:10.

^{1}H NMR (400 MHz, $CDCl_3$) δ 7.32 - 7.24 (m, 4H), 7.21 - 7.16 (m, 1H), 3.34 (dt, J = 9.6, 6.4 Hz, 1H), 3.07 (dt, J = 9.6, 6.0 Hz, 1H), 2.94 - 2.89 (m, 1H), 2.52 (dd, J = 11.2, 2.0 Hz, 1H), 2.48 - 2.35 (m, 3H), 2.17 (s, 6H), 1.69 (d, J = 10.0 Hz, 2H), 1.54 (s, 3H), 1.45 - 1.41 (m, 1H), 1.32 - 1.13 (m, 2H), 1.12 - 1.01 (m, 1H).

^{13}C NMR (100 MHz, $CDCl_3$) δ 143.4, 128.2, 127.2, 127.1, 80.9, 66.9, 60.5, 59.6, 47.6, 46.0, 26.9, 26.2, 25.0, 18.9.

HRMS (ESI, m/z) calcd for $C_{17}H_{29}N_2O^+$ $[M+H]^+$: 277.2280, found: 277.2281.

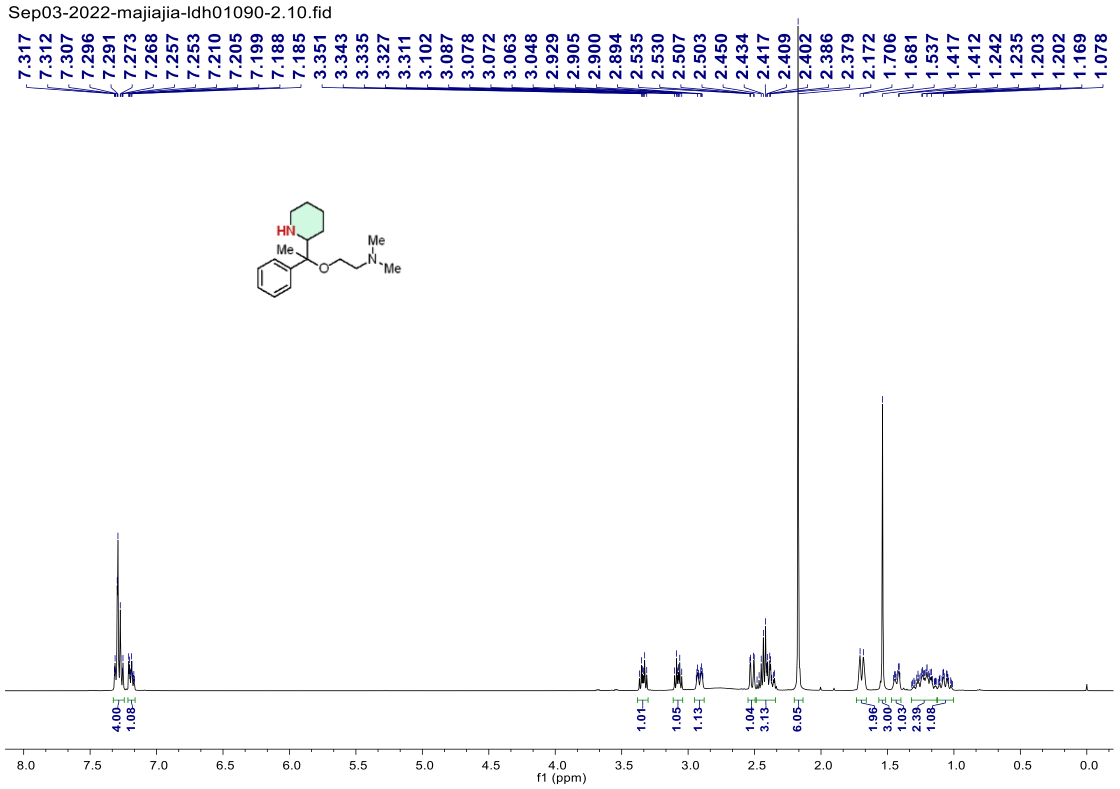

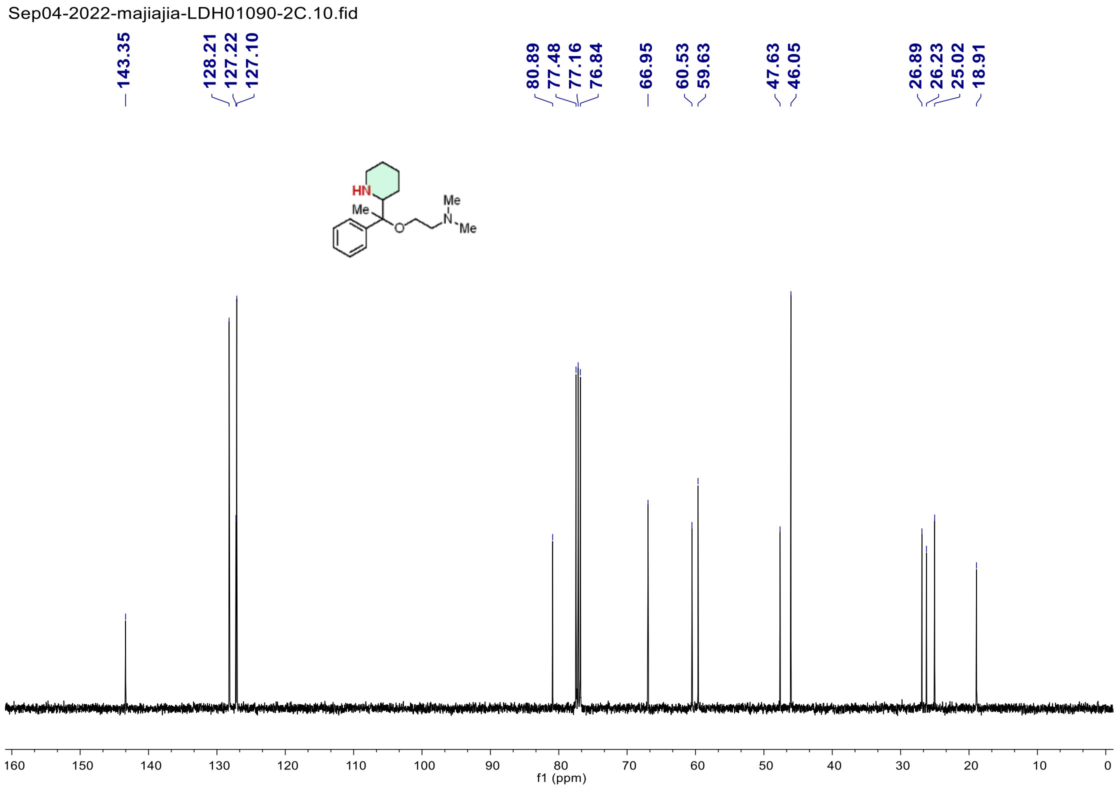

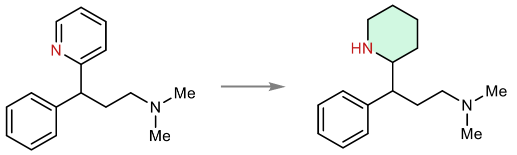

Pheniramine **72** (54% yield)

According to the general procedure, a mixture of **pheniramine** (48.0 mg, 0.2 mmol, 1.0 equiv.), $B_2(OH)_4$ (107.5 mg, 1.2 mmol, 6.0 equiv.) and $[Rh(COD)OH]_2$ (3.7 mg, 4 mol%, 0.008 mmol) in EtOH (1.0 mL, 0.2 M) was stirred under nitrogen atmosphere for 36 hours at 50 °C to afford **72** (26.7 mg, 54% yield, major diastereoisomer) as colorless oil.

^{1}H NMR (400 MHz, $CDCl_3$) δ 7.32 - 7.28 (m, 2H), 7.23 - 7.19 (m, 3H), 2.97 - 2.94 (m, 1H), 2.67 - 2.58 (m, 2H), 2.50 (td, J = 11.8, 2.9 Hz, 1H), 2.21 - 2.14 (m, 7H), 2.07 - 1.99 (m, 1H), 1.97 - 1.91 (m, 2H), 1.84 - 1.81 (m, 1H), 1.73 - 1.64 (m, 1H), 1.59 - 1.56 (m, 1H), 1.45 - 1.25 (m, 4H).

^{13}C NMR (101 MHz, $CDCl_3$) δ 142.5, 128.7, 128.5, 126.8, 61.5, 58.1, 50.2, 47.0, 45.3, 29.7, 29.5, 25.9, 24.8.

HRMS (ESI, *m/z*) calcd for $C_{16}H_{27}N_2^+$ $[M+H]^+$: 247.2174, found: 247.2171.

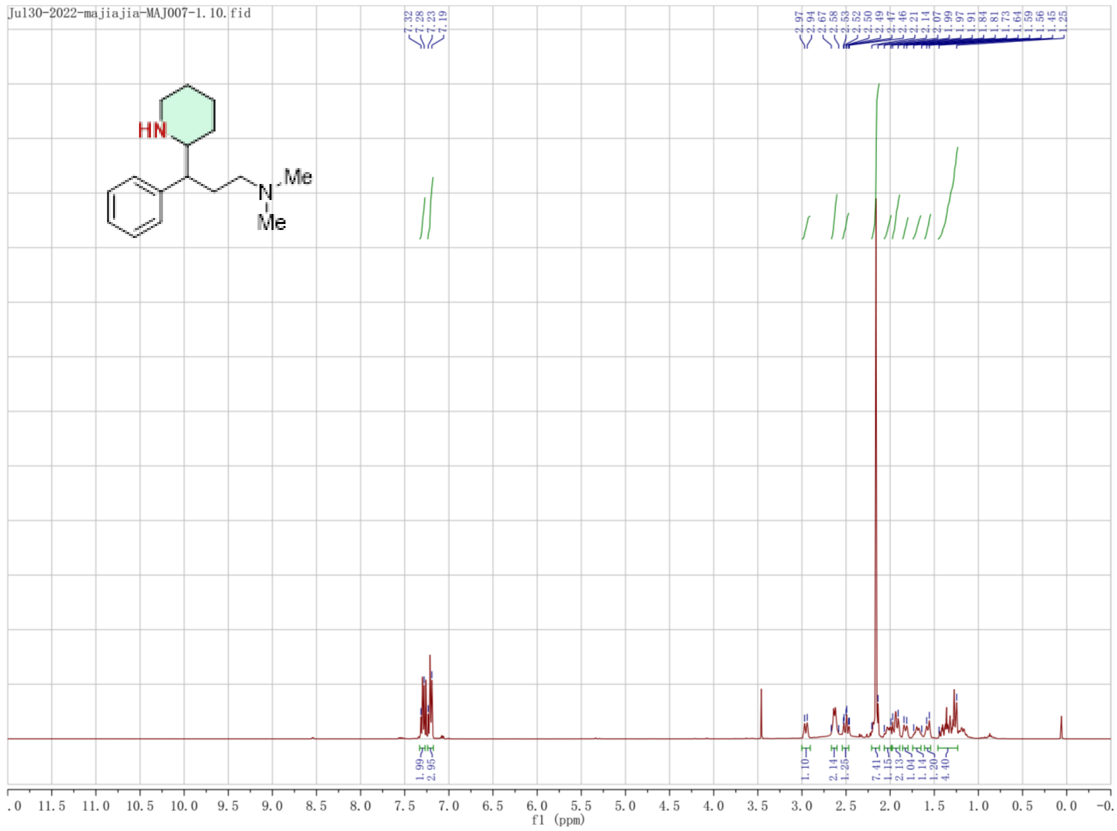
Jul30-2022-majiajia-MAJ007-1.10.fid
7.32
7.28
7.23
7.19
2.97
2.94
2.67
2.58
2.53
2.52
2.50
2.49
2.47
2.46
2.21
2.14
2.07
1.99
1.97
1.91
1.84
1.81
1.73
1.64
1.59
1.56
1.45
1.25
HN
Me
N
Me
1.99
2.95
1.10
2.14
1.25
7.41
1.15
2.13
1.04
1.14
1.20
4.40
f1 (ppm)

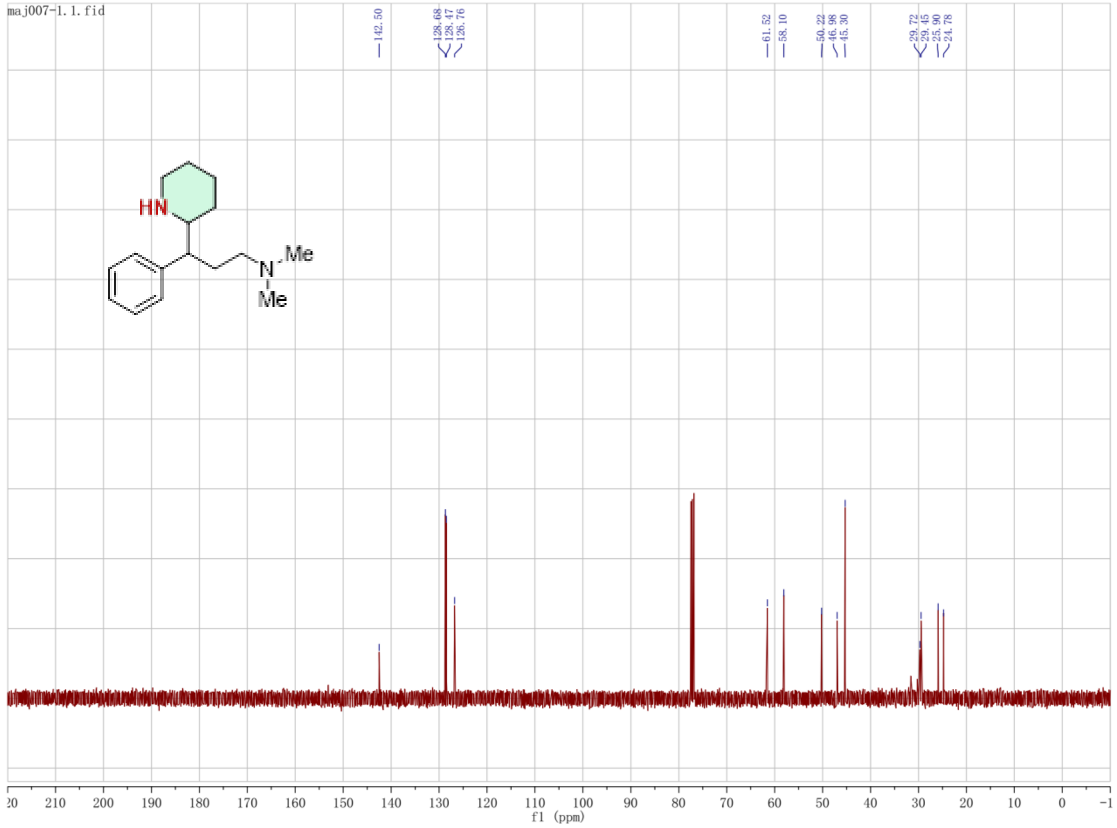
maj007-1.1.fid
142.50
128.68
128.47
126.76
61.52
58.10
50.22
46.98
45.30
29.72
29.45
25.90
24.78
HN
Me
N
Me
f1 (ppm)

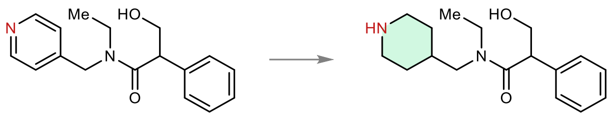

(±)-Tropicamide **73** (67% yield)

According to the general procedure, a mixture of **(±)-tropicamide** (56.9 mg, 0.20 mmol, 1.0 equiv.), $B_2(OH)_4$ (107.5 mg, 1.20 mmol, 6.0 equiv.) and $[Rh(COD)OH]_2$ (3.7 mg, 4.0 mol%, 0.008 mmol) in EtOH (1.0 mL, 0.2 M) was stirred under nitrogen atmosphere for 48 hours at 80 °C to afford **73** as a colorless oil (39.1 mg, 67% yield).

Purification conditions: Reverse-phase column chromatography (MeOH/H_2O = 1:9 to 1.5:1) on SepaBean machine (Santai Tech-nology Inc., China) equipped with C18-bonded SepaFlash columns.

R_f = 0.2 in NH_3(7.0 M solution in MeOH)/CH_2Cl_2 = 1:6.

^{1}H NMR (400 MHz, $CDCl_3$, mixture of rotamer) δ 7.32 - 7.27 (m, 2H, major; m, 2H, minor), 7.25 - 7.18 (m, 3H, major; m, 3H, minor), 4.06 - 3.96 (m, 1H, major; m, 1H, minor), 3.93 - 3.87 (m, 1H, major; m, 1H, minor), 3.75 - 3.64 (m, 1H, major; m, 1H, minor), 3.39 (dd, *J* = 13.6, 7.2 Hz, 1H), 3.27 (dq, *J* = 14.4, 7.2 Hz, 1H), 3.13 - 2.83 (m, 6H, major; m, 6H, minor), 2.74 (dd, *J* = 14.8, 6.4 Hz, 1H, minor), 2.57 - 2.42 (m, 2H, major; m, 2H, minor), 1.87 - 1.74 (m, 1H), 1.68 - 1.61 (m, 1H, minor), 1.60 - 1.40 (m, 2H, major; m, 2H, minor), 1.19 - 1.09 (m, 1H, major; m, 1H, minor), 1.06 (t, *J* = 7.2 Hz, 3H, minor), 0.93 (t, *J* = 7.2 Hz, 3H).

^{13}C NMR (100 MHz, $CDCl_3$) δ 172.7(major), 172.5(minor), 136.8(major), 136.7(minor), 129.1(minor), 129.0(major), 128.1(major), 128.0(minor), 127.52(major), 127.46(minor), 66.1(minor), 66.0(major), 52.7(minor), 52.0(major), 51.8(minor), 50.9(major), 46.2(minor), 46.1(major), 42.8(major), 41.4(minor), 36.1(minor), 34.9(major), 31.1(major), 30.9(minor), 13.6(major), 12.3(minor).

HRMS (ESI, *m*/*z*) calcd for $C_{17}H_{27}N_2O_2^+$ $[M+H]^+$: 291.2073, found: 291.2073.

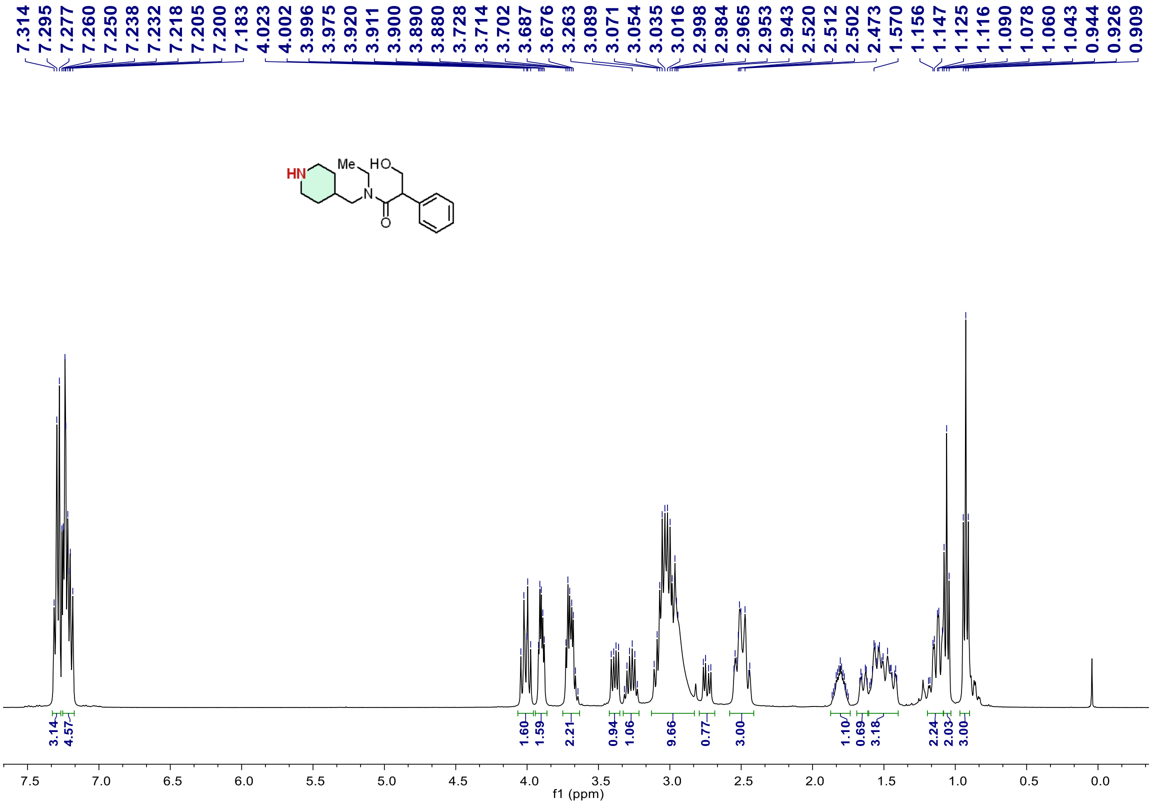
7.314
7.295
7.277
7.260
7.250
7.238
7.232
7.218
7.205
7.200
7.183
4.023
4.002
3.996
3.975
3.920
3.911
3.900
3.890
3.880
3.728
3.714
3.702
3.687
3.676
3.263
3.089
3.071
3.054
3.035
3.016
2.998
2.984
2.965
2.953
2.943
2.520
2.512
2.502
2.473
1.570
1.156
1.147
1.125
1.116
1.090
1.078
1.060
1.043
0.944
0.926
0.909
HN
Me
HO
N
O
3.14
4.57
1.60
1.59
2.21
0.94
1.06
9.66
0.77
3.00
1.10
0.69
3.18
2.24
2.03
3.00
7.5
7.0
6.5
6.0
5.5
5.0
4.5
4.0
3.5
3.0
2.5
2.0
1.5
1.0
0.5
0.0
f1 (ppm)

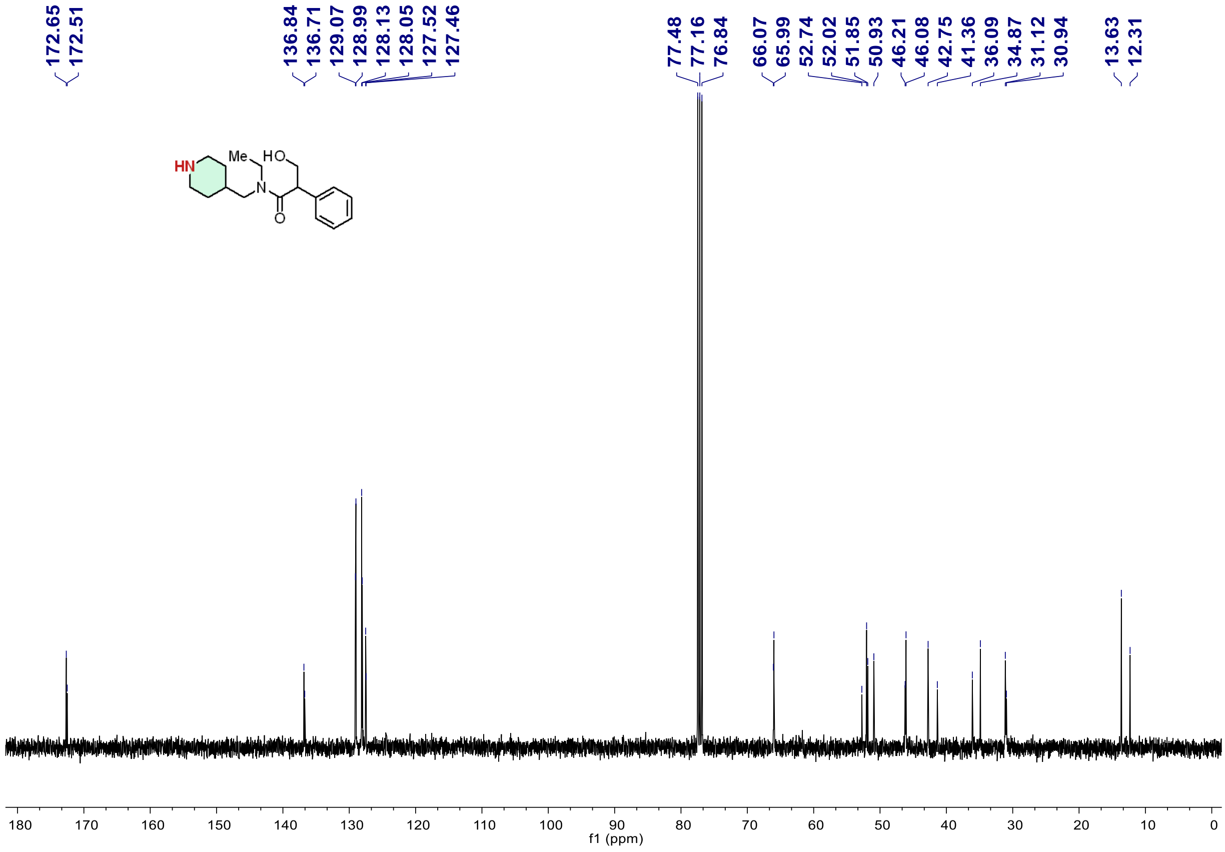
172.65
172.51
136.84
136.71
129.07
128.99
128.13
128.05
127.52
127.46
77.48
77.16
76.84
66.07
65.99
52.74
52.02
51.85
50.93
46.21
46.08
42.75
41.36
36.09
34.87
31.12
30.94
13.63
12.31
HN
Me
HO
N
O
180
170
160
150
140
130
120
110
100
90
80
70
60
50
40
30
20
10
0
f1 (ppm)

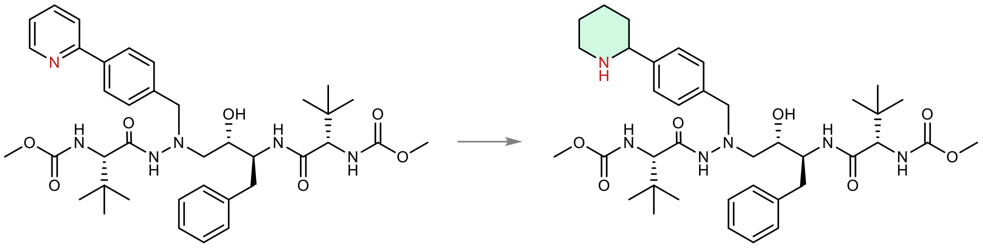

Atazanavir **74** (48% yield, 1:1 d.r.)

According to the general procedure, a mixture of **atazanavir** (140.9 mg, 0.20 mmol, 1.0 equiv.), $B_2(OH)_4$ (107.5 mg, 1.20 mmol, 6.0 equiv.) and $[Rh(COD)OH]_2$ (3.7 mg, 4.0 mol%, 0.008 mmol) in EtOH (1.0 mL, 0.2 M) was stirred under nitrogen atmosphere for 48 hours at 50 °C to afford **74** as a white solid (69.0 mg, 48% yield, 1:1 d.r.).

Purification conditions: CH_2Cl_2/NH_3(7.0 M solution in MeOH) = 50:1 to 20:1.

R_f= 0.3 in CH_2Cl_2/NH_3(7.0 M solution in MeOH) = 15:1.

^{1}H NMR (400 MHz, $CDCl_3$) δ 7.30 - 7.24 (m, 5H), 7.22 - 7.18 (m, 4H), 7.15 - 7.10 (m, 1H), 6.63 (d, *J* = 9.2 Hz, 1H), 5.47 (dd, *J* = 22.0, 9.2 Hz, 2H), 4.98 (br, 1H), 4.06 (q, *J* = 8.4 Hz, 1H), 3.98 (d, *J* = 14.0 Hz, 1H), 3.87 - 3.84 (m, 2H), 3.69 - 3.56 (m, 9H), 3.19 (d, *J* = 11.2 Hz, 1H), 2.93 (d, *J* = 7.6 Hz, 2H), 2.86 - 2.75 (m, 2H), 2.58 (d, *J* = 12.4 Hz, 1H), 2.53 - 2.29 (m, 1H), 1.92 - 1.84 (m, 1H), 1.74 (d, *J* = 8.8 Hz, 1H), 1.69 - 1.62 (m, 1H), 1.60 - 1.43 (m, 3H), 0.88 (s, 9H), 0.72 (s, 9H).

^{13}C NMR (100 MHz, $CDCl_3$) δ 171.0, 170.8, 157.0 (2C), 144.9, 138.2, 134.7, 129.4, 129.1, 128.3, 126.9, 126.3, 67.5, 63.6, 62.3, 62.1, 62.1, 61.4, 61.2, 52.5, 52.4, 52.1, 47.7, 38.8, 34.3, 34.0, 26.7, 26.3, 25.8, 25.4.

HRMS (ESI, *m/z*) calcd for $C_{38}H_{59}N_6O_7^+$ $[M+H]^+$: 711.4445, found: 711.4449.

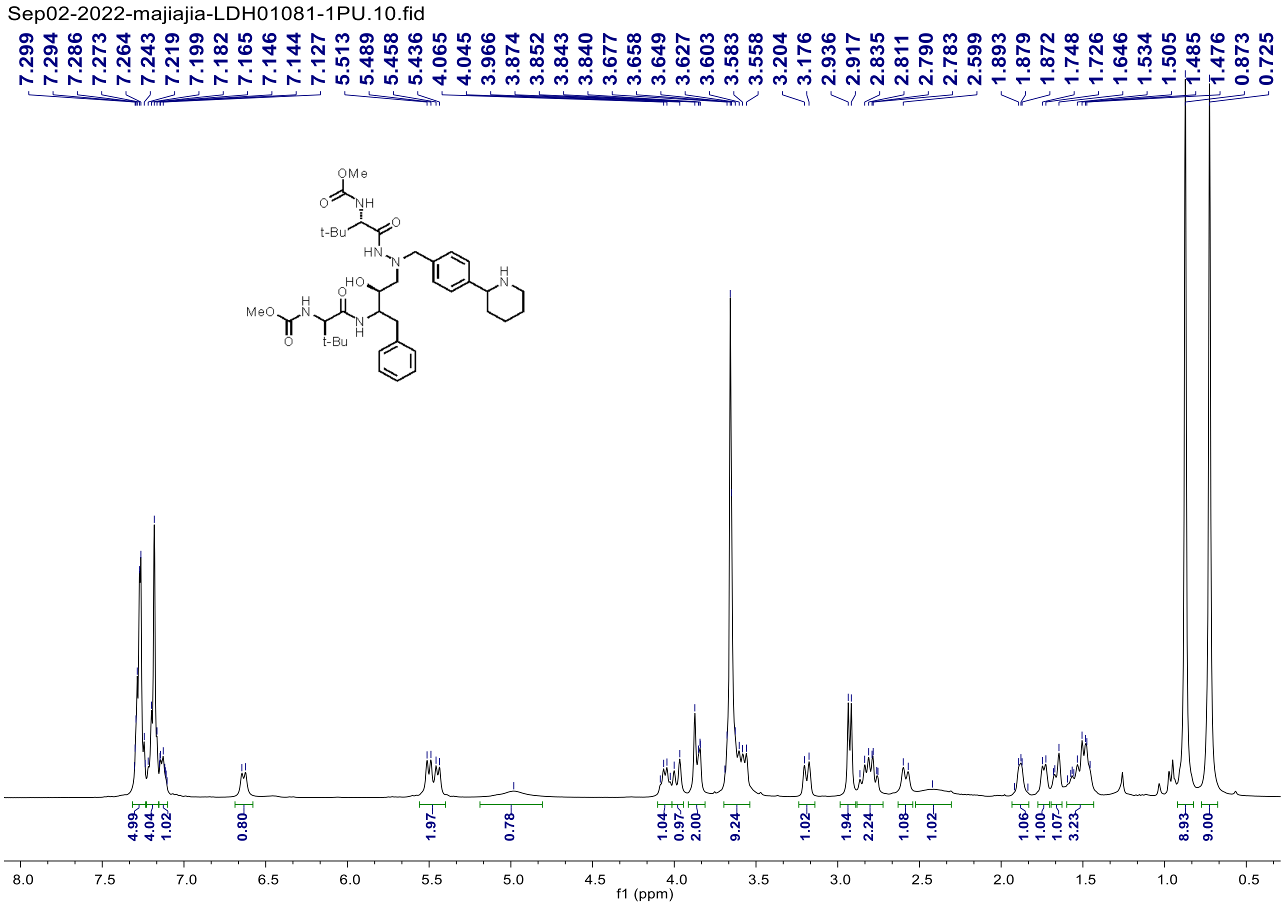
Sep02-2022-majiajia-LDH01081-1PU.10.fid
7.299 7.294 7.286 7.273 7.264 7.243 7.219 7.199 7.182 7.165 7.146 7.144 7.127 5.513 5.489 5.458 5.436 4.065 4.045 3.966 3.874 3.852 3.843 3.840 3.677 3.658 3.649 3.627 3.603 3.583 3.558 3.204 3.176 2.936 2.917 2.835 2.811 2.790 2.783 2.599 1.893 1.879 1.872 1.748 1.726 1.646 1.534 1.505 1.485 1.476 0.873 0.725
OMe
NH
t-Bu
HN
N
HO
H
N
MeO
N
H
t-Bu
4.99 4.04 1.02 0.80 1.97 0.78 1.04 0.97 2.00 9.24 1.02 1.94 2.24 1.08 1.02 1.06 1.00 1.07 3.23 8.93 9.00
f1 (ppm)

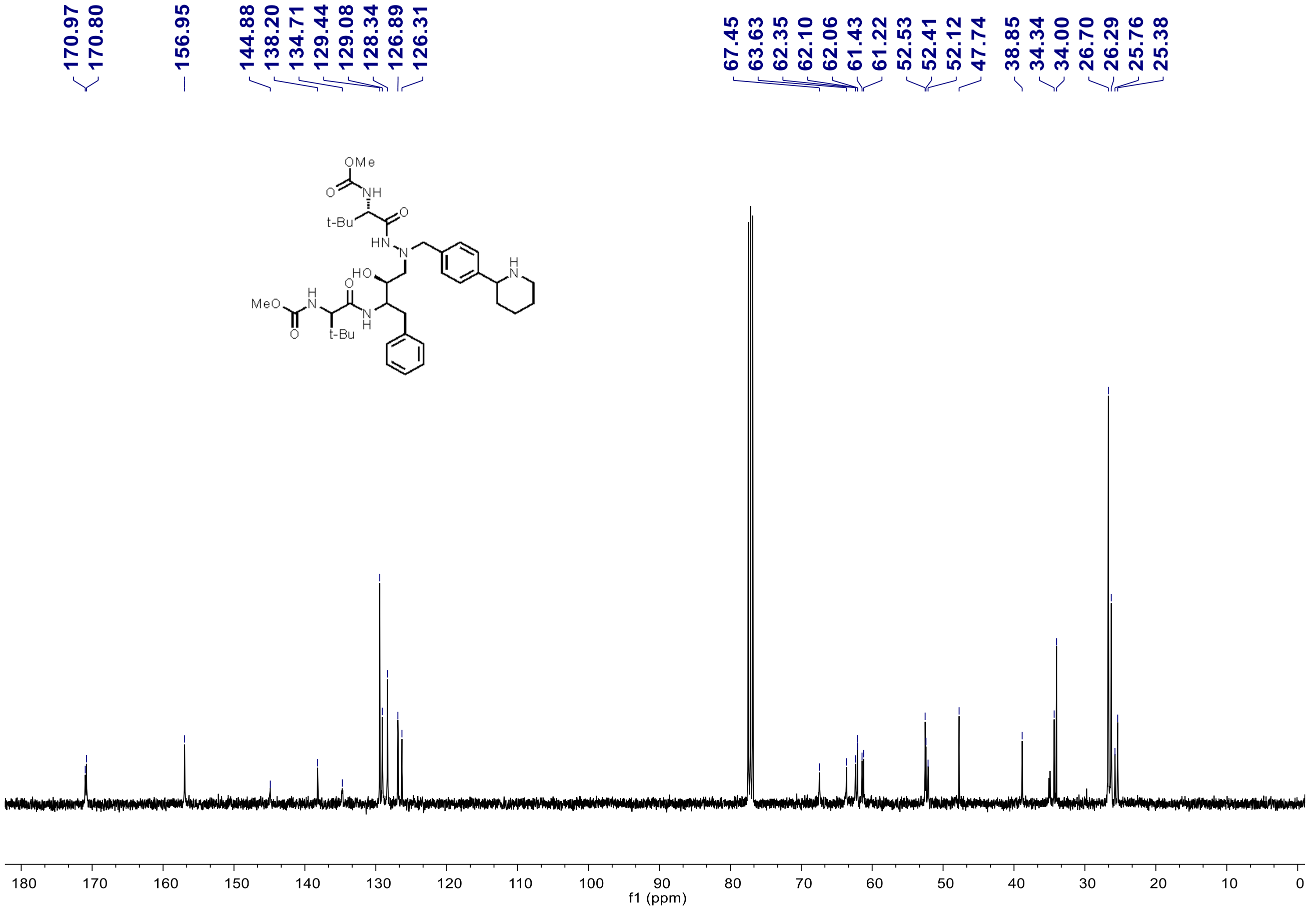
170.97 170.80 156.95 144.88 138.20 134.71 129.44 129.08 128.34 126.89 126.31
67.45 63.63 62.35 62.10 62.06 61.43 61.22 52.53 52.41 52.12 47.74 38.85 34.34 34.00 26.70 26.29 25.76 25.38
OMe
NH
t-Bu
HN
N
HO
H
N
MeO
N
H
t-Bu
f1 (ppm)

3. Procedure for Arene Reduction Comparison Screen

Stock solutions, or suspensions, were prepared as shown in the heatmap preparation table. In an inert atmosphere glovebox, reagents were weighed and dissolved or suspended in anhydrous solvent to achieve their listed concentrations in the associated table. Stock solutions of reagents were stirred until either a clear solution or a uniform slurry was achieved. A 96-well aluminum microvial plate (Analytical Sales & Services #96960) was equipped with oven-dried shell vials (Analytical Sales & Services cat. no. 884001) and then moved into the glovebox.. All stock solutions except for $[Rh(COD)OH]_2$ and $[Rh(cod)Cl]_2$ were prepared in DCM and dosed into the appropriate well locations using single channel micropipetters, then blown down under a stream of nitrogen. After complete removal of solvent from each well, the rhodium catalyst solutions were added last to achieve the final reaction volume (100 µL) and solvent composition for each well. A parylene-coated stir dowel (Analytical Sales & Services #13258) was then added to each vial. The microvial plate was sealed, removed from the glove box, and stirred on a tumble stirrer with heating to 50°C for 24 hours. The reactions were quenched by opening the reaction block and adding 100 µL saturated aqueous $NaHCO_3$ solution and 400 µL EtOAc. Reactions were extracted by resealing the plate and shaking manually. From each reaction, a 40 µL aliquot of the quenched reaction mixture was added into a 96-well polypropylene collection plate (Analytical Sales & Services cat. no. 17P687). The solvent was evaporated by blowing nitrogen down on the analytical plate. An acetonitrile solution of caffeine as internal standard (0.05 mg/mL, 800 µL) was added, and mixed by pipetting up and down. The reactions were then analyzed by UPLC-MS. The assay yields were produced by measuring the MS Ionization Peak Area of the desired product relative to the starting material.

The reaction data points for the 96-well screen are deposited on github:

https://github.com/cernak-lab/late_stage_saturation

**Table S1.** Recipe for Comparison Screen of Different Arene Reduction Conditions			
**Reagents**	**C_{stock} (M)**	**V_{dose} (µL)**	**Wells**
Propranolol	0.198	50	A1-A4, B1-B4, C1-C4, D1-D4, E1-E4, F1-F4, G1-G4, H1-H4
Gemfibrozil	0.198	50	A5-A8, B5-B8, C5-C8, D5-D8, E5-E8, F5-F8, G5-G8, H5-H8
Ketoprofen	0.198	50	A9-A12, B9-B12, C9-C12, D9-D12, E9-E12, F9-F12, G9-G12, H9-H12
$B_2(OH)_4$ (3.5 equiv)	0.693	50	Rows: A, B, E, F<br>Columns:1, 2, 5, 6, 9, 10
$B_2(OH)_4$ (7.0 equiv)	1.386	50	Rows: A, B, E, F<br>Columns: 3, 4, 7, 8, 11, 12
BH_3NH_3 (1.5 equiv)	0.297	50	Rows: C, D, G, H<br>Columns: 1, 2, 5, 6, 9, 10
BH_3NH_3 (3.0 equiv)	0.594	50	Rows: C, D, G, H<br>Columns: 3, 4, 7, 8, 11, 12
[Rh(COD)OH]$_2$ (in EtOH)	0.004	100	Rows: A, C<br>Columns: 1, 3, 5, 7, 9, 11
[Rh(cod)Cl]$_2$ (in EtOH)	0.002	100	Rows: E, G<br>Columns: 1, 3, 5, 7, 9, 11
[Rh(COD)OH]$_2$ (in TFE)	0.004	100	Rows: A, C<br>Columns: 2, 4, 6, 8, 10, 12
[Rh(cod)Cl]$_2$ (in TFE)	0.002	100	Rows: E, G<br>Columns: 2, 4, 6, 8, 10, 12
[Rh(COD)OH]$_2$ (in n-BuOH)	0.004	100	Rows: B, D<br>Columns: 1, 3, 5, 7, 9, 11
[Rh(cod)Cl]$_2$ (in n-BuOH)	0.002	100	Rows: F, H<br>Columns: 1, 3, 5, 7, 9, 11
[Rh(COD)OH]$_2$ (in Ethylene Glycol)	0.004	100	Rows: B, D<br>Columns: 2, 4, 6, 8, 10, 12
[Rh(cod)Cl]$_2$ (in Ethylene Glycol)	0.002	100	Rows: F, H<br>Columns: 2, 4, 6, 8, 10, 12

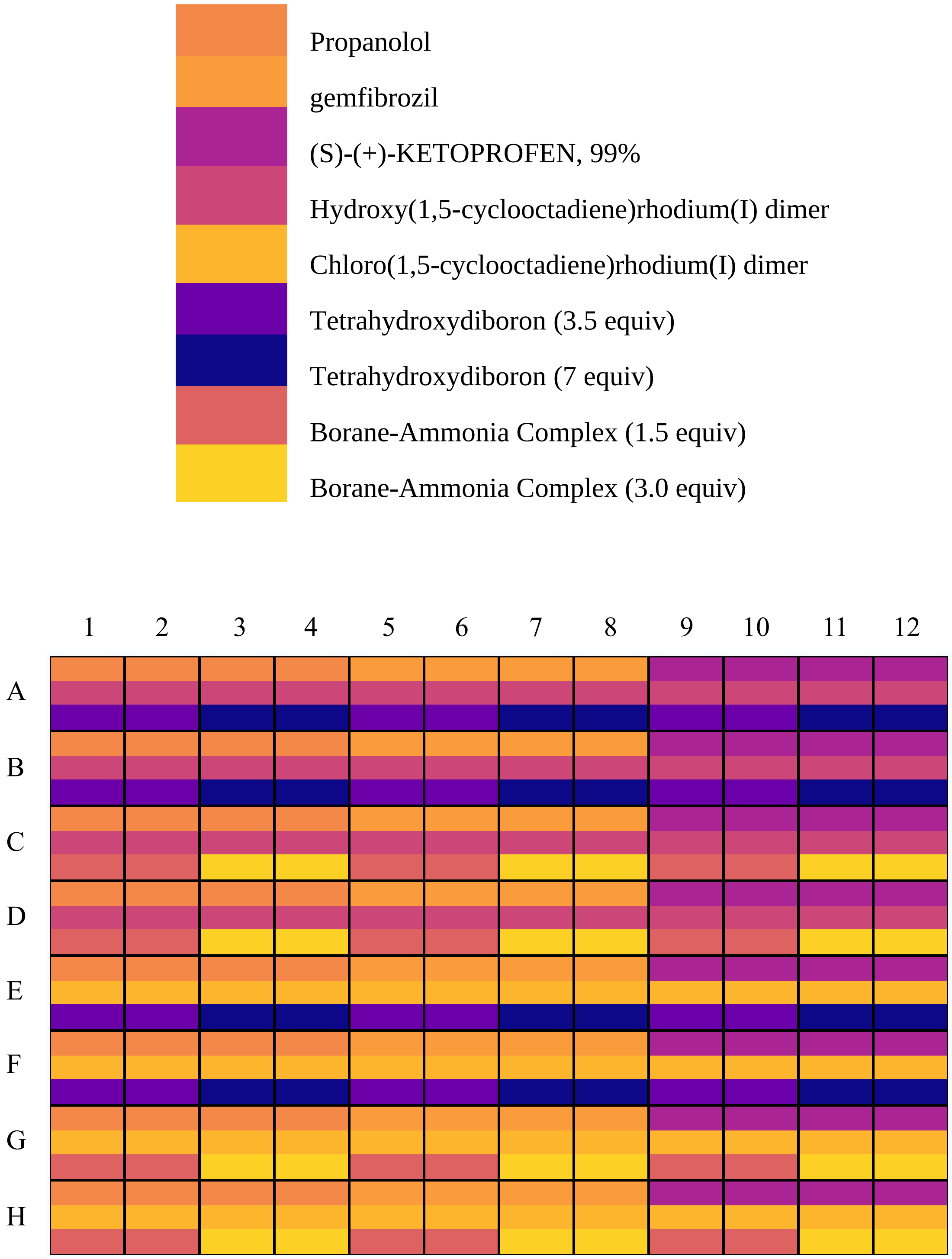

Figure S1. Reagents and Grid of Comparison Screen

a

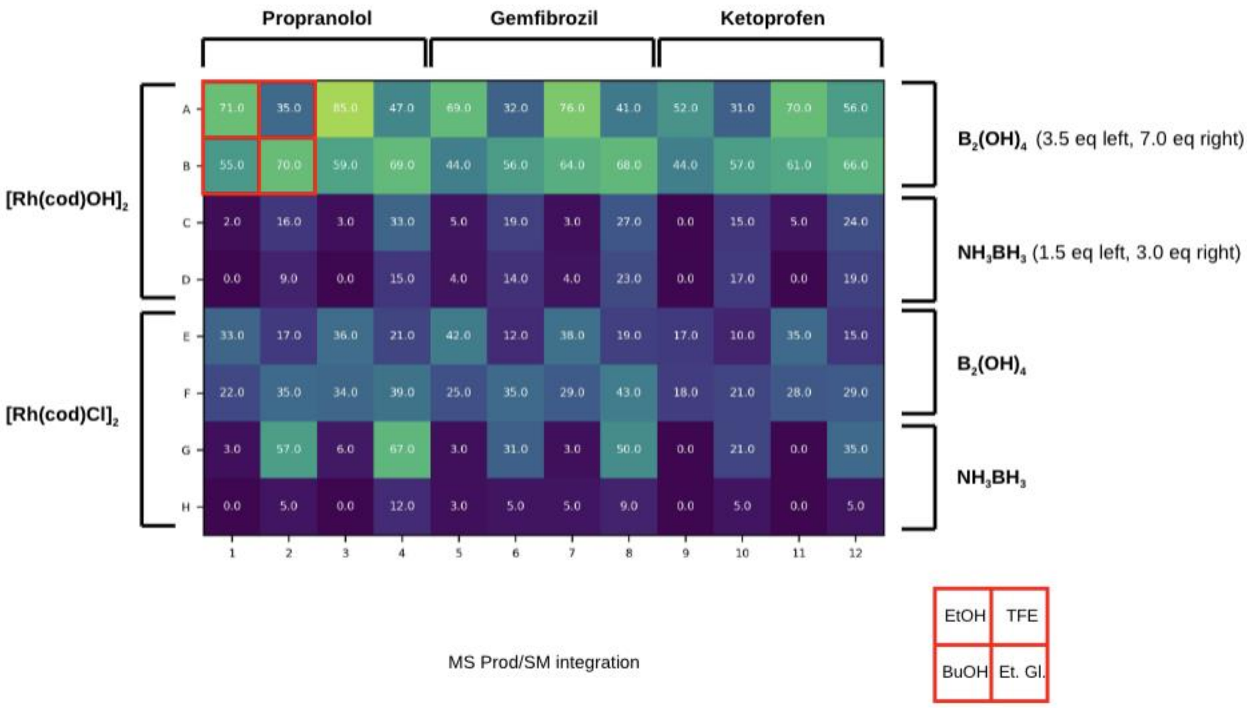

b

Additive	NMR Yield
None	60%
DMSO (0.10 eq)	57%
DMSO (0.10 eq) in EtOH	58%
DMSO (0.25 eq)	52%
DMSO (5% vol.)	N.R.
DMSO (100% vol.)	N.R.
Water (5% vol.)	56%

Figure S2 a; High-throughput experimentation campaign to interrogate mild late-stage saturation conditions on drug substrates, propranolol, gemfibrozil, and ketoprofen under mild autoclave-free hydrogenation conditions. cod = 1,5-cyclooctadiene, TFE = 1,1,1-trifluoroethanol, Et. Gl. = ethylene glycol. **b**; The effect of DMSO and water as an additive on reactions using aminacrine **7**. NMR yields represent the overall yield of mono- and di-reduced products **8** and **9**.

4. Step-Economic Synthesis of Lead Compounds

a) Previous 7-step synthesis of **51** from compound **50** in WO203043625A1, 2003

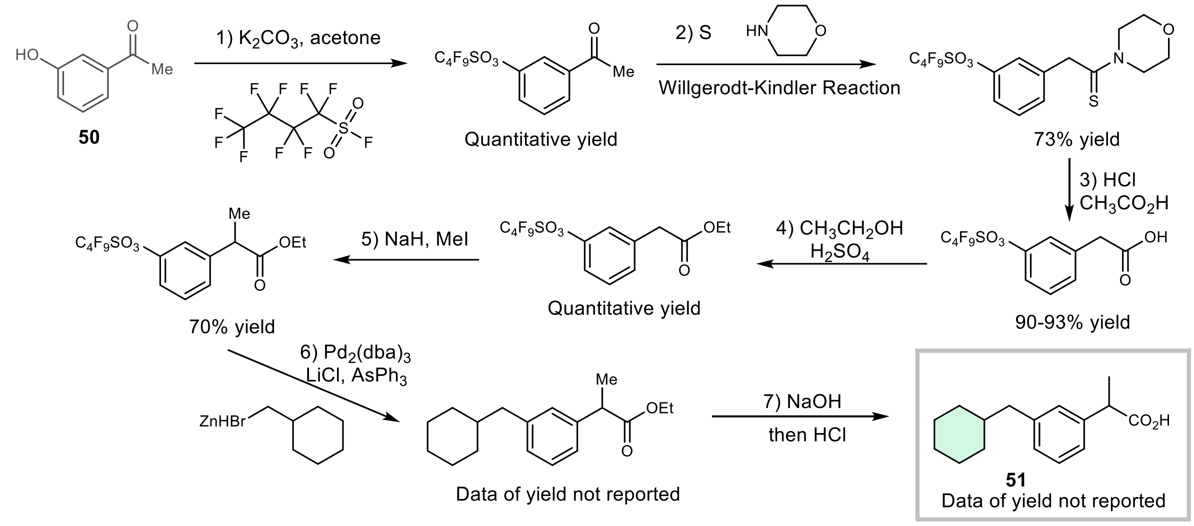

b) This work: single-step synthesis of **51**

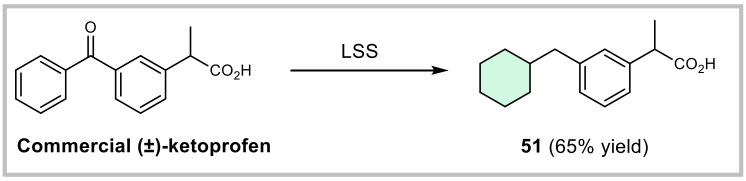

According to the general procedure, a mixture of commercial **(±)-ketoprofen** (50.8 mg, 0.20 mmol, 1.0 equiv.), $B_2(OH)_4$ (80.6 mg, 0.9 mmol, 4.5 equiv.) and $[Rh(COD)OH]_2$ (2.4 mg, 2.5 mol%, 0.005 mmol) in EtOH (1.0 mL, 0.2 M) was stirred under nitrogen atmosphere for 16 hours at 50 °C to afford **51** as a white oil (32.3 mg, 65% yield).

Purification conditions: petroleum ether/EtOAc = 10:1 to 5:1.

R_f(**51**) = 0.4 in petroleum ether/EtOAc (3:1).

^{1}H NMR (400 MHz, $CDCl_3$) δ 7.23 (t, J = 7.6 Hz, 1H), 7.14 (d, J = 7.6 Hz, 1H), 7.08 (s, 1H), 7.05 (d, J = 7.6 Hz, 1H), 3.71 (q, J = 7.2 Hz, 1H), 2.47 (d, J = 6.8 Hz, 2H), 1.73 - 1.61 (m, 5H), 1.51 (d, J = 7.2 Hz, 4H), 1.23 - 1.11 (m, 3H), 1.00 - 0.88 (m, 2H).

^{13}C NMR (100 MHz, $CDCl_3$) δ 180.7, 142.0, 139.6, 128.7, 128.4, 128.4, 124.9, 45.5, 44.2, 39.8, 33.3, 26.7, 26.4, 18.3.

HRMS (ESI, m/z) calcd for $C_{16}H_{22}O_2Na^+$ $[M+Na]^+$: 269.1517, found: 269.1514.

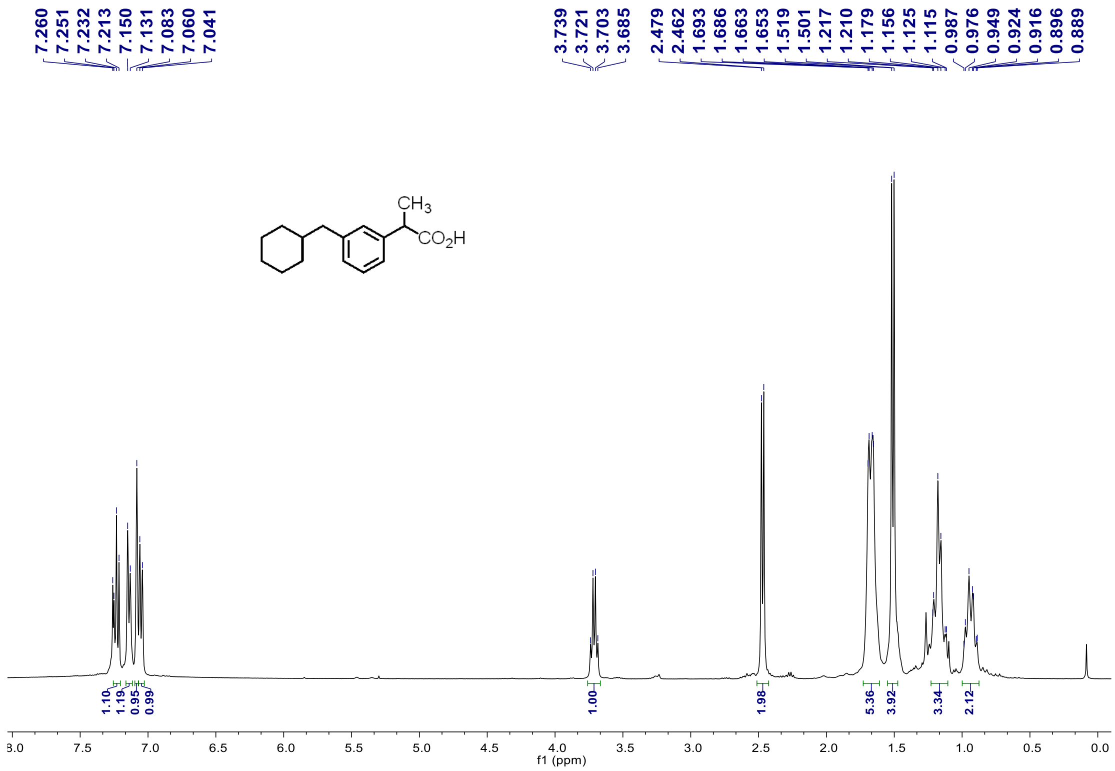
7.260
7.251
7.232
7.213
7.150
7.131
7.083
7.060
7.041
3.739
3.721
3.703
3.685
2.479
2.462
1.693
1.686
1.663
1.653
1.519
1.501
1.217
1.210
1.179
1.156
1.125
1.115
0.987
0.976
0.949
0.924
0.916
0.896
0.889
CH3
CO2H
1.10
1.19
0.95
0.99
1.00
1.98
5.36
3.92
3.34
2.12
f1 (ppm)

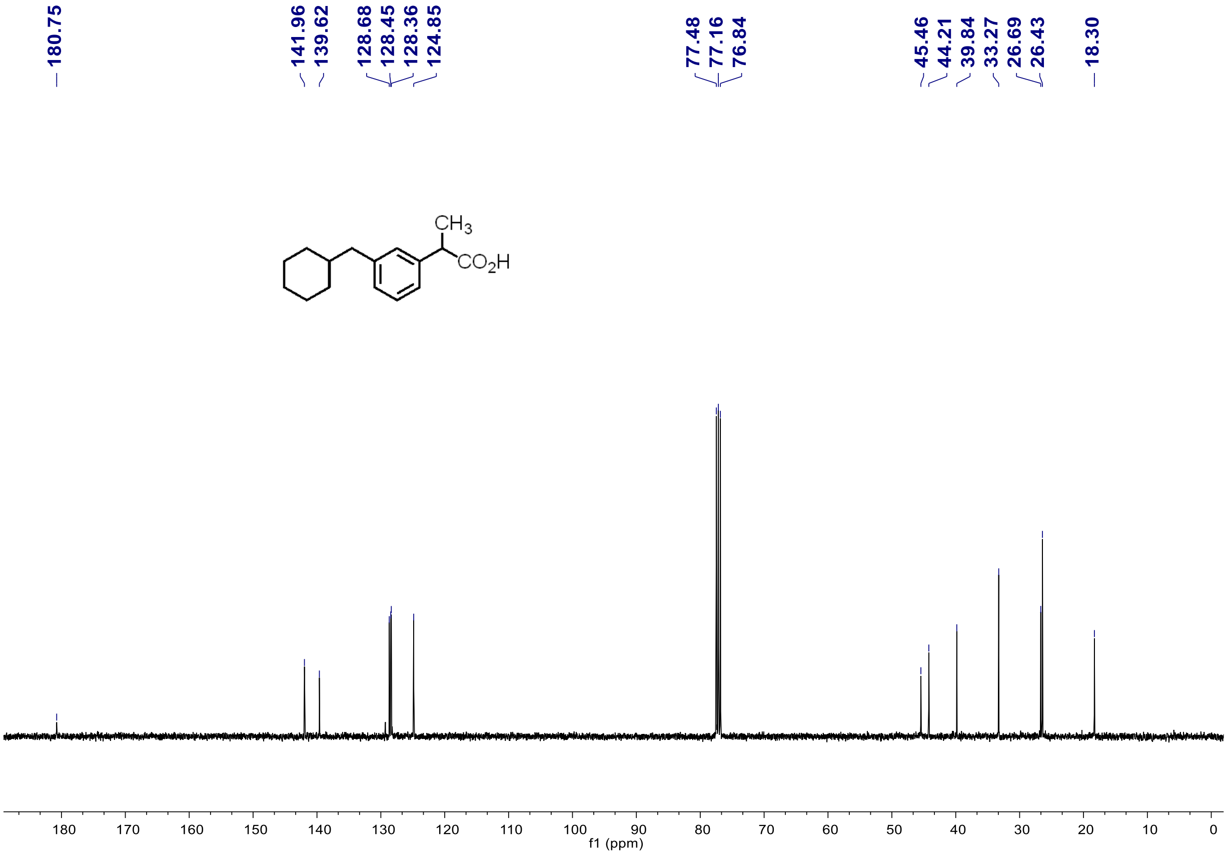
180.75
141.96
139.62
128.68
128.45
128.36
124.85
77.48
77.16
76.84
45.46
44.21
39.84
33.27
26.69
26.43
18.30
CH3
CO2H
f1 (ppm)

a) Previous 13-step synthesis of **54** from compound **53** in J. Med. Chem. 1977, 20, 726

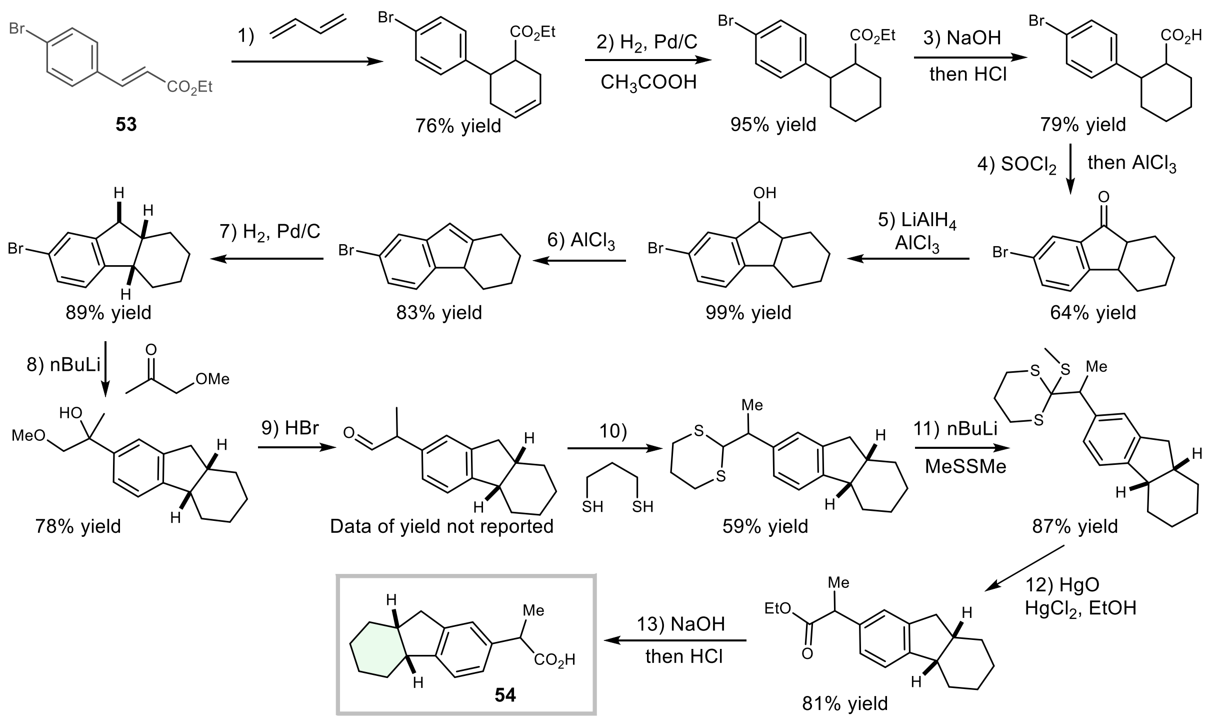

b) This work: single-step synthesis of **54**

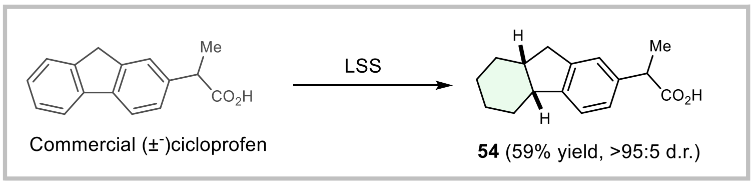

According to the general procedure, a mixture of commercial **(±)-cicloprofen** (47.7 mg, 0.20 mmol, 1.0 equiv.), $B_2(OH)_4$ (164.6 mg, 1.8 mmol, 9.0 equiv.) and $[Rh(COD)OH]_2$ (4.8 mg, 5.0 mol%, 0.01 mmol) in EtOH (1.0 mL, 0.2 M) was stirred under nitrogen atmosphere for 48 hours at 80 °C to afford **54** as a white solid (28.8 mg, 59% yield, >95:5 d.r.).

Purification conditions: petroleum ether/acetone = 20:1 to 8:1.

R_f(**54**) = 0.4 in petroleum ether/acetone = 3:1.

^{1}H NMR (400 MHz, $CDCl_3$) δ 7.18 (s, 1H), 7.13 - 7.07 (m, 2H), 3.71 (q, J = 7.2 Hz, 1H), 3.05 (q, J = 6.0 Hz, 1H), 2.82 (dd, J = 15.2, 6.4 Hz, 1H), 2.57 (dd, J = 15.2, 4.4 Hz, 1H), 2.48 - 2.38 (m, 1H), 1.87 - 1.70 (m, 2H), 1.60 - 1.52 (m, 1H), 1.50 (d, J = 7.2 Hz, 3H), 1.47 - 1.28 (m, 5H).

^{13}C NMR (100 MHz, $CDCl_3$, mixture of diastereomers) δ 180.6, 146.4, 144.5, 137.7, 125.6, 125.4 124.6, 124.4, 123.12, 123.10, 45.5, 43.8, 40.0, 37.55, 37.52, 28.0, 27.4, 23.9, 22.7, 18.5.

HRMS (ESI, m/z) calcd for $C_{16}H_{20}O_2Na^+$ $[M+Na]^+$: 267.1361, found: 267.1359.

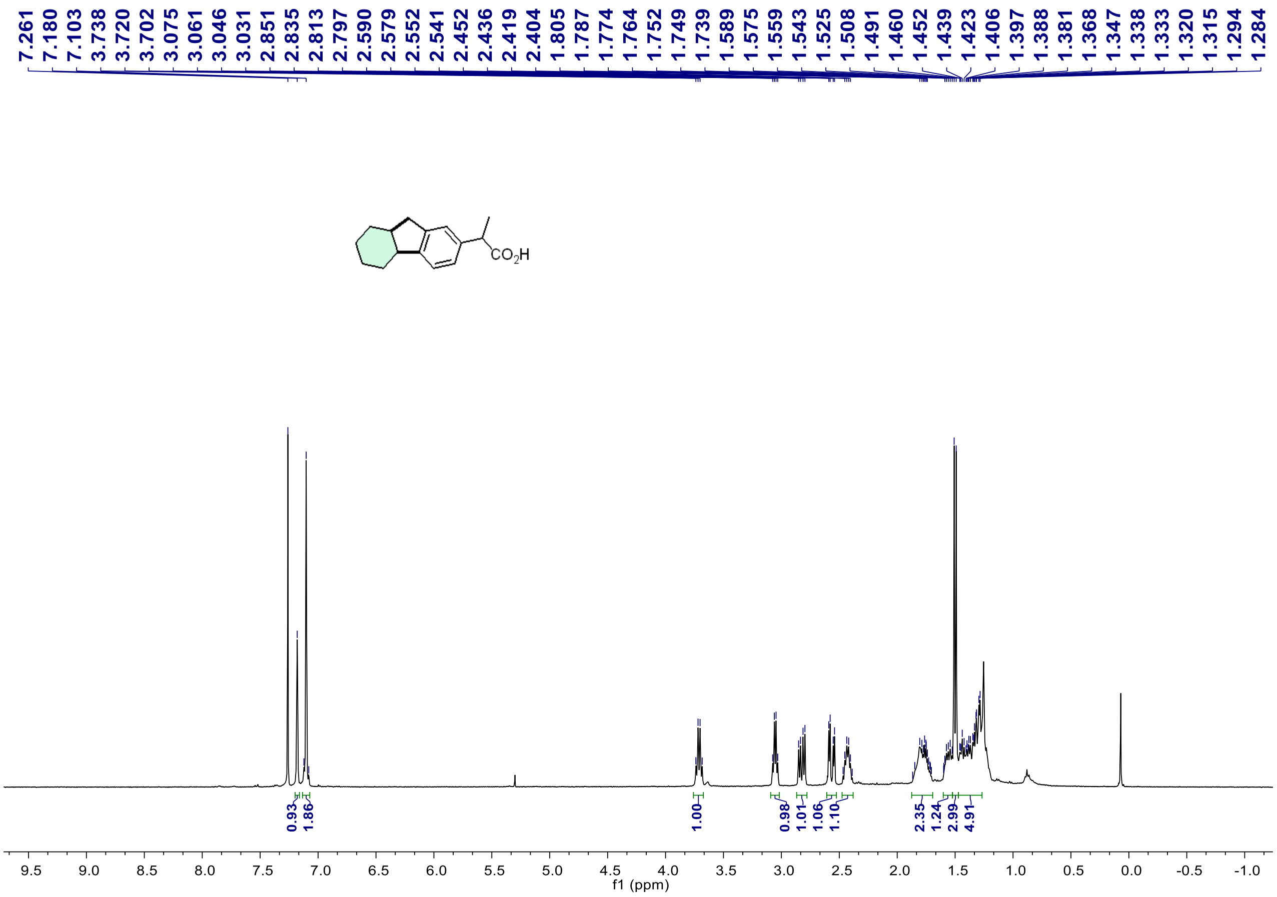

13C-ldh01068.1.fid

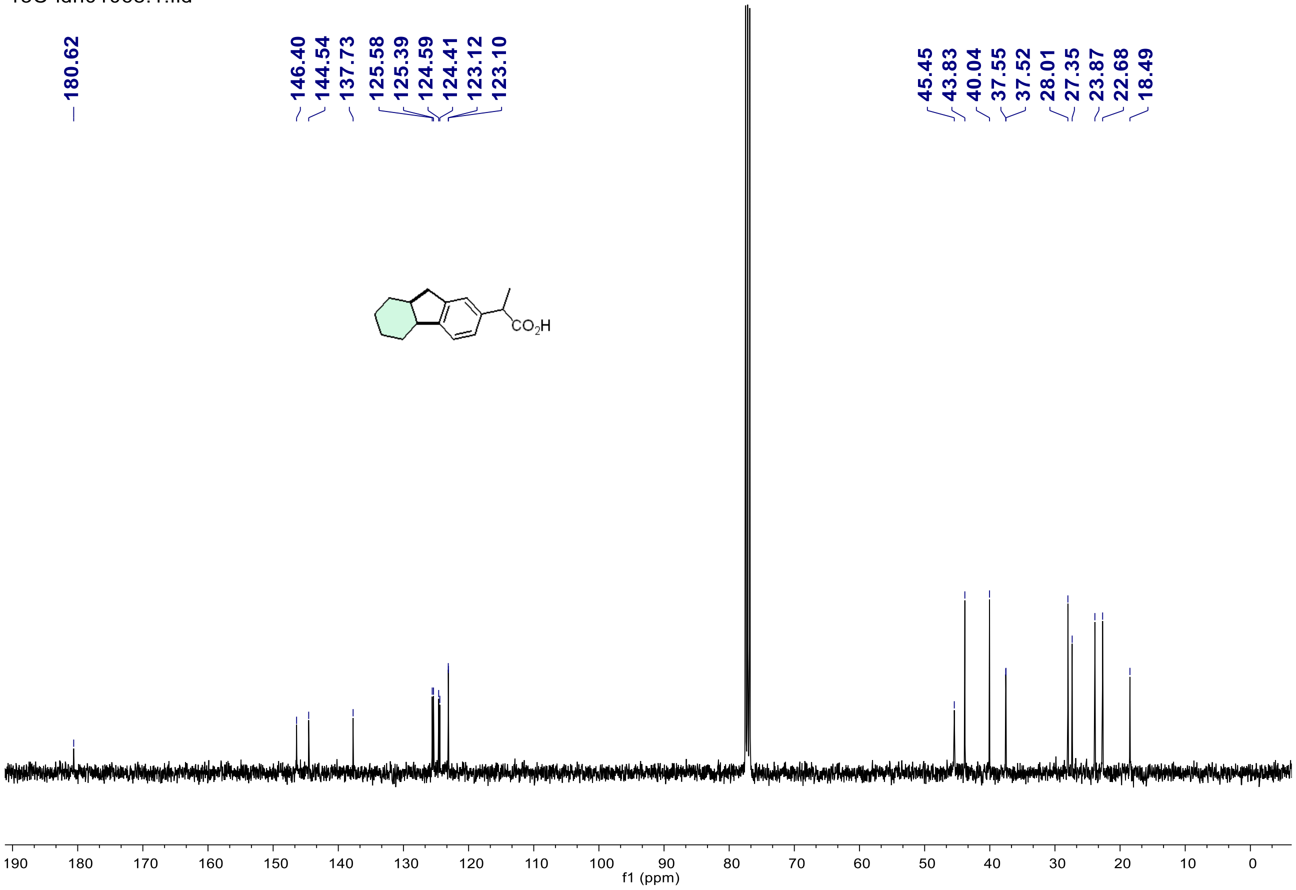

a) Previous 12-step synthesis of **58** and **59** from compound **56** and **57**, respectively, in J. Med. Chem. 1977, 20, 709

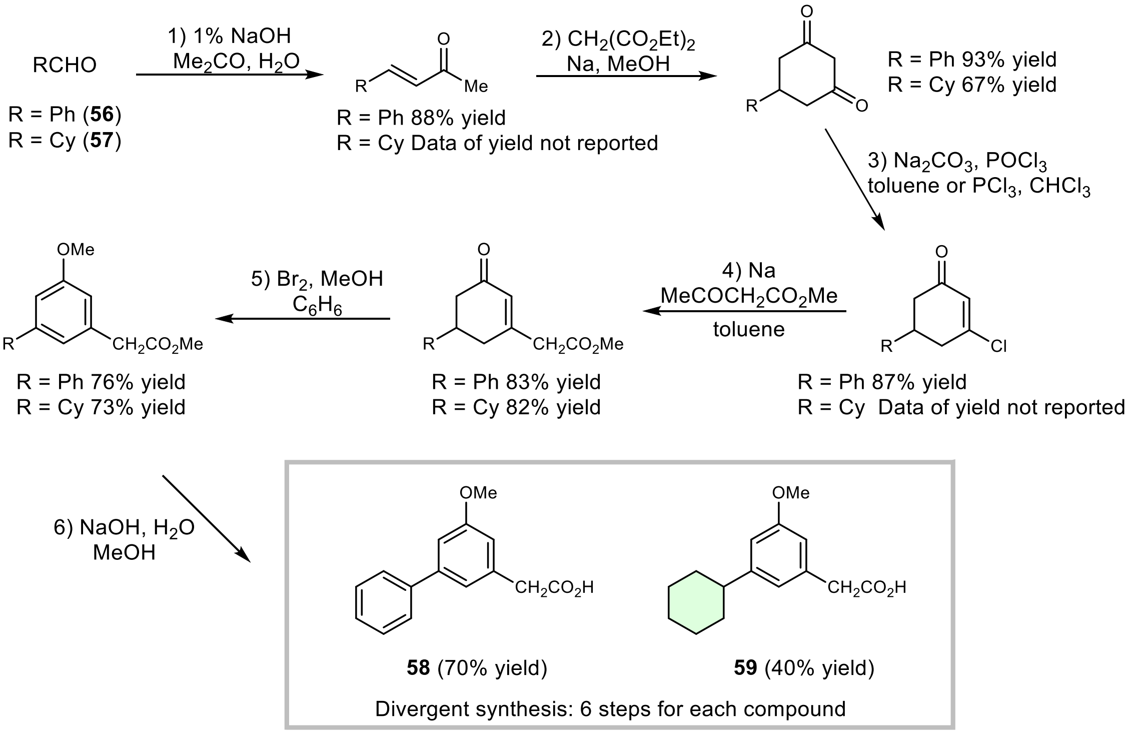

b) This work:7-step synthesis of **58** and **59**

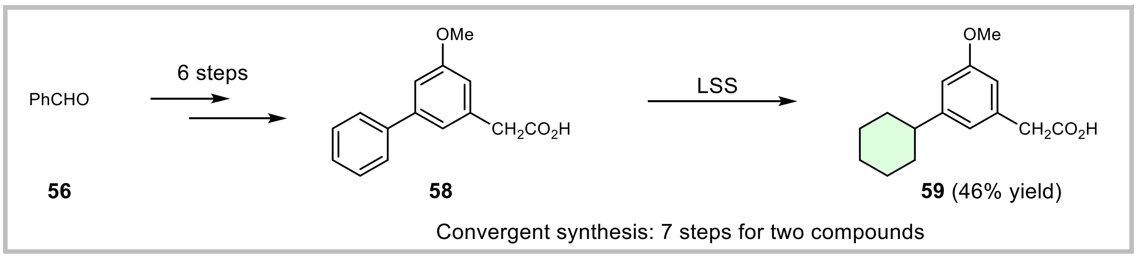

According to the general procedure, a mixture of **58** (48.4 mg, 0.20 mmol, 1.0 equiv.), $B_2(OH)_4$ (164.6 mg, 1.8 mmol, 9.0 equiv.) and $[Rh(COD)OH]_2$ (4.8 mg, 5.0 mol%, 0.01 mmol) in EtOH (1.0 mL, 0.2 M) was stirred under nitrogen atmosphere for 26 hours at 80 °C to afford **59** as a yellow solid (22.8 mg, 46% yield).

Purification conditions: petroleum ether/acetone = 20:1 to 6:1.

R_f = 0.35 in petroleum ether/acetone = 3:1.

^{1}H NMR (400 MHz, $CDCl_3$) δ 6.73 (s, 1H), 6.68 (d, J = 10.0 Hz, 2H), 3.80 (s, 3H), 3.60 (s, 2H), 2.52 - 2.42 (m, 1H) ,1.92 - 1.80 (m, 4H), 1.75 (d, J = 12.4 Hz, 1H), 1.46 - 1.32 (m, 4H), 1.31 - 1.19 (m, 1H).

^{13}C NMR (100 MHz, $CDCl_3$) δ 177.9, 159.9, 150.2, 134.4, 120.7, 112.2, 111.9, 55.3, 44.7, 41.4, 34.5, 27.0, 26.3.

HRMS (ESI, m/z) calcd for $C_{15}H_{20}O_3Na^+$ $[M+Na]^+$: 271.1310, found: 271.1310.

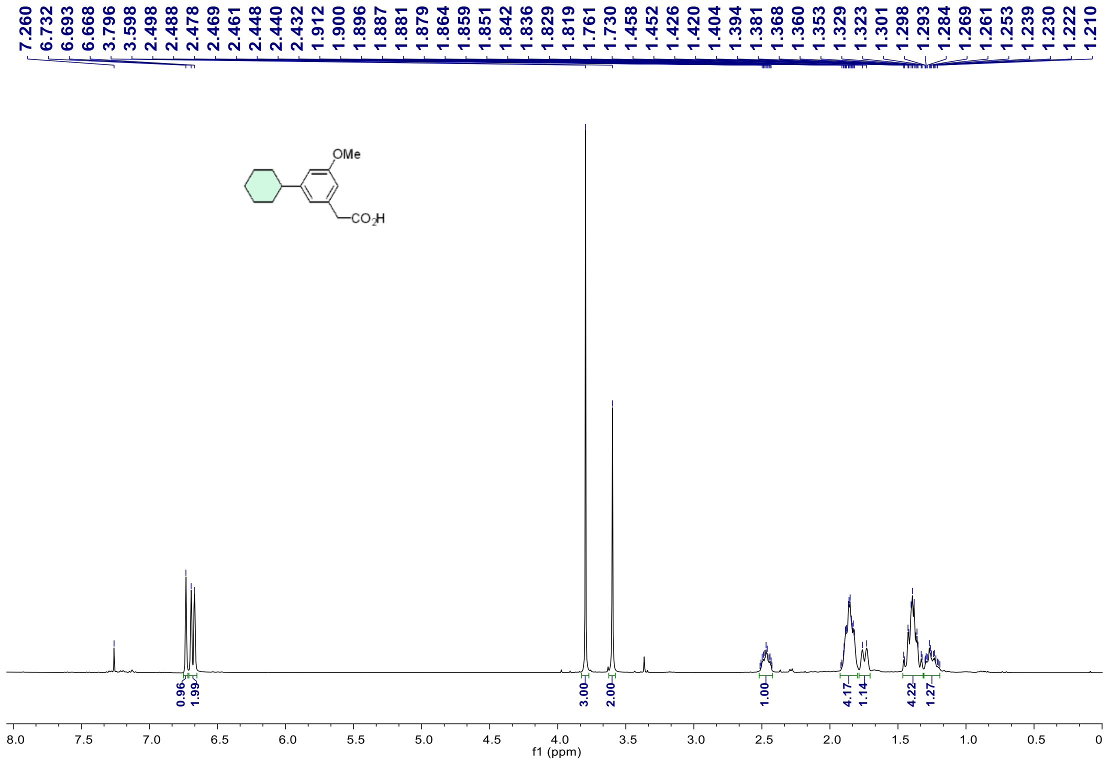

Aug04-2022-majiajia-LDH01060PU.11.fid

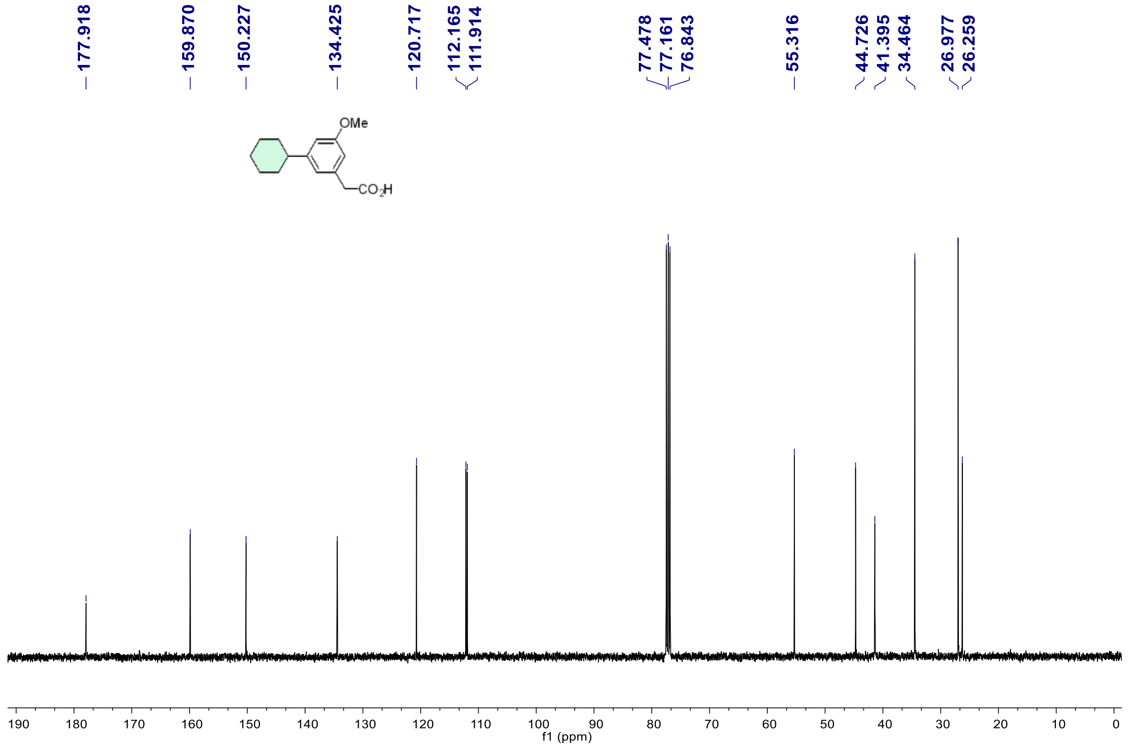

a) Previous 4-step synthesis of **61** from compound **60** J. Med. Chem. 2007, 50, 3984

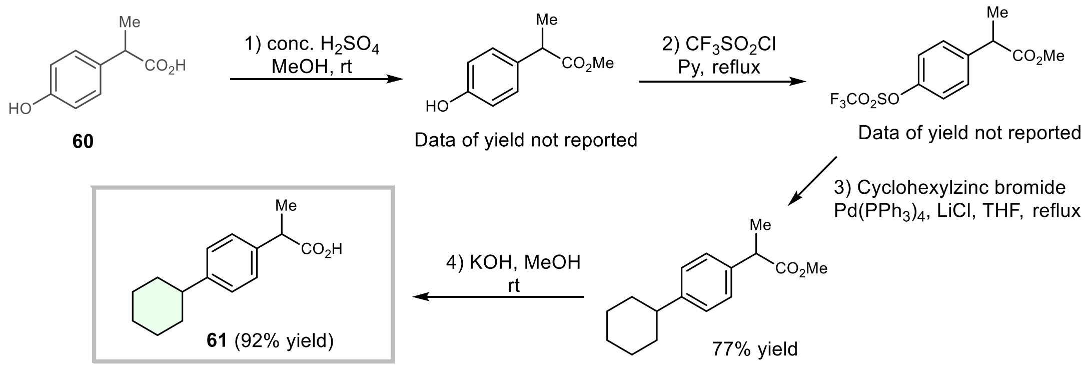

b) This work:single-step synthesis of **61** and **63**

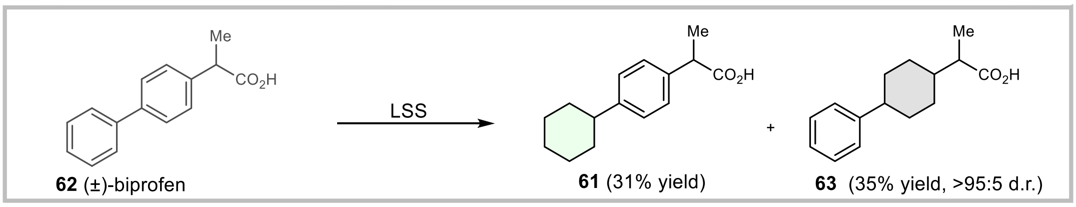

According to the general procedure, a mixture of **(±)-biprofen** (45.3 mg, 0.20 mmol, 1.0 equiv.), $B_2(OH)_4$ (107.5 mg, 1.20 mmol, 6.0 equiv.) and $[Rh(COD)OH]_2$ (3.7 mg, 4.0 mol%, 0.008 mmol) in EtOH (1.0 mL, 0.2 M) was stirred under nitrogen atmosphere for 48 hours at 80 °C to afford **61** as a white solid (14.5 mg, 31% yield) and **63** as a white solid (16.3 mg, 35% yield, >95:5 d.r.).

Purification conditions: Reverse-phase column chromatography (MeOH/H_2O = 1:4 to 2:1) on SepaBean machine (Santai Technology Inc., China) equipped with C18-bonded SepaFlash columns. R_f = 0.4 in petroleum ether/ acetone = 4:1 (the mixture of **61** and **63**).

Analytical data of **61:**

^{1}H NMR (400 MHz, $CDCl_3$) δ 7.24 (d, *J* = 8.0 Hz, 2H), 7.16 (d, *J* = 8.0 Hz, 2H), 3.71 (q, *J* = 7.2 Hz, 1H), 2.52 - 2.44 (m, 1H), 1.90 - 1.79 (m, 4H), 1.78 - 1.71 (m, 1H), 1.50 (d, *J* = 7.2 Hz, 3H), 1.45 - 1.32 (m, 4H), 1.29 - 1.18 (m, 1H).

^{13}C NMR (100 MHz, $CDCl_3$) δ 180.5, 147.4, 137.2, 127.6, 127.2, 45.0, 44.3, 34.5, 27.0, 26.3, 18.2.

HRMS (ESI, *m/z*) calcd for $C_{15}H_{20}O_2Na^+$ $[M+Na]^+$: 255.1361, found: 255.1359.

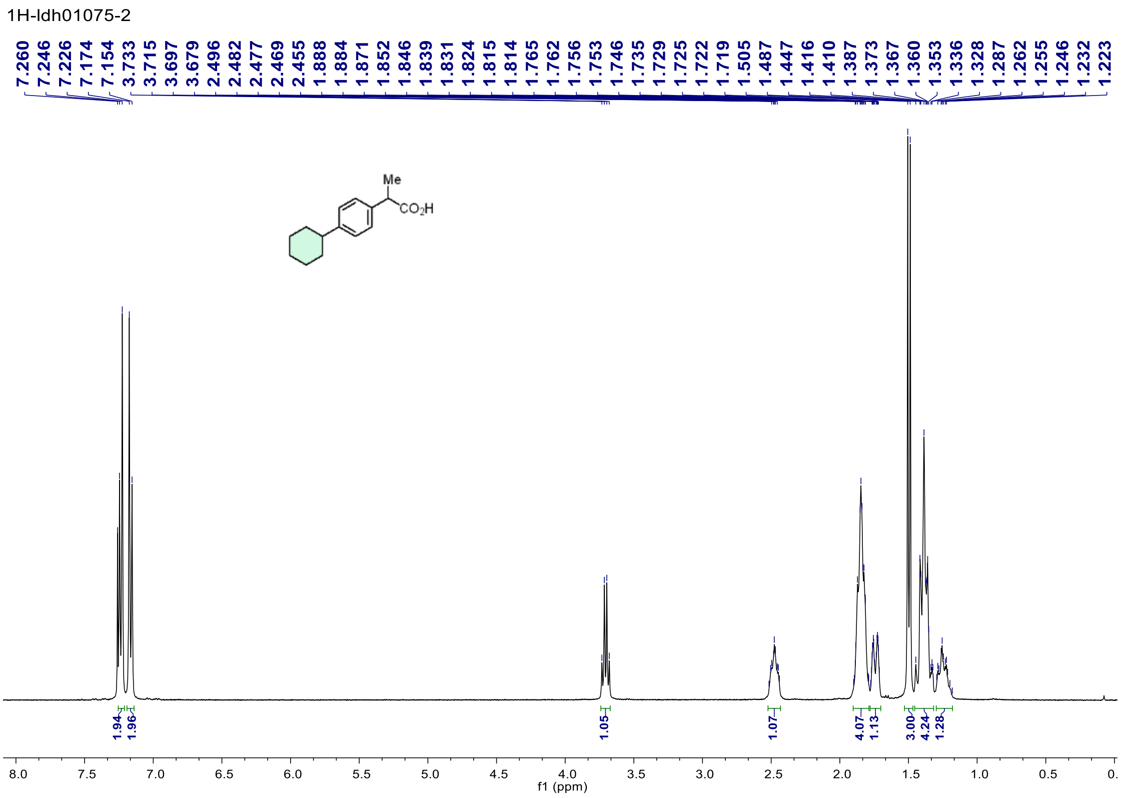

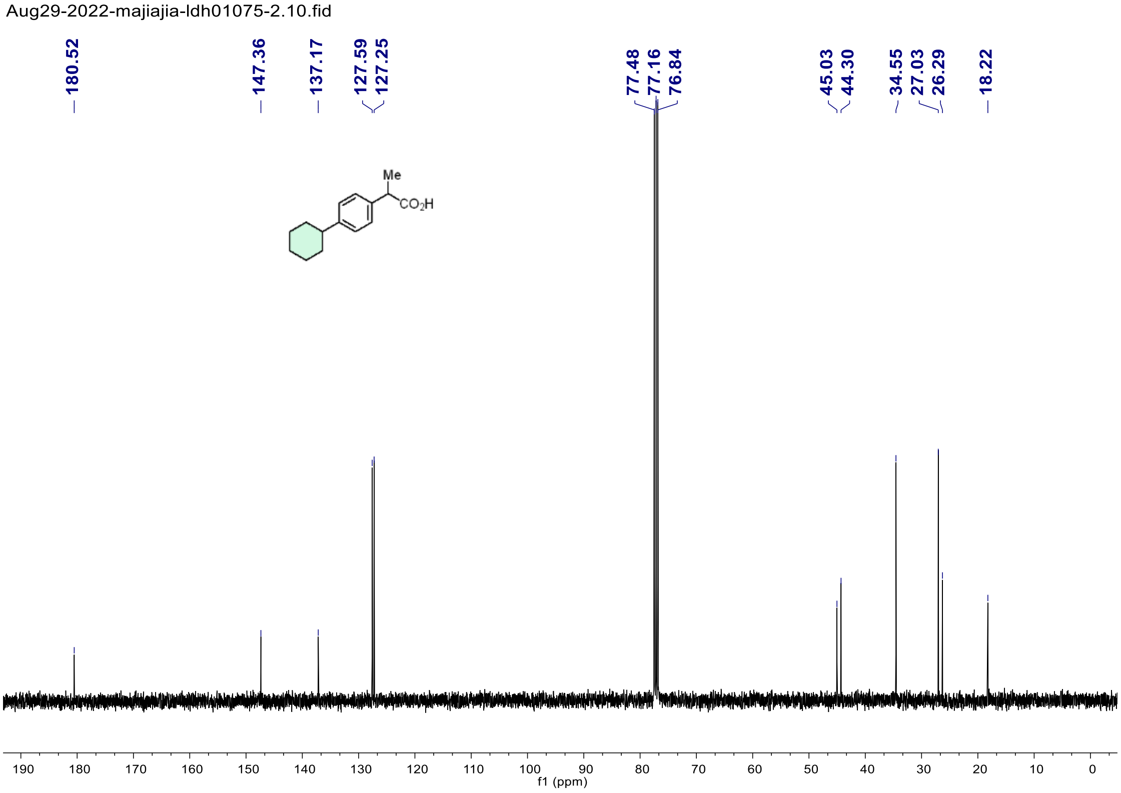

Analytical data of **63:**

^{1}H NMR (400 MHz, $CDCl_3$) δ 7.30 - 7.27 (m, 2H), 7.24 - 7.21 (m, 2H), 7.20 - 7.14 (m, 1H), 2.76 - 2.60 (m, 2H), 1.97 - 1.59 (m, 9H), 1.19 (d, *J* = 6.8 Hz, 3H).

^{13}C NMR (100 MHz, $CDCl_3$) δ 183.2, 146.8, 128.4, 127.1, 126.9, 126.0, 42.8, 40.5, 36.7, 29.1, 29.0, 28.9, 26.7, 15.5.

HRMS (ESI, *m*/*z*) calcd for $C_{15}H_{20}O_2Na^+$ [M+Na]$^+$: 255.1361, found: 255.1356.

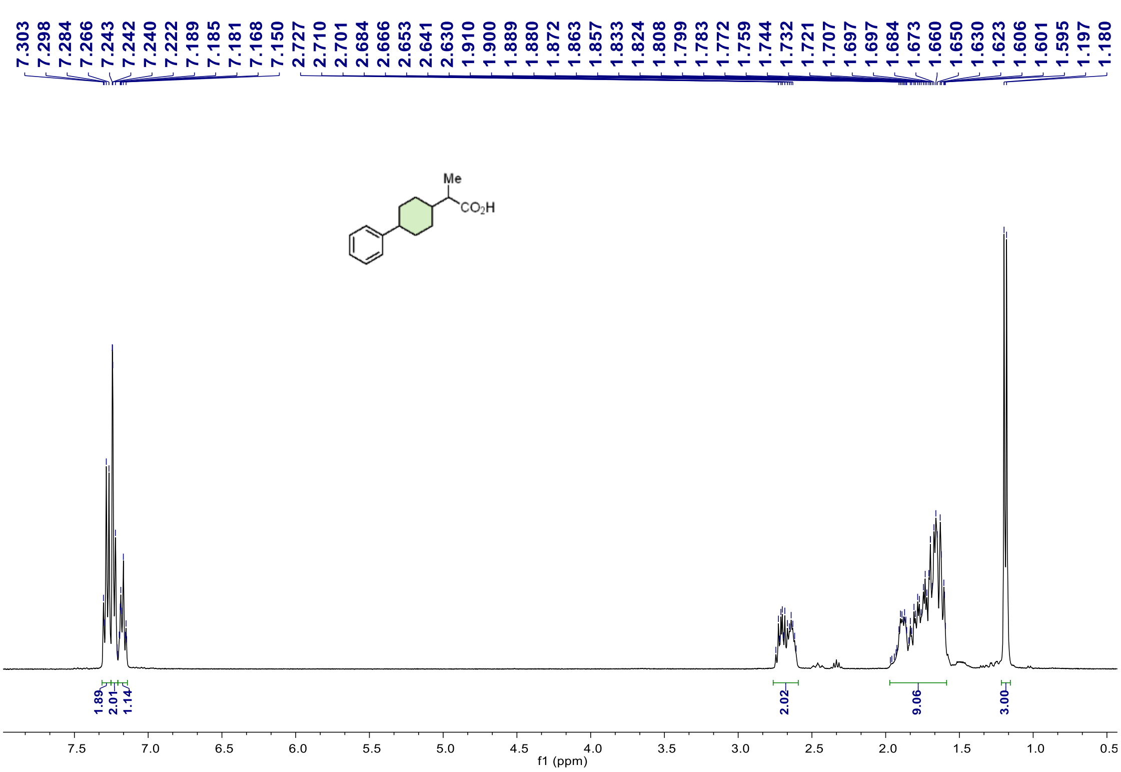

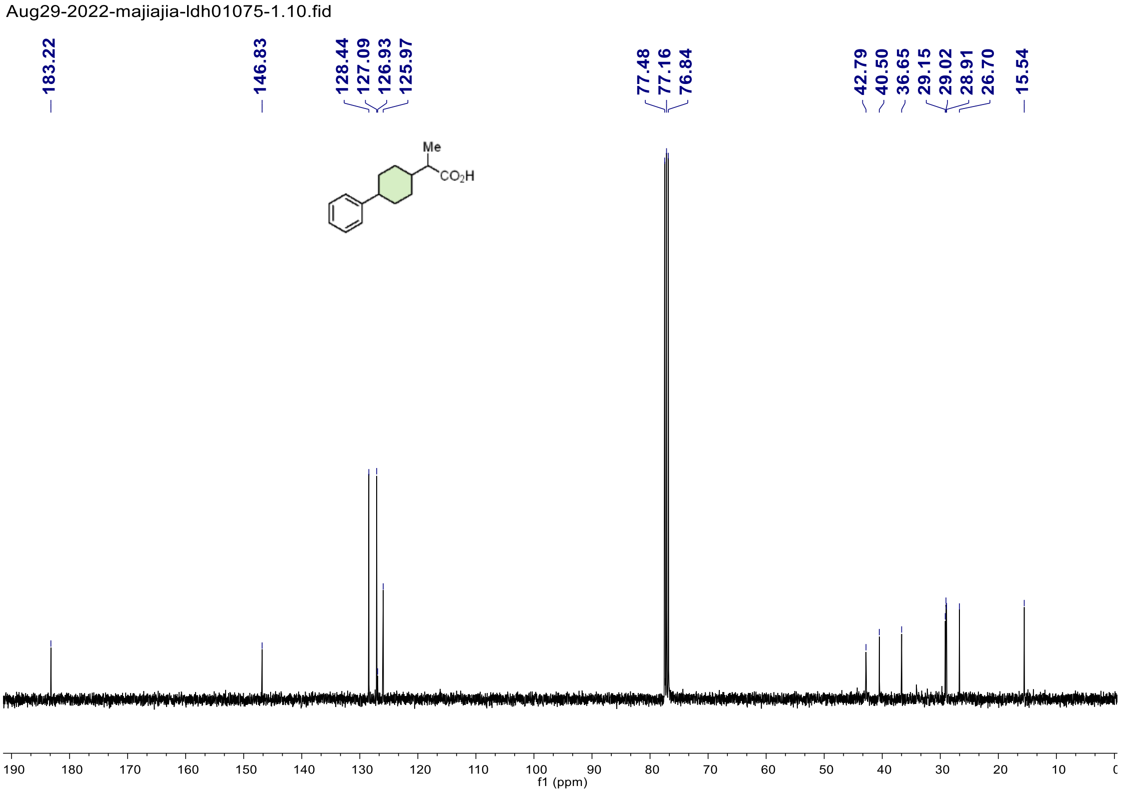

a) Previous 3-step synthesis of **66** from compound **64** in Bioorg. Med. Chem. 1998, 6, 9

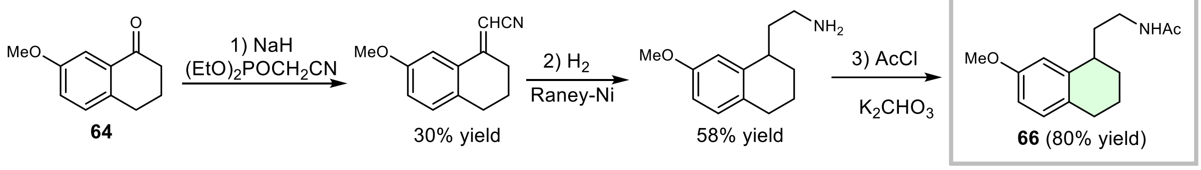

b) This work: single-step synthesis of **66**

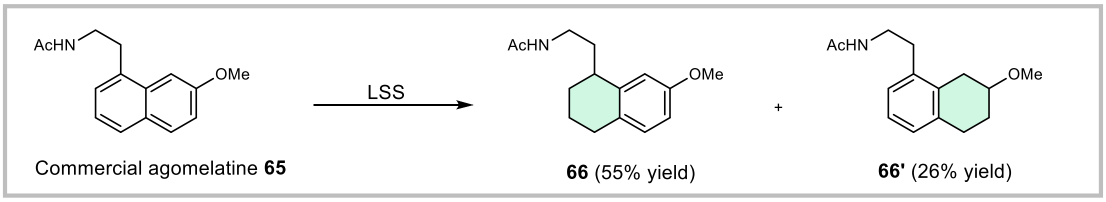

According to the general procedure, a mixture of **agomelatine** (48.6 mg, 0.2 mmol, 1.0 equiv.), $B_2(OH)_4$ (80.6 mg, 0.9 mmol, 4.5 equiv.) and $[Rh(COD)OH]_2$ (2.3 mg, 2.5 mol%, 0.005 mmol) in EtOH (1.0 mL, 0.2 M) was stirred under argon atmosphere for 14 hours at 50 °C to afford **66** (27.0 mg, 55% yield) and **66´**(12.9 mg, 26% yield) as colorless oil.

Analytical data of **66**:

^{1}H NMR (400 MHz, $CDCl_3$) δ 6.98 - 6.96 (m, 1H), 6.69 - 6.66 (m, 2H), 5.58 (br, 1H), 3.77 (s, 3H), 3.41 - 3.29 (m, 2H), 2.83 - 2.77 (m, 1H), 2.74 - 2.61 (m, 2H), 1.96 (s, 3H), 1.91 - 1.62 (m, 6H).

^{13}C NMR (101 MHz, $CDCl_3$) δ 170.2, 157.7, 141.4, 130.1, 129.4, 113.5, 111.9, 55.4, 37.9, 36.6, 35.8, 28.9, 27.7, 23.5, 20.2.

HRMS (ESI, *m*/*z*) calcd for $C_{15}H_{21}NO_2Na^+$ $[M+Na]^+$: 270.1465, found: 270.1461.

Analytical data of **66´**:

^{1}H NMR (400 MHz, $CDCl_3$) δ 7.10 - 7.06 (m, 1H), 7.00 - 6.96 (m, 2H), 5.67 (br, 1H), 3.72 - 3.66 (m, 1H), 3.52 - 3.41 (m, 5H), 3.04 (dd, J = 16.6, 5.1 Hz, 1H), 2.94 (dt, J = 16.8, 6.0 Hz, 1H), 2.80 (t, J = 7.1 Hz, 2H), 2.71 (dd, J = 16.5, 7.1 Hz, 1H), 2.12 - 2.03 (m, 2H), 1.96 (s, 3H), 1.85 - 1.76 (m, 1H).

^{13}C NMR (101 MHz, $CDCl_3$) δ 170.4, 137.3, 136.9, 133.1, 127.4, 127.0, 126.0, 76.1, 56.1, 39.61, 32.59, 32.4, 27.4, 27.2, 23.4.

HRMS (ESI, *m*/*z*) calcd for $C_{15}H_{21}NO_2Na^+$ $[M+Na]^+$: 270.1465, found: 270.1461.

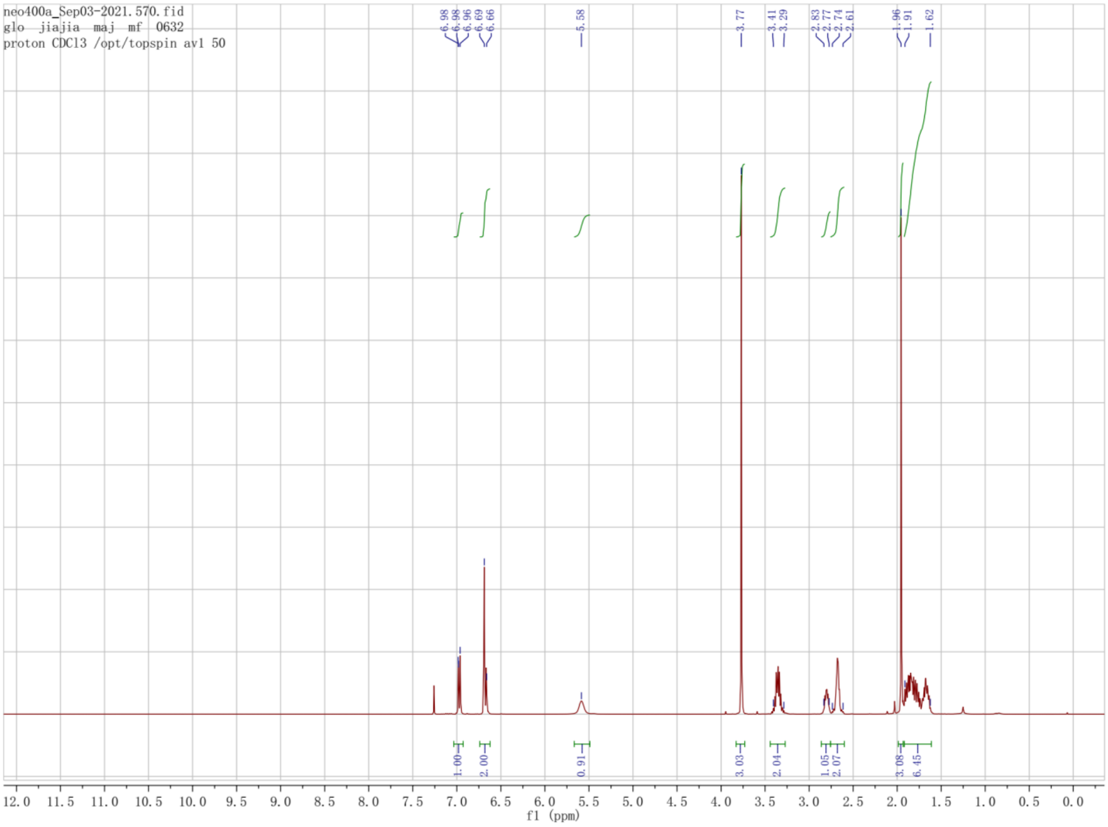
neo400a_Sep03-2021.570.fid
glo jiajia maj mf 0632
proton CDCl3 /opt/topspin av1 50
6.98
6.98
6.96
6.69
6.66
5.58
3.77
3.41
3.29
2.83
2.77
2.74
2.61
1.96
1.91
1.62
1.00
2.00
0.91
3.03
2.04
1.05
2.07
3.08
6.45
12.0 11.5 11.0 10.5 10.0 9.5 9.0 8.5 8.0 7.5 7.0 6.5 6.0 5.5 5.0 4.5 4.0 3.5 3.0 2.5 2.0 1.5 1.0 0.5 0.0
f1 (ppm)

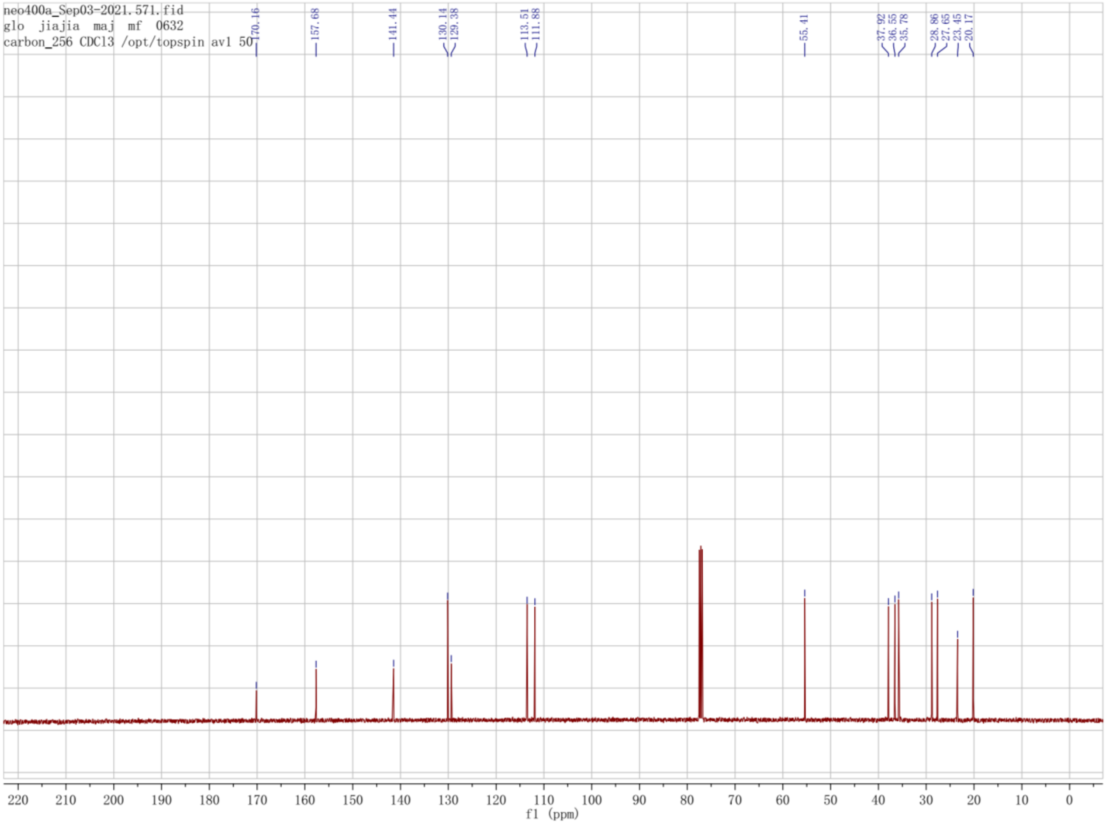
neo400a_Sep03-2021.571.fid
glo jiajia maj mf 0632
carbon_256 CDCl3 /opt/topspin av1 50
170.16
157.68
141.44
130.14
129.38
113.51
111.88
55.41
37.92
36.55
35.78
28.86
27.65
23.45
20.17
220 210 200 190 180 170 160 150 140 130 120 110 100 90 80 70 60 50 40 30 20 10 0
f1 (ppm)

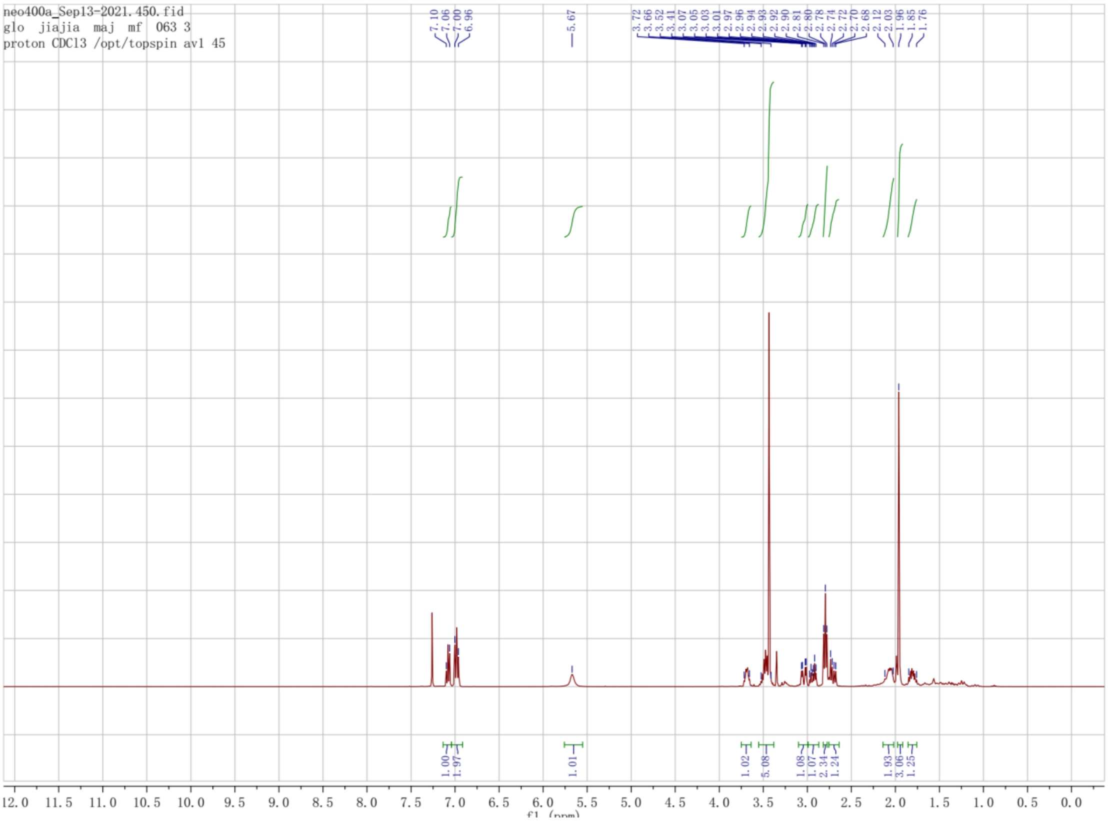
neo400a_Sep13-2021.450.fid
glo jiajia maj mf 063 3
proton CDCl3 /opt/topspin av1 45
7.10
7.06
7.00
6.96
5.67
3.72
3.66
3.52
3.41
3.07
3.05
3.03
3.01
2.97
2.96
2.94
2.93
2.92
2.90
2.81
2.80
2.78
2.74
2.72
2.70
2.68
2.12
2.03
1.96
1.85
1.76
1.00
1.97
1.01
1.02
5.08
1.08
1.07
2.34
1.24
1.93
3.06
1.25
f1 (ppm)

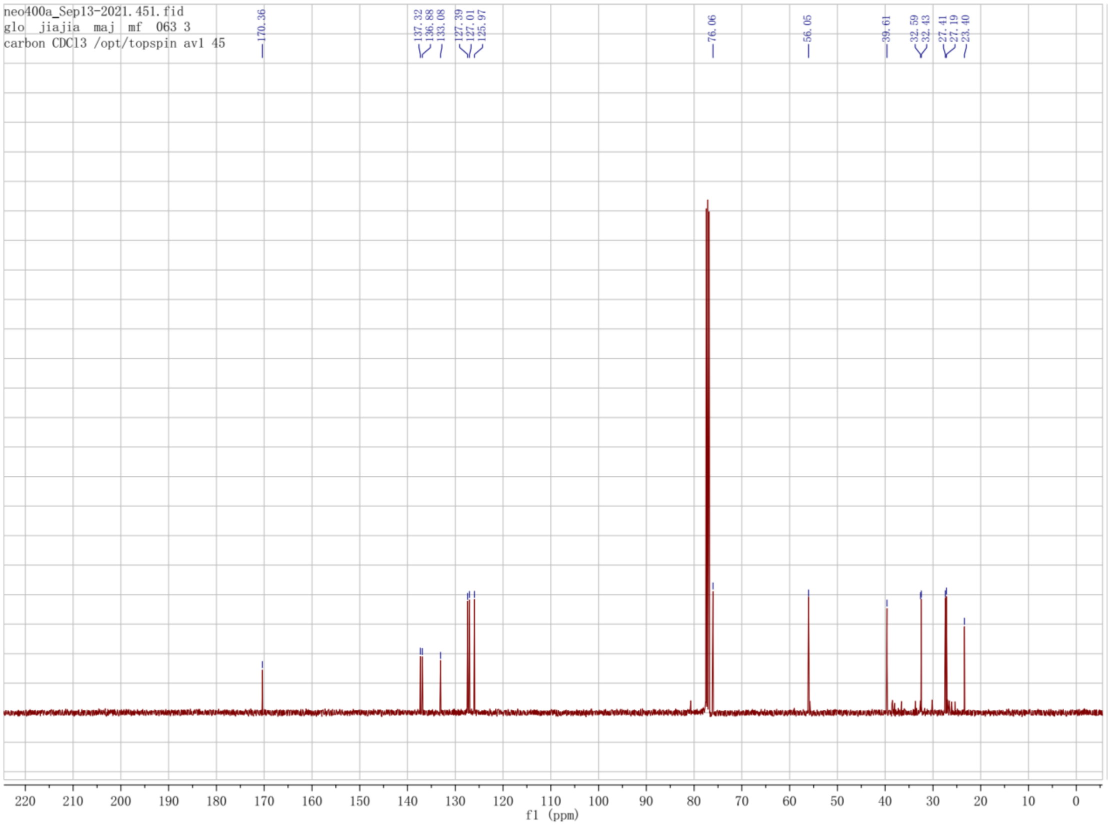
neo400a_Sep13-2021.451.fid
glo jiajia maj mf 063 3
carbon CDCl3 /opt/topspin av1 45
170.36
137.32
136.88
133.08
127.39
127.01
125.97
76.06
56.05
39.61
32.59
32.43
27.41
27.19
23.40
f1 (ppm)

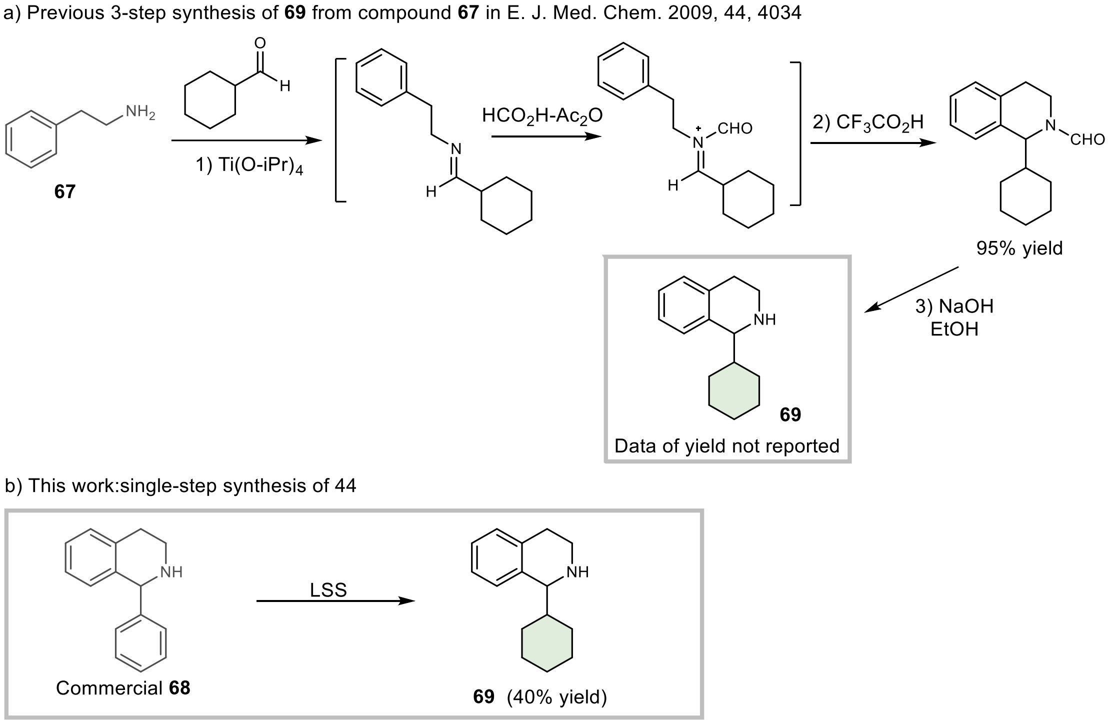

According to the general procedure, a mixture of **68** (41.8 mg, 0.20 mmol, 1.0 equiv.), $B_2(OH)_4$ (107.5 mg, 1.20 mmol, 6.0 equiv.) and $[Rh(COD)OH]_2$ (3.7 mg, 4.0 mol%, 0.008 mmol) in EtOH (1.0 mL, 0.2 M) was stirred under nitrogen atmosphere for 26 hours at 50 °C to afford **69** as a colorless oil (17.2 mg, 40% yield).

Purification conditions: NH_3(7.0 M solution in MeOH)/CH_2Cl_2 = 1:50 to 1:25

R_f = 0.5 in NH_3(7.0 M solution in MeOH)/CH_2Cl_2 = 1:10.

^{1}H NMR (400 MHz, $CDCl_3$) δ 7.19 - 7.03 (m, 4H), 3.91 (br, 1H), 3.32 - 3.22 (m, 1H), 2.98 - 2.89 (m, 1H), 2.88 - 2.78 (m, 1H), 2.73 - 2.63 (m, 1H), 1.98 - 1.88 (m, 1H), 1.83 (d, *J* = 11.6 Hz, 1H), 1.72 - 1.64 (m, 3H), 1.45 - 1.25 (m, 4H), 1.21 - 1.03 (m, 3H).

HRMS (ESI, *m*/*z*) calcd for $C_{15}H_{22}N^+$ $[M+H]^+$: 216.1752, found: 216.1750.

All the other data is consistent with a previous report.[6]

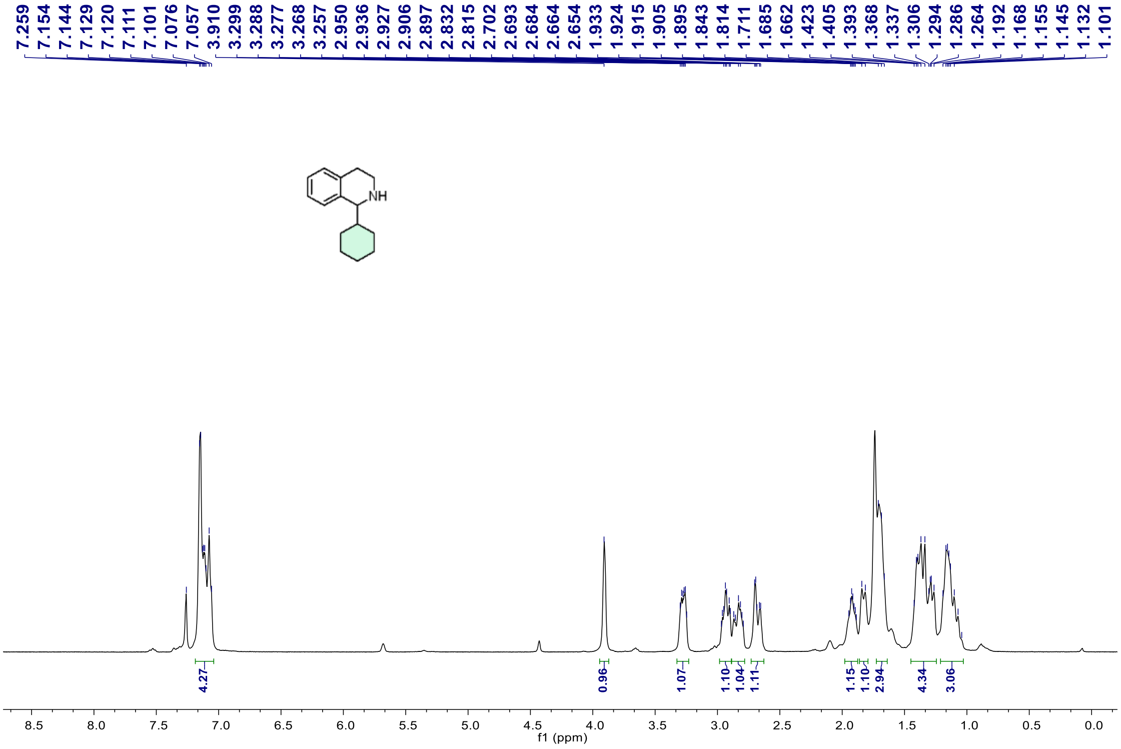
7.259
7.154
7.144
7.129
7.120
7.111
7.101
7.076
7.057
3.910
3.299
3.288
3.277
3.268
3.257
2.950
2.936
2.927
2.906
2.897
2.832
2.815
2.702
2.693
2.684
2.664
2.654
1.933
1.924
1.915
1.905
1.895
1.843
1.814
1.711
1.685
1.662
1.423
1.405
1.393
1.368
1.337
1.306
1.294
1.286
1.264
1.192
1.168
1.155
1.145
1.132
1.101
NH
4.27
0.96
1.07
1.10
1.04
1.11
1.15
1.10
2.94
4.34
3.06
f1 (ppm)

5. Data Guided Validation of the LSS Concept

All code and data are available for non-commercial purposes in the supplied repository:
zivgitlab.uni-muenster.de/ag-glorius/published-paper/Late-Stage-Skeleton-Saturation
All software is licenced under GNU General Public License 3:
www.gnu.org/licenses/gpl-3.0.html

5.1 Theoretical Background

By analysing data about current drugs and bioactive, druglike compounds the following research questions shall be answered:

1. How many examples can be found where a compound and its reduced analogue are both drugs or bioactive.
2. Is hydrogenation currently used to obtain new drugs or drug-like compounds from existing ones?
3. How much could libraries of drug, or drug-like compounds be extended by reduction of their aromatic moieties?
4. Does the drug likeness of a drug like compound is conserved if it gets reduced on its aromatic moieties such as benzene- and pyridines?

To answer these questions two datasets of drugs and candidates as well as bioactive compounds were acquired from publicly sources (5.2 Data Acquisition). These datasets of molecular structures were analysed by different custom-made *python* programs. Thereby, the first program (5.3.1 General Program Structure) is used to perform an *in-silico* reduction of structures to check these new artificial compounds for their presents in the original dataset. Thereby, pairs of compounds can be found which are both drugs or bioactive with one compound being (partially) reduced and one having an aromatic moiety. With these pairs of known compounds question 1 can be answered. To answer question 2, data (on drugs and pairs) is analysed for the occurrence of different aromatic patterns and substitution patterns (5.3.2 Data Analyzation). To assess the possibility to extend current libraries of drug-like compounds by the proposed LSS concept, thereby answering question 3, all possible products for reductions of aromatic moieties are constructed and counted (5.3.3 Library Expansion by LSS). Thereby, it was differentiated between (pseudo) regioisomers and potential stereoisomers. Evaluation was carried out on both, drug and bioactive compound data. Finally, an ADMET analysis was

conducted for all compounds synthesized and used within this work, to evaluate if drug-likeness is conserved over reduction (5.5 ADMET Analysis).

5.2 Data Acquisition

5.2.1 PubChem

To assess marketed drugs as well as compounds in clinical trials regarding their aromatic structures and substitution patterns, *PubChem* data was utilized.[7] Therefore, the *PubChem* TOC Tree was utilized and all compounds having *Drug and Medication Information* were downloaded (Aug. 2021) as *.sdf* file. Even though this also includes compounds in clinical trials or with drug warnings this dataset was chosen as it is publicly available covers compounds approved/tested in the EU, US as well as Japan. Therefore, it gives a broader view than corresponding databases focused on drugs approved in the US. Including compounds in clinical trials improves the actuality of data and results, since approved compounds typically have been developed and tested years ago. 17973 datapoints were downloaded.

5.2.2 ChEMBL

To assess bioactive compounds with drug-like properties regarding aromatic structures, and substitution patterns and to evaluate the LSS concept, *ChEMBL* data was utilized.[8] Data was downloaded (Aug. 2021) using the *ChEMBL* web service.[9] A filter was set to include small molecules only and data was downloaded in six *.sdf*-files. 1947973 datapoints were downloaded.

5.3 Validation of the LSS Concept (Program)

5.3.1 Finding of Drug/LSS Pairs

Program Structure

The designed python code (*Drug_LSS_Pair_Finder.py*) is constructed for finding pairs of compounds, in which one is equivalent to the other but reduced at one or more aromatic six-membered rings. These pairs are referred to as Drug/LSS pairs or LSS pairs for simplicity. It does so by constructing all potential (pseudo) regioisomers resulting from a reduction on any or multiple aromatic six-membered rings in the original structure. It only reduces full rings and does not take stereochemistry

into account. The program consists of the following major parts:

1. General functions
2. Variable initialization
3. Data loading & Cleaning
4. In silico hydrogenation
5. Pair finding

It is constructed to load *.sdf* files exported from *ChEMBL* (*ChemX.sdf*) and *Pubchem* (*Pubchem_drugs.sdf*) (5.2.1 PubChem, 5.2.2 ChEMBL) from its execution folder and saves the following files while running:

aromatics.json List of all compound SMILES found, which contain at least one aromatic six-membered ring and passed applied filters.

filtered.json List of all compound SMILES from the original dataset which passed applied filters.

full_hydro.json List of dictionaries each containing a SMILES from the *aromatics.json* list and a list of all corresponding (pseudo) regioisomer obtained *by in-silico* hydrogenation.

Hits.xlsx *Excel* file with all drug/LSS pairs found in the given dataset.

All above mentioned files are given for the utilized *Pubchem* and *ChEMBL* dataset. To apply the code on this data it can be renamed and placed in the execution folder of the code. In case new data shall be loaded none of the above-mentioned files should be present in the execution folder, as the program can recognize and load those rather that the raw data.

General Functions

General functions cover line 16 to 149 of the code.

find_drug_pairs_mp

This function takes a dictionary from the *full_hydro.json* like list as well as a list of SMILES strings as given in the *filtered.json*. For every (pseudo) regioisomer it checks if it is also present in the given list. Thereby, it can be used to find pairs of drug/LSS compounds. The function can be called in parallel e.g., by the unordered_imap" function from the multiprocessing library.

del_all

The function takes any list, and a list of integers. It deletes all elements in the first list, which indices are present in the second.

remove_heavy_molecules

From a given list of valid SMILES strings it calculates the molecular mass. If the mass is above a set threshold the SMILES will get removed from the list. If the function cannot generate a mol object or cannot calculate the mass the SMILES is not removed, as these cases will be covered in different functions. The main for loop of this function is wrapped by a progressbar, as it can take some minutes for large datasets (> 100k SMILES).

remove_salts

From a given list of SMILES this function splits every SMILES, consisting of two structures at the molecule separator ("."). It only keeps the largest SMILES, as the smaller are typically counterions as chlorine. As these counterions cannot efficiently be treated in the later program and are not necessary for evaluating the given hypothesis they can be removed. Charges, in the remaining molecular SMILES get removed, if they are related to protonation or deprotonation. The main for loop of this function is wrapped by a progressbar, as it can take some minutes for large datasets (> 100k SMILES).

neutralize_atoms

The function takes a *RDKit mol* object and removes charges, if they are related to protonation or deprotonation. It does so by utilizing SMARTS queries to check for any charged atom followed by changing the formal charge and hydrogen-atom-count on the respective atom. If the process fails (e.g. in case of a charge which is not caused by protonation or deprotonation), it returns the original molecule.

check_if_aromatic_bonds

The function takes and *RDKit mol* object and the index of any atom checks if the atom having this index has any aromatic bonds.

hydrogenate_matched_structure

This function takes a *RDKit mol* object, and a tuple of six integers which correspond to the atom indices of an aromatic six-membered ring structure within the molecule. The function iterates over

all combination of two indices and checks it there is a bond between them. If yes, it checks if this bond is aromatic and changes it to a single bond in this case. In case this atom is not part of an aromatic system anymore (i.e. has no aromatic bonds to its neighbors) its aromaticity state is set to *False*. The function returns the reduced molecule.

make_hydrogenated_structures_recursiv

The function takes a *RDKit mol* object, a list of tuples of six integers which correspond to the atom indices (*matches*) of an aromatic six-membered ring structure within the molecule, as well as a list of SMILES holding all already reduced structures. The function is recursiv and generates all potential pseudo regioisomers for a given molecule and given aromatic structures as defined in the *matches* variable. As some fused polycyclic ring systems cannot be reduced by this method, they are excluded. A SMARTS pattern is given to check if a molecule carries this substructure. If it is not present in the molecule the function iterates through all matches in the given list and generates the hydrogenated structure by utilizing the *hydrogenate_matched_structure* function. It converts it into a canonical SMILES and if it has never been generated before, it appends it to the list of already reduced structures, i.e., (pseudo) regioisomers. Then it copies the list of remaining aromatic structures and removes the match which was “hydrogenated" in this iteration. If matches are remaining it calls itself again. With this recursion it can generate all theoretical products of a hydrogenation reaction, i.e., all products if this reaction would be fully chemo unselective. It does not take symmetry constrains into account. However, symmetry related duplicates will be removed by removing all duplicate SMILES in the returned *hydro_smiles* list.

make_all_hydrogenated_structures_recursiv

The function takes a SMILES string, i.e. compound and generates all theoretically possible (pseudo) regioisomers. Therefore, a SMARTS query (e.g. *a1aaaaa1*) which queries for aromatic six-membered-ring structures is defined and applied on the molecule to obtain a list of matching atom indices (matches). With this it generates all possible pseudo regioisomers by calling the *make_hydrogenated_structures_recursiv* function. It removes all duplicates from the returned list of SMILES and return the final list.

make_all_hydrogenated_structures_recursiv

This function takes a list of SMILES strings i.e. compound and generates all theoretically possible (pseudo) regioisomers for every compound. It does so by calling the *make_all_hydrogenated_structures_recursiv* function and generates a new list of dictionaries with the original SMILES and a list of all hydrogenated structures. It is designed for serial use of the program. The main for loop of this function is wrapped by a progressbar, as it can take minutes to hours on large datasets (> 100k SMILES).

Variable Initializations

Variable initializations cover line 155 to 174 of the code.

Initially the database (*Pubchem* or *Chembl*) is defined which will be loaded and searched for drug/LSS pairs. Thereafter, the user can specify if later functions for pair searching shall be executed serially or in parallel. In addition, the number of cores can be set. By default, the program runs in parallel and utilizes all available cores.

In this section, the SMARTS is specified, utilized to find all aromatic six-membered ring structure, while the paths for output-files are defined. In addition, all utilized variables are initialized.

Data Loading & Cleaning

Data loading and cleaning covers line 177 to 244 of the code.

In this section of the code, the program loads data from given *.sdf* files of the defined database, cleans the data by filter operations and duplicate removal and saves final structures in two output files.

To avoid (pre)processing data in ever iteration of the program, the program checks if the *aromatics.json* file exists. If this is the case, it loads SMILES of aromatic structures from it from it as well data of all structures from the *filtered.json* file. In case these files are not present, it loads molecules present the *.sdf* file, using *RDKit*. In case of the *ChEMBL* data, it loads all six input files iteratively by altering the file name. A more elegant and general solution, e.g. utilizing the *pathlib* library and *glob* functions was tested, but failed based on the incompatibility of *RDKit* and *pathlib Path* objects. After loading the molecules into a list, this list is iterated through while all molecules are checked for readability, stereo information is removed and canonical SMILES are generated. These SMILES are added into the *all_SMILES* list. Then, the program uses the *remove_salts* function as well as the *remove_heavy_molecules* function (*mw* =800 Da) to clean the list further. Finally,

duplicates are removed by converting the list into a set and back. Every molecular structure in the resulting final list (*Filtered_SMILES*) is then tested for the prevalence of any aromatic six-membered ring structure by using the *RDKit* substructure match function *HasSubstrucutreMatch*. Every structure having this match is appended to a different list (aromatic_list). Both lists are saved as .json files.

In Silico Hydrogenation

In silico hydrogenation covers line 247 to 265 of the code.

This part of the code is designed to generate all possible (pseudo) regioisomers i.e. theoretical reaction products of a hydrogenation reaction for a list of molecules. Again, the program is able to load already generated data from the *full_hydro.json* file. It initially checks if this file is present, and loads it in this case. If not, the code generates all possible (pseudo) regioisomers for any SMILES in the previously generated *aromatic_list* by using the *make_all_hydrogenated_structures_for_all_molecules* function. The new list of dictionaries (*full_list_pseudo_regiostomers*) is iterated again to remove all duplicates potentially present in the lists of individual (pseudo) regioisomers. It saves the final list into the *full_hydro.json* file.

Pair Finding

Pair finding covers line 268 to 302 of the code.

To find pairs of drug/LSS compounds two functionalities are given. Due to the inherently scaling of this matching algorithm, the search can take days of calculation time for datasets of more than one million compounds, even on state-of-the-art hardware (i7-11800, 2.30 GHz base clock). To speed up the prediction, a parallelized function is given on side of the classical serial function. The serial function works by iterating through the *full_list_pseudo_regiostomers* list and checking for every SMILES of potential (pseudo) regioisomers if it is also present in the *Filtered_SMILES* list, which contains all cleaned structures from the initial database. If it finds it, it appends the initial aromatic SMILES and the theoretical reduction product to a list (*Hitlist*). In case of the parallelized function, a process pool is started using the *multiprocessing* library, and its *imap_unordered*. Thereby, the *partial* function is used to parse the *Filtered_SMILES* list to the *find_drug_pais_mp* function. Thereby, every process pool utilizes the *find_drug_pais_mp* function for one dictionary of the *full_list_pseudo_regiostomers* list. A chunk size of 50 is used as it was determined to speed up

calculations on the utilized Linux operating system. The function is wrapped into a progressbar to surveil remaining time. The results of every pair searching process is appended on the *allHitlist*, which is transformed to the *Hitlist*, by removing empty entries.

Cleaning and Saving

Cleaning and saving covers line 305 to 309 of the code.

Final results, as stored in the *Hitlist* is converted into a *pandas Dataframe* object, duplicates are removed and its saved as *Excel* file (*Hits.xlsx*).

5.3.2 Data Analyzation

Background

To evaluate if hydrogenation is already widely applied to extent the space of druglike or bioactive molecules the discovered drug/LSS pairs are analyzed further. Even though the low absolute number of pairs further (~0.5% of all bioactive molecules have a hydrogenated congener) gives evidence that the concept is not yet applied, the structures of aromatic six-membered rings as well as substitution patterns at benzylic moieties were examined. Thereby, analyses were carried out on the given *PubChem* data to get information about patterns in drugs as well as on the drug/LSS pairs found in the *ChEMBL* dataset. For *PubChem* all aromatic six-membered-ring structures were taken into account, while the altered i.e. hydrogenated moiety was examined in case of the LSS pairs. The comparison allows to draw conclusions about how saturated moieties are most likely introduced to druglike or bioactive compounds in case of found pairs. It also gives general information about underrepresented patterns and how LSS can be used to diversity druglike chemical space.

Program Structure

The python code (*Data_Analysation.py*) is designed to analyze the frequency of substructures at aromatic six-membered ring and the number of substituents at benzylic moieties. It utilizes the SMARTS language to query for aromatic six-membered rings and *mol* objects from the *RDKit* library, to compare and count substructures as well as to analyze ring substitution. Overall, four main analyses are performed:

1. Find and count all different six-membered ring patterns i.e. distinct (hetero)cycles which are different in a drug/LSS pair. This (hetero)cycle is the part which needs to be reduced to convert the drug into its LSS congener.
2. Find and count all different six-membered ring patterns i.e. distinct (hetero)cycles in all filtered PubChem compounds.
3. Count the number of substituents on the benzylic moiety of any a drug/LSS pair which differentiates both. This benzyl is the part which needs to be reduced to convert the drug into its LSS congener.
4. Count the number of substituents on all benzylic moiety in all filtered *PubChem* compounds.

The programs utilized the output files of the *Drug_LSS_Pair_Finder.py* code. It consists of four major

parts:

1. General functions
2. Data loading & conversion
3. Analysis of aromatic substructures
4. Substitution Pattern Analysis

After every of the four individual analysis an *Excel*-readable output-file is generated and saved in a *Statistics* folder in the execution directory:

1. *aromatic_substructure_paris.xlsx*
2. *aromatic_substructure_drugs.xlsx*
3. *substitution_ph_pairs.xlsx*
4. *substitution_ph_drugs.xlsx*

All above mentioned files are given for the utilized *Pubchem* compounds (2 & 4) and pairs found in the *ChEMBL* dataset (1 & 3). The code can be applied on every data output from the *Drug_LSS_Pair_Finder.py* program. If needed hard-coded SMARTS patterns (e.g. to analyze benzylic moieties) could be changes to account for other substructures. It has to be mentioned, that the code is only tested for cyclic aromatic structures and it is expected that changes need to be made to account for different patterns.

General Functions

General functions cover line 11 to 231 of the code.

find_all_different_pattern_in_pairs The function takes a *pandas Dataframe* holding SMILES of the LSS-pairs and finds and counts all different six-membered ring patterns i.e. distinct (hetero)cycles which are different in a LSS pair. It can operate on a *Dataframe* constructed from the *Hit.xlsx* and return a dictionary with the SMILES of all different (hetero)cylce as keys and their occurrence in the altered structure of the pair as values. It iterates through the given *Dataframe*, takes the SMILES from the *Arom* and *Hydro* column, find the differential pattern with the *find_hydrogenated_structure* function and alters the output dictionary accordingly. To monitor the function the main for loop is wrapped by a progressbar.

find_all_patterns

This function takes a *pandas Dataframe* holding SMILES (e.g. of drugs or druglike compounds) and counts all different aromatic six-membered ring patterns i.e. distinct (hetero) in its <u>*Arom*</u> column.

Therefore, it iterates over the *Dataframe* and finds all of its aromatic six-membered ring patterns similar to the function *find_all_different_patterns*. Finally, it alters the output dictionary accordingly. To monitor the function the main for loop is wrapped by a progressbar.

find_aromatic_structures

The function takes a single SMILES string and returns a list of all aromatic substrucutres present in it. Therefore, it finds all atom indices of aromatic six-membered ring structures by applying the *find_aromatic_patterns* function. These indices are wrapped into tuple of six integers (*match*), representing a ring. These matches are grouped into a list (*matches*). The function iterates through the tuple of those bags of 6 indices (*match*) and constructs a SMILES string from it by using the *construct_SMILES_from_match* function. As it is generated this SMILES string from scratch it is canonicalized by converting it into an *RDKit* mol object and back to a string. As this canonicalization fails in rare cases it is wrapped into a try/except statement. In any case a list of all SMILES, each representing one (hetero)cycle in the molecules is returned.

find_hydrogenated_structure_pair

This function is similar to the *find_aromatic_structures* function but works on a pair of SMILES differing in one six-membered ring. It is applied on the two SMILES strings of the LSS pair and returns a SMILES string of the differing structure. If two structures are different it returns *Multiple_rings*. Initially, it finds all aromatic six-membered ring matches, in the aromatic (drug) structure, as described before, then it performs an *in-silico* hydrogenation on the matched structure and checks if it is equivalent to the given LSS congener. If this is the case, it generates a SMILES for the matching substructure, as described before and returns it.

find_aromatic_patterns

This function takes a SMILES and a pattern, which is predefined to match aromatic six-membered ring structures. It converts the pattern into a SMARTS query and the SMILES into a *mol* object. If the conversion of the SMILES string was successful it matches the SMARTS on it and returns the matches.

hydrogenate_smiles_by_aromatic_match

The function takes a SMILES string of an molecules and a tuple of matches for any of its aromatic

structures and performs an *in-silico* hydrogenation on it. It first converts the SMILES to a mol object and after *in-silico* hydrogenation it reconstructs a SMILES string, which it returns.

hydrogenate_matched_structure

Equivalent to the function from *Drug_LSS_Pair_Finder.py*.

check_if_aromatic_bonds

Equivalent to the function from *Drug_LSS_Pair_Finder.py*.

construct_SMILES_from_match

This function takes any SMILES string and match of a simple ring-like substructure within this molecule and generates a SMILES string based on the substructure. Therefore, it initially converts the SMILES into an *mol* object and initializes an empty string as substructure SMILES. As, especially for aromatic rings, not only the atoms in the ring itself, but also potential neighbor atoms are important the function is able to scan these and adds ring closures, as well as branches to the AMILES. Core of this function is a while loop which is run through until all atoms in the ring are embedded in the substructure SMILES. It starts with the first atom-index in the given match, gets the *RDKit atom* object (*active atom*) from input molecules and obtains its atom symbol. This symbol represents the start of the SMILES fragment which will later be added to the substructure SMILES. The code checks if by adding this atom symbol to the SMILES any ring is closed and if this is the case (in later proceedings) it adds the number of the closed ring to the SMILES fragment. Then after, the code finds all neighbor indices of the active atom as well as neighbor being part of the match and neighbor which is not. For the later, it checks if it is bound to the active atom via a double bond and if it is the case they are added to a branch (in brackets, e.g. *(=O)*). Later this branch can be added to the SMILES fragment to give a valid SMILES. In case the active atom has neighbors, which are part of the match, its index is deleted from the match tuple and one of its neighbors becomes the new active atom (index). To account for ring closures, the function "opens" a ring the SMILES-fragment by adding the number of the ring to it. It then updates the *ring_closures* dictionary which stores all current unclosed rings and as every (open) ring needs an individual number, the *ring_closure_index* is counted by one. For the sake of clarity, it has to be mentioned, that in the *RDKit* SMILES nomenclature numbers for ring

closures can occur multiple times if they map fully independent ring systems. However, it is not necessary to take this into account, as the canonicalization algorithm will account for this. Finally, the function adds any potential branch to the SMILES fragment and adds it to the substructure SMILES. This procedure is repeated until all matched atom indices are accounted for. The final substructure SMILES is returned. For the sake of completeness, it must be said that the code does not include bonds in the ring and it outputs all patterns as formally saturated SMILES, although aromatic ones may have been checked for.

check_for_double_bond_to_atom_out_of_ring

This function takes a *mol* object, an atom index as integer and a list of neighbor indices. For every neighbor index it checks, if its bond to the atom index is a double bond. If yes, it returns *True*, otherwise *False*.

get_atom_symbol_of_double_neighbor

This function is similar to *check_for_double_bond_to_atom_out_of_ring,* but instead of returning a Boolean, this function returns the atom symbol of the atom symbol of the neighbor which is double bound to the original atom.

get_neighbors_in_match

For a given *rdkit atom* the function determines the indices of all neighbor atoms which are in a and a *match* (i.e. tuple of atom indices which correspond to any aromatic six-membered ring structure). Therefore, it takes the atom index and match and determines the indices of all neighboring atom (i.e., atoms which it is bond to). It iterates through those and checks if they are in the *match*. If not, their list index is saved in a list (i.e. *droplist*). Finally, all list indices in the drop list are removed from the list of neighbors, which is returned.

get_neighbor_indices

This function takes a *rdkit atom* and determines all neighboring atoms, followed by getting their index and returning a list of all neighbor atom indices.

count_substitutions

This function takes a *pandas Dataframe,* a SMARTS pattern of any aromatic ring-like structure as string and a column name on which it shall work. The pattern is predefined for a benzylic structure, while the column is predefined as *Arom*. It counts the number of substituents on every aromatic ring-like structure defined in the SMARTS and in all given SMILES in the defined column of the *Dataframe*. It stores this information in a dictionary of integers, wherein the key defines one number of substitutions, and the value describes how often this substitution number was found. First, the function initializes this substitution dictionary followed by iterating through the rows of the *Dataframe.* It extracts the SMILES string from every row and uses the function *count_substitutions_smiles* to count all substitutions in the corresponding molecule. The returned dictionary is added to the substitution dictionary by the *sum_substitution_dicts* function. To monitor the function the main for loop is wrapped by a progressbar.

count_substitutions_smiles

The function takes a SMILES string as well as a SMARTS pattern of any aromatic ring-like structure as string. First it initialized a substitution dictionary, followed by getting matches for the pattern and molecule. As every molecule can have multiple matches, the function iterates through the matches and counts the number of substitution patterns in each by the *get_number_of_substitutions* function. The returned value of the integer in the substitution dictionary is counted up and after all iterations the function returns the dictionary.

count_substitutions_in_all_pairs

The function is similar to the *count_substitutions* function, but operates not on one column of SMILES (i.e. structure) but on two columns holding both structures of the drug/LSS pairs. The drug or drug-like compound gets extracted from the *Arom* column, while the LSS compound is taken from the *Hyro* column. It calls the function *count_substitutions_in_pair* to get the number of substitutions. To monitor the function the main for loop is wrapped by a progressbar.

count_substitutions_in_pair

The function is similar to the *count_substitutions_smiles* but operates on the SMILES of both

compounds of any drug/LSS pair. Therefore, it initialized the substation dictionary followed by getting matches for the pattern in the aromatic SMILES. As only these structures can be the differencing substructure between both compounds the algorithms performs and in-silico hydrogenation on this structure and checks if the generated SMILES is equivalent to the given LSS-SMILES. If it is, it checks the number of substitutions on this match (i.e. ring) as in the *count_substitutions_smiles* function. If multiple rings are changed from the drug to the LSS congener, the function will not count the number of substituents.

get_number_of_substitutions

This function takes a SMILES string and a match (i.e. tuple of integers, corresponding to atom indices of matching atoms). It iterates through the atom indices in the match. For each it takes the *RDKit atom* object and determines the number of non-hydrogen neighbor atom (i.e. atoms bound to it). As every atom has two within the ring any atom having a third neighbor is, per definition substituted. In this case the *number_of_substitutions* variable is counted up. In case that more than three neighbors have been found, the function prints a warning.

count_value_of_key_up_by_one

This auxiliary function takes any dictionary with string keys and integer values as well as a key. It counts the value of this key up by one, or if the key is not present in the dictionary, it updates the dictionary by it. It removes the dictionary.

remove_list_from_list

This auxiliary function takes two lists and removes all elements which are in the second list from the first. It returns the cleaned list.

sum_substitution_dicts

This auxiliary function takes two dictionaries with integers keys and values (e.g. the substitution dictionaries). Keys have to be the same in both dictionaries It iterates though all keys of the second dictionary and adds up the values for every key. Theses sums are saved as new value for the current key in the first dictionary. It returns the adjusted first dictionary.

Data Loading

Data loading covers line 240 to 264 of the code.

As the program builds up on results from the *Drug_LSS_Pair_finder.py* it loads data from its output files. Therefore, paths for these files are defined firstly. Files need to be present in the execution folder. For data analysis, as done in this work, *aromatic.json*, *filtered.json* and *full_hydro_list.json* were generated based on the PubChem dataset, while the *Hits.xlsx* file was based on the previously described ChEMBL data. After defining paths, data from the *.json* files is loaded into variables by the *load* function from the *json* library. In addition, data from *.xlsx* files is loaded directly into a *Dataframe* object naming columns so that they match to default column names of major functions. Finally, the lists and dictionaries from the *.json* files are parsed into *Dataframe* objects, whereby columns are specified to match default column names of major functions.

Analysis of Aromatic Substructures (Drug/LSS Pairs)

Analysis of aromatic substructures of the drug/LSS pairs covers line 268 to 275 of the code.

To analyze the differential pattern in every LSS-pair, the *find_all_different_pattern_in_pairs* function is applied on the *Dataframe* of LSS pairs (*hit_df*). The returned dictionary of patterns and their absolute occurrence is then converted into a *pandas Dataframe* and saved in the *Statistics* subfolder. It is named *aromatic_substructures_pairs.xlsx* and has the SMILES of every substructure as column heading, and its frequency as value in the first and only row of the table.

Analysis of Aromatic Substructures (Drugs)

Analysis of aromatic substructures of the drug/LSS pairs covers line 277 to 281 of the code.

Similar to the analysis of differential pattern in every Drug/LSS-pair, aromatic substructures of drugs, as given by the PubChem dataset are analyzed by the *find_all_patterns* function. Therefore, the *aromatic_df* is utilized, as it contains only those structures which have aromatic six-membered ring moieties. The returned dictionary of patterns and their absolute occurrence is subsequently converted into a *pandas Dataframe* and saved in the *Statistics* subfolder. It is named *aromatic_substructures_drugs.xlsx* and has the SMILES of every substructure as column heading, and its frequency as value in the first and only row of the table.

Substitution Pattern Analysis (Drug/LSS Pairs)

Substitution pattern analysis of the drug/LSS pairs covers line 283 to 290 of the code.

To analyze the number of substitution on differential phenyl rings in every Drug/LSS-pair, the *count_substitutions_in_all_pairs* function is applied on the *Dataframe* of pairs (*hit_df*). The returned substitution dictionary is then converted into a *pandas Dataframe* and saved in the *Statistics* subfolder. It is named *substitutions_ph_pairs.xlsx* and has the number of substitutions as column heading, and its frequency as value in the first and only row of the table.

Substitution Pattern Analysis (Drugs)

Substitution pattern analysis of the drug/LSS pairs covers line 292 to 296 of the code.

To analyze the number of substitutions on differential phenyl rings of drugs, as given by the PubChem dataset are analyzed by the *count_substitutions* function. Therefore, the *aromatic_df* is utilized, as it contains only those structures which have aromatic six-membered ring moieties. The returned substitution dictionary is then converted into a *pandas Dataframe* and saved in the *Statistics* subfolder. It is named *substitutions_ph_drugs.xlsx* and has the number of substitutions as column heading, and its frequency as value in the first and only row of the table.

5.3.3 Library Expansion by LSS

Background

To evaluate if the LSS concept is theoretically suited to broadly extend current libraries of drugs or bioactive molecules it was determined how many saturated compounds can be obtained by reducing a single compound or library of compounds. Thereby two different possibilities have be accounted for:

a) *(Pseudo) Regioisomers* – potential products of a hydrogenation reaction which is chemoselective for six-membered ring (hetero)arenes but unselective regarding differentiating between those rings. By such a reaction, every possible combination of saturated and unsaturated rings is generated as product. In case of an unsymmetric structure, carrying three (*N*) arene structures (*A*, *B* and *C*), seven (2^N -1) potential products arise ($A_sB_{us}C_{us}$, $A_{us}B_sC_{us}$, $A_{us}B_{us}C_s$, $A_sB_sC_{us}$, $A_sB_{us}C_s$, $A_{us}B_sC_s$, $A_sB_sC_s$). For the sake of convenience those products will be described as pseudo regioisomers or regioisomers, not

accounting for stereo centers.

b) *Stereoisomers* – As six-membered ring (hetero)arenes can be substituted by more than one residual group, stereo centers are likely be generated within the process of saturation. Therefore, enantiomers as well as diastereomers arise. To determine their number rationally, different symmetry constrains need to be accounted for. I case of a fully unsymmetrical compound carrying M stereocenters, 2^N stereoisomers arise from one (pseudo) regioisomer. For the sake of convenience all products, arising from an unselective reaction, as described above, are named stereoisomers, accounting for (pseudo) regioisomers and arising stereo centers.

In general, the number of potential (pseudo) regioisomers and stereoisomers can only be determined if different molecules symmetries are accounted for. As it poses significant challenges to build a program, which accounts for all possible scenarios and different approach was chosen: The designed algorithm generates all possible products (pseudo regio- and stereoisomers) in a systematic fashion, without accounting for symmetry, and removes any duplicates (i.e. equivalent structures) afterwards by utilizing the *RDkit* SMILES canonicalization. As this algorithm gives a unique and unequivocal string for every molecule, duplicates emerged due to molecular symmetry will be filtered out. For the sake of clarity, it must be mentioned, that unstable or unreasonable product structures or isomers can arise from this approach, however as to the best of our knowledge, no sufficient algorithm exists for stability evaluation. The calculations can be understood as upper limit approximation and should give an overview about the magnitude of scaling. In addition, as current hydrogenation reactions show specific reactivity and selectivity not all products are accessible yet. However, this highly systematic and data-driven approach gives an indication about the potential to develop new, mild hydrogenation reaction with different, or broader selectivity and reactivity.

Program Structure

The developed program (*Library_Expension_by_LSS.py*) is designed to generate and count all potential (pseudo) regioisomers and stereoisomers for every molecule in a given file of drug or bioactive molecules. It works hierarchically by first generating all potential (pseudo) regioisomers followed by stereoisomers. For the former, all aromatic six-membered rings are identified and all permutations for saturated and unsaturated rings are generated. For these theoretical products all

potential stereocenters are identified and by generating all permutations of chirality as well as duplicate removal all possible products are generated. While the (pseudo) regioisomers are saved as SMILES, in case of the stereoisomers only the number of possibilites gets counted while the SMILES are discarded. As, in case of a large dataset of bioactive molecules, more than 1 B stereoisomeric structures will be generated, meaning that multiple terabytes of RAM would be needed if structures are saved within the program. The main functionality of the code (isomer generation) is parallelized to reduce runtime. In case of the serial function, which is also given, a runtime of multiple days is expected if the code is applied on more than 1 M molecules. A maximum of 20 stereocenters is treated by the program. In cases in which more than 20 stereocenters are generated by hydrogenation the isomers cannot be generated stability by the program. In these cases, more than 1 M structures have to be constructed, rendering runtime to exceed 20 to 30 min. In these cases it was observed that processes generated by parallelization become unstable and do not proceed, wherefore the program cannot be finished. The number of generated theoretical products, therefore potential to expend a given drug library, can be seen as a lower-limit approximation. In addition, the program does not account for cases in which a potential product is in the drug or bioactive compound itself (i.e. for LSS pairs).

The program can be run in parallel and serial mode. It loads the *Full_hydro_list.json* generated by the *Drug_LSS_Pair_Finder.py* code and generates *Library_expension_values.txt* and *Possible_regioisomers_chiral_information.json* as output files. The former contains information about all possible (pseudo) regio- and stereo isomers in a human-readable format, while the later contains product structures and numbers of potential stereoisomers. It consists of four major parts:

1. General functions
2. Data loading and program setup
3. (Pseudo) isomer construction
4. (Pseudo) isomer counting
5. Result saving

It can be run on data for the processed and filtered *PubChem* or *ChEMBL* dataset. For the later, even on high performance server hardware, hours of runtime are to be expected.

General Functions

General functions cover line 11 to 177 of the code.

load_json

The function loads a *.json* file from a given path and returns the object stored in it.

count_nr_of_hydro

The function takes a list of dictionaries holding information about all structures, (pseudo) regioisomers and stereoisomers (*drugs_and_hydro_dicts*). It iterates through the list, counts the number of (pseudo) regioisomers and adds it up. This number, i.e. the number of possible regioisomers generated from the given drugs or druglike molecules will be returned.

count_nr_of_isomers

The function is similar to *count_nr_of_hydro* but instead of counting the number of (pseudo) regioisomers it adds up the number of stereoisomers, as determined before.

find_all_atoms_for_chirality

The function takes a *RDKit mol* object as well as a list of *matches* i.e. indices of atoms. The list contains all atoms which have been altered by a simulated chemical reaction and which should be checked for chirality by the function. The function iterates through the matching indices, retrieves the *atom* object and checks for its degree. The degree gives the number of non-hydrogen neighbors. In case of the hydrogenation of an aromatic system, any atom with a degree of 3 can be treated as potential chiral center. As hydrogenation is, per definition, the addition of an hydrogen atom, a degree above 3 is not possible in this case, even though it would also lead to the formation of a chiral center. The function returns matching all atoms ids with a degree equal to 3.

add_stereo_information_to_mol

The function receives a *RDKit mol* object, a vector defining how chirality shall be set and a list of atoms for which chirality shall be defined. It iterates through the list of these atoms, setting its chirality as given by the *chirality_bit_vector*. It does so by using the *add_stereo_information_to_atom* function. Finally, it converts the *mol* object into a SMILES string and returns it.

constructs_stereoisomers_non_recursiv

The function receives a molecule and a list of atom indices which are potential stereo centers. The function constructs all potential stereoisomers for this molecule based on the given list of atom indices. As these indices correspond to atoms altered in a chemical reaction (i.e. hydrogenation) a new stereocenter can be formed for every given atom. Stereocenters at other atoms, previously formed are explicitly not accounted for. The function first determines the number of stereo centers (N), and the number of possibilities which is 2^N. Then it iterates over the number of possibilities and assigns a "bit vector" to every number. This "bit vector", i.e. list of Booleans can be seen as the final configuration of the molecules, as every Boolean corresponds to one specific atom having clockwise or anticlockwise chirality. The function constructs the chiral molecule based on this configuration using the *add_stereo_information_to_mol* function and appends its canonical SMILES string to a list. Duplicates SMILES get removed, to account for symmetry and the list is returned.

add_stereo_information_to_atom

The functions receives a molecule, atom index and Boolean. It sets the chirality of the given atom corresponding to the Boolean. *True* corresponds to counterclockwise and *False* to clockwise chirality.

constructs_all_potential_stereoisomers_on_rings_non_recursively

The function receives a molecule, as altered by a hydrogenation reaction as well as a list of atom indices on which the *in-silico* reaction was performed. Initially, the function searches for all atoms potentially being a stereo center wherefore it uses the *find_all_atoms_for_chirality* function. It generates a deepcopy of the molecule to keep the original structure untouched by the addition of stereo information and constructs all potential stereoisomers for the given structure by the *constructs_stereoisomers_non_recursiv* function. The list of SMILES corresponding to all potential isomers is returned.

hydrogenate_matched_structure

Equivalent to the function from *Drug_LSS_Pair_Finder.py*.

check_if_aromatic_bonds

Equivalent to the function from *Drug_LSS_Pair_Finder.py*.

make_hydrogenated_rings

The function receives a molecule to alter and a list of matching aromatic six-membered ring structures. Those are given as tuple of integers. The function iterates through all matches and hydrogenates the matching ring using the *hydrogenate_matched_structure* function.

get_matches_to_hydrogenate_lists

The function receives *matches* of all aromatic six-membered ring structures in a given molecule, given as list of tuples (similar as in the *make_hydrogenated_rings* function) as well as a “bit vector” of the same length. Positions in this “bit vector” correspond to the aromatic rings in a molecule while the Booleans determine if this structure shall be hydrogenated or not. The function iterates through the “bit vector” and if the Boolean is True, the function appends all indices of the corresponding matching tuple onto a list which is returned.

get_matches_to_hydrogenate

The function is similar to *get_matches_to_hydrogenate_lists* but it returns all aromatic six-membered ring structures to alter as list of tuples instead of one list holding all atom indices.

hydrogenate_with_stereo_non_recursive

This function can be considered as main function to generate all (pseudo) regio- and stereoisomer for a given molecule. It takes a *RDKit mol* object, all aromatic six-membered ring structures of this molecule as *matches* (i.e. a List of Tuples holding integers). In contrast to the functions given in the *Drug_LSS_Pair_Finder.py* program, this and lower-level functions do not require any recursion, wherefore they can be adjusted and debugged more efficiently, while also running more stable in cases of large molecules (i.e. deeper recursion). As some, rarely occurring polycyclic structures can still lead to wrongly hydrogenated and erogenous molecules, SMARTS queries are added which are applied on every molecule before in-silico hydrogenation. If the molecules are not containing these structures the number of aromatic six-membered rings (*N*) and number of possible (pseudo)

regioisomers (2^N-1) is determined. Similar to the generation of all stereoisomers the code iterates over this number of possible regioisomers and the corresponding loop counter (i.e. integer) is converted into a "bit vector". This "bit vector" is determines which rings shall be hydrogenated in-silico. To obtain the matches corresponding to these rings to be hydrogenated the functions *get_matches_to_hydrogenate* and *get_matches_to_hydrogenate_tuple* are used. After a deepcopy of the input molecule was made, the make_hydrogenated_rings function is used to obtain the product based on the *matches*. The molecule is converted to its canonical SMILES and if it has not been generated before (e.g. if the molecule is symmetrical and two configurations give the same product) the function *constructs_all_potential_stereoisomers_on_rings_non_recursively* is used to construct all stereoisomers possible for this product. The number of possibilities for these isomers is counted and a list of (pseudo) regioisomeric SMILES as well as the number of possible stereoisomers is returned.

convert_int_in_bin_vector

The function receives any integer and the length. The function constructs a "bit vector" i.e. a list of Booleans from a given integer and pads it to the given length. It constructs a string of bits using the *numpy binary_repr* function, initializes a list of *False* values and iterates through the letters of the string, thereby setting corresponding values in the list to True.

hydrogenate_with_stereo_non_recursive_wrapper

The function takes the SMILES string of a compound and constructs all possible (pseudo) regioisomers and stereoisomers by using the *hydrogenate_with_stereo_non_recursive* function. It generates a SMARTS query for aromatic six-membered ring structures and used *RDKit* to obtain matching structures. After calling the *hydrogenate_with_stereo_non_recursive* function it removes duplicates and returns a list of (pseudo) regioisomeric SMILES as well as the number of possible stereoisomers.

hydrogenate_with_stereo_all_drugs_non_recursive_for_mp

The function takes a dictionary of molecules and corresponding information as saved in entries of the *Full_hydro_list.json* file and extracts the compound SMILES from it. It calls the

hydrogenate_with_stereo_non_recursive_wrapper function to constructs all possible (pseudo) regioisomers and stereoisomers and writes these information back to the dictionary which it returns. The function is designed to be called by a parallelized function.

hydrogenate_with_stereo_all_drugs_non_recursive

The function works similar to *hydrogenate_with_stereo_all_drugs_non_recursive_for_mp* but takes a list of dictionaries and iterates through this list The function is designed for serial use.

Data loading & Program Setup

Data loading and program setup covers line 182 to 188 of the code.

In this section the user can specify if later functions for isomer construction shall be executed serially or in parallel. In addition, the number of cores can be set. By default, the program runs in parallel and utilizes all available cores. In addition, the path for loading the *Full_hydro_list.json* file is set to be in the parent folder relative to the execution path. The file is loaded by the *load_json* function.

(Pseudo) Isomer Construction

(Pseudo) isomer construction covers line 194 to 203 of the code.

If serial execution was chosen the *hydrogenate_with_stereo_all_drugs_non_recursive* function is called and the returned list, including information about (pseudo) regio- and stereoisomer, is written into the *chial_drug_and_hydro_dicts* variable. In case of the parallelized function, a process pool is started using the *multiprocessing* library, and its *imap_unordered* is utilized. Thereby, every process pool utilizes the *hydrogenate_with_stereo_all_drugs_non_recursive_for_mp* for one dictionary of the *drugs_and_hydro_dicts* list. The function is wrapped into a progressbar to surveil runtime.

(Pseudo) Isomer Counting

(Pseudo) isomer counting covers line 206 to 215 of the code.

To count the number of potential (pseudo) regio- and stereoisomers, as determined for every given molecule before, the *count_nr_of_hydro* and *count_nr_of_isomers* functions are utilized. To estimate the possibility for extending a library of druglike the returned number possibilities is divided by the number of input structures to obtain an expansion factor. The input molecules, originated from the

PubChem and *ChEMBL* datasets have been filtered for aromatic structures before, wherefore this factor is smaller if the whole library is accounted for.

Result Saving

Result saving covers line 218 to 236 of the code.

Expansion factors for as well as the absolute number of potential (pseudo) regio- and stereoisomers are saved into a *.txt* file (*Library_expension_values.txt*) while the final list of dictionaries carrying individual information and structures grouped by the original structure is saved as *.json* file (Possible_isomers_stereo_information.json).

5.4 Validation of the LSS Concept (Results)

5.4.1 LSS Pairs

To obtain data about the frequency of aromatic structures in the chosen databased for Bioactive and Drug(like) compounds the downloaded *PubChem* and *ChEMBL* dataset were analyses separately by the *Drug_LSS_Pair_Finder.py* program. Files, about filtered and duplicate free datasets, aromatic six-membered ring structures and LSS pairs can be found in the corresponding files which are named based on their type and origin database (e.g. *filtered_ChEMBL.json*).

Data

PubChem dataset

Table S2. Absolut and relative sizes for the datasets derived from *PubChem* data and numbers of found LSS pairs.

	Number of compounds	Relative size
Initial size	17973	218%
Filtered and duplicate free	8225	100%
Aromatic structures	5821	70.7%
LSS pairs	9	0.109%

ChEMBL dataset

Table S3. Absolut and relative sizes for the datasets derived from *ChEMBL* data and numbers of found LSS pairs.

	Number of compounds	Relative size
Initial size	1947973	115%
Filtered and duplicate free	1696767	100%
Aromatic structures	1568629	92.4%
LSS pairs	9704	0.572%

Conclusion

Analyzing the cleaned datasets, it is obvious to find, that the *PubChem* data contained remarkably more duplicates or filtered structures than the *ChEMBL* dataset. However, this observation can simply be explained by the fact, that *ChEMBL* is a curated and cleaned database, supposably without duplicates, while *PubChem* is composed on the basis of various dataset. These datasets contain different collections e.g. of approved drugs from different governmental agencies. As those have overlapping approvals the occurrence of duplicates is expectable. For *ChEMBL* the size reduction is majorly caused by compound structures unparsable for *RDKit* as well as compounds of sizes above 800 Da.

Further it can be found that aromatic structures, as queried by the code have a stronger frequency (+21.7%) in the cleaned *ChEMBL* dataset. Even though this seems unusual, it could be explained by the age of underlying data. As *ChEMBL* majorly includes compounds in the preclinical state it is expected that data is comparably new. Therefore, most of the molecules were generated after major advances in sp^2-sp^2 cross couplings have been made. As these allowed for the simple construction of large scaffolds, those reaction have become a major tool for medicinal chemistry. As the PubChem dataset also includes molecules which have been developed and initially testes (even approved) before the cross-coupling era the gap seams relatable.

In opposite to the overall high number of aromatic structures as found in the dataset the number of LSS-pairs is with 0.109% and 0.572% is outstandingly low. Therefore, it can be stated that the LSS concept is not yet applied for drug or library design. However, it was found, that compound pairs are more frequent (x 5.24) in the set of bioactive compounds. This can be explained by the typical lead optimization process, in which a compound gets altered (around a core structure) to improve affinity or specificity. Thereby, new targets are typically synthesized from an intermediate compound, again not by utilizing the LSS concept.

5.4.2 Aromatic Patterns in Drugs and Pairs

To follow the hypophysis that current LSS pairs arise from convergent synthesis based on a synthesis intermediate, patterns within the pairs and within drug structures where analyzed. Therefore, data on the pairs found in the *ChEMBL* database and drugs as in the clean *PubChem* dataset was analyzed. Data can be found in the given repository as *.xlsx* files in the *Statistics* folder.

In case of the drugs from the *PubChem* dataset, every aromatic six-membered ring structure was analyzed and counted. On average 1.89 rings were found per compound. In case of the found LSS pairs, only the differing structure, i.e. moiety saturated in one and aromatic in the other compound was taken into account. In 276 cases (2.8%) two structures have been found to be different. In these cases, no aromatic structures where analyzed. Those examples are counted as own group.

Data

Distribution of Six-Memberd Aromatic Patterns in Marketed Drugs

Table S4. Top 10 most common aromatic patterns and SMILES in the dataset of *PubChem* drugs. 10981 patterns in 5821 compounds where examined.

**Rank**	**Pattern**	**SMILES**	**Relative Frequency**	**Absolute Frequency**
**1**	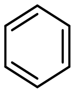	c1ccccc1	74.5%	8178
**2**	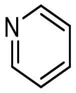	c1ccncc1	10.8%	1185
**3**	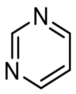	c1cncnc1	5.66%	621
**4**	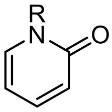	O=c1ccccn1	1.28%	141
**5**	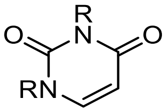	O=c1ccnc(=O)n1	1.25%	137
**6**	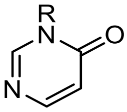	O=c1ccncn1	1.11%	122
**7**	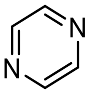	c1cnccn1	1.05%	115
**8**	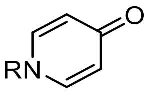	O=c1ccncc1	0.701%	77
**9**	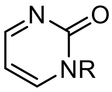	O=c1ncccn1	0.610%	67
**10**	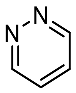	c1ccnnc1	0.510%	56

Number of different Patterns: **48**

Distribution of Six-Memberd Aromatic Patterns in LSS Pairs

Table S5. Top 10 most common, formally reduced, i.e. altered aromatic patterns and SMILES of LSS pairs in the *ChEMBL* dataset. 9704 patterns in 9704 compounds where examined.

**Rank**	**Pattern**	**SMILES**	**Relative Frequency**	**Absolute Frequency**
**1**	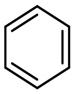	c1ccccc1	88.0%	8286
**2**	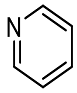	c1ccncc1	5.96%	562
**3**	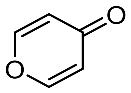	O=c1ccocc1	1.93%	182
**4**	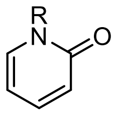	O=c1ccccn1	1.39%	131
**5**	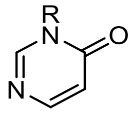	O=c1ccncn1	0.763%	72
**6**	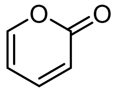	O=c1ccccо1	0.509%	48
**7**	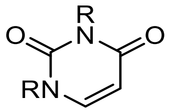	O=c1ccnc(=O)n1	0.414%	39
**8**	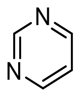	c1cncnc1	0.339%	32
**9**	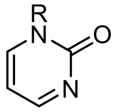	O=c1ncccn1	0.212%	20
**10**	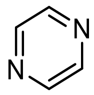	c1cnccn1	0.170%	16

Number of different Patterns: **23**

Conclusion

In general, major difference can be found between the distribution of aromatic six-membered ring structures in drugs and those which are formally reduced in the found LSS-pairs. An obvious observation is, that the diversity of patterns is significantly higher in case of all structures, where 48 different moieties have been identified. In contrast to this, in case of the altered structure in the LSS pairs only 23 different moieties have been identified. This trend is also visible if the top 3 structures are compared. While in both cases benzene can be found as number one structure, it accounts only for 74.5% in case of the drugs, while it accounts for 88.0% of all altered structures in found pairs. One reason for this observation could be, that the introduction of cyclohexyl, as replacement for benzene is a common step in lead-optimization. Thereby, a derivation is typically made in the periphery of the lead to improve interaction and thereby affinity to the target. The opposite observation can be found for pyridine. While it accounts for over 10% of all aromatic structures, it can only be found as changed structure in ~6% of all pairs. However, as piperidines are found to be even more common in drugs than the highly accessible pyridine this finding is surprising.[10] Based on this, it is reasonable to assume that the LSS concept is not utilized widely, as in this case the pyridine would become a target of high interest. Also, it shows the high potential of mild, pyridine reducing reactions for the construction of drug libraries and for optimizing lead compounds. For other patterns similar trend can be found, however it has to be mentioned, that in some cases (e.g. the pyrimidine), products of a hydrogenation reaction are often not stable against hydrolysis wherefore the concept could not be applied widely. Overall, it can be concluded, that the LSS concept is not applied systematically, however is of major interest to convert the current pyridine spaces into a piperidine one.

5.4.3 Substitution Patterns on Arenes in Drugs and Pairs

LSS Pairs

To follow the hypophysis that current LSS pairs arise from convergent synthesis based on a synthesis intermediate, the number of substitutions on arene moieties within the pairs and within drug structures where analyzed. Therefore, data on the pairs found in the *ChEMBL* database and drugs as in the clean *PubChem* dataset was analyzed. Data can be found in the given repository as *.xlsx* files in the *Statistics* folder. As it accounts for the majority of aromatic structures as well as LSS moieties, benzene was chosen as representative group.

In case of the drugs from the *PubChem* dataset, every arene structure was analyzed and counted. In case of the found LSS pairs, only the altered benzene/cyclohexyl structure was considered. In 276 cases (2.8%) two or more structures have been found to be different. In these cases, no aromatic structures where analyzed. Those examples are counted as own group.

Data

Number of Substitution on Arenes in Drug and on in LSS Pairs

Table S6. Number of substitutions on arenes in *Pubchem* drugs and altered arenes in LSS pairs from *ChEMBL*. 8203 (drugs) and 8385 (pairs) arene patters where analyzed.

Number of Substitutions	Relative Frequency (Drugs)	Absolute Frequency (Drugs)	Rank (Pairs)	Relative Frequency (Pairs)	Absolute Frequency (Pairs)	Rank (Pairs)
**1**	16.4%	1345	3	83.6%	7008	1
**2**	39.2%	3217	1	12.2%	1019	2
**3**	29.3%	2402	2	3.74%	314	3
**4**	11.7%	959	4	0.179%	15	4
**5**	2.16%	177	5	0.155%	13	5
**6**	1.24%	102	6	0.179%	15	6

Conclusion

While in the analyses drugs the majority of benzene structures is at least disubstituted (83.6%) in case of altered arenes in the found pairs this pattern is full inverted and multisubstituted structures account only for the minority (16.4%). There, 83.6% of all alteration are done on monosubstituted, peripheral

benzyls. Taking also disubstituted structures as periphery into account, in less than 5% of all pairs a core structure has been formally reduced as cyclohexyl cores could be of high interest, do to their ability to cover diverse 3D spaces, the absence of corresponding pairs clearly shows that the LSS concept, thereby mild arene hydrogenation is not yet applied in pharmaceutical research. Again, the results clearly show the current lead optimization process, in which alterations are made in the periphery of compounds while interesting core scaffolds stay untouched. This peripheric changes are supposed to be done by convergent synthesis, starting from a common synthetic intermediate and coupling it with different reagents. However, as polysubstituted arenes form the majority of structures within drugs a direct approach to obtain sp^3 enriched and altered compounds could be of major interest for lead optimization, as well as library design. As the LSS-concept enables both, changes in the periphery and at cores it is reasonable to assume that it accelerates drug discovery and optimization processes.

5.4.4 Library Expansion by LSS

To investigate the potential to expand current sp^2 rich drug and druglike compound libraries by the means of the LSS concept, potential (pseudo) regioisomers and stereoisomers for a formal reduction of aromatic six-membered ring moieties were counted. Therefore, the *Library_Expension_by_LSS.py* code was applied on the *aromatics.json* files as generated before and results where saves as *.txt* file. As it was applied on both, the *ChEMBL* and *PubChem* dataset two sets of result files can be found, named by their origin database. As the initially loaded datafile accounts only for drugs having aromatic six-membered ring moieties, multiplication values have to be seen as possible expansion for a library of aromatic compounds or need to be recalculated.

Data

Table S7. Number of potential (pseudo) regioisomers and stereoisomers obtainable by applying the LSS concept on current drug or bioactive space.

	PubChem (aromatic only)	PubChem (all)	ChEMBL (aromatic only)	ChEMBL (all)
Compounds	5821	8225	1568629	1696767
(Pseudo) regioisomers	20 443		6965946	
Scale-up factor (regioisomers)	x3.51	x2.48	x4.44	x4.11
Stereoisomers	2393957		1554304971	
Scale-up factor (stereoisomers)	x411	x291	x990	x916

Conclusion

As expected, the potential to diversify a given compounds library by the LSS concept is astonishing. If only accounted for (pseudo) regioisomers, it is possible to scale up the current drug space by a factor of 2.48 while for the current space of bioactive compounds an even greater scaling of 4.11 is expected. This comes unsurprisingly, as complexity of tested compounds raised in the past decades while also more aromatic moieties got introduced. One major advantage of saturated ring-structures

however is their coverage of specific and tunable 3D-spaces which allows drugs to be highly specific against the aimed target. This 3D-strucutre is represented by the number of potential stereoisomers, which could be accessed by utilizing the LSS concept. In case of the *PubChem* drugs over 2 M products could be accessed from the initial 8225 compounds leading to a scaleup of 291. In case of the more complex bioactive compounds extracted from *ChEMBL* each compound could give more than 916 potential products, thereby extending the space to 1.5 B compounds. Even though, it is clear that not all of these products are stable under biological conditions, neither can they be generated by current methods, current strategies allow to generate specific product isomers selectively.[11,12] In addition, current trends to optimize reactions for generality allow to improve synthetic methodologies thereby unlocking large fractions of this LSS space.[13] The authors expect that this potential will trigger the interests of synthesis chemistry to shift towards new hydrogenation reactivities.

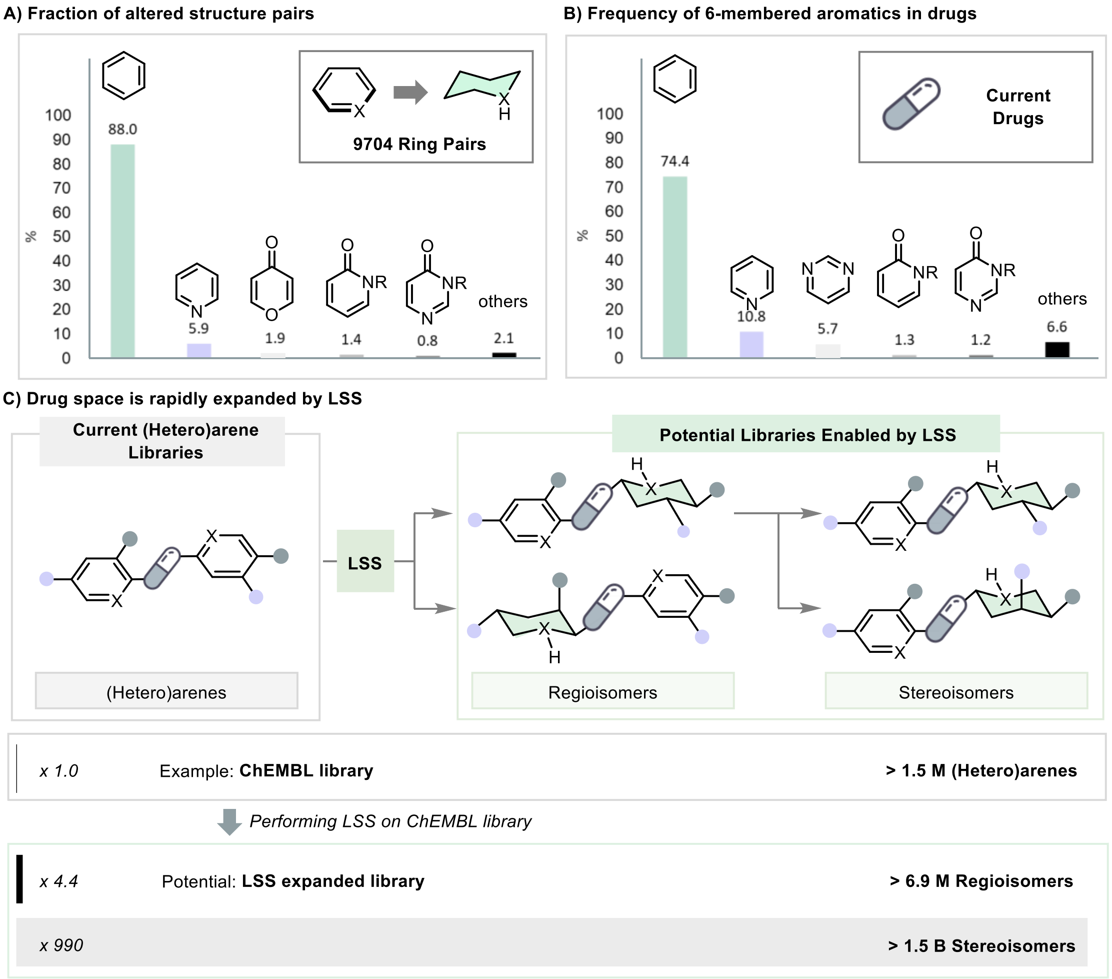

Figure S3 Data-guided validation of the LSS concept. A) Fraction of altered structure pairs; B) Frequency of 6-membered aromatics in drugs. C) Drug space is rapidly expanded owing to the generation of regioisomers and stereoisomers.

5.5 ADMET Analysis

5.5.1 Background

Goal of the ADMET analysis was to validate the hypothesis that most properties and therefore drug likeness of compounds is conserved by LSS transformations. Therefore, different parameters were selected to cover the whole process from drug absorption over metabolism to toxicity. Since a broad variety of parameters could be chosen for this process, a subset was selected based on straight forward and robust predictability by algorithmic and machine learning solutions. The following parameters have been calculated and considered:

- Fraction of C(sp^3)-Atoms
- Topological Polar Surface Area (TPSA/$Å^2$)
- Octanol/Water Partition Coefficient (Log $P_{o/w}$)
- Solubility in Water (mg/ml)
- Gastrointestinal Absorption Class
- Blood Brain Barrier Permeability Class (BBB)
- Inhibition of Cytochrome P450 Enzymes
  - CYP1A2 Inhibition
  - CYP 3A4 Inhibition
  - CYP 2C9 Inhibition
  - CYP 2C19 Inhibition
  - CYP 2D6 Inhibition
- Pan Assay Interference Structures (PAINS)
- Brenk Structural Alerts
- Median Lethal Concentration (LD_{50}/$Log_{mmol/kg}$)

Even though it was considered to calculate these properties for all marketed drugs and their potential LSS analogues, in this case I would have become necessary to predict it for the full expanded LSS-Drug-Library of >2 M compounds. As this would cause unreasonable computational costs and unbearable time burdens, properties were predicted for the presented LSS-scope and corresponding starting materials (i.e. drugs).

5.5.2 Property and Activity prediction

5.5.2.1 Data Preparation

Molecular structures of initial drugs and all isolated LSS analogues were drawn and exported as SMILES string. Thereby, any stereo information present in the previous drug was considered, while newly formed stereocenters were kept as undetermined. If multiple products were formed from one drug all products were considered, while the drug was only inputted once. Structure pairs have been considered if at least one LSS-product has been successfully synthesized from a commercial drug.

Table S8. Smiles strings of drugs utilized in the conducted property predictions.

Molecule	SMILES String
**Aminacrine**	NC1=C2C(C=CC=C2)=NC3=CC=CC=C31
**Adiphenine**	O=C(OCCN(CC)CC)C(C1=CC=CC=C1)C2=CC=CC=C2
**Pridinol**	OC(C1=CC=CC=C1)(C2=CC=CC=C2)CCN3CCCCC3
**Primidone**	O=C(NCN1)C(CC)(C2=CC=CC=C2)C1=O
**Atenolol**	O=C(CC1=CC=C(OCC(CNC(C)C)O)C=C1)N
**Diethyltoluamide**	O=C(N(CC)CC)C1=CC=CC(C)=C1
**Gemfibrozil**	O=C(C(CCCOC1=CC(C)=CC=C1C)(C)C)O
**Praziquantel**	O=C(C1CCCCC1)N(CC2N3CCC4=C2C=CC=C4)CC3=O
**Prilocaine**	O=C(C(NCCC)C)NC1=CC=CC=C1C
**Imipramine**	CN(C)CCCN1C2=CC=CC=C2CCC3=C1C=CC=C3

**Boc-Maprotiline**	CN(C(OC(C)(C)C)=O)CCCC12C3=CC=CC=C3C(CC2)C4=C1C=CC=C4
**Indoprofen**	O=C(C(C=CC=C1)=C1C2)N2C3=CC=C(C(C)C(O)=O)C=C3
**Flurbiprofen**	FC1=CC(C(C)C(O)=O)=CC=C1C2=CC=CC=C2
**Nabumetone**	O=C(C)CCC1=CC2=CC=C(OC)C=C2C=C1
**(S)-Naproxen-OMe**	O=C([C@H](C1=CC2=CC=C(OC)C=C2C=C1)C)OC
**Naftopidil**	OC(COC1=CC=CC2=C1C=CC=C2)CN3CCN(C4=C(OC)C=CC=C4)CC3
**Sacubitril**	O=C(CCC(O)=O)N[C@@H](C[C@H](C)C(OC)=O)CC(C=C1)=CC=C1C2=CC=CC=C2
**Benfluorex**	O=C(OCCNC(CC1=CC(C(F)(F)F)=CC=C1)C)C2=CC=CC=C2
**(R)-Tolterodine**	OC(C=CC(C)=C1)=C1[C@H](CCN(C(C)C)C(C)C)C2=CC=CC=C2
**Phenylethyl resorcinol**	OC(C=C(O)C=C1)=C1C(C)C2=CC=CC=C2
**Boc-Atomoxetine**	CC(C=CC=C1)=C1O[C@@H](CCN(C)C(OC(C)(C)C)=O)C2=CC=CC=C2
**Butenafine**	CN(CC1=CC=C(C(C)(C)C)C=C1)CC2=CC=CC3=C2C=CC=C3

**Cinacalcet**	CC(C1=CC=CC2=C1C=CC=C2)NCCCC3=CC=CC(C(F)(F)F)=C3
**Propranolol**	OC(CNC(C)C)COC1=CC=CC2=CC=CC=C12
**Midostaurin**	[H][C@@]1(C[C@@H](N(C)C(C2=CC=CC=C2)=O)[C@H]3OC)N(C4=C5C=CC=C4)C6=C5C(CNC7=O)=C7C8=C6N([C@@]3(C)O1)C9=C8C=CC=C9
**Pheniramine**	CN(CCC(C1=CC=CC=C1)C2=CC=CC=N2)C
**Doxylamine**	CC(C1=CC=CC=N1)(C2=CC=CC=C2)OCCN(C)C
**Atazanavir**	COC(N[C@H](C(N[C@@H](CC1=CC=CC=C1)C(O)CN(CC2=CC=C(C3=NC=CC=C3)C=C2)NC([C@H](C(C)(C)C)NC(OC)=O)=O)=O)C(C)(C)C)=O
**Tropicamide**	O=C(N(CC)CC1=CC=NC=C1)C(CO)C2=CC=CC=C2
**Melatonin**	O=C(C)NCCC1=CNC2=CC=C(OC)C=C21
**Zolmitriptan**	O=C1OC[C@H](CC2=CC=C(NC=C3CCN(C)C)C3=C2)N1
**Ketoprofen**	O=C(C1=CC=CC(C(C)C(O)=O)=C1)C2=CC=CC=C2
**Agomelatine**	COC1=CC=C2C(C(CCNC(C)=O)=CC=C2)=C1
**Cicloprofen**	CC(C(O)=O)C1=CC(C2)=C(C=C1)C3=C2C=CC=C3
**62**	COC1=CC(C2=CC=CC=C2)=CC(CC(O)=O)=C1
**Biprofen**	CC(C(O)=O)C(C=C1)=CC=C1C2=CC=CC=C2
**68**	C12=CC=CC=C1CCNC2C3=CC=CC=C3

Table S9. Smiles strings of LSS-drug analogues utilized in the conducted property predictions.

Molecule	SMILES String
**9**	NC1=C2C(CCCC2)=NC3=C1CCCC3
**Tacrine**	NC1=C(CCCC2)C2=NC3=C1C=CC=C3
**Drofenine**	O=C(OCCN(CC)CC)C(C1=CC=CC=C1)C2CCCCC2
**Trihexyl phenidyl**	OC(C1=CC=CC=C1)(CCN2CCCCC2)C3CCCCC3
**32**	O=C(NCN1)C(CC)(C2CCCCC2)C1=O
**21**	O=C(CC1CCC(CC1)OCC(CNC(C)C)O)N
**19**	O=C(N(CC)CC)C1CC(C)CCC1
**17**	O=C(C(CCCOC1C(C)CCC(C)C1)(C)C)O
**25**	O=C(C1CCCCC1)N(CC2N3CCC4C2CCCC4)CC3=O
**15**	O=C(C(NCCC)C)NC1CCCCC1C
**35**	CN(C)CCCN1C2CCCCC2CCC3=C1C=CC=C3
**26**	CN(C(OC(C)(C)C)=O)CCCC12C3CCCCC3C(CC2)C4=C1C=CC=C4
**37**	O=C(C(CCCC1)C1C2)N2C3=CC=C(C(C)C(O)=O)C=C3
**27**	FC1=CC(C(C)C(O)=O)=CC=C1C2CCCCC2
**41**	O=C(C)CCC1CCC2=CC(OC)=CC=C2C1
**42**	O=C(C)CCC1=CC2=C(CC(CC2)OC)C=C1
**44**	O=C(C(C1CCC2=CC(OC)=CC=C2C1)C)OC
**45**	O=C(C(C1=CC2=C(CC(CC2)OC)C=C1)C)OC
**28**	OC(COC1=CC=CC2=C1CCCC2)CN3CCN(C4=C(OC)C=CC=C4)CC3
**70**	O=C(CCC(O)=O)N[C@@H](C[C@H](C)C(OC)=O)CC(C=C1)=CC=C1C2CCCCC2
**30**	O=C(C1CCCCC1)OCCNC(C)CC2=CC(C(F)(F)F)=CC=C2
**29**	OC(C=CC(C)=C1)=C1[C@H](CCN(C(C)C)C(C)C)C2CCCCC2
**34**	OC(C=C(O)C=C1)=C1C(C)C2CCCCC2
**31**	CC(C=CC=C1)=C1O[C@@H](CCN(C)C(OC(C)(C)C)=O)C2CCCCC2

**33**	CN(CC1=CC=C(C(C)(C)C)C=C1)CC2=CC=CC3=C2CCCC3
**39**	CC(C1=CC=CC2=C1CCCC2)NCCCC3=CC=CC(C(F)(F)F)=C3
**23'**	OC(CNC(C)C)COC1=CC=CC2=C1CCCC2
**23**	OC(CNC(C)C)COC1CCCC2CCCCC12
**38**	[H][C@@]1(C[C@@H](N(C)C(C2CCCCC2)=O)[C@H]3OC)N(C4=C5C=CC=C4)C6=C5C(CNC7=O)=C7C8=C6N([C@@]3(C)O1)C9=C8C=CC=C9
**72**	CN(CCC(C1=CC=CC=C1)C2CCCCN2)C
**71**	CC(C1CCCCN1)(C2=CC=CC=C2)OCCN(C)C
**74**	COC(N[C@H](C(N[C@@H](CC1=CC=CC=C1)C(O)CN(CC2=CC=C(C3NCCCC3)C=C2)NC([C@H](C(C)(C)C)NC(OC)=O)=O)=O)C(C)(C)C)=O
**73**	O=C(N(CC)CC1CCNCC1)C(CO)C2=CC=CC=C2
**46**	O=C(C)NCCC1CNC2=C1C=C(C=C2)OC
**47**	O=C1OC[C@H](CC2=CC(C(CN3)CCN(C)C)=C3C=C2)N1
**51**	CC(C(O)=O)C1=CC(CC2CCCCC2)=CC=C1
**66**	COC1=CC2=C(C=C1)CCCC2CCNC(C)=O
**54**	CC(C(O)=O)C1=CC2=C(C=C1)C3CCCCC3C2
**59**	COC1=CC(C2CCCCC2)=CC(CC(O)=O)=C1
**61**	CC(C(O)=O)C(C=C1)=CC=C1C2CCCCC2
**63**	CC(C(O)=O)C(CC1)CCC1C2=CC=CC=C2
**69**	C12=CC=CC=C1CCNC2C3CCCCC3

5.5.2.2 Swiss ADMET

SwissADME was used for the prediction of all parameters except Median Lethal Concentration ($LD_{50}/\log_{mmol/kg}$).[14] Therefore, list of given SMILES for drugs and their LSS analogues were copied individually in the interface of the corresponding website (http://www.swissadme.ch) followed by exporting data as .csv. Both raw data files are supplied and can be found as *Drug_swiss_ADME.csv* and *Hydro_swiss_ADME.csv*.

4.5.2.3 Toxicology Prediction

Background

For the prediction of toxicological data, namely the Median Lethal Concentration (LD_{50}) a standalone machine learning model was designed and trained. Therefore, data was concatenated from various sources and standardized. With this data a gradient boosting model (*CatBoost*) was trained and the model was evaluated using 5-fold cross-validation as well as on an external benchmark from the *CATMoS* suite. Data splitting, training, evaluation and deployment was executed utilizing the *EasyChemML* toolbox. The script applied for this process via *EasyChemML* is supplied as *Train_Tox_Models.py*.

Data

Training data was collected from different sources namely the *US National Institute of Health (NIH), European Chemical Agency (ECHA), QSAR Toolbox*, as well as the *CATMoS* train and test data.[15-19] All datasets were filtered for LD_{50} values measured on rats as test animals and applied orally via gavage. As pseudo duplicates arised, i.e. one molecule with different LD_{50} values, from the different sources, values were averaged. All SMILES strings were canonicalized before duplicate removal. In a next step, values were transformed to mmol/kg and logarithmized, if necessary. Finally, SMILES strings in the external test dataset where canonicalized and removed from the training dataset to exclude data leakage. Due to non-consistent labeling in the original datasets, stereo information was removed from every structure. The final dataset consists of 17 580 datapoints. The dataset is supplied as *Tox_Dataset.xlsx* and can be used for non-commercial research purposes.

SMILES of drug molecules and their LSS-analogues were collected in a single *Excel* sheet and two columns were added. One column is used to mark if the corresponding molecule is an original drug or one of the LSS-analogues while a second, auxiliary column of 1 values was added named *Tox_Fake*. This column is a required input in the *EasyChemML* software but has no influence on the final predictions or model.

Table S10. Utilized data sources and number of (non-individual) datapoints.

Name	Reference	Number of datapoints
ChemIDplus	40	9210
ECHA/IUCLID	41	8907
QSAR Toolbox	42	13940
CATMoS train	43	8907
CATMoS test	43	2173
All datasets (non-individual)	-	46075
All datasets (individual)	-	17580

Splitting

To sufficiently evaluate the chosen model and parameters two different splits were applied and used for testing: a) 5-fold cross-validation, b) CATMoS validation set as external test set. In all cases data was split accordingly and a new model was trained with every training data while it was evaluated on the remaining test data afterwards. In case of the 5-fold cross validation the dataset was shuffled before splitting.

Table S11. Splits for model evaluation.

**Name**	**Test set size**	**Indices of the test set**	**Dataset**
5-fold - 1	3516	1 to 3516	shuffled
5-fold - 2	3516	3517 to 7032	shuffled
5-fold - 3	3516	7 033 to 10548	shuffled
5-fold - 4	3516	10548 to 14064	shuffled
5-fold - 5	3516	14065 to 17580	shuffled
CATMoS test	2173	15290 to 17580	ordered

Encoding

To obtain molecular features suitable for the following *CatBoost* model, SMILES strings from all datasets were first converted to *RDKit* mol objects from which *Multiple Feature Fingerprints* (*MFFs*)[20] have been constructed. Thereby, 1024 was chosen as length for all individual fingerprints

within the concatenated representation to keep training time reasonable. The *MFF* was chosen as molecular representation as it robustly encodes the molecular structure and demonstrated excellent performance on various tasks.

Training

Overall, six evaluation models were trained of the above mentioned training sets, followed by performance evaluation on the corresponding test sets. Final a seventh model was trained on the full dataset of 17 580 datapoints which was then deployed on the dataset of drugs and their LSS-analogues. Due to its robustness and typically high performance the *CatBoost* Model was used. Due to the complexity of structure toxicology relationships a comparably large Model was trained wherefor the maximum number of trees (*iterations*) was set on 50 000, while the depth of trees (*depth*) was set to 12.

Evaluation Results

All models were evaluated on their individual test sets and regarding the following metrics: R^2, mean average error (MAE), mean squared error (MSE), explained variance score (EVS), max error (MaxE). For all trained models good performance ($R^2 > 0.65$) on the external and diverse test sets was found. Therefore, chosen setup i.e. combination of molecular representation (*MFF*) and model (*CatBoost*) was considered as suited for predicting the toxicity of given drugs and synthesizes analogues.

Table S12. Evaluation metrics of the models with different train/test splits.

Test Set	R^2	MAE	MSE	EVS	MaxE
5-fold - 1	0.672	0.333	0.234	0.673	4.00
5-fold - 2	0.659	0.340	0.255	0.659	4.67
5-fold - 3	0.692	0.310	0.199	0.693	2.87
5-fold - 4	0.681	0.332	0.225	0.681	3.45
5-fold - 5	0.675	0.321	0.217	0.675	4.61
CATMoS test	0.686	0.315	0.207	0.686	3.80

Final Results

To predict the Median Lethal Concentration (LD_{50}) for given drugs and synthesizes analogues a model was trained again, given the above-mentioned settings. Thereby, the full dataset was utilized to even improve prediction accuracy even further. As all tested splits showed stable performance, a decline in performance is not expected. The final model was then applied on the given drug and analogue structures as test set. To make this deployment possible, structural data was supplied as *.xlsx*-file with three columns. One auxiliary column (*Tox_Fake*) having all values set two 1, as well as a column for the structures (*SMILES Drug*) and a column to differentiate between drug and reduced compounds. The auxiliary column is added as *EasyChemML* needs data to compare its predictions to.

Table S13. Predicted Median Lethal Concentration (LD_{50}) for given drugs.

**Molecule**	**SMILES String**	**LD_{50}/log$_{m}$ mol/kg**
**Aminacrine**	NC1=C2C(C=CC=C2)=NC3=CC=CC=C31	0.520844143
**Adiphenine**	O=C(OCCN(CC)CC)C(C1=CC=CC=C1)C2=CC=CC=C2	0.58572717
**Pridinol**	OC(C1=CC=CC=C1)(C2=CC=CC=C2)CCN3CCCCC3	0.31967018
**Primidone**	O=C(NCN1)C(CC)(C2=CC=CC=C2)C1=O	0.49153826
**Atenolol**	O=C(CC1=CC=C(OCC(CNC(C)C)O)C=C1)N	0.87669413
**Diethyltoluamide**	O=C(N(CC)CC)C1=CC=CC(C)=C1	0.9772883
**Gemfibrozil**	O=C(C(CCCOC1=CC(C)=CC=C1C)(C)C)O	0.82323612
**Praziquantel**	O=C(C1CCCCC1)N(CC2N3CCC4=C2C=CC=C4)CC3=O	0.80018653
**Prilocaine**	O=C(C(NCCC)C)NC1=CC=CC=C1C	0.8301483

		8
**Imipramine**	CN(C)CCCN1C2=CC=CC=C2CCC3=C1C=CC=C3	0.24309814
**Boc-Maprotiline**	CN(C(OC(C)(C)C)=O)CCCC12C3=CC=CC=C3C(CC2)C4=C1C=CC=C4	0.25743817
**Indoprofen**	O=C(C(C=CC=C1)=C1C2)N2C3=CC=C(C(C)C(O)=O)C=C3	-0.14722484
**Flurbiprofen**	FC1=CC(C(C)C(O)=O)=CC=C1C2=CC=CC=C2	-0.24306405
**Nabumetone**	O=C(C)CCC1=CC2=CC=C(OC)C=C2C=C1	1.12350666
**(S)-Naproxen-OMe**	O=C([C@H](C1=CC2=CC=C(OC)C=C2C=C1)C)OC	0.82854141
**Naftopidil**	OC(COC1=CC=CC2=C1C=CC=C2)CN3CCN(C4=C(OC)C=CC=C4)CC3	0.80381125
**Sacubitril**	O=C(CCC(O)=O)N[C@@H](C[C@H](C)C(OC)=O)CC(C=C1)=CC=C1C2=CC=CC=C2	0.44875077
**Benfluorex**	O=C(OCCNC(CC1=CC(C(F)(F)F)=CC=C1)C)C2=CC=CC=C2	0.35882042
**(R)-Tolterodine**	OC(C=CC(C)=C1)=C1[C@H](CCN(C(C)C)C(C)C)C2=CC=CC=C2	0.26820731
**Phenylethyl resorcinol**	OC(C=C(O)C=C1)=C1C(C)C2=CC=CC=C2	0.640716
**Boc-Atomoxetine**	CC(C=CC=C1)=C1O[C@@H](CCN(C)C(OC(C)(C)C)=O)C2=CC=CC=C2	0.28491894
**Butenafine**	CN(CC1=CC=C(C(C)(C)C)C=C1)CC2=CC=CC3=C2C=CC=C3	0.5800926

		7
**Cinacalcet**	CC(C1=CC=CC2=C1C=CC=C2)NCCCC3=CC=CC(C(F)(F)F)=C3	0.2601973 2
**Propranolol**	OC(CNC(C)C)COC1=CC=CC2=CC=CC=C12	0.5094332 1
**Midostauri n**	[H][C@@]1(C[C@@H](N(C)C(C2=CC=CC=C2)=O)[C@H]3OC)N(C4=C5C=CC=C4)C6=C5C(CNC7=O)=C7C8=C6N([C@@]3(C)O1)C9=C8C=CC=C9	0.3648716 9
**Pheniramin e**	CN(CCC(C1=CC=CC=C1)C2=CC=CC=N2)C	0.1887735 1
**Doxylamine**	CC(C1=CC=CC=N1)(C2=CC=CC=C2)OCCN(C)C	0.4246489 2
**Atazanavir**	COC(N[C@H](C(N[C@@H](CC1=CC=CC=C1)C(O)CN(CC2=CC=C(C3=NC=CC=C3)C=C2)NC([C@H](C(C)(C)C)NC(OC)=O)=O)=O)C(C)(C)C)=O	0.2312445
**Tropicamid e**	O=C(N(CC)CC1=CC=NC=C1)C(CO)C2=CC=CC=C2	0.4941051
**Melatonin**	O=C(C)NCCC1=CNC2=CC=C(OC)C=C21	0.9499573 8
**Zolmitripta n**	O=C1OC[C@H](CC2=CC=C(NC=C3CCN(C)C)C3=C2)N1	0.4965915 4
**Ketoprofen**	O=C(C1=CC=CC(C(C)C(O)=O)=C1)C2=CC=CC=C2	- 0.0659342 5
**Agomelatin e**	COC1=CC=C2C(C(CCNC(C)=O)=CC=C2)=C1	0.8530565 5
**Cicloprofen**	CC(C(O)=O)C1=CC(C2)=C(C=C1)C3=C2C=CC=C3	0.3290427 7
**58**	COC1=CC(C2=CC=CC=C2)=CC(CC(O)=O)=C1	0.6002229
**Biprofen**	CC(C(O)=O)C(C=C1)=CC=C1C2=CC=CC=C2	0.4443986

		5
**68**	C12=CC=CC=C1CCNC2C3=CC=CC=C3	0.2911977<br>6

Table S14. Predicted Median Lethal Concentration (LD_{50}) for synthesized LSS-analogues.

**Molecule**	**SMILES String**	**LD_{50}/log$_{mmol/kg}$**
**9**	NC1=C2C(CCCC2)=NC3=C1CCCC3	0.47029352
**Drofenine**	O=C(OCCN(CC)CC)C(C1=CC=CC=C1)C2CCCCC2	0.54226964
**Trihexylphenidyl**	OC(C1=CC=CC=C1)(CCN2CCCCC2)C3CCCCC3	0.74317100
**32**	O=C(NCN1)C(CC)(C2CCCCC2)C1=O	0.43410106
**21**	O=C(CC1CCC(CC1)OCC(CNC(C)C)O)N	0.89157622
**19**	O=C(N(CC)CC)C1CC(C)CCC1	0.92986816
**17**	O=C(C(CCCOC1C(C)CCC(C)C1)(C)C)O	1.11121506
**25**	O=C(C1CCCCC1)N(CC2N3CCC4C2CCCC4)CC3=O	0.73019877
**15**	O=C(C(NCCC)C)NC1CCCCC1C	0.84571058
**35**	CN(C)CCCN1C2CCCCC2CCC3=C1C=CC=C3	0.22800230
**14**	CN(C(OC(C)(C)C)=O)CCCC12C3CCCCC3C(CC2)C4=C1C=CC=C4	0.41213888
**37**	O=C(C(CCCC1)C1C2)N2C3=CC=C(C(C)C(O)=O)C=C3	0.25262706
**27**	FC1=CC(C(C)C(O)=O)=CC=C1C2CCCCC2	-

		0.2883 1298
**41**	O=C(C)CCC1CCC2=CC(OC)=CC=C2C1	1.0741 7507
**42**	O=C(C)CCC1=CC2=C(CC(CC2)OC)C=C1	0.9277 1224
**44**	O=C(C(C1CCC2=CC(OC)=CC=C2C1)C)OC	0.8703 6302
**45**	O=C(C(C1=CC2=C(CC(CC2)OC)C=C1)C)OC	0.6628 8439
**28**	OC(COC1=CC=CC2=C1CCCC2)CN3CCN(C4=C(OC)C=CC=C4)CC3	0.3996 4919
**70**	O=C(CCC(O)=O)N[C@@H](C[C@H](C)C(OC)=O)CC(C=C1)=CC=C1C2CCCCC2	0.3928 6209
**30**	O=C(C1CCCCC1)OCCNC(C)CC2=CC(C(F)(F)F)=CC=C2	0.5080 4716
**29**	OC(C=CC(C)=C1)=C1[C@H](CCN(C(C)C)C(C)C)C2CCCCC2	0.2314 5710
**34**	OC(C=C(O)C=C1)=C1C(C)C2CCCCC2	0.5847 9844
**31**	CC(C=CC=C1)=C1O[C@@H](CCN(C)C(OC(C)(C)C)=O)C2CCCCC2	0.3522 3719
**33**	CN(CC1=CC=C(C(C)(C)C)C=C1)CC2=CC=CC3=C2CCCC3	0.4657 1824
**39**	CC(C1=CC=CC2=C1CCCC2)NCCCC3=CC=CC(C(F)(F)F)=C3	0.3490 0424
**23’**	OC(CNC(C)C)COC1=CC=CC2=C1CCCC2	0.6961 5417
**23**	OC(CNC(C)C)COC1CCCC2CCCCC12	0.7388 0136

**38**	[H][C@@]1(C[C@@H](N(C)C(C2CCCCC2)=O)[C@H]3OC)N(C4=C5C=CC=C4)C6=C5C(CNC7=O)=C7C8=C6N([C@@]3(C)O1)C9=C8C=CC=C9	0.26783157
**72**	CN(CCC(C1=CC=CC=C1)C2CCCCN2)C	-0.02016025
**71**	CC(C1CCCCN1)(C2=CC=CC=C2)OCCN(C)C	0.06828648
**74**	COC(N[C@H](C(N[C@@H](CC1=CC=CC=C1)C(O)CN(CC2=CC=C(C3NCCCC3)C=C2)NC([C@H](C(C)(C)C)NC(OC)=O)=O)=O)C(C)(C)C)=O	0.13279681
**73**	O=C(N(CC)CC1CCNCC1)C(CO)C2=CC=CC=C2	0.46322828
**46**	O=C(C)NCCC1CNC2=C1C=C(C=C2)OC	0.56851524
**47**	O=C1OC[C@H](CC2=CC(C(CN3)CCN(C)C)=C3C=C2)N1	0.37083124
**51**	CC(C(O)=O)C1=CC(CC2CCCCC2)=CC=C1	0.47311040
**66**	COC1=CC2=C(C=C1)CCCC2CCNC(C)=O	0.50582167
**54**	CC(C(O)=O)C1=CC2=C(C=C1)C3CCCCC3C2	0.30273684
**59**	COC1=CC(C2CCCCC2)=CC(CC(O)=O)=C1	0.50376423
**61**	CC(C(O)=O)C(C=C1)=CC=C1C2CCCCC2	0.25409502
**63**	CC(C(O)=O)C(CC1)CCC1C2=CC=CC=C2	0.78131445
**69**	C12=CC=CC=C1CCNC2C3CCCCC3	0.35070602

5.5.2.4 Pk_a Value Prediction

For selected pyridines and pyridines, pk_a-values have been predicted using MolGpka.[23]

Table S16. Computational analysis of changes in pKa values**.**

Drugs-H^+	pKa	Saturated Drugs-H^+	pKa
Doxylamine	4.7	**71**	9.3
Pheniramine	5.6	**72**	10.3
Tropicamide	5.4	**73**	10.4
Atazanavir	4.7	**74**	9.6

5.5.3 Statistical Data Analysis (Program)

5.5.3.1 Program Structure

To standardize, analyze and plot given property data and a simple python (*Data_Analysis.py*) script was created and applied. This script is split into 5 main sections:

1. General functions
2. Loading
3. Statistical property analysis
4. Plotting
5. Export

Overall, the code ready data from its subfolders *SwissAMED* and *Tox/Final_predictions* and writes plots for different features as well as generated statistical data to a *Final_data* folder.

5.5.3.2 General Functions

General functions cover line 14 to 70 of the code.

count_categories_column

This function takes a *pandas Dataframe* as well as the string of any specific column in it and constructs a dictionary of all individual categories i.e. values and their occurrence in this column. It converts all types into strings before comparing them.

plt_histogram

This function takes a *pandas Series* of plottable values (integers or floats), a color as a hex RGB string (e.g. *#c90076*), the number of bins as well as the range of the plot as tuple of two integers or floats.

It constructs the bins and histogram values and constructs the plot.

make_weights_for_log_scale

This function takes a *pandas Series* of values and the edges of bins as *numpy array* and sorts the values into these bins. Thereby, it constructs a list of integers which can be used for histograms plotting. This is necessary to construct buckets of different absolute length e.g. in case of logarithmically scaled histograms.

plt_histogram_log

Similar to function *plt_histogram* but it but it logarithmizes the x-axis while also rescaling the buckets. Those will have equal sizes on the logarithmic scale.

plt_histogram_inverse

Similar to function *plt_histogram* but it multiplies all given values by -1 to turn them into their negatives. Thereby they can be plotted opposite to other values which simplifies comparison in the final plot.

plt_histogram_inverse_log

Similar to function *plt_histogram_log* but it multiplies all given values by -1 to turn them into their negatives. Thereby they can be plotted opposite to other values which simplifies comparison in the final plot.

5.5.3.3 Data Loading

Data loading covers line 73 to 88 of the code.

To analyze predicted properties the program loads data from two the *SwissAMED* and *Tox/Final_predictions* folder in the same directory. Thereby it loads the following files to *Dataframe* objects as from the *pandas* library:

SwissAMED/Drug_swiss_ADME.csv

SwissAMED/Hydro_swiss_ADME.csv

Tox/Final_predictions/Tox_Prediction.xlsx

From the later, it loads both sheets (*Drugs* & *Hydro*) into separate files.

5.5.3.4 Mean and Deviation Prediction

Mean and Deviation Prediction covers line 90 to 167 of the code.

To compare the predicted values for different properties of all given drugs and their LSS-analogues mean and standard deviation was calculated was calculated for numerical parameters. Therefore, the build-in functions of *pandas* were applied on the corresponding column in the related *Dataframe*. Mean and standard deviation was calculated for the following properties and columns:

- Fraction of C(sp^3)-Atoms (column: Fraction Csp3)
- Topological Polar Surface Area (TPSA/$Å^2$, column: TPSA")
- Octanol/Water Partition Coefficient (Log $P_{o/w}$, column: Consensus Log P")
- Solubility in Water (mg/ml, column: Solubility Consensus (mg/ml))
- Median Lethal Concentration (LD_{50}/$Log_{mmol/kg}$, column: logLD50)

As, in the *SwissADME* toolset, different models are used to predict the solubility in water predictions from these models were averaged and added to a new column of the corresponding *Dataframe* (*Solubility Consensus (mg/ml)*)

To count occurrence of specific classes for categorical properties the *count_categories_column* function was used. It was applied on the following properties and columns:

- Gastrointestinal Absorption Class (column: GI absorption")
- Blood Brain Barrier Permeability Class (BBB, column: BBB permeant)
- Pan Assay Interference Structures (PAINS, column: PAINS #alerts")
- Brenk Structural Alerts (column: Brenk #alerts")

- Inhibition of Cytochrome P450 Enzymes
    - CYP1A2 Inhibition (column: CYP1A2 inhibitor”)
    - CYP 3A4 Inhibition (column: CYP3A4 inhibitor”)
    - CYP 2C9 Inhibition (column: CYP2C9 inhibitor”)
    - CYP 2C19 Inhibition (column: CYP2C19 inhibitor”)
    - CYP 2D6 Inhibition (column: CY2D6 inhibitor”)

5.5.3.5 Data Visualization

Data visualization covers line 169 to 565 of the code.

To visualize the distribution and mean of the different, above-mentioned properties, histograms (for numerical properties) and bar charts (for classifying properties) were generated. Therefore, the *matplotlib python* library was utilized.[20] General properties where defined using the *pyplot.rcParams* parameters (line 174 to 183). Herein, the font, font size, plotsize and colors are defined. To plot the numerical data, typically the same code structure was utilized:

1. Initializing and generally defining the plot
2. Setting grid, axis limits and axis labels
3. Selecting the data and plotting the histogram or bar chart
4. Defining and adding the legend
5. Adding and annotating a line for the mean
6. Setting layout, path and Figure parameters and saving the plot as *.png*

Plot are saves as *.png* file in the *Final_data* subfolder.

5.5.3.6 Data Export

Exporting data covers line 567 to 646 of the code.

While plots are saved immediately after generation, statistical data (mean, classification counts, standard derivation) is exported and saved in the final section of the program. Therefore, a hierarchical dictionary is constructed, holding statistical values of different parameter (TPSA, LogP, …) grouped by their ADMET class (Absorption, Distribution, …). This dictionary is saved in the *Final_data* subfolder as *.json* file (*Statistical_results.json*).

5.5.4 Statistical Data Analysis (Results)

5.5.4.1 Mean & Standard Deviation

Table S15. Mean and standard deviation of properties with numerical values for drugs and synthesized LSS-analogues.

**Property**	**Drugs**		**LSS-Analogues**	
	mean	*standard deviation*	*mean*	*standard deviation*
Fraction of C(sp^3)-atoms (%)	0.326	0.138	0.663	0.185
TPSA ($Å^2$)	43.1	29.3	40.7	27.6
$LogP_{o/w}$	3.23	1.10	3.36	1.16
Solubility in Water (mg/ml)	0.380	1.25	0.638	2.05
$LogLD_{50}$ ($log_{mmol/kg}$)	0.488	0.313	0.497	0.290

5.5.4.2 Classification counts

Gastrointestinal Absorption Class

	Low	*High*
**Drugs**	3 (8%)	34 (92%)
**LSS-Analogues**	3 (7%)	40 (93%)

Blood Brain Barrier Permeability Class

	No	*Yes*
**Drugs**	7 (19%)	30 (81%)
**LSS-Analogues**	10 (23%)	33 (77%)

Pan Assay Interference Structures

	Zero	*One*
**Drugs**	36 (97%)	1 (3%)
**LSS-Analogues**	43 (100%)	0 (0%)

Brenk Structural Alerts

	Zero	*One*
**Drugs**	34 (92%)	3 (8%)
**LSS-Analogues**	41 (95%)	2 (5%)

Inhibition of Cytochrome P450 Enzymes

CYP1A2 Inhibition

	No	*Yes*
**Drugs**	24 (65%)	13 (35%)
**LSS-Analogues**	35 (81%)	8 (19%)

CYP 3A4 Inhibition

	No	*Yes*

**Drugs**	27 (73%)	10 (27%)
**LSS-Analogues**	36 (84%)	7 (16%)

CYP 2C9 Inhibition

	No	*Yes*
**Drugs**	33 (89%)	4 (11%)
**LSS-Analogues**	34 (79%)	9 (21%)

CYP 2C19 Inhibition

	No	*Yes*
**Drugs**	28 (76%)	9 (24%)
**LSS-Analogues**	38 (88%)	5 (12%)

CYP 2D6 Inhibition

	No	*Yes*
**Drugs**	15 (41%)	22 (59%)
**LSS-Analogues**	16 (37%)	27 (63%)

5.5.4.3 Distributions

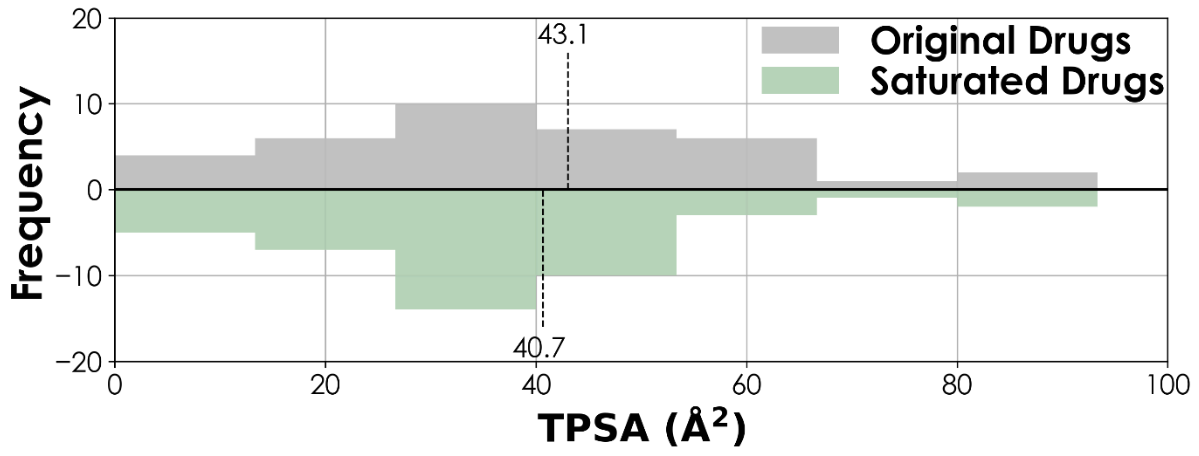

Figure S4. Distribution of the topological polar surface area of analyzed drugs and their saturated analogues.

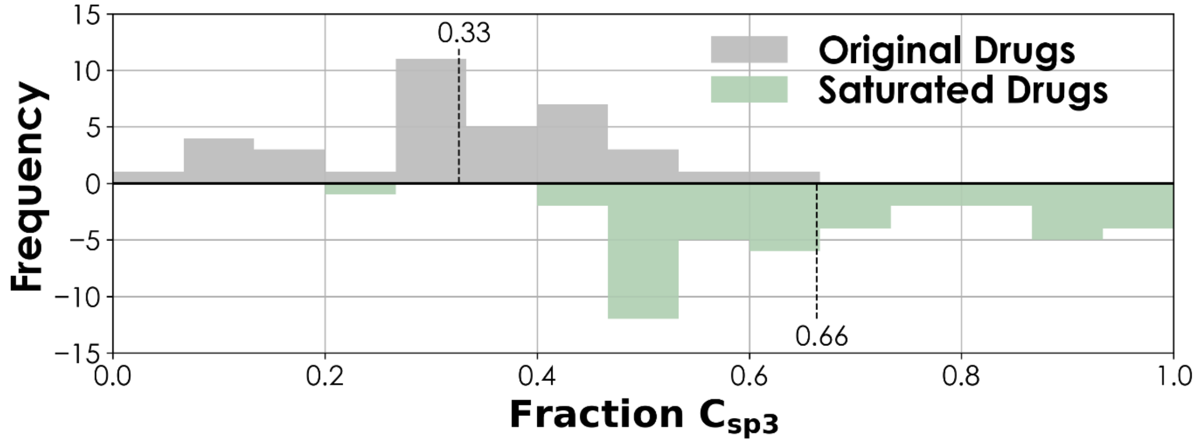

Figure S5. Distribution of the fraction of C(sp^3)-atoms of analyzed drugs and their saturated analogues.

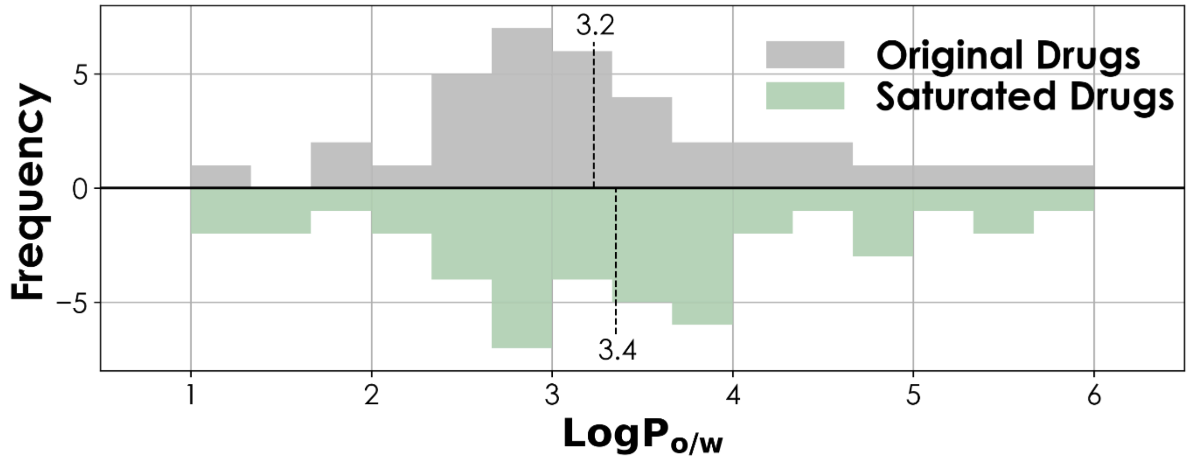

Figure S6. Distribution of the octanol/water partition coefficient of analyzed drugs and their saturated analogues.

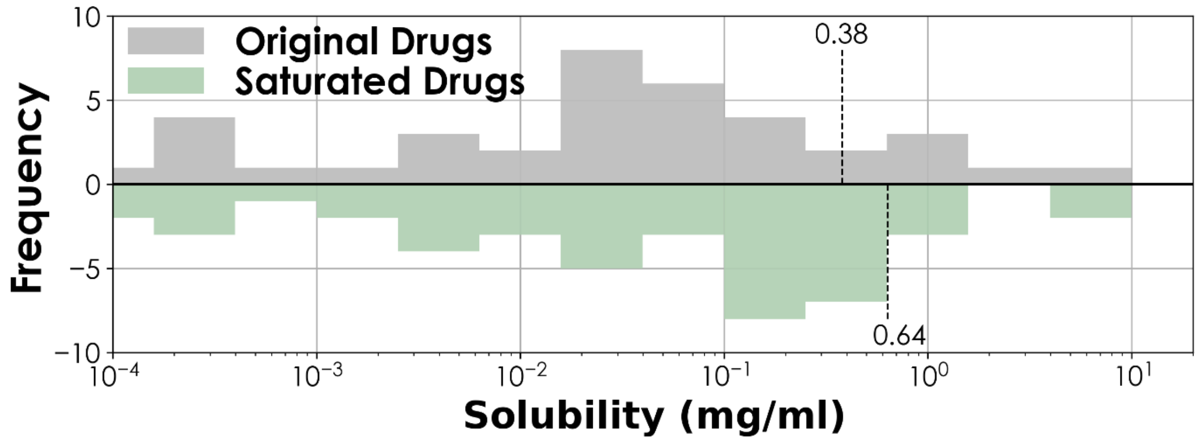

Figure S7. Distribution of the water solubility of analyzed drugs and their saturated analogues.

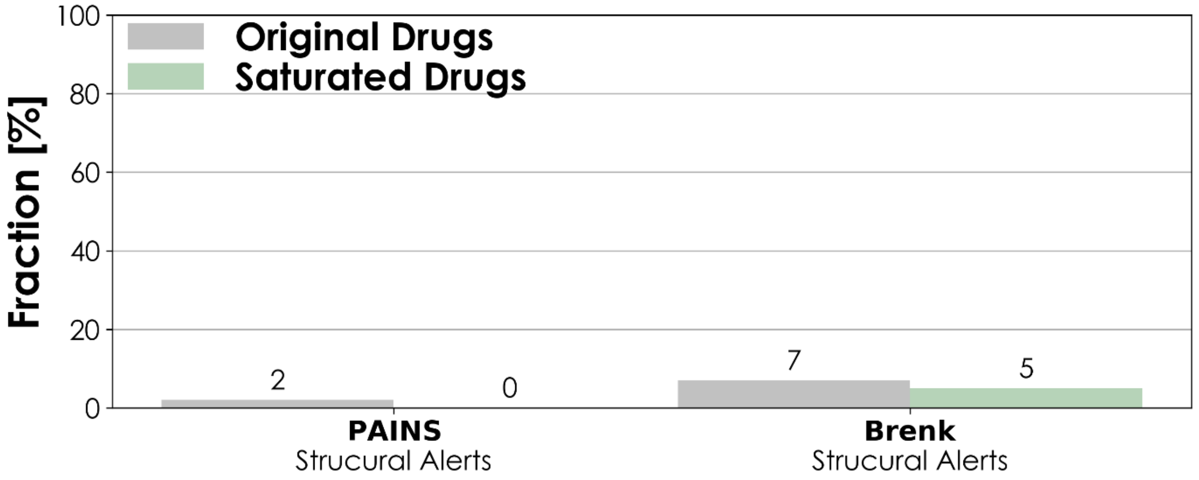

Figure S8. Fraction of analyzed drugs and their saturated analogues with PAINS and Brenk structural alerts.

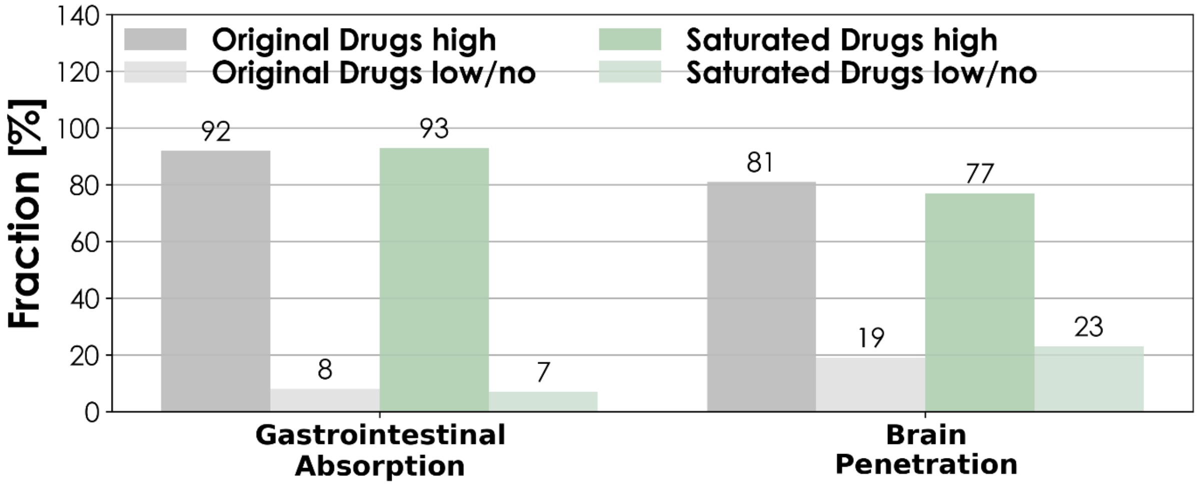

Figure S9. Fraction of analyzed drugs and their saturated analogues with high and low gastrointestinal absorption and brain penetration ability.

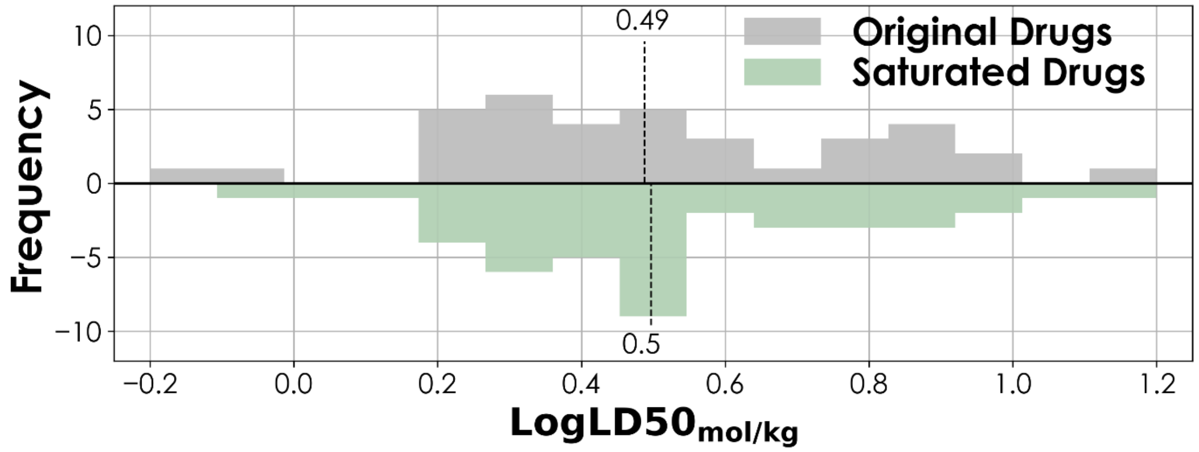

Figure S10. Distribution of the median lethal concentration of analyzed drugs and their saturated analogues.

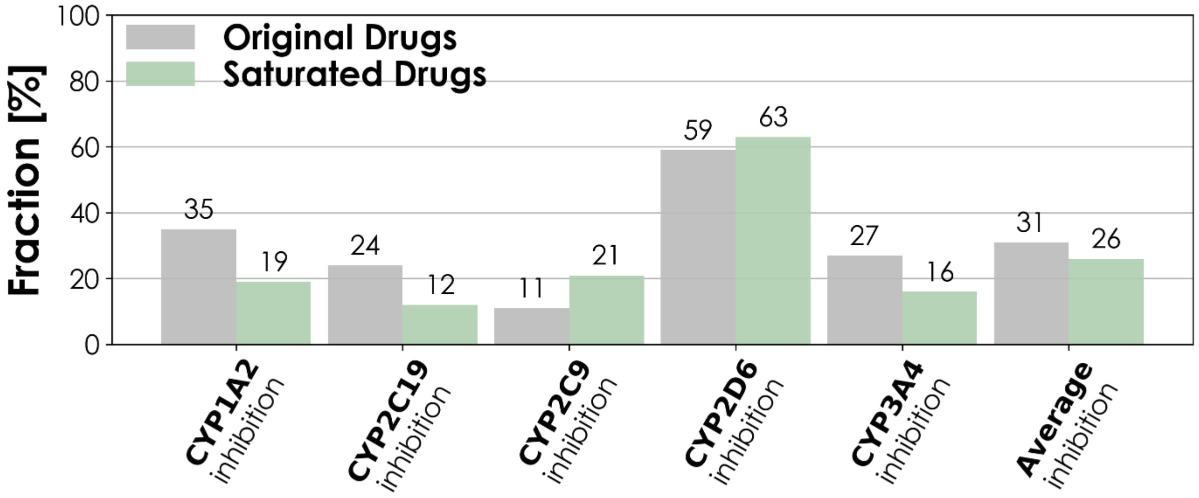

Figure S11. Fraction of analyzed drugs and their saturated analogues inhibiting different cytochrome P450 enzymes.

Table S16. Computational analysis of changes in pKa values.

Drugs-H$^+$	pKa	Saturated Drugs-H$^+$	pKa
Doxylamine	4.7	71	9.3
Pheniramine	5.6	72	10.3
Tropicamide	5.4	73	10.4
Atazanavir	4.7	74	9.6

6. Measurement of Metabolic stability in Human Liver Microsomes

Metabolic stability of cinacalcet and **39** in human liver microsomes was independently performed by Pharmaron (Ningbo, China).

Materials:

The microsome was stored at -80°C prior to use, and the liver microsomes information is listed in **Table** .

**Table S17.** Liver microsomes information				
Species	Vendor	Cat. No.	Lot. No.	Strain & Gender
Human	BD Gentest	452117	38297	Pooled, Mixed Gender

Study Design:

1) The master solution was prepared according to **Table S18**.

**Table S18**. Preparation of master solution			
Reagent	Stock Concentration	Volume	Final Concentration
Phosphate buffer	100 mM	216.25 µL	100 mM
Microsomes	20 mg/mL	6.25 µL	0.5 mg/mL

2) Two separated experiments were performed as follows.

a) With Cofactors (NADPH): 25 µL of 10 mM NADPH was added to the incubations. The final concentrations of microsomes and NADPH were 0.5 mg/mL and 1 mM, respectively.

b) Without Cofactors (NADPH): 25 µL of 100 mM Phosphate buffer was added to the incubations. The final concentration of microsomes was 0.5 mg/mL. The mixture was pre-warmed at 37°C for 10 minutes.

3) The reaction was started with the addition of 2.5 µL of 100 µM control compound or test compound solutions. Verapamil was used as positive control in this study. The final concentration of test compound or control compound was 1 µM. The incubation solution was incubated in water batch at 37°C.

4) Aliquots of 30 µL were taken from the reaction solution at 0.5, 5, 15, 30 and 60 minutes. The

reaction was stopped by the addition of 5 volumes of cold acetonitrile with IS (100 nM alprazolam, 200 nM caffeine and 100 nM tolbutamide). Samples were centrifuged at 3, 220 g for 40 minutes. Aliquot of 100 µL of the supernatant was mixed with 100 µL of ultra-pure H_2O and then used for LC-MS/MS analysis.

5) Data analysis

All calculations were carried out using Microsoft Excel.

Peak areas were determined from extracted ion chromatograms. The slope value, k, was determined by linear regression of the natural logarithm of the remaining percentage of the parent drug vs. incubation time curve.

The in vitro half-life (in vitro $t_{1/2}$) was determined from the slope value:

$$\textit{in vitro}\ t_{1/2} = -(0.693 / k)$$

Conversion of the in vitro $t_{1/2}$ (min) into the in vitro intrinsic clearance (in vitro CL_{int}, in µL/min/mg protein) was done using the following equation (mean of duplicate determinations):

$$\textit{in vitro}\ CL_{int} = \left(\frac{0.693}{(t_{1/2})}\right) * \left(\frac{\text{volume of incubation } (\mu L)}{\text{amount of proteins (mg)}}\right)$$

The calculations of Scale-up CL_{int} (mL/min/kg), Predicted Hepatic CLH (mL/min/kg) and Hepatic Extraction Ratio (ER) were done using the following equation:

Scale-up CL_{int} = (0.693/$T_{1/2}$) × (1/(microsomal protein concentration (0.5 mg/mL))) × Scaling Factors (**Table S19**)

Predicted Hepatic CL_H = (QH × Scale-up CL_{int} × fub) / (QH + Scale-up CL_{int} × fub),

ER = Predicted Hepatic CL_H/QH

where QH is the hepatic blood flow (mL/min/kg) (Table S19), fub is the fraction of unbound drug in plasma which is assumed to be 1.

Table S19. Scaling factors for intrinsic clearance prediction in human microsomes

Species	Microsomal Protein per Gram of Liver	Liver Weight per Kilogram of Body Weight	Scaling Factor[a]	Hepatic Blood Flow (mL/min/kg)
Human	40	25.7	1028	21

[a]Scaling Factor = (microsomal protein per gram of liver) × (liver weight per kilogram of body weight)

Bioanalytical Method

1) Chromatographic conditions

LC system: Shimadzu

MS analysis: Triple Quad™ 4500 instrument from AB Inc with an ESI interface

Column temperature: 40°C

Column :Waters XSelect® HSS T3 2.5μm 2.1×50mm Column XP

Mobile phase: 0.1% Formic Acid in Water (A) and 0.1% Formic Acid in Acetonitrile (B)

Injection volume: 8 μL

Elution rate: 0.8 mL/min

Time (min)	0	0.2	0.5	0.9	0.95	1.2
% B	5	5	98	98	5	5

2) MS parameters

Ion source: Turbo spray

Ionization model: ESI

Scan type: MRM

Curtain gas: 40 L/min

Collision gas: 9 L/min

Nebulize gas: 55 L/min

Auxiliary gas: 55 L/min

Temperature: 500 °C

Ionspray voltage: +5500 V (positive)

Results

1) The experimental data are summarized in **Table S20** and **Table S21**.

Table S20. Remaining percentage of test compounds and control compound in human liver microsome

**Compound ID**	**Species**	**Assay Format**	**Remaining Percentage (%)**			
			**0.5 min**	**5 min**	**15 min**	**30 min**
Verapamil	Human	+Cofactors	100.00	42.13	9.54	4.04
Compound **39**	Human	+Cofactors	100.00	85.98	57.81	38.01
Cinacalcet	Human	+Cofactors	100.00	80.88	55.07	37.55

2) The data processing is summarized in **Figure S12**, **Figure S13**, **Figure S14**.

Mean of Clint (µL/min/mg protein)	384.30
Clint-1	394.98
Clint-2	373.61
Mean of $t_{1/2}$ (min)	**3.61**
$t_{1/2}$-1	3.51
$t_{1/2}$-2	3.71
05 (Mean of %Remair	**Yes**
P<0.05-1	Yes
P<0.05-2	Yes
(Mean of %Remainir	**1.0000**
R^2-1	1.0000
R^2-2	1.0000
IS CV%	**2.87**
Slope	-0.1921

Time (min)	%Remaining-1	%Remaining-2	%Remaining-N	LN of %Remaining-1	LN of %Remaining-2	Mean of %Remaining	LN (Mean of %Remaining)	LN (Mean of %Remaining)
0.5	100.0	100.0	100.0	4.61	4.61	100.0	4.61	4.61
5	41.12	43.14		3.72	3.76	42.13	3.74	3.74
15	9.21	9.87				9.54		#N/A
30	4.31	3.78				4.04		#N/A

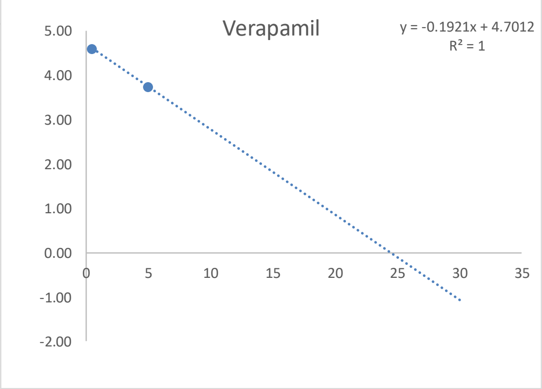

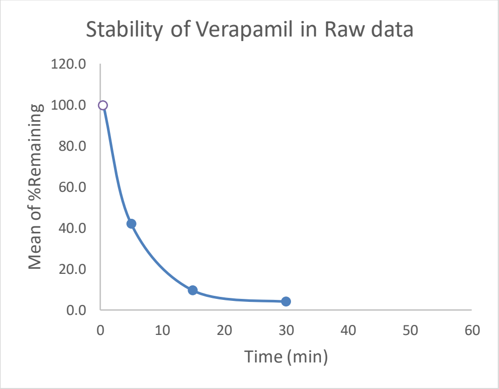

Figure S12. Data processing of Verapamil.

Mean of Clint (µL/min/mg protein)	66.16
Clint-1	64.02
Clint-2	68.31
Mean of $t_{1/2}$ (min)	**20.97**
$t_{1/2}$-1	21.65
$t_{1/2}$-2	20.29
05 (Mean of %Remair	**Yes**
P<0.05-1	Yes
P<0.05-2	Yes
(Mean of %Remainir	**0.9933**
R^2-1	0.9860
R^2-2	0.9967
IS CV%	**2.87**
Slope	-0.0331

Time (min)	%Remaining-1	%Remaining-2	%Remaining-N	LN of %Remaining-1	LN of %Remaining-2	Mean of %Remaining	LN (Mean of %Remaining)	LN (Mean of %Remaining)
0.5	100.0	100.0	100.0	4.61	4.61	100.0	4.61	4.61
5	83.73	88.22		4.43	4.48	85.98	4.45	4.45
15	56.55	59.08		4.04	4.08	57.81	4.06	4.06
30	38.89	37.13		3.66	3.61	38.01	3.64	3.64

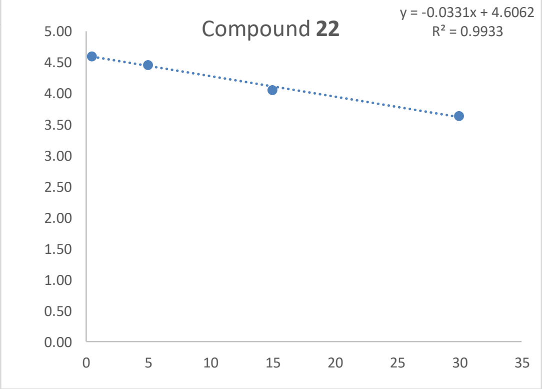

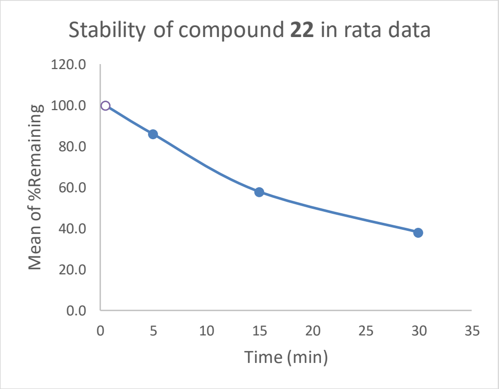

Figure S13. Data processing of compound **39**.

Mean of Clint (µL/min/mg protein)	65.56	Time (min)	%Remaining-1	%Remaining-2	%Remaining-N	LN of %Remaining-1	LN of %Remaining-2	Mean of %Remaining	LN (Mean of %Remaining)	LN (Mean of %Remaining)
Clint-1	66.09	0.5	100.0	100.0	100.0	4.61	4.61	100.0	4.61	4.61
Clint-2	65.03	5	73.48	88.28		4.30	4.48	80.88	4.39	4.39
**Mean of $t_{1/2}$ (min)**	**21.14**	15	54.63	55.52		4.00	4.02	55.07	4.01	4.01
$t_{1/2}$-1	20.97	30	35.78	39.31		3.58	3.67	37.55	3.63	3.63
$t_{1/2}$-2	21.31									

05 (Mean of %Remaii	**Yes**
P<0.05-1	Yes
P<0.05-2	Yes
(Mean of %Remainir	**0.9836**
R^2-1	0.9732
R^2-2	0.9755
IS CV%	**3.85**
Slope	-0.0328

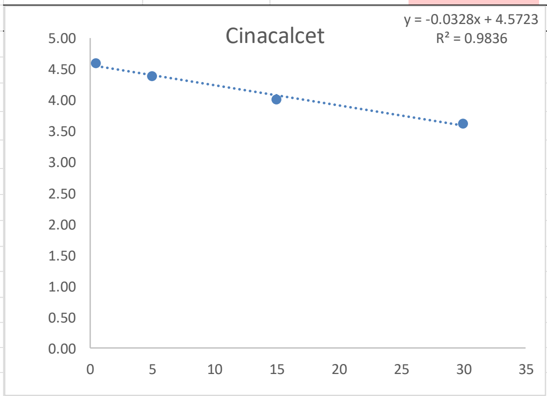

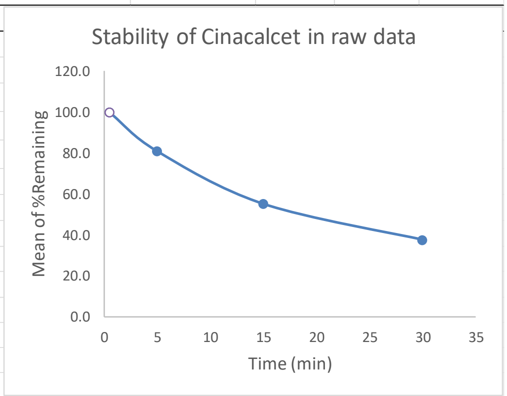

Figure S14. Data processing of Cinacalcet.

**Table S21.** Metabolic stability of test compounds and control compound in human liver microsome						
**Compound ID**	**Species**	***in vitro* $t_{1/2}$ (min)**	***in vitro* CL_{int} (µL/min/mg)**	**Scale-up CL_{int} (mL/min/Kg)**	**Predicted Hepatic CL_H (mL/min/kg)**	**Hepatic Extraction Ratio (ER)**
Verapamil	Human	3.61	384.30	395.06	19.94	0.95
Compound **39**	Human	20.97	66.16	68.01	16.05	0.76
Cinacalcet	Human	21.14	65.56	67.40	16.01	0.76

7. Reaction-Condition-Based Sensitivity Assessment

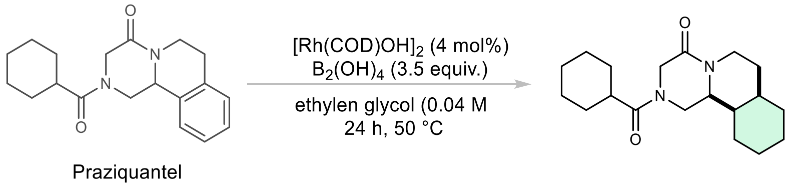

Preparation of stock solution: Praziquantel (375 mg, 1.20 mmol, 1.0 equiv.) was dissolved in dry ethylene glycol (15 mL).

Standard reaction conditions: n = 0.10 mmol, c = 0.04 M, V = 2.5 mL, T = 50 °C, Vial Size = 10 mL.

Standard reaction procedure: A 10 mL pressure tube was charged with $B_2(OH)_4$ (31.4 mg, 0.350 mmol, 3.5 equiv.) and $[Rh(COD)OH]_2$ (1.8 mg, 0.0040 mmol, 4 mol%) under Argon. Stock solution (1.25 mL) and dry ethylene glycol (1.25 mL) were added and the mixture was stirred at 50 °C for 24 h. After cooling to r.t., mesitylene was added as internal standard and the yield was determined *via* GC-FID.

Big Scale Conditions: n = 2.0 mmol, c = 0.04 mmol, V = 50 mL, T = 50 °C, Vial Size = 150 mL.

Entry	Experiment	Deviation	Yield	Yield dev.
1	standard	-	74%	-
2	high c	no extra ethylen glycol (0.08M)	52%	-22%
3	low c	+ 2.5 mL ethylen glycol (0.02M)	72%	-2%
4	+ H_2O	+ 25 µL H_2O	66%	-8%
5	low O_2	degassed solvent	61%	-13%
6	high O_2	+ 25 mL air	70%	-4%
7	high V flask	reaction conducted in 50 mL pressure tube	55%	-19%
8	low V flask	reaction conducted in 5 mL pressure tube	66%	-8%
9	low T	T = 40 °C	74%	0%
10	high T	T = 60 °C	57%	-17%
11	big scale	n = 2 mmol	71%	-3%
12	Rh Catalyst	$[Rh(COD)Cl]_2$ instead of $[Rh(COD)OH]_2$	40%	-34%

13	Boron Reagent	NH_3BH_3 instead of $B_2(OH)_4$	34%	-40%

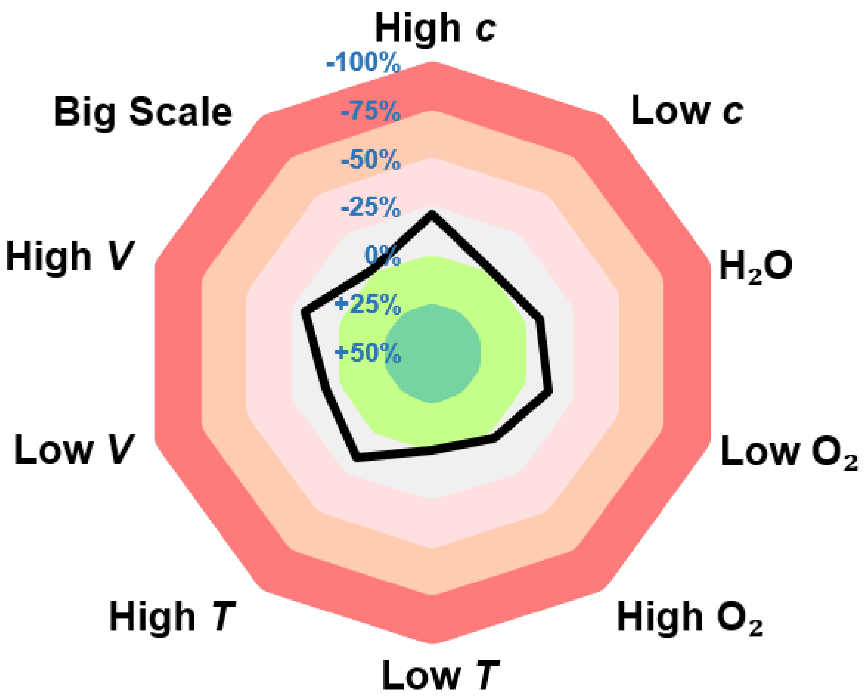

8. Ultra-High Throughput Synthesis of a Diversified Drug Library using LSS

8.1. Determination of DMSO Tolerance

To quantitate DMSO remaining after evaporation and reconstitution cycles of solutions of drug library samples, GC-MS analysis was used to detect residual DMSO in 384-well plate format that could inhibit catalytic activity in rhodium-catalyzed hydrogenation. Initially, a standard calibration curve was produced from GCMS response to DMSO (0.1 mM to 30 mM) in methanol. A standard solution of 100 mM DMSO in methanol was used to prepare solutions of 30 mM, 20 mM, 10 mM, 1 mM, and 0.1 mM DMSO via serial dilution. The standard samples ranging from 30 to 0.1 mM DMSO were run in triplicate to produce a calibration curve of mass spectrometer response to DMSO across relevant concentration ranges that would impede rhodium-catalyzed hydrogenation.

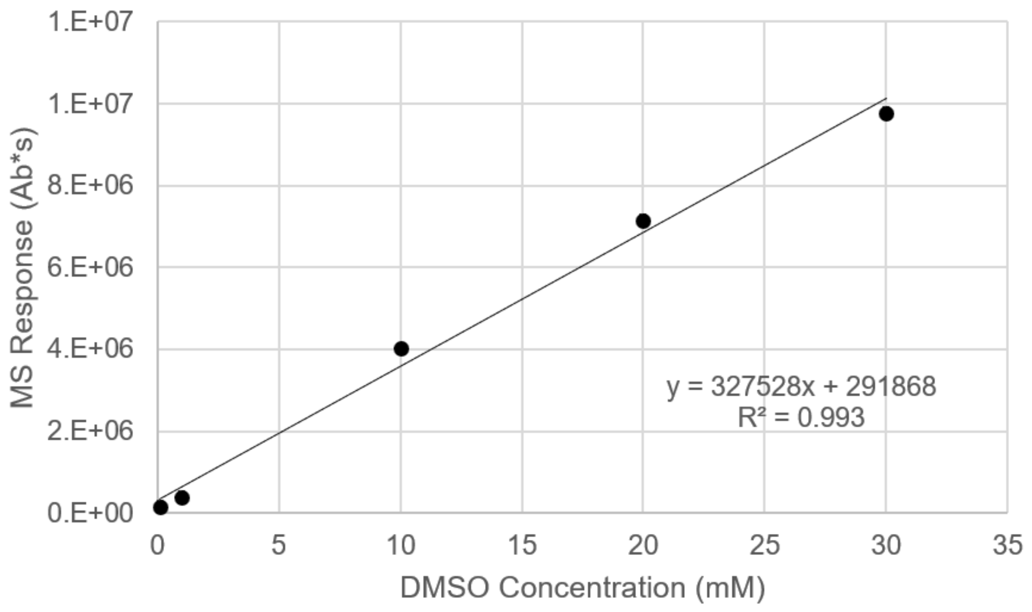

Figure S15. Calibration Curve for GC-MS Response to DMSO

To test efficiency of DMSO removal in well plate format, a single 384-well microtiter plate was filled with DMSO (30 µL per well) and the plate was subjected to a 2-hour evaporation cycle using a Genevac HT-4X. To three selected wells, 30 µL of methanol was added then transferred to the appropriate GC-MS analytical vials pre-filled with 1.0 mL of methanol. The first round of samples were analyzed in triplicate using a standard calibration curve to determine DMSO concentrations after one round of evaporation. The process was then repeated a second and third time, by portioning 30 µL of ethanol to all wells in the 384-well plate, subjecting the plate to a 2-hour evaporation cycle,

and selecting three new wells to aliquot into analytical vials for GCMS analysis. By the end of the third evaporation cycle no detectable DMSO concentrations were evident in the 384-well plate samples.

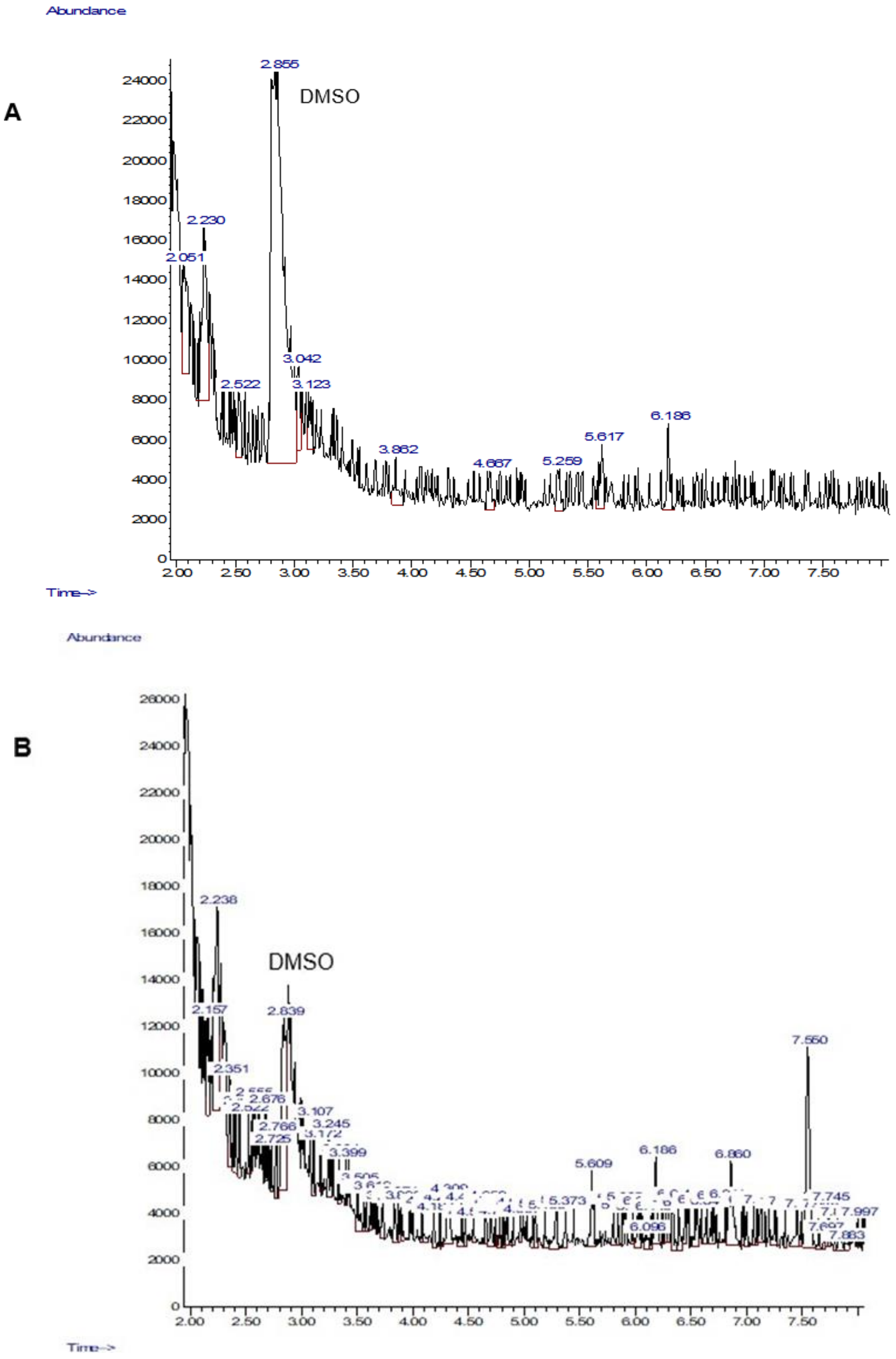

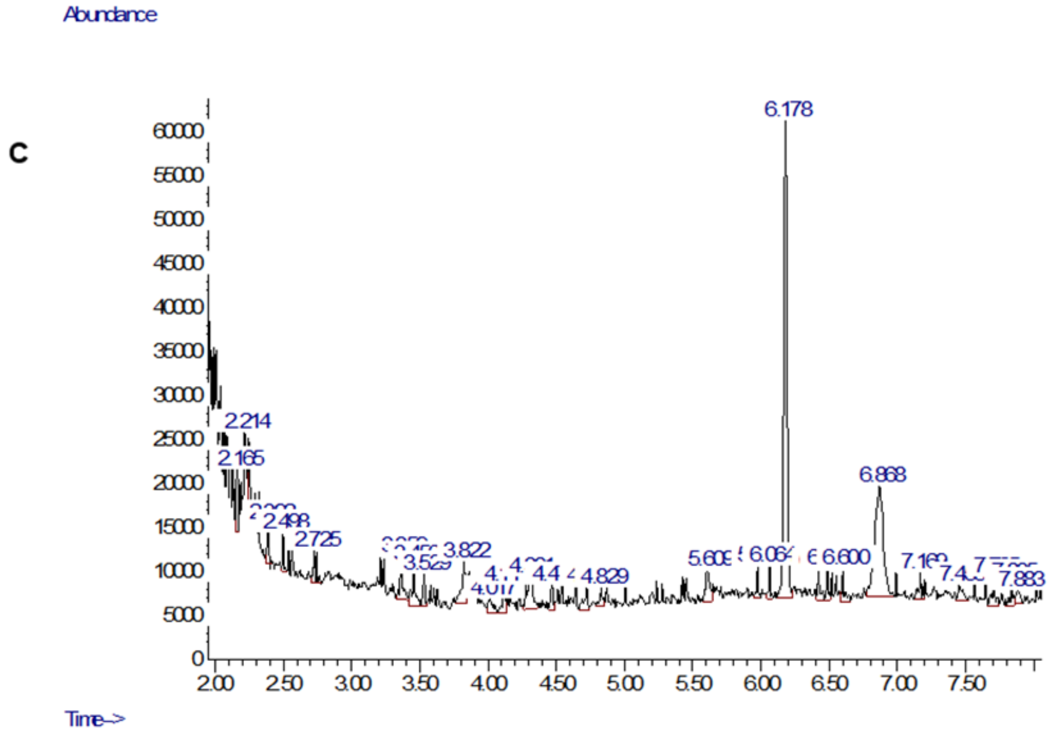

Figure S16. Residual DMSO in 384-Well Plates Following First (A), Second (B), and Third (C) Evaporation Cycles

8.2 Quantitation Method using UPLC-MS

To address the variability in UV detection for unsaturated drugs and their saturated counterparts, a mass spectrometry-based quantitation method was developed for analysis of high throughput experiments. Gemfibrozil, Propranolol, and Ketoprofen were used as model substrates to determine the correlation between substrate conversion determined by integration of UPLC-MS ionization traces and NMR yield. Each substrate was exposed to reducing rhodium conditions for 20 hours (4 mol% Rh, 3.5 equiv $B_2(OH)_4$, 0.2M EtOH). A 20 µL aliquot of each reaction was syringe filtered into acetonitrile (1.0 mL) along with addition of aqueous caffeine (100 µL, 0.1 M) as internal standard. Samples were then taken for UPLC-MS analysis. The MS ionization peak area for each product and starting material was calculated to give $\text{Area}_{\text{product}}$ and Area_{sm} for each substrate. The percent conversion to product was then calculated with the following equation:

$$\%Conversion_{Product}^{MS} = 100\ \times \left(\frac{\text{Area}_{\text{product}}}{Area_{sm} + Area_{product}}\right)$$

For NMR yields, reaction mixtures were quenched by addition of 10 mL saturated $NaHCO_3$, extracted in triplicate with DCM (3 x 10 mL) and solvent was removed *in vacuo*. To each of the crude residues, a 0.1 M stock solution of 1,3,5-trimethoxybenzene (1.68 mg, 100 µL, 0.1 equiv) in $CDCl_3$ was dosed for internal standard. Each sample was diluted up to 600 µL with $CDCl_3$, then ^{1}HNMR yields calculated for each substrate. Satisfactory correlation between traditional NMR yield and percent conversion from MS ionization peak integration was found. Comparison of %MS conversion and NMR yield methods is shown in the table below.

Table S22. Comparison of Conversion by Mass Spectrum Integration and by NMR Yield

Substrate	Conversion by MS (%)	NMR Yield (%)
Gemfibrozil	54	57
Propranolol	85	79
Ketoprofen	77	71

8.3 Microtiter Plate Sealing Studies

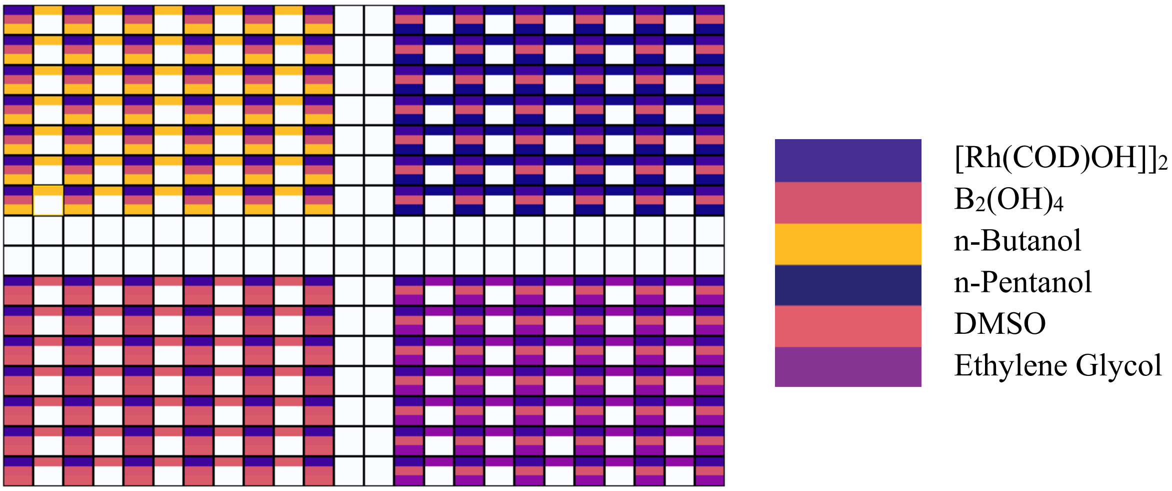

Figure S17. 384-well plate split into 4 quadrants according to solvent along with color mapping for each added reagent.

Solvent effects on well plate sealing integrity were determined from subjecting a series of alcohol-based solvents to model reaction conditions (50°C for 24 hours) in 384-well plate format. A single 384-well microtiter plate was portioned into quadrants. To each quarter a different HTE-viable solvent was allocated (n-Butanol, n-Pentanol, DMSO, or Ethylene Glycol). 76 wells were allocated per

solvent with 30 µL pure solvent added to each well. Two rows/columns were left empty between each quadrant. Four 0.1 M stock solutions of $[Rh(COD)OH]]_2$ and four 0.1 M stock solutions of $B_2(OH)_4$ were freshly prepared by dissolving the corresponding reagent (91 mg Rh or 18 mg $B_2(OH)_4$) in one of the four corresponding solvents (2.0 mL). From these eight total stock solutions, 10 µL Rh stock solution and 10 µL $B_2(OH)_4$ stock solution were added to each well previously dosed with solvent. Butanol-containing stock solutions were allocated to the top left of the plate, pentanol to the top right, DMSO to the bottom left, and ethylene glycol to the bottom right.

n-BuOH n-PeOH

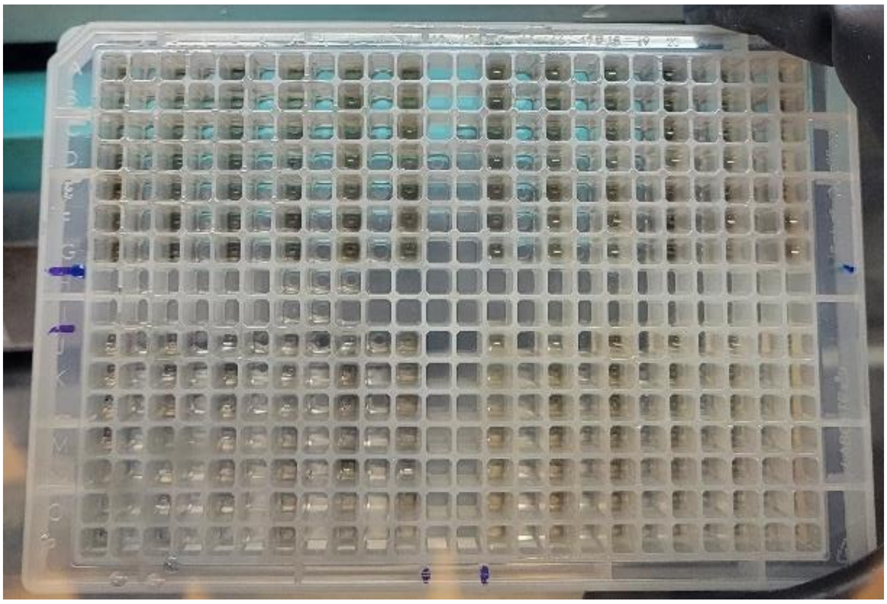

DMSO Ethylene Glycol

n-BuOH n-PeOH

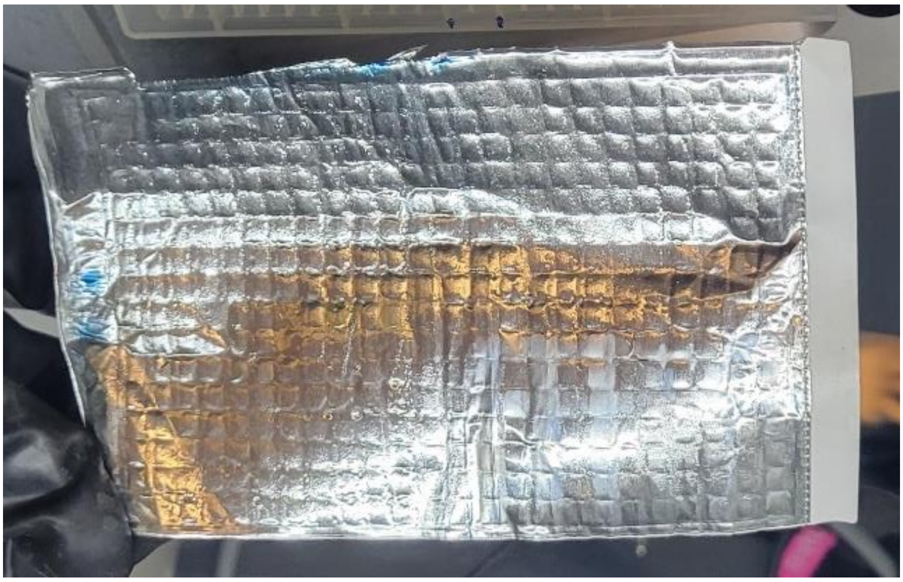

DMSO Ethylene Glycol

Figure S18. 384-well plate dosed with solvents, catalyst, and reductant (Top). Foil seal after heating well plate at 50°C for 24 hours (Bottom).

Significant loss of seal was observed for n-Butanol and n-Pentanol quadrants due to solvent deposition on the surface of the foil seal. Moderate solvent evaporation was also observed in the n-Butanol and n-Pentanol wells due to poor sealing over the heating period. The seal over DMSO and Ethylene Glycol wells remained intact post experiment with little to no solvent deposition or evaporation. From these results Ethylene Glycol was used for further HTE studies due to its low volatility and compatibility with HTE workflow.

8.4 Procedure for 1,536-Well Plate Experiment

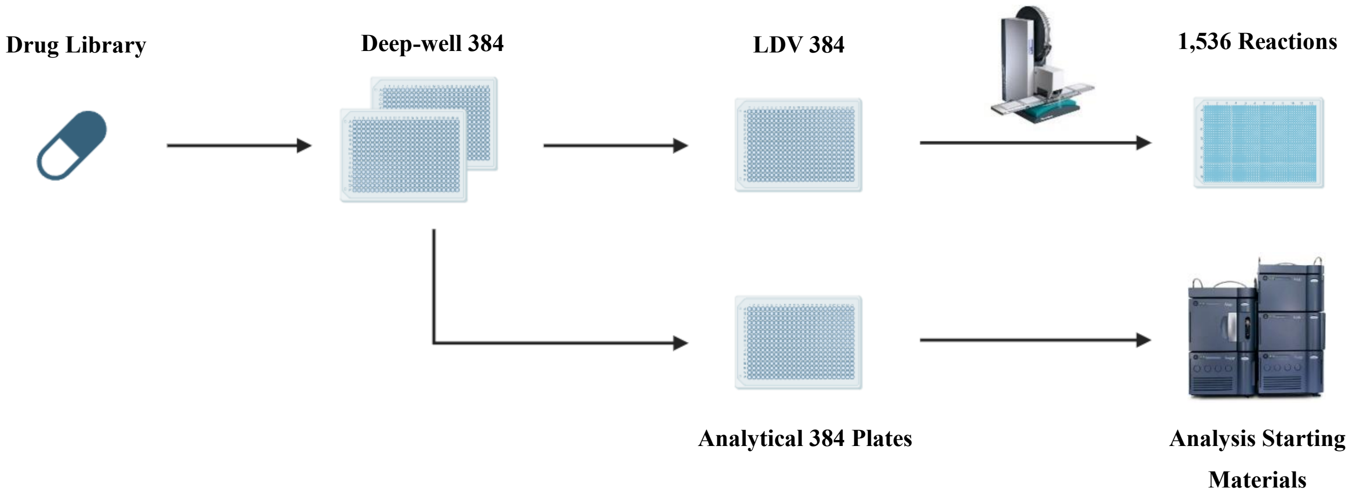

Samples of 734 druglike compounds in 5mM DMSO were sourced from the Dart Neurosciences collection, courtesy of the Center for Chemical Genomics at the University of Michigan Ann Arbor. The workflow for the 1,536-well experiment is outlines above (figure drawn in BioRender) To verify the ionization intensity and purity of the starting materials by UPLC-MS, 6 µL of each sample were transferred into two 384-well microtiter plates (Analytical Sales part No. 38435) using an SPT Labtech mosquito® liquid handling robot. An additional 24 µL of 1.25 mM caffeine in DMSO was then transferred into each well using multi-channel micropipettes to achieve a final concentration of 1 mM analyte and caffeine. The plates containing starting materials were then analyzed by UPLC-MS to determine ionization intensity relative to the caffeine internal standard prior to reaction. Following, another 27 µL of the original DMSO stock solutions were transferred into two low-dead volume (LDV) 384-well microtiter source plates (Greiner Bio-One part No. 784201) for evaporation and reconstitution of starting materials. The plates were subject to solvent evaporation using a

GeneVac HT-4X centrifugal evaporator until wells reached dryness after a two-hour evaporation cycle. Then, 30 μL aliquots of ethanol were added to each well and a second two-hour evaporation cycle was carried out. A final cycle of ethanol addition and evaporation was carried out once more to ensure complete DMSO removal from the samples. The samples were then reconstituted up to 20 mM by addition of 6.75 μL of anhydrous ethylene glycol to each well. Samples of the 34 starting materials reduced by rhodium catalysis in the prior **Experimental** section were also included in the library collection as 20 mM stock solutions in anhydrous ethylene glycol and 6.75 μL of each sample was transferred into the remaining wells in the low-dead volume 384-well source plates. From these 384- well source plates, 4 μL was transferred into the corresponding 2 wells in a 1,536 well reaction plate via mosquito (2 μL per reaction plate well, Analytical Sales part No. 15020). The first 384 well source plate was mapped to columns 1-12 and 25-36 of the 1,536-well reaction plate while the second 384- well source plate was mapped to columns 13-24 and 37-48 of the 1,536-well reaction plate. The 1,536-well reaction plate was subjected to a two-hour evaporation cycle to remove solvent. Stock solutions of $B_2(OH)_4$ (70 mM and 140 mM) in anhydrous ethylene glycol along with $[Rh(COD)OH]_2$ (0.8 mM) in anhydrous ethylene glycol were prepared separately and transferred into a third 384-well source plate via multichannel pipetting. From this source plate, 1 μL of 70 mM $B_2(OH)_4$ was aliquoted into wells in columns 1-24 and 1 μL of 140 mM $B_2(OH)_4$ was aliquoted into wells in columns 25-48 of the 1,536-well reaction plate. After subjecting the 1,536-well reaction plate to a four-hour solvent evaporation cycle to achieve dryness, 1 μL of rhodium catalyst stock solution was aliquoted into each well in the 1,536-well reaction plate, utilizing dispense mixing in triplicate to homogenize reaction well solutions. After dosing and mixing, the reaction plate was sealed with foil sealing tape (ThermoFisher part No. 232699) and centrifuged (Fisher Scientific Mini Plate Spinner) on full speed for ~1 minute. The reaction plate was then placed inside the plate heating apparatus (Eppendorf ThermoMixer C with Eppendorf ThermoTop attachment) to heat at 50°C for 24 hours. The foil seal was removed the reaction wells were quenched by addition of 2 μL of caffeine (120 mM) in DMSO, and the reaction plate centrifuged to settle and particulates. Reaction aliquots of 1 μL were then transferred into four 384-well microtiter plates pre-filled with 29 μL of HPLC-grade DMSO per well for analysis. The plates were then analyzed by UPLC-MS and results processed and visualized as described in the Supplementary Methods section. The output data is available on github (https://github.com/cernak-lab/late_stage_saturation). Example UPLC-MS traces and calculations of percent conversion for several wells from the 1,536-well experiment are shown below along with SMILES strings and yields for all compounds used.

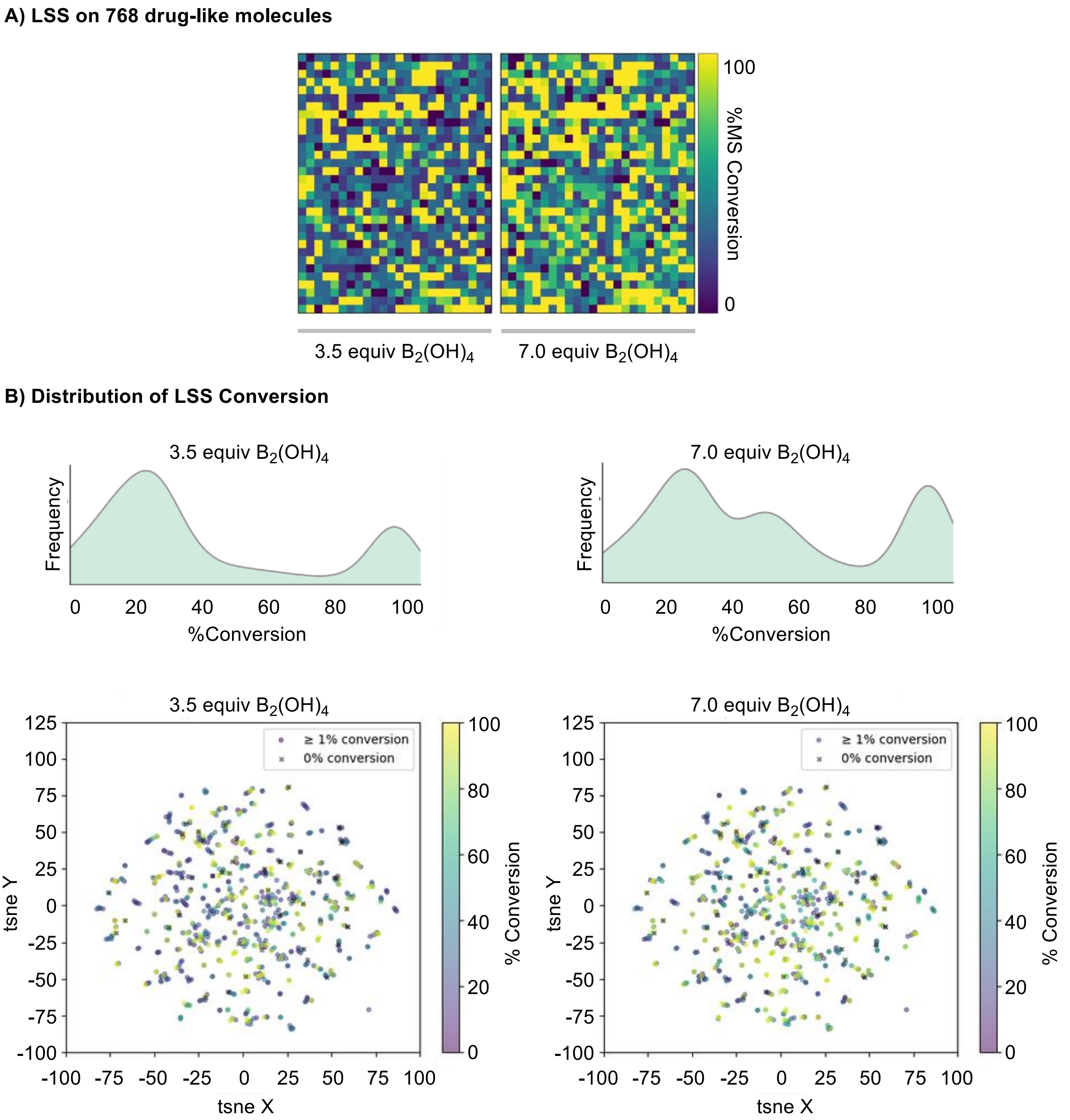

Figure S19. A) Heat map for 1,536-well plate experiment colored by percent conversion determined from UPLC-MS ionization peak area for 768 substrates subjected to two different reductant equivalents. B) Yield and chemical space distributions of 1,536-well plate experiment separated by equivalents of reductant used.

Well A28

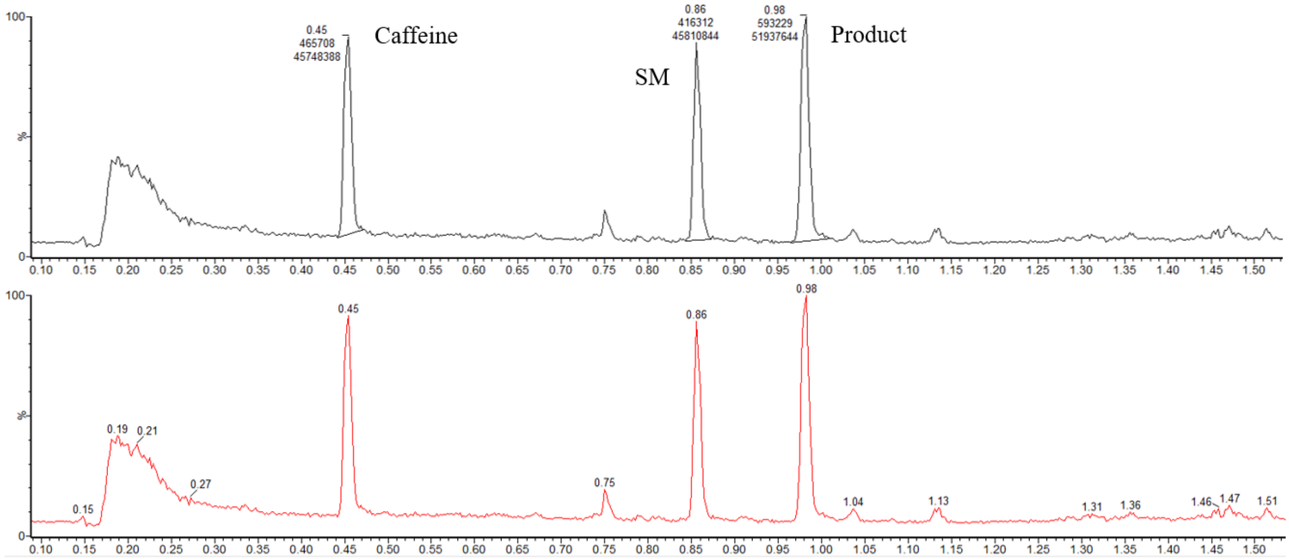

$$\%Conversion_{Product}^{MS} = 100 \times \left(\frac{\text{Area}_{\text{product}}}{Area_{sm} + Area_{product}}\right)$$

$$\%Conversion_{Product}^{MS} = 100 \times \left(\frac{51937644 \text{ counts}}{45810844\ counts + 51937644\ counts}\right)$$

$$\%Conversion_{Product}^{MS} = \mathbf{53.1}\%$$

Well B34

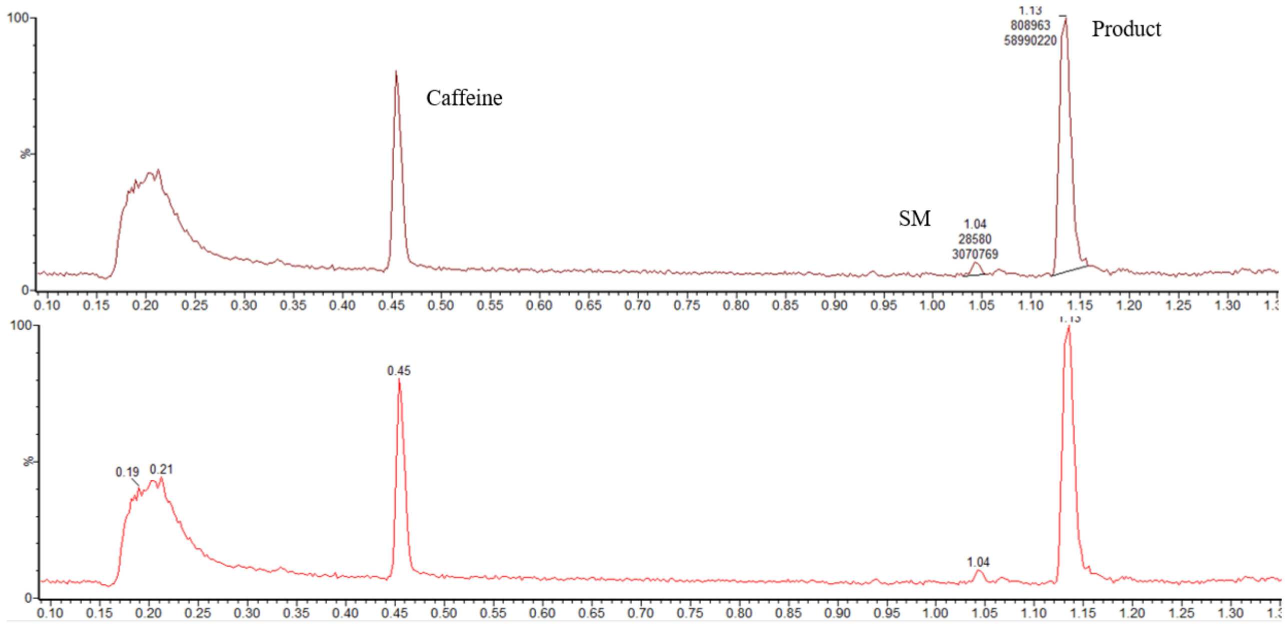

$$\%Conversion_{Product}^{MS} = 100 \times \left(\frac{\mathrm{Area}_{\mathrm{product}}}{Area_{sm} + Area_{product}}\right)$$

$$\%Conversion_{Product}^{MS} = 100 \times \left(\frac{58990220\ \mathrm{counts}}{3070769\ counts + 58990220\ counts}\right)$$

$$\%Conversion_{Product}^{MS} = \mathbf{95.1}\%$$

Well T32

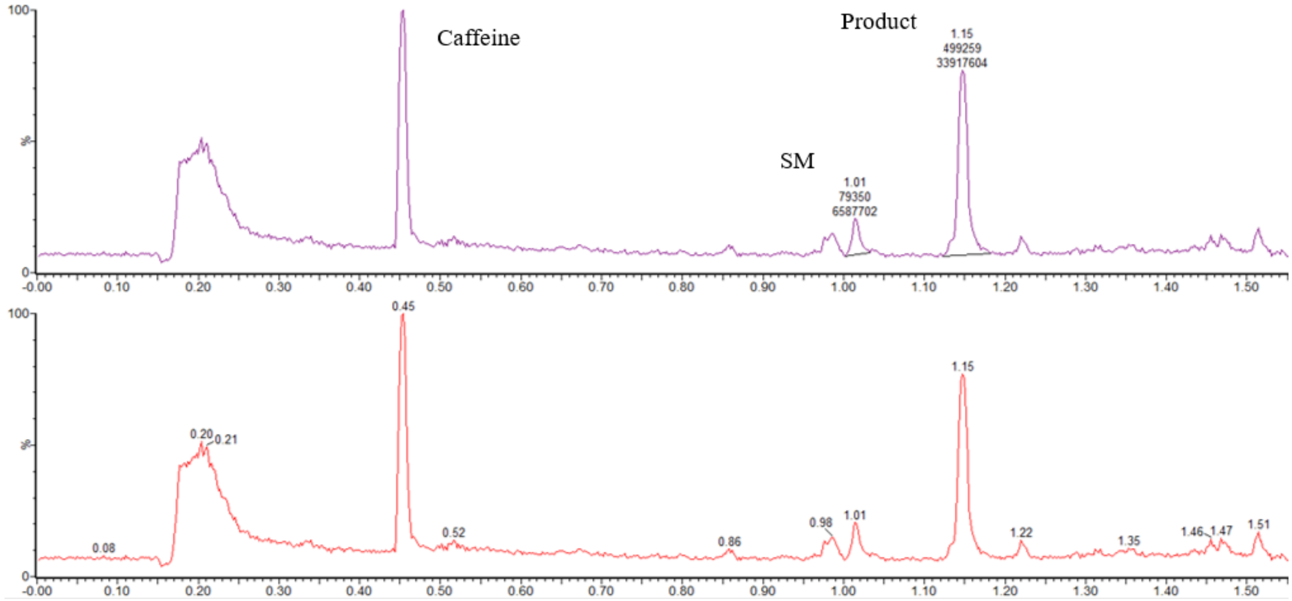

$$\%Conversion_{Product}^{MS} = 100 \times \left(\frac{\text{Area}_{\text{product}}}{Area_{sm} + Area_{product}}\right)$$

$$\%Conversion_{Product}^{MS} = 100 \times \left(\frac{33917604 \text{ counts}}{6587702\ counts + 33917604\ counts}\right)$$

$$\%Conversion_{Product}^{MS} = \mathbf{83.7}\%$$

8.5 SMILES Strings

Table S23. Data for 1,536-well plate HTE Experiment

Wells	Structure	SMILES String	%Conversion 3.5 equiv. (7.0 equiv.) $B_2(OH)_4$
A1/A25	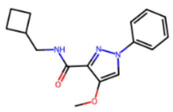	COC1=CN(N=C1C(=O)NCC2CCC2)C3=CC=CC=C3	35 (34)
A2/A26	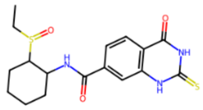	CCS(=O)C1CCCCC1NC(=O)C2=CC3=C(C=C2)C(=O)NC(=S)N3	0 (0)
A3/A27	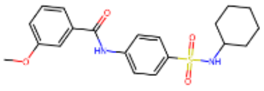	COC1=CC=CC(=C1)C(=O)NC2=CC=C(C=C2)S(=O)(=O)NC3CCCCC3	22 (0)
A4/A28	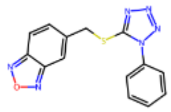	C1=CC=C(C=C1)N2C(=NN=N2)SCC3=CC4=NON=C4C=C3	27 (53)
A5/A29	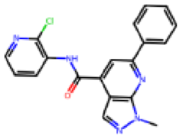	CN1C2=C(C=N1)C(=CC(=N2)C3=CC=CC=C3)C(=O)NC4=C(N=CC=C4)Cl	6 (21)
A6/A30	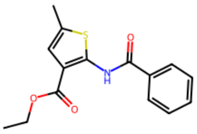	CCOC(C1=C(NC(C2=CC=CC=C2)=O)SC(C)=C1)=O	23 (27)
A7/A31	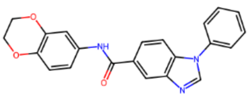	C1COC2=C(O1)C=CC(=C2)NC(=O)C3=CC4=C(C=C3)N(C=N4)C5=CC=CC=C5	89 (94)
A8/A32	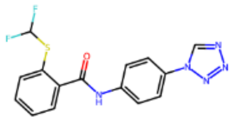	C1=CC=C(C(=C1)C(=O)NC2=CC=C(C=C2)N3C=NN=N3)SC(F)F	27 (50)
A9/A33	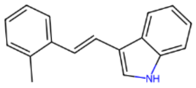	CC1=CC=CC=C1C=CC2=CNC3=CC=CC=C32	22 (52)
A10/A34	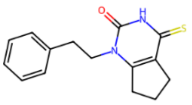	C1CC2=C(C1)N(C(=O)NC2=S)CCC3=CC=CC=C3	57 (53)

**A11/A35**	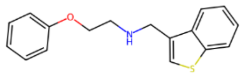	C1=CC=C(C=C1)OCCNCC2=CSC3=CC=CC=C32	11 (10)
**A12/A36**	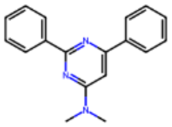	CN(C)C1=NC(=NC(=C1)C2=CC=CC=C2)C3=CC=CC=C3	18 (88)
**A13/A37**	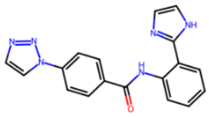	C1=CC=C(C(=C1)C2=NC=CN2)NC(=O)C3=CC=C(C=C3)N4C=CN=N4	88 (93)
**A14/A38**	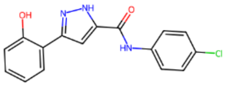	C1=CC=C(C(=C1)C2=NNC(=C2)C(=O)NC3=CC=C(C=C3)Cl)O	11 (70)
**A15/A39**	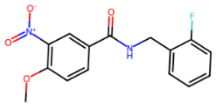	COC1=C(C=C(C=C1)C(=O)NCC2=CC=CC=C2F)[N+](=O)[O-]	2 (21)
**A16/A40**	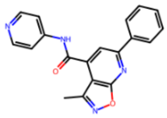	CC1=NOC2=C1C(=CC(=N2)C3=CC=CC=C3)C(=O)NC4=CC=NC=C4	7 (19)
**A17/A41**	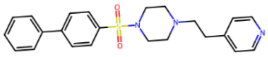	C1CN(CCN1CCC2=CC=NC=C2)S(=O)(=O)C3=CC=C(C=C3)C4=CC=CC=C4	21 (30)
**A18/A42**	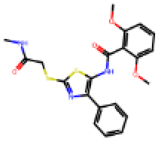	CNC(=O)CSC1=NC(=C(S1)NC(=O)C2=C(C=CC=C2OC)OC)C3=CC=CC=C3	16 (32)
**A19/A43**	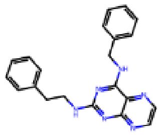	C1=CC=C(C=C1)CCNC2=NC3=NC=CN=C3C(=N2)NCC4=CC=CC=C4	16 (22)
**A20/A44**	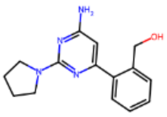	C1CCN(C1)C2=NC(=CC(=N2)N)C3=CC=CC=C3CO	24 (17)
**A21/A45**	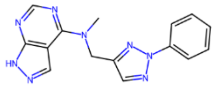	CN(CC1=NN(N=C1)C2=CC=CC=C2)C3=NC=NC4=C3C=NN4	11 (25)
**A22/A46**	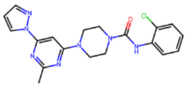	CC1=NC(=CC(=N1)N2CCN(CC2)C(=O)NC3=CC=CC=C3Cl)N4C=CC=N4	34 (79)

**A23/A47**	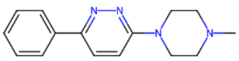	CN1CCN(CC1)C2=NN=C(C=C2)C3=CC=CC=C3	22 (20)
**A24/A48**	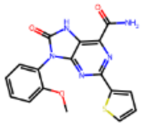	COC1=CC=CC=C1N2C3=NC(=NC(=C3NC2=O)C(=O)N)C4=CC=CS4	23 (22)
**B1/B25**	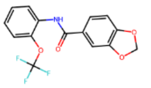	C1OC2=C(O1)C=C(C=C2)C(=O)NC3=CC=CC=C3OC(F)(F)F	0 (49)
**B2/B26**	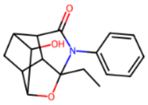	CCC12C3C4CC(C(O)C4O2)C3C(N1C5=CC=CC=C5)=O	20 (0)
**B3/B27**	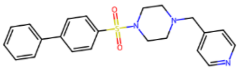	C1CN(CCN1CC2=CC=NC=C2)S(=O)(=O)C3=CC=C(C=C3)C4=CC=CC=C4	90 (88)
**B4/B28**	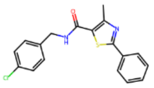	CC1=C(SC(=N1)C2=CC=CC=C2)C(=O)NCC3=CC=C(C=C3)Cl	32 (36)
**B5/B29**	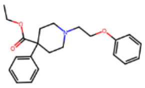	CCOC(=O)C1(CCN(CC1)CCOC2=CC=CC=C2)C3=CC=CC=C3	79 (81)
**B6/B30**	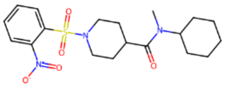	CN(C1CCCCC1)C(=O)C2CCN(CC2)S(=O)(=O)C3=CC=CC=C3[N+](=O)[O-]	27 (22)
**B7/B31**	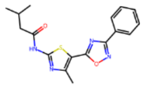	CC1=C(SC(=N1)NC(=O)CC(C)C)C2=NC(=NO2)C3=CC=CC=C3	40 (39)
**B8/B32**	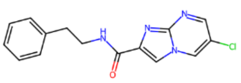	C1=CC=C(C=C1)CCNC(=O)C2=CN3C=C(C=NC3=N2)Cl	10 (45)
**B9/B33**	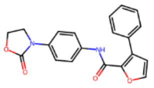	C1COC(=O)N1C2=CC=C(C=C2)NC(=O)C3=C(C=CO3)C4=CC=CC=C4	6 (49)
**B10/B34**	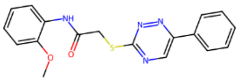	COC1=CC=CC=C1NC(=O)CSC2=NC=C(N=N2)C3=CC=CC=C3	81 (95)

**B11/B35**	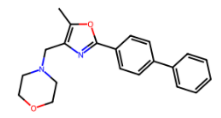	CC1=C(N=C(O1)C2=CC=C(C=C2)C3=CC=CC=C3)CN4CCOCC4	26 (31)
**B12/B36**	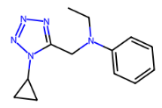	CCN(C1=CC=CC=C1)CC2=NN=NN2C3CC3	18 (22)
**B13/B37**	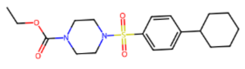	CCOC(=O)N1CCN(CC1)S(=O)(=O)C2=CC=C(C=C2)C3CCCCC3	25 (63)
**B14/B38**	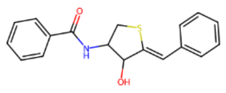	C1C(C(C(=CC2=CC=CC=C2)S1)O)NC(=O)C3=CC=CC=C3	33 (63)
**B15/B39**	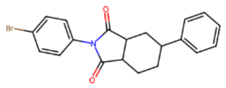	C1CC2C(CC1C3=CC=CC=C3)C(=O)N(C2=O)C4=CC=C(C=C4)Br	46 (28)
**B16/B40**	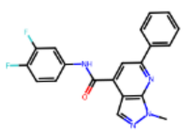	CN1C2=C(C=N1)C(=CC(=N2)C3=CC=CC=C3)C(=O)NC4=CC(=C(C=C4)F)F	94 (96)
**B17/B41**	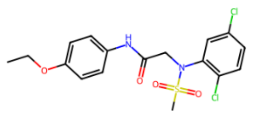	CCOC1=CC=C(C=C1)NC(=O)CN(C2=C(C=CC(=C2)Cl)Cl)S(=O)(=O)C	90 (97)
**B18/B42**	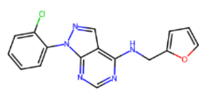	C1=CC=C(C(=C1)N2C3=NC=NC(=C3C=N2)NCC4=CC=CO4)Cl	92 (92)
**B19/B43**	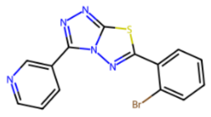	C1=CC=C(C(=C1)C2=NN3C(=NN=C3S2)C4=CN=CC=C4)Br	92 (94)
**B20/B44**	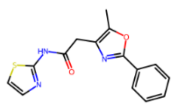	CC1=C(N=C(O1)C2=CC=CC=C2)CC(=O)NC3=NC=CS3	12 (13)
**B21/B45**	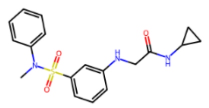	CN(C1=CC=CC=C1)S(=O)(=O)C2=CC=CC(=C2)NCC(=O)NC3CC3	11 (19)
**B22/B46**	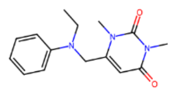	CCN(CC1=CC(=O)N(C(=O)N1C)C)C2=CC=CC=C2	52 (40)

**B23/B47**	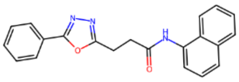	C1=CC=C(C=C1)C2=NN=C(O2)CCC(=O)NC3=CC=CC4=CC=CC=C43	25 (93)
**B24/B48**	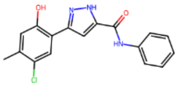	CC1=CC(=C(C=C1Cl)C2=NNC(=C2)C(=O)NC3=CC=CC=C3)O	7 (44)
**C1/C25**	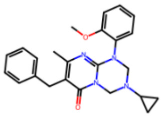	CC1=C(C(=O)N2CN(CN(C2=N1)C3=CC=CC=C3OC)C4CC4)CC5=CC=CC=C5	74 (70)
**C2/C26**	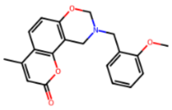	CC1=CC(=O)OC2=C1C=CC3=C2CN(CO3)CC4=CC=CC=C4OC	23 (43)
**C3/C27**	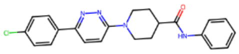	C1CN(CCC1C(=O)NC2=CC=CC=C2)C3=NN=C(C=C3)C4=CC=C(C=C4)Cl	11 (50)
**C4/C28**	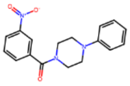	C1CN(CCN1C2=CC=CC=C2)C(=O)C3=CC(=CC=C3)[N+](=O)[O-]	0 (4)
**C5/C29**	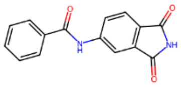	C1=CC=C(C=C1)C(=O)NC2=CC3=C(C=C2)C(=O)NC3=O	29 (52)
**C6/C30**	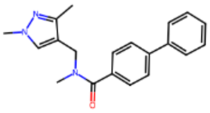	CC1=NN(C=C1CN(C)C(=O)C2=CC=C(C=C2)C3=CC=CC=C3)C	32 (31)
**C7/C31**	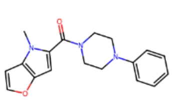	CN1C2=C(C=C1C(=O)N3CCN(CC3)C4=CC=CC=C4)OC=C2	50 (96)
**C8/C32**	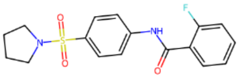	C1CCN(C1)S(=O)(=O)C2=CC=C(C=C2)NC(=O)C3=CC=CC=C3F	8 (26)
**C9/C33**	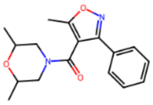	CC1CN(CC(O1)C)C(=O)C2=C(ON=C2C3=CC=CC=C3)C	91 (89)
**C10/C34**	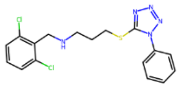	C1=CC=C(C=C1)N2C(=NN=N2)SCCCNCC3=C(C=CC=C3Cl)Cl	31 (31)

**C11/C35**	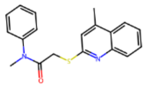	CC1=CC(=NC2=CC=CC=C12)SCC(=O)N(C)C3=CC=CC=C3	28 (63)
**C12/C36**	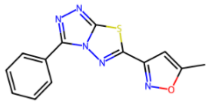	CC1=CC(C2=NN3C(C4=CC=CC=C4)=NN=C3S2)=NO1	93 (95)
**C13/C37**	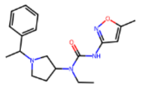	CCN(C1CCN(C1)C(C)C2=CC=CC=C2)C(=O)NC3=NOC(=C3)C	20 (23)
**C14/C38**	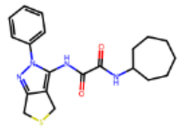	C1CCCC(CC1)NC(=O)C(=O)NC2=C3CSCC3=NN2C4=CC=CC=C4	26 (15)
**C15/C39**	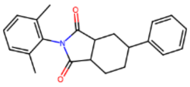	CC1=C(C(=CC=C1)C)N2C(=O)C3CCC(CC3C2=O)C4=CC=CC=C4	95 (88)
**C16/C40**	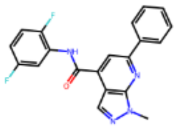	CN1C2=C(C=N1)C(=CC(=N2)C3=CC=CC=C3)C(=O)NC4=C(C=CC(=C4)F)F	94 (98)
**C17/C41**	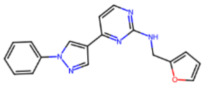	C1=CC=C(C=C1)N2C=C(C=N2)C3=NC(=NC=C3)NCC4=CC=CO4	96 (97)
**C18/C42**	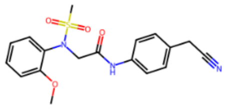	COC1=CC=CC=C1N(CC(=O)NC2=CC=C(C=C2)CC#N)S(=O)(=O)C	16 (42)
**C19/C43**	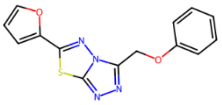	C1=CC=C(C=C1)OCC2=NN=C3N2N=C(S3)C4=CC=CO4	29 (21)
**C20/C44**	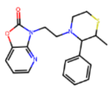	CC1C(N(CCS1)CCN2C3=C(C=CC=N3)OC2=O)C4=CC=CC=C4	36 (39)
**C21/C45**	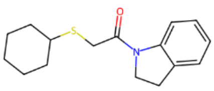	C1CCC(CC1)SCC(=O)N2CCC3=CC=CC=C32	51 (47)
**C22/C46**	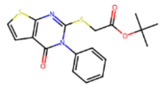	CC(C)(C)OC(=O)CSC1=NC2=C(C=CS2)C(=O)N1C3=CC=CC=C3	12 (32)

**C23/C47**	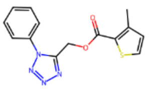	CC1=C(SC=C1)C(=O)OCC2=NN=NN2C3=CC=CC=C3	28 (25)
**C24/C48**	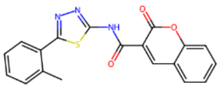	CC1=CC=CC=C1C2=NN=C(S2)NC(=O)C3=CC4=CC=CC=C4OC3=O	26 (19)
**D1/D25**	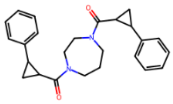	C1CN(CCN(C1)C(=O)C2CC2C3=CC=CC=C3)C(=O)C4CC4C5=CC=CC=C5	21 (20)
**D2/D26**	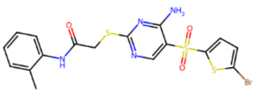	CC1=CC=CC=C1NC(=O)CSC2=NC=C(C(=N2)N)S(=O)(=O)C3=CC=C(S3)Br	91 (92)
**D3/D27**	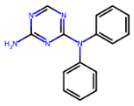	C1=CC=C(C=C1)N(C2=CC=CC=C2)C3=NC=NC(=N3)N	24 (63)
**D4/D28**	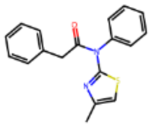	CC1=CSC(=N1)N(C2=CC=CC=C2)C(=O)CC3=CC=CC=C3	89 (97)
**D5/D29**	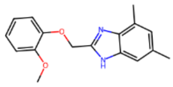	CC1=CC(=C2C(=C1)NC(=N2)COC3=CC=CC=C3OC)C	91 (90)
**D6/D30**	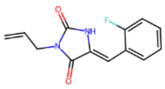	C=CCN1C(=O)C(=CC2=CC=CC=C2F)NC1=O	31 (64)
**D7/D31**	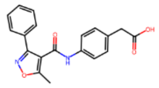	CC1=C(C(=NO1)C2=CC=CC=C2)C(=O)NC3=CC=C(C=C3)CC(=O)O	38 (21)
**D8/D32**	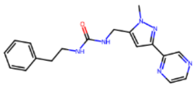	CN1C(=CC(=N1)C2=NC=CN=C2)CNC(=O)NCCC3=CC=CC=C3	88 (95)
**D9/D33**	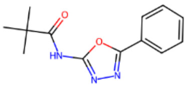	CC(C)(C)C(=O)NC1=NN=C(O1)C2=CC=CC=C2	19 (15)
**D10/D34**	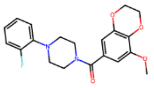	COC1=CC(=CC2=C1OCCO2)C(=O)N3CCN(CC3)C4=CC=CC=C4F	95 (95)

**D11/D35**	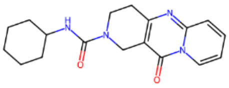	C1CCC(CC1)NC(=O)N2CCC3=C(C2)C(=O)N4C=CC=CC4=N3	14 (31)
**D12/D36**	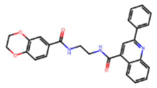	C1COC2=C(O1)C=CC(=C2)C(=O)NCCNC(=O)C3=CC(=NC4=CC=CC=C43)C5=CC=CC=C5	57 (52)
**D13/D37**	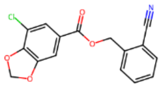	C1OC2=C(O1)C(=CC(=C2)C(=O)OCC3=CC=CC=C3C#N)Cl	13 (18)
**D14/D38**	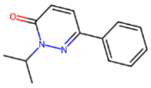	CC(C)N1C(=O)C=CC(=N1)C2=CC=CC=C2	28 (0)
**D15/D39**	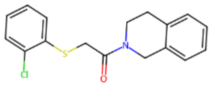	C1CN(CC2=CC=CC=C21)C(=O)CSC3=CC=CC=C3Cl	89 (97)
**D16/D40**	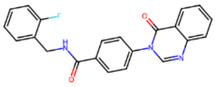	C1=CC=C(C(=C1)CNC(=O)C2=CC=C(C=C2)N3C=NC4=CC=CC=C4C3=O)F	89 (90)
**D17/D41**	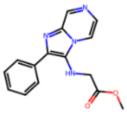	COC(=O)CNC1=C(N=C2N1C=CN=C2)C3=CC=CC=C3	79 (91)
**D18/D42**	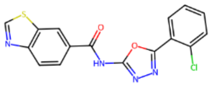	C1=CC=C(C(=C1)C2=NN=C(O2)NC(=O)C3=CC4=C(C=C3)N=CS4)Cl	12 (44)
**D19/D43**	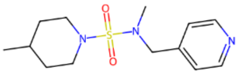	CC1CCN(CC1)S(=O)(=O)N(C)CC2=CC=NC=C2	27 (27)
**D20/D44**	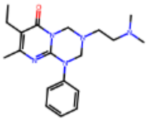	CCC1=C(N=C2N(CN(CN2C1=O)CCN(C)C)C3=CC=CC=C3)C	28 (10)
**D21/D45**	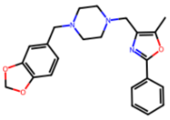	CC1=C(N=C(O1)C2=CC=CC=C2)CN3CCN(CC3)CC4=CC5=C(C=C4)OCO5	0 (26)
**D22/D46**	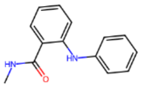	CNC(=O)C1=CC=CC=C1NC2=CC=CC=C2	32 (15)

**D23/D47**	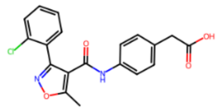	CC1=C(C(=NO1)C2=CC=CC=C2Cl)C(=O)NC3=CC=C(C=C3)CC(=O)O	27 (23)
**D24/D48**	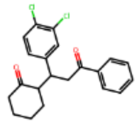	C1CCC(=O)C(C1)C(CC(=O)C2=CC=CC=C2)C3=CC(=C(C=C3)Cl)Cl	7 (10)
**E1/E25**	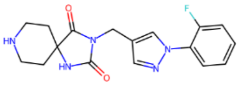	C1CNCCC12C(=O)N(C(=O)N2)CC3=CN(N=C3)C4=CC=CC=C4F	72 (96)
**E2/E26**	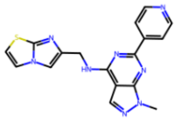	CN1C2=NC(=NC(=C2C=N1)NCC3=CN4C=CSC4=N3)C5=CC=NC=C5	22 (13)
**E3/E27**	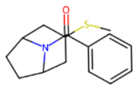	CSC1CC2CCC(C1)N2C(=O)C3=CC=CC=C3	89 (95)
**E4/E28**	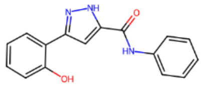	C1=CC=C(C=C1)NC(=O)C2=CC(=NN2)C3=CC=CC=C3O	20 (28)
**E5/E29**	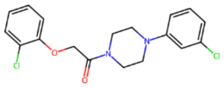	C1CN(CCN1C2=CC(=CC=C2)Cl)C(=O)COC3=CC=CC=C3Cl	96 (93)
**E6/E30**	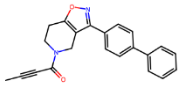	CC#CC(=O)N1CCC2=C(C1)C(=NO2)C3=CC=C(C=C3)C4=CC=CC=C4	89 (91)
**E7/E31**	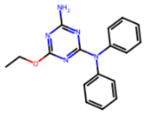	CCOC1=NC(=NC(=N1)N(C2=CC=CC=C2)C3=CC=CC=C3)N	18 (21)
**E8/E32**	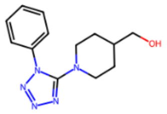	C1CN(CCC1CO)C2=NN=NN2C3=CC=CC=C3	19 (49)
**E9/E33**	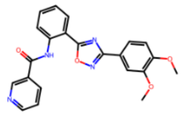	COC1=C(C=C(C=C1)C2=NOC(=N2)C3=CC=CC=C3NC(=O)C4=CN=CC=C4)OC	26 (0)
**E10/E34**	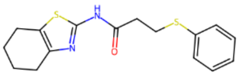	C1CCC2=C(C1)N=C(S2)NC(=O)CCSC3=CC=CC=C3	24 (27)

**E11/E35**	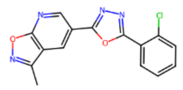	CC1=NOC2=C1C=C(C=N2)C3=NN=C(O3)C4=CC=CC=C4Cl	34 (38)
**E12/E36**	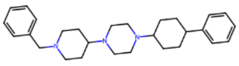	C1CC(CCC1C2=CC=CC=C2)N3CCN(CC3)C4CCN(CC4)CC5=CC=CC=C5	17 (22)
**E13/E37**	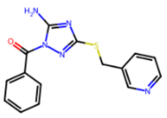	C1=CC=C(C=C1)C(=O)N2C(=NC(=N2)SCC3=CN=CC=C3)N	26 (14)
**E14/E38**	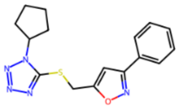	C1CCC(C1)N2C(=NN=N2)SCC3=CC(=NO3)C4=CC=CC=C4	27 (53)
**E15/E39**	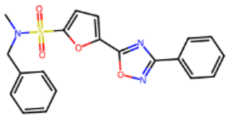	CN(CC1=CC=CC=C1)S(=O)(=O)C2=CC=C(O2)C3=NC(=NO3)C4=CC=CC=C4	24 (10)
**E16/E40**	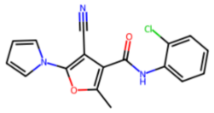	CC1=C(C(=C(O1)N2C=CC=C2)C#N)C(=O)NC3=CC=CC=C3Cl	28 (60)
**E17/E41**	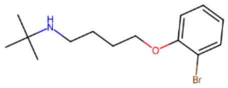	CC(C)(C)NCCCCOC1=CC=CC=C1Br	8 (25)
**E18/E42**	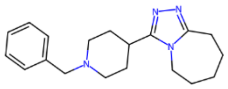	C1CCC2=NN=C(N2CC1)C3CCN(CC3)CC4=CC=CC=C4	0 (18)
**E19/E43**	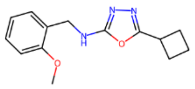	COC1=CC=CC=C1CNC2=NN=C(O2)C3CCC3	16 (22)
**E20/E44**	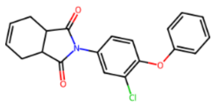	C1C=CCC2C1C(=O)N(C2=O)C3=CC(=C(C=C3)OC4=CC=CC=C4)Cl	25 (25)
**E21/E45**	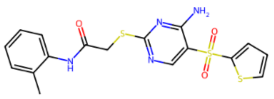	CC1=CC=CC=C1NC(=O)CSC2=NC=C(C(=N2)N)S(=O)(=O)C3=CC=CS3	34 (31)
**E22/E46**	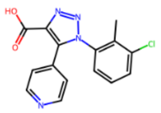	CC1=C(C=CC=C1Cl)N2C(=C(N=N2)C(=O)O)C3=CC=NC=C3	25 (11)

**E23/E47**	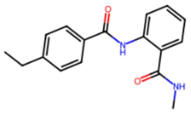	CCC1=CC=C(C=C1)C(=O)NC2=CC=CC=C2C(=O)NC	17 (18)
**E24/E48**	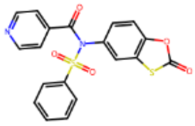	C1=CC=C(C=C1)S(=O)(=O)N(C2=CC3=C(C=C2)OC(=O)S3)C(=O)C4=CC=NC=C4	8 (45)
**F1/F25**	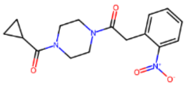	C1CC1C(=O)N2CCN(CC2)C(=O)CC3=CC=CC=C3[N+](=O)[O-]	26 (49)
**F2/F26**	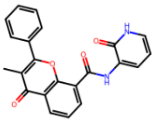	CC1=C(OC2=C(C1=O)C=CC=C2C(=O)NC3=CC=CNC3=O)C4=CC=CC=C4	8 (13)
**F3/F27**	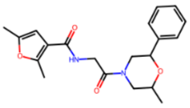	CC1CN(CC(O1)C2=CC=CC=C2)C(=O)CNC(=O)C3=C(OC(=C3)C)C	19 (20)
**F4/F28**	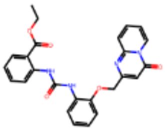	CCOC(=O)C1=CC=CC=C1NC(=O)NC2=CC=CC=C2OCC3=CC(=O)N4C=CC=CC4=N3	17 (12)
**F5/F29**	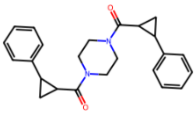	C1CN(CCN1C(=O)C2CC2C3=CC=CC=C3)C(=O)C4CC4C5=CC=CC=C5	7 (49)
**F6/F30**	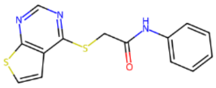	C1=CC=C(C=C1)NC(=O)CSC2=NC=NC3=C2C=CS3	96 (94)
**F7/F31**	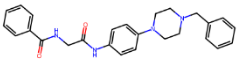	C1CN(CCN1CC2=CC=CC=C2)C3=CC=C(C=C3)NC(=O)CNC(=O)C4=CC=CC=C4	9 (27)
**F8/F32**	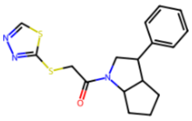	C1CC2C(C1)N(CC2C3=CC=CC=C3)C(=O)CSC4=NN=CS4	0 (8)
**F9/F33**	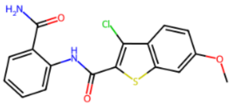	COC1=CC2=C(C=C1)C(=C(S2)C(=O)NC3=CC=CC=C3C(=O)N)Cl	0 (0)

**F10/F34**	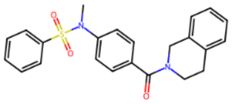	CN(C1=CC=C(C=C1)C(=O)N2CCC3=CC=CC=C3C2)S(=O)(=O)C4=CC=CC=C4	0 (51)
**F11/F35**	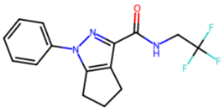	C1CC2=C(C1)N(N=C2C(=O)NCC(F)(F)F)C3=CC=CC=C3	27 (10)
**F12/F36**	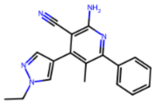	CCN1C=C(C=N1)C2=C(C(=NC(=C2C#N)N)C3=CC=CC=C3)C	26 (0)
**F13/F37**	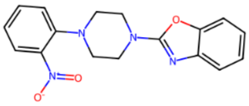	C1CN(CCN1C2=CC=CC=C2[N+](=O)[O-])C3=NC4=CC=CC=C4O3	90 (91)
**F14/F38**	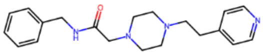	C1CN(CCN1CCC2=CC=NC=C2)CC(=O)NCC3=CC=CC=C3	97 (93)
**F15/F39**	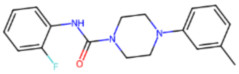	CC1=CC(=CC=C1)N2CCN(CC2)C(=O)NC3=CC=CC=C3F	97 (97)
**F16/F40**	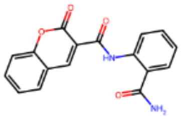	C1=CC=C2C(=C1)C=C(C(=O)O2)C(=O)NC3=CC=CC=C3C(=O)N	11 (23)
**F17/F41**	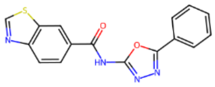	C1=CC=C(C=C1)C2=NN=C(O2)NC(=O)C3=CC4=C(C=C3)N=CS4	22 (45)
**F18/F42**	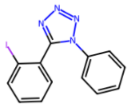	C1=CC=C(C=C1)N2C(=NN=N2)C3=CC=CC=C3I	93 (89)
**F19/F43**	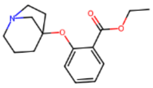	CCOC(=O)C1=CC=CC=C1OC23CCCN(C2)CC3	10 (9)
**F20/F44**	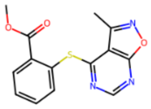	CC1=NOC2=C1C(=NC=N2)SC3=CC=CC=C3C(=O)OC	36 (80)
**F21/F45**	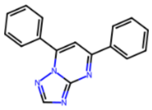	C1=CC=C(C=C1)C2=CC(=NC3=NC=NN23)C4=CC=CC=C4	14 (11)

**F22/F46**	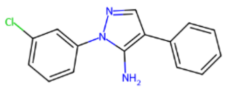	C1=CC=C(C=C1)C2=C(N(N=C2)C3=CC(=CC=C3)Cl)N	90 (97)
**F23/F47**	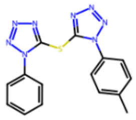	CC1=CC=C(C=C1)N2C(=NN=N2)SC3=NN=NN3C4=CC=CC=C4	29 (72)
**F24/F48**	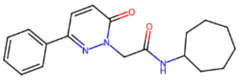	C1CCCC(CC1)NC(=O)CN2C(=O)C=CC(=N2)C3=CC=CC=C3	7 (20)
**G1/G25**	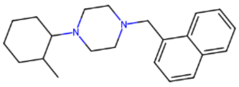	CC1CCCCC1N2CCN(CC2)CC3=CC=CC4=CC=CC=C43	19 (19)
**G2/G26**	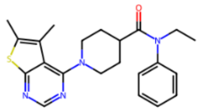	CCN(C1=CC=CC=C1)C(=O)C2CCN(CC2)C3=C4C(=C(SC4=NC=N3)C)C	88 (91)
**G3/G27**	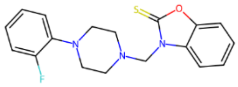	C1CN(CCN1CN2C3=CC=CC=C3OC2=S)C4=CC=CC=C4F	91 (89)
**G4/G28**	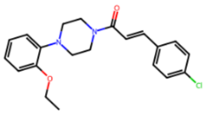	CCOC1=CC=CC=C1N2CCN(CC2)C(=O)C=CC3=CC=C(C=C3)Cl	21 (19)
**G5/G29**	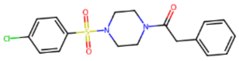	C1CN(CCN1C(=O)CC2=CC=CC=C2)S(=O)(=O)C3=CC=C(C=C3)Cl	12 (24)
**G6/G30**	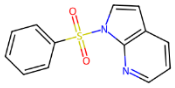	C1=CC=C(C=C1)S(=O)(=O)N2C=CC3=C2N=CC=C3	98 (97)
**G7/G31**	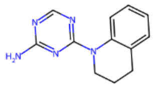	C1CC2=CC=CC=C2N(C1)C3=NC=NC(=N3)N	7 (22)
**G8/G32**	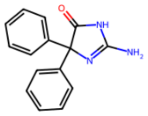	C1=CC=C(C=C1)C2(C(=O)NC(=N2)N)C3=CC=CC=C3	16 (13)
**G9/G33**	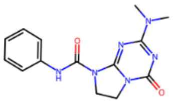	CN(C)C1=NC(=O)N2CCN(C2=N1)C(=O)NC3=CC=CC=C3	98 (91)

**G10/G34**	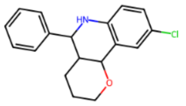	C1CC2C(NC3=C(C2OC1)C=C(C=C3)Cl)C4=CC=CC=C4	97 (85)
**G11/G35**	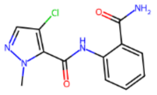	CN1C(=C(C=N1)Cl)C(=O)NC2=CC=CC=C2C(=O)N	92 (97)
**G12/G36**	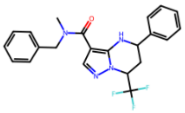	CN(CC1=CC=CC=C1)C(=O)C2=C3NC(CC(N3N=C2)C(F)(F)F)C4=CC=CC=C4	88 (93)
**G13/G37**	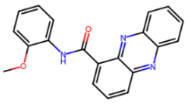	COC1=CC=CC=C1NC(=O)C2=CC=CC3=NC4=CC=CC=C4N=C32	5 (0)
**G14/G38**	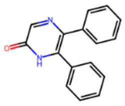	C1=CC=C(C=C1)C2=C(N=CC(=O)N2)C3=CC=CC=C3	91 (89)
**G15/G39**	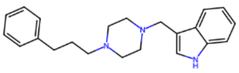	C1CN(CCN1CCCC2=CC=CC=C2)CC3=CNC4=CC=CC=C43	97 (98)
**G16/G40**	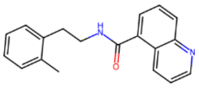	CC1=CC=CC=C1CCNC(=O)C2=C3C=CC=NC3=CC=C2	20 (45)
**G17/G41**	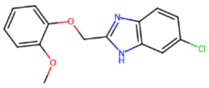	COC1=CC=CC=C1OCC2=NC3=C(N2)C=C(C=C3)Cl	95 (89)
**G18/G42**	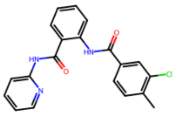	CC1=C(C=C(C=C1)C(=O)NC2=CC=CC=C2C(=O)NC3=CC=CC=N3)Cl	90 (97)
**G19/G43**	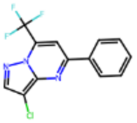	C1=CC=C(C=C1)C2=NC3=C(C=NN3C(=C2)C(F)(F)F)Cl	21 (48)
**G20/G44**	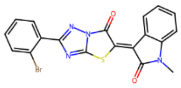	CN1C2=CC=CC=C2C(=C3C(=O)N4C(=NC(=N4)C5=CC=CC=C5Br)S3)C1=O	21 (51)
**G21/G45**	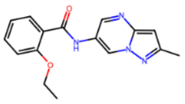	CCOC1=CC=CC=C1C(=O)NC2=CN3C(=CC(=N3)C)N=C2	92 (94)

**G22/G46**	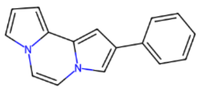	C1=CC=C(C=C1)C2=CN3C=CN4C=CC=C4C3=C2	95 (98)
**G23/G47**	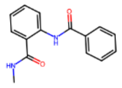	CNC(=O)C1=CC=CC=C1NC(=O)C2=CC=CC=C2	0 (27)
**G24/G48**	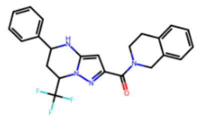	C1CN(CC2=CC=CC=C21)C(=O)C3=NN4C(CC(NC4=C3)C5=CC=CC=C5)C(F)(F)F	91 (89)
**H1/H25**	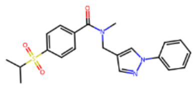	CC(C)S(=O)(=O)C1=CC=C(C=C1)C(=O)N(C)CC2=CN(N=C2)C3=CC=CC=C3	60 (98)
**H2/H26**	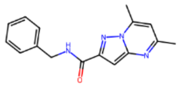	CC1=CC(=NC2=CC(=NN12)C(=O)NCC3=CC=CC=C3)C	66 (65)
**H3/H27**	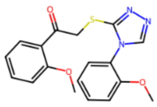	COC1=CC=CC=C1C(=O)CSC2=NN=CN2C3=CC=CC=C3OC	89 (90)
**H4/H28**	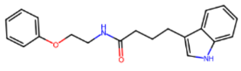	C1=CC=C(C=C1)OCCNC(=O)CCCC2=CNC3=CC=CC=C32	91 (98)
**H5/H29**	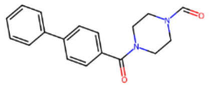	C1CN(CCN1C=O)C(=O)C2=CC=C(C=C2)C3=CC=CC=C3	98 (93)
**H6/H30**	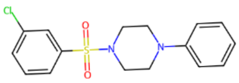	C1CN(CCN1C2=CC=CC=C2)S(=O)(=O)C3=CC(=CC=C3)Cl	0 (25)
**H7/H31**	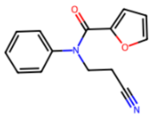	C1=CC=C(C=C1)N(CCC#N)C(=O)C2=CC=CO2	29 (52)
**H8/H32**	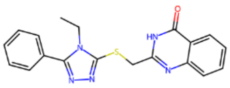	CCN1C(=NN=C1SCC2=NC3=CC=CC=C3C(=O)N2)C4=CC=CC=C4	98 (92)

**H9/H33**	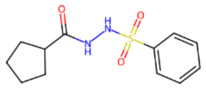	C1CCC(C1)C(=O)NNS(=O)(=O)C2=CC=CC=C2	97 (89)
**H10/H34**	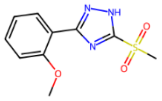	COC1=CC=CC=C1C2=NNC(=N2)S(=O)(=O)C	95 (88)
**H11/H35**	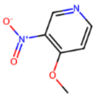	COC1=C(C=NC=C1)[N+](=O)[O-]	43 (89)
**H12/H36**	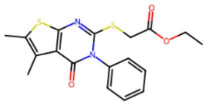	CCOC(CSC1=NC2=C(C(N1C3=CC=CC=C3)=O)C(C)=C(C)S2)=O	93 (91)
**H13/H37**	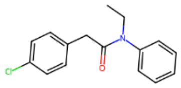	CCN(C1=CC=CC=C1)C(=O)CC2=CC=C(C=C2)Cl	93 (90)
**H14/H38**	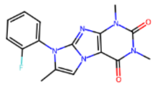	CC1=CN2C3=C(N=C2N1C4=CC=CC=C4F)N(C(=O)N(C3=O)C)C	52 (93)
**H15/H39**	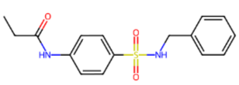	CCC(=O)NC1=CC=C(C=C1)S(=O)(=O)NCC2=CC=CC=C2	89 (86)
**H16/H40**	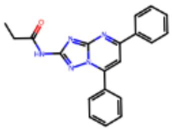	CCC(=O)NC1=NN2C(=CC(=NC2=N1)C3=CC=CC=C3)C4=CC=CC=C4	96 (96)
**H17/H41**	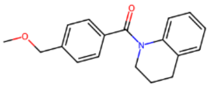	COCC1=CC=C(C=C1)C(=O)N2CCCC3=CC=CC=C32	0 (11)
**H18/H42**	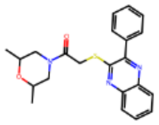	CC1CN(CC(O1)C)C(=O)CSC2=NC3=CC=CC=C3N=C2C4=CC=CC=C4	0 (0)
**H19/H43**	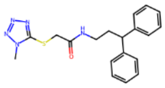	CN1C(=NN=N1)SCC(=O)NCCC(C2=CC=CC=C2)C3=CC=CC=C3	30 (36)
**H20/H44**	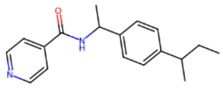	CCC(C)C1=CC=C(C=C1)C(C)NC(=O)C2=CC=NC=C2	24 (12)

**H21/H45**	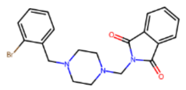	C1CN(CCN1CC2=CC=CC=C2Br)CN3C(=O)C4=CC=CC=C4C3=O	92 (90)
**H22/H46**	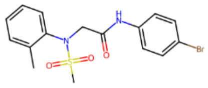	CC1=CC=CC=C1N(CC(=O)NC2=CC=C(C=C2)Br)S(=O)(=O)C	13 (10)
**H23/H47**	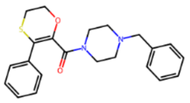	C1CN(CCN1CC2=CC=CC=C2)C(=O)C3=C(SCCO3)C4=CC=CC=C4	26 (10)
**H24/H48**	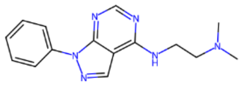	CN(C)CCNC1=C2C=NN(C2=NC=N1)C3=CC=CC=C3	93 (97)
**I1/I25**	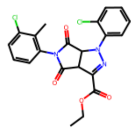	CCOC(=O)C1=NN(C2C1C(=O)N(C2=O)C3=C(C(=CC=C3)Cl)C)C4=CC=CC=C4Cl	91 (95)
**I2/I26**	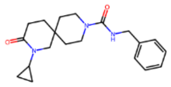	C1CC1N2CC3(CCC2=O)CCN(CC3)C(=O)NCC4=CC=CC=C4	20 (50)
**I3/I27**	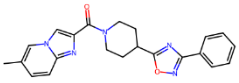	CC1=CN2C=C(N=C2C=C1)C(=O)N3CCC(CC3)C4=NC(=NO4)C5=CC=CC=C5	13 (43)
**I4/I28**	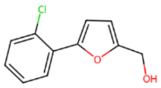	C1=CC=C(C(=C1)C2=CC=C(O2)CO)Cl	91 (98)
**I5/I29**	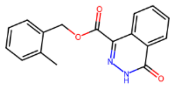	CC1=CC=CC=C1COC(=O)C2=NNC(=O)C3=CC=CC=C32	23 (34)
**I6/I30**	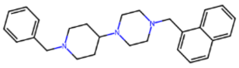	C1CN(CCC1N2CCN(CC2)CC3=CC=CC4=CC=CC=C43)CC5=CC=CC=C5	88 (97)
**I7/I31**	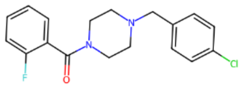	C1CN(CCN1CC2=CC=C(C=C2)Cl)C(=O)C3=CC=CC=C3F	0 (28)

**I8/I32**	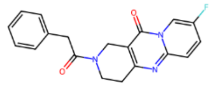	C1CN(CC2=C1N=C3C=CC(=CN3C2=O)F)C(=O)CC4=CC=CC=C4	0 (7)
**I9/I33**	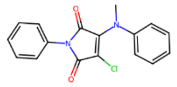	CN(C1=CC=CC=C1)C2=C(C(=O)N(C2=O)C3=CC=CC=C3)Cl	29 (52)
**I10/I34**	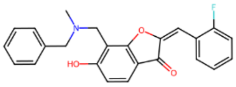	CN(CC1=CC=CC=C1)CC2=C(C=CC3=C2OC(=CC4=CC=CC=C4F)C3=O)O	17 (66)
**I11/I35**	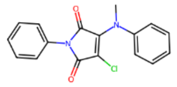	CN(C1=CC=CC=C1)C2=C(C(=O)N(C2=O)C3=CC=CC=C3)Cl	19 (14)
**I12/I36**	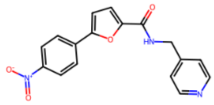	C1=CC(=CC=C1C2=CC=C(O2)C(=O)NCC3=CC=NC=C3)[N+](=O)[O-]	23 (38)
**I13/I37**	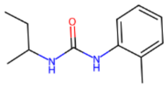	CCC(NC(NC1=CC=CC=C1C)=O)C	50 (51)
**I14/I38**	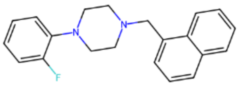	C1CN(CCN1CC2=CC=CC3=CC=CC=C32)C4=CC=CC=C4F	15 (30)
**I15/I39**	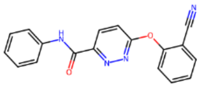	C1=CC=C(C=C1)NC(=O)C2=NN=C(C=C2)OC3=CC=CC=C3C#N	24 (6)
**I16/I40**	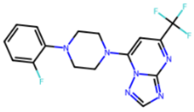	C1CN(CCN1C2=CC=CC=C2F)C3=CC(=NC4=NC=NN34)C(F)(F)F	17 (19)
**I17/I41**	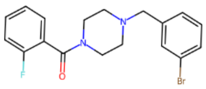	C1CN(CCN1CC2=CC(=CC=C2)Br)C(=O)C3=CC=CC=C3F	0 (25)
**I18/I42**	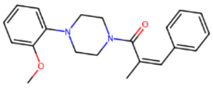	CC(=CC1=CC=CC=C1)C(=O)N2CCN(CC2)C3=CC=CC=C3OC	13 (52)
**I19/I43**	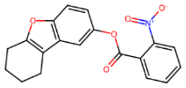	C1CCC2=C(C1)C3=C(O2)C=CC(=C3)OC(=O)C4=CC=CC=C4[N+](=O)[O-]	0 (29)

**I20/I44**	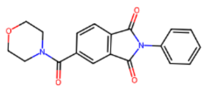	C1COCCN1C(=O)C2=CC3=C(C=C2)C(=O)N(C3=O)C4=CC=CC=C4	0 (0)
**I21/I45**	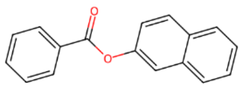	C1=CC=C(C=C1)C(=O)OC2=CC3=CC=CC=C3C=C2	18 (0)
**I22/I46**	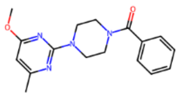	CC1=CC(=NC(=N1)N2CCN(CC2)C(=O)C3=CC=CC=C3)OC	13 (64)
**I23/I47**	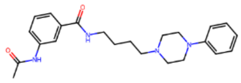	CC(=O)NC1=CC=CC(=C1)C(=O)NCCCCN2CCN(CC2)C3=CC=CC=C3	29 (50)
**I24/I48**	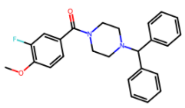	COC1=C(C=C(C=C1)C(=O)N2CCN(CC2)C(C3=CC=CC=C3)C4=CC=CC=C4)F	22 (28)
**J1/J25**	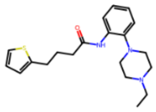	CCN1CCN(CC1)C2=CC=CC=C2NC(=O)CCCC3=CC=CS3	93 (94)
**J2/J26**	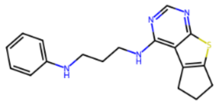	C1(NCCCNC2=C3C4=C(SC3=NC=N2)CCC4)=CC=CC=C1	26 (7)
**J3/J27**	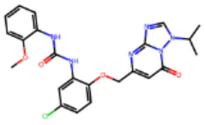	CC(C)N1C=NC2=NC(=CC(=O)N21)COC3=C(C=C(C=C3)Cl)NC(=O)NC4=CC=CC=C4OC	12 (26)
**J4/J28**	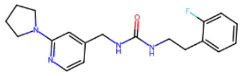	C1CCN(C1)C2=NC=CC(=C2)CNC(=O)NCCC3=CC=CC=C3F	88 (93)
**J5/J29**	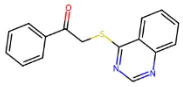	C1=CC=C(C=C1)C(=O)CSC2=NC=NC3=CC=CC=C32	11 (3)
**J6/J30**	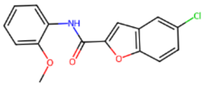	COC1=CC=CC=C1NC(=O)C2=CC3=C(O2)C=CC(=C3)Cl	11 (51)

**J7/J31**	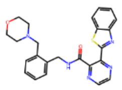	C1COCCN1CC2=CC=CC=C2CNC(=O)C3=NC=CN=C3C4=NC5=CC=CC=C5S4	31 (30)
**J8/J32**	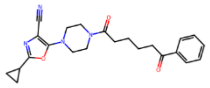	C1CC1C2=NC(=C(O2)N3CCN(CC3)C(=O)CCCCC(=O)C4=CC=CC=C4)C#N	25 (24)
**J9/J33**	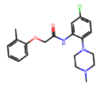	CC1=CC=CC=C1OCC(=O)NC2=C(C=CC(=C2)Cl)N3CCN(CC3)C	64 (65)
**J10/J34**	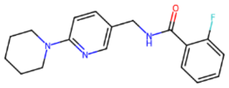	C1CCN(CC1)C2=NC=C(C=C2)CNC(=O)C3=CC=CC=C3F	43 (87)
**J11/J35**	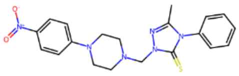	CC1=NN(C(=S)N1C2=CC=CC=C2)CN3CCN(CC3)C4=CC=C(C=C4)[N+](=O)[O-]	6 (10)
**J12/J36**	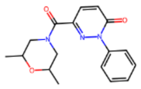	CC1CN(CC(O1)C)C(=O)C2=NN(C(=O)C=C2)C3=CC=CC=C3	96 (90)
**J13/J37**	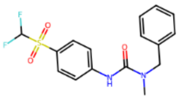	CN(CC1=CC=CC=C1)C(=O)NC2=CC=C(C=C2)S(=O)(=O)C(F)F	26 (11)
**J14/J38**	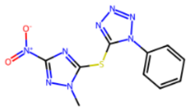	CN1C(SC2=NN=NN2C3=CC=CC=C3)=NC([N+]([O-])=O)=N1	63 (94)
**J15/J39**	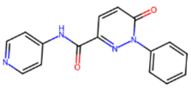	C1=CC=C(C=C1)N2C(=O)C=CC(=N2)C(=O)NC3=CC=NC=C3	26 (43)
**J16/J40**	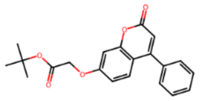	CC(C)(C)OC(=O)COC1=CC2=C(C=C1)C(=CC(=O)O2)C3=CC=CC=C3	20 (26)
**J17/J41**	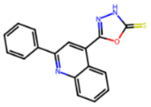	C1=CC=C(C=C1)C2=NC3=CC=CC=C3C(=C2)C4=NNC(=S)O4	91 (90)

**J18/J42**	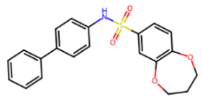	C1COC2=C(C=C(C=C2)S(=O)(=O)NC3=CC=C(C=C3)C4=CC=CC=C4)OC1	21 (28)
**J19/J43**	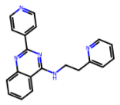	C1=CC=C2C(=C1)C(=NC(=N2)C3=CC=NC=C3)NCCC4=CC=CC=N4	14 (28)
**J20/J44**	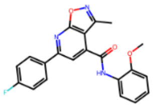	CC1=NOC2=C1C(=CC(=N2)C3=CC=C(C=C3)F)C(=O)NC4=CC=CC=C4OC	30 (27)
**J21/J45**	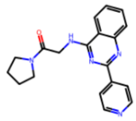	C1CCN(C1)C(=O)CNC2=NC(=NC3=CC=CC=C32)C4=CC=NC=C4	88 (89)
**J22/J46**	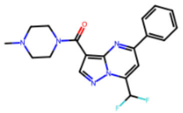	CN1CCN(CC1)C(=O)C2=C3N=C(C=C(N3N=C2)C(F)F)C4=CC=CC=C4	19 (44)
**J23/J47**	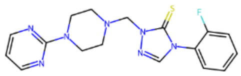	C1CN(CCN1CN2C(=S)N(C=N2)C3=CC=CC=C3F)C4=NC=CC=N4	23 (18)
**J24/J48**	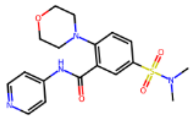	CN(C)S(=O)(=O)C1=CC(=C(C=C1)N2CCOCC2)C(=O)NC3=CC=NC=C3	0 (19)
**K1/K25**	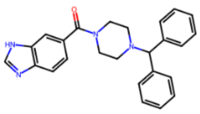	C1CN(CCN1C(C2=CC=CC=C2)C3=CC=CC=C3)C(=O)C4=CC5=C(C=C4)N=CN5	12 (11)
**K2/K26**	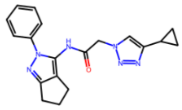	C1CC2=C(N(N=C2C1)C3=CC=CC=C3)NC(=O)CN4C=C(N=N4)C5CC5	26 (47)
**K3/K27**	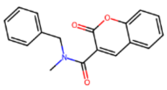	CN(CC1=CC=CC=C1)C(=O)C2=CC3=CC=CC=C3OC2=O	22 (0)
**K4/K28**	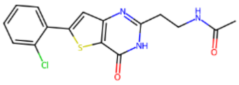	CC(=O)NCCC1=NC2=C(C(=O)N1)SC(=C2)C3=CC=CC=C3Cl	91 (98)
**K5/K29**	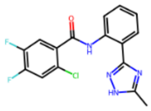	CC1=NC(=NN1)C2=CC=CC=C2NC(=O)C3=CC(=C(C=C3Cl)F)F	96 (89)

**K6/K30**	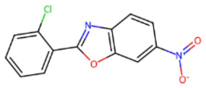	C1=CC=C(C(=C1)C2=NC3=C(O2)C=C(C=C3)[N+](=O)[O-])Cl	14 (26)
**K7/K31**	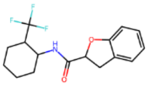	C1CCC(C(C1)C(F)(F)F)NC(=O)C2CC3=CC=CC=C3O2	92 (92)
**K8/K32**	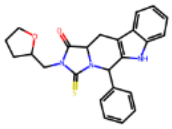	C1CC(OC1)CN2C(=O)C3CC4=C(C(N3C2=S)C5=CC=CC=C5)NC6=CC=CC=C46	88 (95)
**K9/K33**	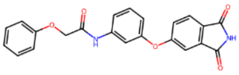	C1=CC=C(C=C1)OCC(=O)NC2=CC(=CC=C2)OC3=CC4=C(C=C3)C(=O)NC4=O	0 (0)
**K10/K34**	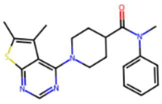	CC1=C(SC2=NC=NC(=C12)N3CCC(CC3)C(=O)N(C)C4=CC=CC=C4)C	89 (93)
**K11/K35**	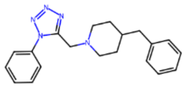	C1CN(CCC1CC2=CC=CC=C2)CC3=NN=NN3C4=CC=CC=C4	30 (92)
**K12/K36**	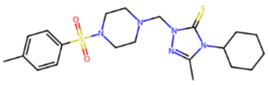	CC1=CC=C(C=C1)S(=O)(=O)N2CCN(CC2)CN3C(=S)N(C(=N3)C)C4CCCCC4	24 (48)
**K13/K37**	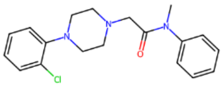	CN(C1=CC=CC=C1)C(=O)CN2CCN(CC2)C3=CC=CC=C3Cl	11 (22)
**K14/K38**	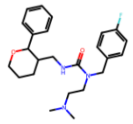	CN(C)CCN(CC1=CC=C(C=C1)F)C(=O)NCC2CCCOC2C3=CC=CC=C3	9 (13)
**K15/K39**	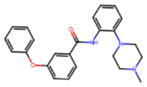	CN1CCN(CC1)C2=CC=CC=C2NC(=O)C3=CC(=CC=C3)OC4=CC=CC=C4	15 (44)
**K16/K40**	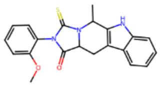	CC1C2=C(CC3N1C(=S)N(C3=O)C4=CC=CC=C4OC)C5=CC=CC=C5N2	29 (7)

**K17/K41**	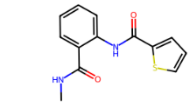	CNC(=O)C1=CC=CC=C1NC(=O)C2=CC=CS2	34 (55)
**K18/K42**	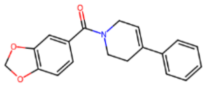	C1CN(CC=C1C2=CC=CC=C2)C(=O)C3=CC4=C(C=C3)OCO4	9 (48)
**K19/K43**	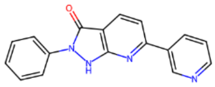	C1=CC=C(C=C1)N2C(=O)C3=C(N2)N=C(C=C3)C4=CN=CC=C4	7 (18)
**K20/K44**	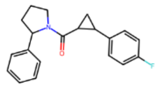	C1CC(N(C1)C(=O)C2CC2C3=CC=C(C=C3)F)C4=CC=CC=C4	21 (18)
**K21/K45**	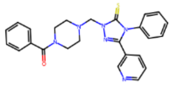	C1CN(CCN1CN2C(=S)N(C(=N2)C3=CN=CC=C3)C4=CC=CC=C4)C(=O)C5=CC=CC=C5	34 (67)
**K22/K46**	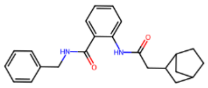	C1CC2CC1CC2CC(=O)NC3=CC=CC=C3C(=O)NCC4=CC=CC=C4	18 (59)
**K23/K47**	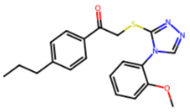	CCCC1=CC=C(C=C1)C(=O)CSC2=NN=CN2C3=CC=CC=C3OC	12 (29)
**K24/K48**	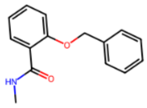	CNC(=O)C1=CC=CC=C1OCC2=CC=CC=C2	94 (94)
**L1/L25**	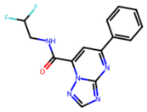	C1=CC=C(C=C1)C2=NC3=NC=NN3C(=C2)C(=O)NCC(F)F	23 (14)
**L2/L26**	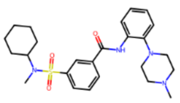	CN1CCN(CC1)C2=CC=CC=C2NC(=O)C3=CC(=CC=C3)S(=O)(=O)N(C)C4CCCCC4	34 (43)
**L3/L27**	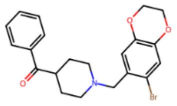	C1CN(CCC1C(=O)C2=CC=CC=C2)CC3=CC4=C(C=C3Br)OCCO4	0 (6)

**L4/L28**	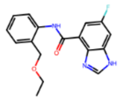	CCOCC1=CC=CC=C1NC(=O)C2=C3C(=CC(=C2)F)NC=N3	25 (59)
**L5/L29**	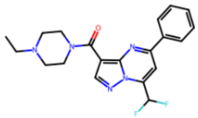	CCN1CCN(CC1)C(=O)C2=C3N=C(C=C(N3N=C2)C(F)F)C4=CC=CC=C4	17 (19)
**L6/L30**	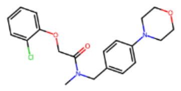	CN(CC1=CC=C(C=C1)N2CCOCC2)C(=O)COC3=CC=CC=C3Cl	23 (22)
**L7/L31**	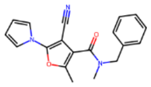	CC1=C(C(=C(O1)N2C=CC=C2)C#N)C(=O)N(C)CC3=CC=CC=C3	92 (94)
**L8/L32**	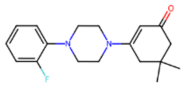	CC1(CC(=CC(=O)C1)N2CCN(CC2)C3=CC=CC=C3F)C	95 (93)
**L9/L33**	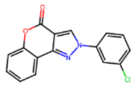	C1=CC=C2C(=C1)C3=NN(C=C3C(=O)O2)C4=CC(=CC=C4)Cl	80 (98)
**L10/L34**	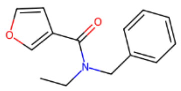	CCN(CC1=CC=CC=C1)C(=O)C2=COC=C2	94 (97)
**L11/L35**	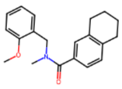	CN(CC1=CC=CC=C1OC)C(=O)C2=CC3=C(CCCC3)C=C2	92 (90)
**L12/L36**	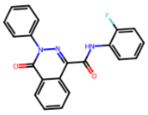	C1=CC=C(C=C1)N2C(=O)C3=CC=CC=C3C(=N2)C(=O)NC4=CC=CC=C4F	20 (18)
**L13/L37**	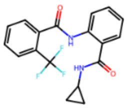	C1CC1NC(=O)C2=CC=CC=C2NC(=O)C3=CC=CC=C3C(F)(F)F	25 (30)
**L14/L38**	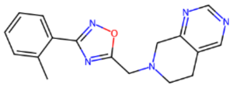	CC1=CC=CC=C1C2=NOC(=N2)CN3CCC4=CN=CN=C4C3	91 (96)
**L15/L39**	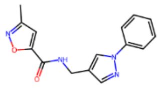	CC1=NOC(=C1)C(=O)NCC2=CN(N=C2)C3=CC=CC=C3	54 (93)

**L16/L40**	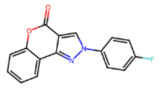	C1=CC=C2C(=C1)C3=NN(C=C3C(=O)O2)C4=CC=C(C=C4)F	36 (36)
**L17/L41**	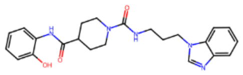	C1CN(CCC1C(=O)NC2=CC=CC=C2O)C(=O)NCCCN3C=NC4=CC=CC=C43	8 (27)
**L18/L42**	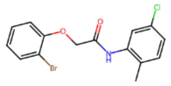	CC1=C(C=C(C=C1)Cl)NC(=O)COC2=CC=CC=C2Br	24 (45)
**L19/L43**	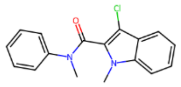	CN1C2=CC=CC=C2C(=C1C(=O)N(C)C3=CC=CC=C3)Cl	93 (89)
**L20/L44**	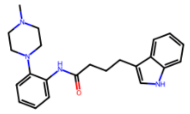	CN1CCN(CC1)C2=CC=CC=C2NC(=O)CCCC3=CNC4=CC=CC=C43	8 (20)
**L21/L45**	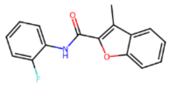	CC1=C(OC2=CC=CC=C12)C(=O)NC3=CC=CC=C3F	96 (97)
**L22/L46**	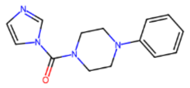	C1CN(CCN1C2=CC=CC=C2)C(=O)N3C=CN=C3	23 (47)
**L23/L47**	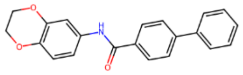	C1COC2=C(O1)C=CC(=C2)NC(=O)C3=CC=C(C=C3)C4=CC=CC=C4	29 (42)
**L24/L48**	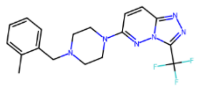	CC1=CC=CC=C1CN2CCN(CC2)C3=NN4C(=NN=C4C(F)(F)F)C=C3	14 (17)
**M1/M25**	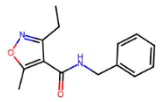	CCC1=NOC(C)=C1C(NCC2=CC=CC=C2)=O	22 (56)
**M2/M26**	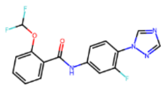	C1=CC=C(C(=C1)C(=O)NC2=CC(=C(C=C2)N3C=NC=N3)F)OC(F)F	33 (75)
**M3/M27**	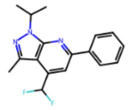	CC1=NN(C2=C1C(=CC(=N2)C3=CC=CC=C3)C(F)F)C(C)C	89 (96)

**M4/M28**	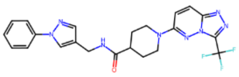	C1CN(CCC1C(=O)NCC2=CN(N=C2)C3=CC=CC=C3)C4=NN5C(=NN=C5C(F)(F)F)C=C4	11 (0)
**M5/M29**	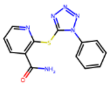	C1=CC=C(C=C1)N2C(=NN=N2)SC3=C(C=CC=N3)C(=O)N	90 (68)
**M6/M30**	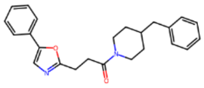	C1CN(CCC1CC2=CC=CC=C2)C(=O)CCC3=NC=C(O3)C4=CC=CC=C4	73 (92)
**M7/M31**	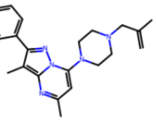	CC1=NC2=C(C(=NN2C(=C1)N3CCN(CC3)CC(=C)C)C4=CC=CC=C4)C	28 (25)
**M8/M32**	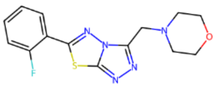	C1COCCN1CC2=NN=C3N2N=C(S3)C4=CC=CC=C4F	18 (28)
**M9/M33**	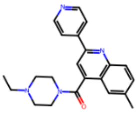	CCN1CCN(CC1)C(=O)C2=CC(=NC3=C2C=C(C=C3)C)C4=CC=NC=C4	12 (28)
**M10/M34**	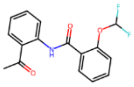	CC(=O)C1=CC=CC=C1NC(=O)C2=CC=CC=C2OC(F)F	23 (41)
**M11/M35**	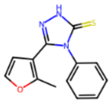	CC1=C(C2=NNC(N2C3=CC=CC=C3)=S)C=CO1	53 (98)
**M12/M36**	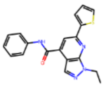	CCN1C2=C(C=N1)C(=CC(=N2)C3=CC=CS3)C(=O)NC4=CC=CC=C4	30 (13)
**M13/M37**	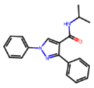	CC(C)NC(=O)C1=CN(N=C1C2=CC=CC=C2)C3=CC=CC=C3	28 (18)
**M14/M38**	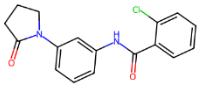	C1CC(=O)N(C1)C2=CC=CC(=C2)NC(=O)C3=CC=CC=C3Cl	62 (61)
**M15/M39**	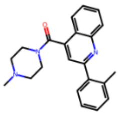	CC1=CC=CC=C1C2=NC3=CC=CC=C3C(=C2)C(=O)N4CCN(CC4)C	18 (66)

**M16/M40**	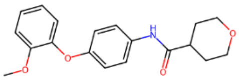	COC1=CC=CC=C1OC2=CC=C(C=C2)NC(=O)C3CCOCC3	25 (0)
**M17/M41**	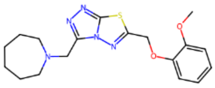	COC1=CC=CC=C1OCC2=NN3C(=NN=C3S2)CN4CCCCCC4	49 (98)
**M18/M42**	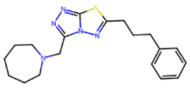	C1CCCN(CC1)CC2=NN=C3N2N=C(S3)CCCC4=CC=CC=C4	95 (91)
**M19/M43**	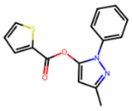	CC1=NN(C(=C1)OC(=O)C2=CC=CS2)C3=CC=CC=C3	27 (57)
**M20/M44**	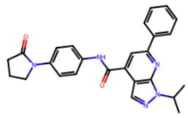	CC(C)N1C2=C(C=N1)C(=CC(=N2)C3=CC=CC=C3)C(=O)NC4=CC=C(C=C4)N5CCCC5=O	23 (28)
**M21/M45**	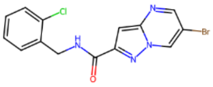	C1=CC=C(C(=C1)CNC(=O)C2=NN3C=C(C=NC3=C2)Br)Cl	88 (89)
**M22/M46**	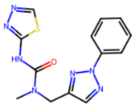	CN(CC1=NN(N=C1)C2=CC=CC=C2)C(=O)NC3=NN=CS3	24 (52)
**M23/M47**	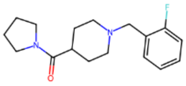	C1CCN(C1)C(=O)C2CCN(CC2)CC3=CC=CC=C3F	96 (98)
**M24/M48**	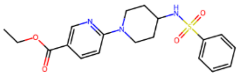	CCOC(=O)C1=CN=C(C=C1)N2CCC(CC2)NS(=O)(=O)C3=CC=CC=C3	15 (46)
**N1/N25**	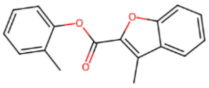	CC1=CC=CC=C1OC(=O)C2=C(C3=CC=CC=C3O2)C	23 (28)
**N2/N26**	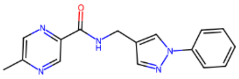	CC1=CN=C(C=N1)C(=O)NCC2=CN(N=C2)C3=CC=CC=C3	46 (50)
**N3/N27**	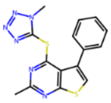	CC1=NC2=C(C(=CS2)C3=CC=CC=C3)C(=N1)SC4=NN=NN4C	24 (25)

**N4/N28**	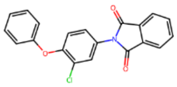	C1=CC=C(C=C1)OC2=C(C=C(C=C2)N3C(=O)C4=CC=CC=C4C3=O)Cl	33 (23)
**N5/N29**	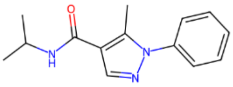	CC1=C(C=NN1C2=CC=CC=C2)C(=O)NC(C)C	25 (19)
**N6/N30**	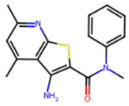	CC1=CC(=NC2=C1C(=C(S2)C(=O)N(C)C3=CC=CC=C3)N)C	22 (0)
**N7/N31**	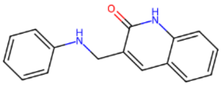	C1=CC=C(C=C1)NCC2=CC3=CC=CC=C3NC2=O	25 (5)
**N8/N32**	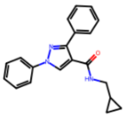	C1CC1CNC(=O)C2=CN(N=C2C3=CC=CC=C3)C4=CC=CC=C4	27 (10)
**N9/N33**	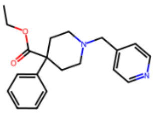	CCOC(=O)C1(CCN(CC1)CC2=CC=NC=C2)C3=CC=CC=C3	41 (50)
**N10/N34**	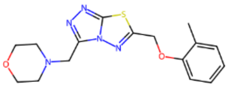	CC1=CC=CC=C1OCC2=NN3C(=NN=C3S2)CN4CCOCC4	0 (11)
**N11/N35**	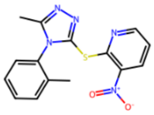	CC1=CC=CC=C1N2C(=NN=C2SC3=C(C=CC=N3)[N+](=O)[O-])C	93 (95)
**N12/N36**	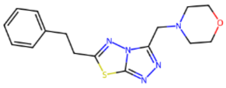	C1COCCN1CC2=NN=C3N2N=C(S3)CCC4=CC=CC=C4	21 (26)
**N13/N37**	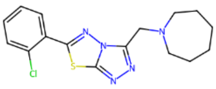	C1CCCN(CC1)CC2=NN=C3N2N=C(S3)C4=CC=CC=C4Cl	49 (90)
**N14/N38**	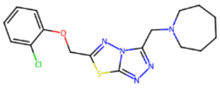	C1CCCN(CC1)CC2=NN=C3N2N=C(S3)COC4=CC=CC=C4Cl	22 (54)
**N15/N39**	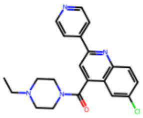	CCN1CCN(CC1)C(=O)C2=CC(=NC3=C2C=C(C=C3)Cl)C4=CC=NC=C4	5 (9)

**N16/N40**	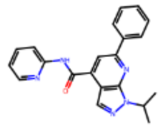	CC(C)N1C2=C(C=N1)C(=CC(=N2)C3=CC=CC=C3)C(=O)NC4=CC=CC=N4	51 (57)
**N17/N41**	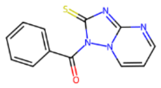	C1=CC=C(C=C1)C(=O)N2C(=S)N=C3N2C=CC=N3	26 (13)
**N18/N42**	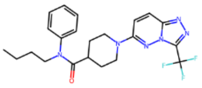	CCCCN(C1=CC=CC=C1)C(=O)C2CCN(CC2)C3=NN4C(=NN=C4C(F)(F)F)C=C3	25 (26)
**N19/N43**	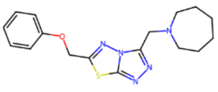	C1CCCN(CC1)CC2=NN=C3N2N=C(S3)COC4=CC=CC=C4	15 (42)
**N20/N44**	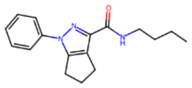	CCCCNC(=O)C1=NN(C2=C1CCC2)C3=CC=CC=C3	12 (23)
**N21/N45**	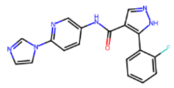	C1=CC=C(C(=C1)C2=C(C=NN2)C(=O)NC3=CN=C(C=C3)N4C=CN=C4)F	23 (30)
**N22/N46**	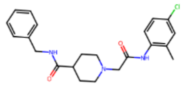	CC1=C(C=CC(=C1)Cl)NC(=O)CN2CCC(CC2)C(=O)NCC3=CC=CC=C3	11 (44)
**N23/N47**	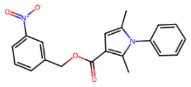	CC1=CC(=C(N1C2=CC=CC=C2)C)C(=O)OCC3=CC(=CC=C3)[N+](=O)[O-]	89 (90)
**N24/N48**	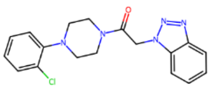	C1CN(CCN1C2=CC=CC=C2Cl)C(=O)CN3C4=CC=CC=C4N=N3	28 (56)
**O1/O25**	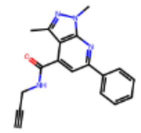	CC1=NN(C2=C1C(=CC(=N2)C3=CC=CC=C3)C(=O)NCC#C)C	46 (92)
**O2/O26**	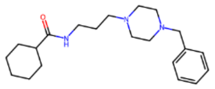	C1CCC(CC1)C(=O)NCCCN2CCN(CC2)CC3=CC=CC=C3	29 (7)
**O3/O27**	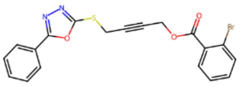	C1=CC=C(C=C1)C2=NN=C(O2)SCC#CCOC(=O)C3=CC=CC=C3Br	14 (0)

**O4/O28**	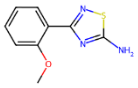	COC1=CC=CC=C1C2=NSC(=N2)N	73 (97)
**O5/O29**	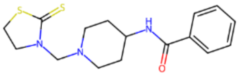	C1CN(CCC1NC(=O)C2=CC=CC=C2)CN3CCSC3=S	92 (94)
**O6/O30**	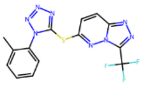	CC1=CC=CC=C1N2C(=NN=N2)SC3=NN4C(=NN=C4C(F)(F)F)C=C3	23 (35)
**O7/O31**	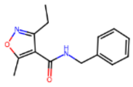	CCC1=NOC(=C1C(=O)NCC2=CC=CC=C2)C	10 (47)
**O8/O32**	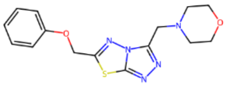	C1COCCN1CC2=NN=C3N2N=C(S3)COC4=CC=CC=C4	82 (79)
**O9/O33**	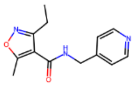	CCC1=NOC(=C1C(=O)NCC2=CC=NC=C2)C	0 (26)
**O10/O34**	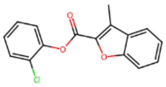	CC1=C(OC2=CC=CC=C12)C(=O)OC3=CC=CC=C3Cl	9 (19)
**O11/O35**	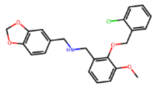	COC1=CC=CC(=C1OCC2=CC=CC=C2Cl)CNCC3=CC4=C(C=C3)OCO4	0 (17)
**O12/O36**	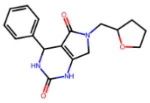	C1CC(OC1)CN2CC3=C(C2=O)C(NC(=O)N3)C4=CC=CC=C4	0 (26)
**O13/O37**	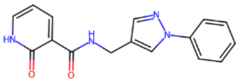	C1=CC=C(C=C1)N2C=C(C=N2)CNC(=O)C3=CC=CNC3=O	72 (37)
**O14/O38**	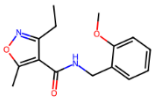	CCC1=NOC(=C1C(=O)NCC2=CC=CC=C2OC)C	16 (30)
**O15/O39**	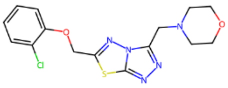	C1COCCN1CC2=NN=C3N2N=C(S3)COC4=CC=CC=C4Cl	23 (7)

**O16/O40**	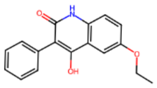	CCOC1=CC2=C(C=C1)NC(=O)C(=C2O)C3=CC=CC=C3	16 (21)
**O17/O41**	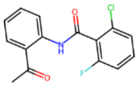	CC(=O)C1=CC=CC=C1NC(=O)C2=C(C=CC=C2Cl)F	88 (94)
**O18/O42**	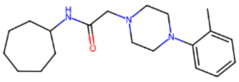	CC1=CC=CC=C1N2CCN(CC2)CC(=O)NC3CCCCCC3	89 (92)
**O19/O43**	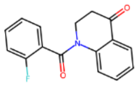	C1CN(C2=CC=CC=C2C1=O)C(=O)C3=CC=CC=C3F	17 (42)
**O20/O44**	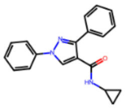	C1CC1NC(=O)C2=CN(N=C2C3=CC=CC=C3)C4=CC=CC=C4	23 (52)
**O21/O45**	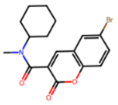	CN(C1CCCCC1)C(=O)C2=CC3=C(C=CC(=C3)Br)OC2=O	21 (47)
**O22/O46**	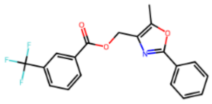	CC1=C(N=C(O1)C2=CC=CC=C2)COC(=O)C3=CC(=CC=C3)C(F)(F)F	97 (92)
**O23/O47**	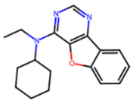	CCN(C1CCCCC1)C2=NC=NC3=C2OC4=CC=CC=C43	0 (25)
**O24/O48**	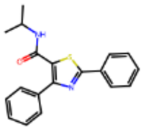	CC(C)NC(=O)C1=C(N=C(S1)C2=CC=CC=C2)C3=CC=CC=C3	0 (51)
**P1/P25**	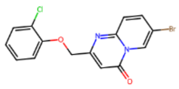	C1=CC=C(C(=C1)OCC2=CC(=O)N3C=C(C=CC3=N2)Br)Cl	66 (72)
**P2/P26**	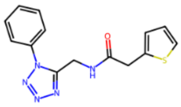	O=C(CC1=CC=CS1)NCC2=NN=NN2C3=CC=CC=C3	97 (88)
**P3/P27**	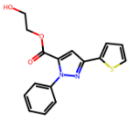	C1=CC=C(C=C1)N2C(=CC(=N2)C3=CC=CS3)C(=O)OCCO	19 (82)

**P4/P28**	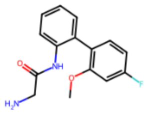	COC1=C(C=CC(=C1)F)C2=CC=CC=C2NC(=O)CN	21 (46)
**P5/P29**	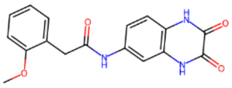	COC1=CC=CC=C1CC(=O)NC2=CC3=C(C=C2)NC(=O)C(=O)N3	0 (10)
**P6/P30**	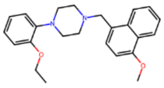	CCOC1=CC=CC=C1N2CCN(CC2)CC3=CC=C(C4=CC=CC=C34)OC	12 (14)
**P7/P31**	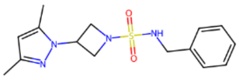	CC1=CC(=NN1C2CN(C2)S(=O)(=O)NCC3=CC=CC=C3)C	27 (57)
**P8/P32**	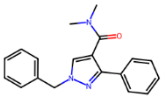	CN(C)C(=O)C1=CN(N=C1C2=CC=CC=C2)CC3=CC=CC=C3	18 (26)
**P9/P33**	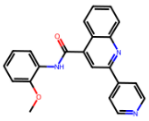	COC1=CC=CC=C1NC(=O)C2=CC(=NC3=CC=CC=C32)C4=CC=NC=C4	20 (46)
**P10/P34**	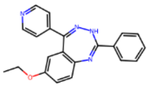	CCOC1=CC2=C(C=C1)N=C(NN=C2C3=CC=NC=C3)C4=CC=CC=C4	13 (30)
**P11/P35**	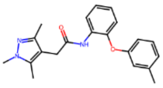	CC1=CC(=CC=C1)OC2=CC=CC=C2NC(=O)CC3=C(N(N=C3C)C)C	9 (25)
**P12/P36**	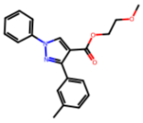	CC1=CC(=CC=C1)C2=NN(C=C2C(=O)OCCOC)C3=CC=CC=C3	11 (18)
**P13/P37**	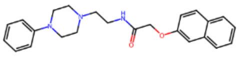	C1CN(CCN1CCNC(=O)COC2=CC3=CC=CC=C3C=C2)C4=CC=CC=C4	15 (22)
**P14/P38**	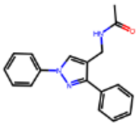	CC(=O)NCC1=CN(N=C1C2=CC=CC=C2)C3=CC=CC=C3	21 (11)
**P15/P39**	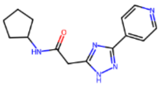	C1CCC(C1)NC(=O)CC2=NC(=NN2)C3=CC=NC=C3	8 (0)

**P16/P40**	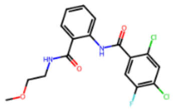	COCCNC(=O)C1=CC=CC=C1NC(=O)C2=CC(=C(C=C2Cl)Cl)F	10 (0)
**P17/P41**	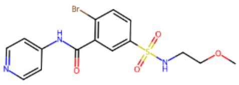	COCCNS(=O)(=O)C1=CC(=C(C=C1)Br)C(=O)NC2=CC=NC=C2	95 (91)
**P18/P42**	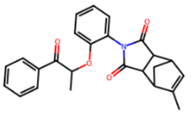	CC1=CC2CC1C3C2C(=O)N(C3=O)C4=CC=CC=C4OC(C)C(=O)C5=CC=CC=C5	22 (30)
**P19/P43**	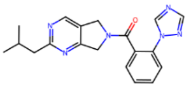	CC(C)CC1=NC=C2CN(CC2=N1)C(=O)C3=CC=CC=C3N4C=NC=N4	44 (90)
**P20/P44**	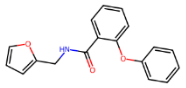	C1=CC=C(C=C1)OC2=CC=CC=C2C(=O)NCC3=CC=CO3	12 (18)
**P21/P45**	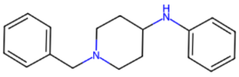	C1CN(CCC1NC2=CC=CC=C2)CC3=CC=CC=C3	0 (28)
**P22/P46**	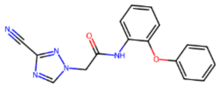	C1=CC=C(C=C1)OC2=CC=CC=C2NC(=O)CN3C=NC(=N3)C#N	26 (16)
**P23/P47**	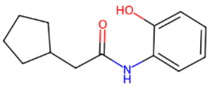	C1CCC(C1)CC(=O)NC2=CC=CC=C2O	16 (21)
**P24/P48**	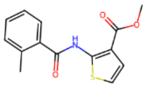	CC1=CC=CC=C1C(=O)NC2=C(C=CS2)C(=O)OC	0 (23)
**Q1/Q25**	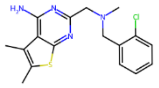	CC1=C(SC2=NC(=NC(=C12)N)CN(C)CC3=CC=CC=C3Cl)C	94 (95)
**Q2/Q26**	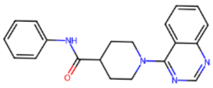	C1CN(CCC1C(=O)NC2=CC=CC=C2)C3=NC=NC4=CC=CC=C43	92 (97)
**Q3/Q27**	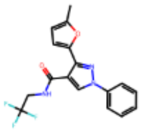	CC1=CC=C(O1)C2=NN(C=C2C(=O)NCC(F)(F)F)C3=CC=CC=C3	25 (14)

**Q4/Q28**	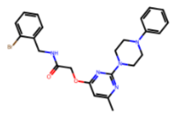	CC1=CC(=NC(=N1)N2CCN(CC2)C3=CC=CC=C3)OCC(=O)NCC4=CC=CC=C4Br	10 (22)
**Q5/Q29**	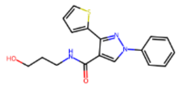	C1=CC=C(C=C1)N2C=C(C(=N2)C3=CC=CS3)C(=O)NCCCO	32 (69)
**Q6/Q30**	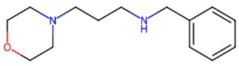	C1COCCN1CCCNCC2=CC=CC=C2	10 (23)
**Q7/Q31**	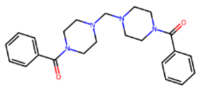	C1CN(CCN1CN2CCN(CC2)C(=O)C3=CC=CC=C3)C(=O)C4=CC=CC=C4	26 (21)
**Q8/Q32**	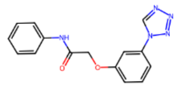	C1=CC=C(C=C1)NC(=O)COC2=CC=CC(=C2)N3C=NN=N3	37 (38)
**Q9/Q33**	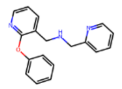	C1=CC=C(C=C1)OC2=C(C=CC=N2)CNCC3=CC=CC=N3	27 (63)
**Q10/Q34**	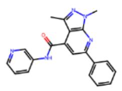	CC1=NN(C2=C1C(=CC(=N2)C3=CC=CC=C3)C(=O)NC4=CN=CC=C4)C	0 (43)
**Q11/Q35**	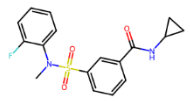	CN(C1=CC=CC=C1F)S(=O)(=O)C2=CC=CC(=C2)C(=O)NC3CC3	0 (43)
**Q12/Q36**	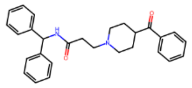	C1CN(CCC1C(=O)C2=CC=CC=C2)CCC(=O)NC(C3=CC=CC=C3)C4=CC=CC=C4	28 (43)
**Q13/Q37**	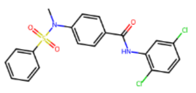	CN(C1=CC=C(C=C1)C(=O)NC2=C(C=CC(=C2)Cl)Cl)S(=O)(=O)C3=CC=CC=C3	36 (37)
**Q14/Q38**	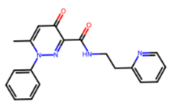	CC1=CC(=O)C(=NN1C2=CC=CC=C2)C(=O)NCCC3=CC=CC=N3	20 (27)

**Q15/Q39**	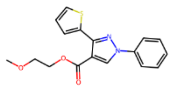	COCCOC(=O)C1=CN(N=C1C2=CC=CS2)C3=CC=CC=C3	13 (48)
**Q16/Q40**	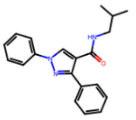	CC(C)CNC(=O)C1=CN(N=C1C2=CC=CC=C2)C3=CC=CC=C3	88 (92)
**Q17/Q41**	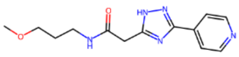	COCCCNC(=O)CC1=NC(=NN1)C2=CC=NC=C2	24 (38)
**Q18/Q42**	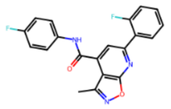	CC1=NOC2=C1C(=CC(=N2)C3=CC=CC=C3F)C(=O)NC4=CC=C(C=C4)F	25 (21)
**Q19/Q43**	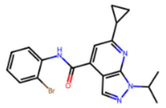	CC(C)N1C2=C(C=N1)C(=CC(=N2)C3CC3)C(=O)NC4=CC=CC=C4Br	10 (10)
**Q20/Q44**	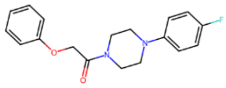	C1CN(CCN1C2=CC=C(C=C2)F)C(=O)COC3=CC=CC=C3	96 (91)
**Q21/Q45**	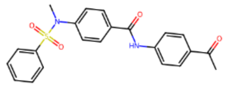	CC(=O)C1=CC=C(C=C1)NC(=O)C2=CC=C(C=C2)N(C)S(=O)(=O)C3=CC=CC=C3	33 (38)
**Q22/Q46**	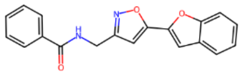	C1=CC=C(C=C1)C(=O)NCC2=NOC(=C2)C3=CC4=CC=CC=C4O3	30 (15)
**Q23/Q47**	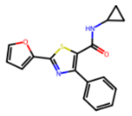	C1CC1NC(=O)C2=C(N=C(S2)C3=CC=CO3)C4=CC=CC=C4	64 (89)
**Q24/Q48**	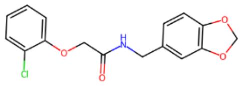	C1OC2=C(O1)C=C(C=C2)CNC(=O)COC3=CC=CC=C3Cl	7 (14)
**R1/R25**	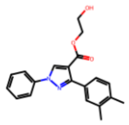	CC1=C(C=C(C=C1)C2=NN(C=C2C(=O)OCCO)C3=CC=CC=C3)C	98 (93)
**R2/R26**	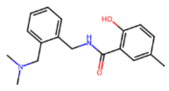	CC1=CC(=C(C=C1)O)C(=O)NCC2=CC=CC=C2CN(C)C	46 (85)

**R3/R27**	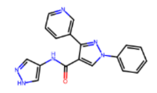	C1=CC=C(C=C1)N2C=C(C(=N2)C3=CN=CC=C3)C(=O)NC4=CNN=C4	27 (53)
**R4/R28**	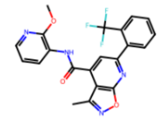	CC1=NOC2=C1C(=CC(=N2)C3=CC=CC=C3C(F)(F)F)C(=O)NC4=C(N=CC=C4)OC	29 (51)
**R5/R29**	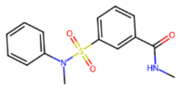	CNC(=O)C1=CC(=CC=C1)S(=O)(=O)N(C)C2=CC=CC=C2	7 (0)
**R6/R30**	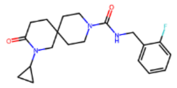	C1CC1N2CC3(CCC2=O)CCN(CC3)C(=O)NCC4=CC=CC=C4F	35 (36)
**R7/R31**	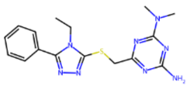	CCN1C(=NN=C1SCC2=NC(=NC(=N2)N(C)C)N)C3=CC=CC=C3	0 (28)
**R8/R32**	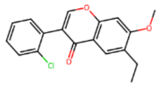	CCC1=CC2=C(C=C1OC)OC=C(C2=O)C3=CC=CC=C3Cl	16 (25)
**R9/R33**	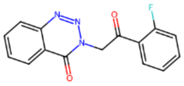	C1=CC=C2C(=C1)C(=O)N(N=N2)CC(=O)C3=CC=CC=C3F	89 (92)
**R10/R34**	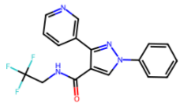	C1=CC=C(C=C1)N2C=C(C(=N2)C3=CN=CC=C3)C(=O)NCC(F)(F)F	19 (8)
**R11/R35**	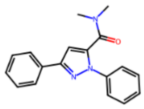	CN(C)C(=O)C1=CC(=NN1C2=CC=CC=C2)C3=CC=CC=C3	25 (43)
**R12/R36**	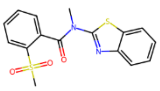	CN(C1=NC2=CC=CC=C2S1)C(=O)C3=CC=CC=C3S(=O)(=O)C	20 (48)
**R13/R37**	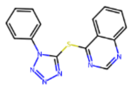	C1=CC=C(C=C1)N2C(=NN=N2)SC3=NC=NC4=CC=CC=C43	25 (51)
**R14/R38**	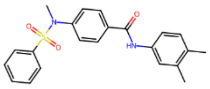	CC1=C(C=C(C=C1)NC(=O)C2=CC=C(C=C2)N(C)S(=O)(=O)C3=CC=CC=C3)C	31 (10)

		C	
**R15/R39**	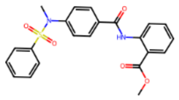	CN(C1=CC=C(C=C1)C(=O)NC2=CC=CC=C2C(=O)OC)S(=O)(=O)C3=CC=CC=C3	8 (17)
**R16/R40**	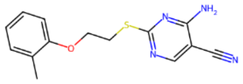	CC1=CC=CC=C1OCCSC2=NC=C(C(=N2)N)C#N	68 (90)
**R17/R41**	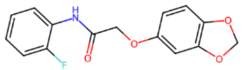	C1OC2=C(O1)C=C(C=C2)OCC(=O)NC3=CC=CC=C3F	97 (89)
**R18/R42**	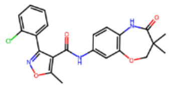	CC1=C(C(=NO1)C2=CC=CC=C2Cl)C(=O)NC3=CC4=C(C=C3)NC(=O)C(CO4)(C)C	29 (21)
**R19/R43**	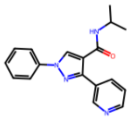	CC(C)NC(=O)C1=CN(N=C1C2=CN=CC=C2)C3=CC=CC=C3	11 (8)
**R20/R44**	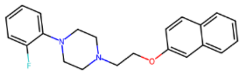	C1CN(CCN1CCOC2=CC3=CC=CC=C3C=C2)C4=CC=CC=C4F	41 (45)
**R21/R45**	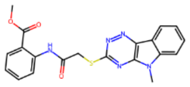	CN1C2=CC=CC=C2C3=C1N=C(N=N3)SCC(=O)NC4=CC=CC=C4C(=O)OC	30 (18)
**R22/R46**	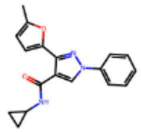	CC1=CC=C(O1)C2=NN(C=C2C(=O)NC3CC3)C4=CC=CC=C4	15 (50)
**R23/R47**	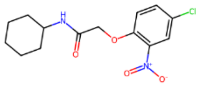	C1CCC(CC1)NC(=O)COC2=C(C=C(C=C2)Cl)[N+](=O)[O-]	32 (63)
**R24/R48**	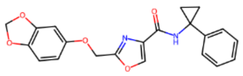	C1CC1(C2=CC=CC=C2)NC(=O)C3=COC(=N3)COC4=CC5=C(C=C4)OCO5	89 (94)

**S1/S25**	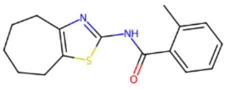	CC1=CC=CC=C1C(=O)NC2=NC3=C(S2)CCCCC3	39 (41)
**S2/S26**	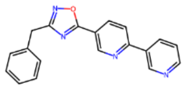	C1=CC=C(C=C1)CC2=NOC(=N2)C3=CN=C(C=C3)C4=CN=CC=C4	13 (41)
**S3/S27**	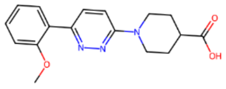	COC1=CC=CC=C1C2=NN=C(C=C2)N3CCC(CC3)C(=O)O	34 (77)
**S4/S28**	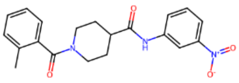	CC1=CC=CC=C1C(=O)N2CCC(CC2)C(=O)NC3=CC(=CC=C3)[N+](=O)[O-]	26 (45)
**S5/S29**	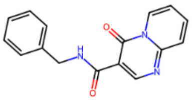	O=C(C1=CN=C2C=CC=CN2C1=O)NCC3=CC=CC=C3	0 (27)
**S6/S30**	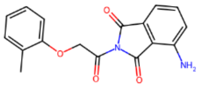	CC1=CC=CC=C1OCC(=O)N2C(=O)C3=C(C2=O)C(=CC=C3)N	22 (36)
**S7/S31**	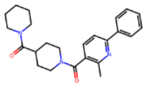	CC1=C(C=CC(=N1)C2=CC=CC=C2)C(=O)N3CCC(CC3)C(=O)N4CCCCC4	11 (27)
**S8/S32**	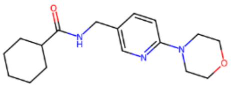	C1CCC(CC1)C(=O)NCC2=CN=C(C=C2)N3CCOCC3	21 (40)
**S9/S33**	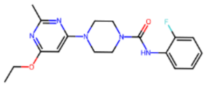	CCOC1=NC(=NC(=C1)N2CCN(CC2)C(=O)NC3=CC=CC=C3F)C	88 (47)
**S10/S34**	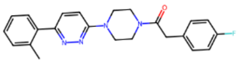	CC1=CC=CC=C1C2=NN=C(C=C2)N3CCN(CC3)C(=O)CC4=CC=C(C=C4)F	4 (29)
**S11/S35**	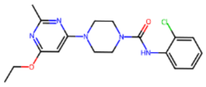	CCOC1=NC(=NC(=C1)N2CCN(CC2)C(=O)NC3=CC=CC=C3Cl)C	20 (8)
**S12/S36**	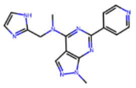	CN1C2=C(C=N1)C(=NC(=N2)C3=CC=NC=C3)N(C)CC4=NC=CN4	95 (93)

**S13/S37**	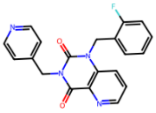	C1=CC=C(C(=C1)CN2C3=C(C(=O)N(C2=O)CC4=CC=NC=C4)N=CC=C3)F	28 (37)
**S14/S38**	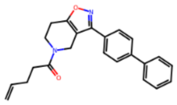	C=CCCC(=O)N1CCC2=C(C1)C(=NO2)C3=CC=C(C=C3)C4=CC=CC=C4	14 (23)
**S15/S39**	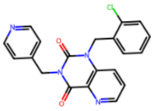	C1=CC=C(C(=C1)CN2C3=C(C(=O)N(C2=O)CC4=CC=NC=C4)N=CC=C3)Cl	51 (90)
**S16/S40**	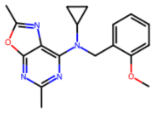	CC1=NC(=C2C(=N1)OC(=N2)C)N(CC3=CC=CC=C3OC)C4CC4	0 (28)
**S17/S41**	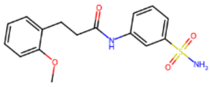	COC1=CC=CC=C1CCC(=O)NC2=CC(=CC=C2)S(=O)(=O)N	24 (27)
**S18/S42**	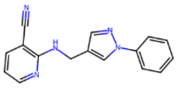	C1=CC=C(C=C1)N2C=C(C=N2)CNC3=C(C=CC=N3)C#N	25 (48)
**S19/S43**	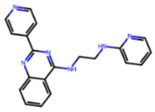	C1=CC=C2C(=C1)C(=NC(=N2)C3=CC=NC=C3)NCCNC4=CC=CC=N4	94 (96)
**S20/S44**	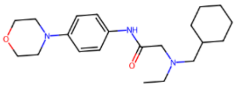	CCN(CC1CCCCC1)CC(=O)NC2=CC=C(C=C2)N3CCOCC3	21 (22)
**S21/S45**	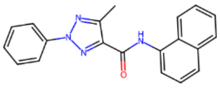	CC1=NN(N=C1C(=O)NC2=CC=CC3=CC=CC=C32)C4=CC=CC=C4	27 (25)
**S22/S46**	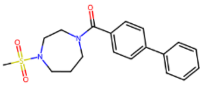	CS(=O)(=O)N1CCCN(CC1)C(=O)C2=CC=C(C=C2)C3=CC=CC=C3	0 (0)
**S23/S47**	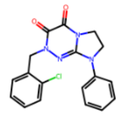	C1CN2C(=O)C(=O)N(N=C2N1C3=CC=CC=C3)CC4=CC=CC=C4Cl	28 (59)
**S24/S48**	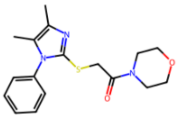	CC1=C(N(C(=N1)SCC(=O)N2CCOCC2)C3=CC=CC=C3)C	5 (28)

**T1/T25**	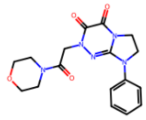	C1CN2C(=O)C(=O)N(N=C2N1C3=CC=CC=C3)CC(=O)N4CCOCC4	16 (30)
**T2/T26**	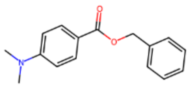	CN(C)C1=CC=C(C=C1)C(=O)OCC2=CC=CC=C2	89 (97)
**T3/T27**	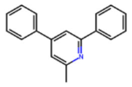	CC1=CC(=CC(=N1)C2=CC=CC=C2)C3=CC=CC=C3	91 (91)
**T4/T28**	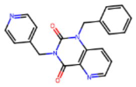	C1=CC=C(C=C1)CN2C3=C(C(=O)N(C2=O)CC4=CC=NC=C4)N=CC=C3	25 (27)
**T5/T29**	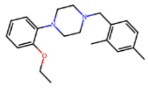	CCOC1=CC=CC=C1N2CCN(CC2)CC3=C(C=C(C=C3)C)C	12 (43)
**T6/T30**	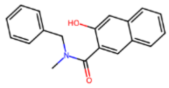	CN(CC1=CC=CC=C1)C(=O)C2=CC3=CC=CC=C3C=C2O	94 (91)
**T7/T31**	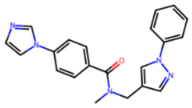	CN(CC1=CN(N=C1)C2=CC=CC=C2)C(=O)C3=CC=C(C=C3)N4C=CN=C4	39 (78)
**T8/T32**	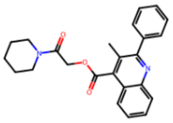	CC1=C(C2=CC=CC=C2N=C1C3=CC=CC=C3)C(=O)OCC(=O)N4CCCCC4	95 (84)
**T9/T33**	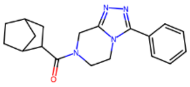	C1CC2CC1CC2C(=O)N3CCN4C(=NN=C4C5=CC=CC=C5)C3	10 (25)
**T10/T34**	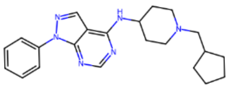	C1CCC(C1)CN2CCC(CC2)NC3=C4C=NN(C4=NC=N3)C5=CC=CC=C5	41 (43)
**T11/T35**	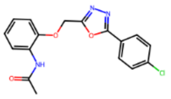	CC(=O)NC1=CC=CC=C1OCC2=NN=C(O2)C3=CC=C(C=C3)Cl	21 (53)
**T12/T36**	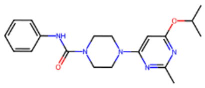	CC1=NC(=CC(=N1)OC(C)C)N2CCN(CC2)C(=O)NC3=CC=CC=C3	18 (31)

**T13/T37**	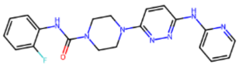	C1CN(CCN1C2=NN=C(C=C2)NC3=CC=CC=N3)C(=O)NC4=CC=CC=C4F	6 (28)
**T14/T38**	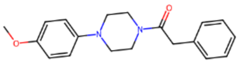	COC1=CC=C(C=C1)N2CCN(CC2)C(=O)CC3=CC=CC=C3	19 (18)
**T15/T39**	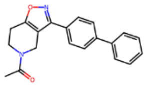	CC(=O)N1CCC2=C(C1)C(=NO2)C3=CC=C(C=C3)C4=CC=CC=C4	95 (92)
**T16/T40**	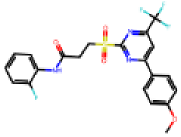	COC1=CC=C(C=C1)C2=CC(=NC(=N2)S(=O)(=O)CCC(=O)NC3=CC=CC=C3F)C(F)(F)F	90 (95)
**T17/T41**	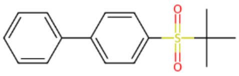	CC(C)(C)S(=O)(=O)C1=CC=C(C=C1)C2=CC=CC=C2	27 (50)
**T18/T42**	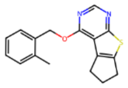	CC1=CC=CC=C1COC2=C3C4=C(CCC4)SC3=NC=N2	57 (96)
**T19/T43**	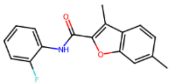	CC1=CC2=C(C=C1)C(=C(O2)C(=O)NC3=CC=CC=C3F)C	24 (43)
**T20/T44**	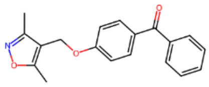	CC1=C(C(=NO1)C)COC2=CC=C(C=C2)C(=O)C3=CC=CC=C3	94 (89)
**T21/T45**	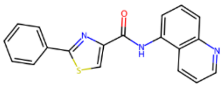	C1=CC=C(C=C1)C2=NC(=CS2)C(=O)NC3=CC=CC4=C3C=CC=N4	22 (11)
**T22/T46**	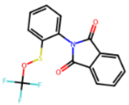	C1=CC=C2C(=C1)C(=O)N(C2=O)C3=CC=CC=C3SOC(F)(F)F	26 (60)
**T23/T47**	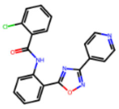	C1=CC=C(C(=C1)C2=NC(=NO2)C3=CC=NC=C3)NC(=O)C4=CC=CC=C4Cl	15 (55)
**T24/T48**	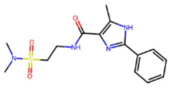	CC1=C(N=C(N1)C2=CC=CC=C2)C(=O)NCCS(=O)(=O)N(C)C	24 (25)

**U1/U25**	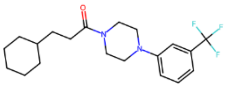	C1CCC(CC1)CCC(=O)N2CCN(CC2)C3=CC=CC(=C3)C(F)(F)F	39 (14)
**U2/U26**	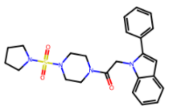	C1CCN(C1)S(=O)(=O)N2CCN(CC2)C(=O)CN3C4=CC=CC=C4C=C3C5=CC=CC=C5	41 (50)
**U3/U27**	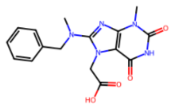	CN1C2=C(C(=O)NC1=O)N(C(=N2)N(C)CC3=CC=CC=C3)CC(=O)O	56 (94)
**U4/U28**	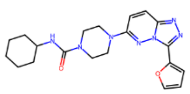	C1CCC(CC1)NC(=O)N2CCN(CC2)C3=NN4C(=NN=C4C5=CC=CO5)C=C3	39 (34)
**U5/U29**	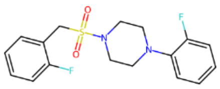	C1CN(CCN1C2=CC=CC=C2F)S(=O)(=O)CC3=CC=CC=C3F	29 (6)
**U6/U30**	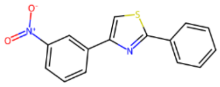	C1=CC=C(C=C1)C2=NC(=CS2)C3=CC(=CC=C3)[N+](=O)[O-]	14 (59)
**U7/U31**	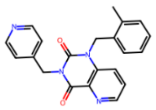	CC1=CC=CC=C1CN2C3=C(C(=O)N(C2=O)CC4=CC=NC=C4)N=CC=C3	14 (26)
**U8/U32**	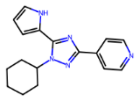	C1CCC(CC1)N2C(=NC(=N2)C3=CC=NC=C3)C4=CC=CN4	46 (47)
**U9/U33**	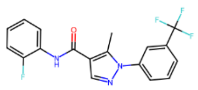	CC1=C(C=NN1C2=CC=CC(=C2)C(F)(F)F)C(=O)NC3=CC=CC=C3F	97 (97)
**U10/U34**	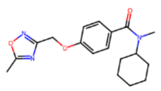	CC1=NC(=NO1)COC2=CC=C(C=C2)C(=O)N(C)C3CCCCC3	59 (57)
**U11/U35**	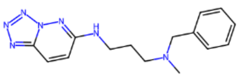	CN(CCCNC1=NN2C(=NN=N2)C=C1)CC3=CC=CC=C3	61 (58)
**U12/U36**	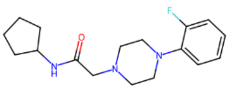	C1CCC(C1)NC(=O)CN2CCN(CC2)C3=CC=CC=C3F	18 (28)

**U13/U37**	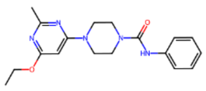	CCOC1=NC(=NC(=C1)N2CCN(CC2)C(=O)NC3=CC=CC=C3)C	95 (96)
**U14/U38**	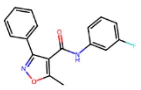	CC1=C(C(=NO1)C2=CC=CC=C2)C(=O)NC3=CC(=CC=C3)F	4 (24)
**U15/U39**	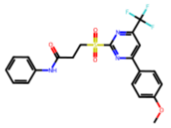	COC1=CC=C(C=C1)C2=CC(=NC(=N2)S(=O)(=O)CCC(=O)NC3=CC=CC=C3)C(F)(F)F	28 (34)
**U16/U40**	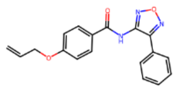	C=CCOC1=CC=C(C=C1)C(=O)NC2=NON=C2C3=CC=CC=C3	96 (91)
**U17/U41**	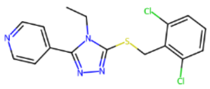	CCN1C(=NN=C1SCC2=C(C=CC=C2Cl)Cl)C3=CC=NC=C3	90 (72)
**U18/U42**	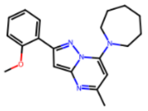	CC1=NC2=CC(=NN2C(=C1)N3CCCCCC3)C4=CC=CC=C4OC	0 (51)
**U19/U43**	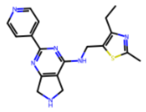	CCC1=C(SC(=N1)C)CNC2=NC(=NC3=C2CNC3)C4=CC=NC=C4	22 (22)
**U20/U44**	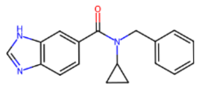	C1CC1N(CC2=CC=CC=C2)C(=O)C3=CC4=C(C=C3)N=CN4	20 (22)
**U21/U45**	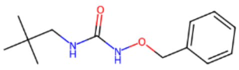	CC(C)(C)CNC(=O)NOCC1=CC=CC=C1	91 (90)
**U22/U46**	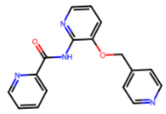	C1=CC=NC(=C1)C(=O)NC2=C(C=CC=N2)OCC3=CC=NC=C3	10 (12)
**U23/U47**	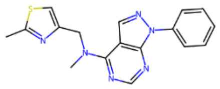	CC1=NC(=CS1)CN(C)C2=NC=NC3=C2C=NN3C4=CC=CC=C4	74 (81)
**U24/U48**	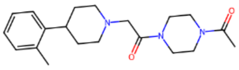	CC1=CC=CC=C1C2CCN(CC2)CC(=O)N3CCN(CC3)C(=O)C	15 (27)

**V1/V25**	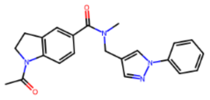	CC(=O)N1CCC2=C1C=CC(=C2)C(=O)N(C)CC3=CN(N=C3)C4=CC=CC=C4	23 (43)
**V2/V26**	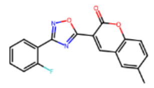	CC1=CC2=C(C=C1)OC(=O)C(=C2)C3=NC(=NO3)C4=CC=CC=C4F	13 (0)
**V3/V27**	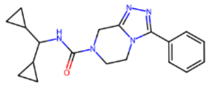	C1CC1C(C2CC2)NC(=O)N3CCN4C(=NN=C4C5=CC=CC=C5)C3	45 (52)
**V4/V28**	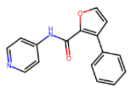	C1=CC=C(C=C1)C2=C(OC=C2)C(=O)NC3=CC=NC=C3	96 (91)
**V5/V29**	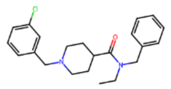	CCN(CC1=CC=CC=C1)C(=O)C2CCN(CC2)CC3=CC(=CC=C3)Cl	9 (27)
**V6/V30**	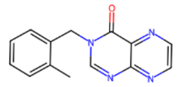	CC1=CC=CC=C1CN2C=NC3=NC=CN=C3C2=O	23 (56)
**V7/V31**	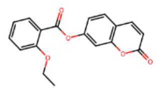	CCOC1=CC=CC=C1C(=O)OC2=CC3=C(C=C2)C=CC(=O)O3	95 (94)
**V8/V32**	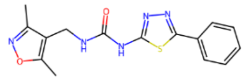	CC1=C(C(=NO1)C)CNC(=O)NC2=NN=C(S2)C3=CC=CC=C3	23 (18)
**V9/V33**	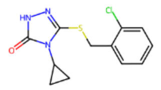	C1CC1N2C(=O)NN=C2SCC3=CC=CC=C3Cl	5 (49)
**V10/V34**	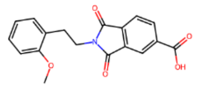	COC1=CC=CC=C1CCN2C(=O)C3=C(C2=O)C=C(C=C3)C(=O)O	10 (55)
**V11/V35**	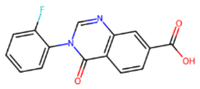	C1=CC=C(C(=C1)N2C=NC3=C(C2=O)C=CC(=C3)C(=O)O)F	0 (13)
**V12/V36**	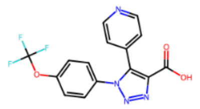	C1=CC(=CC=C1N2C(=C(N=N2)C(=O)O)C3=CC=NC=C3)OC(F)(F)F	25 (24)

**V13/V37**	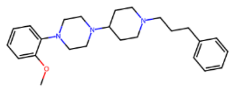	COC1=CC=CC=C1N2CCN(CC2)C3CCN(CC3)CCCC4=CC=CC=C4	31 (68)
**V14/V38**	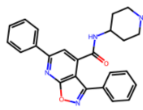	CN1CCC(CC1)NC(=O)C2=CC(=NC3=C2C(=NO3)C4=CC=CC=C4)C5=CC=CC=C5	16 (26)
**V15/V39**	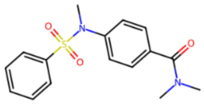	CN(C)C(=O)C1=CC=C(C=C1)N(C)S(=O)(=O)C2=CC=CC=C2	27 (46)
**V16/V40**	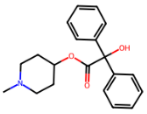	CN1CCC(CC1)OC(=O)C(C2=CC=CC=C2)(C3=CC=CC=C3)O	46 (87)
**V17/V41**	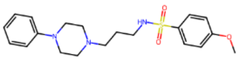	COC1=CC=C(C=C1)S(=O)(=O)NCCCN2CCN(CC2)C3=CC=CC=C3	28 (22)
**V18/V42**	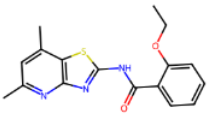	CCOC1=CC=CC=C1C(=O)NC2=NC3=C(S2)C(=CC(=N3)C)C	13 (9)
**V19/V43**	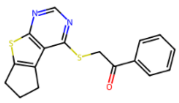	C1CC2=C(C1)SC3=C2C(=NC=N3)SCC(=O)C4=CC=CC=C4	32 (75)
**V20/V44**	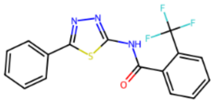	C1=CC=C(C=C1)C2=NN=C(S2)NC(=O)C3=CC=CC=C3C(F)(F)F	17 (20)
**V21/V45**	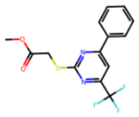	COC(=O)CSC1=NC(=CC(=N1)C(F)(F)F)C2=CC=CC=C2	26 (0)
**V22/V46**	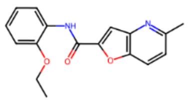	CCOC1=CC=CC=C1NC(=O)C2=CC3=C(O2)C=CC(=N3)C	5 (40)
**V23/V47**	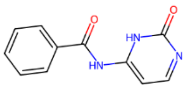	C1=CC=C(C=C1)C(=O)NC2=CC=NC(=O)N2	18 (24)
**V24/V48**	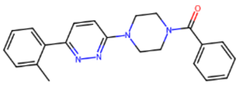	CC1=CC=CC=C1C2=NN=C(C=C2)N3CCN(CC3)C(=O)C4=CC=CC=C4	22 (11)

**W1/W25**	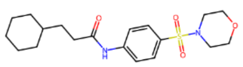	C1CCC(CC1)CCC(=O)NC2=CC=C(C=C2)S(=O)(=O)N3CCOCC3	34 (90)
**W2/W26**	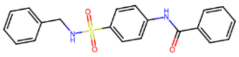	C1=CC=C(C=C1)CNS(=O)(=O)C2=CC=C(C=C2)NC(=O)C3=CC=CC=C3	14 (30)
**W3/W27**	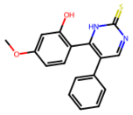	COC1=CC(=C(C=C1)C2=C(C=NC(=S)N2)C3=CC=CC=C3)O	19 (4)
**W4/W28**	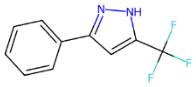	C1=CC=C(C=C1)C2=NNC(=C2)C(F)(F)F	23 (24)
**W5/W29**	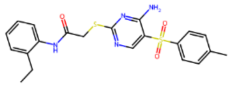	CCC1=CC=CC=C1NC(=O)CSC2=NC=C(C(=N2)N)S(=O)(=O)C3=CC=C(C=C3)C	0 (22)
**W6/W30**	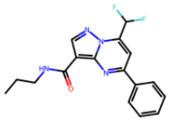	CCCNC(=O)C1=C2N=C(C=C(N2N=C1)C(F)F)C3=CC=CC=C3	11 (9)
**W7/W31**	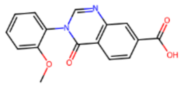	COC1=CC=CC=C1N2C=NC3=C(C2=O)C=CC(=C3)C(=O)O	28 (48)
**W8/W32**	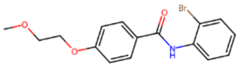	COCCOC1=CC=C(C=C1)C(=O)NC2=CC=CC=C2Br	97 (93)
**W9/W33**	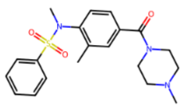	CC1=C(C=CC(=C1)C(=O)N2CCN(CC2)C)N(C)S(=O)(=O)C3=CC=CC=C3	45 (44)
**W10/W34**	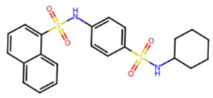	C1CCC(CC1)NS(=O)(=O)C2=CC=C(C=C2)NS(=O)(=O)C3=CC=CC4=CC=CC=C43	22 (50)
**W11/W35**	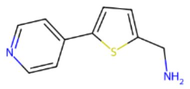	C1=CN=CC=C1C2=CC=C(S2)CN	96 (87)

**W12/W36**	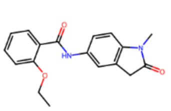	CCOC1=CC=CC=C1C(=O)NC2=CC3=C(C=C2)N(C(=O)C3)C	23 (45)
**W13/W37**	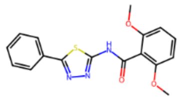	COC1=C(C(=CC=C1)OC)C(=O)NC2=NN=C(S2)C3=CC=CC=C3	12 (15)
**W14/W38**	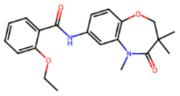	CCOC1=CC=CC=C1C(=O)NC2=CC3=C(C=C2)OCC(C(=O)N3C)(C)C	21 (17)
**W15/W39**	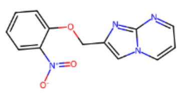	C1=CC=C(C(=C1)[N+](=O)[O-])OCC2=CN3C=CC=NC3=N2	16 (45)
**W16/W40**	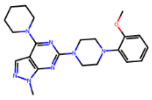	CN1C2=C(C=N1)C(=NC(=N2)N3CCN(CC3)C4=CC=CC=C4OC)N5CCCCC5	13 (15)
**W17/W41**	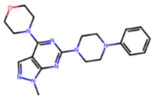	CN1C2=C(C=N1)C(=NC(=N2)N3CCN(CC3)C4=CC=CC=C4)N5CCOCC5	25 (49)
**W18/W42**	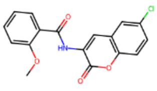	COC1=CC=CC=C1C(=O)NC2=CC3=C(C=CC(=C3)Cl)OC2=O	23 (42)
**W19/W43**	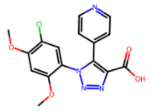	COC1=CC(=C(C=C1N2C(=C(N=N2)C(=O)O)C3=CC=NC=C3)Cl)OC	20 (18)
**W20/W44**	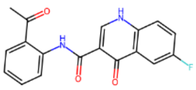	CC(=O)C1=CC=CC=C1NC(=O)C2=CNC3=C(C2=O)C=C(C=C3)F	92 (98)
**W21/W45**	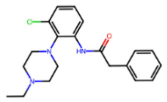	CCN1CCN(CC1)C2=C(C=CC=C2Cl)NC(=O)CC3=CC=CC=C3	25 (45)
**W22/W46**	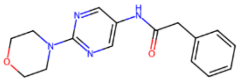	C1COCCN1C2=NC=C(C=N2)NC(=O)CC3=CC=CC=C3	92 (92)
**W23/W47**	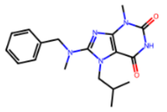	CC(C)CN1C2=C(N=C1N(C)CC3=CC=CC=C3)N(C(=O)NC2=O)C	60 (88)

**W24/W48**	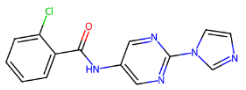	C1=CC=C(C(=C1)C(=O)NC2=CN=C(N=C2)N3C=CN=C3)Cl	5 (22)
**X1/X25**	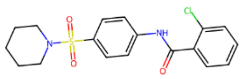	C1CCN(CC1)S(=O)(=O)C2=CC=C(C=C2)NC(=O)C3=CC=CC=C3Cl	96 (96)
**X2/X26**	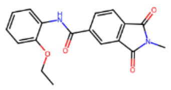	CCOC1=CC=CC=C1NC(=O)C2=CC3=C(C=C2)C(=O)N(C3=O)C	14 (21)
**X3/X27**	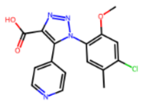	CC1=CC(=C(C=C1Cl)OC)N2C(=C(N=N2)C(=O)O)C3=CC=NC=C3	93 (94)
**X4/X28**	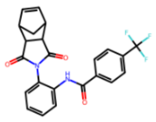	C1C2C=CC1C3C2C(=O)N(C3=O)C4=CC=CC=C4NC(=O)C5=CC=C(C=C5)C(F)(F)F	22 (44)
**X5/X29**	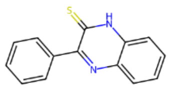	C1=CC=C(C=C1)C2=NC3=CC=CC=C3NC2=S	48 (45)
**X6/X30**	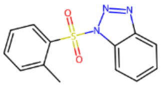	CC1=CC=CC=C1S(=O)(=O)N2C3=CC=CC=C3N=N2	62 (62)
**X7/X31**	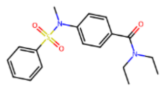	CCN(CC)C(=O)C1=CC=C(C=C1)N(C)S(=O)(=O)C2=CC=CC=C2	0 (21)
**X8/X32**	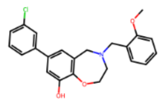	COC1=CC=CC=C1CN2CCOC3=C(C2)C=C(C=C3O)C4=CC(=CC=C4)Cl	4 (47)
**X9/X33**	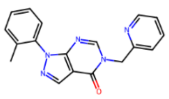	CC1=CC=CC=C1N2C3=C(C=N2)C(=O)N(C=N3)CC4=CC=CC=N4	96 (93)
**X10/X34**	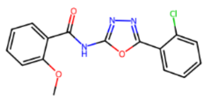	COC1=CC=CC=C1C(=O)NC2=NN=C(O2)C3=CC=CC=C3Cl	8 (42)
**X11/X35**	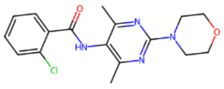	CC1=C(C(=NC(=N1)N2CCOCC2)C)NC(=O)C3=CC=CC=C3Cl	18 (23)

**X12/X36**	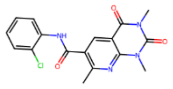	CC1=C(C=C2C(=N1)N(C(=O)N(C2=O)C)C)C(=O)NC3=CC=CC=C3Cl	53 (90)
**X13/X37**	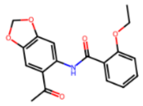	CCOC1=CC=CC=C1C(=O)NC2=CC3=C(C=C2C(=O)C)OCO3	28 (10)
**X14/X38**	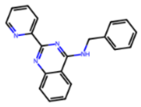	C1=CC=C(C=C1)CNC2=NC(=NC3=CC=CC=C32)C4=CC=CC=N4	20 (19)
**X15/X39**	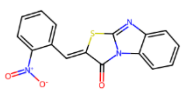	C1=CC=C(C(=C1)C=C2C(=O)N3C4=CC=CC=C4N=C3S2)[N+](=O)[O-]	93 (89)
**X16/X40**	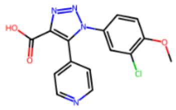	COC1=C(C=C(C=C1)N2C(=C(N=N2)C(=O)O)C3=CC=NC=C3)Cl	11 (30)
**X17/X41**	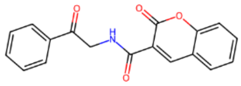	C1=CC=C(C=C1)C(=O)CNC(=O)C2=CC3=CC=CC=C3OC2=O	18 (39)
**X18/X42**	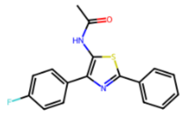	CC(=O)NC1=C(N=C(S1)C2=CC=CC=C2)C3=CC=C(C=C3)F	82 (47)
**X19/X43**	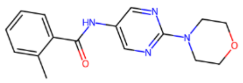	CC1=CC=CC=C1C(=O)NC2=CN=C(N=C2)N3CCOCC3	16 (5)
**X20/X44**	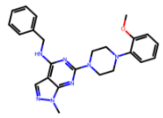	CN1C2=NC(=NC(=C2C=N1)NCC3=CC=CC=C3)N4CCN(CC4)C5=CC=CC=C5OC	8 (45)
**X21/X45**	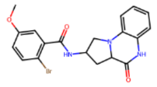	COC1=CC(=C(C=C1)Br)C(=O)NC2CC3C(=O)NC4=CC=CC=C4N3C2	91 (98)
**X22/X46**	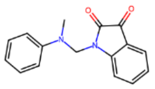	CN(CN1C2=CC=CC=C2C(=O)C1=O)C3=CC=CC=C3	22 (45)
**X23/X47**	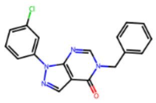	C1=CC=C(C=C1)CN2C=NC3=C(C2=O)C=NN3C4=CC(=CC=C4)Cl	15 (18)

**X24/X48**	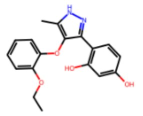	CCOC1=CC=CC=C1OC2=C(NN=C2C3=C(C=C(C=C3)O)O)C	11 (26)
**Y1/Y25**	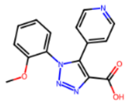	COC1=CC=CC=C1N2C(=C(N=N2)C(=O)O)C3=CC=NC=C3	12 (12)
**Y2/Y26**	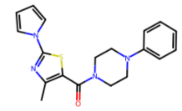	CC1=C(C(N2CCN(C3=CC=CC=C3)CC2)=O)SC(N4C=CC=C4)=N1	14 (0)
**Y3/Y27**	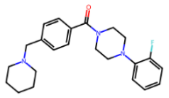	C1CCN(CC1)CC2=CC=C(C=C2)C(=O)N3CCN(CC3)C4=CC=CC=C4F	89 (91)
**Y4/Y28**	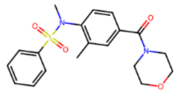	CC1=C(C=CC(=C1)C(=O)N2CCOCC2)N(C)S(=O)(=O)C3=CC=CC=C3	28 (18)
**Y5/Y29**	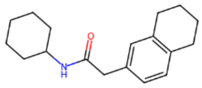	C1CCC(CC1)NC(=O)CC2=CC3=C(CCCC3)C=C2	24 (29)
**Y6/Y30**	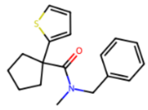	CN(CC1=CC=CC=C1)C(=O)C2(CCCC2)C3=CC=CS3	38 (28)
**Y7/Y31**	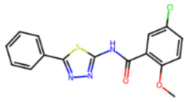	COC1=C(C=C(C=C1)Cl)C(=O)NC2=NN=C(S2)C3=CC=CC=C3	10 (21)
**Y8/Y32**	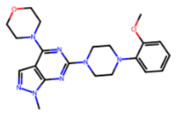	CN1C2=C(C=N1)C(=NC(=N2)N3CCN(CC3)C4=CC=CC=C4OC)N5CCOCC5	15 (63)
**Y9/Y33**	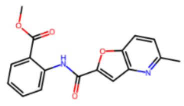	CC1=NC2=C(C=C1)OC(=C2)C(=O)NC3=CC=CC=C3C(=O)OC	17 (24)
**Y10/Y34**	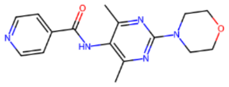	CC1=C(C(=NC(=N1)N2CCOCC2)C)NC(=O)C3=CC=NC=C3	26 (22)
**Y11/Y35**	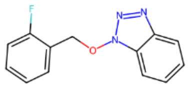	C1=CC=C(C(=C1)CON2C3=CC=CC=C3N=N2)F	62 (57)

**Y12/Y36**	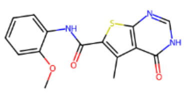	CC1=C(SC2=C1C(=O)NC=N2)C(=O)NC3=CC=CC=C3OC	22 (23)
**Y13/Y37**	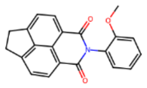	COC1=CC=CC=C1N2C(=O)C3=C4C(=CC=C5C4=C(CC5)C=C3)C2=O	0 (17)
**Y14/Y38**	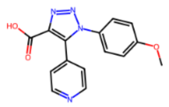	COC1=CC=C(C=C1)N2C(=C(N=N2)C(=O)O)C3=CC=NC=C3	18 (28)
**Y15/Y39**	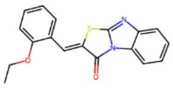	CCOC1=CC=CC=C1C=C2C(=O)N3C4=CC=CC=C4N=C3S2	79 (92)
**Y16/Y40**	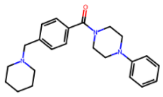	C1CCN(CC1)CC2=CC=C(C=C2)C(=O)N3CCN(CC3)C4=CC=CC=C4	25 (43)
**Y17/Y41**	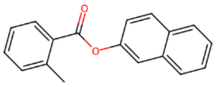	CC1=CC=CC=C1C(=O)OC2=CC3=CC=CC=C3C=C2	14 (27)
**Y18/Y42**	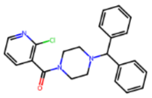	C1CN(CCN1C(C2=CC=CC=C2)C3=CC=CC=C3)C(=O)C4=C(N=CC=C4)Cl	11 (29)
**Y19/Y43**	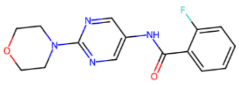	C1COCCN1C2=NC=C(C=N2)NC(=O)C3=CC=CC=C3F	93 (97)
**Y20/Y44**	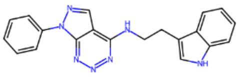	C1=CC=C(C=C1)N2C3=NN=NC(=C3C=N2)NCCC4=CNC5=CC=CC=C54	17 (34)
**Y21/Y45**	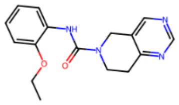	CCOC1=CC=CC=C1NC(=O)N2CCC3=NC=NC=C3C2	8 (24)
**Y22/Y46**	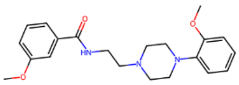	COC1=CC=CC(=C1)C(=O)NCCN2CCN(CC2)C3=CC=CC=C3OC	27 (55)
**Y23/Y47**	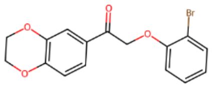	C1COC2=C(O1)C=CC(=C2)C(=O)COC3=CC=CC=C3Br	70 (82)

**Y24/Y48**	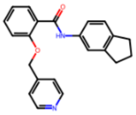	C1CC2=C(C1)C=C(C=C2)NC(=O)C3=CC=CC=C3OCC4=CC=NC=C4	0 (19)
**Z1/Z25**	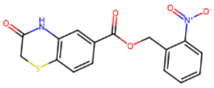	C1C(=O)NC2=C(S1)C=CC(=C2)C(=O)OCC3=CC=CC=C3[N+](=O)[O-]	25 (22)
**Z2/Z26**	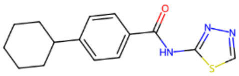	C1CCC(CC1)C2=CC=C(C=C2)C(=O)NC3=NN=CS3	90 (92)
**Z3/Z27**	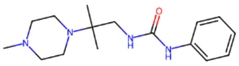	CC(C)(CNC(=O)NC1=CC=CC=C1)N2CCN(CC2)C	22 (29)
**Z4/Z28**	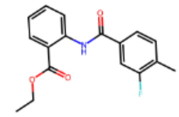	CCOC(=O)C1=CC=CC=C1NC(=O)C2=CC(=C(C=C2)C)F	22 (10)
**Z5/Z29**	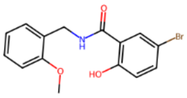	COC1=CC=CC=C1CNC(=O)C2=C(C=CC(=C2)Br)O	32 (29)
**Z6/Z30**	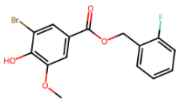	COC1=C(C(=CC(=C1)C(=O)OCC2=CC=CC=C2F)Br)O	27 (25)
**Z7/Z31**	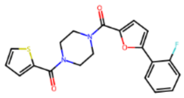	C1CN(CCN1C(=O)C2=CC=C(O2)C3=CC=CC=C3F)C(=O)C4=CC=CS4	28 (51)
**Z8/Z32**	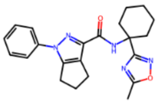	CC1=NC(=NO1)C2(CCCCC2)NC(=O)C3=NN(C4=C3CCC4)C5=CC=CC=C5	28 (49)
**Z9/Z33**	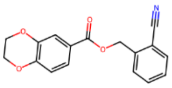	C1COC2=C(O1)C=CC(=C2)C(=O)OCC3=CC=CC=C3C#N	20 (32)
**Z10/Z34**	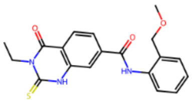	CCN1C(=O)C2=C(C=C(C=C2)C(=O)NC3=CC=CC=C3COC)NC1=S	97 (94)
**Z11/Z35**	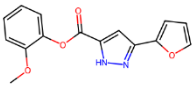	COC1=CC=CC=C1OC(=O)C2=CC(=NN2)C3=CC=CO3	10 (19)

**Z12/Z36**	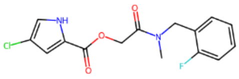	CN(CC1=CC=CC=C1F)C(=O)COC(=O)C2=CC(=CN2)Cl	27 (31)
**Z13/Z37**	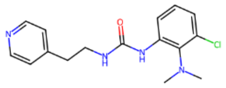	CN(C)C1=C(C=CC=C1Cl)NC(=O)NCC2=CC=NC=C2	54 (90)
**Z14/Z38**	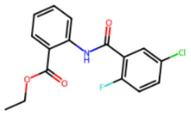	CCOC(=O)C1=CC=CC=C1NC(=O)C2=C(C=CC(=C2)Cl)F	18 (11)
**Z15/Z39**	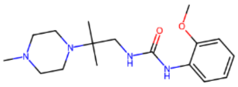	CC(C)(CNC(=O)NC1=CC=CC=C1OC)N2CCN(CC2)C	15 (25)
**Z16/Z40**	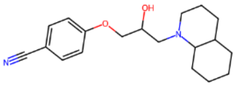	C1CCC2C(C1)CCCN2CC(COC3=CC=C(C=C3)C#N)O	25 (36)
**Z17/Z41**	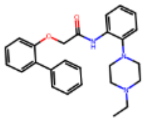	CCN1CCN(CC1)C2=CC=CC=C2NC(=O)COC3=CC=CC=C3C4=CC=CC=C4	43 (46)
**Z18/Z42**	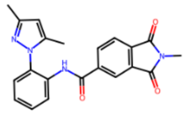	CC1=CC(=NN1C2=CC=CC=C2NC(=O)C3=CC4=C(C=C3)C(=O)N(C4=O)C)C	27 (20)
**Z19/Z43**	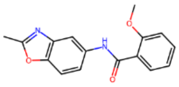	CC1=NC2=C(O1)C=CC(=C2)NC(=O)C3=CC=CC=C3OC	26 (27)
**Z20/Z44**	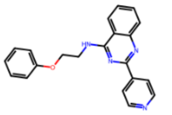	C1=CC=C(C=C1)OCCNC2=NC(=NC3=CC=CC=C32)C4=CC=NC=C4	26 (34)
**Z21/Z45**	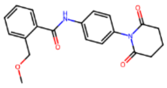	COCC1=CC=CC=C1C(=O)NC2=CC=C(C=C2)N3C(=O)CCCC3=O	94 (97)
**Z22/Z46**	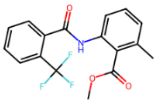	CC1=C(C(=CC=C1)NC(=O)C2=CC=CC=C2C(F)(F)F)C(=O)OC	94 (93)
**Z23/Z47**	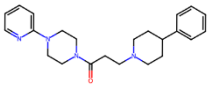	C1CN(CCC1C2=CC=CC=C2)CCC(=O)N3CCN(CC3)C4=CC=CC=N4	15 (32)

**Z24/Z48**	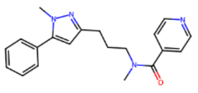	CN1C(=CC(=N1)CCCN(C)C(=O)C2=CC=NC=C2)C3=CC=CC=C3	22 (41)
**AA1/AA25**	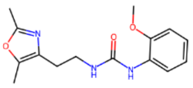	CC1=C(N=C(O1)C)CCNC(=O)NC2=CC=CC=C2OC	24 (6)
**AA2/AA26**	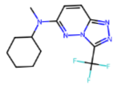	CN(C1CCCCC1)C2=NN3C(=NN=C3C(F)(F)F)C=C2	0 (47)
**AA3/AA27**	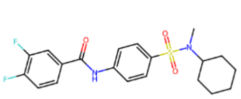	CN(C1CCCCC1)S(=O)(=O)C2=CC=C(C=C2)NC(=O)C3=CC(=C(C=C3)F)F	42 (38)
**AA4/AA28**	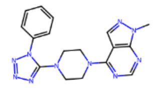	CN1C2=C(C=N1)C(=NC=N2)N3CCN(CC3)C4=NN=NN4C5=CC=CC=C5	13 (23)
**AA5/AA29**	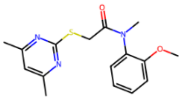	CC1=CC(=NC(=N1)SCC(=O)N(C)C2=CC=CC=C2OC)C	12 (23)
**AA6/AA30**	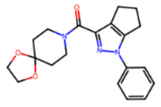	C1CC2=C(C1)N(N=C2C(=O)N3CCC4(CC3)OCCO4)C5=CC=CC=C5	8 (0)
**AA7/AA31**	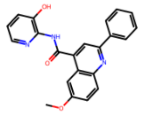	COC1=CC2=C(C=C1)N=C(C=C2C(=O)NC3=C(C=CC=N3)O)C4=CC=CC=C4	10 (52)
**AA8/AA32**	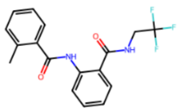	CC1=CC=CC=C1C(=O)NC2=CC=CC=C2C(=O)NCC(F)(F)F	0 (27)
**AA9/AA33**	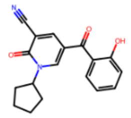	C1CCC(C1)N2C=C(C=C(C2=O)C#N)C(=O)C3=CC=CC=C3O	0 (17)
**AA10/AA34**	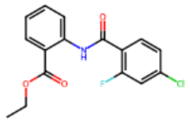	CCOC(=O)C1=CC=CC=C1NC(=O)C2=C(C=C(C=C2)Cl)F	16 (8)
**AA11/AA35**	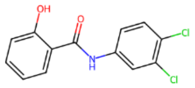	C1=CC=C(C(=C1)C(=O)NC2=CC(=C(C=C2)Cl)Cl)O	22 (22)

**AA12/AA36**	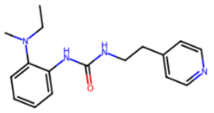	CCN(C)C1=CC=CC=C1NC(=O)NCCC2=CC=NC=C2	95 (98)
**AA13/AA37**	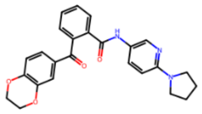	C1CCN(C1)C2=NC=C(C=C2)NC(=O)C3=CC=CC=C3C(=O)C4=CC5=C(C=C4)OCCO5	18 (42)
**AA14/AA38**	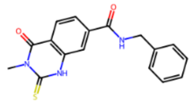	CN1C(=O)C2=C(C=C(C=C2)C(=O)NCC3=CC=CC=C3)NC1=S	96 (47)
**AA15/AA39**	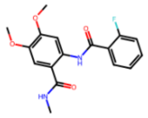	CNC(=O)C1=CC(=C(C=C1NC(=O)C2=CC=CC=C2F)OC)OC	97 (93)
**AA16/AA40**	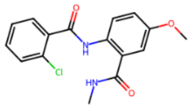	CNC(=O)C1=C(C=CC(=C1)OC)NC(=O)C2=CC=CC=C2Cl	34 (39)
**AA17/AA41**	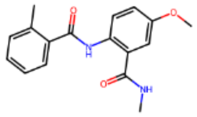	CC1=CC=CC=C1C(=O)NC2=C(C=C(C=C2)OC)C(=O)NC	10 (47)
**AA18/AA42**	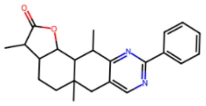	CC1C2CCC3(CC4=CN=C(N=C4C(C3C2OC1=O)C)C5=CC=CC=C5)C	31 (19)
**AA19/AA43**	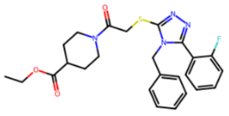	CCOC(=O)C1CCN(CC1)C(=O)CSC2=NN=C(N2CC3=CC=CC=C3)C4=CC=CC=C4F	75 (79)
**AA20/AA44**	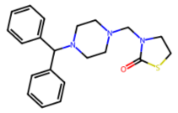	C1CN(CCN1CN2CCSC2=O)C(C3=CC=CC=C3)C4=CC=CC=C4	15 (27)
**AA21/AA45**	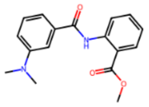	CN(C)C1=CC=CC(=C1)C(=O)NC2=CC=CC=C2C(=O)OC	21 (53)
**AA22/AA46**	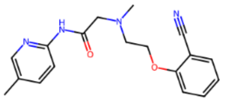	CC1=CN=C(C=C1)NC(=O)CN(C)CCOC2=CC=CC=C2C#N	39 (40)

**AA23/AA47**	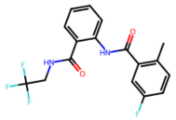	CC1=C(C=C(C=C1)F)C(=O)NC2=CC=CC=C2C(=O)NCC(F)(F)F	97 (91)
**AA24/AA48**	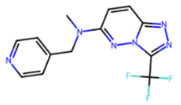	CN(CC1=CC=NC=C1)C2=NN3C(=NN=C3C(F)(F)F)C=C2	93 (96)
**AB1/AB25**	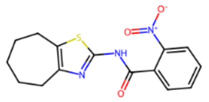	C1CCC2=C(CC1)SC(=N2)NC(=O)C3=CC=CC=C3[N+](=O)[O-]	44 (27)
**AB2/AB26**	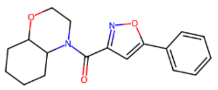	C1CCC2C(C1)N(CCO2)C(=O)C3=NOC(=C3)C4=CC=CC=C4	19 (0)
**AB3/AB27**	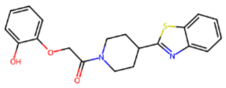	C1CN(CCC1C2=NC3=CC=CC=C3S2)C(=O)COC4=CC=CC=C4O	24 (49)
**AB4/AB28**	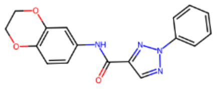	C1COC2=C(O1)C=CC(=C2)NC(=O)C3=NN(N=C3)C4=CC=CC=C4	79 (97)
**AB5/AB29**	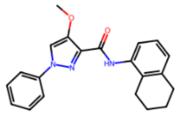	COC1=CN(N=C1C(=O)NC2=CC=CC3=C2CCCC3)C4=CC=CC=C4	98 (89)
**AB6/AB30**	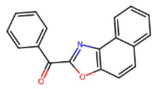	C1=CC=C(C=C1)C(=O)C2=NC3=C(O2)C=CC4=CC=CC=C43	13 (23)
**AB7/AB31**	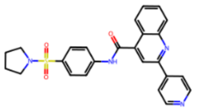	C1CCN(C1)S(=O)(=O)C2=CC=C(C=C2)NC(=O)C3=CC(=NC4=CC=CC=C43)C5=CC=NC=C5	17 (47)
**AB8/AB32**	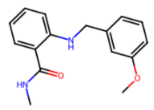	CNC(=O)C1=CC=CC=C1NCC2=CC(=CC=C2)OC	14 (23)
**AB9/AB33**	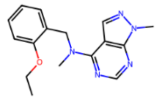	CCOC1=CC=CC=C1CN(C)C2=NC=NC3=C2C=NN3C	52 (92)
**AB10/AB34**	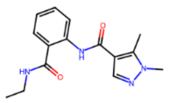	CCNC(=O)C1=CC=CC=C1NC(=O)C2=C(N(N=C2)C)C	21 (14)

**AB11/AB35**	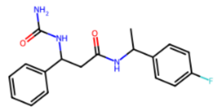	CC(C1=CC=C(C=C1)F)NC(=O)CC(C2=CC=CC=C2)NC(=O)N	26 (48)
**AB12/AB36**	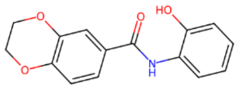	C1COC2=C(O1)C=CC(=C2)C(=O)NC3=CC=CC=C3O	39 (21)
**AB13/AB37**	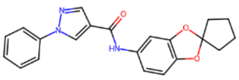	C1CCC2(C1)OC3=C(O2)C=C(C=C3)NC(=O)C4=CN(N=C4)C5=CC=CC=C5	95 (93)
**AB14/AB38**	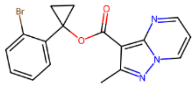	CC1=NN2C=CC=NC2=C1C(=O)OC3(CC3)C4=CC=CC=C4Br	29 (22)
**AB15/AB39**	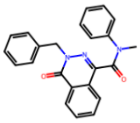	CN(C1=CC=CC=C1)C(=O)C2=NN(C(=O)C3=CC=CC=C32)CC4=CC=CC=C4	25 (22)
**AB16/AB40**	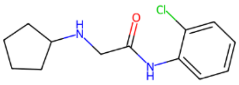	C1CCC(C1)NCC(=O)NC2=CC=CC=C2Cl	90 (88)
**AB17/AB41**	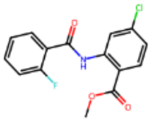	COC(=O)C1=C(C=C(C=C1)Cl)NC(=O)C2=CC=CC=C2F	94 (90)
**AB18/AB42**	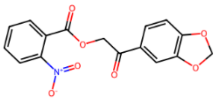	C1OC2=C(O1)C=C(C=C2)C(=O)COC(=O)C3=CC=CC=C3[N+](=O)[O-]	89 (91)
**AB19/AB43**	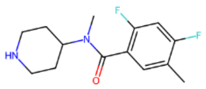	CC1=CC(=C(C=C1F)F)C(=O)N(C)C2CCNCC2	25 (28)
**AB20/AB44**	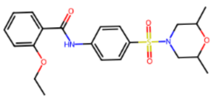	CCOC1=CC=CC=C1C(=O)NC2=CC=C(C=C2)S(=O)(=O)N3CC(OC(C3)C)C	10 (47)
**AB21/AB45**	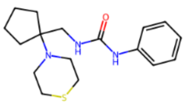	C1CCC(C1)(CNC(=O)NC2=CC=CC=C2)N3CCSCC3	14 (26)
**AB22/AB46**	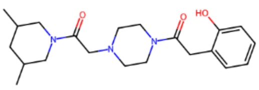	CC1CC(CN(C1)C(=O)CN2CCN(CC2)C(=O)CC3=CC=CC=C3O)C	38 (40)

**AB23/AB47**	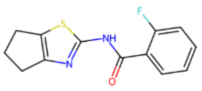	C1CC2=C(C1)SC(=N2)NC(=O)C3=CC=CC=C3F	16 (28)
**AB24/AB48**	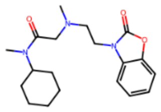	CN(CCN1C2=CC=CC=C2OC1=O)CC(=O)N(C)C3CCCCC3	14 (30)
**AC1/AC25**	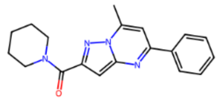	CC1=CC(=NC2=CC(=NN12)C(=O)N3CCCCC3)C4=CC=CC=C4	94 (88)
**AC2/AC26**	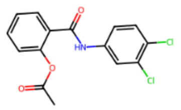	CC(=O)OC1=CC=CC=C1C(=O)NC2=CC(=C(C=C2)Cl)Cl	80 (36)
**AC3/AC27**	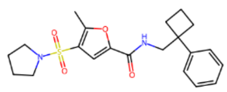	CC1=C(C=C(O1)C(=O)NCC2(CCC2)C3=CC=CC=C3)S(=O)(=O)N4CCCC4	25 (13)
**AC4/AC28**	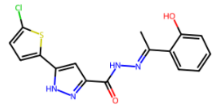	CC(=NNC(=O)C1=NNC(=C1)C2=CC=C(S2)Cl)C3=CC=CC=C3O	25 (48)
**AC5/AC29**	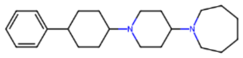	C1CCCN(CC1)C2CCN(CC2)C3CCC(CC3)C4=CC=CC=C4	49 (98)
**AC6/AC30**	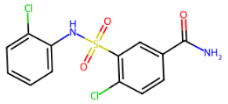	C1=CC=C(C(=C1)NS(=O)(=O)C2=C(C=CC(=C2)C(=O)N)Cl)Cl	19 (58)
**AC7/AC31**	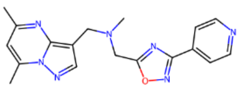	CC1=CC(=NC2=C(C=NN12)CN(C)CC3=NC(=NO3)C4=CC=NC=C4)C	4 (43)
**AC8/AC32**	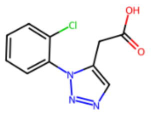	C1=CC=C(C(=C1)N2C(=CN=N2)CC(=O)O)Cl	27 (29)
**AC9/AC33**	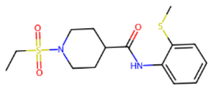	CCS(=O)(=O)N1CCC(CC1)C(=O)NC2=CC=CC=C2SC	8 (47)
**AC10/AC34**	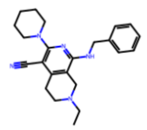	CCN1CCC2=C(C1)C(=NC(=C2C#N)N3CCCCC3)NCC4=CC=CC=C4	35 (90)

**AC11/AC35**	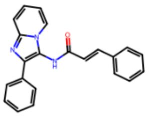	C1=CC=C(C=C1)C=CC(=O)NC2=C(N=C3N2C=CC=C3)C4=CC=CC=C4	22 (0)
**AC12/AC36**	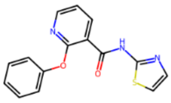	C1=CC=C(C=C1)OC2=C(C=CC=N2)C(=O)NC3=NC=CS3	20 (18)
**AC13/AC37**	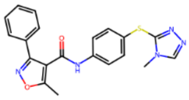	CC1=C(C(=NO1)C2=CC=CC=C2)C(=O)NC3=CC=C(C=C3)SC4=NN=CN4C	22 (20)
**AC14/AC38**	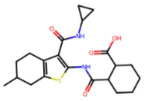	CC1CCC2=C(C1)SC(=C2C(=O)NC3CC3)NC(=O)C4CCCCC4C(=O)O	94 (97)
**AC15/AC39**	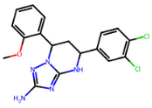	COC1=CC=CC=C1C2CC(NC3=NC(=NN23)N)C4=CC(=C(C=C4)Cl)Cl	28 (0)
**AC16/AC40**	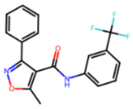	CC1=C(C(=NO1)C2=CC=CC=C2)C(=O)NC3=CC=CC(=C3)C(F)(F)F	49 (57)
**AC17/AC41**	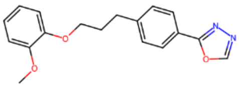	COC1=CC=CC=C1OCCCC2=CC=C(C=C2)C3=NN=CO3	20 (11)
**AC18/AC42**	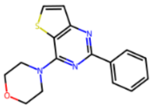	C1COCCN1C2=NC(=NC3=C2SC=C3)C4=CC=CC=C4	11 (54)
**AC19/AC43**	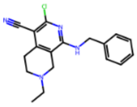	CCN1CCC2=C(C1)C(=NC(=C2C#N)Cl)NCC3=CC=CC=C3	30 (59)
**AC20/AC44**	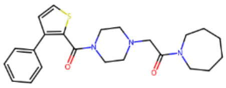	C1CCCN(CC1)C(=O)CN2CCN(CC2)C(=O)C3=C(C=CS3)C4=CC=CC=C4	29 (24)
**AC21/AC45**	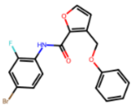	C1=CC=C(C=C1)OCC2=C(OC=C2)C(=O)NC3=C(C=C(C=C3)Br)F	29 (25)
**AC22/AC46**	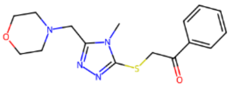	CN1C(=NN=C1SCC(=O)C2=CC=CC=C2)CN3CCOCC3	65 (35)

**AC23/AC47**	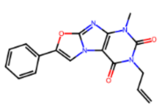	CN1C2=C(C(=O)N(C1=O)CC=C)N3C=C(OC3=N2)C4=CC=CC=C4	25 (15)
**AC24/AC48**	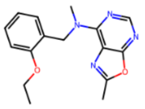	CCOC1=CC=CC=C1CN(C)C2=C3C(=NC=N2)OC(=N3)C	12 (18)
**AD1/AD25**	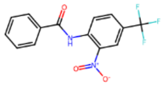	C1=CC=C(C=C1)C(=O)NC2=C(C=C(C=C2)C(F)(F)F)[N+](=O)[O-]	26 (60)
**AD2/AD26**	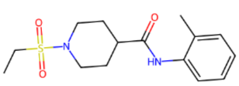	CCS(=O)(=O)N1CCC(CC1)C(=O)NC2=CC=CC=C2C	57 (92)
**AD3/AD27**	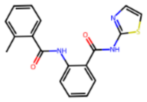	CC1=CC=CC=C1C(=O)NC2=CC=CC=C2C(=O)NC3=NC=CS3	24 (28)
**AD4/AD28**	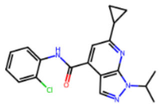	CC(C)N1C2=C(C=N1)C(=CC(=N2)C3CC3)C(=O)NC4=CC=CC=C4Cl	28 (48)
**AD5/AD29**	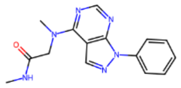	CNC(=O)CN(C)C1=NC=NC2=C1C=NN2C3=CC=CC=C3	42 (96)
**AD6/AD30**	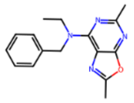	CCN(CC1=CC=CC=C1)C2=C3C(=NC(=N2)C)OC(=N3)C	12 (20)
**AD7/AD31**	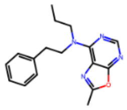	CCCN(CCC1=CC=CC=C1)C2=C3C(=NC=N2)OC(=N3)C	40 (20)
**AD8/AD32**	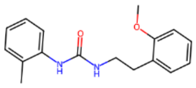	CC1=CC=CC=C1NC(=O)NCCC2=CC=CC=C2OC	23 (57)
**AD9/AD33**	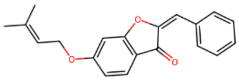	CC(=CCOC1=CC2=C(C=C1)C(=O)C(=CC3=CC=CC=C3)O2)C	52 (26)
**AD10/AD34**	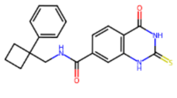	C1CC(C1)(CNC(=O)C2=CC3=C(C=C2)C(=O)NC(=S)N3)C4=CC=CC=C4	0 (23)

**AD11/AD35**	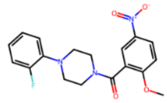	COC1=C(C=C(C=C1)[N+](=O)[O-])C(=O)N2CCN(CC2)C3=CC=CC=C3F	0 (0)
**AD12/AD36**	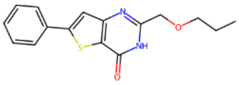	CCCOCC1=NC2=C(C(=O)N1)SC(=C2)C3=CC=CC=C3	30 (30)
**AD13/AD37**	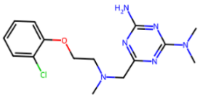	CN(C)C1=NC(=NC(=N1)N)CN(C)CCOC2=CC=CC=C2Cl	55 (22)
**AD14/AD38**	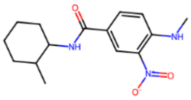	CC1CCCCC1NC(=O)C2=CC(=C(C=C2)NC)[N+](=O)[O-]	15 (27)
**AD15/AD39**	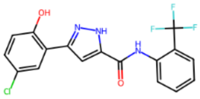	C1=CC=C(C(=C1)C(F)(F)F)NC(=O)C2=CC(=NN2)C3=C(C=CC(=C3)Cl)O	27 (59)
**AD16/AD40**	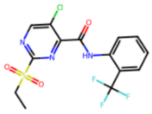	CCS(=O)(=O)C1=NC=C(C(=N1)C(=O)NC2=CC=CC=C2C(F)(F)F)Cl	64 (95)
**AD17/AD41**	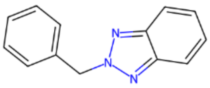	C1=CC=C(C=C1)CN2N=C3C=CC=CC3=N2	97 (97)
**AD18/AD42**	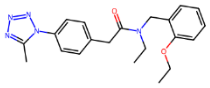	CCN(CC1=CC=CC=C1OCC)C(=O)CC2=CC=C(C=C2)N3C(=NN=N3)C	98 (88)
**AD19/AD43**	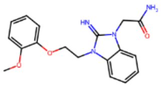	COC1=CC=CC=C1OCCN2C3=CC=CC=C3N(C2=N)CC(=O)N	90 (95)
**AD20/AD44**	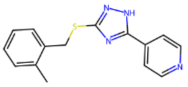	CC1=CC=CC=C1CSC2=NNC(=N2)C3=CC=NC=C3	91 (89)
**AD21/AD45**	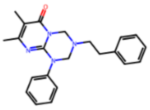	CC1=C(N=C2N(CN(CN2C1=O)CCC3=CC=CC=C3)C4=CC=CC=C4)C	35 (58)
**AD22/AD46**	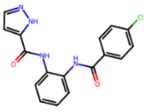	C1=CC=C(C(=C1)NC(=O)C2=CC=C(C=C2)Cl)NC(=O)C3=CC=NN3	19 (26)

**AD23/AD47**	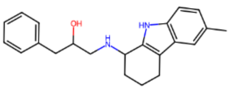	CC1=CC2=C(C=C1)NC3=C2CCCC3NCC(CC4=CC=CC=C4)O	23 (73)
**AD24/AD48**	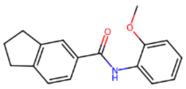	COC1=CC=CC=C1NC(=O)C2=CC3=C(CCC3)C=C2	27 (97)
**AE1/AE25**	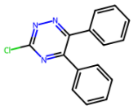	C1=CC=C(C=C1)C2=C(N=NC(=N2)Cl)C3=CC=CC=C3	12 (26)
**AE2/AE26**	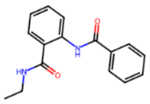	CCNC(=O)C1=CC=CC=C1NC(=O)C2=CC=CC=C2	32 (30)
**AE3/AE27**	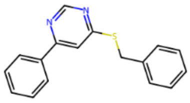	C1=CC=C(C=C1)CSC2=NC=NC(=C2)C3=CC=CC=C3	36 (27)
**AE4/AE28**	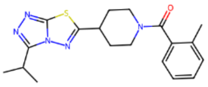	CC1=CC=CC=C1C(=O)N2CCC(CC2)C3=NN4C(=NN=C4S3)C(C)C	21 (45)
**AE5/AE29**	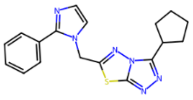	C1CCC(C1)C2=NN=C3N2N=C(S3)CN4C=CN=C4C5=CC=CC=C5	13 (28)
**AE6/AE30**	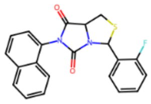	C1C2C(=O)N(C(=O)N2C(S1)C3=CC=CC=C3F)C4=CC=CC5=CC=CC=C54	83 (90)
**AE7/AE31**	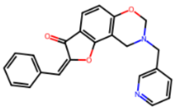	C1C2=C(C=CC3=C2OC(=CC4=CC=CC=C4)C3=O)OCN1CC5=CN=CC=C5	93 (91)
**AE8/AE32**	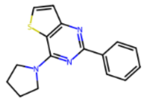	C1CCN(C1)C2=NC(=NC3=C2SC=C3)C4=CC=CC=C4	89 (94)
**AE9/AE33**	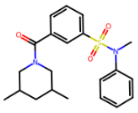	CC1CC(CN(C1)C(=O)C2=CC(=CC=C2)S(=O)(=O)N(C)C3=CC=CC=C3)C	24 (21)
**AE10/AE34**	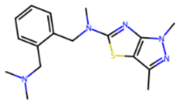	CC1=NN(C2=C1SC(=N2)N(C)CC3=CC=CC=C3CN(C)C)C	10 (12)

**AE11/AE35**	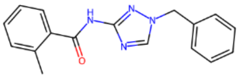	CC1=CC=CC=C1C(=O)NC2=NN(C=N 2)CC3=CC=CC=C3	18 (25)
**AE12/AE36**	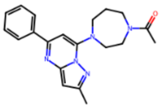	CC1=NN2C(=C1)N=C(C=C2N3CCCN (CC3)C(=O)C)C4=CC=CC=C4	22 (36)
**AE13/AE37**	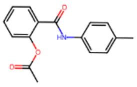	CC1=CC=C(C=C1)NC(=O)C2=CC=CC =C2OC(=O)C	0 (7)
**AE14/AE38**	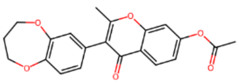	CC(OC1=CC=C2C(OC(C)=C(C2=O)C 3=CC4=C(OCCCO4)C=C3)=C1)=O	23 (25)
**AE15/AE39**	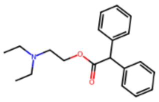	CCN(CC)CCOC(=O)C(C1=CC=CC=C 1)C2=CC=CC=C2	44 (54)
**AE16/AE40**	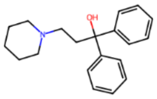	C1CCN(CC1)CCC(C2=CC=CC=C2)(C 3=CC=CC=C3)O	35 (92)
**AE17/AE41**	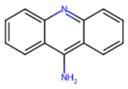	C1=CC=C2C(=C1)C(=C3C=CC=CC3= N2)N	35 (62)
**AE18/AE42**	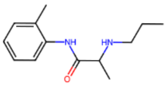	CCCNC(C)C(=O)NC1=CC=CC=C1C	59 (88)
**AE19/AE43**	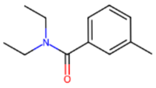	CCN(CC)C(=O)C1=CC=CC(=C1)C	51 (72)
**AE20/AE44**	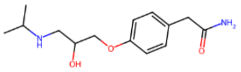	CC(C)NCC(COC1=CC=C(C=C1)CC(= O)N)O	21 (54)
**AE21/AE45**	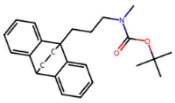	CN(C(OC(C)(C)C)=O)CCCC12CCC(C 3=CC=CC=C23)C4=CC=CC=C41	90 (97)
**AE22/AE46**	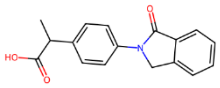	CC(C1=CC=C(C=C1)N2CC3=CC=CC =C3C2=O)C(=O)O	60 (71)

**AE23/AE47**	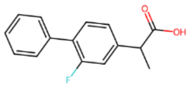	CC(C1=CC(=C(C=C1)C2=CC=CC=C2)F)C(=O)O	26 (40)
**AE24/AE48**	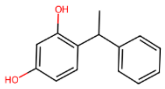	CC(C1=CC=CC=C1)C2=C(O)C=C(O)C=C2	85 (97)
**AF1/AF25**	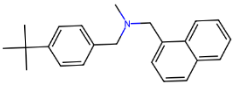	CC(C)(C)C1=CC=C(C=C1)CN(C)CC2=CC=CC3=CC=CC=C32	41 (88)
**AF2/AF26**	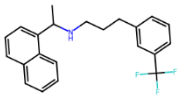	CC(C1=CC=CC2=CC=CC=C21)NCCCC3=CC(=CC=C3)C(F)(F)F	92 (97)
**AF3/AF27**	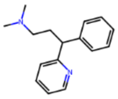	CN(C)CCC(C1=CC=CC=C1)C2=CC=CC=N2	0 (24)
**AF4/AF28**	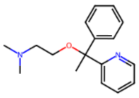	CC(C1=CC=CC=C1)(C2=CC=CC=N2)OCCN(C)C	92 (92)
**AF5/AF29**	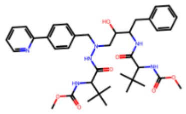	CC(C)(C)C(C(=O)NC(CC1=CC=CC=C1)C(CN(CC2=CC=C(C=C2)C3=CC=CC=N3)NC(=O)C(C(C)(C)C)NC(=O)OC)O)NC(=O)OC	91 (92)
**AF6/AF30**	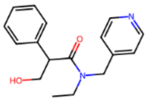	CCN(CC1=CC=NC=C1)C(=O)C(CO)C2=CC=CC=C2	33 (75)
**AF7/AF31**	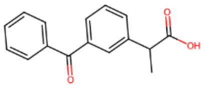	CC(C1=CC(=CC=C1)C(=O)C2=CC=CC=C2)C(=O)O	17 (24)
**AF8/AF32**	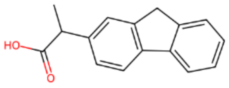	CC(C1=CC2=C(C=C1)C3=CC=CC=C3C2)C(=O)O	36 (69)
**AF9/AF33**	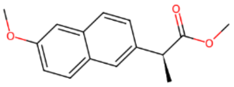	O=C([C@H](C1=CC2=CC=C(OC)C=C2C=C1)C)OC	27 (40)

**AF10/AF34**	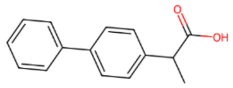	CC(C1=CC=C(C=C1)C2=CC=CC=C2)C(=O)O	17 (13)
**AF11/AF35**	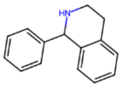	C1CNC(C2=CC=CC=C21)C3=CC=CC=C3	20 (23)
**AF12/AF36**	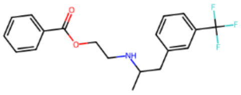	CC(CC1=CC(=CC=C1)C(F)(F)F)NCCOC(=O)C2=CC=CC=C2	16 (0)
**AF13/AF37**	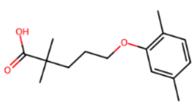	CC1=CC(=C(C=C1)C)OCCCC(C)(C)C(=O)O	93 (96)
**AF14/AF38**	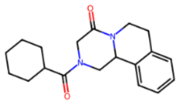	C1CCC(CC1)C(=O)N2CC3C4=CC=CC=C4CCN3C(=O)C2	71 (95)
**AF15/AF39**	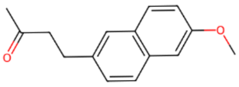	CC(=O)CCC1=CC2=C(C=C1)C=C(C=C2)OC	89 (94)
**AF16/AF40**	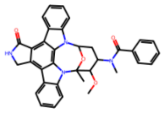	CC12C(C(CC(O1)N3C4=CC=CC=C4C5=C6C(=C7C8=CC=CC=C8N2C7=C53)CNC6=O)N(C)C(=O)C9=CC=CC=C9)OC	53 (62)
**AF17/AF41**	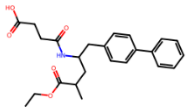	CCOC(=O)C(C)CC(CC1=CC=C(C=C1)C2=CC=CC=C2)NC(=O)CCC(=O)O	10 (24)
**AF18/AF42**	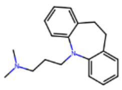	CN(C)CCCN1C2=CC=CC=C2CCC3=CC=CC=C31	20 (49)
**AF19/AF43**	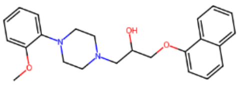	COC1=CC=CC=C1N2CCN(CC2)CC(COC3=CC=CC4=CC=CC=C43)O	65 (96)
**AF20/AF44**	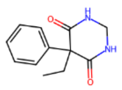	CCC1(C(=O)NCNC1=O)C2=CC=CC=C2	93 (90)

**AF21/AF45**	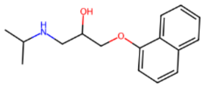	CC(C)NCC(COC1=CC=CC2=CC=CC=C21)O	92 (21)
**AF22/AF46**	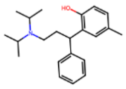	CC1=CC(=C(C=C1)O)C(CCN(C(C)C)C(C)C)C2=CC=CC=C2	96 (95)
**AF23/AF47**	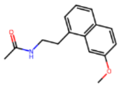	CC(=O)NCCC1=CC=CC2=C1C=C(C=C2)OC	92 (97)
**AF24/AF48**	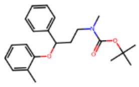	CC1=CC=CC=C1OC(C2=CC=CC=C2)CCN(C(OC(C)(C)C)=O)C	12 (15)

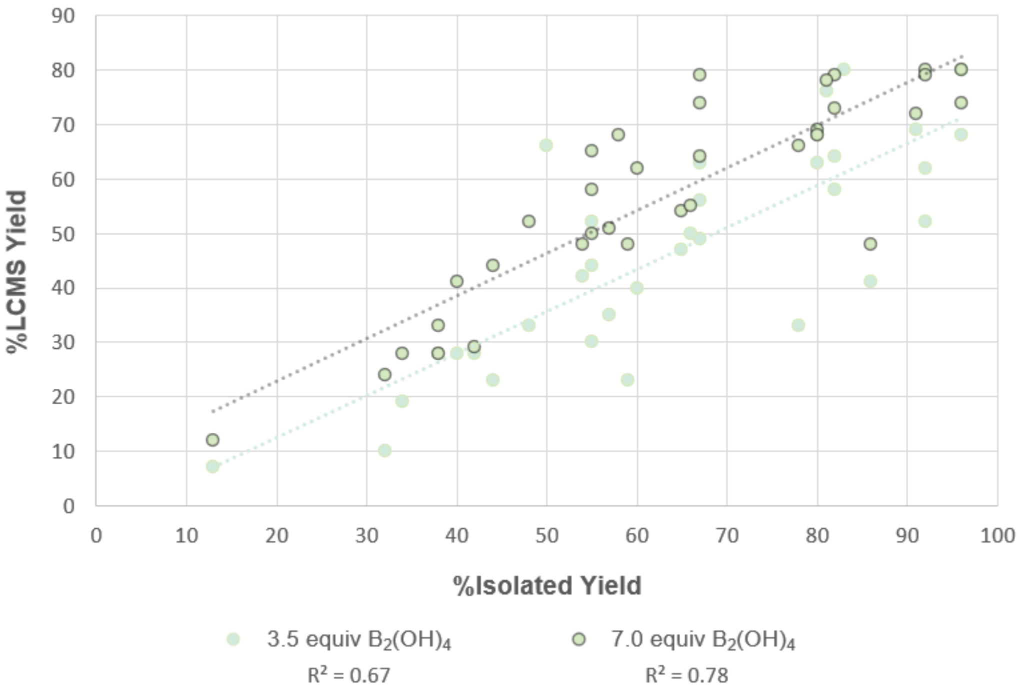

Figure S20 Comparison of reaction performance, %LCMS Conversion versus % Isolated Yield, for 34 selected compounds from the 690 total compounds that were successfully reduced in ultraHTE format in Figure 5.

8.6 Procedure for High-Throughput Human Liver Microsomal Stability Assay

General setup and execution of the high-throughput experiment to be coupled directly into the microsomal stability assay remains the same as outlined in the above section "Procedure for Arene Reduction Comparison Screen." Stock solutions, or suspensions, of the appropriate reagents were prepared, under inert atmosphere, as detailed in the heatmap preparation table below. A 24-well aluminum microvial plate (Analytical Sales & Services #24253) was equipped with oven-dried shell vials (Analytical Sales & Services cat. no. 884001) and parylene-coated stir dowels were added to each vial (Analytical Sales & Services #13258). All stock solutions were prepared in EtOH and dosed into the appropriate well locations using single or multichannel micropipetters. Sequence of addition is as follows: substrate addition, $B_2(OH)_4$ addition, and finally $[Rh(COD)OH]_2$ addition to reach the final reaction volume (100 μL). The microvial plate was quickly sealed, removed from the glove box, and stirred on a tumble stirrer with heating to 50°C for 24 hours.

The reactions were quenched by opening the reaction block and adding 100 μL saturated aqueous $NaHCO_3$ solution, then the reaction plate was allowed to stir for 15 minutes. Following, approximately 3 mg (0.63 mmol/g loading, >5.0 equiv relative to Rh loading) of SiliaMetS DMT metal scavenging resin (SiliCycle #R79030B) was added to each well and stirred for another 60 minutes to sequester remaining rhodium catalyst. From each reaction, a 20 μL aliquot of the quenched reaction mixture was transferred to a separate vial containing 80 μL of acetonitrile to reach a final concentration of 10 mM for each crude reaction mixture to be tested in the metabolism assay.

The human liver microsomal stability assay was conducted by slightly adapting the previous high- throughput protocol[22] for UPLC-MS analysis. Briefly, 197.5 μL pre-warmed potassium phosphate buffer (0.1 M, pH 7.4) was added to a 96-well plate (Greiner # Z667234) via multichannel micropipettor and incubated for 5 minutes at 37°C. Then, NADPH generating system (25 μL, 10 mM, XenoTech # K5100) was added to all wells (using the buffer as a substitute for the negative control). Then, stock solutions of the test compounds (2.5 μL, 10 mM) were added to the assay plate. Finally, each well was charged with thawed mixed gender human liver microsomes (12.5 μL, 20 mg/mL, XenoTech # H0604). The final solutions were gently mixed by pipetting up and down two to three times. Immediately, 50 μL of each sample for the 0-minute time point was withdrawn and transfered to a quench solution of 100 μL ice-cold acetonitrile with internal standard (100 mg/L, caffeine).

The well plate was incubated at 37°C for 60 minutes with additional aliquots taken from the corresponding wells at the 15, 30, and 60 minute marks to be quenched. The quenched acetonitrile plate was then centrifuged for 5 minutes at 3500 × g. Finally, 100 µl of the quenched supernatant fraction was transferred into a 96-well polypropylene collection plate for analysis. Samples were stored at -20 °C until analyzed by UPLC-MS. Percent remaining of each substrate was determined by comparing the ratio of MS ionization peak area of each substrate/caffeine relative to the substrate/caffeine area at the 0-minute time point. Additionally, the pure unsaturated starting materials for each substrate were analyzed by the same assay procedure to determine the percent change in metabolism over 60 minutes between the unsaturated drug and it reduced congener. Data for the 20 of 24 substrates that were detected at quantitatable concentrations following LSS and subseqeuent microsome exposure are shown below in **Table S25** and **Figure S22.**

**Table S24.** Recipe for 24-Well LSS Screen for Microsomal Stability Studies			
**Reagents**	**C_{stock} (M)**	**V_{dose} (µL)**	**Wells**
Gemfibrozil	0.3	33	A1
(±)-Propranolol	0.3	33	A2
Aminacrine	0.3	33	A3
Ketoprofen	0.3	33	A4
Atorvastatin Calcium	0.3	33	A5
Atenolol	0.3	33	A6
Diethyltoluamide	0.3	33	B1
Praziquantel	0.3	33	B2
Prilocaine	0.3	33	B3
Indoprofen	0.3	33	B4
Flurbiprofen	0.3	33	B5
Nabumetone	0.3	33	B6
Naftopidil	0.3	33	C1
Sacubitril	0.3	33	C2
Atazanavir	0.3	33	C3
Tropicamide	0.3	33	C4
Imipramine	0.3	33	C5
(*S*)-Naproxen-OMe	0.3	33	C6
Butenafine	0.3	33	D1
Boc-Maprolitine	0.3	33	D2
Primidone	0.3	33	D3
Benfluorex	0.3	33	D4
(*R*)-Cinacalcet	0.3	33	D5
Phenethyl Resorcinol	0.3	33	D6
$B_2(OH)_4$ (5.0 equiv)	1.5	33	All
$[Rh(COD)OH]_2$	0.012	33	All

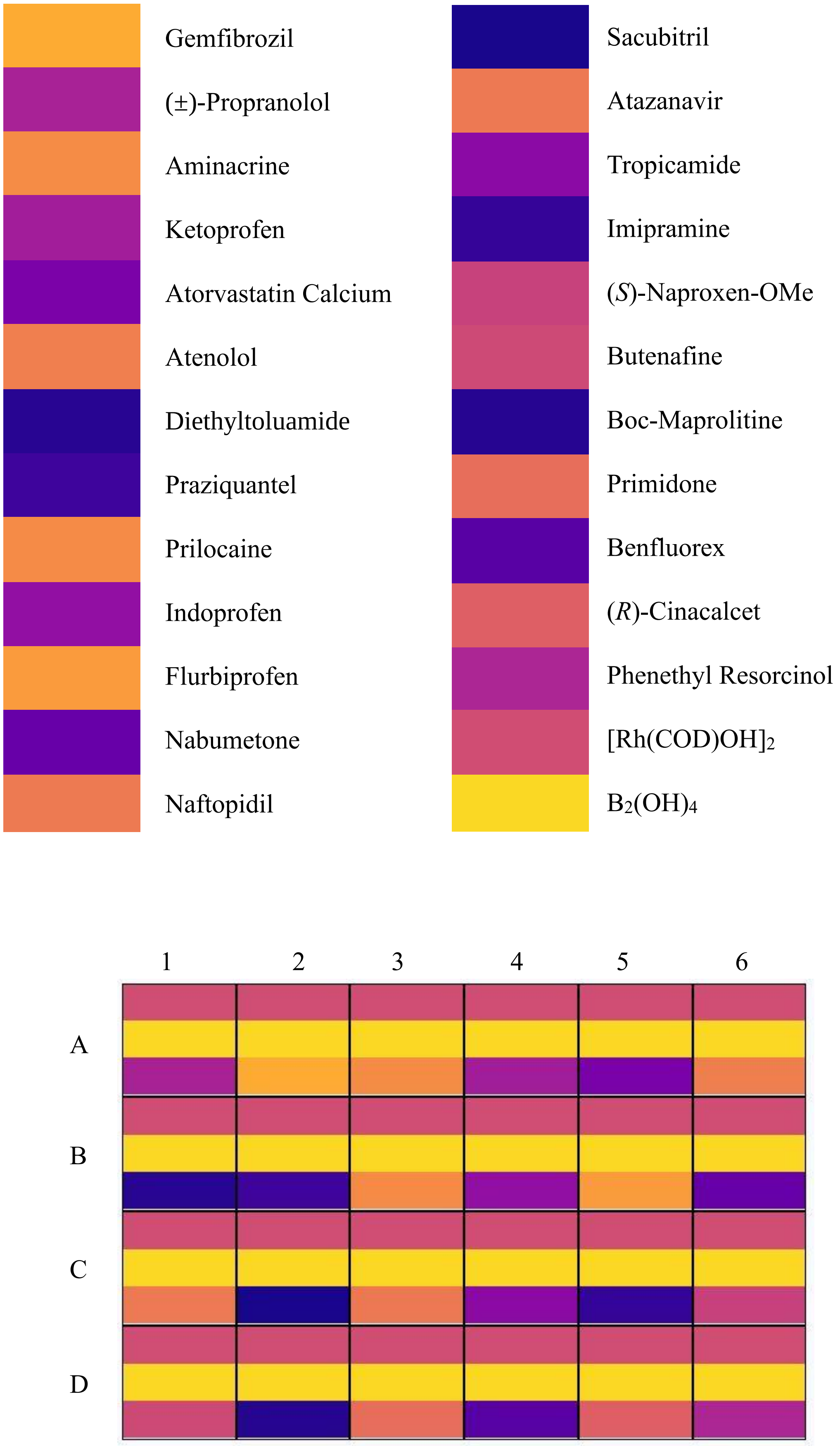

Figure S21. Reagents and Grid of Screen for Microsomal Stability Studies

**Table S25.** Comparison of %Remaining of Compounds in human liver microsomes									
**Compound ID**	**%Remaining Before LSS**				**%Remaining After LSS**				**%Change After 60 min**
	**0 min**	**15 min**	**30 min**	**60 min**	**0 min**	**15 min**	**30 min**	**60 min**	
Gemfibrozil	N.D.	N.D.	N.D.	**N.D.**	N.D.	N.D.	N.D.	**N.D.**	**-**
(±)-Propranolol	100	95.73	92.97	**90.62**	100	92.51	85.76	**81.28**	**-9.35**
Aminacrine	100	83.65	75.11	**66.24**	100	81.54	68.51	**53.85**	**-12.39**
Ketoprofen	100	61.21	39.88	**30.47**	100	60.22	37.09	**28.25**	**-2.22**
Atorvastatin Calcium	100	96.55	93.76	**91.69**	100	91.03	83.61	**78.58**	**-13.1**
Atenolol	100	90.62	82.55	**75.78**	100	89.79	82.82	**77.49**	**1.71**
Diethyltoluamide	100	89.01	81.69	**75.45**	100	89.66	82.06	**76.26**	**0.82**
Praziquantel	100	62.99	31.78	**15.26**	100	60.01	27.08	**0**	**-15.26**
Prilocaine	100	86.01	75.01	**67.4**	100	78.04	64.04	**53.63**	**-13.77**
Indoprofen	100	97.61	99.05	**101.53**	100	95.1	91.05	**87.49**	**-14.04**
Flurbiprofen	100	50.08	25.06	**9.28**	100	65.01	29.01	**16.41**	**7.13**
Nabumetone	100	58.01	40.66	**21.73**	100	57.53	37.99	**26.97**	**5.23**
Naftopidil	100	72.22	58.9	**47.01**	100	69.53	39.56	**21.37**	**-25.63**
Sacubitril	100	60.16	34.05	**22.58**	100	58.76	33.78	**20.67**	**-1.91**
Atazanavir	100	84.65	72.9	**65.29**	100	81.14	68.89	**59.92**	**-5.37**
Tropicamide	100	95.19	91.88	**88.12**	100	93.81	89.56	**85.51**	**-2.61**
Imipramine	100	76.13	62.51	**53.47**	100	74.77	59.61	**48.1**	**-5.36**
(*S*)-Naproxen-OMe	N.D.	N.D.	N.D.	**N.D.**	100	N.D.	N.D.	**N.D.**	**-**
Butenafine	100	97.75	95.61	**94.86**	100	96.73	93.97	**92.17**	**-2.69**
Boc-Maprolitine	100	71.56	59.39	**45.29**	100	69.65	46.83	**38.05**	**-7.24**
Primidone	100	71.03	43.6	**27.78**	100	70.43	44.65	**29.94**	**2.16**
Benfluorex	100	N.D.	N.D.	**N.D.**	N.D.	N.D.	N.D.	**N.D.**	**-**
(*R*)-Cinacalcet	100	67.41	49.02	**35.4**	100	63.72	45.98	**33.7**	**-1.7**
Phenethyl Resorcinol	N.D.	N.D.	N.D.	**N.D.**	N.D.	N.D.	N.D.	**N.D.**	**-**

N.D. = Not determined

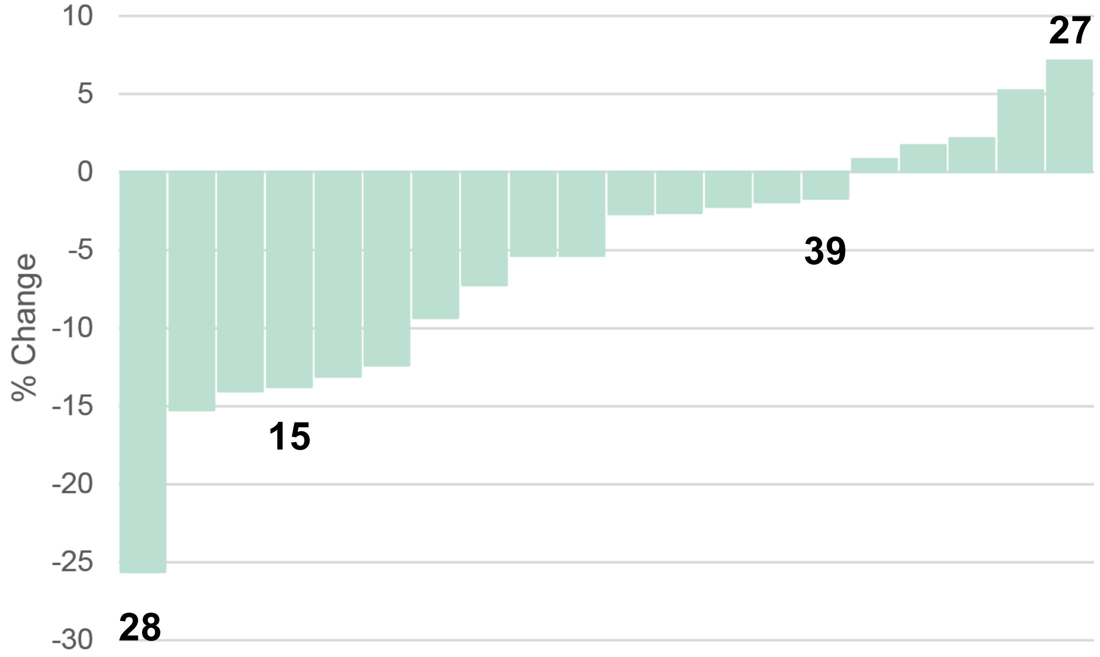

Figure S22. Difference in metabolic stability of 20 drugs determined before and after late-stage saturation. Ordered by increasing metabolic stability of saturated congener relative to its unsaturated parent material.

9. Relative Configuration Consideration

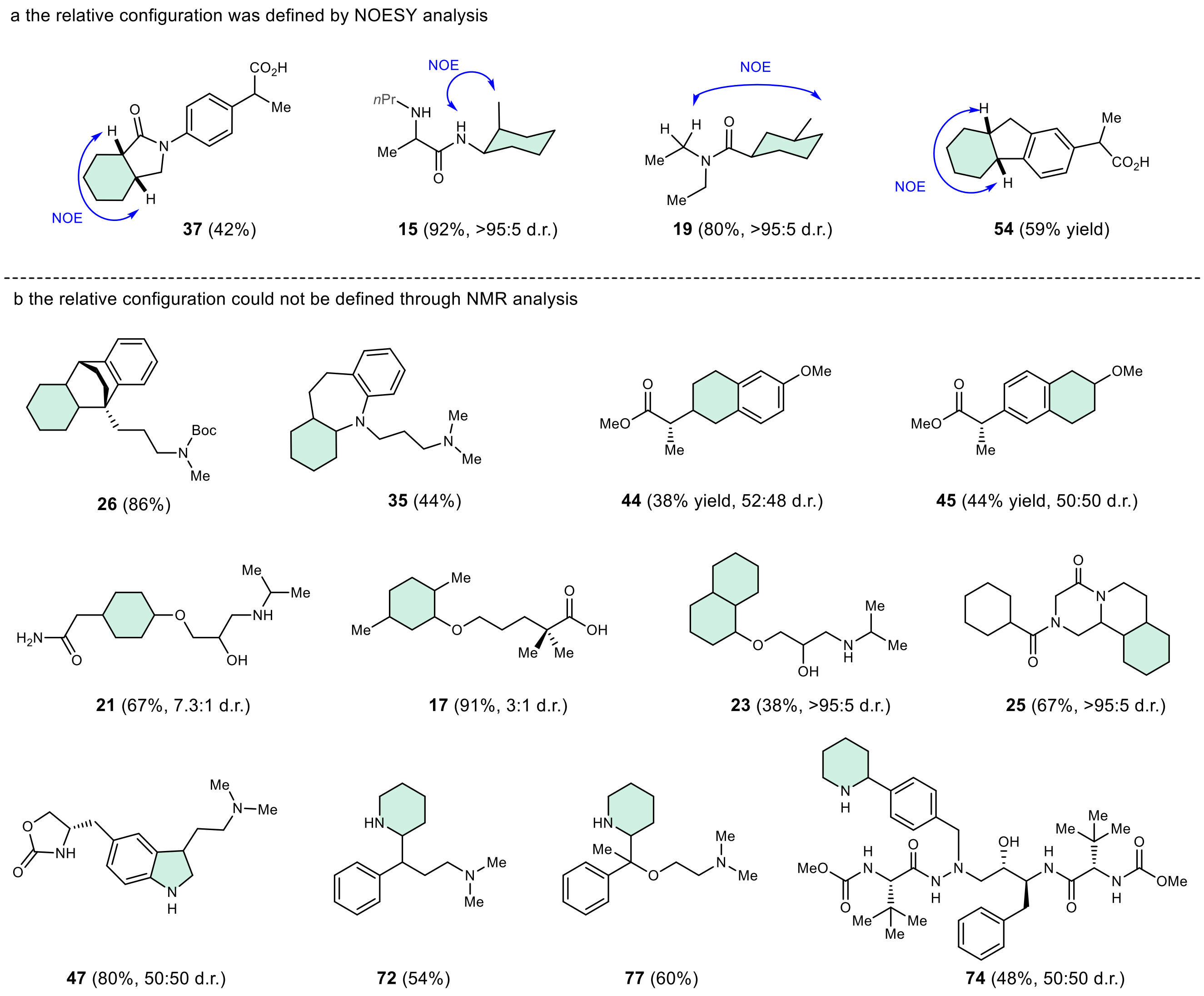

Figure S23. Relative configuration consideration

10. Measurement of LogPo/w

General procedure

1). Preparation of standard solutions for compound.

The compound (5.00 mg) was weighed precisely, placed in a 10 mL volumetric flask, added with acetonitrile and dissolved by ultrasonication, fixed volume, and shaken well to obtain a solution of the compound with a mass concentration of 0.5 mg/mL.

2). Establishment of standard curve.

To acetonitrile (1.0 ml) 10, 20, 30, 40, 50, 60, 70 μl of the solution were added to obtain 7 sets of standard solutions of A1 to A7 with different mass concentrations. The samples (A1 - A7) were filtered through 0.22 μm microporous filter membrane and injected into Reversed-phase HPLC (RP-HPLC) for determination and the peak areas (Y1 - Y7) were recorded. The regression equation in table S26 was derived by linear regression analysis using the mass concentration (X, mg/mL) of the compounds as the horizontal coordinate and the peak area value (Y) as the vertical coordinate. (linear regression equation: Y = a•X + b; a, b are fit coefficients)

Table S26. RP-HPLC separation conditions of compounds

**Compound**	**Wavelength** (nm)	**MeCN/H$_2$O**	**Flow Rate** (mL/min)	**T$_r$** (min)
(±)-Flurbiprofen	254	80 : 20	0.2	5.555
27	220	90 : 10	1.0	1.440
(±)-Indoprofen	210	90 : 10	0.2	5.384
37	210	90 : 10	0.2	5.755
(*2S*,*4R*)-Sacubitril	210	90 : 10	0.2	5.708
70	220	90 : 10	0.2	6.799

Chromatographic conditions: Agilent Ltd Poroshell 120 (EC-C18, 4.6 × 100 mm, 2.7 μm), MeCN/H$_2$O, 40 °C

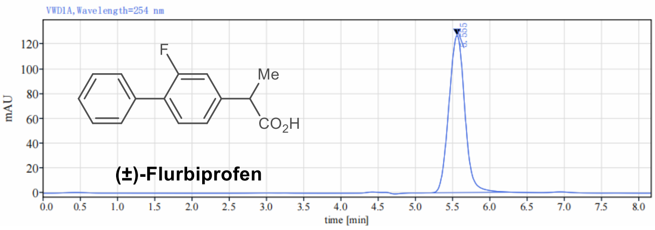

rettime [min]	type	width [min]	area	height	area%
5.555	MM m	1.06	1883.73	126.63	100.00

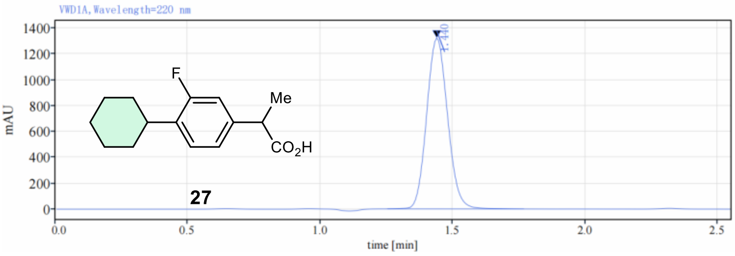

rettime [min]	type	width [min]	area	height	area%
1.440	MM m	0.52	7247.36	1321.62	100.00

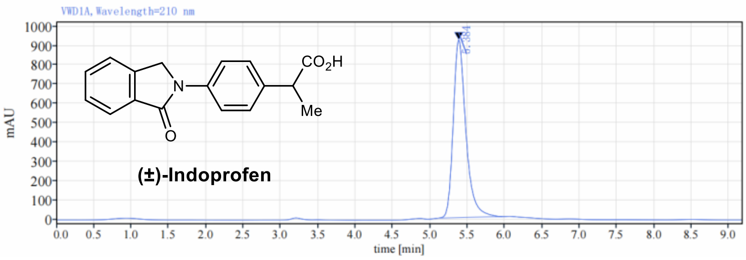

rettime [min]	type	width [min]	area	height	area%
5.384	MM m	0.86	10815.79	918.06	100.00

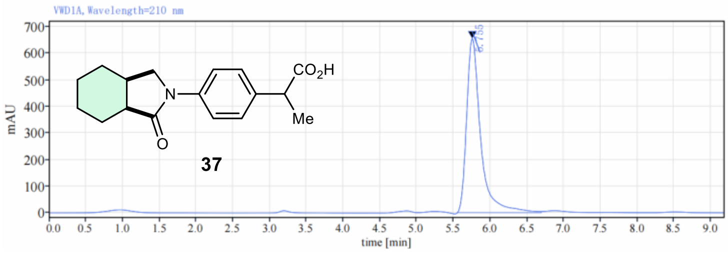

rettime [min]	type	width [min]	area	height	area%
5.755	MM m	1.14	8677.62	654.85	100.00

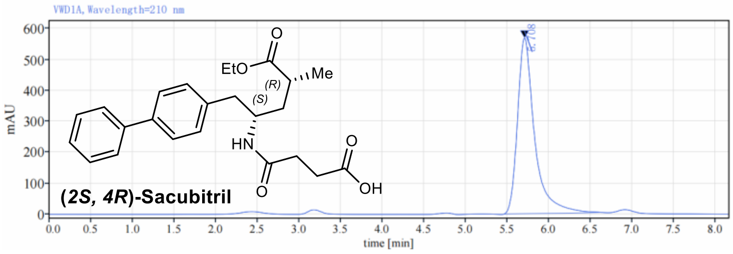

rettime [min]	type	width [min]	area	height	area%
5.708	MM m	1.18	8113.46	568.71	100.00

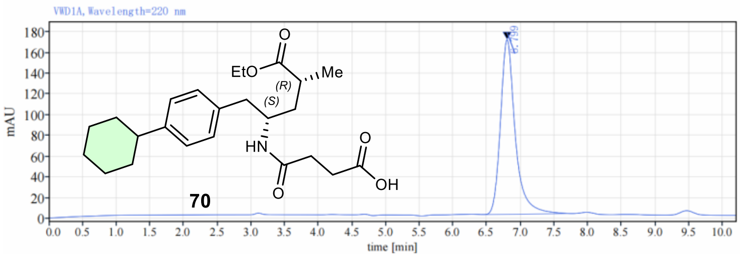

rettime [min]	type	width [min]	area	height	area%
6.799	MM m	1.39	2560.11	168.21	100.00

**Table S27**. Standard equation and linearity of compounds (n = 7)			
**Compound**	**Linear Regression Equation**	R^2	**Linearity Range** (mg/mL)
(±)-Flurbiprofen	Y = 197293.665•X − 59.181	0.99917	0.005 - 0.033
**27**	Y = 15834.360•X − 16.576	0.99908	0.010 - 0.041
(±)-Indoprofen	Y = 334318.323•X − 172.298	0.99977	0.005 - 0.033
**37**	Y = 262994.605•X + 131.012	0.99942	0.005 - 0.033
(2S,4R)-Sacubitril	Y = 326954.136•X + 263.693	0.99946	0.005 - 0.033
**70**	Y = 113686.699•X + 93.459	0.99957	0.005 - 0.033

3). Measurement of $logP_{o/w}$.

The compounds (2.0, 3.0, 5.0 or 10.0 mg) and the solution (2.0 mL) of n-octanol and purified water (*v*/*v* = 1:1) were added to a 5 ml sample vial. The mixture was then stirred at 600 rpm for 24 hours at 30 °C. After standing for stratification, the samples were separated into oil phase and water phase and collected by 0.22μm microporous filter membrane. The organic and aqueous phases were extracted separately, diluted with acetonitrile and analyzed by RP-HPLC in three parallel experiments. The peak areas were recorded, and the mass concentration (X) was calculated based on the standard curve. The mass concentration of compound in the organic and aqueous phases was then calculated with the following equation: C = X•D, (D is the dilution factor). The $logP_{o/w}$ of the compound was calculated with the following equation: $P_{o/w}$ = C(oil)/C(aqueous).

**Table S28**. n-Octanol-water partition coefficients of 14 compounds (n = 3)				
**Compound**	**C_0** (mg/ml)	**C(oil)** (mg/mL)	**C(aqueous)** (mg/mL)	**$LogP_{o/w}$**
(±)-Flurbiprofen	5.0 (1:1)	1.858	0.135	1.138
**27**	5.0 (1:1)	2.453	0.071	1.541
(±)-Indoprofen	2.0 (1:1)	0.943	0.091	1.018
**37**	2.0 (1:1)	0.897	0.138	0.813
(2S,4R)-Sacubitril	2.0 (1:1)	1.368	0.047	1.467
**70**	2.0 (1:1)	0.737	0.030	1.385
(±)-Prilocaine	3.0 (1:1)	1.306	0.124	1.021
**15**	3.0 (1:1)	1.569	0.064	1.392
(±)-Naftopidil	3.0 (1:1)	1.697	0.016	2.036
**28**	3.0 (1:1)	1.761	0.014	2.099
(±)-Propranolol	5.0 (1:1)	2.871	0.015	2.290
**23**	5.0 (1:1)	2.697	0.007	2.617
(R)-Cinacalcet	10.0 (1:1)	8.962	0.0002	4.613
**39**	10.0 (1:1)	9.541	0.0001	4.788

11. References

1. Harris, R. K.; Becker, E. D.; Cabral De Menezes, S. M.; Goodfellow, R.; Granger, P., NMR nomenclature. Nuclear spin properties and conventions for chemical shifts (IUPAC recommendations 2001). *Pure Appl. Chem.* **2001,** *73* (11), 1795-1818.
2. Gelis, C.; Heusler, A.; Nairoukh, Z.; Glorius, F., Catalytic Transfer Hydrogenation of Arenes and Heteroarenes. *Chem. - Eur. J.* **2020,** *26* (62), 14090-14094.
3. Wang, Y.; Chang, Z.; Hu, Y.; Lin, X.; Dou, X., Mild and Selective Rhodium-Catalyzed Transfer Hydrogenation of Functionalized Arenes. *Org. Lett.* **2021,** *23* (5), 1910-1914.
4. Lavoie, C. M.; MacQueen, P. M.; Rotta-Loria, N. L.; Sawatzky, R. S.; Borzenko, A.; Chisholm, A. J.; Hargreaves, B. K. V.; McDonald, R.; Ferguson, M. J.; Stradiotto, M., Challenging nickel-catalysed amine arylations enabled by tailored ancillary ligand design. *Nat. Commun.* **2016,** *7* (1), 11073.
5. Zeng, Y.-F.; Li, Y.-N.; Zhou, M.-X.; Han, S.; Guo, Y.; Wang, Z., Metal-Free Hydrogenation of N-Heterocycles with Trimethylamine Borane and TFA in Aqueous Solution. *Adv. Synth. Catal.* **2022,** *364* (21), 3664-3669.
6. Abe, M.; Munakata, H.; Abe, K.; Saito, T.; Horiguchi, Y.; Nojima, H.; Taguchi, K., Effects of 1-cyclohexyl- and 1-cyclohexyl-N-propargyl-1,2,3,4-tetrahydroisoquinoline on dopaminergic spontaneous discharge in nigral neurons of rats. *Brain Res. Bull.* **2016,** *121*, 201-208.
7. Kim, S.; Chen, J.; Cheng, T.; Gindulyte, A.; He, J.; He, S.; Li, Q.; Shoemaker, B. A.; Thiessen, P. A.; Yu, B.; Zaslavsky, L.; Zhang, J.; Bolton, E. E., Zhang, J.; PubChem 2023 update. *Nucleic Acids Res.* **2023,** *51* (D1), D1373-D1380.
8. Gaulton, A.; Hersey, A.; Nowotka, M.; Bento, A. P.; Chambers, J.; Mendez, D.; Mutowo, P.; Atkinson, F.; Bellis, L. J.; Cibrián-Uhalte, E.; Davies, M.; Dedman, N.; Karlsson, A.; Magariños, M. P.; Overington, J. P.; Papadatos, G.; Smit, I.; Leach, A. R., The ChEMBL database in 2017. *Nucleic Acids Res.* **2017,** *45* (D1), D945-D954.
9. Davies, M.; Nowotka, M.; Papadatos, G.; Dedman, N.; Gaulton, A.; Atkinson, F.; Bellis, L.; Overington, J. P., ChEMBL web services: streamlining access to drug discovery data and utilities. *Nucleic Acids Res.* **2015,** *43* (W1), W612-W620.
10. Ling, Y.; Hao, Z.-Y.; Liang, D.; Zhang, C.-L.; Liu, Y.-F.; Wang, Y., The Expanding Role of

Pyridine and Dihydropyridine Scaffolds in Drug Design. *Drug Des., Dev. Ther.* **2021,** *15*, 4289-4338.

11. Wiesenfeldt, M. P.; Nairoukh, Z.; Li, W.; Glorius, F., Hydrogenation of fluoroarenes: Direct access to all-cis-(multi)fluorinated cycloalkanes. *Science* **2017,** *357* (6354), 908-912.
12. Nairoukh, Z.; Wollenburg, M.; Schlepphorst, C.; Bergander, K.; Glorius, F., The formation of all-cis-(multi)fluorinated piperidines by a dearomatization–hydrogenation process. *Nat. Chem.* **2019,** *11* (3), 264-270.
13. Wagen, C. C.; McMinn, S. E.; Kwan, E. E.; Jacobsen, E. N., Jacobsen, Screening for generality in asymmetric catalysis. *Nature* **2022,** *610* (7933), 680-686.
14. Daina, A.; Michielin, O.; Zoete, V., SwissADME: a free web tool to evaluate pharmacokinetics, drug-likeness and medicinal chemistry friendliness of small molecules. *Sci. Rep.* **2017,** *7*, 42717.
15. Mansouri, K.; Karmaus, A. L.; Fitzpatrick, J.; Patlewicz, G.; Pradeep, P.; Alberga, D.; Alepee, N.; Allen, T. E. H.; Allen, D.; Alves, V. M.; Andrade, C. H.; Auernhammer, T. R.; Ballabio, D.; Bell, S.; Benfenati, E.; Bhattacharya, S.; Bastos, J. V.; Boyd, S.; Brown, J. B.; Capuzzi, S. J.; Chushak, Y.; Ciallella, H.; Clark, A. M.; Consonni, V.; Daga, P. R.; Ekins, S.; Farag, S.; Fedorov, M.; Fourches, D.; Gadaleta, D.; Gao, F.; Gearhart, J. M.; Goh, G.; Goodman, J. M.; Grisoni, F.; Grulke, C. M.; Hartung, T.; Hirn, M.; Karpov, P.; Korotcov, A.; Lavado, G. J.; Lawless, M.; Li, X.; Luechtefeld, T.; Lunghini, F.; Mangiatordi, G. F.; Marcou, G.; Marsh, D.; Martin, T.; Mauri, A.; Muratov, E. N.; Myatt, G. J.; Nguyen, D.-T.; Nicolotti, O.; Note, R.; Pande, P.; Parks, A. K.; Peryea, T.; Polash, A. H.; Rallo, R.; Roncaglioni, A.; Rowlands, C.; Ruiz, P.; Russo, D. P.; Sayed, A.; Sayre, R.; Sheils, T.; Siegel, C.; Silva, A. C.; Simeonov, A.; Sosnin, S.; Southall, N.; Strickland, J.; Tang, Y.; Teppen, B.; Tetko, I. V.; Thomas, D.; Tkachenko, V.; Todeschini, R.; Toma, C.; Tripodi, I.; Trisciuzzi, D.; Tropsha, A.; Varnek, A.; Vukovic, K.; Wang, Z.; Wang, L.; Waters, K. M.; Wedlake, A. J.; Wijeyesakere, S. J.; Wilson, D.; Xiao, Z.; Yang, H.; Zahoranszky-Kohalmi, G.; Zakharov, A. V.; Zhang, F. F.; Zhang, Z.; Zhao, T.; Zhu, H.; Zorn, K. M.; Casey, W.; Kleinstreuer, N. C., CATMoS: Collaborative Acute Toxicity Modeling Suite. *Environ. Health Perspect.* **2021,** *129* (4), 47013.
16. National Library of Medicine. ChemIDplus, chem.nlm.nih.gov/chemidplus/ (2022).
17. European Chemicals Agency. IUCLID6, iuclid6.echa.europa.eu/de/home (2022).

18. Dimitrov, S. D.; Diderich, R.; Sobanski, T.; Pavlov, T. S.; Chankov, G. V.; Chapkanov, A. S.; Karakolev, Y. H.; Temelkov, S. G.; Vasilev, R. A.; Gerova, K. D.; Kuseva, C. D.; Todorova, N. D.; Mehmed, A. M.; Rasenberg, M.; Mekenyan, O. G., QSAR Toolbox - workflow and major functionalities. *SAR QSAR Environ. Res.* **2016,** *27* (3), 203-219.

19. Sandfort, F.; Strieth-Kalthoff, F.; Kuehnemund, M.; Beecks, C.; Glorius, F., A Structure-Based Platform for Predicting Chemical Reactivity. *Chem* **2020,** *6* (6), 1379-1390.

20. J. D. Hunter, Matplotlib: A 2D Graphics Environment. *Comput. Sci. Eng.* **2017,** *9*, 90-95.

21. Liu, D.-H.; Nagashima, K.; Liang, H.; Yue, X.-L.; Chu, Y.-P.; Chen, S.; Ma, J., Chemoselective Quinoline and Isoquinoline Reduction by Energy Transfer Catalysis Enabled Hydrogen Atom Transfer. *Angew. Chem. Int. Ed.* **2023,** *62* (48), e202312203.

22. Knights, K. M.; Stresser, D. M.; Miners, J. O.; Crespi, C. L., In Vitro Drug Metabolism Using Liver Microsomes. *Curr. Protoc. Pharmacol.* **2016,** *74*, 7.8.1-7.8.24.

23. Pan, X.; Wang, H.; Li, C.; Zhang, J. Z. H.; Ji, C., MolGpka: A Web Server for Small Molecule pKa Prediction Using a Graph-Convolutional Neural Network. *J. Chem. Inf. Model.* **2021,** *61* (7), 3159-3165.
